# Supplementary material for: Chiral phosphoric acid-catalyzed stereodivergent synthesis of trisubstituted allenes and computational mechanistic studies
Source: Nat Commun. 2020 Nov 2;11:5527. doi: 10.1038/s41467-020-19294-8 (PMC7608664; doi:10.1038/s41467-020-19294-8)
Supplement: Supplementary file 1 — Supplementary Information [file 41467_2020_19294_MOESM1_ESM.pdf]

Supplementary Information

Chiral phosphoric acid-catalyzed stereodivergent synthesis of trisubstituted allenes and computational mechanistic studies

Wang et al.

Table of Contents

General Information: 3

Supplementary Table S1. Screening of the R group on oxazolones and optimizations of solvents 4

Synthesis of catalyst *(S)*-B2: 7

Synthesis of substrates: 7

Asymmetric synthesis of products: 14

Mechanistic Study 41

Derivatizations of chiral products: 43

Computational Studies 50

X-Ray Structures 62

HPLC traces: 72

NMR Spectrums: 127

Supplementary References 209

General Information:

Unless otherwise noted, all commercial reagents were used without further purification. Dichloromethane, toluene, ether, THF were purified by passage through an activated alumina column under argon. Thin-layer chromatography (TLC) analysis of reaction mixtures was performed using Huanghai silica gel HSGF254 TLC plates, and visualized under UV or by staining with ceric ammonium molybdate or potassium permanganate. Flash column chromatography was carried out on Huanghai Silica Gel HHGJ-300, 300-400 mesh. Nuclear magnetic resonance (NMR) spectra were recorded using Bruker Avance III HD spectrometer (FT, 400 MHz for ^{1}H, 101 MHz for ^{13}C). ^{1}H and ^{13}C chemical shifts are reported in ppm downfield of tetramethylsilane and referenced to residual solvent peak ($CHCl_3$; δH = 7.26 and δC = 77.16, CD_3OD, δH = 3.31 and δC = 49.00, $(CD_3)_2CO$, δH = 2.05 and δC = 29.84). Multiplicities are reported using the following abbreviations: s = singlet, d = doublet, t = triplet, q = quartet, m = multiplet, br = broad resonance. FT-IR spectra were recorded on PerkinElmer Frontier FT-IR Spectrometer, and absorption frequencies are reported in reciprocal centimeters (cm^{-1}). Mass spectral data were obtained from the Agilent Technologies 6230 TOF LC/MS spectrometer in electrospray ionization (ESI^{+}) mode. Optical rotations were measured with an Autopol V Plus/VI digital polarimeter. X-Ray structure analyses were performed using a Bruker D8 Venture X-ray single crystal diffractometer. Enantiomeric excesses were determined on an Agilent 1260 Chiral HPLC using IA, IB, IC columns. The racemic products were synthesized by using (±)-**A4** as catalyst.

Supplementary Table 1. Screening of the R group on oxazolones and optimizations of solvents

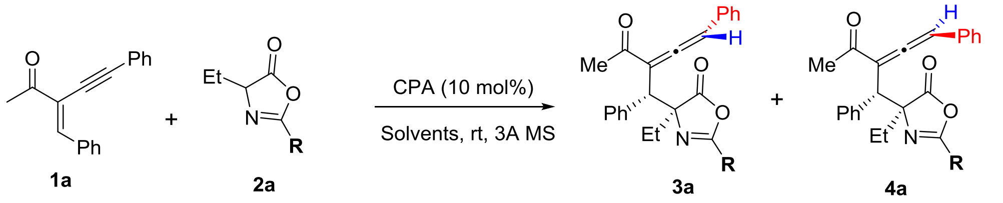

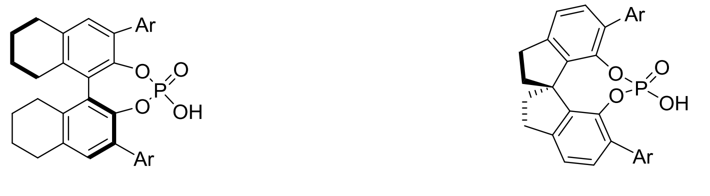

B2, Ar = 1-(4-Me-napthyl) **C1**, Ar = 1-naphthyl

Entry[a]	R	Catalyst	Solvents	yield (%)b	d.r. (**3a**:**4a**)[b]	ee (%)[c]
1	4-OMe-Ph	**B2**	toluene	91	10:1	87/-
2	Ph	**B2**	toluene	83	1:1	65/-
3	4-F-Ph	**B2**	toluene	87	5:1	87/-
4	4-CF_3-Ph	**B2**	toluene	97	9:1	90/-
5	2,6-$(OMe)_2$-Ph	**B2**	toluene	30	2:1	80/-
6	3,5-$(OMe)_2$-Ph	**B2**	toluene	99	12:1	91/-
7	3,5-$(OMe)_2$-Ph	**C1**	toluene	80	1:9	-/94
8	3,5-$(OMe)_2$-Ph	**B2**	xylenes	92	13:1	90/-
9	3,5-$(OMe)_2$-Ph	**C1**	Xylenes	68	1:6	-/87
10	3,5-$(OMe)_2$-Ph	**B2**	DCM	71%	2.7： 1	97/-
11	3,5-$(OMe)_2$-Ph	**C1**	DCM	64	1:8	-/90
12	3,5-$(OMe)_2$-Ph	**B2**	CCl_4	98	20:1	91/-
13	3,5-$(OMe)_2$-Ph	**C1**	CCl_4	85	1:12	-/98
14[d]	3,5-$(OMe)_2$-Ph	**B2**	CCl_4	67	16:1	90/-
15e	3,5-$(OMe)_2$-Ph	**B2**	CCl_4	63	9:1	94/-

[a]Reactions were carried with **1a** (0.15 mmol), **2a** (0.1 mmol), **cat** (0.01 mmol), solvents (1 mL) at ambient temperature for 24 h under N_2 atmosphere. [b]Yields and d.r. value were determined by crude ^{1}H NMR. [c]Ee values were determined by HPLC analysis on a chiral stationary phase. [d]Without 3 Å MS. [e]Under air.

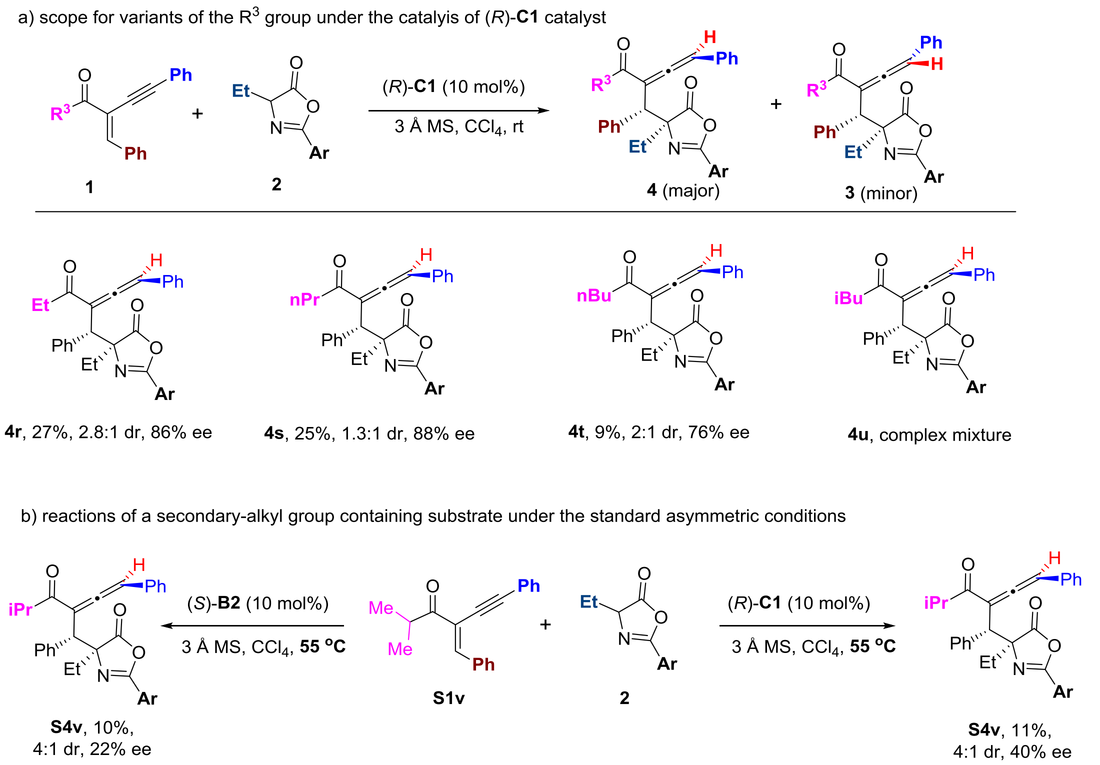

Supplementary Figure 1. Scope for asymmetric synthesis of allene 4 with variants of the R^3 group of substrate 1. Unless otherwise noted, reactions were performed with **1** (0.15 mmol), **2** (0.1 mmol), CPA (*R*)-**C1** (0.01 mmol) and 3 Å MS (100 mg) in CCl_4 (0.5 mL) at RT for 24 h. Yields were combined isolated yields of allenes **3** and **4**. Dr values were determined by crude ^1H NMR analysis. Ee values were determined by HPLC analysis on a chiral stationary phase. Ar = 3,5-dimethoxyphenyl.

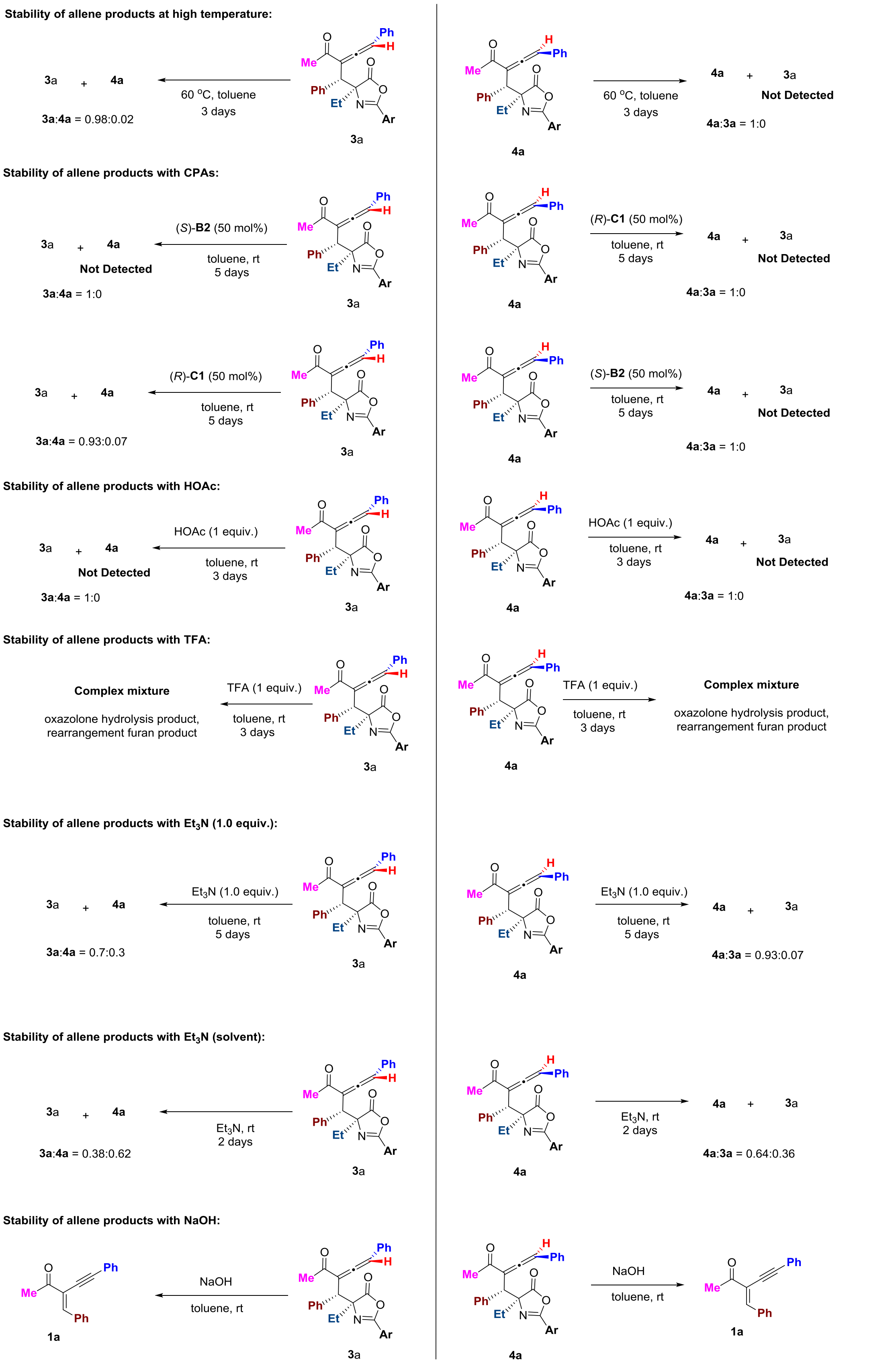

Supplementary Figure 2. Configurational stabilities of the allene products under various conditions.

Synthesis of catalyst *(S)*-B2:

Catalyst (*S*)-**B2** was synthesized by adopting the procedure reported by Akiyama[1].

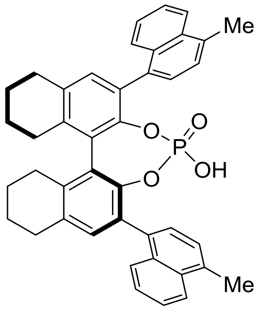

^{1}H NMR (400 MHz, Acetone) δ 8.02 (t, *J* = 9.6 Hz, 2H), 7.91 – 7.23 (m, 10H), 7.14 (d, *J* = 7.6 Hz, 2H), 3.12 – 2.80 (m, 6H), 2.68 (s, 6H), 2.60 – 2.43 (m, 2H), 2.01 – 1.83 (m, 6H), 1.82 – 1.66 (m, 2H). ^{13}C NMR (126 MHz, $CDCl_3$) δ 144.2, 144.2, 144.0, 143.9, 143.8, 143.8, 137.3, 137.3, 137.2, 137.2, 135.0, 135.0, 135.0, 134.4, 134.4, 133.9, 133.7, 133.7, 133.0, 132.9, 132.9, 132.9, 132.4, 132.3, 132.3, 132.20, 132.1, 132.0, 132.0, 131.8, 131.6, 130.8, 130.8, 130.6, 130.6, 130.5, 130.4, 130.4, 128.1, 128.0, 127.9, 127.5 , 127.5, 127.1, 127.1, 126.7, 126.7, 126.5, 126.4, 126.3, 126.2, 125.9, 125.8, 125.4, 125.4, 125.2, 125.1, 125.0, 124.9, 124.8, 124.8, 124.2, 124.2, 123.9, 123.8, 29.3, 29.3, 28.2, 28.1, 28.1, 28.1, 22.8, 22.8, 22.8, 22.8, 22.7, 22.7, 19.4, 19.3, 19.2, 19.1. ^{31}P NMR (162 MHz, Acetone) δ -2.03, -2.23. HRMS-ESI (m/z) calculated for $C_{42}H_{38}O_4P^+$: 637.2502; found: 637.2495, $[\alpha]_D^{25}$ = 191.90 (c 1.0, $CHCl_3$). IR：ν = 3051, 2932, 2858, 1715, 1586, 1504 cm^{-1}

Synthesis of substrates:

Substrate **1a**[2], **1b**[3], **1d**[2], **1e**[4], **1f**[3], **1k**[3], **1l**[3], **1n**[3], **1o**[5], **1q**[6], **1r**[7] and **1t**[8] were synthesized according previous reports.

General procedure A[3] for synthesis of 1,3-enynes 1:

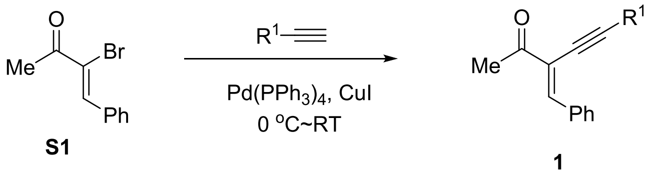

S1 **1**

To a solution of **S1**[3] in anhydrous THF (0.2 M) was added $Pd(PPh_3)_2Cl_2$ (0.02 equiv.) and CuI (0.04 equiv.) at rt. The reaction mixture was cooled to 0 °C and degassed with argon. Alkyne (1.5 equiv.) and diisopropylamine (3.0 equiv.) was added and the mixture was stirred overnight at ambient temperature.

Upon completion, the reaction mixture was diluted with H_2O (40 mL). The aqueous phase was extracted with Et_2O for 3 times, washed with brine and the combined organic phase was dried over Na_2SO_4, filtered and concentrated under vacuum to give a residue, which was purified by flash column chromatography.

(*E*)-3-benzylidene-5-(4-chlorophenyl)pent-4-yn-2-one (**1c**)

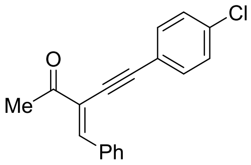

^{1}H NMR (400 MHz, $CDCl_3$) δ 8.06 (dd, *J* = 6.5, 2.8 Hz, 2H), 7.83 (s, 1H), 7.51 – 7.41 (m, 5H), 7.37 (d, *J* = 8.5 Hz, 2H), 2.60 (s, 3H). ^{13}C NMR (101 MHz, $CDCl_3$) δ 196.1, 143.6, 135.2, 134.5, 132.7, 131.0, 130.8, 129.1, 128.8, 121.4, 119.9, 98.0, 88.0, 28.3. IR: ν = 3056, 2196, 1899, 1721, 1692, 1593, 1581, 1562cm^{-1}. HRMS-ESI (m/z) calculated for $C_{18}H_{14}ClO^{+}$: 281.0728; found: 281.0723 $[M+H]^{+}$.

Methyl (*E*)-3-(3-benzylidene-4-oxopent-1-yn-1-yl)benzoate (**1g**)

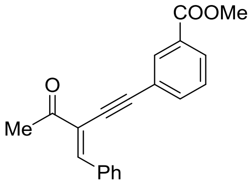

^{1}H NMR (400 MHz, $CDCl_3$) δ 8.21 (s, 1H), 8.13 – 8.00 (m, 3H), 7.85 (s, 1H), 7.71 (d, *J* = 7.7 Hz, 1H), 7.52 – 7.39 (m, 4H), 3.95 (s, 3H), 2.62 (s, 3H). ^{13}C NMR (101 MHz, $CDCl_3$) δ 196.0, 166.3, 143.8, 135.6, 134.5, 132.5, 131.1, 130.9, 130.8, 129.9, 128.9, 128.8, 97.9, 87.9, 52.5, 28.3. IR: ν = 3061, 2951, 1722, 1692, 1599, 1583, 1563cm^{-1}. HRMS-ESI (m/z) calculated for $C_{20}H_{17}O_3^{+}$: 305.1172; found: 305.1169 $[M+H]^{+}$.

(*E*)-3-benzylidene-5-(2-chlorophenyl)pent-4-yn-2-one (**1h**)

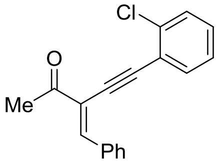

^{1}H NMR (400 MHz, $CDCl_3$) δ 8.16 (dd, *J* = 6.6, 2.8 Hz, 2H), 7.88 (s, 1H), 7.57 (dd, *J* = 7.2, 2.1 Hz, 1H), 7.50 – 7.40 (m, 4H), 7.36 – 7.27 (m, 2H), 2.67 (s, 3H). ^{13}C NMR (101 MHz, $CDCl_3$) δ 196.2,

143.8, 136.1, 134.4, 133.4, 131.1, 131.0, 130.0, 129.7, 128.7, 126.8, 123.0, 119.6, 96.2, 92.0, 28.7. IR: ν = 3060, 2196, 1692, 1591, 1581, 1562cm^{-1}. HRMS-ESI (m/z) calculated for $C_{18}H_{14}ClO^+$: 281.0728; found: 281.0724 [M+H]$^+$.

(*E*)-3-benzylidene-5-(thiophen-3-yl)pent-4-yn-2-one (**1i**)

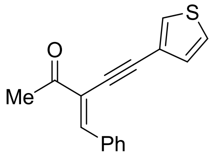

^{1}H NMR (400 MHz, CDCl$_3$) δ 8.07 (dd, *J* = 6.5, 2.8 Hz, 2H), 7.81 (s, 1H), 7.61 – 7.55 (m, 1H), 7.48 – 7.40 (m, 3H), 7.36 (dd, *J* = 4.9, 3.0 Hz, 1H), 7.23 (dd, *J* = 5.0, 0.8 Hz, 1H), 2.60 (s, 3H). ^{13}C NMR (101 MHz, CDCl$_3$) δ 196.4, 143.0, 134.6, 130.9, 130.8, 129.6, 129.3, 128.7, 126.0, 122.0, 120.1, 94.6, 86.6, 28.3. IR: ν = 3054, 2199, 1692, 1584, 1566 cm^{-1}. HRMS-ESI (m/z) calculated for $C_{16}H_{13}OS^+$: 253.0682; found: 253.0678 [M+H]$^+$.

(*E*)-3-benzylidene-5-(cyclohex-1-en-1-yl)pent-4-yn-2-one (**1j**)

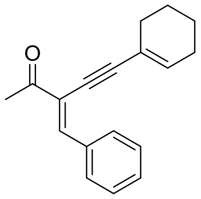

^{1}H NMR (400 MHz, CDCl$_3$) δ 8.11 – 7.95 (m, 2H), 7.70 (s, 1H), 7.40 (d, *J* = 5.5 Hz, 3H), 6.27 (s, 1H), 2.53 (s, 3H), 2.27 (d, *J* = 2.0 Hz, 2H), 2.22 – 2.14 (m, 2H), 1.79 – 1.59 (m, 4H). ^{13}C NMR (101 MHz, CDCl$_3$) δ 196.7, 141.5, 136.5, 134.8, 130.7, 130.5, 128.5, 120.8, 120.4, 101.5, 84.8, 28.7, 28.2, 26.0, 22.3, 21.5. IR: ν = 3058, 3028, 2928, 2859, 2180, 1691, 1625, 1582, 1560cm^{-1}. HRMS-ESI (m/z) calculated for $C_{18}H_{10}O^+$: 251.1430; found: 251.1425 [M+H]$^+$.

General procedure B for synthesis of 1,3-enynes 1:

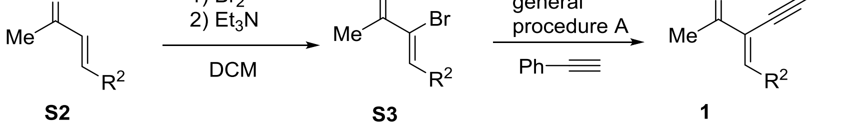

A solution of bromine (1.05 equiv.) in DCM (1 M) was added dropwise to a suspension of α,β-unsaturated ketone **S2** (1.0 equiv.) in DCM (1 M) at 0 ^{o}C. The mixture was stirred for 30 min

before adding a solution of triethylamine (1.2 equiv.) in DCM (1 M) dropwisely at 0 ^{o}C. After stirring overnight at ambient temperature, the reaction mixture was diluted with H_2O (30 mL). The aqueous phase was extracted with Et_2O for 3 times and the combined organic phase was dried over Na_2SO_4, filtered and concentrated under vacuum to give a residue, which was purified by flash column chromatography to give **S3**.

The coupling of **S3** with phenylacetylene for the synthesis of **1** adopted the **general procedure A**.

(E)-3-(4-fluorobenzylidene)-5-phenylpent-4-yn-2-one (**1m**)

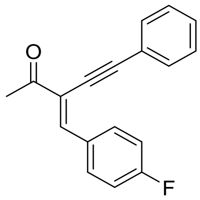

^{1}H NMR (400 MHz, $CDCl_3$) δ 8.11 (dd, *J* = 8.6, 5.6 Hz, 2H), 7.78 (s, 1H), 7.60 – 7.49 (m, 2H), 7.46 – 7.34 (m, 3H), 7.13 (t, *J* = 8.6 Hz, 2H), 2.61 (s, 3H). ^{13}C NMR (101 MHz, $CDCl_3$) δ 196.2, 164.0 (d, *J* = 253.3 Hz), 141.6, 132.9 (d, *J* = 8.6 Hz), 131.5, 130.94(d, *J* = 3.3 Hz), 129.2, 128.8, 122.8, 119.6 (d, *J* = 2.3 Hz), 115.9(d, *J* = 21.7 Hz), 99.3, 86.9, 28.3. ^{19}F NMR (376 MHz, $CDCl_3$) δ -107.71.

(*E*)-3-(naphthalen-2-ylmethylene)-5-phenylpent-4-yn-2-one (**1p**)

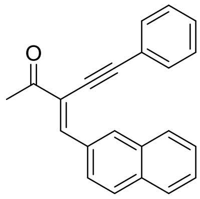

^{1}H NMR (400 MHz, $CDCl_3$) δ 8.57 (s, 1H), 8.25 (dd, *J* = 8.6, 1.3 Hz, 1H), 7.99 (s, 1H), 7.91 – 7.79 (m, 3H), 7.61 (dd, *J* = 6.4, 2.9 Hz, 2H), 7.58 – 7.48 (m, 2H), 7.48 – 7.37 (m, 3H), 2.66 (s, 3H). ^{13}C NMR (101 MHz, $CDCl_3$) δ 196.4, 143.1, 134.5, 133.2, 132.3, 132.1, 131.5, 129.1, 129.0, 128.8, 128.3, 127.9, 127.8, 127.0, 126.7, 123.0, 120.1, 99.4, 87.5, 28.4. IR: ν = 3054, 1691, 1597, 1576, 1564cm^{-1}. HRMS-ESI (m/z) calculated for $C_{22}H_{17}O^{+}$: 297.1274; found: 297.1268 $[M+H]^{+}$.

General procedure C for synthesis of 1,3-enynes 1:

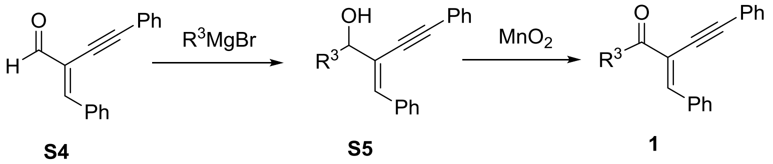

To a solution of **S4**[9] (1.0 equiv.) in dry THF (1.0 M) cooled to –78 ℃ was added R^3MgBr (1.5 equiv.). The reaction was stirred at the same temperature for 10 min and then stirred overnight at room temperature. After completion, this reaction was quenched with saturated NH_4Cl (5 mL) and the resulting mixture was extracted with EtOAc for 3 times. The organic layer was washed with brine, dried over anhydrous Na_2SO4, filtered and concentrated to give a residue, which was purified by flash column chromatography to give alcohol **S5**.

To the solution of **S5** (1.0 equiv.) in dry THF (1.0 M) was added MnO_2 (20 equiv) at RT. After stirring at room temperature for 24 h, the reaction mixture was filtered through a short pad of Celite, which was rinsed with EtOAc. The combined organic layer was concentrated under reduced pressure, and the residue was purified by flash column chromatography to give substrate **1**.

(*E*)-3-benzylidene-1-phenylhept-1-yn-4-one (**1s**)

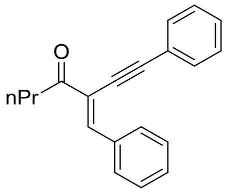

^{1}H NMR (400 MHz, $CDCl_3$) δ 8.15 – 8.05 (m, 2H), 7.85 (s, 1H), 7.62 – 7.51 (m, 2H), 7.51 – 7.34 (m, 6H), 3.00 (t, *J* = 7.2 Hz, 2H), 1.78 (dd, *J* = 14.6, 7.3 Hz, 2H), 1.03 (t, *J* = 7.4 Hz, 3H). ^{13}C NMR (101 MHz, $CDCl_3$) δ 198.5, 142.9, 134.8, 131.5, 130.8, 130.7, 129.0, 128.7, 128.7, 123.0, 119.9, 99.2, 87.0, 42.4, 17.9, 14.0. IR: ν = 2964, 1693, 1583, 1565cm^{-1}. HRMS-ESI (m/z) calculated for $C_{20}H_{19}O^+$: 275.1430; found: 275.1423 $[M+H]^+$.

(*E*)-3-benzylidene-6-methyl-1-phenylhept-1-yn-4-one (**1u**)

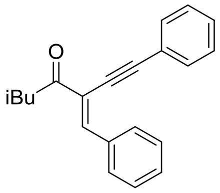

^{1}H NMR (400 MHz, $CDCl_3$) δ 8.16 – 8.04 (m, 2H), 7.84 (s, 1H), 7.55 (dd, *J* = 6.4, 2.9 Hz, 2H), 7.43 (m, 6H), 2.88 (d, *J* = 6.8 Hz, 2H), 2.32 (qt, *J* = 13.0, 6.4 Hz, 1H), 1.02 (d, *J* = 6.7 Hz, 6H). ^{13}C NMR (126 MHz, Chloroform-*d*) δ 198.2, 143.0, 134.8, 131.5, 130.9, 130.8, 129.0, 128.8, 128.7, 123.0, 120.2, 99.2, 87.1, 49.2, 25.3, 22.9. IR: ν = 2961, 1689, 1598, 1583, 1565cm^{-1}. HRMS-ESI (m/z) calculated for $C_{21}H_{21}O^+$: 289.1587; found: : 289.1579 $[M+H]^+$.

General procedure for the synthesis of oxazolone 2[10]:

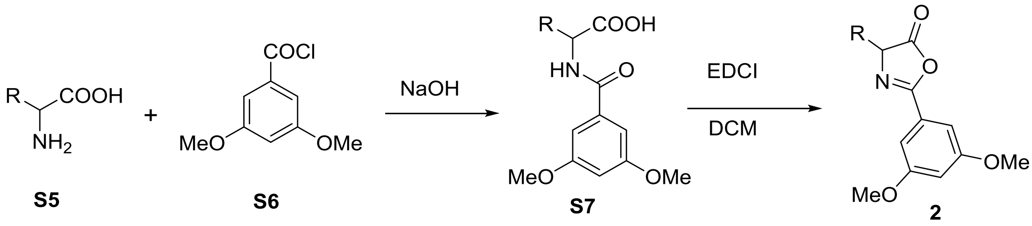

The amino acid **S5** (1 equiv) was dissolved in a 2 M solution of NaOH (4 equiv) in water. After cooling to 0 ℃, and benzoyl chloride **S6** (1.1 equiv) was added dropwisely. The resulting mixture was allowed to warm to room temperature and stir for 24 h. Aqueous 6 M HCl solution was added to the reaction mixture until pH < 2 (as monitored by pH paper), and the solution was extracted with DCM for 3 times. The combined organic layers were then dried over Na_2SO_4, filtered, and concentrated to give the acylated intermediate **S7**, which was used directly in the next step.

To a suspension of **S7** (1 equiv) in dry DCM (0.1 M) under N_2 at 0 ℃ was added EDC HCl (1.3 equiv). After completion of the reaction, the mixture was diluted with DCM, and washed successively with H_2O then dried over anhydrous Na_2SO_4 and concentrated under reduced pressure to give a residue, which was purified by flash column chromatography to give oxazolone **2**.

2-(3,5-dimethoxyphenyl)-4-ethyloxazol-5(4H)-one (**2a**)

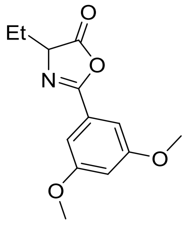

^{1}H NMR (400 MHz, $CDCl_3$) δ 7.15 (d, *J* = 2.2 Hz, 2H), 6.66 (t, *J* = 2.1 Hz, 1H), 4.40 (t, *J* = 6.0 Hz, 1H), 3.84 (d, *J* = 7.4 Hz, 6H), 2.16 – 2.02 (m, 1H), 1.97 (dd, *J* = 14.1, 7.0 Hz, 1H), 1.05 (t, *J* = 7.4 Hz, 3H). ^{13}C NMR (101 MHz, $CDCl_3$) δ 178.4, 161.8, 161.1, 127.7, 105.9, 105.5, 66.6, 55.8, 25.0, 9.6. IR:

ν = 3380, 2969, 1826, 1711, 1654, 1593, 1540 cm^{-1}. HRMS-ESI (m/z) calculated for $C_{13}H_{16}NO_4^+$: 250.1074; found: 250.1067 $[M+H]^+$.

4-Butyl-2-(3,5-dimethoxyphenyl)oxazol-5(4H)-one (**2v**)

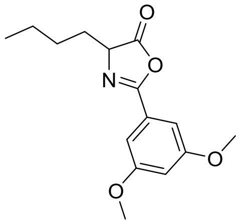

^{1}H NMR (400 MHz, $CDCl_3$) δ 7.13 (s, 2H), 6.64 (d, *J* = 2.1 Hz, 1H), 4.40 (t, *J* = 6.0 Hz, 1H), 3.83 (s, 6H), 2.01 (dt, *J* = 10.6, 6.9 Hz, 1H), 1.95 – 1.77 (m, 1H), 1.57 – 1.29 (m, 4H), 0.92 (t, *J* = 6.9 Hz, 3H). ^{13}C NMR (101 MHz, $CDCl_3$) δ 178.6, 161.6, 161.1, 127.7, 105.8, 105.5, 65.6, 55.8, 31.4, 27.4, 22.5, 13.9. IR: ν = 2962, 1825, 1652, 1594 cm^{-1}. HRMS-ESI (m/z) calculated for $C_{15}H_{20}NO_4^+$: 278.1387; found: 278.1382 $[M+H]^+$.

4-benzyl-2-(3,5-dimethoxyphenyl)oxazol-5(4H)-one (**2w**)

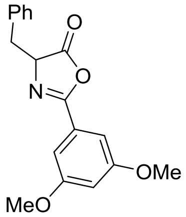

^{1}H NMR (500 MHz, $CDCl_3$) δ 7.31 – 7.18 (m, 5H), 7.05 (s, 2H), 6.63 (s, 1H), 4.69 (t, *J* = 5.7 Hz, 1H), 3.82 (s, 6H), 3.37 (dd, *J* = 13.9, 4.9 Hz, 1H), 3.20 (dd, *J* = 13.9, 6.5 Hz, 1H). ^{13}C NMR (126 MHz, $CDCl_3$) δ 177.6, 161.8, 161.0, 135.3, 129.7, 128.6, 127.5, 127.4, 105.8, 105.6, 66.7, 55.8, 37.4. IR: ν = 3058, 3027, 3006, 2929, 2832, 1694, 1628, 1602, 1596, 1528 cm^{-1}. HRMS-ESI (m/z) calculated for $C_{18}H_{18}NO_4^+$:312.1230; found: 312.1227 $[M+H]^+$.

2-(3,5-dimethoxyphenyl)-4-(2-(methylthio)ethyl)oxazol-5(4H)-one (**2x**)

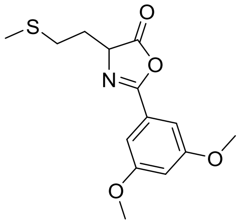

^{1}H NMR (400 MHz, $CDCl_3$) δ 7.13 (s, 2H), 6.65 (d, *J* = 2.0 Hz, 1H), 4.60 (t, *J* = 5.9 Hz, 1H), 3.84 (s, 6H), 2.73 (t, *J* = 6.5 Hz, 2H), 2.30 (dt, *J* = 12.6, 6.5 Hz, 1H), 2.13 (m, 4H). ^{13}C NMR (101 MHz, $CDCl_3$) δ 178.5, 162.1, 161.1, 127.6, 105.9, 105.6, 63.9, 55.8, 30.5, 30.2, 15.3. IR: ν = 2920, 1825, 1706, 1651, 1596, 1527 cm^{-1}. HRMS-ESI (m/z) calculated for $C_{14}H_{18}NO_4S$ $^+$: 296.0951; found: 296.0945 $[M+H]^+$.

tert-butyl (4-(2-(3,5-dimethoxyphenyl)-5-oxo-4,5-dihydrooxazol-4-yl)butyl)carbamate (**2y**)

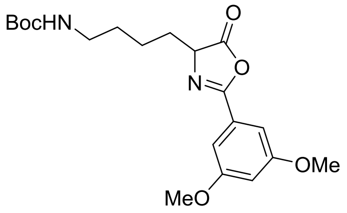

^{1}H NMR (400 MHz, Chloroform-*d*) δ 7.14 (d, *J* = 2.4 Hz, 2H), 6.66 (t, *J* = 2.4 Hz, 1H), 4.53 (s, 1H), 4.40 (dd, *J* = 7.2, 5.5 Hz, 1H), 3.85 (s, 6H), 3.14 (d, *J* = 7.3 Hz, 2H), 2.04 (td, *J* = 8.9, 8.3, 4.2 Hz, 1H), 1.94 – 1.82 (m, 1H), 1.56 – 1.48 (m, 4H), 1.43 (s, 9H). ^{13}C NMR (101 MHz, $CDCl_3$) δ 178.3, 161.7, 161.0, 155.9, 127.5, 105.8, 105.5, 77.2, 65.4, 55.7, 40.2, 31.2, 29.7, 28.4, 22.6.IR: ν =3356, 3006, 2982, 2932, 2864, 2837, 1823, 1694, 1644,1589, 1507. HRMS-ESI (m/z) calculated for $C_{20}H_{29}N_2O_6^+$: 393.2020; found: 393.2016 $[M+H]^+$

Asymmetric synthesis of products:

General procedure for asymmetric synthesis of the chiral products: To a dried 3 ml vial was added **1** (0.15 mmol), **2** (0.1 mmol), CPA catalyst (0.01 mmol) and activated 3 Å molecular sieves (100 mg). The vial was purged with N_2 for 3 times and then followed by adding CCl_4 (0.5 mL). After stirring for 24 h, the reaction mixture was quenched by adding K_2CO_3. After filtration, the filtrate was concentrated under vacuum to give a residue, which was purified by flash column chromatography to give the allene product **3 or 4.**

(*S*)-4-((*1R,3R*)-2-acetyl-1,4-diphenylbuta-2,3-dien-1-yl)-2-(3,5-dimethoxyphenyl)-4-ethyloxazol-5(4H) -one (**3a**)

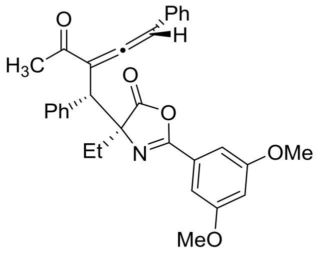

37 mg, 75% yield. ^{1}H NMR (400 MHz, $CDCl_3$) δ 7.29 (m, 7H), 7.17 – 7.07 (m, 3H), 6.95 (d, *J* = 2.2 Hz, 2H), 6.93 (s, 1H), 6.58 (t, *J* = 2.2 Hz, 1H), 4.71 (s, 1H), 3.70 (s, 6H), 2.27 (s, 3H), 2.04 (td, *J* = 12.3, 6.8 Hz, 2H), 0.80 (t, *J* = 7.4 Hz, 3H). ^{13}C NMR (101 MHz, $CDCl_3$) δ 217.0, 196.7, 178.7, 160.9, 160.4, 137.2, 131.7, 130.0, 129.2, 128.4, 128.3, 127.8, 127.5, 127.5, 112.1, 105.8, 105.3, 101.3, 77.9, 55.6, 48.5, 29.4, 27.0, 8.5. $[\alpha]_D^{25}$ = 63.4 (c 1.0, $CHCl_3$). IR: ν = 2971, 2938, 2841, 1928, 1825, 1805, 1680, 1653, 1596 cm^{-1}. HRMS-ESI (m/z) calculated for $C_{31}H_{30}NO_5^+$: 496.2118; found: 496.2108 $[M+H]^+$. HPLC: Chiralpak IA column, 90:10 hexanes/isopropanol, 1 ml/min; t_R = 5.8 min (major), 10.5 min (minor); 91% ee.

(*S*)-4-((*1R*,*3R*)-2-acetyl-1-phenyl-4-(p-tolyl)buta-2,3-dien-1-yl)-2-(3,5-dimethoxyphenyl)-4-ethyloxazol-5(4H)-one (**3b**)

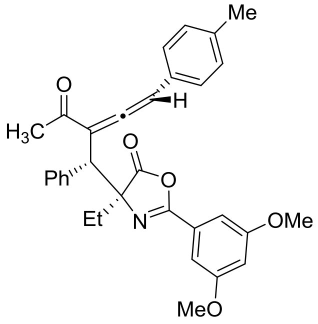

34 mg, 67% yield. ^{1}H NMR (400 MHz, $CDCl_3$) δ 7.38 – 7.29 (m, 2H), 7.20 – 7.05 (m, 7H), 6.95 (d, *J* = 2.3 Hz, 2H), 6.90 (s, 1H), 6.59 (t, *J* = 2.2 Hz, 1H), 4.71 (s, 1H), 4.71 (s, 1H), 3.71 (s, 6H), 2.36 (s, 3H), 2.26 (s, 3H), 2.13 – 1.92 (m, 2H), 0.80 (t, *J* = 7.4 Hz, 3H). ^{13}C NMR (101 MHz, $CDCl_3$) δ 216.8, 196.8, 178.7, 160.9, 160.3, 138.4, 137.3, 130.0, 129.8, 128.6, 128.2, 127.8, 127.6, 127.4, 112.1, 105.7, 105.3, 101.2, 77.9, 55.6, 48.5, 29.4, 26.9, 21.4, 8.4. $[\alpha]_D^{25}$ = 44.80 (c 1.0, $CHCl_3$). IR: ν = 3006, 2989, 1718, 1663, 1595 cm^{-1}. HRMS-ESI (m/z) calculated for $C_{32}H_{32}NO_5^+$: 510.2275; found: 510.2271 $[M+H]^+$. HPLC: Chiralpak IA column, 90:10 hexanes/isopropanol, 1 ml/min; t_R = 5.5 min (major), 9.1 min (minor); 91% ee.

(*S*)-4-((*1R,3R*)-2-acetyl-4-(4-chlorophenyl)-1-phenylbuta-2,3-dien-1-yl)-2-(3,5-dimethoxyphenyl)-4-ethyloxazol-5(4H)-one (**3c**)

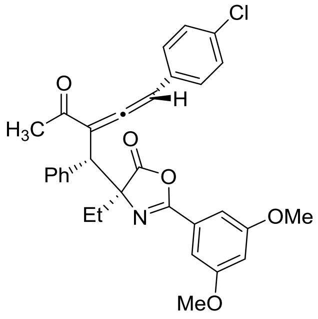

34 mg, 64% yield. ^{1}H NMR (400 MHz, $CDCl_3$) δ 7.30 (m, 4H), 7.20 – 7.07 (m, 5H), 6.94 (d, *J* = 2.3 Hz, 2H), 6.89 (s, 1H), 6.60 (t, *J* = 2.2 Hz, 1H), 4.70 (s, 1H), 3.73 (s, 6H), 2.26 (s, 3H), 2.03 (dd, *J* = 7.7, 3.6 Hz, 2H), 0.80 (t, *J* = 7.4 Hz, 3H). ^{13}C NMR (101 MHz, $CDCl_3$) δ 216.9, 196.3, 178.6, 161.0, 160.4, 137.2, 134.2, 130.2, 129.8, 129.4, 128.6, 128.4, 127.9, 127.5, 112.4, 105.6, 105.4, 100.4, 77.8, 55.6, 48.5, 29.4, 27.0, 8.4. $[\alpha]_D^{25}$ = 44.30 (c 1.0, $CHCl_3$). IR: ν = 3006, 2989, 1805, 1683, 1599 cm^{-1}. HRMS-ESI (m/z) calculated for $C_{31}H_{29}NO_5Cl^+$: 530.1729; found 530.1725 $[M+H]^+$. HPLC: Chiralpak IA column, 90:10 hexanes/isopropanol, 1 ml/min; t_R = 6.0 min (major), 8.9 min (minor); 90% ee.

(*S*)-4-((*1R,3R*)-2-acetyl-4-(4-methoxyphenyl)-1-phenylbuta-2,3-dien-1-yl)-2-(3,5-dimethoxyphenyl)-4-ethyloxazol-5(4H)-one (**3d**)

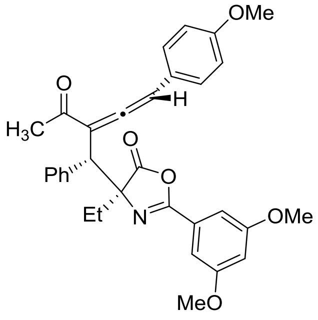

28 mg, 53% yield. ^{1}H NMR (400 MHz, $CDCl_3$) δ 7.38 – 7.29 (m, 2H), 7.21 – 7.08 (m, 5H), 6.96 (d, *J* = 2.3 Hz, 2H), 6.87 (d, *J* = 4.1 Hz, 2H), 6.85 (s, 1H), 6.59 (t, *J* = 2.3 Hz, 1H), 4.71 (s, 1H), 3.82 (s, 3H), 3.72 (s, 6H), 2.25 (s, 3H), 2.10 – 1.94 (m, 2H), 0.79 (t, *J* = 7.4 Hz, 3H). ^{13}C NMR (101 MHz, $CDCl_3$) δ 216.6, 196.9, 178.7, 160.9, 160.3, 159.8, 137.4, 130.0, 128.8, 128.3, 127.8, 127.6, 123.7, 114.6, 112.2, 105.7, 105.3, 100.9, 77.8, 55.6, 55.5, 48.4, 29.4, 26.9, 8.5. $[\alpha]_D^{25}$ = 62.5 (c 1.0, $CHCl_3$). IR: ν = 3006, 2989, 1945, 1805, 1673, 1595 cm^{-1}. HRMS-ESI (m/z) calculated for $C_{32}H_{32}NO_6^+$: 526.2224; found: 526.2218 $[M+H]^+$. HPLC: Chiralpak IA column, 90:10 hexanes/isopropanol, 1 ml/min; t_R = 6.9 min

(major), 14.0 min (minor); 91% ee.

(*S*)-4-((*1R,3R*)-2-acetyl-4-(4-fluorophenyl)-1-phenylbuta-2,3-dien-1-yl)-2-(3,5-dimethoxyphenyl)-4-ethyloxazol-5(4H)-one (**3e**)

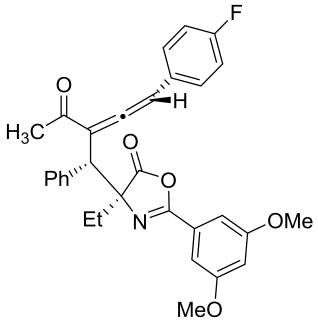

32 mg, 62% yield.^{1}H NMR (500 MHz, $CDCl_3$) δ 7.34 – 7.24 (m, 3H), 7.19 – 7.09 (m, 3H), 7.02 (d, *J* = 7.7 Hz, 1H), 6.98 (td, *J* = 8.5, 2.3 Hz, 1H), 6.95 (d, *J* = 2.0 Hz, 2H), 6.91 (m, 2H), 6.60 (s, 1H), 4.71 (s, 1H), 3.72 (s, 6H), 2.27 (s, 3H), 2.13 – 1.93 (m, 2H), 0.80 (t, *J* = 7.4 Hz, 3H). ^{13}C NMR (126 MHz, $CDCl_3$) δ 217.0, 196.2, 178.6, 163.3 (d, *J* = 246.8 Hz), 161.0, 160.5, 137.1, 134.1 (d, *J* = 7.9 Hz), 130.6 (d, *J* = 8.4 Hz), 129.8, 128.4, 127.9, 127.5, 123.2 (d, *J* = 2.9 Hz), 115.3 (d, *J* = 21.3 Hz), 114.1 (d, *J* = 22.6 Hz), 112.4, 105.7, 105.4, 100.6 (d, *J* = 2.7 Hz), 77.8, 55.6, 48.5, 29.4, 27.0, 8.4. ^{19}F NMR (471 MHz, $CDCl_3$) δ -112.36. $[\alpha]_D^{25}$ = 42.5 (c 1.0, $CHCl_3$). IR: ν = 3058, 2977, 1805, 1683, 1654, 1589 cm^{-1}. HRMS-ESI (m/z) calculated for $C_{31}H_{29}FNO_5^+$: 514.2024; found: 514.2016 $[M+H]^+$. HPLC: Chiralpak IA column, 90:10 hexanes/isopropanol, 1 ml/min; t_R = 5.9 min (major), 9.6 min (minor); 90% ee.

(*S*)-4-((*1R,3R*)-2-acetyl-1-phenyl-4-(4-(trifluoromethyl)phenyl)buta-2,3-dien-1-yl)-2-(3,5-dimethoxyphenyl)-4-ethyloxazol-5(4H)-one (**3f**)

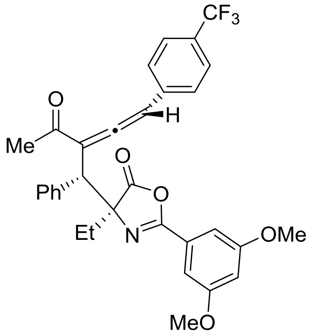

33 mg, 59% yield. ^{1}H NMR (400 MHz, $CDCl_3$) δ 7.59 (d, *J* = 8.1 Hz, 2H), 7.35 (d, *J* = 8.0 Hz, 2H), 7.30 (d, *J* = 6.7 Hz, 2H), 7.20 – 7.06 (m, 3H), 6.96 (s, 1H), 6.92 (d, *J* = 2.3 Hz, 2H), 6.59 (d, *J* = 2.2 Hz,

1H), 4.71 (s, 1H), 3.71 (s, 6H), 2.27 (s, 3H), 2.05 (ddd, *J* = 10.0, 6.8, 2.7 Hz, 2H), 0.81 (t, *J* = 7.4 Hz, 3H). ^{13}C NMR (101 MHz, $CDCl_3$) δ 217.4, 196.0, 178.5, 161.0, 160.5, 137.0, 135.6, 130.2 (q, J = 32.7 Hz), 129.8, 128.4, 128.0, 127.6, 127.4, 126.1 (q, *J* = 3.8 Hz), 124.1 (q, J = 272.1 Hz), 112.5, 105.5, 105.4, 100.4, 77.8, 55.6, 48.6, 29.4, 27.1, 8.4. ^{19}F NMR (376 MHz, $CDCl_3$) δ -62.62. $[\alpha]_D^{25}$ = -31.60 (c 1.0, $CHCl_3$). IR: ν = 2966, 2928, 2032, 1928, 1805, 1683, 1653, 1595 cm^{-1}. HRMS-ESI (m/z) calculated for $C_{32}H_{29}F_3NO_5^+$: 564.1992; found: 564.1991 $[M+H]^+$. HPLC: Chiralpak IA column, 90:10 hexanes/isopropanol, 1 ml/min; t_R = 5.6 min (major), 8.1 min (minor); 88% ee.

Methyl3-((*R*)-3-((*R*)-((*S*)-2-(3,5-dimethoxyphenyl)-4-ethyl-5-oxo-4,5-dihydrooxazol-4-yl)(phenyl)methyl)-4-oxopenta-1,2-dien-1-yl)benzoate (**3g**)

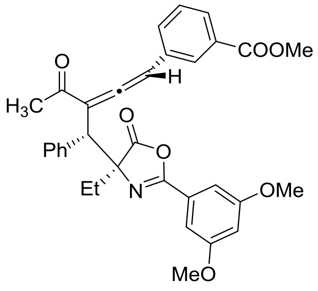

36 mg, 65% yield. ^{1}H NMR (400 MHz, $CDCl_3$) δ 7.97 (d, *J* = 4.6 Hz, 2H), 7.43 – 7.35 (m, 2H), 7.34 – 7.29 (m, 2H), 7.13 (m, 3H), 6.96 (s, 1H), 6.92 (d, *J* = 2.3 Hz, 2H), 6.58 (t, *J* = 2.2 Hz, 1H), 4.71 (s, 1H), 3.88 (s, 3H), 3.69 (s, 6H), 2.27 (s, 3H), 2.15 – 1.96 (m, 2H), 0.80 (t, *J* = 7.4 Hz, 3H). ^{13}C NMR (126 MHz, $CDCl_3$) δ 216.9, 196.3, 178.6, 166.6, 160.9, 160.5, 137.1, 132.3, 131.5, 131.2, 129.9, 129.4, 129.2, 128.5, 128.3, 127.9, 127.5, 112.4, 105.6, 105.4, 100.7, 77.8, 55.6, 52.4, 48.6, 29.4, 27.1, 8.4. $[\alpha]_D^{25}$ = 18.00 (c 1.0, $CHCl_3$). IR: ν = 2965, 2842, 1932, 1825, 1805, 1722, 1680, 1653, 1596cm^{-1}. HRMS-ESI (m/z) calculated for $C_{33}H_{32}NO_7^+$: 554.2173; found: 554.2166 $[M+H]^+$. HPLC: Chiralpak IA column, 90:10 hexanes/isopropanol, 1 ml/min; t_R = 7.8 min (major), 22.9 min (minor); 90% ee.

(*S*)-4-((*1R,3R*)-2-acetyl-4-(2-chlorophenyl)-1-phenylbuta-2,3-dien-1-yl)-2-(3,5-dimethoxyphenyl)-4-ethyloxazol-5(4H)-one **(3h)**

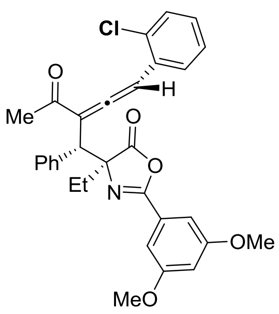

34 mg, 64% yield. ^{1}H NMR (500 MHz, $CDCl_3$) δ 7.42 (d, *J* = 7.3 Hz, 2H), 7.28 (d, *J* = 7.3 Hz, 2H), 7.25 (s, 1H), 7.23 – 7.16 (m, 1H), 7.15 – 7.05 (m, 4H), 6.99 (d, *J* = 1.9 Hz, 2H), 6.59 (s, 1H), 4.71 (s, 1H), 3.75 (s, 6H), 2.30 (d, *J* = 8.9 Hz, 3H), 2.04 (q, *J* = 7.3 Hz, 2H), 0.80 (t, *J* = 7.4 Hz, 3H). ^{13}C NMR (126 MHz, $CDCl_3$) δ 218.0, 196.3, 178.6, 161.0, 160.5, 137.3, 132.6, 130.2, 129.8, 129.6, 129.4, 128.6, 128.4, 127.9, 127.5, 127.3, 112.4, 106.0, 105.3, 97.6, 77.9, 55.7, 48.3, 29.5, 27.1, 8.4. IR: ν = 2965, 2842, 1932, 1825, 1805, 1722, 1680, 1653, 1596 cm^{-1}. $[\alpha]_D^{25}$ = 28.40 (c 1.0, $CHCl_3$). HRMS-ESI (m/z) calculated for $C_{31}H_{29}ClNO_5^+$: 530.1729; found: 530.1733 $[M+H]^+$. HPLC: Chiralpak IC column, 95:05 hexanes/isopropanol, 1 ml/min; t_R = 16.9 min (minor), 25.6 min (major); 90% ee.

(*S*)-4-((*1R*,*3R*)-2-acetyl-1-phenyl-4-(thiophen-3-yl)buta-2,3-dien-1-yl)-2-(3,5-dimethoxyphenyl)-4-ethyloxazol-5(4H)-one (**3i**)

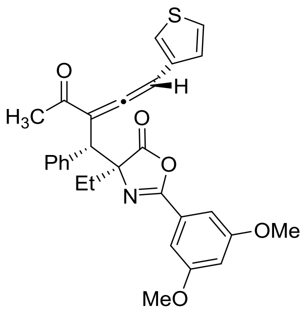

28 mg, 56% yield. ^{1}H NMR (400 MHz, $CDCl_3$) δ 7.26 (dd, *J* = 7.4, 1.7 Hz, 2H), 7.22 (dd, *J* = 4.9, 3.0 Hz, 1H), 7.15 (d, *J* = 1.8 Hz, 1H), 7.07 (t, *J* = 5.8 Hz, 3H), 6.93 (s, 1H), 6.89 (d, *J* = 2.3 Hz, 2H), 6.82 (dd, *J* = 5.0, 0.9 Hz, 1H), 6.53 (t, *J* = 2.3 Hz, 1H), 4.63 (s, 1H), 3.67 (s, 6H), 2.19 (s, 3H), 2.03 – 1.87 (m, 2H), 0.72 (t, *J* = 7.4 Hz, 3H). ^{13}C NMR (101 MHz, $CDCl_3$) δ 217.3, 196.6, 178.7, 160.9, 160.4, 137.3, 132.3, 129.9, 128.3, 127.8, 127.5, 126.9, 126.3, 123.2, 111.4, 105.7, 105.4, 95.9, 77.8, 55.6, 48.3, 29.4, 27.0, 8.4. $[\alpha]_D^{25}$ = 33.50 (c 1.0, $CHCl_3$). IR: ν = 3006, 2989, 2032, 1932, 1802, 1680, 1654, 1599cm^{-1}. HRMS-ESI (m/z) calculated for $C_{29}H_{28}NO_5S^+$: 502.1683; found: 502.1672 $[M+H]^+$. HPLC: Chiralpak IA column, 90:10 hexanes/isopropanol, 1 ml/min; t_R = 6.7 min (major), 12.0 min (minor); 92%

ee.

(*S*)-4-((*1R,3R*)-2-acetyl-4-(cyclohex-1-en-1-yl)-1-phenylbuta-2,3-dien-1-yl)-2-(3,5-dimethoxyphenyl)-4-ethyloxazol-5(4H)-one (**3j**)

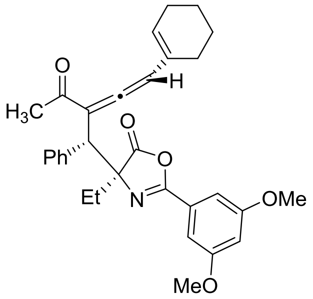

33 mg, 66% yield. ^{1}H NMR (400 MHz, $CDCl_3$) δ 7.33 (dd, *J* = 6.4, 2.9 Hz, 2H), 7.21 – 7.11 (m, 3H), 7.06 (d, *J* = 2.3 Hz, 2H), 6.64 (t, *J* = 2.3 Hz, 1H), 6.52 (s, 1H), 5.86 (s, 1H), 4.66 (s, 1H), 3.83 (s, 6H), 2.22 (s, 3H), 2.16 (d, *J* = 3.0 Hz, 2H), 1.95 (q, *J* = 7.4 Hz, 2H), 1.81 – 1.64 (m, 2H), 1.62 – 1.48 (m, 4H), 0.75 (t, *J* = 7.4 Hz, 3H). ^{13}C NMR (101 MHz, $CDCl_3$) δ 215.6, 197.3, 178.8, 161.0, 160.3, 137.7, 130.5, 129.9, 129.9, 128.1, 127.7, 127.7, 111.5, 105.6, 105.5, 104.9, 77.9, 55.7, 47.7, 29.6, 27.1, 26.2, 26.1, 22.4, 22.2, 8.4. $[\alpha]_D^{25}$ = -18.80 (c 1.0, $CHCl_3$). IR: ν = 3006, 2991, 1977, 1715, 1595 cm^{-1}. HRMS-ESI (m/z) calculated for $C_{31}H_{34}NO_5^{\ +}$: 500.2431; found: 500.2431 $[M+H]^+$. HPLC: Chiralpak IA column, 90:10 hexanes/isopropanol, 1 ml/min; t_R = 5.1 min (major), 6.6 min (minor); 92% ee.

(*S*)-4-((*1R,3R*)-2-acetyl-1-phenylocta-2,3-dien-1-yl)-2-(3,5-dimethoxyphenyl)-4-ethyloxazol-5(4H)-one (**3k**)

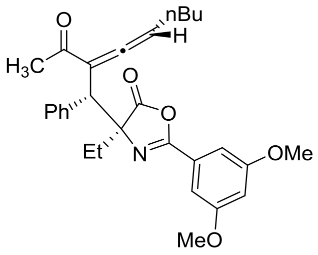

23 mg, 48% yield. ^{1}H NMR (400 MHz, $CDCl_3$) δ 7.33 (dd, *J* = 6.4, 2.9 Hz, 2H), 7.21 – 7.13 (m, 3H), 7.05 (d, *J* = 2.3 Hz, 2H), 6.64 (t, *J* = 2.2 Hz, 1H), 5.84 (t, *J* = 7.2 Hz, 1H), 4.60 (s, 1H), 3.83 (s, 6H), 2.31 – 2.17 (m, 5H), 1.94 (qd, *J* = 13.8, 7.2 Hz, 2H), 1.57 – 1.34 (m, 4H), 0.90 (t, *J* = 7.1 Hz, 3H), 0.75 (t, *J* = 7.4 Hz, 3H). ^{13}C NMR (101 MHz, $CDCl_3$) δ 213.1, 197.5, 178.7, 160.9, 160.0, 137.6, 129.9, 128.0, 127.6, 127.5, 108.7, 105.4, 105.3, 98.5, 77.4, 55.6, 47.3, 31.0, 29.5, 28.2, 26.6, 22.5, 13.8, 8.2.

$[\alpha]_D^{25}$ = 2.80 (c 1.0, $CHCl_3$). IR: ν = 2971, 2013, 1928, 1805, 1679, 1655, 1599 cm^{-1}. HRMS-ESI (m/z) calculated for $C_{29}H_{34}NO_5^+$: 476.2431; found: 476.2426 $[M+H]^+$. HPLC: Chiralpak IA column, 90:10 hexanes/isopropanol, 1 ml/min; t_R = 5.0 min (major), 6.1 min (minor); 92% ee.

(*S*)-4-((*1R*,*3R*)-2-acetyl-4-phenyl-1-(p-tolyl)buta-2,3-dien-1-yl)-2-(3,5-dimethoxyphenyl)-4-ethyloxazol-5(4H)-one (**3l**)

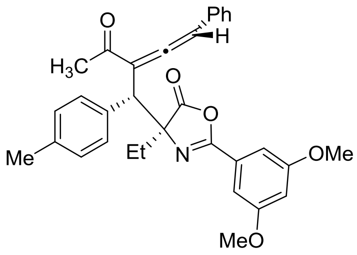

30 mg, 59% yield. ^{1}H NMR (400 MHz, $CDCl_3$) δ 7.40 – 7.24 (m, 5H), 7.21 (d, *J* = 8.0 Hz, 2H), 6.96 (d, *J* = 2.3 Hz, 2H), 6.93 (d, *J* = 9.3 Hz, 3H), 6.59 (t, *J* = 2.3 Hz, 1H), 4.68 (s, 1H), 3.70 (s, 6H), 2.27 (s, 3H), 2.22 (s, 3H), 2.04 (dt, *J* = 13.6, 6.7 Hz, 2H), 0.80 (t, *J* = 7.4 Hz, 3H). ^{13}C NMR (101 MHz, $CDCl_3$) δ 216.8, 196.7, 178.7, 160.9, 160.3, 137.4, 134.1, 131.8, 129.8, 129.1, 129.0, 128.3, 127.6, 127.5, 112.3, 105.7, 105.3, 101.3, 77.9, 55.6, 48.2, 29.4, 27.0, 21.1, 8.4. $[\alpha]_D^{25}$ = 21.10 (c 1.0, $CHCl_3$). IR: ν = 3006, 2989, 2016, 1806, 1680, 1599 cm^{-1}. HRMS-ESI (m/z) calculated for $C_{32}H_{32}NO_5^+$: 510.2275; found: 510.2270 $[M+H]^+$. HPLC: Chiralpak IA column, 90:10 hexanes/isopropanol, 1 ml/min; t_R = 5.8 min (major), 9.2 min (minor); 90% ee.

(*S*)-4-((*1R*,*3R*)-2-acetyl-1-(4-fluorophenyl)-4-phenylbuta-2,3-dien-1-yl)-2-(3,5-dimethoxyphenyl)-4-ethyloxazol-5(4H)-one (**3m**)

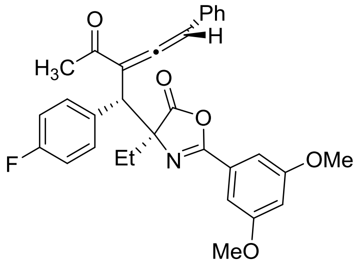

31 mg, 60% yield. ^{1}H NMR (400 MHz, $CDCl_3$) δ 7.39 – 7.27 (m, 5H), 7.25 – 7.19 (m, 2H), 6.95 (d, *J* = 2.3 Hz, 3H), 6.79 (t, *J* = 8.7 Hz, 2H), 6.59 (t, *J* = 2.2 Hz, 1H), 4.71 (s, 1H), 3.71 (s, 6H), 2.28 (s, 3H), 2.03 (td, *J* = 11.5, 6.9 Hz, 2H), 0.80 (t, *J* = 7.4 Hz, 3H). ^{13}C NMR (101 MHz, $CDCl_3$) δ 216.8, 196.6,

178.7, 162.4 (d, J = 246.6 Hz), 161.0, 160.5, 133.1 (d, J = 3.2 Hz), 131.6, 131.5 (d, J = 3.9 Hz), 129.2, 128.5, 127.4, 127.4, 115.2 (d, J = 21.3 Hz), 112.1, 105.8, 105.3, 101.5, 77.9, 55.6, 47.6, 29.3, 27.0, 8.4. ^{19}F NMR (376 MHz, $CDCl_3$) δ -114.62. $[\alpha]_D^{25}$ = -1.10 (c 1.0, $CHCl_3$). IR: ν = 2977, 2032, 1932, 1805, 1680, 1654, 1599 cm^{-1}. HRMS-ESI (m/z) calculated for $C_{31}H_{29}FNO_5^+$: 514.2024; found: 514.2019 $[M+H]^+$. HPLC: Chiralpak IA column, 90:10 hexanes/isopropanol, 1 ml/min; t_R = 6.0 min (major), 10.0 min (minor); 92% ee.

(*S*)-4-((*1R,3R*)-2-acetyl-1-(4-chlorophenyl)-4-phenylbuta-2,3-dien-1-yl)-2-(3,5-dimethoxyphenyl)-4-ethyloxazol-5(4H)-one (**3n**)

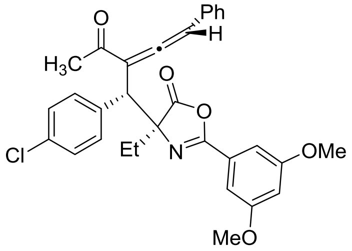

34 mg, 64% yield. ^{1}H NMR (400 MHz, $CDCl_3$) δ 7.32 – 7.21 (m, 3H), 7.21 – 7.13 (m, 4H), 7.01 (d, J = 8.5 Hz, 2H), 6.90 – 6.83 (m, 3H), 6.52 (t, J = 2.3 Hz, 1H), 4.61 (s, 1H), 3.63 (s, 6H), 2.20 (s, 3H), 2.04 – 1.85 (m, 2H), 0.72 (t, J = 7.4 Hz, 3H). ^{13}C NMR (101 MHz, $CDCl_3$) δ 216.7, 196.5, 178.6, 161.0, 160.5, 135.9, 133.8, 131.4, 131.3, 129.3, 128.6, 128.5, 127.4, 127.3, 111.9, 105.9, 105.3, 101.6, 77.7, 55.6, 47.7, 29.4, 26.9, 8.4. $[\alpha]_D^{25}$ = 23.40 (c 1.0, $CHCl_3$). IR: ν = 3006, 2971, 1932, 1825, 1805, 1680, 1653, 1595 cm^{-1}. HRMS-ESI (m/z) calculated for $C_{31}H_{29}ClNO_5^+$: 530.1729; found: 530.1724 $[M+H]^+$. HPLC: Chiralpak IA column, 90:10 hexanes/isopropanol, 1 ml/min; t_R = 6.2 min (major), 10.5 min (minor); 92% ee.

(*S*)-4-((*1R,3R*)-2-acetyl-1-(4-methoxyphenyl)-4-phenylbuta-2,3-dien-1-yl)-2-(3,5-dimethoxyphenyl)-4-ethyloxazol-5(4H)-one (**3o**)

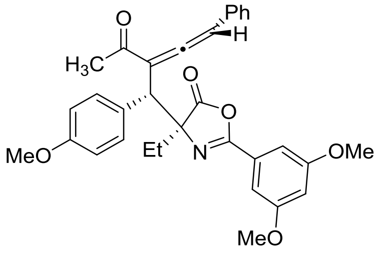

33 mg, 63% yield. ^{1}H NMR (400 MHz, $CDCl_3$) δ 7.40 – 7.18 (m, 7H), 6.95 (d, *J* = 2.3 Hz, 2H), 6.92 (s, 1H), 6.64 (d, *J* = 8.7 Hz, 2H), 6.58 (t, *J* = 2.3 Hz, 1H), 4.67 (s, 1H), 3.70 (s, 6H), 3.69 (s, 3H), 2.27 (s, 3H), 2.10 – 1.98 (m, 2H), 0.80 (t, *J* = 7.4 Hz, 3H). ^{13}C NMR (101 MHz, $CDCl_3$) δ 216.8, 196.7, 178.8, 160.9, 160.3, 159.1, 131.7, 131.0, 129.2, 129.2, 128.3, 127.6, 127.5, 113.6, 112.3, 105.7, 105.3, 101.3, 78.1, 55.6, 55.2, 47.7, 29.3, 27.0, 8.5. $[\alpha]_D^{25}$ = 28.10 (c 1.0, $CHCl_3$). IR: ν = 3006, 2989, 1929 , 1805, 1679, 1599 cm^{-1}. HRMS-ESI (m/z) calculated for $C_{32}H_{32}NO_6^+$: 526.2224; found: 526.2225 $[M+H]^+$. HPLC: Chiralpak IA column, 90:10 hexanes/isopropanol, 1 ml/min; t_R = 7.5 min (major), 11.6 min (minor); 89% ee.

(*S*)-4-((*1R,3R*)-2-acetyl-1-(naphthalen-2-yl)-4-phenylbuta-2,3-dien-1-yl)-2-(3,5-dimethoxyphenyl)-4-ethyloxazol-5(4H)-one (**3p**)

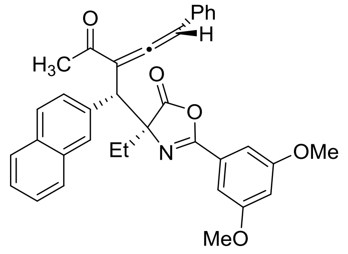

32 mg, 59% yield. ^{1}H NMR (400 MHz, $CDCl_3$) δ 7.81 (s, 1H), 7.70 (d, *J* = 7.4 Hz, 1H), 7.59 (d, *J* = 8.6 Hz, 1H), 7.55 (d, *J* = 7.4 Hz, 1H), 7.47 (dd, *J* = 8.5, 1.5 Hz, 1H), 7.42 – 7.28 (m, 7H), 7.01 (s, 1H), 6.94 (d, *J* = 2.3 Hz, 2H), 6.58 (t, *J* = 2.2 Hz, 1H), 4.91 (s, 1H), 3.68 (s, 6H), 2.29 (s, 3H), 2.11 (qd, *J* = 13.8, 7.2 Hz, 2H), 0.84 (t, *J* = 7.4 Hz, 3H). ^{13}C NMR (101 MHz, $CDCl_3$) δ 216.9, 196.6, 178.7, 160.9, 160.5, 134.7, 133.1, 132.8, 131.7, 129.5, 129.2, 128.4, 128.1, 127.8, 127.6, 127.5, 127.5, 126.1, 126.0, 112.2, 105.9, 105.2, 101.4, 77.9, 55.6, 48.7, 29.4, 27.0, 8.4. $[\alpha]_D^{25}$ = 22.10 (c 1.0, $CHCl_3$). IR: ν = 3006, 2989, 1928, 1805, 1679, 1653, 1598 cm^{-1}. HRMS-ESI (m/z) calculated for $C_{35}H_{32}NO_5^+$: 546.2275; found: 546.2269 $[M+H]^+$. HPLC: Chiralpak IA column, 90:10 hexanes/isopropanol, 1 ml/min; t_R = 7.3 min (major), 12.8 min (minor); 90% ee.

(*S*)-4-((*1R,3R*)-2-acetyl-1-cyclohexyl-4-phenylbuta-2,3-dien-1-yl)-2-(3,5-dimethoxyphenyl)-4-ethyloxazol-5(4H)-one (**3q**)

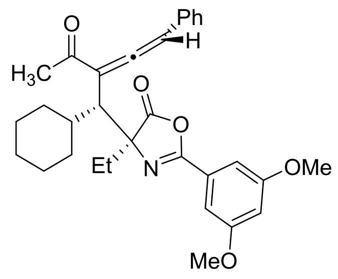

19 mg, 38% yield. ^{1}H NMR (400 MHz, $CDCl_3$) δ 7.34 (dt, *J* = 27.3, 7.7 Hz, 5H), 7.09 (d, *J* = 2.3 Hz, 2H), 6.80 (s, 1H), 6.63 (t, *J* = 2.2 Hz, 1H), 3.81 (s, 6H), 3.42 (d, *J* = 6.4 Hz, 1H), 2.34 (s, 3H), 1.93 (ddd, *J* = 21.0, 13.7, 6.9 Hz, 2H), 1.77 – 1.39 (m, 6H), 1.18 – 0.91 (m, 5H), 0.73 (t, *J* = 7.4 Hz, 3H). ^{13}C NMR (101 MHz, $CDCl_3$) δ 216.6, 197.5, 180.0, 161.0, 159.6, 132.3, 129.2, 128.2, 127.8, 127.5, 111.6, 105.6, 105.5, 100.4, 55.7, 40.7, 32.6, 31.0, 29.6, 26.8, 26.6, 26.3, 26.3, 7.9. $[\alpha]_D^{25}$ = 84.60 (c 1.0, $CHCl_3$). IR: ν = 3006, 2931, 2854, 2022, 1928, 1819, 1676, 1596cm^{-1}. HRMS-ESI (m/z) calculated for $C_{31}H_{36}NO_5^+$: 502.2588; found: 502.2582 [M+H]$^+$. HPLC:Chiralpak IA column, 90:10 hexanes/isopropanol, 1 ml/min; t_R = 5.1 min (major), 7.2 min (minor); 93% ee.

(*S*)-2-(3,5-dimethoxyphenyl)-4-ethyl-4-((*R*)-3-oxo-1-phenyl-2-((*R*)-2-phenylvinylidene)pentyl)oxazol-5(4H)-one (**3r**)

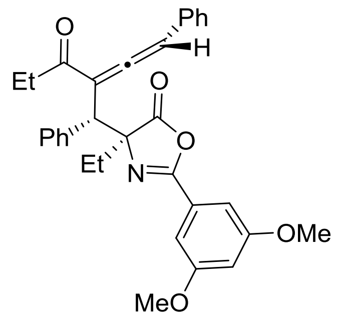

32 mg, 63% yield. ^{1}H NMR (400 MHz, $CDCl_3$) δ 7.40 – 7.21 (m, 7H), 7.20 – 7.06 (m, 3H), 6.94 (t, *J* = 4.8 Hz, 2H), 6.91 (s, 1H), 6.58 (t, *J* = 2.2 Hz, 1H), 4.73 (s, 1H), 3.71 (d, *J* = 14.0 Hz, 6H), 2.78 (dq, *J* = 16.9, 7.3 Hz, 1H), 2.62 – 2.45 (m, 1H), 2.15 – 1.91 (m, 2H), 0.99 (t, *J* = 7.3 Hz, 3H), 0.80 (t, *J* = 7.4 Hz, 3H). ^{13}C NMR (101 MHz, $CDCl_3$) δ 216.0, 199.7, 178.7, 160.9, 160.3, 137.3, 131.8, 130.0, 129.1, 128.3, 128.3, 127.8, 127.5, 127.5, 111.3, 105.8, 105.3, 101.5, 77.9, 55.6, 48.7, 32.6, 29.4, 8.7, 8.5. $[\alpha]_D^{25}$ = 41.70 (c 1.0, $CHCl_3$). IR: ν = 3006, 2989, 1931, 1802, 1683, 1654, 1598 cm^{-1}. HRMS-ESI (m/z) calculated for $C_{32}H_{32}NO_5^+$: 510.2275; found: 510.2264 [M+H]$^+$. HPLC: Chiralpak IA column, 90:10 hexanes/isopropanol, 1 ml/min; t_R = 5.3 min (major), 9.6 min (minor); 98% ee.

(*S*)-2-(3,5-dimethoxyphenyl)-4-ethyl-4-((*R*)-3-oxo-1-phenyl-2-((*R*)-2-phenylvinylidene)hexyl)oxazol-5(4H)-one (**3s**)

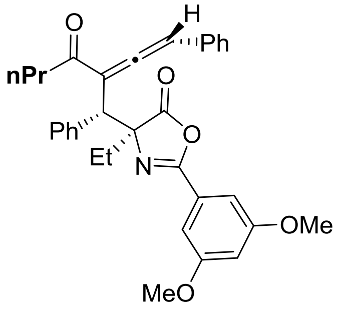

33 mg, 63% yield. ^{1}H NMR (400 MHz, $CDCl_3$) δ 7.38 – 7.20 (m, 7H), 7.18 – 7.06 (m, 3H), 6.95 (d, *J* = 2.2 Hz, 2H), 6.92 (s, 1H), 6.58 (t, *J* = 2.1 Hz, 1H), 4.73 (s, 1H), 3.70 (s, 6H), 2.79 – 2.63 (m, 1H), 2.56 – 2.42 (m, 1H), 2.04 (dt, *J* = 13.7, 6.7 Hz, 2H), 1.55 (td, *J* = 13.8, 6.8 Hz, 2H), 0.79 (m, 6H). ^{13}C NMR (101 MHz, $CDCl_3$) δ 216.1, 199.2, 178.7, 160.9, 160.3, 137.3, 131.8, 130.0, 129.1, 128.3, 128.3, 127.8, 127.5, 127.5, 111.7, 105.8, 105.3, 101.5, 77.8, 55.6, 48.6, 41.1, 29.4, 18.3, 13.8, 8.4. $[\alpha]_D^{25}$ = 75.50 (c 1.0, $CHCl_3$). IR: ν = 3006, 2989, 1931, 1802, 1683, 1654, 1598 cm^{-1}. HRMS-ESI (m/z) calculated for $C_{33}H_{34}NO_5^+$: 524.2431; found: 524.2422 $[M+H]^+$. HPLC: Chiralpak IA column, 90:10 hexanes/isopropanol, 1 ml/min; t_R = 5.1 min (major), 7.7 min (minor); 98% ee.

(*S*)-2-(3,5-dimethoxyphenyl)-4-ethyl-4-((*R*)-3-oxo-1-phenyl-2-((*R*)-2-phenylvinylidene)heptyl)oxazol-5(4H)-one (**3t**)

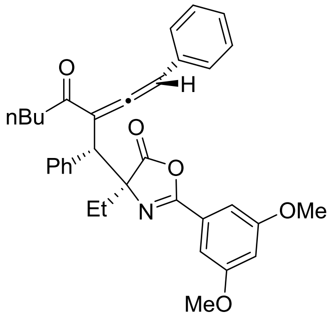

33 mg, 61% yield. ^{1}H NMR (400 MHz, $CDCl_3$) δ 7.39 – 7.27 (m, 5H), 7.26 – 7.20 (m, 2H), 7.13 (m, 3H), 6.96 (d, *J* = 2.3 Hz, 2H), 6.91 (s, 1H), 6.59 (t, *J* = 2.2 Hz, 1H), 4.73 (s, 1H), 3.71 (s, 6H), 2.72 (ddd, *J* = 15.4, 8.3, 6.7 Hz, 1H), 2.58 – 2.44 (m, 1H), 2.13 – 1.95 (m, 2H), 1.48 (dd, *J* = 15.2, 7.0 Hz, 2H), 1.17 (dd, *J* = 14.5, 7.2 Hz, 2H), 0.78 (dt, *J* = 14.8, 7.4 Hz, 6H). ^{13}C NMR (101 MHz, $CDCl_3$) δ 216.1, 199.3, 178.7, 160.9, 160.3, 137.3, 131.8, 130.0, 129.1, 128.3, 128.3, 127.8, 127.5, 127.5, 111.7, 105.8, 105.3, 101.5, 77.8, 55.6, 48.6, 39.0, 29.4, 27.1, 22.4, 13.9, 8.4. $[\alpha]_D^{25}$ = 23.2 (c 1.0, $CHCl_3$). IR:

ν = 3006, 2989, 1802, 1654, 1599 cm^{-1}. HRMS-ESI (m/z) calculated for $C_{34}H_{36}NO_5^+$: 538.2588; found: 538.2585 $[M+H]^+$. HPLC: Chiralpak IA column, 90:10 hexanes/isopropanol, 1 ml/min; t_R = 5.0 min (major), 7.0 min (minor); 99% ee.

(*S*)-2-(3,5-dimethoxyphenyl)-4-ethyl-4-((*R*)-5-methyl-3-oxo-1-phenyl-2-((*R*)-2-phenylvinylidene)hexyl)oxazol-5(4H)-one (**3u**)

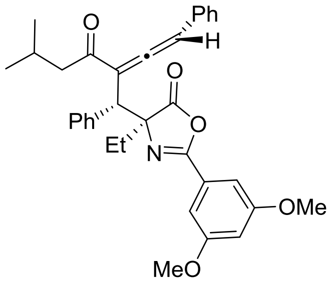

34 mg, 63% yield. ^{1}H NMR (500 MHz, $CDCl_3$) δ 7.38 – 7.20 (m, 7H), 7.13 (p, *J* = 6.6 Hz, 3H), 6.95 (s, 2H), 6.90 (s, 1H), 6.58 (s, 1H), 4.73 (s, 1H), 3.70 (s, 6H), 2.56 (dd, *J* = 15.0, 7.2 Hz, 1H), 2.39 (dd, *J* = 15.0, 6.6 Hz, 1H), 2.12 – 1.95 (m, 3H), 0.80 (t, *J* = 6.2 Hz, 6H), 0.75 (d, *J* = 6.6 Hz, 3H). ^{13}C NMR (126 MHz, $CDCl_3$) δ 216.3, 198.9, 178.7, 160.9, 160.4, 137.4, 131.8, 130.0, 129.1, 128.3, 128.3, 127.8, 127.6, 127.5, 112.3, 105.8, 105.3, 101.6, 77.8, 55.6, 48.6, 47.9, 29.4, 25.9, 22.8, 22.6, 8.4. $[\alpha]_D^{25}$ = 10.70 (c 1.0, $CHCl_3$). IR: ν = 2961, 2035, 1932, 1805, 1673, 1598 cm^{-1}. HRMS-ESI (m/z) calculated for $C_{34}H_{36}NO_5^+$: 538.2588; found: 538.2582 $[M+H]^+$. HPLC: Chiralpak IA column, 90:10 hexanes/isopropanol, 1 ml/min; t_R = 5.0 min (major), 7.0 min (minor); 98% ee.

(*S*)-4-((*1R*,*3R*)-2-acetyl-1,4-diphenylbuta-2,3-dien-1-yl)-4-butyl-2-(3,5-dimethoxyphenyl)oxazol-5(4H)-one (**3v**)

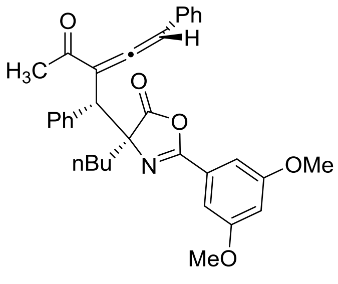

35 mg, 67% yield. ^{1}H NMR (400 MHz, $CDCl_3$) δ 7.38 – 7.21 (m, 9H), 7.18 – 7.06 (m, 3H), 6.94 (d, *J* = 2.1 Hz, 3H), 6.58 (t, *J* = 2.2 Hz, 1H), 4.71 (s, 1H), 3.69 (s, 6H), 2.28 (s, 3H), 2.03 – 1.92 (m, 2H), 1.29 (dq, *J* = 14.1, 6.9 Hz, 2H), 1.23 – 1.11 (m, 1H), 1.11 – 0.98 (m, 1H), 0.84 (t, *J* = 7.3 Hz, 3H). ^{13}C NMR

(101 MHz, $CDCl_3$) δ 217.0, 196.6, 178.9, 160.9, 160.2, 137.2, 131.7, 130.0, 129.1, 128.4, 128.3, 127.8, 127.6, 127.5, 112.1, 1058, 105.3, 101.3, 77.4, 55.6, 48.8, 35.9, 27.0, 26.2, 22.7, 14.0. $[\alpha]_D^{25}$ = 6.5 (c 1.0, $CHCl_3$). IR: ν = 3006, 2991, 2032, 1717, 1653, 1559 cm^{-1}. HRMS-ESI (m/z) calculated for $C_{33}H_{34}NO_5^+$: 524.2431; found: 524.2426 $[M+H]^+$. HPLC: Chiralpak IA column, 90:10 hexanes/isopropanol, 1 ml/min; t_R = 5.4 min (major), 10.7 min (minor); 95% ee.

(*S*)-4-((*1R*,*3R*)-2-acetyl-1,4-diphenylbuta-2,3-dien-1-yl)-4-benzyl-2-(3,5-dimethoxyphenyl)oxazol-5(4H)-one (**3w**)

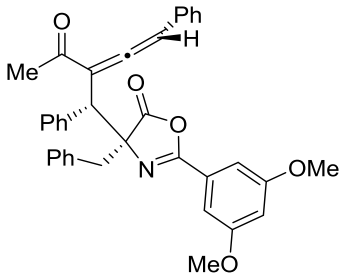

27 mg, 48% yield. ^{1}H NMR (400 MHz, $CDCl_3$) δ 7.46 – 7.23 (m, 9H), 7.21 – 7.11 (m, 6H), 6.98 (s, 1H), 6.76 (d, *J* = 2.2 Hz, 2H), 6.53 (t, *J* = 2.2 Hz, 1H), 4.86 (s, 1H), 3.66 (s, 6H), 3.43 – 3.36 (m, 1H), 3.20 (d, *J* = 13.3 Hz, 1H), 2.30 (s, 3H). ^{13}C NMR (101 MHz, $CDCl_3$) δ 216.9, 196.7, 177.9, 160.7, 160.1, 137.2, 134.3, 1317, 130.4, 130.1, 129.2, 128.4, 128.3, 128.2, 127.9, 127.5, 127.5, 127.3, 112.2, 105.4, 105.3, 101.5, 78.3, 55.5, 48.7, 42.4, 27.1. $[\alpha]_D^{25}$ = -41.50 (c 1.0, $CHCl_3$). IR: ν = 2989, 1812, 1680, 1598 cm^{-1}. HRMS-ESI (m/z) calculated for $C_{36}H_{32}NO_5^+$: 558.2275; found: 558.2269 $[M+H]^+$. HPLC: Chiralpak IC column, 90:10 hexanes/isopropanol, 1 ml/min; t_R = 14.7 min (minor), 23.2 min (major); 95% ee.

(*S*)-4-((*1R*,*3R*)-2-acetyl-1,4-diphenylbuta-2,3-dien-1-yl)-2-(3,5-dimethoxyphenyl)-4-(2-(methylthio)ethyl)oxazol-5(4H)-one (**3x**)

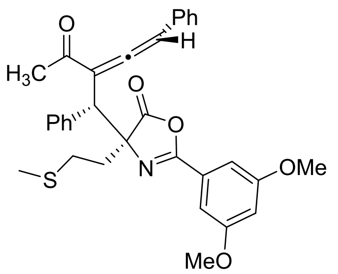

32 mg, 59% yield. ^{1}H NMR (400 MHz, $CDCl_3$) δ 7.39 – 7.22 (m, 7H), 7.18 – 7.05 (m, 3H), 6.95 (s,

1H), 6.91 (d, J = 2.3 Hz, 2H), 6.58 (t, J = 2.2 Hz, 1H), 4.70 (s, 1H), 3.68 (s, 6H), 2.47 – 2.29 (m, 4H), 2.28 (s, 3H), 2.04 (s, 3H). ^{13}C NMR (101 MHz, $CDCl_3$) δ 217.0, 196.6, 178.5, 160.9, 160.8, 136.6, 131.6, 129.9, 129.2, 128.4, 128.3, 128.0, 127.5, 127.4, 111.9, 105.9, 105.3, 101.4, 76.5, 55.6, 48.8, 35.3, 28.8, 27.0, 15.4. $[\alpha]_D^{25}$ = 6.50 (c 1.0, $CHCl_3$). IR: ν = 3006, 2991, 2032, 1815, 1677, 1652, 1599 cm^{-1}. HRMS-ESI (m/z) calculated for $C_{32}H_{32}NO_5S^+$: 542.1996; found: 542.1990 $[M+H]^+$. HPLC: Chiralpak IA column, 90:10 hexanes/isopropanol, 1 ml/min; t_R = 7.5min (major), 19.1 min (minor); 91% ee.

tert-butyl(4-((*S*)-4-((*1R,3R*)-2-acetyl-1,4-diphenylbuta-2,3-dien-1-yl)-2-(3,5-dimethoxyphenyl)-5-oxo-4,5-dihydrooxazol-4-yl)butyl)carbamate (**3y**)

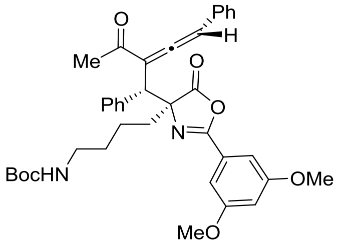

43 mg, 67% yield. ^{1}H NMR (400 MHz, $CDCl_3$) δ 7.29 (m, 7H), 7.12 (dd, J = 10.2, 7.2 Hz, 3H), 6.96 (s, 1H), 6.92 (d, J = 1.9 Hz, 2H), 6.58 (t, J = 2.1 Hz, 1H), 4.69 (s, 1H), 4.49 (s, 1H), 3.69 (s, 6H), 3.03 (d, J = 5.8 Hz, 2H), 2.28 (s, 3H), 2.01 (td, J = 11.2, 4.7 Hz, 2H), 1.55 – 1.34 (m, 11H), 1.27 – 1.04 (m, 2H). ^{13}C NMR (101 MHz, $CDCl_3$) δ 217.0, 196.7, 178.7, 160.9, 160.4, 156.0, 137.0, 131.7, 129.9, 129.1, 128.4, 128.3, 127.9, 127.5, 127.4, 112.0, 105.9, 105.3, 101.4, 79.2, 77.3, 55.6, 48.7, 40.3, 35.7, 30.0, 28.5, 27.0, 21.5. $[\alpha]_D^{25}$ = 47.60 (c 1.0, $CHCl_3$). IR: ν = 3006, 2989, 2032, 1805, 1711, 1654, 1598 cm^{-1}. HRMS-ESI (m/z) calculated for $C_{38}H_{43}N_2O_7^+$: 639.3065; found: 639.3065 $[M+H]^+$. HPLC: Chiralpak IA column, 90:10 hexanes/isopropanol, 1 ml/min; t_R = 8.9 min (major), 20.3 min (minor); 89% ee.

(*S*)-4-((*1R,3S*)-2-acetyl-1,4-diphenylbuta-2,3-dien-1-yl)-2-(3,5-dimethoxyphenyl)-4-ethyloxazol-5(4H) -one (**4a**)

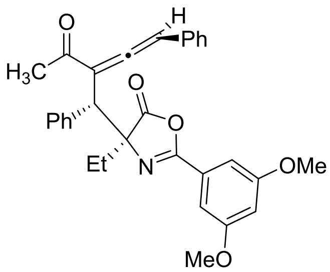

34 mg, 69% yield. ^{1}H NMR (400 MHz, $CDCl_3$) δ 7.54 (d, J = 7.4 Hz, 2H), 7.36 (dd, J = 10.1, 5.4 Hz,

4H), 7.29 (d, J = 7.3 Hz, 1H), 7.21 – 7.14 (m, 3H), 6.81 (s, 1H), 6.78 (d, J = 2.2 Hz, 2H), 6.52 (t, J = 2.0 Hz, 1H), 4.77 (s, 1H), 3.52 (s, 6H), 2.30 (s, 3H), 2.18 – 2.01 (m, 2H), 0.82 (t, J = 7.4 Hz, 3H). ^{13}C NMR (101 MHz, $CDCl_3$) δ 217.6, 196.7, 178.7, 160.8, 160.4, 137.9, 131.9, 129.7, 129.0, 128.3, 128.3, 128.2, 127.9, 127.4, 111.9, 106.0, 105.1, 100.8, 77.8, 55.4, 47.4, 29.9, 26.9, 8.4. $[\alpha]_D^{25}$ = -375.5 (c 1.0, $CHCl_3$). IR: ν = 3006, 2989, 2032, 1743, 1664, 1595 cm^{-1}. HRMS-ESI (m/z) calculated for $C_{31}H_{30}NO_5^+$: 496.2118; found: 496.2109 $[M+H]^+$. HPLC: Chiralpak IA column, 90:10 hexanes/isopropanol, 1 ml/min; t_R = 5.9 min (minor), 7.3min (major); 97% ee.

(*S*)-4-((*1R,3S*)-2-acetyl-1-phenyl-4-(p-tolyl)buta-2,3-dien-1-yl)-2-(3,5-dimethoxyphenyl)-4-ethyloxazol-5(4H)-one (**4b**)

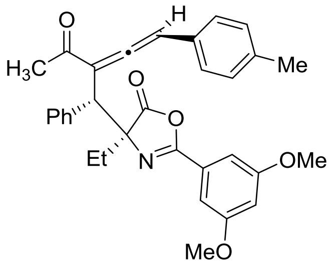

30 mg, 59% yield. ^{1}H NMR (400 MHz, $CDCl_3$) δ 7.39 (d, J = 7.8 Hz, 4H), 7.22 – 7.16 (m, 3H), 7.13 (d, J = 7.9 Hz, 2H), 6.84 – 6.73 (m, 3H), 6.54 (d, J = 2.3 Hz, 1H), 4.77 (s, 1H), 3.55 (s, 6H), 2.34 (s, 3H), 2.29 (s, 3H), 2.07 (t, J = 7.5 Hz, 2H), 0.80 (t, J = 7.4 Hz, 3H). ^{13}C NMR (101 MHz, $CDCl_3$) δ 217.1, 196.8, 178.8, 160.8, 160.3, 138.3, 137.9, 129.8, 129.7, 128.7, 128.3, 128.1, 127.8, 127.5, 111.9, 105.9, 105.2, 100.9, 77.6, 55.4, 47.7, 30.1, 26.9, 21.3, 8.4. $[\alpha]_D^{25}$ = -327.70 (c 1.0, $CHCl_3$). IR: ν = 3006, 2987, 2035, 1928, 1825, 1802, 1679, 1653, 1598 cm^{-1}. HRMS-ESI (m/z) calculated for $C_{32}H_{32}NO_5{}^+$: 510.2275; found: 510.2269 $[M+H]^+$. HPLC:Chiralpak IA column, 90:10 hexanes/isopropanol, 1 ml/min; t_R = 5.5 min (minor), 6.7 min (major); 96% ee.

(*S*)-4-((*1R,3S*)-2-acetyl-4-(4-chlorophenyl)-1-phenylbuta-2,3-dien-1-yl)-2-(3,5-dimethoxyphenyl)-4-ethyloxazol-5(4H)-one (**4c**)

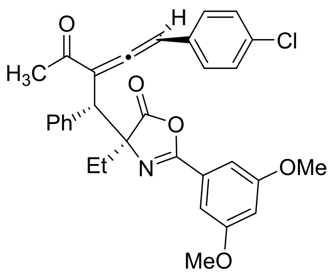

34 mg, 64% yield. ^{1}H NMR (400 MHz, $CDCl_3$) δ 7.44 (d, *J* = 8.4 Hz, 2H), 7.36 (dd, *J* = 6.5, 2.9 Hz, 2H), 7.29 (d, *J* = 8.4 Hz, 2H), 7.19 (dd, *J* = 5.0, 1.6 Hz, 3H), 6.77 (s, 1H), 6.75 (d, *J* = 2.3 Hz, 2H), 6.56 (t, *J* = 2.3 Hz, 1H), 4.75 (s, 1H), 3.61 (s, 6H), 2.29 (s, 3H), 2.11 – 1.95 (m, 2H), 0.80 (t, *J* = 7.4 Hz, 3H). ^{13}C NMR (101 MHz, $CDCl_3$) δ 217.0, 196.3, 178.5, 160.9, 160.5, 137.7, 134.2, 130.3, 129.7, 129.3, 129.2, 128.4, 127.9, 127.3, 112.2, 105.7, 105.3, 100.2, 77.4, 55.5, 47.7, 30.2, 27.0, 8.3. $[\alpha]_D^{25}$ = -432.60 (c 1.0, $CHCl_3$). IR: ν = 3006, 2989, 2032, 1805, 1683, 1653, 1599 cm^{-1}. HRMS-ESI (m/z) calculated for $C_{31}H_{29}NO_5Cl^+$: 530.1729; found: 530.1722 $[M+H]^+$. HPLC: Chiralpak IA column, 90:10 hexanes/isopropanol, 1 ml/min; t_R = 6.4 min (minor), 7.4 min (major); 98% ee.

(*S*)-4-((*1R,3S*)-2-acetyl-4-(4-methoxyphenyl)-1-phenylbuta-2,3-dien-1-yl)-2-(3,5-dimethoxyphenyl)-4-ethyloxazol-5(4H)-one (**4d**)

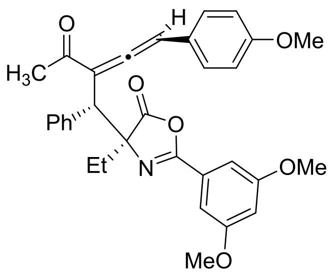

25 mg, 48% yield. ^{1}H NMR (500 MHz, $CDCl_3$) δ 8.27 (d, *J* = 8.8 Hz, 2H), 7.38 (t, *J* = 7.2 Hz, 2H), 7.34 (d, *J* = 7.0 Hz, 1H), 7.28 (d, *J* = 7.1 Hz, 2H), 7.00 (d, *J* = 2.1 Hz, 2H), 6.97 (d, *J* = 8.8 Hz, 2H), 6.63 (t, *J* = 2.1 Hz, 1H), 6.38 (s, 1H), 5.14 (s, 1H), 3.86 (s, 3H), 3.84 (s, 6H), 2.18 (d, *J* = 1.1 Hz, 3H), 1.47 (dd, *J* = 14.8, 7.5 Hz, 1H), 1.14 (dq, *J* = 14.7, 7.4 Hz, 1H), 0.80 (t, *J* = 7.4 Hz, 3H). ^{13}C NMR (126 MHz, $CDCl_3$) δ 193.4, 166.9, 161.1, 160.7, 151.1, 143.6, 136.7, 136.2, 129.4, 128.6, 127.7, 127.5, 127.6, 122.7, 121.5, 114.3, 105.3, 104.0, 74.1, 55.8, 55.5, 47.5, 27.8, 13.2, 7.6. $[\alpha]_D^{25}$ = 78.8 (c 1.0, $CHCl_3$). IR: ν = 3006, 2989, 2841, 2022, 1712, 1664, 1592 cm^{-1}. HRMS-ESI (m/z) calculated for $C_{32}H_{32}NO_6^+$: 526.2224; found: 526.2215 $[M+H]^+$. HPLC:Chiralpak IA column, 90:10 hexanes/isopropanol, 1 ml/min; t_R = 6.8 min (minor), 8.4 min (major); 96% ee.

(S)-4-(($1R$,$3S$)-2-acetyl-4-(4-fluorophenyl)-1-phenylbuta-2,3-dien-1-yl)-2-(3,5-dimethoxyphenyl)-4-ethyloxazol-5(4H)-one (**4e**)

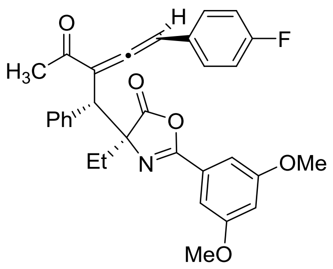

32 mg, 62% yield. ^{1}H NMR (500 MHz, $CDCl_3$) δ 7.32 (m, 5H), 7.18 (m, 3H), 7.01 – 6.93 (m, 1H), 6.83 – 6.73 (m, 3H), 6.54 (s, 1H), 4.76 (s, 1H), 3.58 (s, 6H), 2.30 (s, 3H), 2.17 – 1.97 (m, 2H), 0.82 (t, J = 7.4 Hz, 3H). ^{13}C NMR (126 MHz, $CDCl_3$) δ 217.6, 196.3, 178.6, 163.2 (d, J = 246.4 Hz), 160.9, 160.5, 137.7, 134.3 (d, J = 7.9 Hz), 130.5 (d, J = 8.3 Hz), 129.6, 128.4, 127.9, 127.4, 123.9 (d, J = 2.8 Hz), 115.2 (d, J = 21.4 Hz), 114.8 (d, J = 22.7 Hz), 112.1, 106.0, 105.2, 100.2 (d, J = 2.7 Hz), 77.7, 55.4, 47.5, 30.1, 26.9, 8.3. ^{19}F NMR (471 MHz, $CDCl_3$) δ -112.26. $[\alpha]_D^{25}$ = -309.20 (c 1.0, $CHCl_3$). IR: ν = 3059, 2980, 1932, 1805, 1683, 1653, 1599 cm^{-1}. HRMS-ESI (m/z) calculated for $C_{31}H_{29}FNO_5^+$: 514.2024; found: 514.2014 $[M+H]^+$. HPLC: Chiralpak IA column, 90:10 hexanes/isopropanol, 1 ml/min; t_R = 6.1min (minor), 7.8min (major); 97% ee.

(S)-4-(($1R$,$3S$)-2-acetyl-1-phenyl-4-(4-(trifluoromethyl)phenyl)buta-2,3-dien-1-yl)-2-(3,5-dimethoxyphenyl)-4-ethyloxazol-5(4H)-one (**4f**)

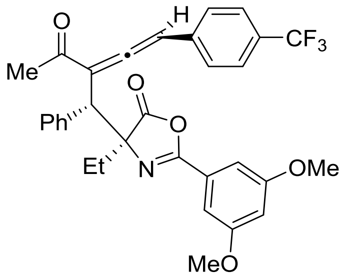

35 mg, 62% yield. ^{1}H NMR (400 MHz, $CDCl_3$) δ 7.65 (dd, J = 21.2, 8.3 Hz, 4H), 7.39 – 7.31 (m, 2H), 7.22 – 7.14 (m, 3H), 6.84 (s, 1H), 6.73 (d, J = 2.2 Hz, 2H), 6.53 (t, J = 2.2 Hz, 1H), 4.77 (s, 1H), 3.55 (s, 6H), 2.31 (s, 3H), 2.17 – 1.95 (m, 2H), 0.82 (t, J = 7.4 Hz, 3H). ^{13}C NMR (101 MHz, $CDCl_3$) δ 217.9, 196.1, 178.4, 160.9, 160.7, 137.6, 135.8, 130.1 (q, J = 32.6 Hz), 129.6, 128.4, 128.3, 128.0, 127.2, 126.0 (q, J = 3.7 Hz), 124.1 (q, J = 272.1 Hz), 112.3, 105.7, 105.3, 100.1, 77.6, 55.3, 47.5, 30.2,

27.0, 8.4. ^{19}F NMR (376 MHz, $CDCl_3$) δ -62.65. $[\alpha]_D^{25}$ = -269.10 (c 1.0, $CHCl_3$). IR: ν = 3006, 2990, 2032, 1928, 1804, 1683, 1650, 1599 cm^{-1}. HRMS-ESI (m/z) calculated for $C_{32}H_{29}F_3NO_5^+$: 564.1992; found: 564.1993 $[M+H]^+$. HPLC: Chiralpak IA column, 90:10 hexanes/isopropanol, 1 ml/min; t_R = 5.9 min (minor), 6.7 min (major); 98% ee.

Methyl 3-((*S*)-3-((*R*)-((*S*)-2-(3,5-dimethoxyphenyl)-4-ethyl-5-oxo-4,5-dihydrooxazol-4-yl) (phenyl)methyl)-4-oxopenta-1,2-dien-1-yl)benzoate (**4g**)

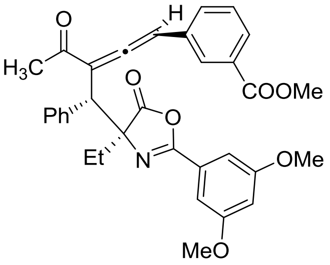

40 mg, 72% yield. ^{1}H NMR (400 MHz, $CDCl_3$) δ 8.22 (s, 1H), 7.94 (d, *J* = 7.8 Hz, 1H), 7.70 (d, *J* = 7.7 Hz, 1H), 7.43 (t, *J* = 7.7 Hz, 1H), 7.37 (dd, *J* = 6.4, 2.8 Hz, 2H), 7.24 – 7.13 (m, 3H), 6.87 (s, 1H), 6.70 (d, *J* = 2.3 Hz, 2H), 6.49 (t, *J* = 2.2 Hz, 1H), 4.77 (s, 1H), 3.90 (s, 3H), 3.53 (s, 6H), 2.30 (s, 3H), 2.08 (dt, *J* = 15.4, 7.7 Hz, 2H), 0.82 (t, *J* = 7.4 Hz, 3H). ^{13}C NMR (126 MHz, $CDCl_3$) δ 217.3, 196.3, 178.6, 166.6, 160.8, 160.4, 137.7, 132.4, 132.3, 131.1, 129.7, 129.4, 129.1, 129.1, 128.4, 127.9, 127.3, 112.2, 105.7, 105.2, 100.3, 77.6, 55.4, 52.4, 47.6, 30.2, 27.0, 8.3. $[\alpha]_D^{25}$ = -314.40 (c 1.0, $CHCl_3$). IR: ν = 3006, 2989, 1931, 1805, 1721, 1680, 1653, 1599 cm^{-1}. HRMS-ESI (m/z) calculated for $C_{33}H_{32}NO_7^+$: 554.2173; found: 554.2169 $[M+H]^+$. HRMS: m/z (ESI) calculated for ($C_{33}H_{31}NO_7$) $[M+H]^+$: 554.2173, found: 554.2169. HPLC: Chiralpak IA column, 90:10 hexanes/isopropanol, 1 ml/min; t_R = 8.2 min (minor), 11.0 min (major); 97% ee.

(*S*)-4-((*1R,3S*)-2-acetyl-4-(2-chlorophenyl)-1-phenylbuta-2,3-dien-1-yl)-2-(3,5-dimethoxyphenyl)-4-ethyloxazol-5(4H)-one (**4h**)

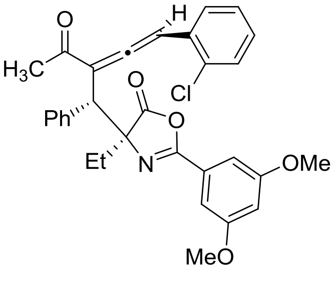

28 mg, 53% yield. ^{1}H NMR (400 MHz, $CDCl_3$) δ 7.83 (dd, *J* = 7.4, 1.9 Hz, 1H), 7.42 (dd, *J* = 7.7, 1.8 Hz, 1H), 7.36 (dd, *J* = 6.5, 2.9 Hz, 2H), 7.31 (s, 1H), 7.25 – 7.17 (m, 4H), 7.11 (m, 1H), 6.79 (d, *J* = 2.3 Hz, 2H), 6.54 (t, *J* = 2.3 Hz, 1H), 4.76 (s, 1H), 3.59 (s, 6H), 2.32 (s, 3H), 2.06 (m, 2H), 0.81 (t, *J* = 7.4 Hz, 3H). ^{13}C NMR (101 MHz, $CDCl_3$) δ 218.1, 196.3, 178.6, 160.9, 160.5, 137.6, 132.8, 130.0, 130.0, 129.8, 129.7, 129.3, 128.4, 128.0, 127.4, 127.3, 112.0, 106.0, 105.2, 97.2, 55.5, 47.6, 30.0, 27.0, 8.4. $[\alpha]_D^{25}$ = -172.80 (c 1.0, $CHCl_3$). IR: ν = 3006, 2989, 2025, 1805, 1680, 1599 cm^{-1}. HRMS: m/z (ESI) calculated for $C_{31}H_{29}ClNO_5^+$: 530.1729, found: 530.1722 $[M+H]^+$. HPLC: Chiralpak IC column, 95:05 hexanes/isopropanol, 1 ml/min; t_R = 16.0 min (minor), 28.8 min (major); 96% ee.

(*S*)-4-((*1R,3R*)-2-acetyl-1-phenyl-4-(thiophen-3-yl)buta-2,3-dien-1-yl)-2-(3,5-dimethoxyphenyl)-4-ethyloxazol-5(4H)-one (**4i**)

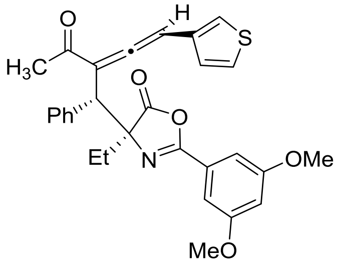

34 mg, 68% yield. ^{1}H NMR (400 MHz, $CDCl_3$) δ 7.41 (s, 1H), 7.39 – 7.30 (m, 4H), 7.17 (m, 3H), 6.89 (s, 1H), 6.84 (d, *J* = 2.3 Hz, 2H), 6.55 (t, *J* = 2.2 Hz, 1H), 4.76 (s, 1H), 3.61 (s, 6H), 2.30 (s, 3H), 2.18 – 1.92 (m, 2H), 0.82 (t, *J* = 7.4 Hz, 3H). ^{13}C NMR (101 MHz, $CDCl_3$) δ 218.2, 196.6, 178.7, 160.9, 160.5, 137.9, 132.6, 129.7, 128.3, 127.9, 127.5, 127.2, 126.6, 123.6, 111.1, 106.0, 105.2, 95.4, 77.9, 55.5, 47.3, 29.8, 26.9, 8.5. $[\alpha]_D^{25}$ = -264.60 (c 1.0, $CHCl_3$). IR: ν = 3006, 2989, 2032, 1932, 1805, 1679, 1653, 1598 cm^{-1}. HRMS: m/z (ESI) calculated for $C_{29}H_{28}NO_5S^+$: 502.1683, found: 502.1675 $[M+H]^+$. HPLC: Chiralpak IA column, 90:10 hexanes/isopropanol, 1 ml/min; t_R = 6.9 min (minor), 8.0 min (major); 99% ee.

(*S*)-4-((*1R,3S*)-2-acetyl-4-(cyclohex-1-en-1-yl)-1-phenylbuta-2,3-dien-1-yl)-2-(3,5-dimethoxyphenyl)-4-ethyloxazol-5(4H)-one (**4j**)

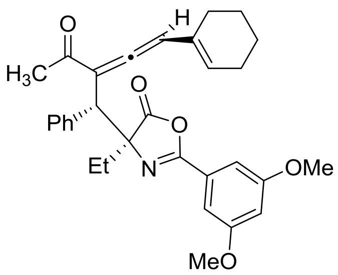

27 mg, 54% yield. ^{1}H NMR (400 MHz, $CDCl_3$) δ 7.31 (dd, *J* = 6.6, 2.9 Hz, 2H), 7.14 (m, 3H), 7.02 (d, *J* = 2.3 Hz, 2H), 6.62 (t, *J* = 2.3 Hz, 1H), 6.46 (s, 1H), 5.94 (s, 1H), 4.69 (s, 1H), 3.80 (s, 6H), 2.65 (d, *J* = 16.1 Hz, 1H), 2.28 (s, 3H), 2.25 – 1.92 (m, 5H), 1.77 – 1.57 (m, 4H), 0.76 (t, *J* = 7.4 Hz, 3H). ^{13}C NMR (101 MHz, $CDCl_3$) δ 216.9, 197.4, 178.9, 161.0, 160.1, 138.3, 131.1, 129.7, 128.2, 127.7, 127.7, 111.4, 105.6, 105.4, 104.1, 78.2, 55.7, 47.0, 29.6, 26.9, 26.5, 26.2, 22.6, 22.3, 8.4. $[\alpha]_D^{25}$ = -184.10 (c 1.0, $CHCl_3$). IR: ν = 3006, 2989, 2048, 1802, 1673, 1595 cm^{-1}. HRMS: m/z (ESI) calculated for $C_{31}H_{34}NO_5^+$: 500.2431, found: 500.2422 $[M+H]^+$. HPLC: Chiralpak IA column, 90:10 hexanes/isopropanol, 1 ml/min; t_R = 5.4 min (minor), 5.9 min (major); 97% ee.

(*S*)-4-((*1R,3S*)-2-acetyl-1-phenylocta-2,3-dien-1-yl)-2-(3,5-dimethoxyphenyl)-4-ethyloxazol-5(4H)-one (**4k**)

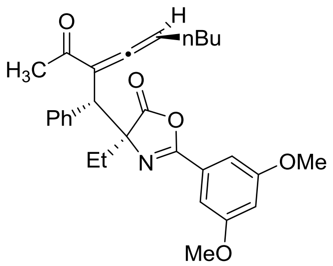

32 mg, 67% yield. ^{1}H NMR (400 MHz, $CDCl_3$) δ 7.34 (dd, *J* = 6.4, 2.9 Hz, 2H), 7.21 – 7.11 (m, 3H), 7.05 (d, *J* = 2.3 Hz, 2H), 6.63 (t, *J* = 2.2 Hz, 1H), 5.81 (t, *J* = 7.1 Hz, 1H), 4.64 (s, 1H), 3.82 (s, 6H), 2.34 (dd, *J* = 15.2, 7.0 Hz, 2H), 2.24 (s, 3H), 1.95 (dt, *J* = 13.5, 6.7 Hz, 2H), 1.61 (dd, *J* = 15.4, 7.5 Hz, 2H), 1.41 (dd, *J* = 14.9, 7.4 Hz, 2H), 0.93 (t, *J* = 7.3 Hz, 3H), 0.75 (t, *J* = 7.4 Hz, 3H). ^{13}C NMR (101 MHz, $CDCl_3$) δ 213.9, 197.7, 179.0, 161.0, 160.0, 138.1, 129.9, 128.1, 127.8, 127.6, 108.7, 105.6, 105.3, 98.3, 77.7, 55.7, 47.0, 31.3, 29.7, 28.7, 26.6, 22.7, 14.0, 8.3. $[\alpha]_D^{25}$ = -127.30 (c 1.0, $CHCl_3$). IR: ν = 2961, 1945, 1802, 1673, 1595 cm^{-1}. HRMS: m/z (ESI) calculated for $C_{29}H_{34}NO_5^+$: 476.2431, found: 476.2433 $[M+H]^+$. HPLC: Chiralpak IB column, 98:02 hexanes/isopropanol, 0.5 ml/min; t_R = 12.6 min (major), 13.3 min (minor); 95% ee.

(*S*)-4-((*1R*,*3S*)-2-acetyl-4-phenyl-1-(p-tolyl)buta-2,3-dien-1-yl)-2-(3,5-dimethoxyphenyl)-4-ethyloxazol-5(4H)-one (**4l**)

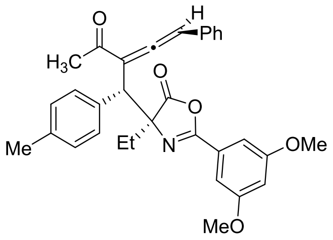

33 mg, 65% yield. ^{1}H NMR (400 MHz, $CDCl_3$) δ 7.54 (d, *J* = 7.3 Hz, 2H), 7.36 (t, *J* = 7.5 Hz, 2H), 7.27 (m, 3H), 6.99 (d, *J* = 7.9 Hz, 2H), 6.84 – 6.75 (m, 3H), 6.53 (t, *J* = 2.3 Hz, 1H), 4.74 (s, 1H), 3.53 (s, 6H), 2.30 (s, 3H), 2.25 (s, 3H), 2.17 – 2.01 (m, 2H), 0.82 (t, *J* = 7.4 Hz, 3H). ^{13}C NMR (101 MHz, $CDCl_3$) δ 217.6, 196.7, 178.8, 160.8, 160.3, 137.5, 134.9, 132.0, 129.5, 129.1, 129.0, 128.2, 128.2, 127.5, 112.1, 106.0, 105.2, 100.8, 77.9, 55.4, 47.1, 30.0, 26.9, 21.2, 8.4. $[\alpha]_D^{25}$ = -337.70 (c 1.0, $CHCl_3$). IR: ν = 3006, 2989, 2840, 1928, 1825, 1802, 1679, 1653, 1598 cm^{-1}. HRMS: m/z (ESI) calculated for $C_{32}H_{32}NO_5^+$: 510.2275, found: 510.2270 $[M+H]^+$. HPLC: Chiralpak IA column, 90:10 hexanes/isopropanol, 1 ml/min; t_R = 6.1 min (minor), 7.8 min (major); 96% ee.

(*S*)-4-((*1R*,*3S*)-2-acetyl-1-(4-fluorophenyl)-4-phenylbuta-2,3-dien-1-yl)-2-(3,5-dimethoxyphenyl)-4-ethyloxazol-5(4H)-one (**4m**)

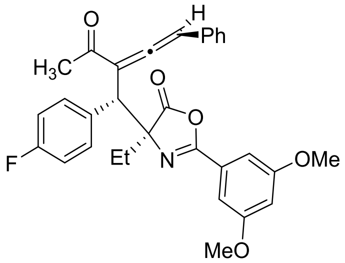

40 mg, 78% yield. ^{1}H NMR (400 MHz, $CDCl_3$) δ 7.53 (d, *J* = 7.3 Hz, 2H), 7.42 – 7.31 (m, 4H), 7.29 (d, *J* = 7.3 Hz, 1H), 6.86 (t, *J* = 8.7 Hz, 2H), 6.82 (s, 1H), 6.78 (d, *J* = 2.3 Hz, 2H), 6.53 (t, *J* = 2.2 Hz, 1H), 4.76 (s, 1H), 3.53 (s, 6H), 2.31 (s, 3H), 2.12 – 1.98 (m, 2H), 0.82 (t, *J* = 7.4 Hz, 3H). ^{13}C NMR (101 MHz, $CDCl_3$) δ 217.4, 196.7, 178.7, 162.40 (d, *J* = 246.6 Hz), 160.9, 160.5, 133.7 (d, *J* = 3.2 Hz), 131.7, 131.3(d, *J* = 8.0 Hz), 129.1, 128.4, 128.2, 127.2, 115.2 (d, *J* = 21.2 Hz), 111.8, 106.1, 105.2, 101.1, 77.8, 55.4, 46.6, 29.9, 26.9, 8.4. ^{19}F NMR (376 MHz, $CDCl_3$) δ -114.54. $[\alpha]_D^{25}$ = -265.80 (c 1.0, $CHCl_3$). IR: ν = 3006, 2989, 2032, 1805, 1679, 1653, 1599 cm^{-1}. HRMS: m/z (ESI) calculated for

$C_{31}H_{29}FNO_5^+$: 514.2024, found: 514.2020 $[M+H]^+$. HPLC: Chiralpak IA column, 90:10 hexanes/isopropanol, 1 ml/min; t_R = 6.1 min (minor), 7.4 min (major); 94% ee.

(*S*)-4-((*1R,3S*)-2-acetyl-1-(4-chlorophenyl)-4-phenylbuta-2,3-dien-1-yl)-2-(3,5-dimethoxyphenyl)-4-ethyloxazol-5(4H)-one (**4n**)

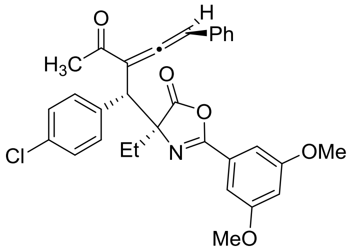

40 mg, 76% yield. ^{1}H NMR (400 MHz, $CDCl_3$) δ 7.53 (d, *J* = 7.3 Hz, 2H), 7.33 (dt, *J* = 15.9, 7.3 Hz, 5H), 7.15 (d, *J* = 8.5 Hz, 2H), 6.82 (s, 1H), 6.78 (d, *J* = 2.3 Hz, 2H), 6.54 (t, *J* = 2.2 Hz, 1H), 4.75 (s, 1H), 3.54 (s, 6H), 2.30 (s, 3H), 2.06 (dt, *J* = 11.0, 6.6 Hz, 2H), 0.81 (t, *J* = 7.4 Hz, 3H). ^{13}C NMR (101 MHz, $CDCl_3$) δ 217.3, 196.6, 178.6, 160.9, 160.6, 136.4, 133.9, 131.6, 131.0, 129.1, 128.5, 128.4, 128.2, 127.2, 111.6, 106.1, 105.2, 101.2, 77.6, 55.4, 46.8, 30.0, 26.9, 8.3. $[\alpha]_D^{25}$ = -300.40 (c 1.0, $CHCl_3$). IR: ν = 3006, 2989, 2026, 1928, 1805, 1679, 1653, 1598 cm^{-1}. HRMS: m/z (ESI) calculated for $C_{31}H_{29}ClNO_5^+$: 530.1729, found: 530.1725 $[M+H]^+$. HPLC: Chiralpak IA column, 90:10 hexanes/isopropanol, 1 ml/min; t_R = 6.5 min (minor), 7.5 min (major); 97% ee.

(*S*)-4-((*1R,3S*)-2-acetyl-1-(4-methoxyphenyl)-4-phenylbuta-2,3-dien-1-yl)-2-(3,5-dimethoxyphenyl)-4-ethyloxazol-5(4H)-one (**4o**)

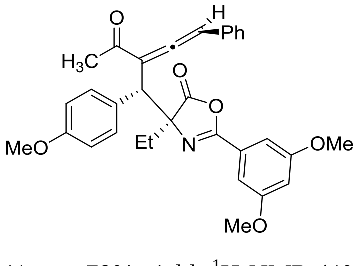

41 mg, 78% yield. ^{1}H NMR (400 MHz, $CDCl_3$) δ 7.54 (d, *J* = 7.3 Hz, 2H), 7.36 (t, *J* = 7.5 Hz, 2H), 7.32 – 7.27 (m, 3H), 6.83 – 6.76 (m, 3H), 6.71 (d, *J* = 8.7 Hz, 2H), 6.52 (t, *J* = 2.2 Hz, 1H), 4.73 (s, 1H), 3.72 (s, 3H), 3.52 (s, 6H), 2.30 (s, 3H), 2.15 – 2.00 (m, 2H), 0.82 (t, *J* = 7.4 Hz, 3H). ^{13}C NMR (101 MHz, $CDCl_3$) δ 217.6, 196.8, 178.9, 160.8, 160.3, 159.1, 132.0, 130.7, 130.0, 129.0, 128.3, 128.2, 127.5, 113.7, 112.2, 106.0, 105.2, 100.8, 78.1, 55.4, 55.2, 46.7, 29.9, 26.9, 8.5. $[\alpha]_D^{25}$ = -273.40 (c 1.0,

$CHCl_3$). IR: ν = 3006, 2840, 2032, 1928, 1805, 679, 1653, 1599 cm^{-1}. HRMS: m/z (ESI) calculated for $C_{32}H_{32}NO_6^+$: 526.2224, found: 526.2219 $[M+H]^+$. HPLC: Chiralpak IA column, 90:10 hexanes/isopropanol, 1 ml/min; t_R = 7.7 min (minor), 10.5 min (major); 95% ee.

(*S*)-4-((*1R,3S*)-2-acetyl-1-(naphthalen-2-yl)-4-phenylbuta-2,3-dien-1-yl)-2-(3,5-dimethoxyphenyl)-4-ethyloxazol-5(4H)-one (**4p**)

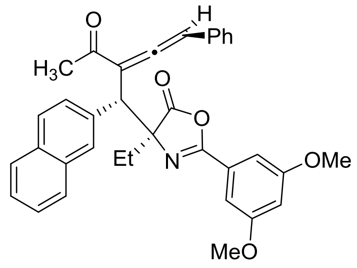

32 mg, 59% yield. ^{1}H NMR (400 MHz, $CDCl_3$) δ 7.85 (s, 1H), 7.77 – 7.67 (m, 2H), 7.64 (d, *J* = 8.6 Hz, 1H), 7.55 (m, 3H), 7.39 (m, 4H), 7.30 (d, *J* = 7.3 Hz, 1H), 6.85 (s, 1H), 6.79 (d, *J* = 2.2 Hz, 2H), 6.54 (t, *J* = 2.2 Hz, 1H), 4.96 (s, 1H), 3.54 (s, 6H), 2.31 (s, 3H), 2.14 (dd, *J* = 14.7, 7.1 Hz, 2H), 0.84 (t, *J* = 7.4 Hz, 3H). ^{13}C NMR (101 MHz, $CDCl_3$) δ 217.4, 196.7, 178.7, 160.8, 160.6, 135.4, 133.2, 132.8, 131.9, 129.3, 129.1, 128.4, 128.2, 128.2, 127.9, 127.5, 127.4, 127.0, 126.1, 126.1, 111.9, 106.2, 105.1, 101.1, 77.8, 55.4, 47.7, 30.1, 26.9, 8.4. $[\alpha]_D^{25}$ = -347.00 (c 1.0, $CHCl_3$). IR: ν = 3006, 2989, 1813, 1680, 1652, 1599 cm^{-1}. HRMS: m/z (ESI) calculated for $C_{35}H_{32}NO_5^+$: 546.2275, found: 546.2269 $[M+H]^+$. HPLC: Chiralpak IA column, 90:10 hexanes/isopropanol, 1 ml/min; t_R = 8.2 min (minor), 10.2 min (major); 97% ee.

(*S*)-4-((*1R,3S*)-2-acetyl-1-cyclohexyl-4-phenylbuta-2,3-dien-1-yl)-2-(3,5-dimethoxyphenyl)-4-ethyloxazol-5(4H)-one (**4q**)

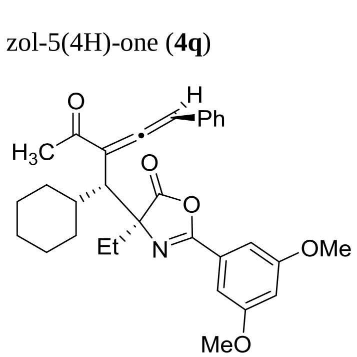

25 mg, 50% yield. ^{1}H NMR (400 MHz, $CDCl_3$) δ 7.50 (d, *J* = 7.5 Hz, 2H), 7.36 (t, *J* = 7.5 Hz, 2H), 7.27 (d, *J* = 9.0 Hz, 1H), 6.88 (d, *J* = 2.2 Hz, 2H), 6.72 (s, 1H), 6.54 (t, *J* = 2.1 Hz, 1H), 3.54 (s, 6H),

3.42 (d, J = 6.4 Hz, 1H), 2.36 (s, 3H), 1.98 (q, J = 7.3 Hz, 2H), 1.75 – 1.53 (m, 7H), 1.06 (m, 4H), 0.74 (t, J = 7.4 Hz, 3H). ^{13}C NMR (101 MHz, $CDCl_3$) δ 217.6, 197.7, 180.4, 160.9, 159.6, 132.5, 129.0, 128.1, 128.0, 127.7, 111.3, 105.9, 105.3, 99.8, 76.6, 55.5, 40.9, 32.6, 30.7, 30.1, 26.8, 26.5, 26.3, 7.8. $[\alpha]_D^{25}$ = -225.00 (c 1.0, $CHCl_3$). IR: ν = 3006, 2991, 1718, 1641, 1561 cm^{-1}. HRMS: m/z (ESI) calculated for $C_{31}H_{36}NO_5^+$: 502.2588, found: 502.2587 $[M+H]^+$. HPLC: Chiralpak IA column, 90:10 hexanes/isopropanol, 1 ml/min; t_R = 5.2 min (minor), 6.5 min (major); 99% ee.

(*S*)-4-((*1R,3S*)-2-acetyl-1,4-diphenylbuta-2,3-dien-1-yl)-4-butyl-2-(3,5-dimethoxyphenyl)oxazol-5(4H)-one (**4v**)

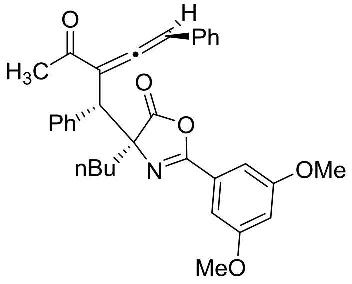

35 mg, 67% yield. ^{1}H NMR (400 MHz, $CDCl_3$) δ 7.55 (d, J = 7.3 Hz, 2H), 7.36 (t, J = 7.1 Hz, 4H), 7.30 (d, J = 7.3 Hz, 1H), 7.21 – 7.14 (m, 3H), 6.82 (s, 1H), 6.78 (d, J = 2.3 Hz, 2H), 6.53 (t, J = 2.2 Hz, 1H), 4.76 (s, 1H), 3.53 (s, 6H), 2.31 (s, 3H), 2.11 – 1.97 (m, 2H), 1.38 – 1.22 (m, 2H), 1.23 – 1.02 (m, 2H), 0.85 (t, J = 7.3 Hz, 3H). ^{13}C NMR (101 MHz, $CDCl_3$) δ 217.7, 196.7, 179.0, 160.8, 160.2, 137.8, 132.0, 129.7, 129.0, 128.3, 128.3, 128.2, 127.9, 127.5, 111.9, 106.0, 105.2, 100.8, 77.4, 55.4, 47.7, 36.2, 26.9, 26.2, 22.6, 14.0. $[\alpha]_D^{25}$ = -259.20 (c 1.0, $CHCl_3$). IR: ν = 3006, 2989, 1825, 1680, 1658, 1599 cm^{-1}. HRMS: m/z (ESI) calculated for $C_{33}H_{34}NO_5^+$: 524.2431, found: 524.2423 $[M+H]^+$. HPLC: Chiralpak IA column, 90:10 hexanes/isopropanol, 1 ml/min; t_R = 5.6 min (minor), 7.1 min (major); 99% ee.

(*S*)-4-((*1R,3S*)-2-acetyl-1,4-diphenylbuta-2,3-dien-1-yl)-4-benzyl-2-(3,5-dimethoxyphenyl)oxazol-5(4H)-one (**4w**)

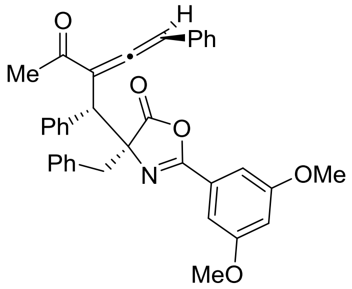

43 mg, 77% yield. ^{1}H NMR (400 MHz, $CDCl_3$) δ 7.69 (d, *J* = 7.3 Hz, 2H), 7.40 (m, 5H), 7.22 – 7.16 (m, 3H), 7.16 – 7.04 (m, 5H), 6.88 (s, 1H), 6.59 (d, *J* = 2.3 Hz, 2H), 6.47 (t, *J* = 2.3 Hz, 1H), 4.96 (s, 1H), 3.50 (s, 6H), 3.37 (dd, *J* = 32.1, 13.3 Hz, 2H), 2.37 (s, 3H). ^{13}C NMR (101 MHz, $CDCl_3$) δ 218.0, 196.8, 177.8, 160.6, 160.3, 137.8, 134.2, 131.9, 130.3, 129.7, 129.1, 128.4, 128.4, 128.2, 128.2, 127.9, 127.3, 127.2, 111.9, 105.8, 105.0, 101.0, 78.5, 55.4, 47.7, 42.7, 26.9. $[\alpha]_D^{25}$ = 349.00 (c 1.0, $CHCl_3$). IR: ν = 3006, 2963, 2843, 1928, 1812, 1679, 1653, 1596 cm^{-1}. HRMS: m/z (ESI) calculated for $C_{36}H_{32}NO_5^+$: 558.2275, found: 558.2267 $[M+H]^+$. HPLC: Chiralpak IC column, 90:10 hexanes/isopropanol, 1 ml/min; t_R = 13.7 min (minor), 17.7 min (major); >99% ee.

(*S*)-4-((*1R,3S*)-2-acetyl-1,4-diphenylbuta-2,3-dien-1-yl)-2-(3,5-dimethoxyphenyl)-4-(2-(methylthio)ethyl)oxazol-5(4H)-one (**4x**)

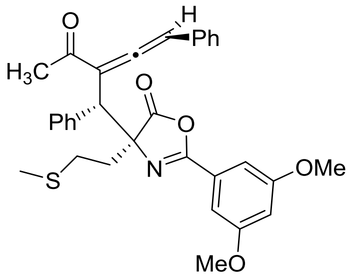

34 mg, 63% yield. ^{1}H NMR (400 MHz, $CDCl_3$) δ 7.56 (d, *J* = 7.3 Hz, 2H), 7.35 (ddt, *J* = 20.2, 14.7, 7.3 Hz, 5H), 7.23 – 7.11 (m, 3H), 6.84 (s, 1H), 6.77 (d, *J* = 2.3 Hz, 2H), 6.53 (t, *J* = 2.2 Hz, 1H), 4.76 (s, 1H), 3.52 (s, 6H), 2.55 – 2.33 (m, 4H), 2.32 (s, 3H), 2.04 (s, 3H). ^{13}C NMR (101 MHz, $CDCl_3$) δ 218.0, 196.7, 178.5, 161.0, 160.8, 137.3, 131.8, 129.6, 129.1, 128.4, 128.2, 128.0, 127.3, 111.6, 106.2, 105.2, 100.9, 76.5, 55.4, 47.6, 35.5, 28.8, 26.9, 15.4. $[\alpha]_D^{25}$ = -295.30 (c 1.0, $CHCl_3$). IR: ν = 3006, 2989, 2035, 1805, 1679, 1651,1 599 cm^{-1}. HRMS: m/z (ESI) calculated for $C_{32}H_{32}NO_5S^+$: 542.1996, found: 542.1993 $[M+H]^+$. HPLC: Chiralpak IA column, 90:10 hexanes/isopropanol, 1 ml/min; t_R = 7.5 min (minor), 9.7 min (major); 94% ee.

tert-butyl(4-((*S*)-4-((*1R,3S*)-2-acetyl-1,4-diphenylbuta-2,3-dien-1-yl)-2-(3,5-dimethoxyphenyl)-5-oxo-4,5-dihydrooxazol-4-yl)butyl)carbamate (**4y**)

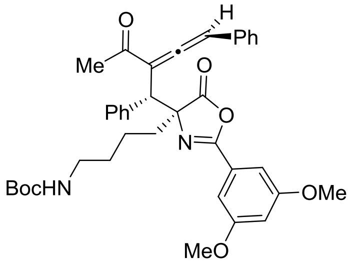

42 mg, 66% yield. ^{1}H NMR (400 MHz, $CDCl_3$) δ 7.52 (d, *J* = 7.4 Hz, 2H), 7.43 – 7.32 (m, 4H), 7.29 (d, *J* = 7.3 Hz, 1H), 7.17 (dd, *J* = 5.0, 1.4 Hz, 3H), 6.82 (s, 1H), 6.77 (d, *J* = 2.2 Hz, 2H), 6.53 (t, *J* = 2.2 Hz, 1H), 4.74 (s, 1H), 4.47 (s, 1H), 3.54 (s, 6H), 3.15 – 2.90 (m, 2H), 2.30 (s, 3H), 2.14 – 1.92 (m, 2H), 1.57 – 1.33 (m, 11H), 1.08 – 1.25 (m, 2H). ^{13}C NMR (101 MHz, $CDCl_3$) δ 217.6, 196.7, 178.8, 160.8, 160.4, 156.0, 137.6, 131.9, 129.7, 129.1, 128.3, 128.1, 127.9, 127.3, 111.7, 106.1, 105.2, 100.9, 79.2, 77.4, 55.4, 47.7, 40.3, 36.2, 30.1, 28.5, 26.9, 21.5. $[\alpha]_D^{25}$ = -221.2 (c 1.0, $CHCl_3$). IR: ν = 3006, 2991, 1715, 1639, 1560 cm^{-1}. HRMS: m/z (ESI) calculated for $C_{38}H_{43}N_2O_7{}^{+}$: 639.3065, found: 639.3065 $[M+H]^+$. HPLC: Chiralpak IA column, 90:10 hexanes/isopropanol, 1 ml/min; t_R = 10.2 min (minor), 12.6 min (major); 98% ee.

Large scale Experiments:

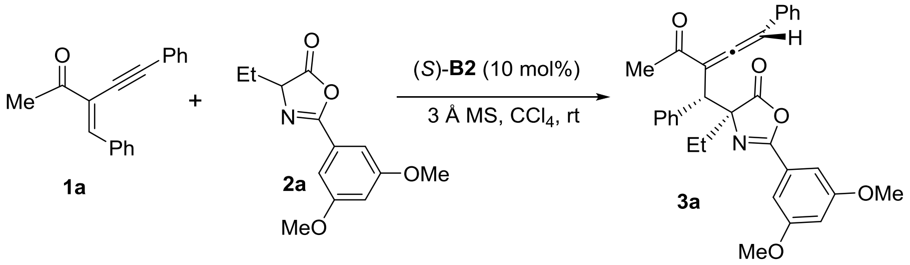

To a dried 50 mL flask was added **1a** (740 mg, 3.0 mmol), **2a** (500 mg, 2.0 mmol), (*S*)-**B2** cat (120 mg, 0.2 mmol), activated 3Å molecular sieves (2.0 g). The flask was purged with N_2 for 3 times and then followed by adding CCl_4 (10 mL). After stirring for 24 h, the reaction mixture was quenched by adding K_2CO_3 and filtered. The filtrate was concentrated under vacuum to give a residue, which was purified by flash column chromatography (PE:EA, 12:1) to give the allene product **3a** (760 mg, 77% yield, 91% ee).

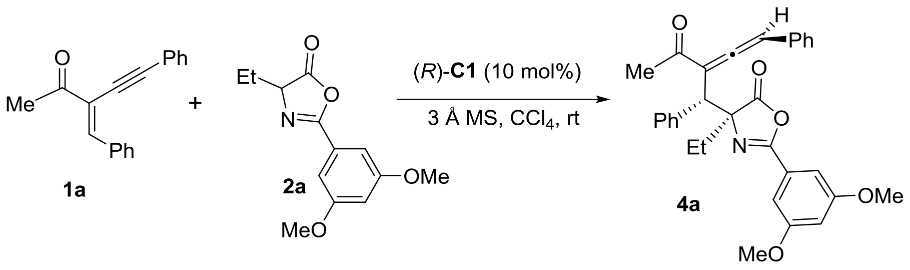

To a dried 50 ml flask was added **1a** (300 mg, 1.2 mmol), **2a** (200 mg, 0.8 mmol), (*R*)-**C1** cat (50mg 0.08 mmol), activated 3Å molecular sieves (0.8 g). The flask was purged with N_2 for 3 times and then followed by adding CCl_4 (4 mL). After stirring for 24 h, the reaction mixture was quenched by adding K_2CO_3 and filtered. The filtrate was then concentrated under vacuum to give a residue, which was purified by flash column chromatography (PE:EA, 12:1) to give the allene product **4a** (290 mg, 75% yield, 97% ee).

Mechanistic Study

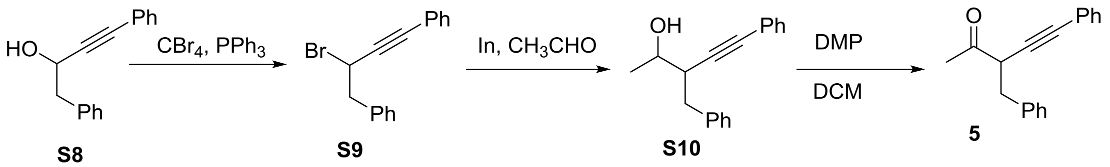

General procedure for synthesis of 5a:

To a solution of CBr_4 (5.3g, 16 mmol) in DCM (50 mL) was added **S8** (2.97 g, 13 mmol) at 0 ^{o}C. After stirring at 0 ℃ for an additional 10 min, PPh_3 (5.3g, 20 mmol) was added and the reaction mixture was allowed to stir at RT. After consumption of the starting material by TLC monitoring, the reaction mixture was concentrated under vacuum gave a residue, which was purified by flash column chromatography (PE) to give the product **S9** (3.8 g, 99%).

S9 (1.9 g, 6.7 mmol) was added to a well stirred suspension of the acetaldehyde (0.66mL, 5.0 M, 3.3 mmol) and indium powder (0.76 g, 6.7 mmol) in THF/H_2O (1:1, 16 mL) at 0 ^{o}C. NH_4Cl (aq. sat.) (23 mL) was added after one hour and the reaction was stirred at room temperature until disappearance of the starting material (TLC). The reaction mixture was extracted with ethyl acetate (3 x 10 mL). The organic extract was washed with brine, dried ($MgSO_4$) and concentrated under reduced pressure. Chromatography of the residue using ethyl acetate/hexanes mixtures gave a mixture of **S10** and allene byproduct. To a solution of the mixture product in DCM (10mL) was added AgOTf (20 mol%, 114 mg) at RT, After stirring for 12h, the reaction mixture was concentrated and purified by flash column

chromatography (PE:EA, 12:1) to give pure product **S10** (252 mg, 15% yield).

To a solution of **S10** (252 mg, 1.0 mmol) in DCM (10 mL) was added Dess-Martin periodinane (848 mg, 2.0 mmol) at RT. After consumption of the starting material by TLC monitoring, the reaction mixture was diluted with DCM and then quenched with adding H_2O. The combined organic layers were dried over Na_2SO_4 and concentrated under vacuum to give a residue, which was added 5 mL PE to give a suspension. After filter through celite, the filtrate was concentrated under vacuum to give **5a** (197 mg, 79% yield) as a green oil. ^{1}H NMR (500 MHz, Chloroform-*d*) δ 7.31 – 7.27 (m, 2H), 7.26 – 7.20 (m, 7H), 7.19 – 7.15 (m, 1H), 3.62 (dd, *J* = 8.5, 5.8 Hz, 1H), 3.13 (dd, *J* = 13.6, 5.8 Hz, 1H), 2.95 (dd, *J* = 13.6, 8.5 Hz, 1H), 2.27 (s, 3H). ^{13}C NMR (126 MHz, $CDCl_3$) δ 204.5, 138.3, 131.6, 129.3, 128.4, 128.3, 126.7, 122.9, 86.2, 85.9, 48.1, 37.2, 28.4. IR: ν = 3069, 3024, 2956, 2922, 2853, 2203, 1715, 1665, 1591 cm^{-1}. HRMS: m/z (ESI) calculated for $C_{18}H_{17}O^+$: 249.1274, found: 249.1271 $[M+H]^+$.

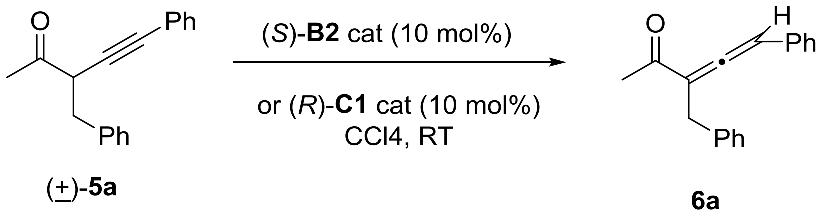

General procedure for asymmetric isomerization of 5a to allene 6a:

To a solution of **5a** (12.5mg 0.05 mmol) in CCl_4 (0.5 mL) was added (*S*)-**B2** cat (3.2 mg, 10 mol%) at RT. After stirring for 5h, the reaction mixture was purified by preparative TLC (PE:EA, 7:1) to give product **6a** (12 mg, 92% yield). HPLC: Chiralpak IC column, 99.7:0.3 hexanes/isopropanol, 1 ml/min; t_R = 17.7 min (major), 18.9 min (minor); 80% ee.

To a solution of **5a** (12.5 mg, 0.05 mmol) in CCl_4 (0.5 mL) was added (*R*)-**C1** cat (2.8 mg, 10 mol%) at RT. After full conversion as indicated by HPLC analysis, the reaction mixture was purified by preparative TLC (PE:EA, 7:1) to give product **6a** (10 mg, 80% yield) HPLC: Chiralpak IC column, 99.7:0.3 hexanes/isopropanol, 1 ml/min; t_R = 16.9 min (minor), 18.2 min (major); 48% ee.

^{1}H NMR (400 MHz, Chloroform-*d*) δ 7.35 – 7.14 (m, 10H), 6.54 (t, *J* = 2.4 Hz, 1H), 3.72 – 3.56 (m, 2H), 2.30 (s, 3H). ^{13}C NMR (101 MHz, $CDCl_3$) δ 215.7, 197.5, 139.3, 131.8, 129.0 129.0, 128.3, 128.0, 127.1, 126.3, 113.0, 99.2, 33.6, 27.3. IR: ν = 3068, 2927, 1797, 1670 cm^{-1}. HRMS: m/z (ESI) calculated for $C_{18}H_{17}O^+$: 249.1274, found: 249.1270 $[M+H]^+$.

Derivatizations of chiral products:

(*S*)-2-(3,5-dimethoxyphenyl)-4-ethyl-4-((*R*)-(2-methyl-5-phenylfuran-3-yl)(phenyl)methyl)oxazol-5(4H)-one (**7a**)

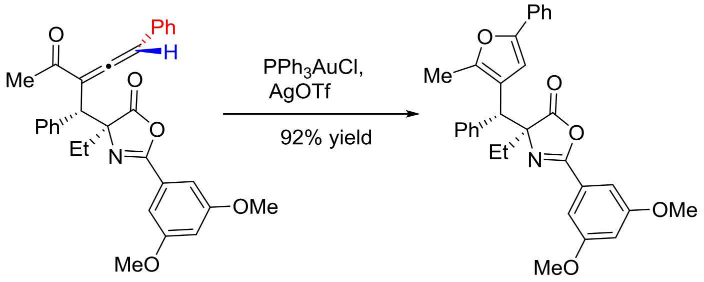

3a, 97% ee

7a 97% ee

To a solution of **3a** (73 mg, 0.15 mmol) in DCM (1 mL) was added a solution of PPh_3AuOTf in DCM (0.015 mmol, in situ prepared by mixing PPh_3AuCl and AgOTf in DCM) at rt. After stirring for 0.5 h, the reaction mixture was purified by preparative TLC (PE:EA, 10:1) to give product **7a** (67 mg, 92% yield).^{1}H NMR (400 MHz, $CDCl_3$) δ 7.72 (d, *J* = 7.4 Hz, 2H), 7.39 (t, *J* = 7.7 Hz, 2H), 7.30 (d, *J* = 4.7 Hz, 3H), 7.25 (d, *J* = 6.9 Hz, 1H), 7.20 – 7.06 (m, 5H), 6.66 (t, *J* = 2.2 Hz, 1H), 4.29 (s, 1H), 3.85 (s, 6H), 2.35 (s, 3H), 2.06 (dt, *J* = 14.9, 7.4 Hz, 1H), 2.01 – 1.89 (m, 1H), 0.82 (t, *J* = 7.4 Hz, 3H). ^{13}C NMR (101 MHz, $CDCl_3$) δ 180.1, 161.0, 160.3, 151.6, 149.2, 138.4, 131.1, 129.3, 128.7, 128.3, 127.6, 127.4, 127.0, 123.6, 119.5, 107.2, 105.8, 105.1, 78.8, 55.8, 49.2, 29.5, 11.9, 8.4. $[\alpha]_D^{25}$ = -188.90 (c 1.0, $CHCl_3$). IR: ν = 3006, 2989, 2032, 1805, 1653, 596 cm^{-1}. HRMS: m/z (ESI) calculated for $C_{31}H_{30}NO_5^+$: 496.2118, found: 496.2113 $[M+H]^+$. HPLC: Chiralpak IC column, 95:5 hexanes/isopropanol, 1 ml/min; t_R = 4.5 min (major), 4.7 min (minor); 97% ee.

Methyl-(*S*)-2-(3,5-dimethoxybenzamido)-2-((*R*)-(2-methyl-5-phenylfuran-3-yl)(phenyl)methyl)butanoate (**8a**)

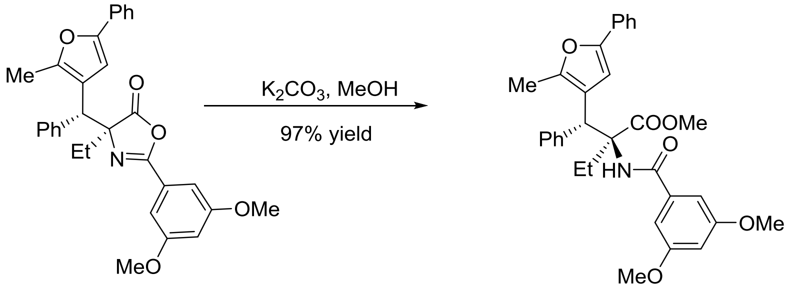

7a 97% ee

8a 98% ee

To a solution of **7a** (40 mg, 0.08 mmol) in MeOH (2mL) was added K_2CO_3 (6mg, 0.04mmol) at RT. After consumption of the starting material by TLC monitoring, the reaction mixture was filtered through celite and the filtrate was concentrated under vacuum to give a residue, which was purified with flash column chromatography (PE:EA, 10:1) to give the product **8a** (39 mg, 97% yield). ^{1}H NMR (400 MHz, $CDCl_3$) δ 7.61 (d, *J* = 7.7 Hz, 2H), 7.44 (d, *J* = 7.5 Hz, 2H), 7.38 – 7.28 (m, 4H), 7.22 (dd, *J* = 13.9, 6.8 Hz, 2H), 7.15 (s, 1H), 6.81 – 6.67 (m, 2H), 6.52 (s, 1H), 5.14 (s, 1H), 3.76 (s, 3H), 3.70 (s, 6H), 2.94 (dq, *J* = 14.5, 7.2 Hz, 1H), 2.18 (s, 3H), 1.95 (dq, *J* = 14.2, 7.1 Hz, 1H), 1.59 (d, *J* = 4.5 Hz, 1H), 0.77 (t, *J* = 7.2 Hz, 3H). ^{13}C NMR (101 MHz, $CDCl_3$) δ 174.0, 166.6, 161.0, 151.6, 150.5, 139.6, 137.9, 131.0, 130.3, 128.7, 128.0, 127.1, 123.4, 119.6, 105.7, 104.5, 104.0, 69.4, 55.6, 52.6, 47.4, 26.9, 11.8, 9.0. $[\alpha]_D^{25}$ = -20.10 (c 1.0, $CHCl_3$). IR: ν = 3404, 2955, 2032, 1805, 1653, 1596 cm^{-1}. HRMS: m/z (ESI) calculated for $C_{32}H_{34}NO_6{}^+$: 528.2381, found: 528.2374 [M+H]$^+$. HPLC: Chiralpak IC column, 95:5 hexanes/isopropanol, 1 ml/min; t_R = 15.2 min (major),19.6 min (minor); 98% ee.

N-((3S,4R,6R)-3-ethyl-6-methyl-2-oxo-4-phenyl-5-((R)-2-phenylvinylidene)tetrahydro-2H-pyran-3-yl) -3,5-dimethoxybenzamide (**9a**)

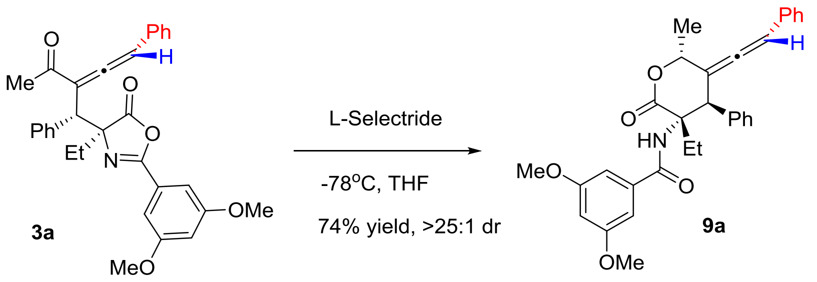

To a solution of **3a** (532mg, 1.0 mmol) in THF (10mL) was added L-Slectride (1.0 M, 1.8 mL, 1.8 mmol) at -78 °C. After consumption of the starting material by TLC monitoring, the reaction was quenched by H_2O and then extracted with EA for 3 times. The combined organic layers were dried over Na_2SO_4 and concentrated under vacuum to give a residue, which was purified with flash column chromatography (PE:EA, 4:1) to give the product **9a** (396 mg, 74% yield). ^{1}H NMR (500 MHz, $CDCl_3$) δ 7.29 (dd, *J* = 7.6, 1.4 Hz, 2H), 7.22 (dd, *J* = 14.2, 7.4 Hz, 5H), 7.16 (d, *J* = 6.6 Hz, 1H), 7.14 – 7.10 (m, 2H), 6.78 (d, *J* = 2.2 Hz, 2H), 6.57 (d, *J* = 1.0 Hz, 1H), 6.39 (dd, *J* = 4.5, 2.9 Hz, 1H), 5.82 (s, 1H), 5.43 (d, *J* = 4.6 Hz, 1H), 5.34 (dd, *J* = 6.3, 2.1 Hz, 1H), 3.79 (d, *J* = 2.1 Hz, 6H), 2.20 (dd, *J* = 14.8, 7.5 Hz, 1H), 1.78 (d, *J* = 6.4 Hz, 3H), 1.69 (dt, *J* = 15.0, 7.5 Hz, 1H), 1.03 (t, *J* = 7.5 Hz, 3H). ^{13}C NMR (126 MHz, $CDCl_3$) δ 201.2, 169.2, 167.7, 161.0, 136.6, 135.7, 133.4, 130.6, 128.8, 128.0, 127.9, 127.7,

126.9, 106.6, 105.1, 103.9, 100.7, 75.8, 62.4, 55.7, 44.5, 26.0, 22.6, 8.4. $[\alpha]_D^{25}$ = 245.40 (c 1.0, $CHCl_3$). IR: ν = 3059, 2989, 2032, 1743, 1664, 1595 cm^{-1}. HRMS: m/z (ESI) calculated for $C_{31}H_{32}NO_5^+$: 498.2275, found: 498.2266 $[M+H]^+$. HPLC: Chiralpak IA column, 80:20 hexanes/isopropanol, 1 ml/min; t_R = 6.8 min (minor), 13.7 min (major); 91% ee.

N-((*3S,4R,6R*)-5-((*R*)-1-hydroxyethyl)-4,7-diphenyl-3-(propylcarbamoyl)hepta-5,6-dien-3-yl)-3,5-dimethoxybenzamide (**10a**)

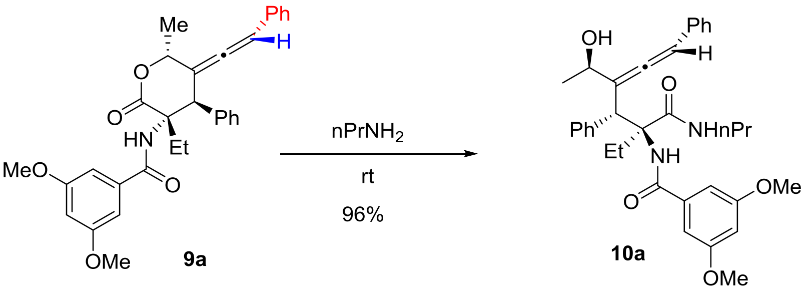

To a flask containing **9a** (50 mg, 0.1 mmol) was added $nPrNH_2$ (0.5 mL) at rt. After stirring for 36 h, he reaction mixture was concentrated under vacuum to give a residue, which was purified with flash column chromatography (DCM) to give the product **10a** (54 mg, 96% yield). ^{1}H NMR (500 MHz, $CDCl_3$) δ 8.35 (s, 1H), 7.56 (dd, *J* = 6.2, 3.0 Hz, 2H), 7.38 – 7.29 (m, 7H), 7.25 – 7.22 (m, 1H), 7.02 (d, *J* = 2.2 Hz, 2H), 6.61 (t, *J* = 2.2 Hz, 1H), 6.50 (s, 1H), 5.16 (t, *J* = 4.9 Hz, 1H), 5.04 (s, 1H), 4.21 (d, *J* = 5.3 Hz, 1H), 3.86 (s, 6H), 3.11 – 2.95 (m, 2H), 2.79 (ddd, *J* = 13.4, 8.3, 5.8 Hz, 1H), 1.27 (dd, *J* = 13.6, 6.6 Hz, 1H), 1.18 (d, *J* = 6.4 Hz, 3H), 1.10 (dd, *J* = 15.9, 8.0 Hz, 2H), 0.71 (t, *J* = 7.2 Hz, 3H), 0.61 (t, *J* = 7.4 Hz, 3H). ^{13}C NMR (126 MHz, $CDCl_3$) δ 202.6, 170.7, 166.3, 161.2, 138.8, 137.9 – 137.5 (m), 134.7 – 134.3 (m), 131.4, 128.9, 128.6, 128.2, 127.6, 127.2, 112.4, 104.9, 103.9, 100.9, 70.2, 67.1, 55.8, 48.9, 41.9, 28.4, 22.8, 22.1, 11.5, 8.1.

3,5-dimethoxy-*N-((1R,2S*)-1-((*2R,5S*)-2-methyl-5-phenyl-2,5-dihydrofuran-3-yl)-1-phenyl-2-(propylcarbamoyl)butan-2-yl)benzamide (**11a**)

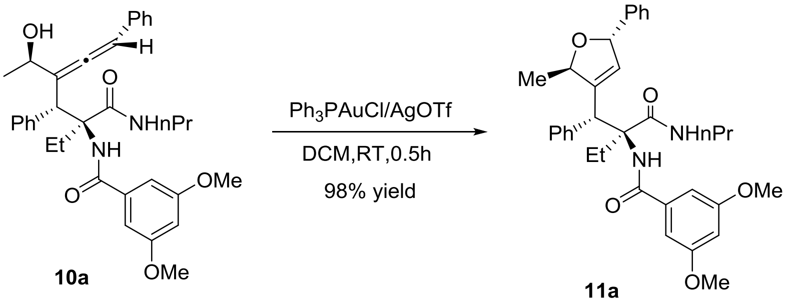

To a solution of **10a** (43 mg, 0.078 mmol) in DCM (1 mL) was added a solution of PPh_3AuOTf in DCM (0.008 mmol, in situ prepared by mixing PPh_3AuCl and AgOTf in DCM) at rt. After stirring for 0.5 h, the reaction mixture was purified by preparative TLC (PE:EA, 4:1) to give product **11a** (42 mg, 98% yield). ^1H NMR (400 MHz, $CDCl_3$) δ 8.18 (s, 1H), 7.51 – 7.31 (m, 7H), 7.31 – 7.21 (m, 4H), 6.97 (d, *J* = 2.1 Hz, 2H), 6.61 (d, *J* = 1.9 Hz, 1H), 5.99 (s, 1H), 5.80 (d, *J* = 5.0 Hz, 1H), 5.16 (s, 1H), 5.08 (s, 1H), 4.70 – 4.56 (m, 1H), 3.86 (s, 6H), 3.11 (m, 3H), 1.37 (dd, *J* = 14.2, 7.1 Hz, 1H), 1.29 – 1.22 (m, 2H), 1.20 (d, *J* = 6.3 Hz, 3H), 0.73 (m, 6H). ^{13}C NMR (101 MHz, $CDCl_3$) δ 171.8, 165.8, 161.1, 142.8, 142.1, 137.9, 136.7, 128.7, 128.6, 128.5, 127.7, 126.4, 125.0, 104.9, 103.7, 87.2, 84.1, 66.1, 55.7, 47.2, 42.0, 28.5, 22.5, 20.7, 11.5, 8.0. $[\alpha]_D^{25}$ = -111.80 (c 1.0, $CHCl_3$). IR: ν =3006, 2989, 1650, 1595 cm^{-1}. HRMS: m/z (ESI) calculated for $C_{34}H_{41}N_2O_5^+$: 557.3010, found: 557.3003 $[M+H]^+$. HPLC: Chiralpak IA column, 90:10 hexanes/isopropanol, 1 ml/min; t_R = 4.6 min (major), 5.5 min (minor); 92% ee.

N-((*1S,4R,6R,10bR*)-1-ethyl-5-iodo-4-methyl-2-oxo-6-phenyl-1,4,6,10b-tetrahydro-2H-benzo[f]isochromen-1-yl)-3,5-dimethoxybenzamide (**12a**)

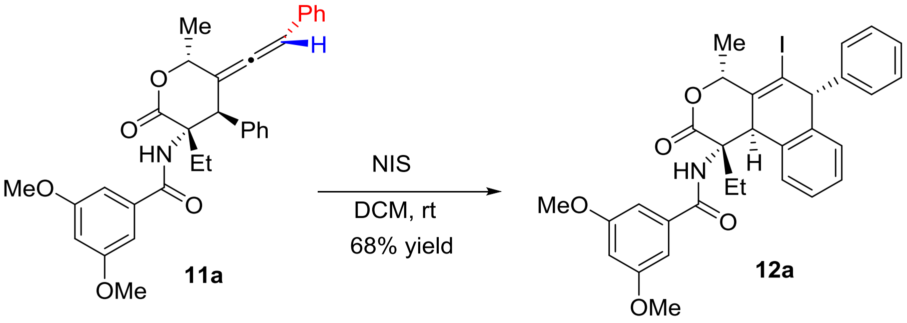

To a solution of **11a** (50 mg, 0.1 mmol) in DCM (1.5 mL) was added NIS (33 mg, 0.15 mmol) at rt. After stirring overnight, the reaction mixture was concentrated under vacuum to give a residue, which was purified with flash column chromatography (PE:EA, 10:1) to give the product **12a** (42 mg, 68% yield). ^1H NMR (400 MHz, $CDCl_3$) δ 7.36 – 7.25 (m, 3H), 7.19 – 7.07 (m, 3H), 7.00 (dd, *J* = 8.7, 4.6 Hz, 4H), 6.91 – 6.83 (m, 1H), 6.66 (d, *J* = 1.9 Hz, 1H), 6.31 (s, 1H), 5.64 (q, *J* = 6.5 Hz, 1H), 5.42 (d, *J* = 3.9 Hz, 1H), 5.01 (d, *J* = 3.8 Hz, 1H), 3.85 (s, 6H), 1.90 – 1.72 (m, 4H), 1.31 (dd, *J* = 14.8, 7.5 Hz, 1H), 0.91 (t, *J* = 7.5 Hz, 3H). ^{13}C NMR (101 MHz, $CDCl_3$) δ 169.2, 167.7, 161.2, 146.2, 138.3, 135.9, 135.4, 130.3, 129.4, 129.1, 128.8, 128.6, 127.9, 127.4, 126.7, 106.7, 105.2, 104.3, 85.7, 64.3, 57.0, 55.8, 40.0, 25.2, 20.3, 8.0. $[\alpha]_D^{25}$ = -15.70 (c 1.0, $CHCl_3$). IR: ν = 3359, 3004, 2963, 2924, 2850, 2048, 1731, 1643, 1589, 1530 cm^{-1}. HRMS: m/z (ESI) calculated for $C_{31}H_{31}INO_5^+$: 624.1241, found: 624.1233

[M+H]$^+$. HPLC: Chiralpak IA column, 90:10 hexanes/isopropanol, 1 ml/min; t_R = 9.2 min (minor), 10.7 min (major); 91% ee.

N-((3*S*,4*S*,6*S*)-3-ethyl-6-methyl-2-oxo-4-phenyl-5-((*S*)-2-phenylvinylidene)tetrahydro-2H-pyran-3-yl)-3,5-dimethoxybenzamide (**13a**)

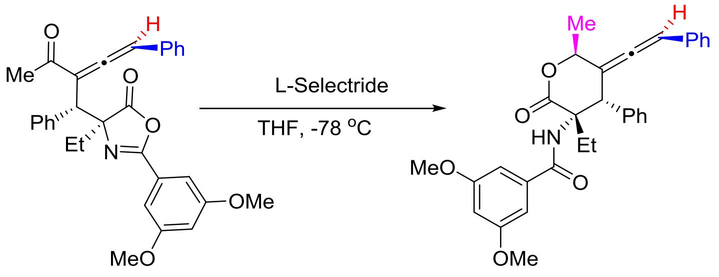

4a 97 % ee **13a**, 75%, 97% ee

To a solution of **4a** (532mg 1.07mmol) in THF (4mL) was added L-Slectride (1.0 M, 1.8mL, 0.25 mmol) at -78°C. After consumption of the starting material by TLC monitoring, the reaction was quenched by H_2O and then extracted with EA for 3 times. The combined organic layers were dried over Na_2SO_4 and concentrated under vacuum to give a residue, which was purified with flash column chromatography (PE:EA, 4:1) to give the product **13a (**400mg, 75% yield). ^{1}H NMR (500 MHz, $CDCl_3$) δ 7.38 (t, *J* = 7.4 Hz, 2H), 7.30 (t, *J* = 8.3 Hz, 3H), 7.20 (dt, *J* = 15.8, 7.5 Hz, 5H), 6.83 (d, *J* = 2.1 Hz, 2H), 6.59 (d, *J* = 2.0 Hz, 1H), 6.33 (t, *J* = 4.0 Hz, 1H), 6.02 (s, 1H), 5.50 (dd, *J* = 5.7, 4.2 Hz, 1H), 4.95 (d, *J* = 4.1 Hz, 1H), 3.81 (s, 6H), 2.13 (dd, *J* = 14.3, 7.4 Hz, 1H), 1.76 (dd, *J* = 14.4, 7.4 Hz, 1H), 1.59 (d, *J* = 6.3 Hz, 3H), 1.10 (t, *J* = 7.5 Hz, 3H). ^{13}C NMR (126 MHz, $CDCl_3$) δ 201.9, 169.6, 167.5, 161.1, 135.9, 135.7, 133.4, 130.8, 129.2, 128.0, 128.0, 128.0, 127.0, 106.4, 105.1, 104.1, 101.2, 76.4, 62.5, 55.8, 49.2, 27.3, 19.6, 8.7. $[\alpha]_D^{25}$ = -276.00 (c 1.0, $CHCl_3$). IR: ν = 3006, 2989, 2032, 1743, 1664, 1595 cm^{-1}. HRMS: m/z (ESI) calculated for $C_{31}H_{32}NO_5^+$: 498.2275, found: 498.2265 [M+H]$^+$. HPLC: Chiralpak IA column, 80:20 hexanes/isopropanol, 1 ml/min; t_R = 8.2 min (minor), 9.8 min (major); 97% ee.

N-((3*S*,4*R*,6*S*)-5-((*S*)-1-hydroxyethyl)-4,7-diphenyl-3-(propylcarbamoyl)hepta-5,6-dien-3-yl)-3,5-dimethoxybenzamide (**14a**)

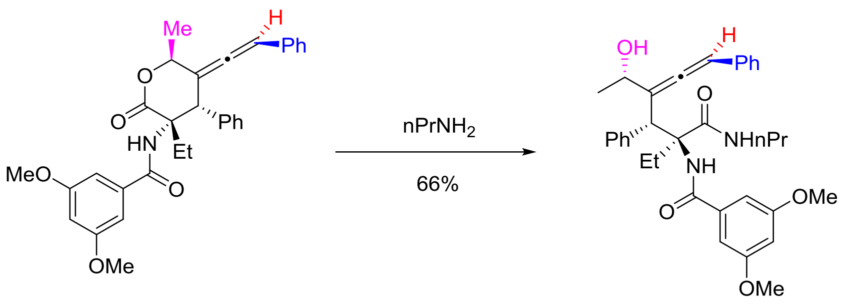

13a, 97% ee

14a

To a flask containing **13a** (50 mg, 0.1 mmol) was added nPrNH$_2$ (0.5 mL) at rt. After stirring for 36 h, the reaction mixture was concentrated under vacuum to give a residue, which was purified with flash column chromatography (DCM) to give the product **14a** (37 mg, 66% yield). ^{1}H NMR (500 MHz, CDCl$_3$) δ 8.25 (s, 1H), 7.58 (d, J = 7.1 Hz, 2H), 7.39 (dt, J = 21.8, 7.1 Hz, 3H), 7.14 (d, J = 7.1 Hz, 2H), 7.10 (t, J = 7.3 Hz, 1H), 7.02 (t, J = 7.5 Hz, 2H), 6.94 (d, J = 2.2 Hz, 2H), 6.63 (t, J = 2.2 Hz, 1H), 6.46 (s, 1H), 5.31 (s, 1H), 4.98 (t, J = 5.2 Hz, 1H), 4.11 – 4.02 (m, 1H), 3.81 (s, 6H), 3.08 – 2.88 (m, 3H), 1.51 (dd, J = 14.0, 7.1 Hz, 1H), 1.36 (ddd, J = 17.1, 14.1, 6.7 Hz, 2H), 1.14 (d, J = 6.3 Hz, 3H), 0.79 (t, J = 7.4 Hz, 3H), 0.68 (t, J = 7.3 Hz, 3H). ^{13}C NMR (126 MHz, CDCl$_3$) δ 202.3, 170.3, 166.3, 161.1, 138.3, 137.2, 134.2, 131.6, 128.6, 128.6, 128.4, 127.3, 127.2, 111.8, 104.9, 104.5, 101.0, 68.9, 66.8, 55.7, 50.7, 41.9, 28.9, 22.4, 21.8, 11.6, 8.1. $[\alpha]_D^{25}$ = -178.80.

3,5-dimethoxy-*N*-((*1R*,*2S*)-1-((*2S*,*5R*)-2-methyl-5-phenyl-2,5-dihydrofuran-3-yl)-1-phenyl-2-(propylcarbamoyl)butan-2-yl)benzamide (**15a**)

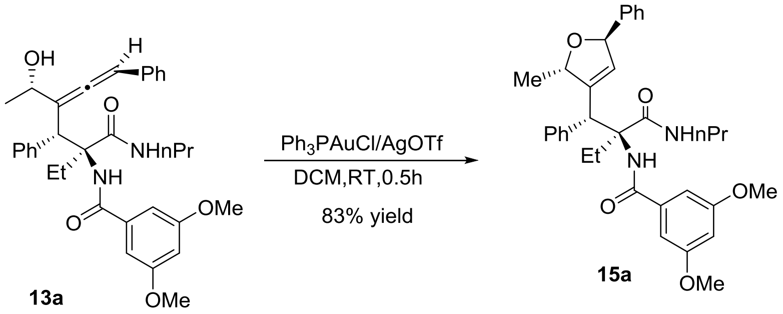

13a

15a

To a solution of **13a** (30 mg, 0.053 mmol) in DCM (1 mL) was added a solution of PPh$_3$AuOTf in DCM (0.005 mmol, in situ prepared by mixing PPh$_3$AuCl and AgOTf in DCM) at rt. After stirring for 0.5 h, the reaction mixture was purified by preparative TLC (PE:EA, 3:1) to give product **15a** (25 mg, 83% yield). ^{1}H NMR (500 MHz, CDCl$_3$) δ 8.20 (s, 1H), 7.52 (d, J = 7.0 Hz, 2H), 7.44 – 7.32 (m, 3H), 7.13 (t, J = 7.1 Hz, 1H), 7.08 (d, J = 7.0 Hz, 2H), 7.01 (t, J = 7.2 Hz, 2H), 6.94 (s, 2H), 6.62 (s, 1H),

5.89 (s, 1H), 5.80 (d, J = 4.8 Hz, 1H), 5.17 (s, 1H), 4.97 (m, 2H), 3.79 (s, 6H), 3.20 (dd, J = 13.0, 6.5 Hz, 1H), 3.15 – 3.01 (m, 2H), 1.54 (dd, J = 14.1, 7.1 Hz, 1H), 1.39 (dt, J = 14.2, 7.0 Hz, 2H), 0.82 (m, 6H), 0.74 (t, J = 6.8 Hz, 3H). ^{13}C NMR (126 MHz, $CDCl_3$) δ 171.4, 165.4, 161.1, 142.0, 141.8, 137.1, 137.0, 131.7, 128.4, 128.4, 128.3, 127.8, 126.8, 125.5, 104.7, 104.4, 88.0, 86.1, 66.9, 55.7, 47.6, 42.0, 28.2, 22.5, 20.9, 11.5, 8.2. $[\alpha]_D^{25}$ = -41.20 (c 1.0, $CHCl_3$). IR: ν = 3006, 2989, 1650, 1595 cm^{-1}. HRMS: m/z (ESI) calculated for $C_{34}H_{41}N_2O_5^+$: 557.3010, found: 557.3001 $[M+H]^+$. HPLC: Chiralpak IA column, 90:10 hexanes/isopropanol, 1 ml/min; t_R = 4.2min (major), 5.6min (minor); 97% ee.

(*1R,5S,8S,9S*)-3-(3,5-dimethoxyphenyl)-5-ethyl-1-((*Z*)-1-iodo-2-phenylvinyl)-8-methyl-9-phenyl-2,7-dioxa-4-azabicyclo[3.3.1]non-3-en-6-one (**16a**)

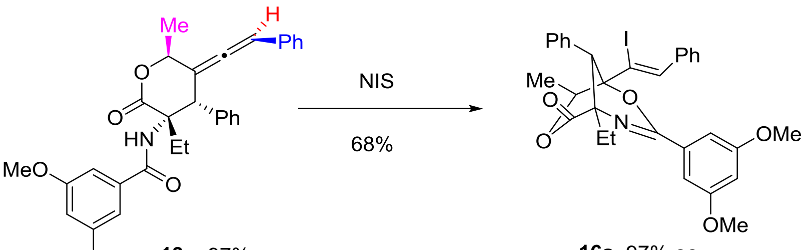

13a, 97% ee **16a**, 97% ee

To a solution of **13a** (20 mg, 0.04 mmol) in DCM (0.5 mL) was added NIS (15 mg, 0.067 mmol) at rt. After stirring overnight, the reaction mixture was concentrated under vacuum to give a residue, which was purified preparative TLC (PE:EA, 4:1) to give the product **16a** (17 mg, 68% yield). ^{1}H NMR (400 MHz, $CDCl_3$) δ 7.41 (m, 7H), 7.34 – 7.27 (m, 3H), 7.19 – 7.08 (m, 2H), 6.94 (s, 1H), 6.62 (t, J = 2.3 Hz, 1H), 5.14 (q, J = 6.9 Hz, 1H), 3.85 (s, 6H), 3.57 (s, 1H), 2.34 (dd, J = 14.2, 7.4 Hz, 1H), 1.75 (d, J = 7.0 Hz, 3H), 1.27 (dd, J = 14.3, 7.0 Hz, 1H), 0.86 (t, J = 7.4 Hz, 3H). ^{13}C NMR (101 MHz, $CDCl_3$) δ 168.9, 160.6, 157.1, 139.9, 138.1, 135.0, 133.7, 129.5, 128.7, 128.4, 128.3, 128.2, 107.9, 105.8, 104.6, 85.2, 81.5, 62.9, 55.7, 50.2, 26.9, 20.5, 6.7. $[\alpha]_D^{25}$ = 32.0 (c 1.0, $CHCl_3$). IR: ν =3359, 3056, 2969, 1741, 1647, 1589 cm^{-1}. HRMS: m/z (ESI) calculated for $C_{31}H_{31}INO_5^+$: 624.1241, found: 624.1234 $[M+H]^+$. HPLC: Chiralpak IA column, 90:10 hexanes/isopropanol, 1 ml/min; t_R = 5.3min (major), 13.5 min (minor); 97% ee.

Computational Studies

Computational Methods:

All theoretical calculations were performed with the Gaussian 09[11] program. All structures were completely optimized at the M06-2X[12,13]/6-31G(d) level of theory. The single point energies were further estimated using a larger basis set 6-311++G(d,p) for all atoms with the SMD solvation model[14]. All optimized species were verified as either minima or transition structures by the presence of zero or a single imaginary vibrational frequency. Free energies were evaluated at 298K using harmonic vibrational frequencies. Saddle points were connected to minima in the usual way with intrinsic reaction coordinate (IRC) calculations[15,16]. The ee values and dr values were computed based on the Boltzmann distribution[17]. The EDA-NOCV analysis[18,19] was carried out using the ADF (2019.103) program package[20,21] at the B3LYP-D3/TZ2P[22] level. The frozen core approximation was not employed in these computations. The calculated structures were displayed with the CYLview software[23]. Noncovalent interactions (NCI) shown in VMD 1.9.3 program[24] was employed to gain more insights into the important noncovalent interactions that are present in the proton transfer transition states.

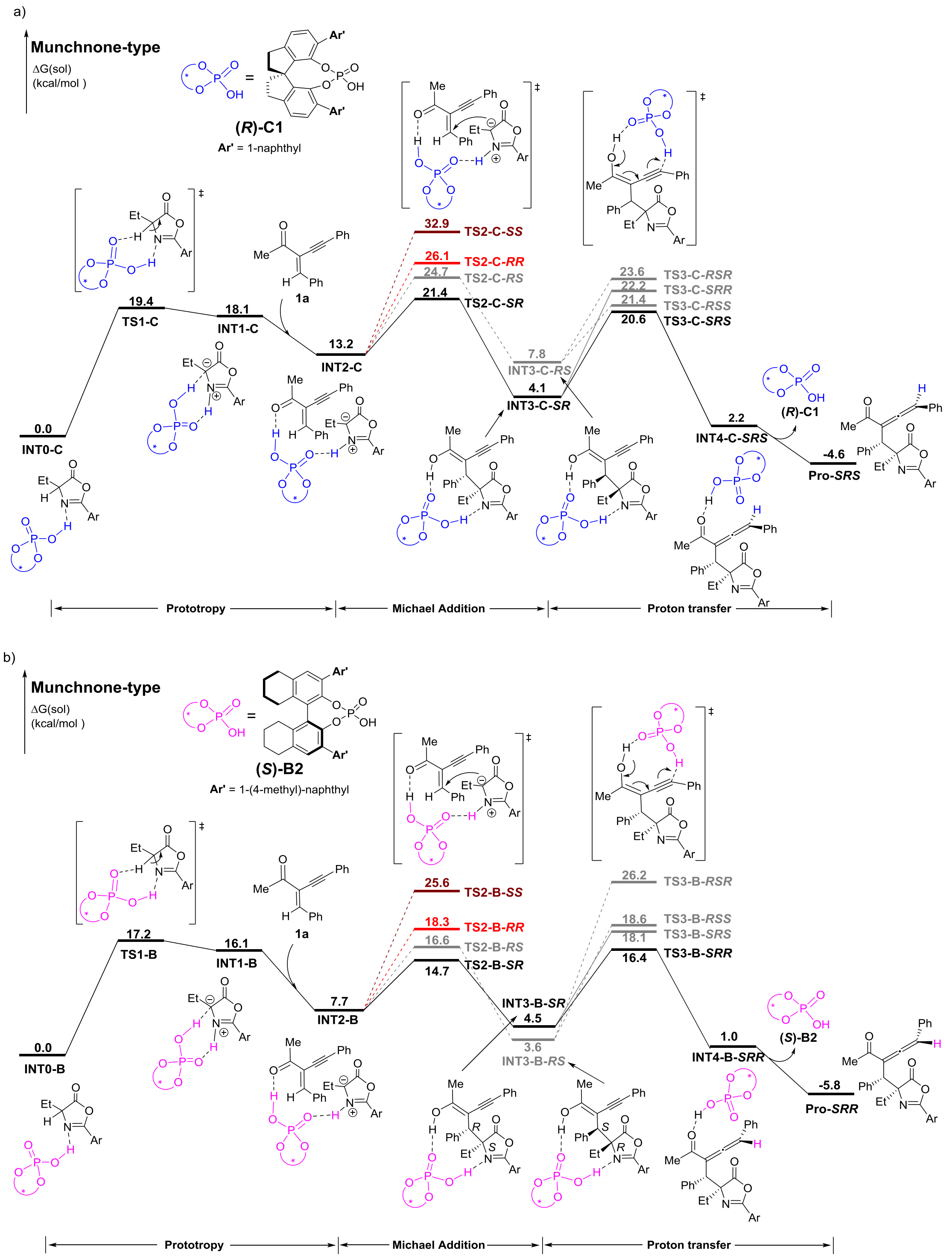

Supplementary Figure 3. Full reaction pathway computed by DFT based on Münchnone-type catalytic mechanism.

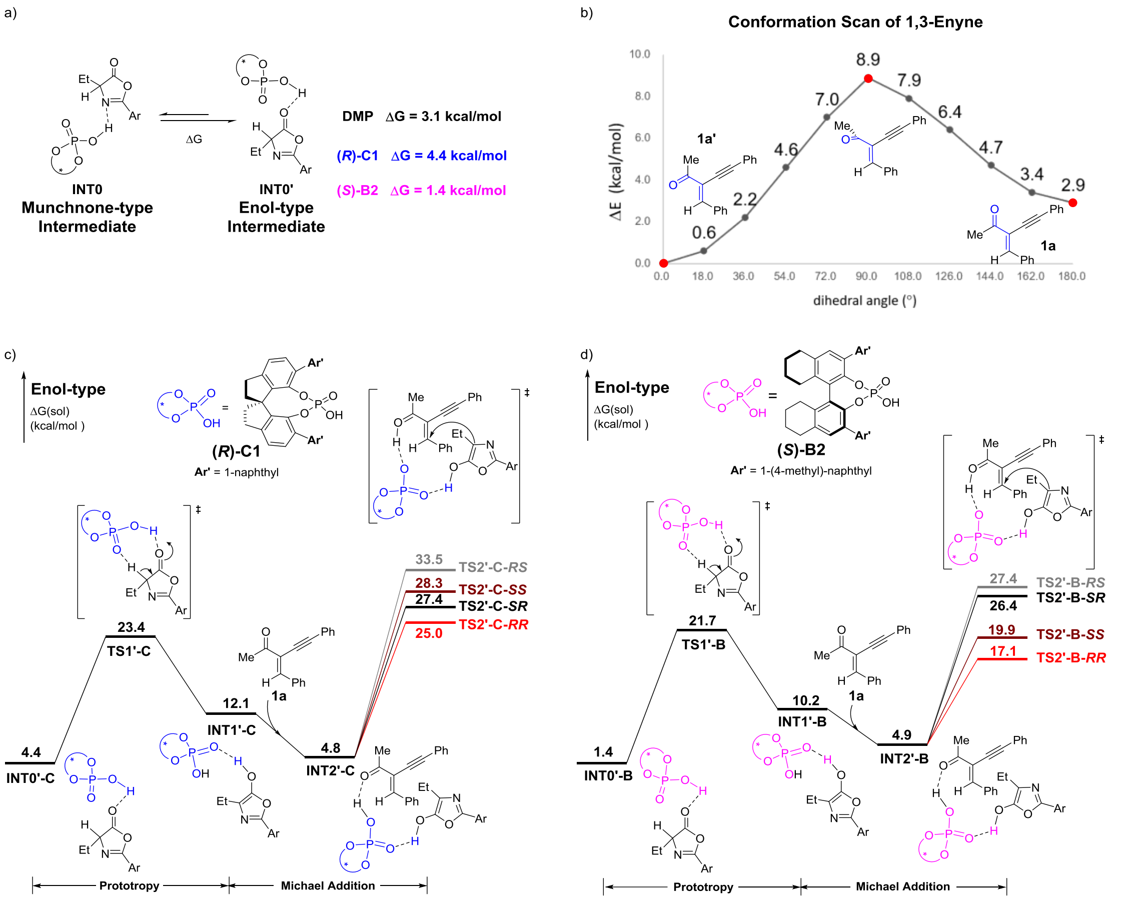

Supplementary Figure 4. a) The comparison of intermediates between enol-type and Münchnone-type. b) Conformation scan of the substrate 1,3-enyne. **1a** is 2.9 kcal/mol less stable than **1a'** in electron energy, which would require 6.0 kcal/mol turn over the electron energy barrier to get **1a'**. c) Gibbs free energy profiles for (*R*)-**C1** catalyzed reaction via enol-type catalytic model. d) Gibbs free energy profiles for (*S*)-**B2** catalyzed reaction via enol-type catalytic model.

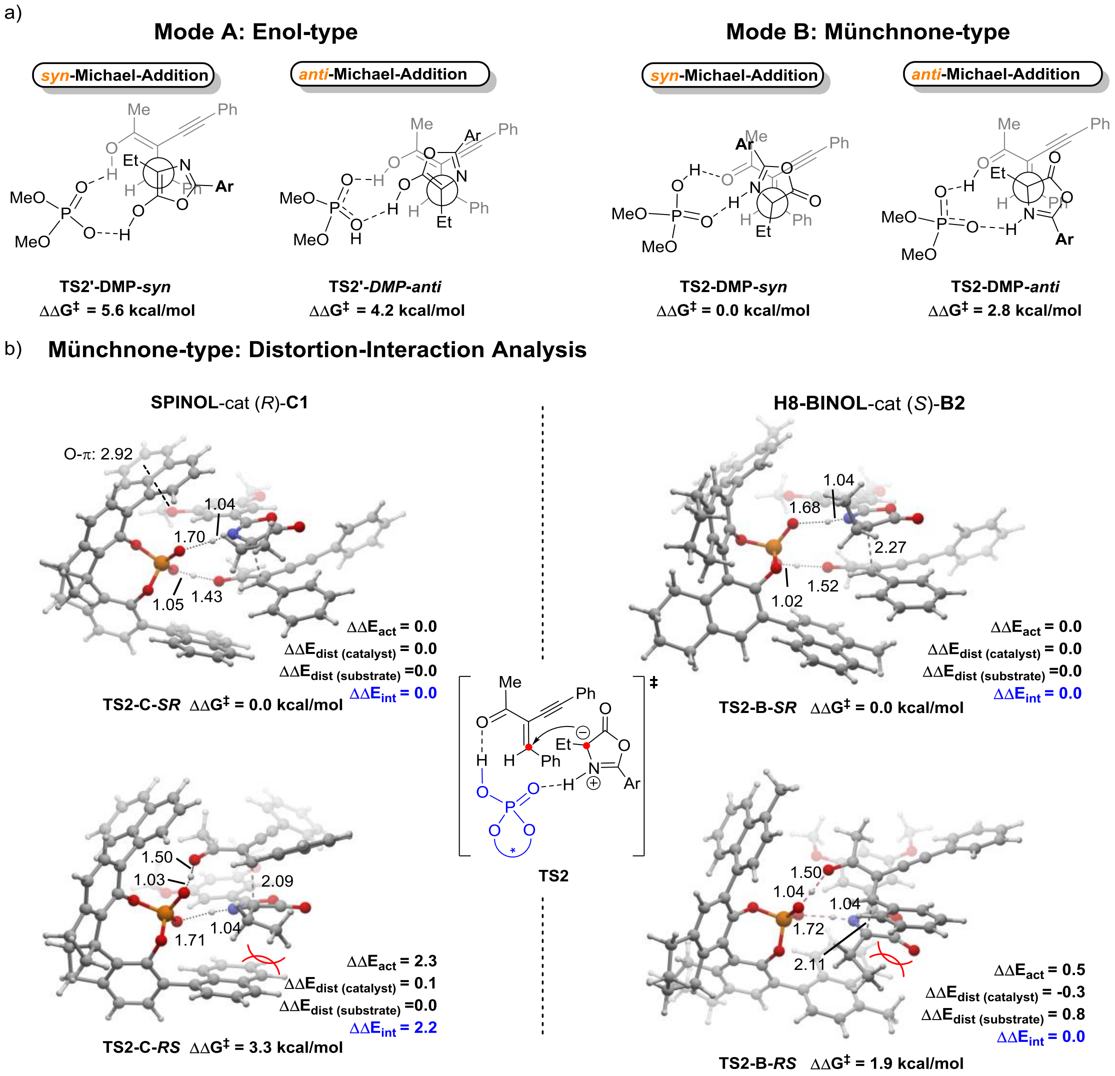

Supplementary Figure 5. (a) Comparison of two possible activation modes for Michael addition step based on achiral dimethyl phosphate (DMP) model catalyst. In Enol-type, the *anti*-Michael-addition-TS is 1.4 kcal/mol lower than *syn*-Michael-addition-TS, which was not in agreement with the experimental *syn*-addition observations. In Münchnone-type, the *syn*-Michael-addition-TS is 2.8 kcal/mol more stable than *anti*-Michael-addition-TS, suggesting the correct *syn*-addition outcomes. (b) Distortion-interaction analysis[25-29] of Michael-Addition TS performed at M06-2X/6-311++G(d,p) level of theory in Gaussian 09. For **TS2-C-*SR*** and **TS2-C-*RS***, the distortion-interaction analysis reveals that the major contribution of activation energy ($\Delta\Delta E_{act}$) is from the interaction energies ($\Delta\Delta E_{int}$) between (*R*)-**C1** catalyst fragment and substrate fragment, probably because of the steric repulsion between 3,3'-substitutent of C1 catalyst and the ethyl group of **2a** in **TS2-C-*RS***, which indicates that the (*R*)-**C1** catalyst can differentiate the substrates with different configurations via interactions between catalyst and substrate in Michael addition transition states. In contrast, the distortion-interaction analysis of **TS2-B-*SR*** and **TS2-B-*RS*** shows that (*S*)-**B2** catalyst cannot easily distinguish the different configurations in Michael addition transition states by interacting with substrates. Furthermore, the substrate distortion destabilizing **TS2-B-*RS*** comes mainly from sterically repulsive interactions between the phenyl group (R^2) at the beta position of 1a and the ethyl group of **2a**. And these delicate differences of transition states for stereoselectivity may originate from the flexible conformation of the two catalysts.

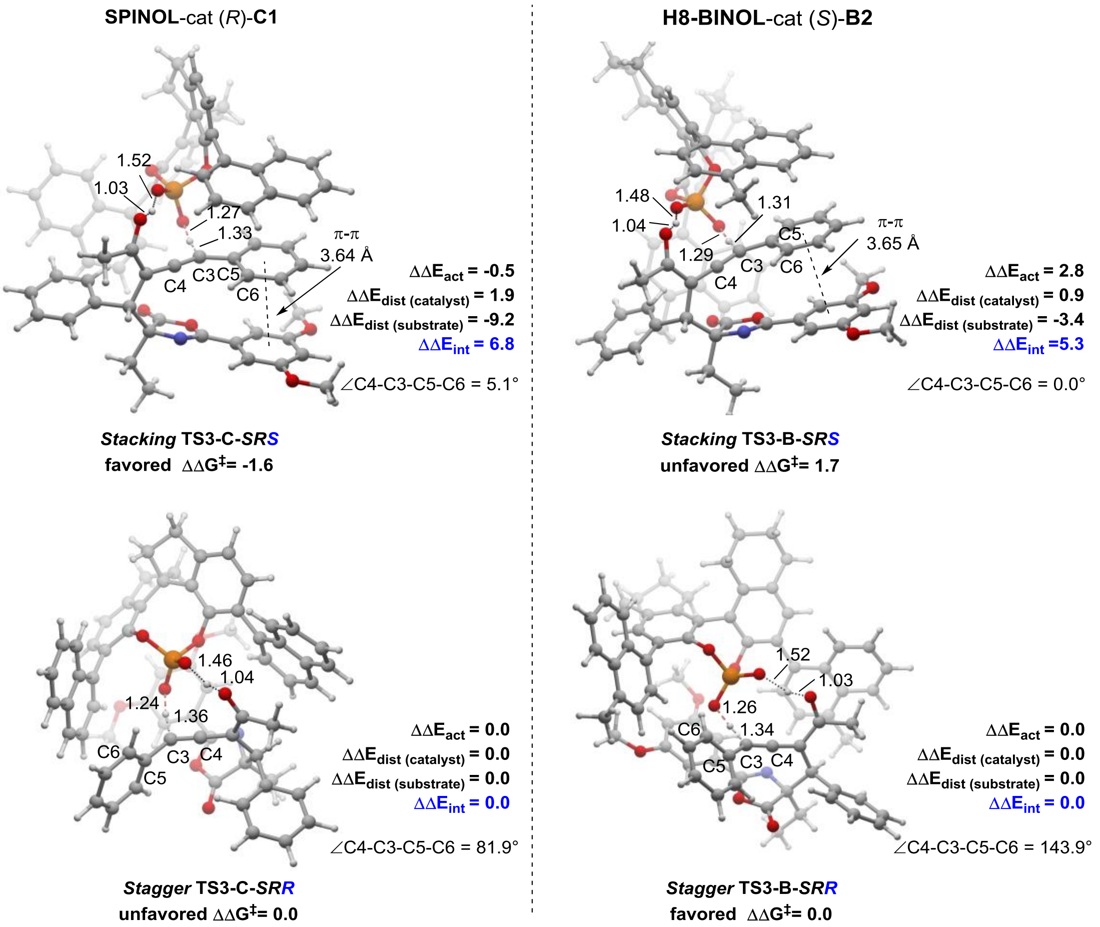

Supplementary Figure 6. Distortion-interaction analysis of TSs for proton transfer performed at M06-2X/6-311++G(d,p) level of theory in Gaussian 09. For both the catalysts (*R*)-SPINOL-**C1** and (*S*)-H8-BINOL-**B2**, the distortion-interaction analysis reveals that the interactions between catalyst fragment and substrate fragment are more favorable in *stagger*-type TSs. And the distortion energies of substrate fragment are lower in *stacking*-type TSs, which indicate that the substrate intermediate has strong intramolecular interactions. In addition, focusing on the direction of the phenyl group at the R^1 position of α-alkynyl enones **1a**, the dihedral angles ∠ C4-C3-C5-C6 of phenyl group (R^1) and the allenic moiety in *stacking*-type TSs, which are closer to planar than that in *stagger*-type TSs. The coplanarity of the dihedral angle results in the resonance stabilization between the phenyl group (R^1) and the allenic moiety, which also decreases the distortion energy of substrate fragment in *stacking*-type TSs.

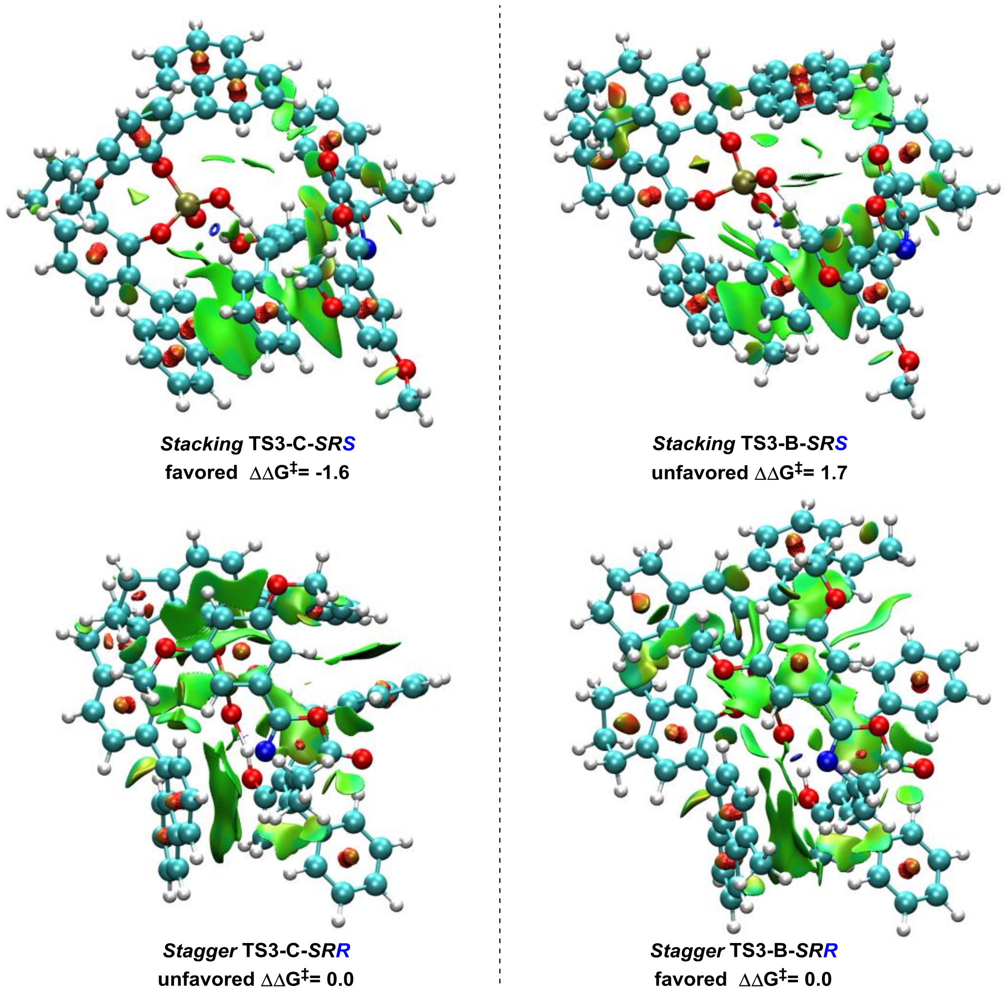

Supplementary Figure 7. NCIs analysis of transition state for proton transfer (green, dispersion effect; red, steric effect). There is weak π-π stacking between allenic moiety (include phenyl group) of **1a** and oxazolone moiety (include Ar group) of **2a** in *stacking*-type TSs. And, there are clear dispersion effect between the naphthyl or 1-(4-Me)-naphthyl group of the catalysts and acetyl group of **1a**, as well as between spiro-ring or bi-naphthyl moiety of catalyst and 3,5-$(MeO)_2C_6H_3$ group of **2a** in *stagger*-type TSs. Therefore, the intramolecular interactions mainly exist in *stacking*-type TSs, and intermolecular interactions mainly occur in *stagger*-type TSs.

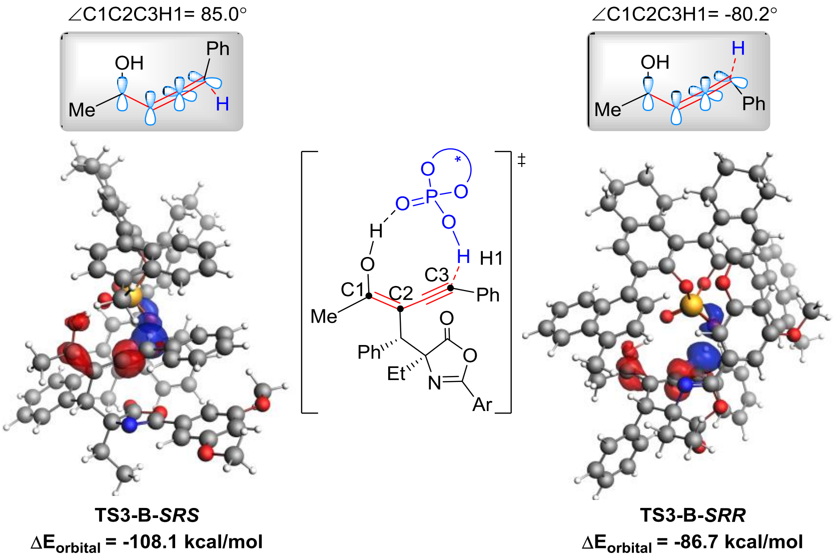

Supplementary Figure 8. Orbital interaction in (*S*)-**B2** catalyzed proton transfer transition states.

Supplementary Table 2. Relative activation energies (in kcal/mol) for Michael-addition transition states.

Transition States	Conformation	ΔE	ΔH	ΔG	ΔΔG
**TS2-DMP-*syn***	**Conf 1**	1.4	1.1	20.2	0.0
**TS2-DMP-*syn***	**Conf 2**	3.6	3.3	24.0	3.8
**TS2-DMP-*anti***	**Conf 1**	7.1	5.6	23.0	2.8
**TS2-DMP-*anti***	**Conf 2**	8.0	7.1	28.7	8.5
**TS2’-DMP-*syn***	**Conf 1**	8.4	6.0	25.8	5.6
**TS2’-DMP-*syn***	**Conf 2**	9.3	9.6	26.6	6.4
**TS2’-DMP-*anti***	**Conf 1**	5.8	3.8	24.4	4.2
**TS2’-DMP-*anti***	**Conf 2**	7.1	5.8	26.8	6.6
**TS2-C-*SR***	**Conf 1**	0.6	0.7	21.4	0.0
**TS2-C-RS**	**Conf 1**	2.9	3.4	24.7	3.3
**TS2-C-*RR***	**Conf 1**	8.7	8.1	26.1	4.7
**TS2-C-*SS***	**Conf 2**	10.3	10.9	32.9	11.5
**TS2’-C-*SR***	**Conf 1**	8.5	6.6	27.4	6.0
**TS2’-C-*SR***	**Conf 2**	11.6	11.9	30.1	8.7
**TS2’-C-*RS***	**Conf 2**	14.4	15.0	33.5	12.1
**TS2’-C-*RR***	**Conf 1**	7.8	6.2	25.0	3.6
**TS2’-C-*RR***	**Conf 2**	7.9	6.6	26.8	5.4
**TS2’-C-*SS***	**Conf 1**	11.5	9.2	28.2	6.8
**TS2-B-*SR***	**Conf 1**	-1.2	-1.1	14.7	0.0
**TS2-B-*RS***	**Conf 1**	-2.4	-2.1	16.6	1.9
**TS2-B-*RR***	**Conf 1**	2.6	1.5	18.3	3.6
**TS2-B-*SS***	**Conf 2**	8.0	6.5	25.6	10.9
**TS2’-B-*SR***	**Conf 1**	6.5	5.4	24.8	11.1
**TS2’-B-*SR***	**Conf 2**	7.6	7.0	26.4	11.7
**TS2’-B-*RS***	**Conf 2**	10.6	10.1	27.4	12.7
**TS2’-B-*RR***	**Conf 1**	2.6	-0.6	17.1	2.4
**TS2’-B-*RR***	**Conf 2**	6.6	4.6	26.1	11.4
**TS2’-B-*SS***	**Conf 1**	4.5	2.0	19.9	5.2

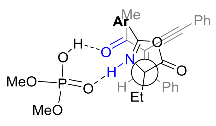
TS2-DMP-*syn* (TS2-C/B-*SR*/*RS*) Conf 1

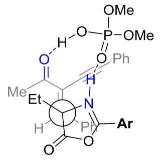
TS2-DMP-*syn* Conf 2

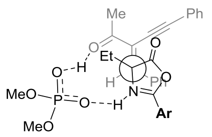
TS2-DMP-*anti* (TS2-C/B-*RR*) Conf 1

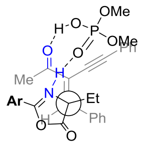
TS2-DMP-*anti* (TS2-C/B-*SS*) Conf 2

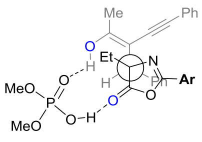
TS2'-DMP-*syn* (TS2'-C/B-*SR*) Conf 1

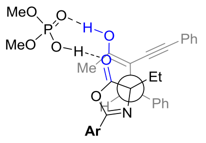
TS2'-DMP-*syn* (TS2'-C/B-*RS*/*SR*) Conf 2

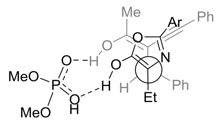
TS2'-DMP-*anti* (TS2'-C/B-*RR*) Conf 1

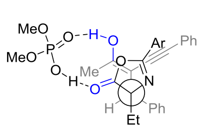
TS2'-DMP-*anti* (TS2'-C/B-*RR*) Conf 2

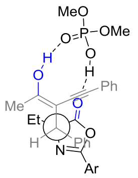
TS2'-C/B-*SS* Conf 1

Supplementary Table 3. Relative activation energies (in kcal/mol) for Proton transfer transition states.

Transition States	Mode	Conformation	ΔE	ΔH	ΔG	ΔΔG
**TS3-C-*SRS***	***stacking***	**Conf 1**	2.4	0.6	20.6	0.0
**TS3-C-*SRS***	***stacking***	**Conf 2**	5.9	3.5	22.8	2.2
**TS3-C-*SRS***	***stacking***	**Conf 3**	3.4	0.8	24.0	3.4
**TS3-C-*SRS***	***stagger***		10.6	9.0	28.7	8.1
**TS3-C-*SRR***	***stacking***		8.9	1.9	27.2	6.4
**TS3-C-*SRR***	***stagger***	**Conf 1**	2.9	0.4	22.2	1.6
**TS3-C-*SRR***	***stagger***	**Conf 2**	4.4	2.3	24.0	3.4
**TS3-C-*SRR***	***stagger***	**Conf 3**	6.0	3.7	26.0	5.4
**TS3-C-*RSS***	***stacking***	**Conf 1**	1.4	-0.6	21.4	0.8
**TS3-C-*RSS***	***stacking***	**Conf 2**	5.6	3.5	24.9	4.3
**TS3-C-*RSR***	***stacking***		11.3	8.8	28.6	8.0
**TS3-C-*RSR***	***stagger***	**Conf 1**	7.3	4.9	23.6	3.0
**TS3-C-*RSR***	***stagger***	**Conf 2**	8.4	6.1	25.5	4.9
**TS3-B-*SRR***	***stagger***		-3.2	-5.7	16.4	0.0
**TS3-B-*SRR***	***stacking***		0.8	-1.9	20.5	4.1
**TS3-B-*SRS***	***stagger***		8.4	6.2	24.7	8.3
**TS3-B-*SRS***	***stacking***		-0.4	-3.0	18.1	1.7
**TS3-B-*RSR***	***stagger***		8.0	5.5	26.2	9.8
**TS3-B-*RSS***	***stacking***	**Conf 1**	0.6	-2.0	18.6	2.2
**TS3-B-*RSS***	***stacking***	**Conf 2**	2.7	0.2	24.5	8.1

Supplementary Table 4. Relative energy components (in kcal/mol) for proton transfer transition states.

TS3	[a]$\Delta E_{dist(catalyst)}$	[a]$\Delta E_{dist(substrate)}$	ΔE_{pauli}	ΔE_{elstat}	ΔE_{orbit}	ΔE_{disper}
**TS3-C-*SRS***	-12153.83	-11129.12	122.90	-60.07	-102.79	-25.81
**TS3-C-*SRR***	-12155.52	-11117.09	137.67	-69.18	-99.69	-40.12
**TS3-B-*SRS***	-14303.33	-11127.28	123.56	-61.03	-108.10	-25.92
**TS3-B-*SRR***	-14305.87	-11134.40	121.76	-58.77	-86.68	-44.39

[a]The ΔE_{dist} values are the absolute energy rather than relative energy respect to free catalyst and substrate, respectively, which is due to **TS3-C-*SRS*** and **TS3-C-*SRR*** (or **TS3-B-*SRS*** and **TS3-B-*SRR***) are stemmed from the same catalyst precursor and reactant precursor.

Supplementary Table 5. Calculated free energies of activation (in kcal/mol) for proton transfer transition states in stereodivergent synthesis of trisubstituted allenes for substrates with different R^2/R^3 substituents.

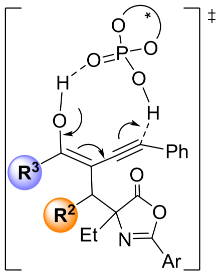

TS3	R^2	R^3	exp [a]d.r.	exp [b]$\Delta\Delta G^{\ddagger}$	calc [b]$\Delta\Delta G^{\ddagger}$
**TS3-B-*SRS***<br>**TS3-B-*SRR***	Ph	Me	20:1	1.8	1.7
**TS3-B-*SRS*-Cy**<br>**TS3-B-*SRR*-Cy**	Cy	Me	4:1	0.8	1.1
**TS3-B-*SRS*-nPr**<br>**TS3-B-*SRR*-nPr**	Ph	nPr	23:1	1.9	3.0
**TS3-C-*SRS***<br>**TS3-C-*SRR***	Ph	Me	12:1	1.5	1.6
**TS3-C-*SRS*-Cy**<br>**TS3-C-*SRR*-Cy**	Cy	Me	13:1	1.5	1.8

[a]In **B2** catalysis, the diastereoselective ratio is **3** (major) : **4** (minor), and in **C1** catalysis, the diastereoselective ratio is **4** (major) : **3** (minor). [b]The value $\Delta\Delta G^{\ddagger}$ in **TS3-B** is $\Delta G^{\ddagger}_{\textit{stacking}\text{-TS}}$ - $\Delta G^{\ddagger}_{\textit{stagger}\text{-TS}}$, and the value $\Delta\Delta G^{\ddagger}$ in **TS3-C** is $\Delta G^{\ddagger}_{\textit{stagger}\text{-TS}}$ - $\Delta G^{\ddagger}_{\textit{stacking}\text{-TS}}$.

Supplementary Table 6. Zero-point correction (ZPE), electronic energies (E), enthalpies (H), and Gibbs free energies (G) (in Hartree) of the structures calculated and imaginary frequency (cm^{-1}) of transition state structures at M06-2X-SMD(CCl_4)/6-311++G(d,p)//M06-2X/6-31G(d) level of theory.

Structures	ZPE	E	H	G	E(solvent)	Imaginary Frequency
**INT0-C**	0.823266	-2927.608666	-2926.735171	-2926.869147	-2928.370527	
**TS1-C**	0.819119	-2927.576159	-2926.707451	-2926.83913	-2928.337059	-922.8
**INT1-C**	0.822731	-2927.578324	-2926.705403	-2926.83891	-2928.341561	
**INT2-C**	1.091344	-3696.794219	-3695.634937	-3695.807085	-3697.774740	
**1a**	0.266385	-769.171047	-768.887284	-768.951293	-769.397331	
**TS2-C-*SR***	1.091393	-3696.790825	-3695.633502	-3695.798481	-3697.766825	-272.9
**TS2-C-*RS***	1.236960	-3696.786793	-3695.628689	-3695.792806	-3697.763172	-281.8
**TS2-C-*RR***	1.089613	-3696.774652	-3695.618402	-3695.787762	-3697.753935	-335.2
**TS2-C-*SS***	1.092478	-3696.774727	-3695.616427	-3695.779337	-3697.751491	-174.0
**INT3-C-*SR***	1.093309	-3696.814608	-3695.654614	-3695.824838	-3697.791873	
**INT3-C-*RS***	1.093741	-3696.812526	-3695.652597	-3695.819371	-3697.789234	
**TS3-C-*SRS***	1.088380	-3696.786818	-3695.632452	-3695.798664	-3697.76396	-1088.4
**TS3-C-*SRR***	1.087728	-3696.789153	-3695.635960	-3695.799135	-3697.761473	-1135.13
**TS3-C-*RSS***	1.088584	-3696.792104	-3695.638051	-3695.800975	-3697.765683	-992.4
**TS3-C-*RSR***	1.087216	-3696.779216	-3695.625766	-3695.794034	-3697.756204	-1116.0
**INT4-C-*SRS***	1.092262	-3696.820563	-3695.66111	-3695.832239	-3697.793441	
**Pro-*SRS***	0.538636	-1628.925385	-1628.35187	-1628.4539	-1629.399975	
**(*R*)-C1**	0.551987	-2067.866694	-2067.282573	-2067.376116	-2068.378075	
**INT0-B**	0.968736	-3124.091477	-3123.065468	-3123.215743	-3124.905838	
**TS1-B**	0.963865	-3124.058485	-3123.037873	-3123.18594	-3124.875196	-943.7
**INT1-B**	0.967561	-3124.061248	-3123.03641	-3123.18549	-3124.880215	
**INT2-B**	1.236296	-3893.292268	-3891.98165	-3892.165543	-3894.322055	
**1a'**	0.266043	-769.175757	-768.892233	-768.957217	-769.400061	
**TS2-B-*SR***	1.235676	-3893.275084	-3891.965483	-3892.151416	-3894.307855	-169.43
**TS2-B-*RS***	1.236960	-3893.272943	-3891.962659	-3892.147163	-3894.306984	-281.8
**TS2-B-*RR***	1.233970	-3893.264868	-3891.956879	-3892.144335	-3894.299013	-123.8
**TS2-B-*SS***	1.234070	-3893.259193	-3891.951827	-3892.135571	-3894.290436	-257.9
**INT3-B-*SR***	1.236688	-3893.286929	-3891.976757	-3892.16175	-3894.319212	
**INT3-B-*RS***	1.237321	-3893.295963	-3891.985086	-3892.169738	-3894.328224	
**TS3-B-*SRS***	1.232971	-3893.271667	-3891.965958	-3892.146600	-3894.303838	-1082.1
**TS3-B-*SRR***	1.233276	-3893.280093	-3891.974403	-3892.153367	-3894.308243	-1124.8
**TS3-B-*RSS***	1.233447	-3893.276405	-3891.970769	-3892.147112	-3894.302283	-940.5
**TS3-B-*RSR***	1.233066	-3893.263057	-3891.95732	-3892.138667	-3894.290341	-1117.7
**INT4-B-*SRS***	1.238632	-3893.309706	-3891.997889	-3892.179599	-3894.336276	
**Pro-*SRR***	0.538851	-1628.918845	-1628.344887	-1628.449496	-1629.394084	

**(*S*)-B2**	0.696801	-2264.351919	-2263.615994	-2263.726032	-2264.918042	
**INT0'-C**	0.824137	-2927.600876	-2926.726461	-2926.861545	-2928.363335	
**TS1'-C**	0.816848	-2927.564563	-2926.69829	-2926.830413	-2928.327947	-1183.9
**INT1'-C**	0.823178	-2927.58376	-2926.710056	-2926.845514	-2928.34991	
**INT2'-C**	1.090864	-3696.811269	-3695.652921	-3695.824118	-3697.788045	
**TS2'-C-*SR***	1.088797	-3696.773212	-3695.618961	-3695.783785	-3697.754381	-290.2
**TS2'-C-*RS***	1.091677	-3696.764795	-3695.606584	-3695.775078	-3697.744978	-168.4
**TS2'-C-*RR***	1.088312	-3696.776746	-3695.621902	-3695.790025	-3697.755492	-291.8
**TS2'-C-*SS***	1.087047	-3696.76742	-3695.613789	-3695.781481	-3697.749506	-403.8
**INT0'-B**	0.968745	-3124.082542	-3123.056204	-3123.209118	-3124.901239	
**TS1'-B**	0.961046	-3124.045067	-3123.027658	-3123.175952	-3124.864641	-1193.0
**INT1'-B**	0.968720	-3124.070756	-3123.044901	-3123.193315	-3124.891154	
**INT2'-B**	1.236258	-3893.291137	-3891.980155	-3892.167365	-3894.323521	
**TS2'-B-*SR***	1.234276	-3893.258265	-3891.950749	-3892.133459	-3894.292875	-306.0
**TS2'-B-*RS***	1.235306	-3893.25121	-3891.942235	-3892.128797	-3894.286341	-151.9
**TS2'-B-*RR***	1.230771	-3893.266127	-3891.961515	-3892.147505	-3894.299043	-596.4
**TS2'-B-*SS***	1.232149	-3893.259819	-3891.953935	-3892.139663	-3894.296068	-193.3
**INT0-DMP**	0.379822	-1582.283201	-1581.875081	-1581.965287	-1582.703353	
**INT0'-DMP**	0.380136	-1582.280666	-1581.872481	-1581.960427	-1582.698561	
**TS1-DMP**	0.375008	-1582.248271	-1581.845526	-1581.934545	-1582.669681	-864.6
**TS1'-DMP**	0.373266	-1582.242313	-1581.842012	-1581.926747	-1582.661857	-1233.0
**TS2-DMP–*syn***	0.646975	-2351.461494	-2350.770144	-2350.893914	-2352.098429	-247.5
**TS2-DMP-*anti***	0.644640	-2351.450149	-2350.760567	-2350.887220	-2352.089384	-183.7
**TS2'-DMP-*syn***	0.643889	-2351.448208	-2350.760088	-2350.882759	-2352.087371	-494.2
**TS2'-DMP-*anti***	0.644568	-2351.453477	-2350.764799	-2350.886120	-2352.091450	-383.8
**TS3-C-*SRS*-Cy**	1.160028	-3700.387184	-3699.160153	-3699.328509	-3701.368523	-1086.37
**TS3-C-*SRR*-Cy**	1.160177	-3700.396111	-3699.169348	-3699.333853	-3701.369247	-1159.78
**TS3-B-*SRS*-Cy**	1.303682	-3896.879929	-3895.502301	-3895.685681	-3897.913979	-969.48
**TS3-B-*SRR*-Cy**	1.305190	-3896.885902	-3895.507205	-3895.689346	-3897.91811	-1103.05
**TS3-B-*SRS*-nPr**	1.289189	-3971.854218	-3970.489855	-3970.677150	-3972.909461	-1132.90
**TS3-B-*SRR*-nPr**	1.291208	-3971.867901	-3970.501376	-3970.687044	-3972.918019	-1092.16

X-Ray Structures

X-Ray structure of (±)-**3m** (with CCDC number 1971171)

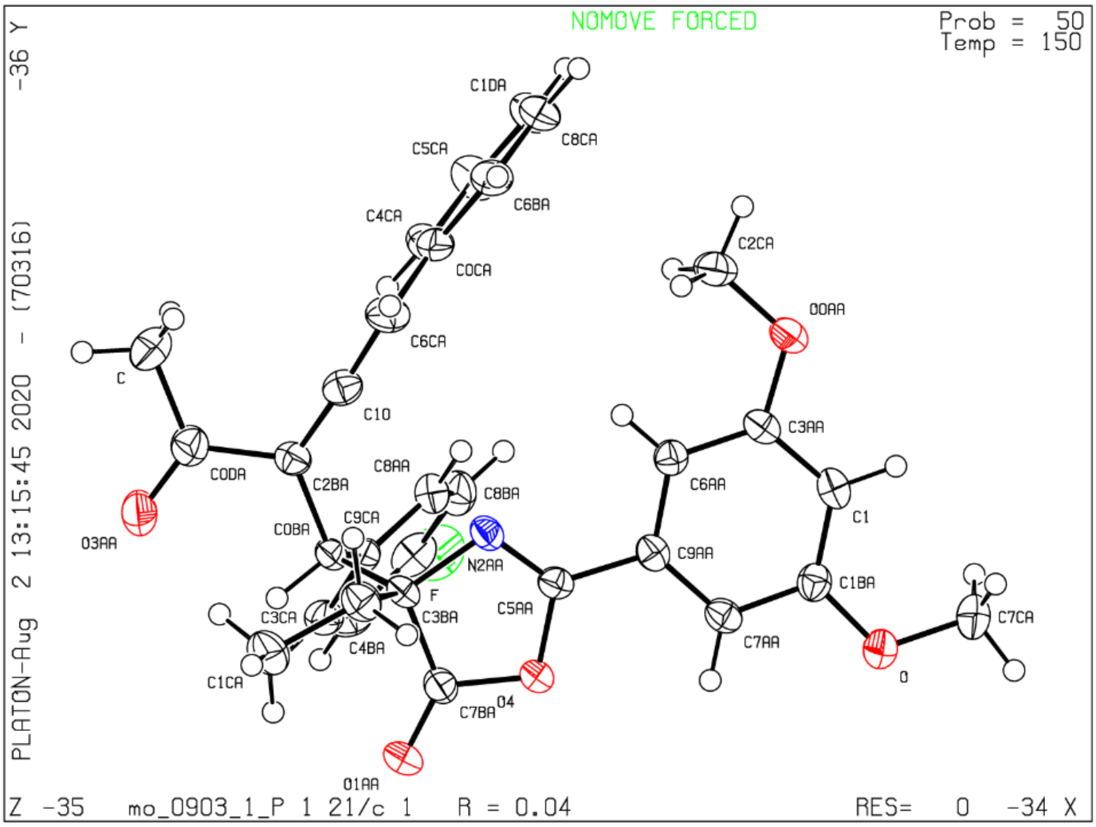

Supplementary Table 7. Crystal data and structure refinement for (±)-3m.

Identification code	
Empirical formula	$C_{31}H_{28}FNO_5$
Formula weight	513.54
Temperature/K	149.99
Crystal system	monoclinic
Space group	$P2_1/c$
a/Å	8.9281(5)
b/Å	8.6663(4)
c/Å	33.9189(18)
α/°	90.00
β/°	91.605(2)
γ/°	90.00
Volume/Å^3	2623.4(2)
Z	4
ρ_{calc}g/cm^3	1.300
μ/mm^{-1}	0.093
F(000)	1080.0
Crystal size/mm^3	0.2 × 0.1 × 0.1
Radiation	MoKα (λ = 0.71073)
2Θ range for data collection/°	4.56 to 52.04
Index ranges	-11 ≤ h ≤ 11, -10 ≤ k ≤ 10, -41 ≤ l ≤ 41
Reflections collected	33133
Independent reflections	5154 [R_{int} = 0.0462, R_{sigma} = 0.0326]
Data/restraints/parameters	5154/0/347
Goodness-of-fit on F^2	1.048
Final R indexes [I>=2σ (I)]	R_1 = 0.0449, wR_2 = 0.1001
Final R indexes [all data]	R_1 = 0.0704, wR_2 = 0.1128
Largest diff. peak/hole / e Å^{-3}	0.23/-0.23

X-Ray structure of **4c** (with CCDC number 1971946)

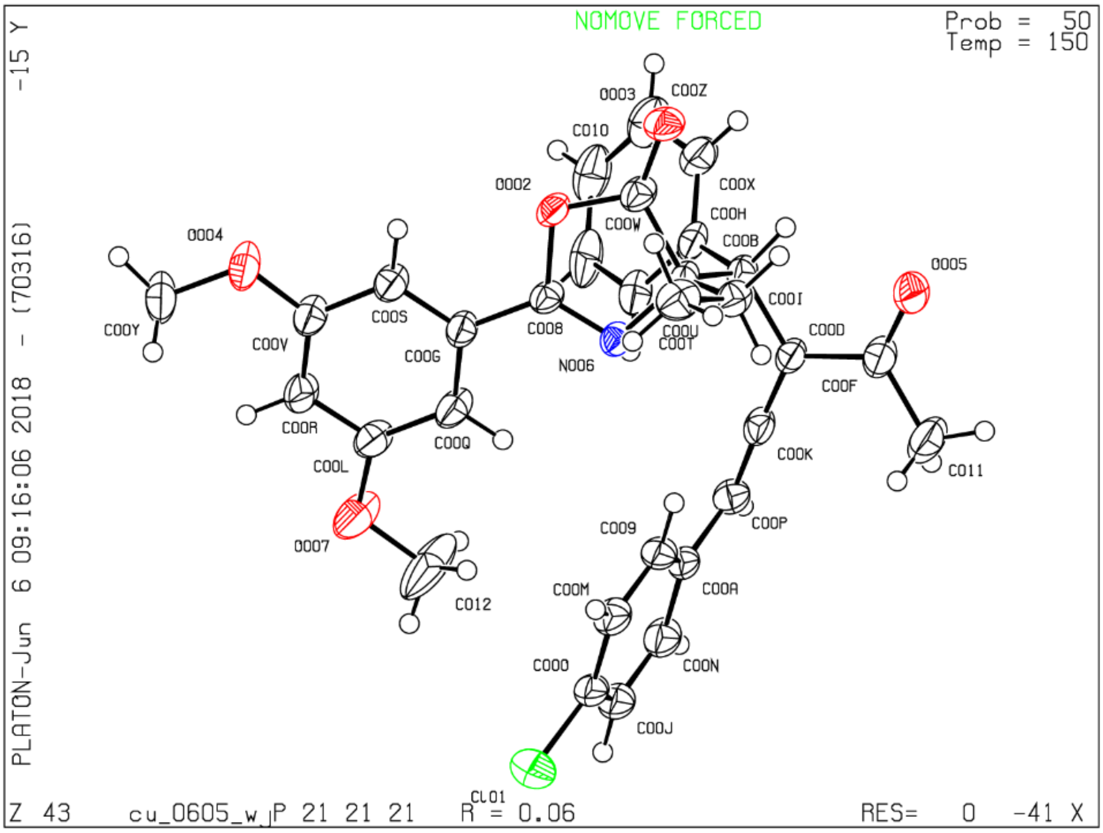

Supplementary Table 8. Crystal data and structure refinement for 4c.

Identification code	
Empirical formula	$C_{31}H_{28}ClNO_5$
Formula weight	529.99
Temperature/K	150.03
Crystal system	orthorhombic
Space group	$P2_12_12_1$
a/Å	16.4628(6)
b/Å	19.0905(6)
c/Å	8.8358(3)
α/°	90.00
β/°	90.00
γ/°	90.00
Volume/$Å^3$	2776.94(16)
Z	4
$\rho_{calc}g/cm^3$	1.268
μ/mm^{-1}	1.547
F(000)	1112.0
Crystal size/mm^3	0.01 × 0.01 × 0.01
Radiation	CuKα (λ = 1.54178)
2Θ range for data collection/°	7.1 to 148.98
Index ranges	$-20 \leqslant h \leqslant 20, -23 \leqslant k \leqslant 23, -10 \leqslant l \leqslant 10$
Reflections collected	26413
Independent reflections	5430 [R_{int} = 0.0999, R_{sigma} = 0.0596]
Data/restraints/parameters	5430/0/357
Goodness-of-fit on F^2	1.135
Final R indexes [I>=2σ (I)]	R_1 = 0.0551, wR_2 = 0.1079
Final R indexes [all data]	R_1 = 0.0761, wR_2 = 0.1175
Largest diff. peak/hole / e $Å^{-3}$	0.23/-0.29
Flack parameter	0.07(2)

X-Ray structure of **11a** (with CCDC number 1971947)

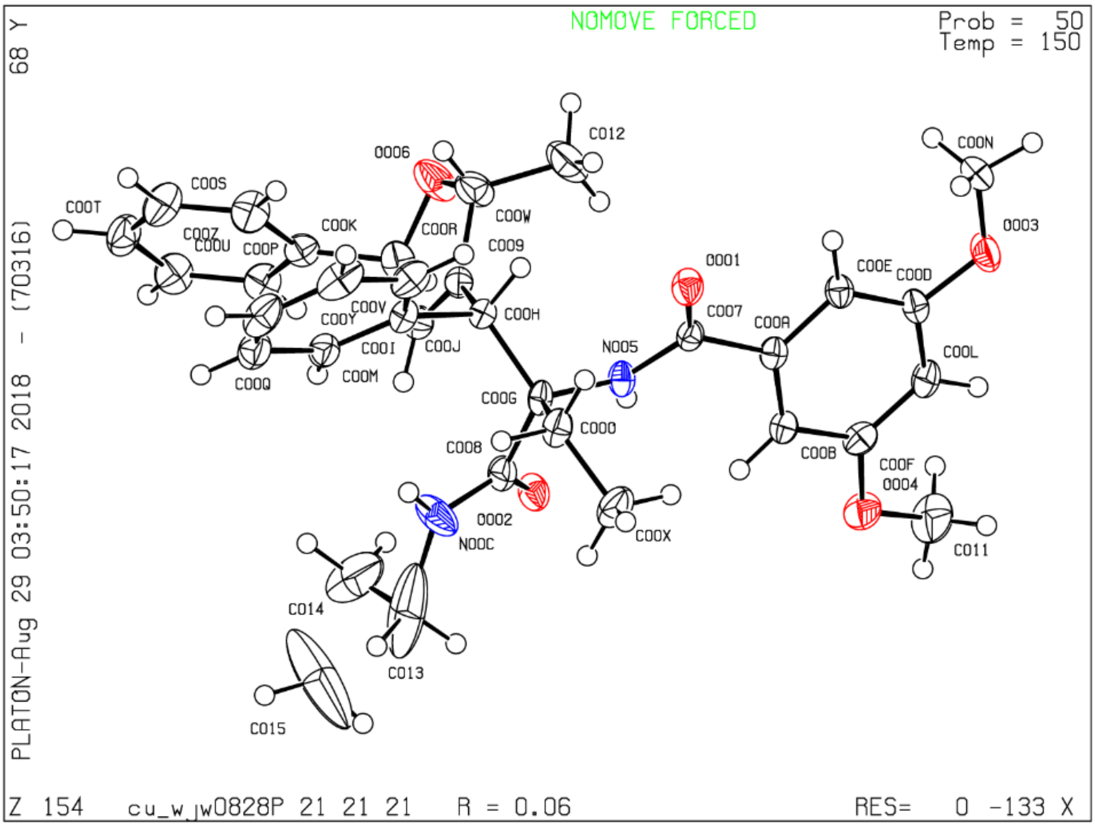

Supplementary Table 9. Crystal data and structure refinement for 11a.

Identification code	
Empirical formula	$C_{34}H_{40}N_2O_5$
Formula weight	556.68
Temperature/K	150.0
Crystal system	orthorhombic
Space group	$P2_12_12_1$
a/Å	10.4433(3)
b/Å	11.8284(3)
c/Å	24.0925(7)
α/°	90
β/°	90
γ/°	90
Volume/$Å^3$	2976.09(14)
Z	4
$\rho_{calc}g/cm^3$	1.242
μ/mm^{-1}	0.665
F(000)	1192.0
Crystal size/mm^3	0.35 × 0.1 × 0.1
Radiation	CuKα (λ = 1.54178)
2Θ range for data collection/ °	7.338 to 133.166
Index ranges	$-12 \leqslant h \leqslant 12, -14 \leqslant k \leqslant 13, -28 \leqslant l \leqslant 28$
Reflections collected	34835
Independent reflections	5248 [R_{int} = 0.0496, R_{sigma} = 0.0319]
Data/restraints/parameters	5248/38/386
Goodness-of-fit on F^2	1.032
Final R indexes [I>=2σ (I)]	R_1 = 0.0469, wR_2 = 0.1184
Final R indexes [all data]	R_1 = 0.0495, wR_2 = 0.1205
Largest diff. peak/hole / e $Å^{-3}$	0.79/-0.63
Flack parameter	0.08(7)

X-Ray structure of **12a** (with CCDC number 1971365)

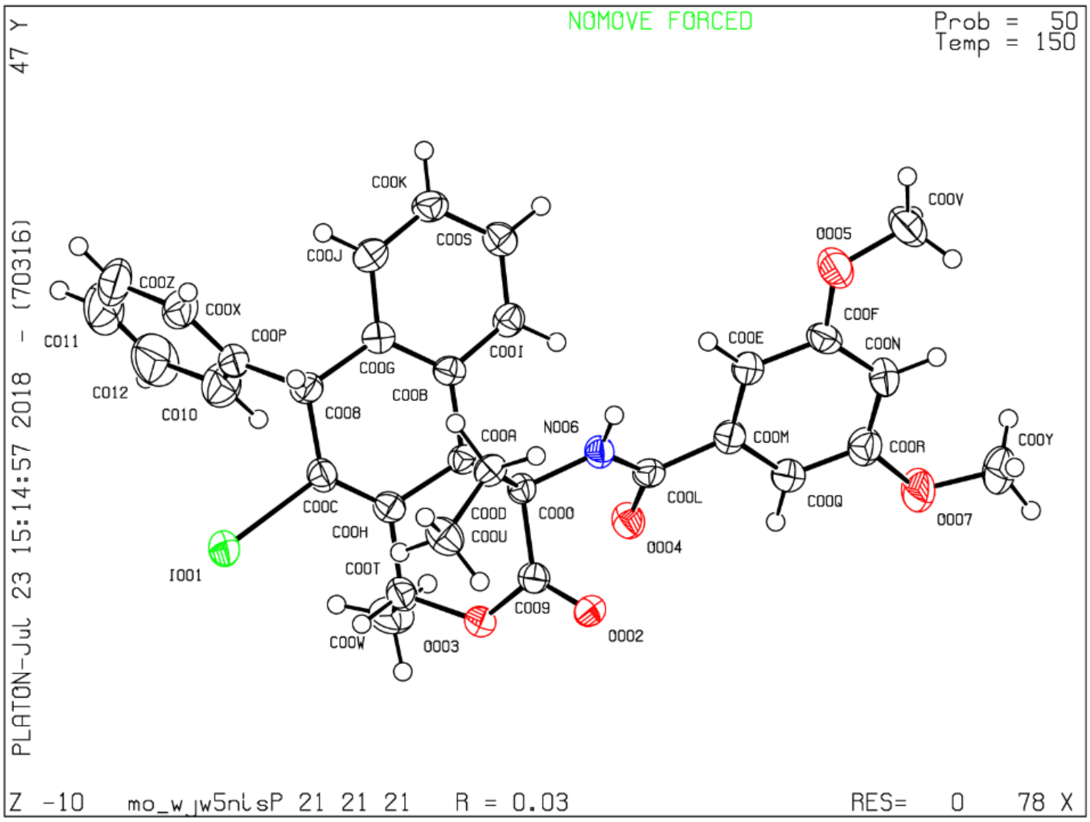

Supplementary Table 10. Crystal data and structure refinement for 12a.

Identification code	
Empirical formula	$C_{31}H_{30}INO_5$
Formula weight	623.46
Temperature/K	149.96
Crystal system	orthorhombic
Space group	$P2_12_12_1$
a/Å	17.1360(10)
b/Å	16.5257(9)
c/Å	9.6866(6)
α/°	90
β/°	90
γ/°	90
Volume/$Å^3$	2743.1(3)
Z	4
$\rho_{calc}g/cm^3$	1.510
μ/mm^{-1}	1.208
F(000)	1264.0
Crystal size/mm^3	0.4 × 0.2 × 0.2
Radiation	MoKα (λ = 0.71073)
2Θ range for data collection/°	4.754 to 52.864
Index ranges	$-21 \leqslant h \leqslant 21$, $-20 \leqslant k \leqslant 20$, $-12 \leqslant l \leqslant 12$
Reflections collected	34341
Independent reflections	5636 [R_{int} = 0.0553, R_{sigma} = 0.0364]
Data/restraints/parameters	5636/0/347
Goodness-of-fit on F^2	1.013
Final R indexes [I>=2σ (I)]	R_1 = 0.0317, wR_2 = 0.0621
Final R indexes [all data]	R_1 = 0.0432, wR_2 = 0.0668
Largest diff. peak/hole / e $Å^{-3}$	0.76/-0.41
Flack parameter	-0.037(11)

X-Ray structure of (±)-**16a** (with CCDC number 1971366)

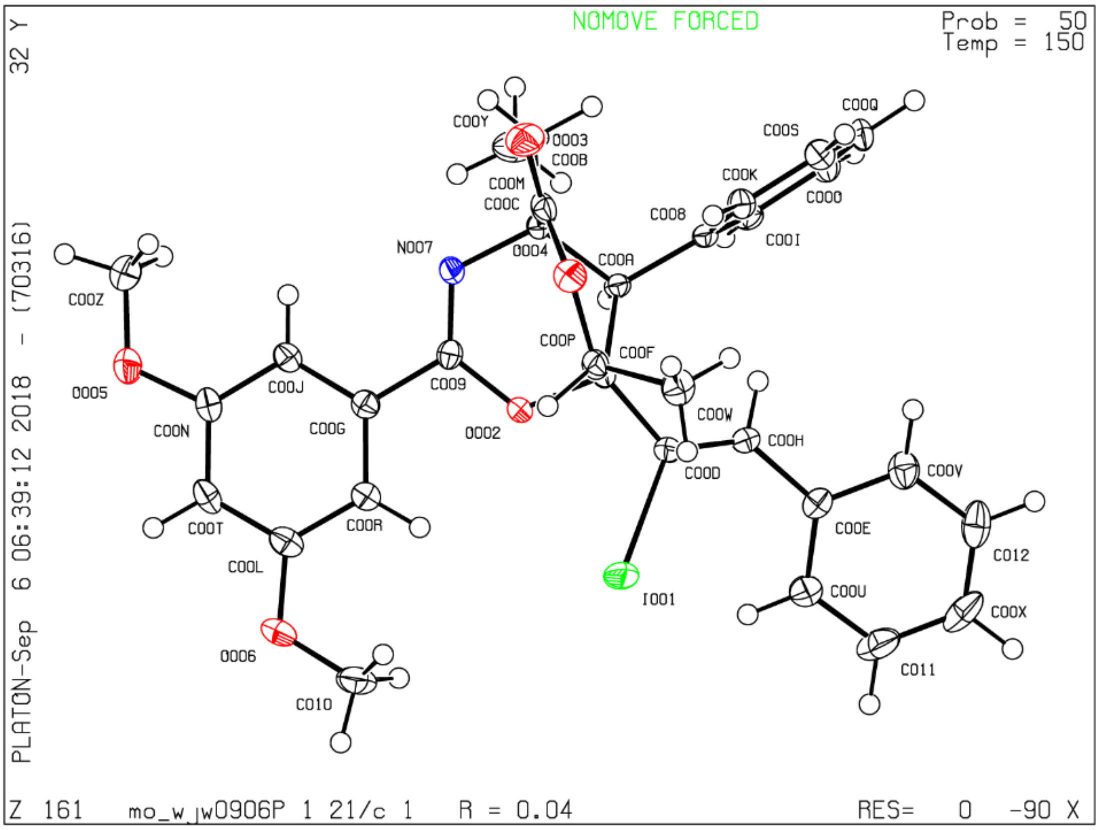

Supplementary Table 11. Crystal data and structure refinement for 16a.

Identification code	
Empirical formula	$C_{31}H_{30}INO_5$
Formula weight	623.46
Temperature/K	150.0
Crystal system	monoclinic
Space group	$P2_1/c$
a/Å	8.8780(5)
b/Å	14.3449(9)
c/Å	21.4134(12)
α/°	90.00
β/°	98.826(2)
γ/°	90.00
Volume/Å^3	2694.8(3)
Z	4
ρ_{calc}g/cm^3	1.537
μ/mm^{-1}	1.230
F(000)	1264.0
Crystal size/mm^3	0.1 × 0.1 × 0.1
Radiation	MoKα (λ = 0.71073)
2Θ range for data collection/°	4.64 to 52.88
Index ranges	-11 ≤ h ≤ 11, -17 ≤ k ≤ 17, -26 ≤ l ≤ 26
Reflections collected	69940
Independent reflections	5531 [R_{int} = 0.0865, R_{sigma} = 0.0320]
Data/restraints/parameters	5531/0/347
Goodness-of-fit on F^2	1.052
Final R indexes [I>=2σ (I)]	R_1 = 0.0350, wR_2 = 0.0769
Final R indexes [all data]	R_1 = 0.0569, wR_2 = 0.0864
Largest diff. peak/hole / e Å^{-3}	1.08/-0.84

HPLC traces:

(*S*)-4-((*1R*,*3R*)-2-acetyl-1,4-diphenylbuta-2,3-dien-1-yl)-2-(3,5-dimethoxyphenyl)-4-ethyloxazol-5(4H)-one **(3a)**

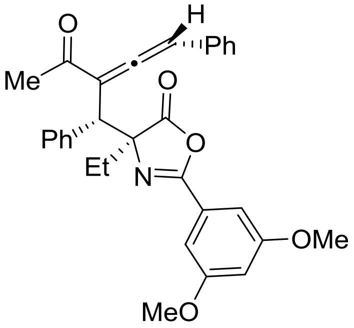

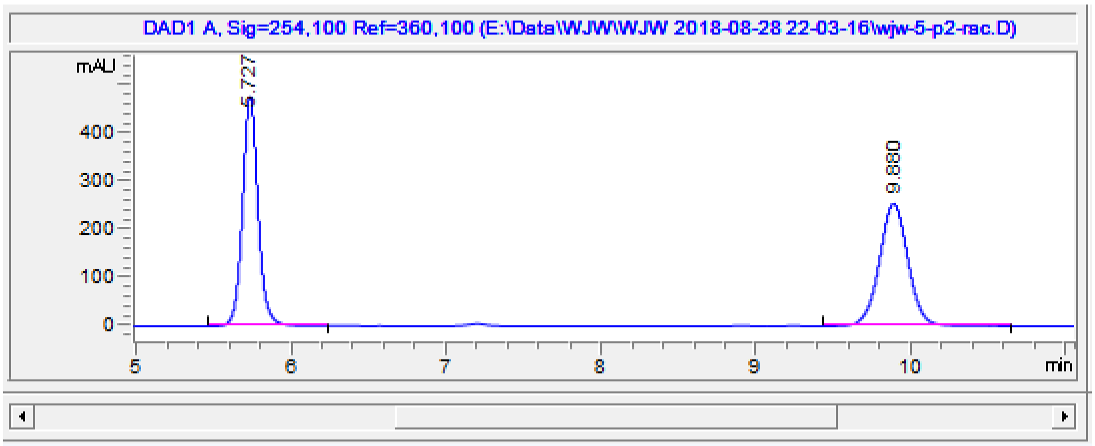

#	Time	Type	Area	Height	Width	Area%	Symmetry
1	5.727	BB	3306.4	476.6	0.1054	50.304	0.908
2	9.88	BB	3266.4	254.9	0.198	49.696	0.966

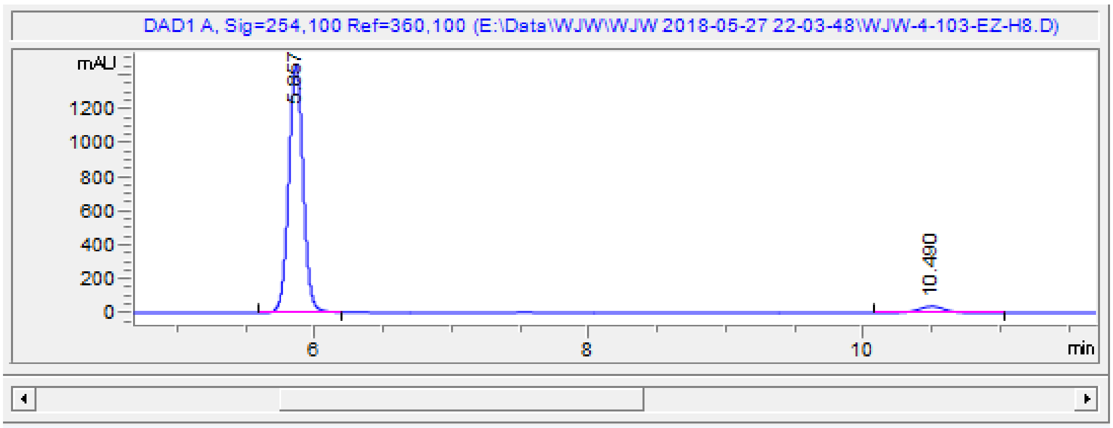

#	Time	Type	Area	Height	Width	Area%	Symmetry
1	5.857	BB	10560.2	1455.2	0.1122	95.428	0.954
2	10.49	BB	506	37.6	0.2012	4.572	0.991

(*S*)-4-((*1R*,*3R*)-2-acetyl-1-phenyl-4-(p-tolyl)buta-2,3-dien-1-yl)-2-(3,5-dimethoxyphenyl)-4-ethyloxazol-5(4H)-one **(3b)**

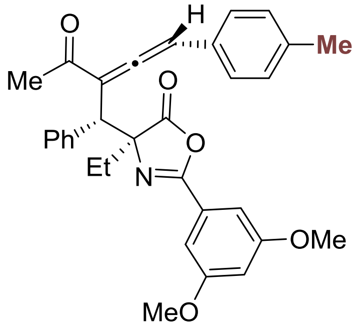

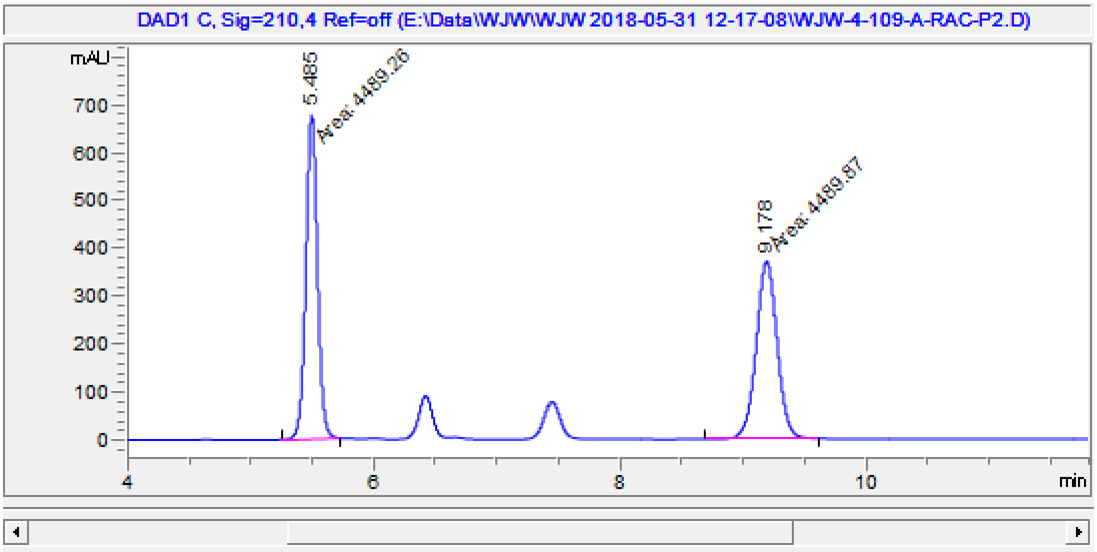

#	Time	Type	Area	Height	Width	Area%	Symmetry
1	5.485	MM	4489.3	676.6	0.1106	49.997	0.971
2	9.178	MM	4489.9	370.8	0.2018	50.003	0.984

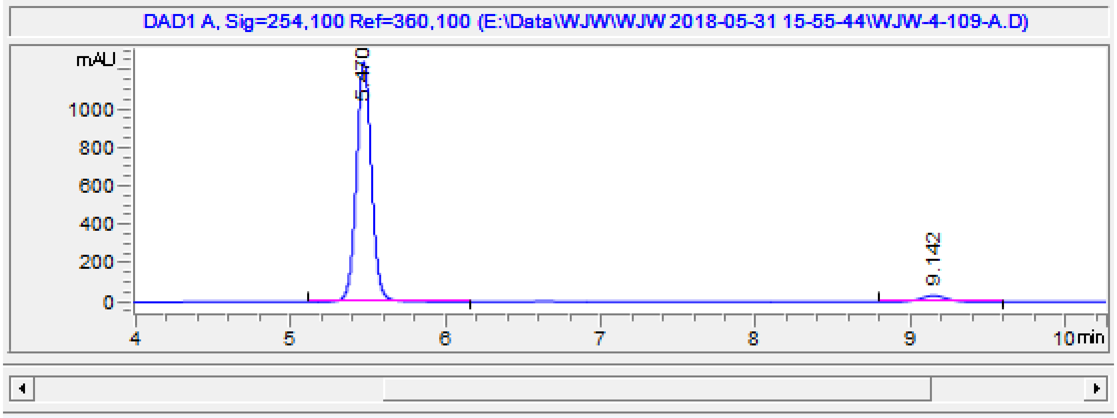

#	Time	Type	Area	Height	Width	Area%	Symmetry
1	5.47	BB	8428.1	1244.7	0.1045	95.700	0.941
2	9.142	BB	378.7	32	0.179	4.300	0.993

(*S*)-4-((*1R*,*3R*)-2-acetyl-4-(4-chlorophenyl)-1-phenylbuta-2,3-dien-1-yl)-2-(3,5-dimethoxyphenyl)-4-ethyloxazol-5(4H)-one **(3c)**

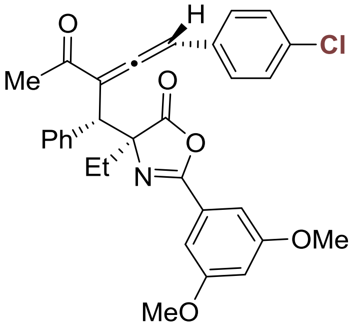

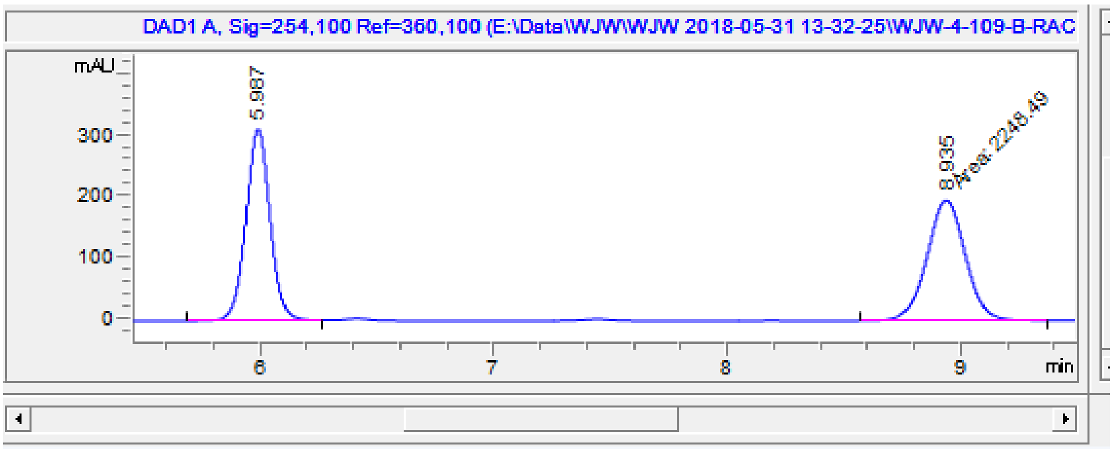

#	Time	Type	Area	Height	Width	Area%	Symmetry
1	5.987	BB	2236.9	310.6	0.11	49.870	0.969
2	8.935	FM	2248.5	195.2	0.1919	50.130	0.986

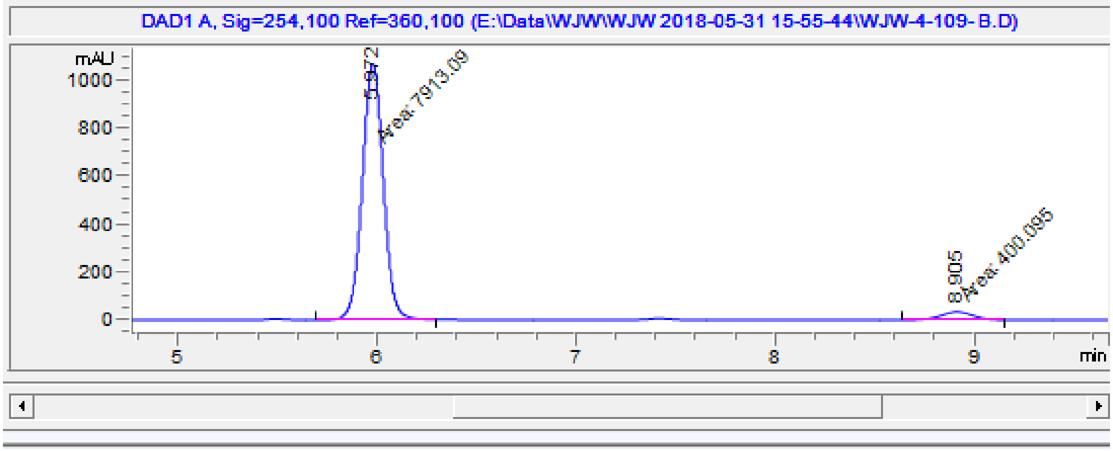

#	Time	Type	Area	Height	Width	Area%	Symmetry
1	5.972	MM	7913.1	1081.1	0.122	95.187	0.984
2	8.905	MM	400.1	35.3	0.1889	4.813	1.042

(*S*)-4-((*1R*,*3R*)-2-acetyl-4-(4-methoxyphenyl)-1-phenylbuta-2,3-dien-1-yl)-2-(3,5-dimethoxyphenyl)-4-ethyloxazol-5(4H)-one **(3d)**

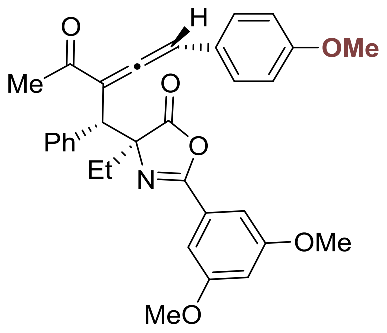

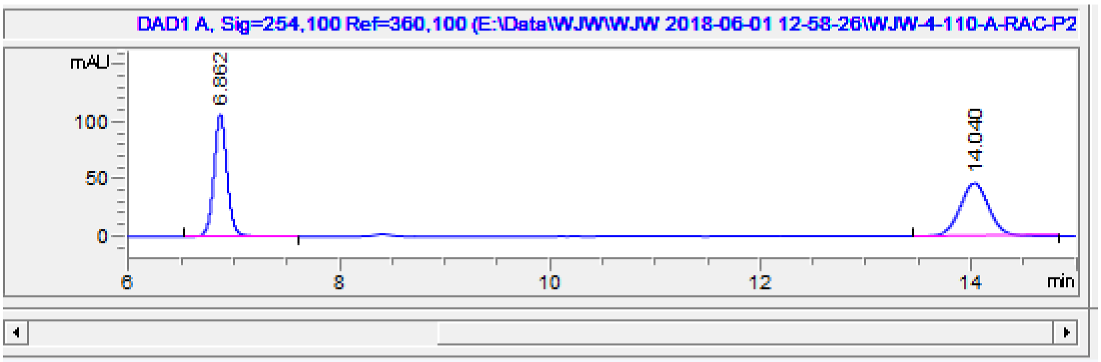

#	Time	Type	Area	Height	Width	Area%	Symmetry
1	6.862	BB	912.9	105.8	0.1322	50.926	0.929
2	14.04	BB	879.7	46	0.2907	49.074	0.958

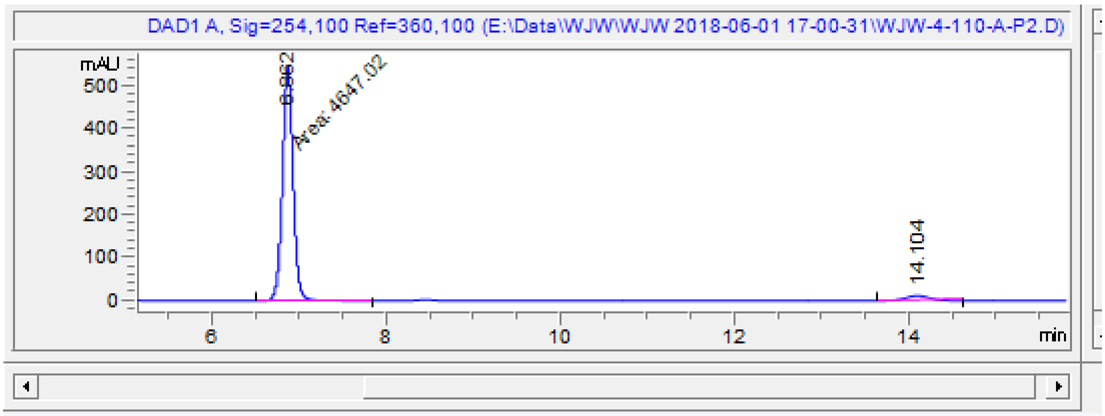

#	Time	Type	Area	Height	Width	Area%	Symmetry
1	6.862	MM	4647	548.4	0.1412	95.315	0.96
2	14.104	BB	228.4	12	0.2272	4.685	0.966

(*S*)-4-((*1R*,*3R*)-2-acetyl-4-(4-fluorophenyl)-1-phenylbuta-2,3-dien-1-yl)-2-(3,5-dimethoxyphenyl)-4-ethyloxazol-5(4H)-one **(3e)**

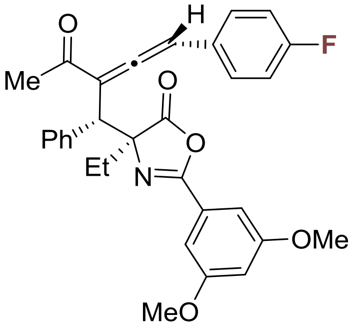

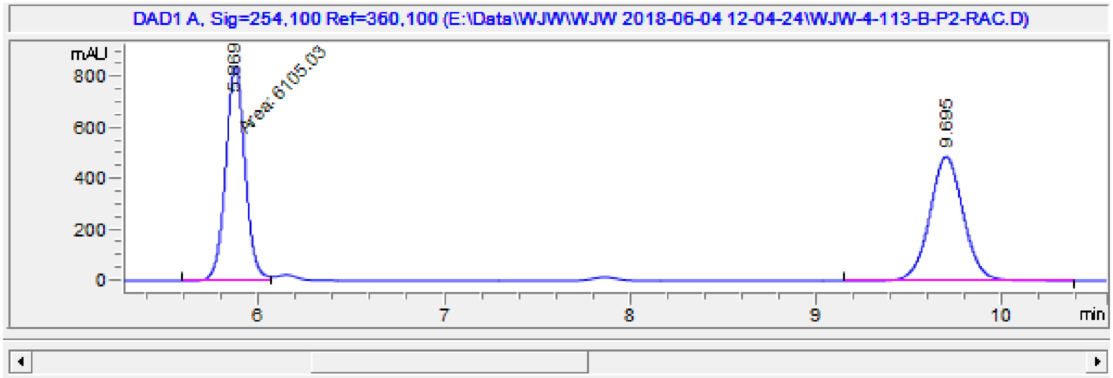

#	Time	Type	Area	Height	Width	Area%	Symmetry
1	5.869	MF	6105	838.6	0.1213	49.961	0
2	9.695	BB	6114.6	485.8	0.1934	50.039	0.93

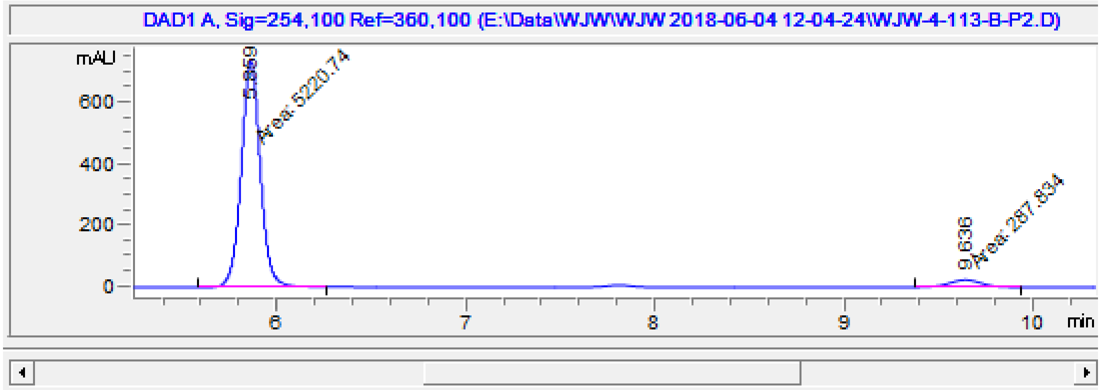

#	Time	Type	Area	Height	Width	Area%	Symmetry
1	5.859	MM	5220.7	742	0.1173	94.775	0.965
2	9.636	MM	287.8	23.9	0.2005	5.225	0.995

(*S*)-4-((*1R*,*3R*)-2-acetyl-1-phenyl-4-(4-(trifluoromethyl)phenyl)buta-2,3-dien-1-yl)-2-(3,5-dimethoxyphenyl)-4-ethyloxazol-5(4H)-one **(3f)**

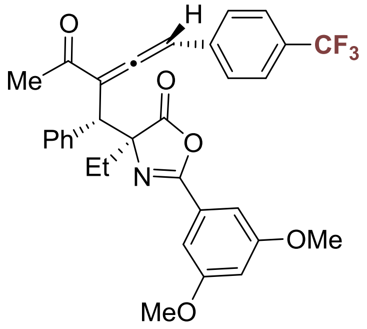

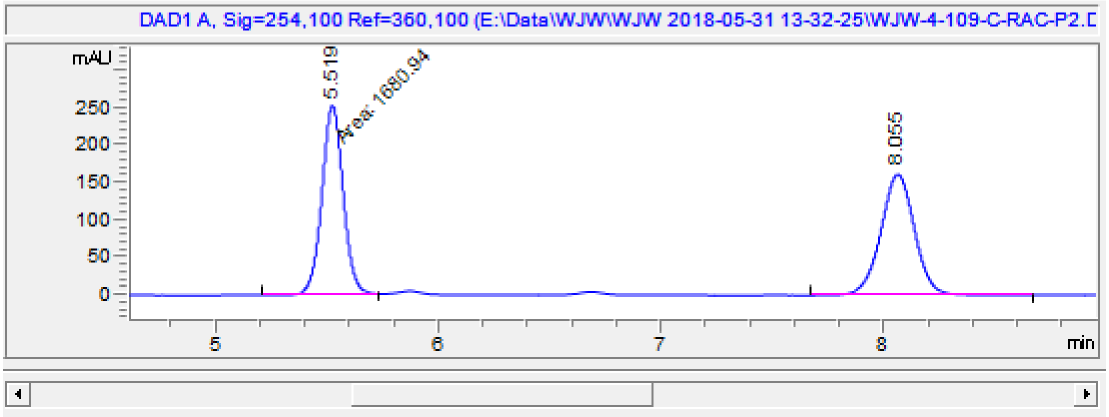

#	Time	Type	Area	Height	Width	Area%	Symmetry
1	5.519	MF	1680.9	251.5	0.1114	50.012	0.965
2	8.055	BB	1680.2	160	0.161	49.988	0.995

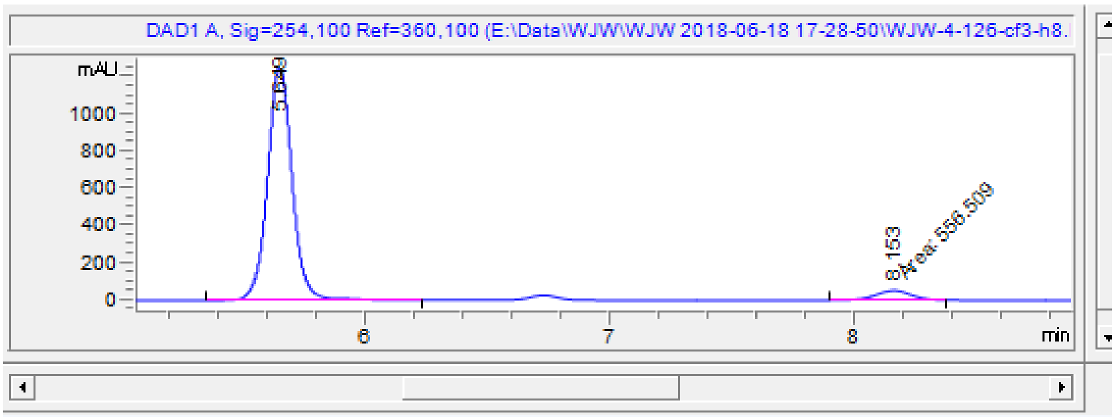

#	Time	Type	Area	Height	Width	Area%	Symmetry
1	5.649	BB	8653.7	1235.1	0.1077	93.958	0.936
2	8.153	MM	556.5	52.4	0.1769	6.042	1.029

Methyl-3-((*R*)-3-((*R*)-((*S*)-2-(3,5-dimethoxyphenyl)-4-ethyl-5-oxo-4,5-dihydrooxazol-4-yl)(phenyl)methyl)-4-oxopenta-1,2-dien-1-yl)benzoate **(3g)**

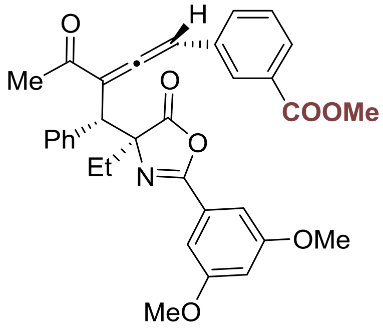

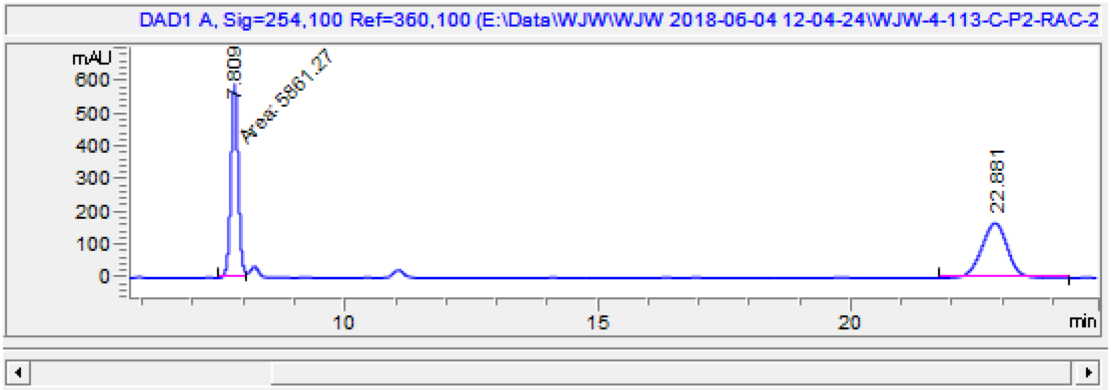

#	Time	Type	Area	Height	Width	Area%	Symmetry
1	7.809	MM	5861.3	588.2	0.1661	49.925	0.958
2	22.881	BB	5878.8	165.9	0.5135	50.075	1.074

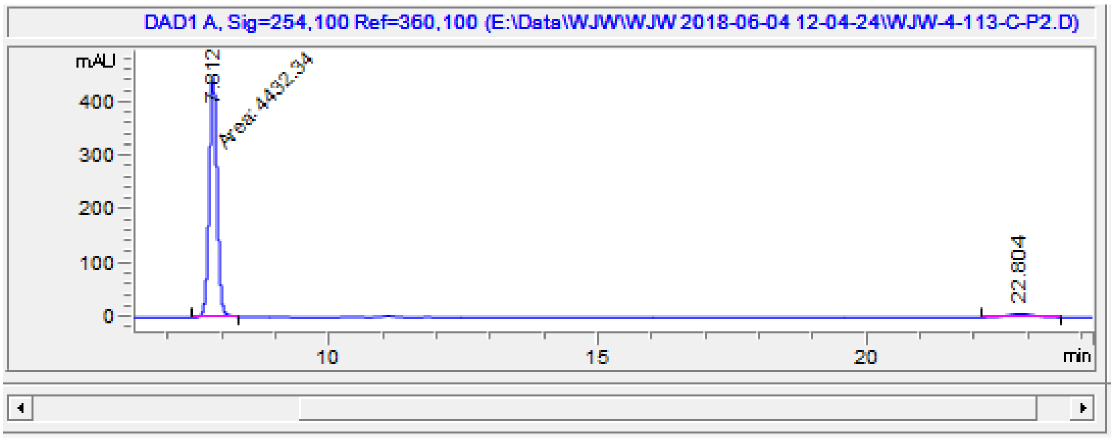

#	Time	Type	Area	Height	Width	Area%	Symmetry
1	7.812	MM	4432.3	444.8	0.1661	95.166	0.965
2	22.804	BB	225.1	6.7	0.3961	4.834	1.001

(*S*)-4-((*1R*,*3R*)-2-acetyl-4-(2-chlorophenyl)-1-phenylbuta-2,3-dien-1-yl)-2-(3,5-dimethoxyphenyl)-4-ethyloxazol-5(4H)-one **(3h)**

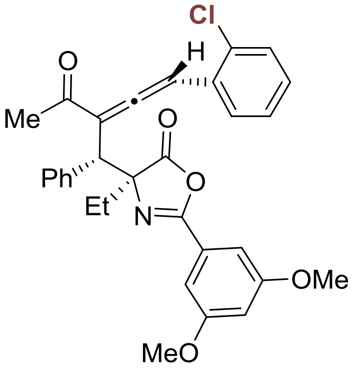

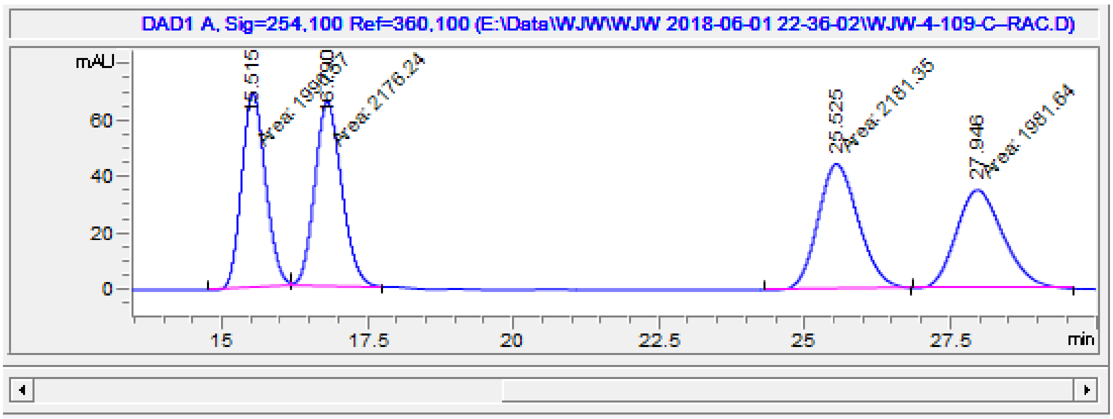

#	Time	Type	Area	Height	Width	Area%	Symmetry
1	15.515	MM	1990.6	69.8	0.4755	23.897	0.843
2	16.79	MM	2176.2	66.5	0.5451	26.126	0.829
3	25.525	MM	2181.4	44.9	0.8097	26.187	0.819
4	27.946	MM	1981.6	35.1	0.9402	23.790	0.804

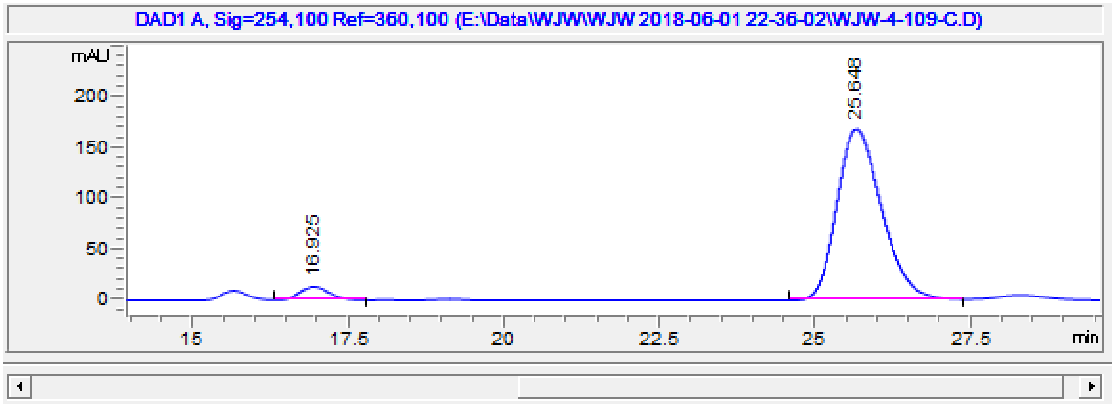

#	Time	Type	Area	Height	Width	Area%	Symmetry
1	16.925	BB	424.5	13.1	0.3837	4.799	0.875
2	25.648	BB	8420.7	169.1	0.6555	95.201	0.725

(*S*)-4-((*1R*,*3R*)-2-acetyl-1-phenyl-4-(thiophen-3-yl)buta-2,3-dien-1-yl)-2-(3,5-dimethoxyphenyl)-4-ethyloxazol-5(4H)-one **(3i)**

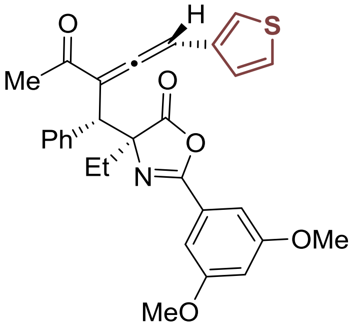

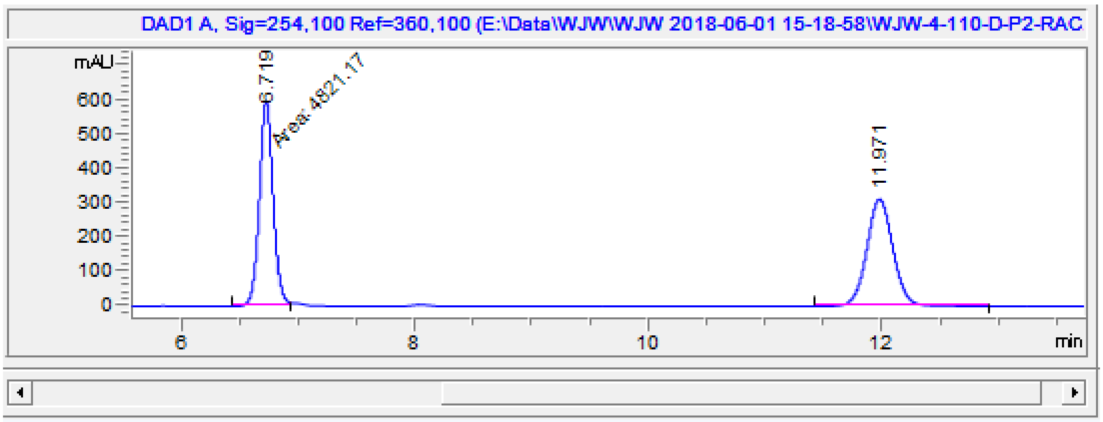

#	Time	Type	Area	Height	Width	Area%	Symmetry
1	6.719	MF	4821.2	593.1	0.1355	50.033	0.952
2	11.971	BB	4814.7	310.4	0.2365	49.967	0.92

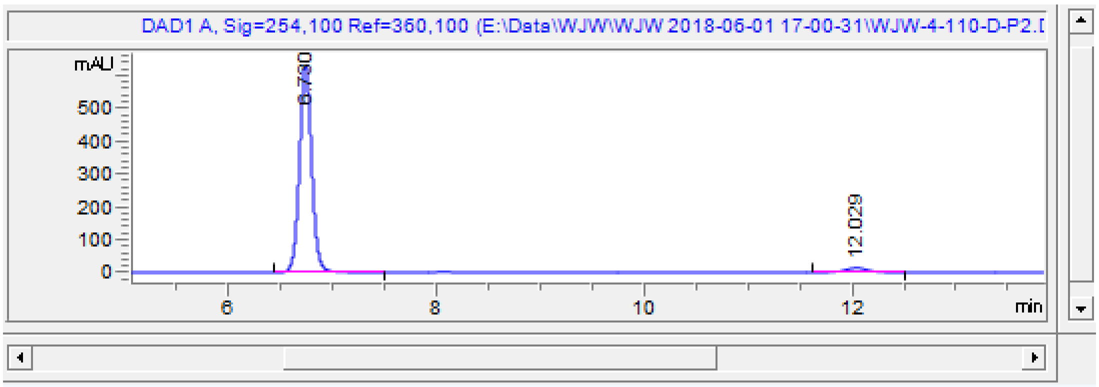

#	Time	Type	Area	Height	Width	Area%	Symmetry
1	6.73	BB	5179	634	0.1248	96.078	0.937
2	12.029	BB	211.4	13.7	0.2032	3.922	1.012

(*S*)-4-((*1R*,*3R*)-2-acetyl-4-(cyclohex-1-en-1-yl)-1-phenylbuta-2,3-dien-1-yl)-2-(3,5-dimethoxyphenyl)-4-ethyloxazol-5(4H)-one **(3j)**

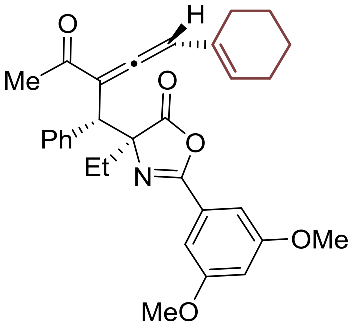

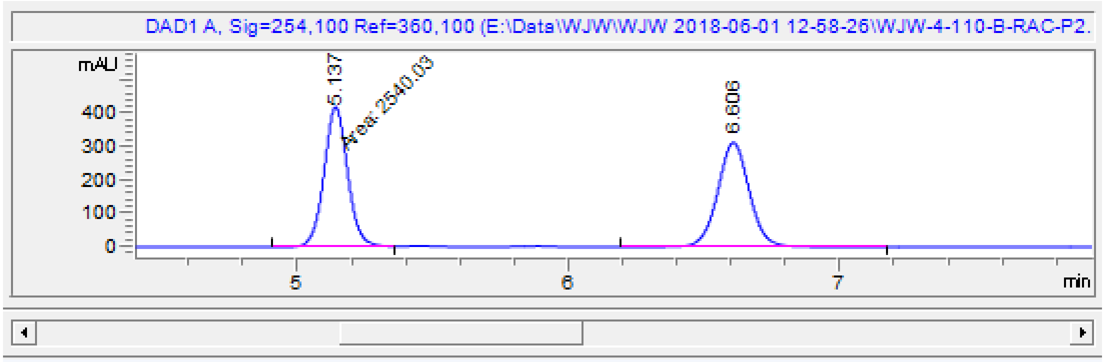

#	Time	Type	Area	Height	Width	Area%	Symmetry
1	5.137	MF	2540	416.7	0.1016	49.977	0.941
2	6.606	BB	2542.4	310.7	0.1255	50.023	0.96

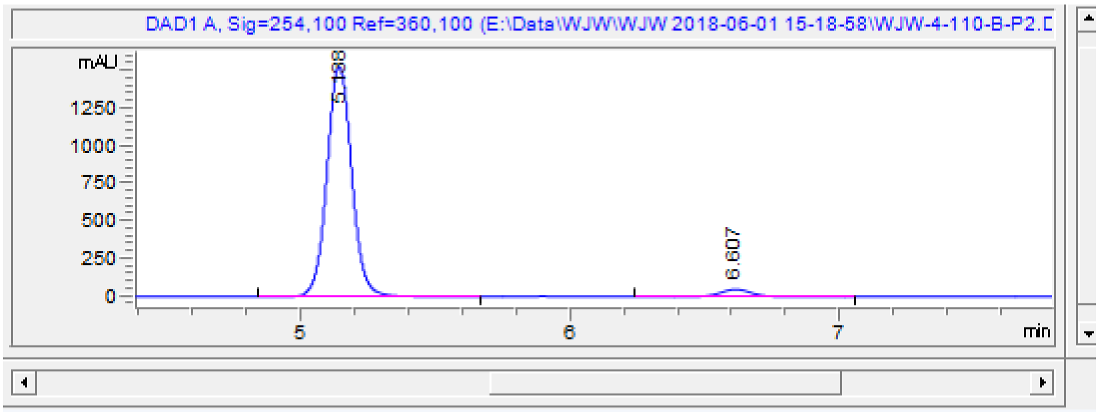

#	Time	Type	Area	Height	Width	Area%	Symmetry
1	5.138	BB	9986.7	1550.4	0.0997	96.004	0.919
2	6.607	BB	415.7	50.5	0.1266	3.996	0.968

(*S*)-4-((*1R*,*3R*)-2-acetyl-1-phenylocta-2,3-dien-1-yl)-2-(3,5-dimethoxyphenyl)-4-ethyloxazol-5(4H)-one **(3k)**

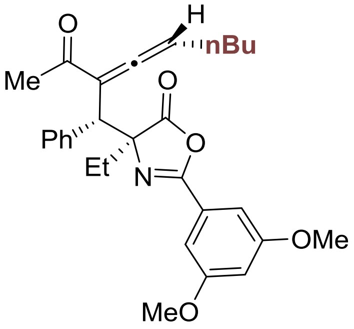

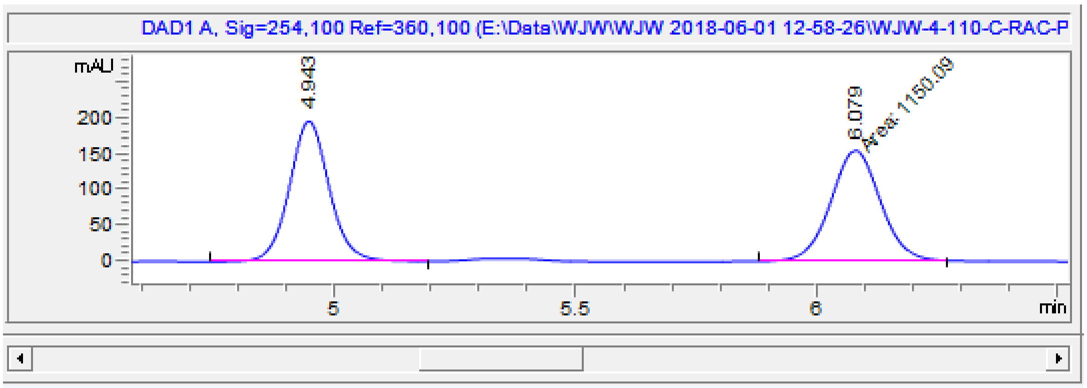

#	Time	Type	Area	Height	Width	Area%	Symmetry
1	4.943	BB	1148.1	198.1	0.0886	49.957	0.927
2	6.079	MF	1150.1	157.7	0.1216	50.043	0.966

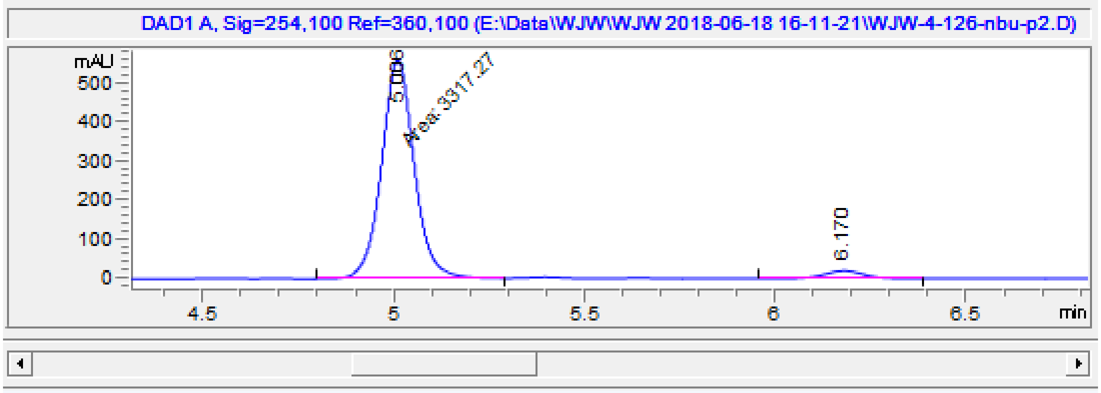

#	Time	Type	Area	Height	Width	Area%	Symmetry
1	5.006	MM	3317.3	555.7	0.0995	95.906	0.933
2	6.17	BB	141.6	19.3	0.1132	4.094	0.995

(*S*)-4-((*1R*,*3R*)-2-acetyl-4-phenyl-1-(p-tolyl)buta-2,3-dien-1-yl)-2-(3,5-dimethoxyphenyl)-4-ethyloxazol-5(4H)-one **(3l)**

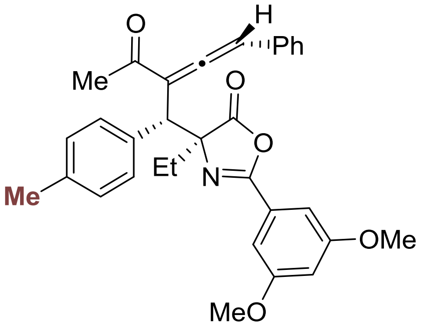

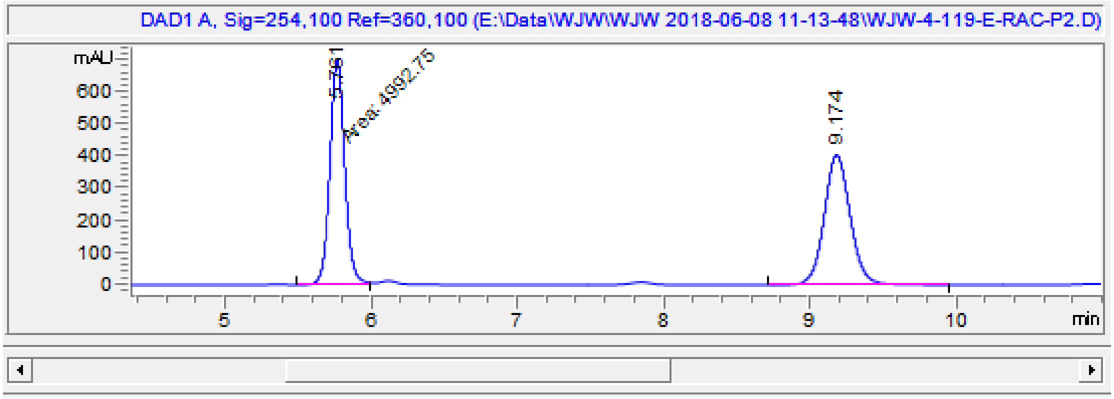

#	Time	Type	Area	Height	Width	Area%	Symmetry
1	5.761	FM	4992.8	705.3	0.118	49.968	0.916
2	9.174	BB	4999.2	409.2	0.187	50.032	0.883

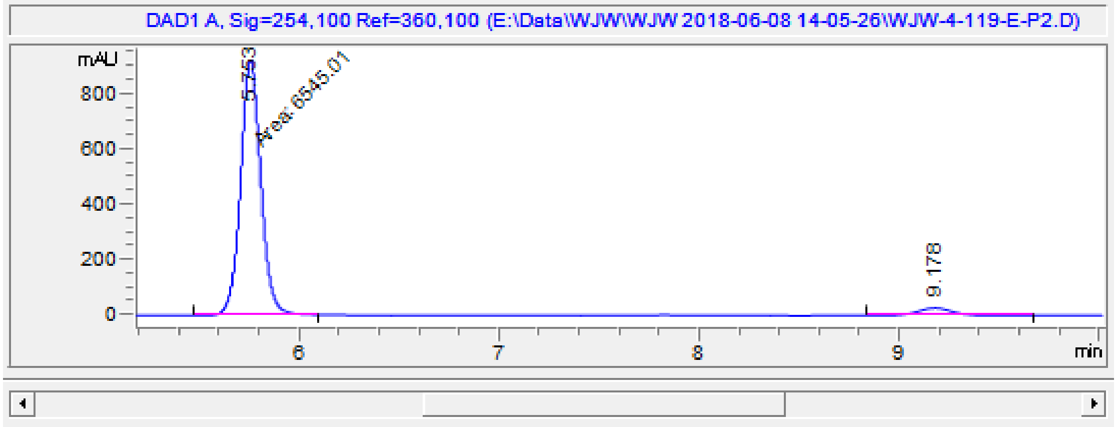

#	Time	Type	Area	Height	Width	Area%	Symmetry
1	5.753	MF	6545	918.4	0.1188	95.071	0.927
2	9.178	BB	339.3	27.8	0.1814	4.929	0.961

(*S*)-4-((*1R*,*3R*)-2-acetyl-1-(4-fluorophenyl)-4-phenylbuta-2,3-dien-1-yl)-2-(3,5-dimethoxyphenyl)-4-ethyloxazol-5(4H)-one **(3m)**

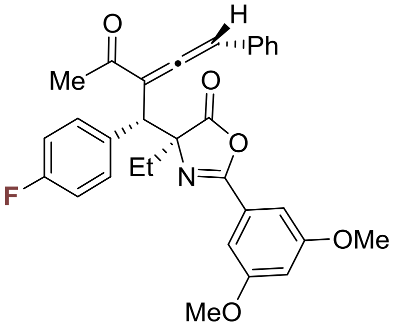

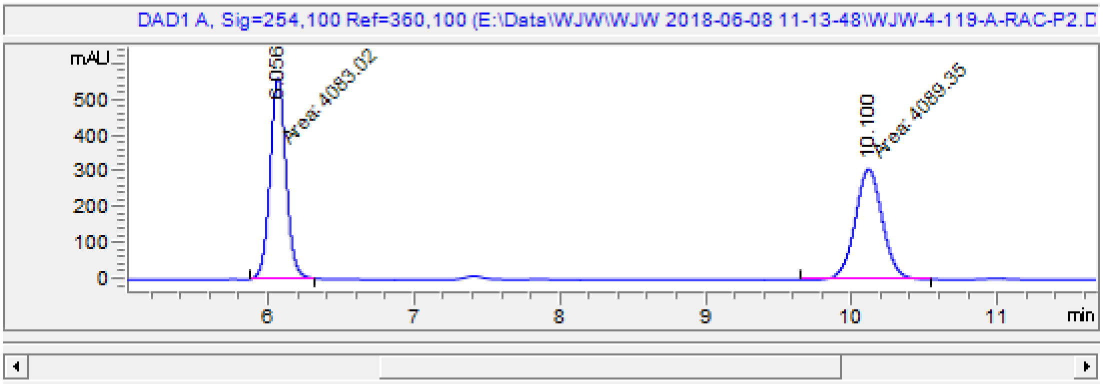

#	Time	Type	Area	Height	Width	Area%	Symmetry
1	6.056	MM	4083	555.9	0.1224	49.961	0.933
2	10.1	MM	4089.3	308.4	0.221	50.039	0.94

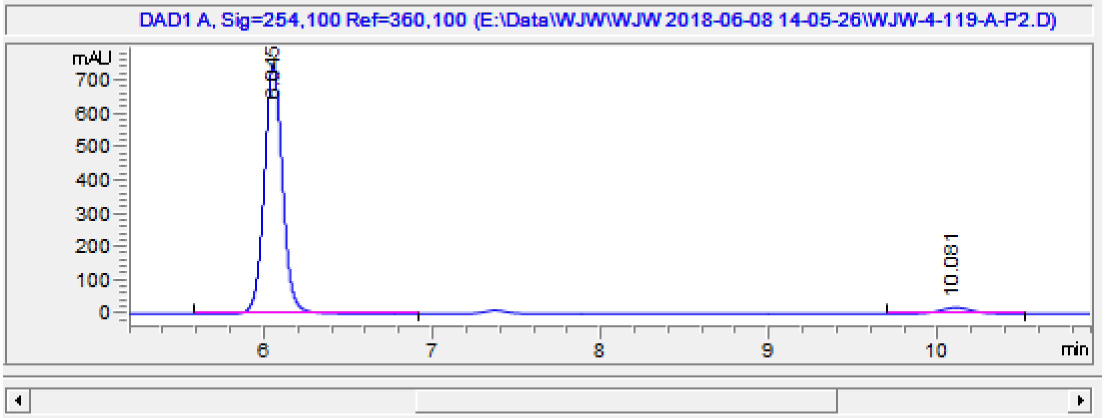

#	Time	Type	Area	Height	Width	Area%	Symmetry
1	6.045	BB	5609.6	759.8	0.1131	95.812	0.934
2	10.081	BB	245.2	18.8	0.1932	4.188	0.982

(*S*)-4-((*1R*,*3R*)-2-acetyl-1-(4-chlorophenyl)-4-phenylbuta-2,3-dien-1-yl)-2-(3,5-dimethoxyphenyl)-4-ethyloxazol-5(4H)-one **(3n)**

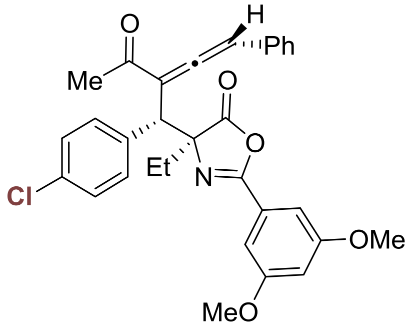

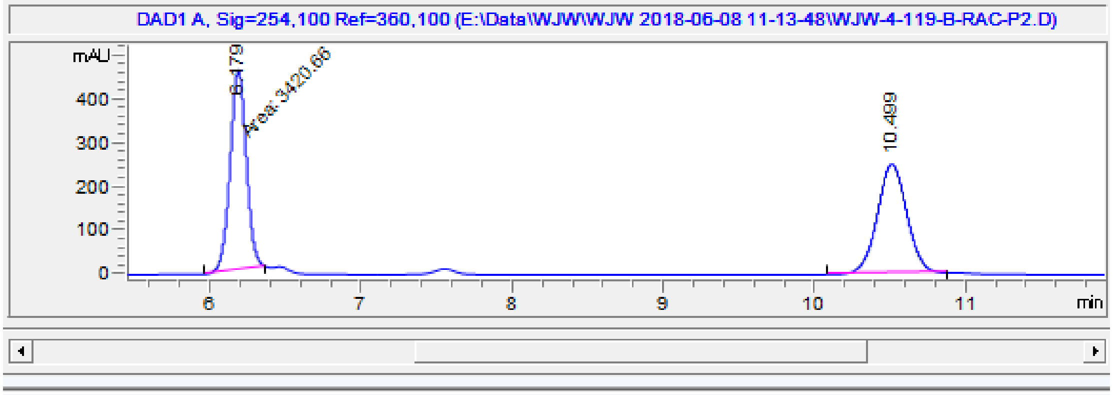

#	Time	Type	Area	Height	Width	Area%	Symmetry
1	6.179	MM	3420.7	460.1	0.1239	49.987	0.993
2	10.499	BB	3422.4	251.5	0.2098	50.013	0.956

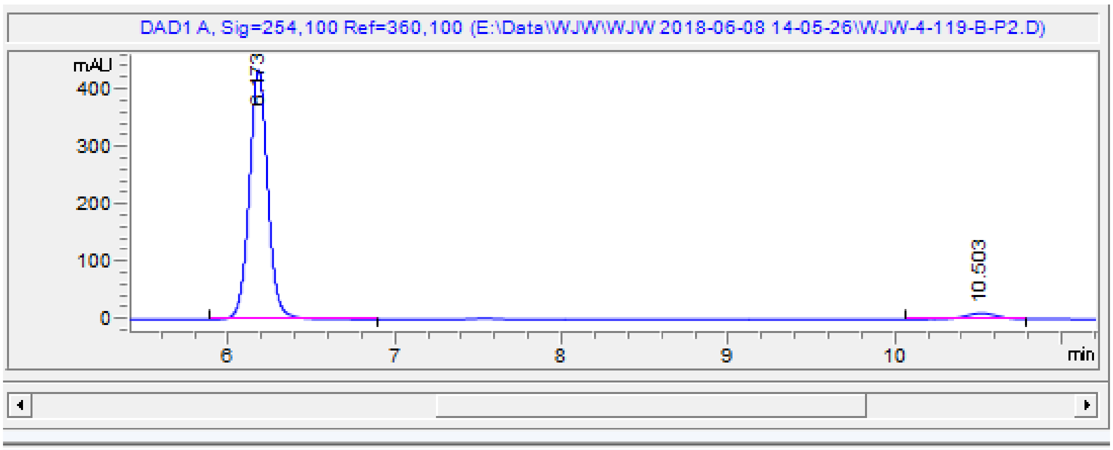

#	Time	Type	Area	Height	Width	Area%	Symmetry
1	6.173	BB	3337.4	438.2	0.1163	96.005	0.932
2	10.503	BB	138.9	10.3	0.1753	3.995	1.094

(*S*)-4-((*1R*,*3R*)-2-acetyl-1-(4-methoxyphenyl)-4-phenylbuta-2,3-dien-1-yl)-2-(3,5-dimethoxyphenyl)-4-ethyloxazol-5(4H)-one **(3o)**

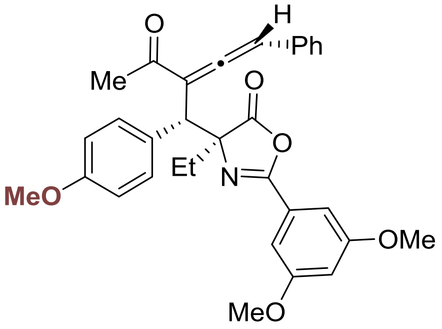

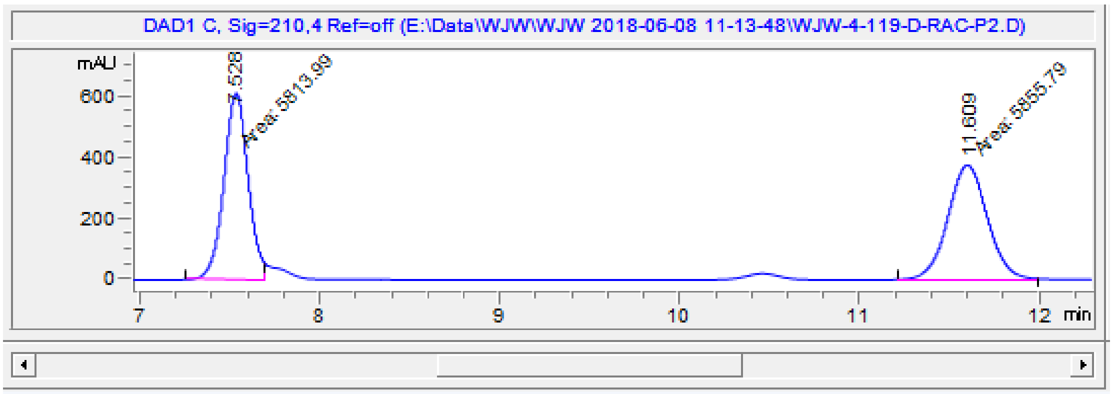

#	Time	Type	Area	Height	Width	Area%	Symmetry
1	7.528	MM	5814	608.8	0.1592	49.821	0.977
2	11.609	MM	5855.8	375.2	0.2601	50.179	0.949

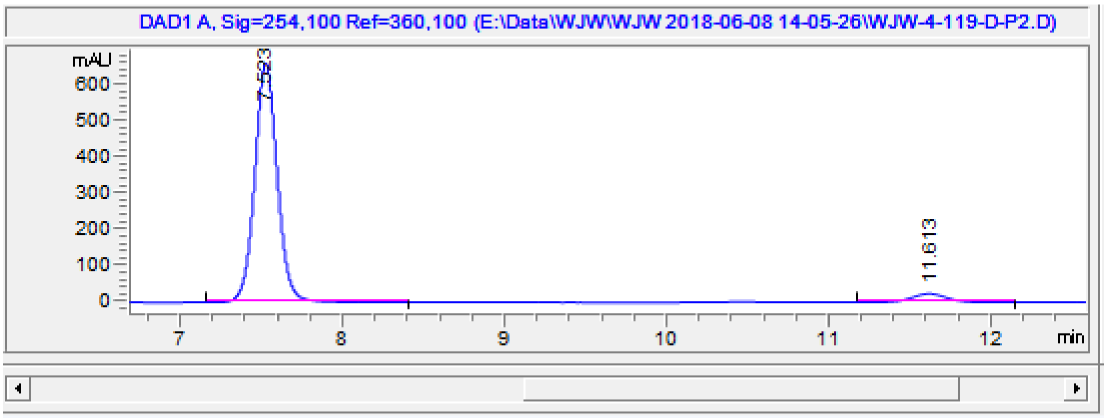

#	Time	Type	Area	Height	Width	Area%	Symmetry
1	7.523	BB	6354.2	663.7	0.1467	94.520	0.924
2	11.613	BB	368.4	24	0.2268	5.480	0.987

(*S*)-4-((*1R*,*3R*)-2-acetyl-1-(naphthalen-2-yl)-4-phenylbuta-2,3-dien-1-yl)-2-(3,5-dimethoxyphenyl)-4-ethyloxazol-5(4H)-one **(3p)**

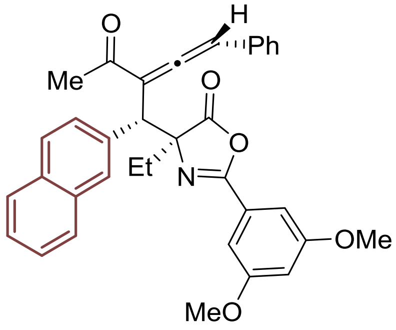

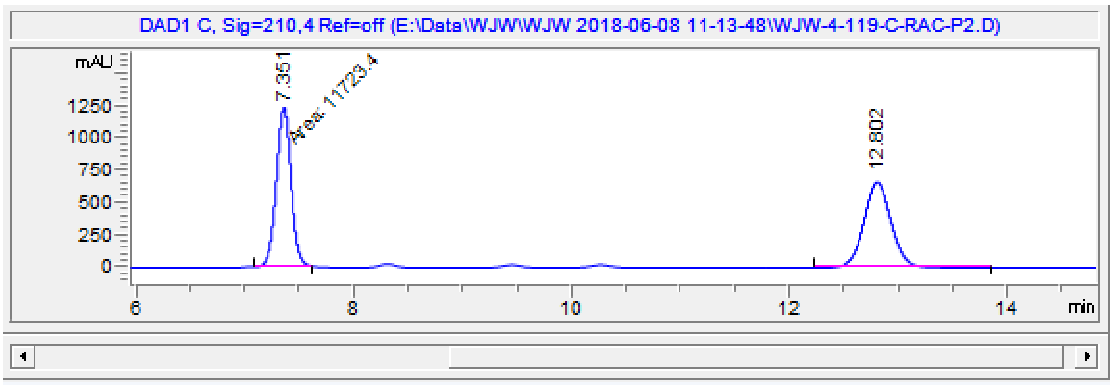

#	Time	Type	Area	Height	Width	Area%	Symmetry
1	7.351	MM	11723.4	1252.9	0.156	49.962	0.981
2	12.802	BB	11741.2	673.3	0.266	50.038	0.92

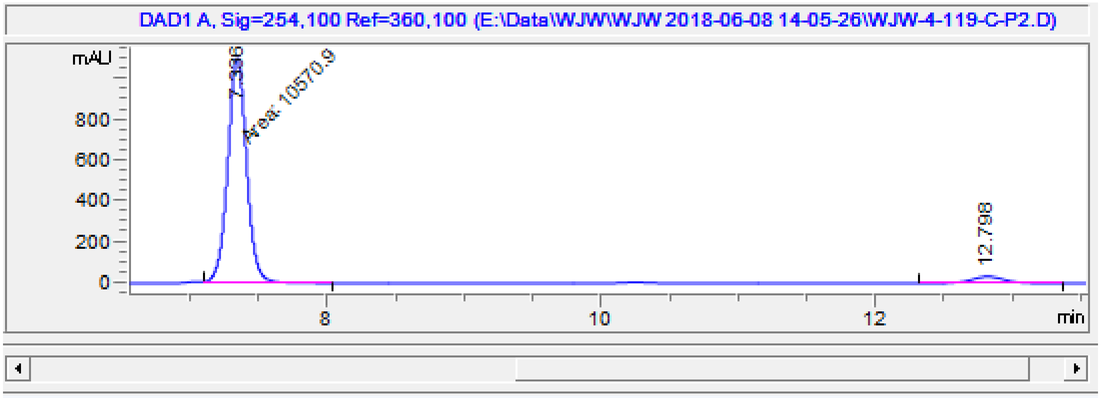

#	Time	Type	Area	Height	Width	Area%	Symmetry
1	7.336	FM	10570.9	1099.6	0.1602	95.056	0.949
2	12.798	BB	549.8	31.8	0.2557	4.944	0.987

(*S*)-4-((*1R*,*3R*)-2-acetyl-1-cyclohexyl-4-phenylbuta-2,3-dien-1-yl)-2-(3,5-dimethoxyphenyl)-4-ethyloxazol-5(4H)-one **(3q)**

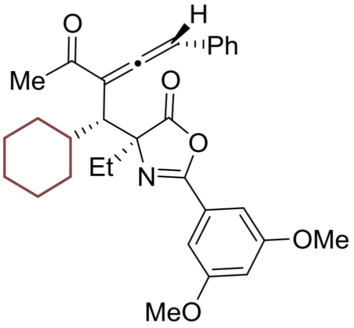

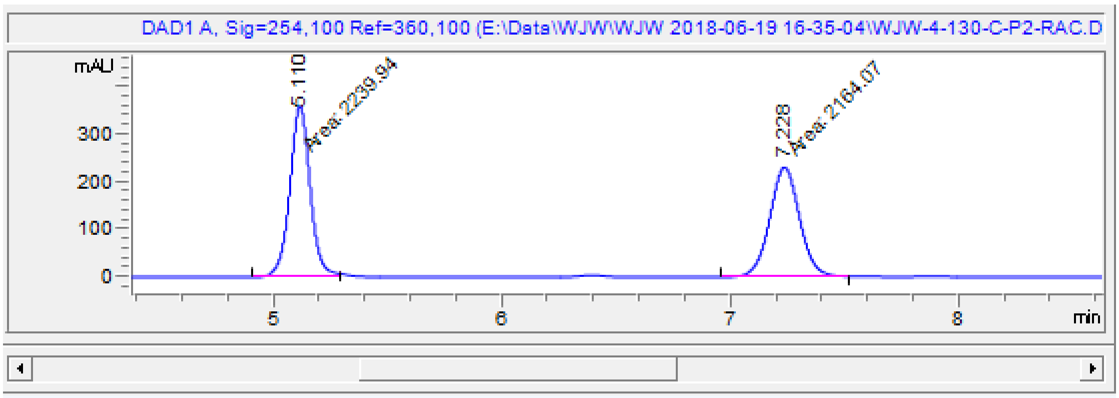

#	Time	Type	Area	Height	Width	Area%	Symmetry
1	5.11	MM	2239.9	361	0.1034	50.861	0.959
2	7.228	MM	2164.1	233.5	0.1544	49.139	0.969

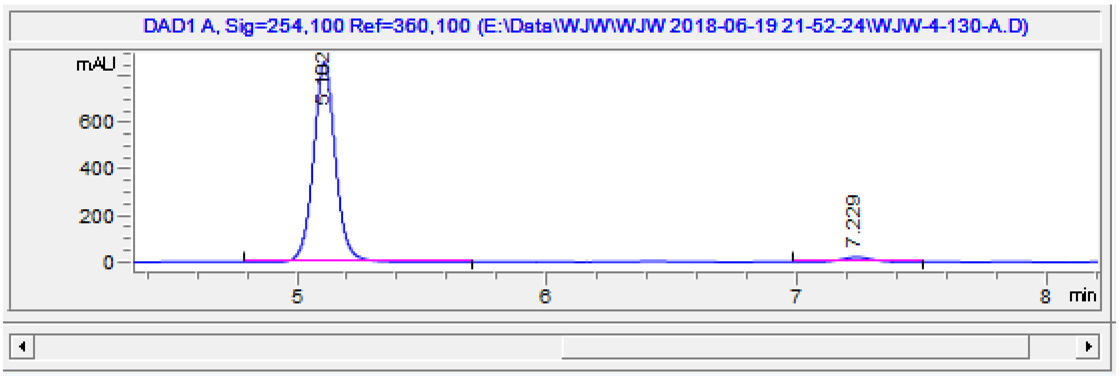

#	Time	Type	Area	Height	Width	Area%	Symmetry
1	5.102	BB	5272.7	858.2	0.0937	96.669	0.924
2	7.229	BB	181.7	20.4	0.1371	3.331	0.971

(*S*)-2-(3,5-dimethoxyphenyl)-4-ethyl-4-((*R*)-3-oxo-1-phenyl-2-((*R*)-2-phenylvinylidene)pentyl)oxazol-5(4H)-one **(3r)**

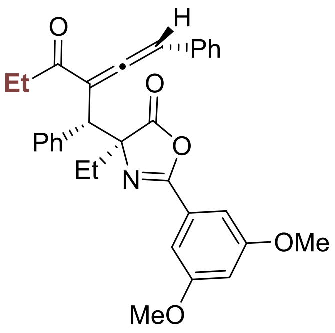

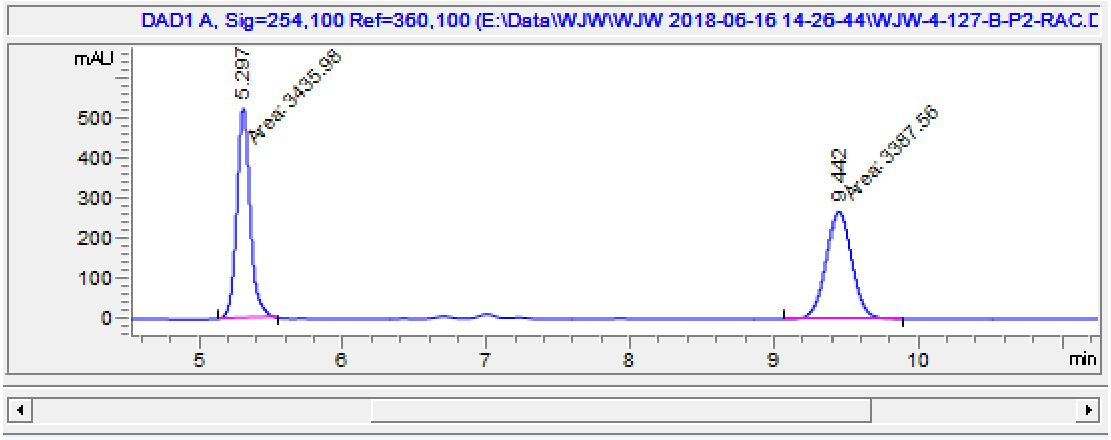

#	Time	Type	Area	Height	Width	Area%	Symmetry
1	5.297	MM	3436	525.5	0.109	50.355	0.903
2	9.442	MM	3387.6	270.7	0.2086	49.645	0.958

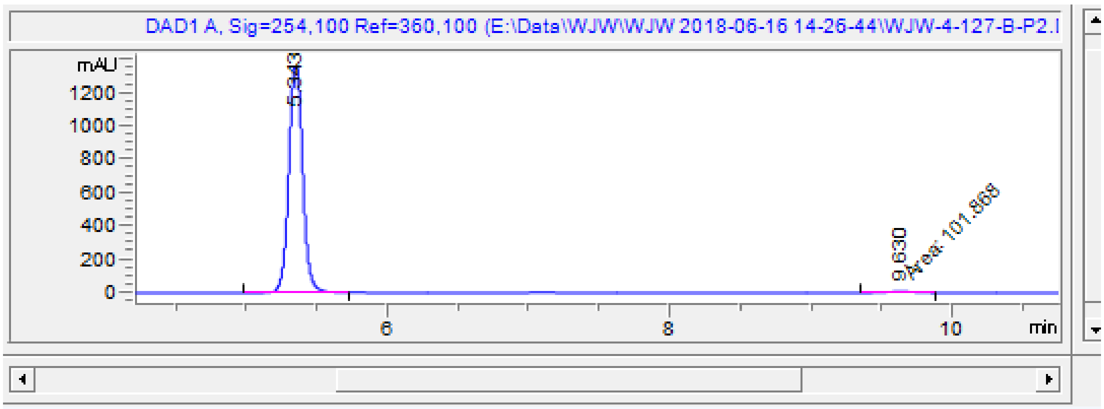

#	Time	Type	Area	Height	Width	Area%	Symmetry
1	5.343	BB	9255.2	1363.6	0.1052	98.911	0.939
2	9.63	MM	101.9	8.4	0.203	1.089	1.028

(*S*)-2-(3,5-dimethoxyphenyl)-4-ethyl-4-((*R*)-3-oxo-1-phenyl-2-((*R*)-2-phenylvinylidene)hexyl)oxazol-5(4H)-one **(3s)**

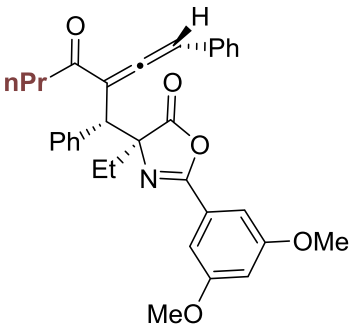

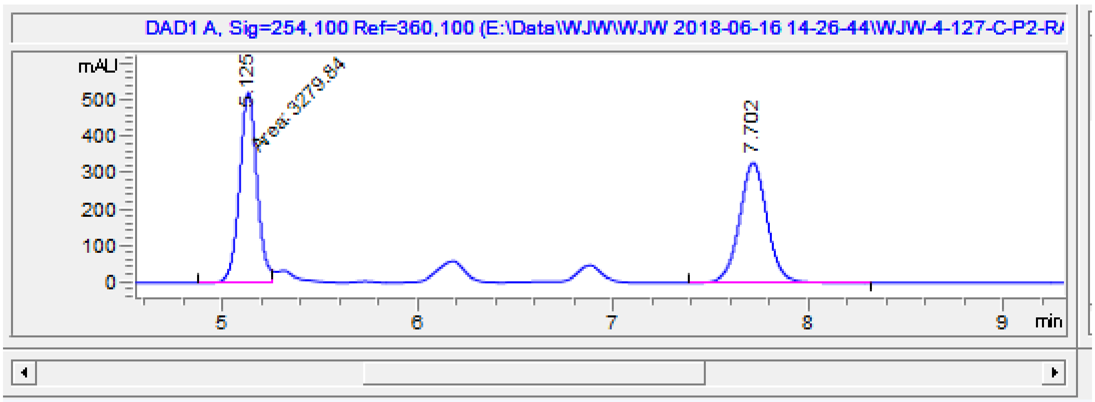

#	Time	Type	Area	Height	Width	Area%	Symmetry
1	5.125	MF	3279.8	526.9	0.1038	50.265	0.944
2	7.702	BB	3245.2	331.9	0.1491	49.735	0.952

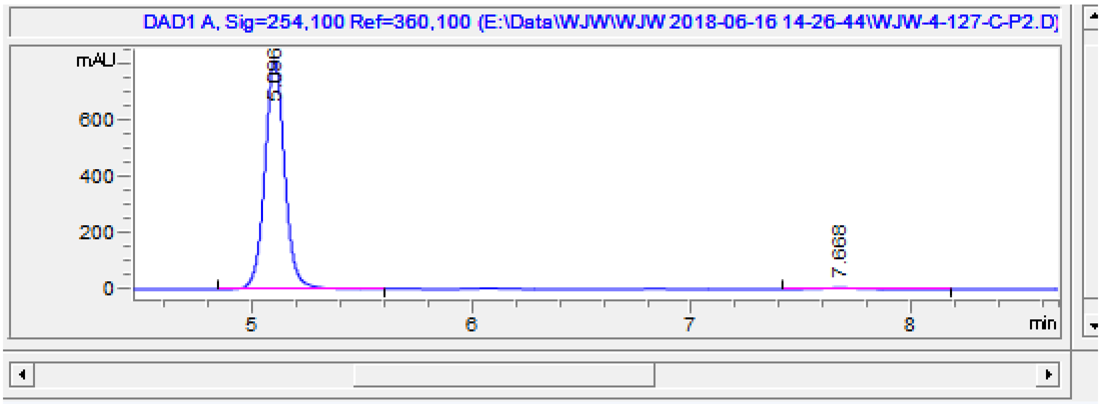

#	Time	Type	Area	Height	Width	Area%	Symmetry
1	5.096	BB	5027.5	813.7	0.0941	99.076	0.925
2	7.668	BB	46.9	4.8	0.1482	0.924	0.966

(*S*)-2-(3,5-dimethoxyphenyl)-4-ethyl-4-((*R*)-3-oxo-1-phenyl-2-((*R*)-2-phenylvinylidene)heptyl)oxazol-5(4H)-one **(3t)**

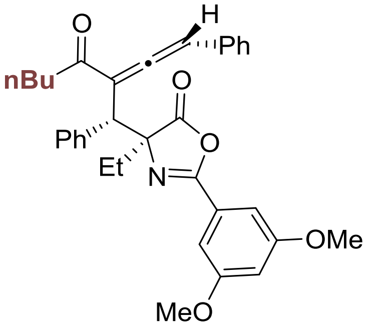

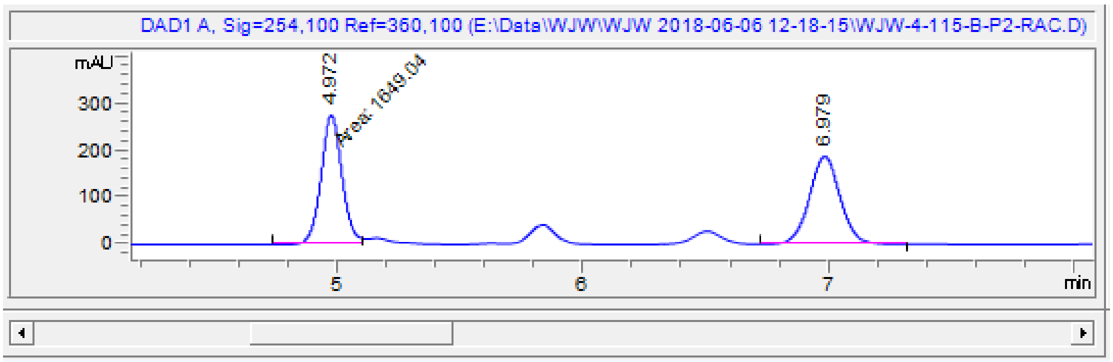

#	Time	Type	Area	Height	Width	Area%	Symmetry
1	4.972	MF	1649	275.4	0.0998	50.053	0.926
2	6.979	VB	1645.5	187.3	0.135	49.947	0.965

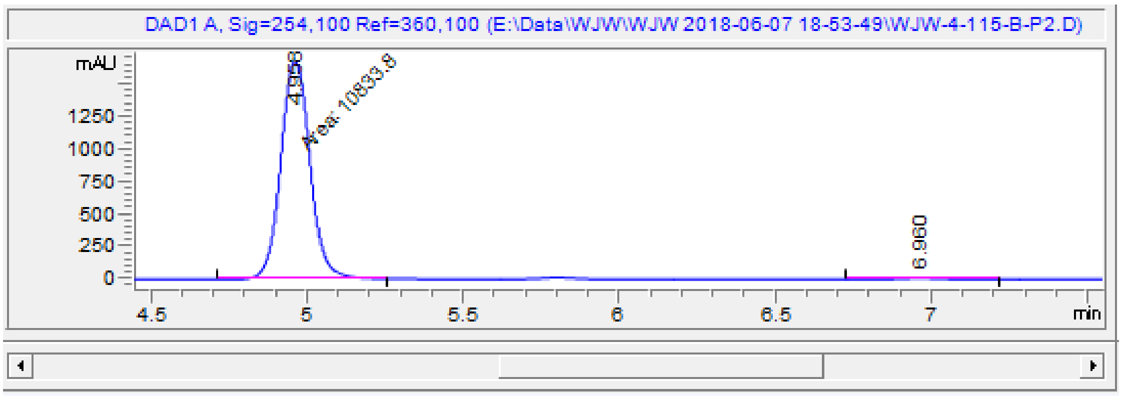

#	Time	Type	Area	Height	Width	Area%	Symmetry
1	4.958	MM	10833.8	1660.4	0.1087	99.418	0.91
2	6.96	BB	63.4	7.3	0.1297	0.582	1.005

(*S*)-2-(3,5-dimethoxyphenyl)-4-ethyl-4-((*R*)-5-methyl-3-oxo-1-phenyl-2-((*R*)-2-phenylvinylidene)hexyl)oxazol-5(4H)-one **(3u)**

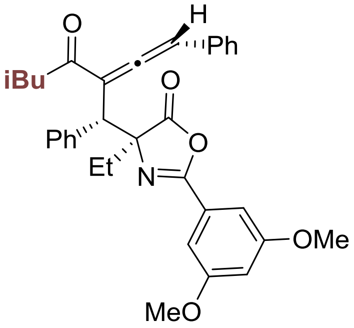

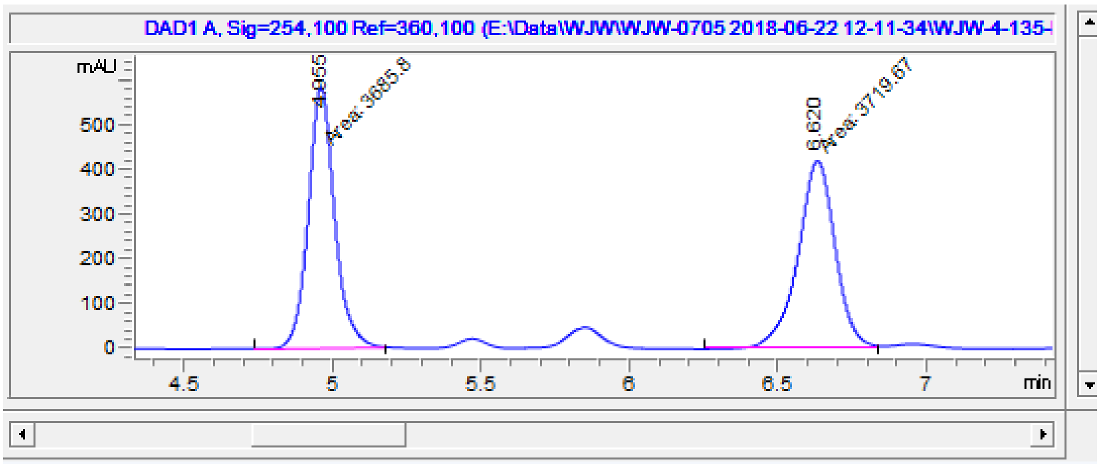

#	Time	Type	Area	Height	Width	Area%	Symmetry
1	4.955	MM	3685.8	589.2	0.1043	49.771	0.883
2	6.62	MM	3719.7	422.2	0.1468	50.229	1.075

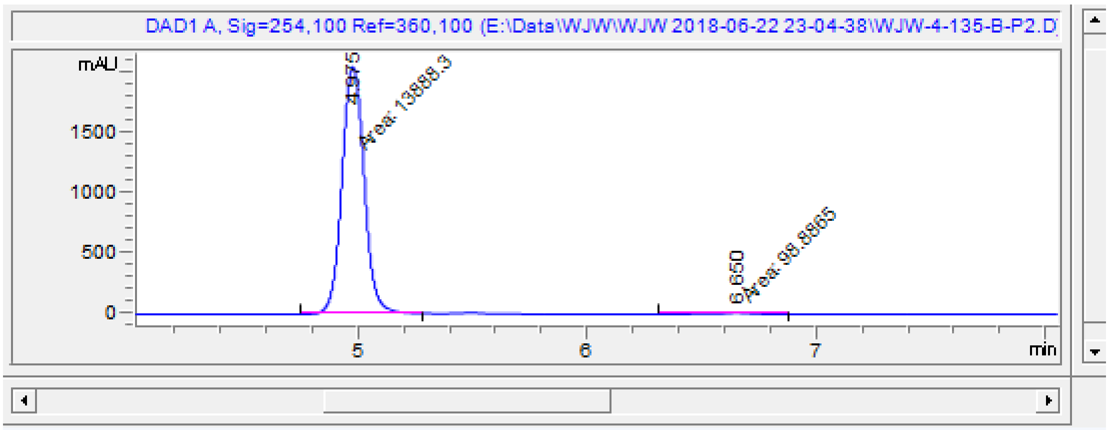

#	Time	Type	Area	Height	Width	Area%	Symmetry
1	4.975	MM	13888.3	2049.9	0.1129	99.293	0.942
2	6.65	MM	98.9	10	0.1643	0.707	1.416

(S)-4-((*1R*,*3R*)-2-acetyl-1,4-diphenylbuta-2,3-dien-1-yl)-4-butyl-2-(3,5-dimethoxyphenyl)oxazol-5(4H)-one **(3v)**

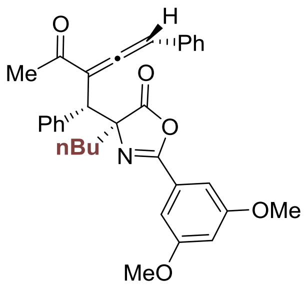

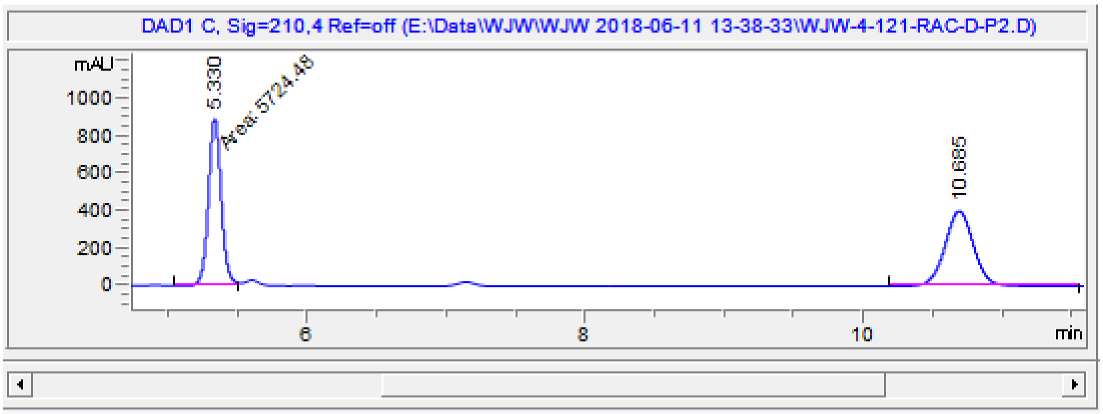

#	Time	Type	Area	Height	Width	Area%	Symmetry
1	5.33	MF	5724.5	887.7	0.1075	50.021	0.924
2	10.685	BB	5719.7	399	0.2197	49.979	0.974

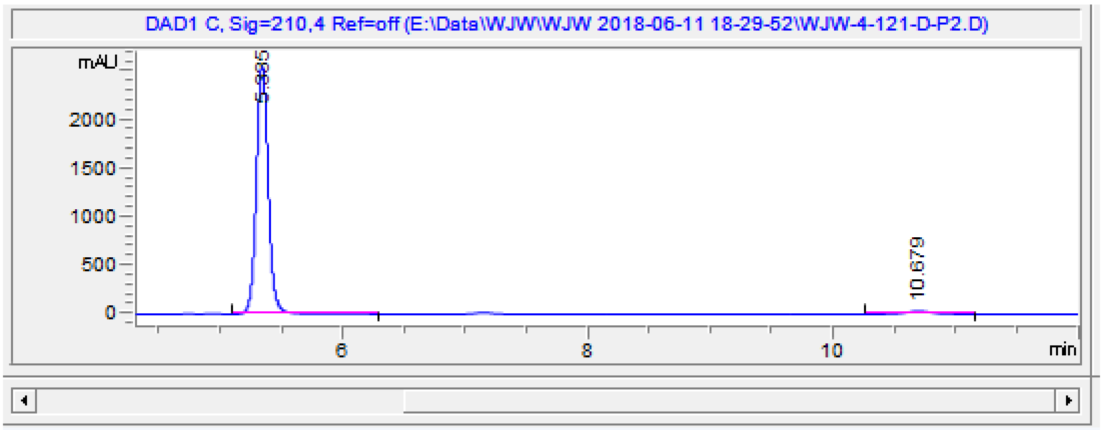

#	Time	Type	Area	Height	Width	Area%	Symmetry
1	5.335	BV R	17466.7	2576	0.1051	97.485	0.893
2	10.679	BB	450.6	31.5	0.1846	2.515	0.986

(*S*)-4-((*1R,3R*)-2-acetyl-1,4-diphenylbuta-2,3-dien-1-yl)-4-benzyl-2-(3,5-dimethoxyphenyl)oxazol-5(4H)-one **(3w)**

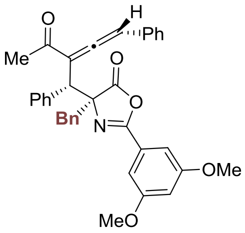

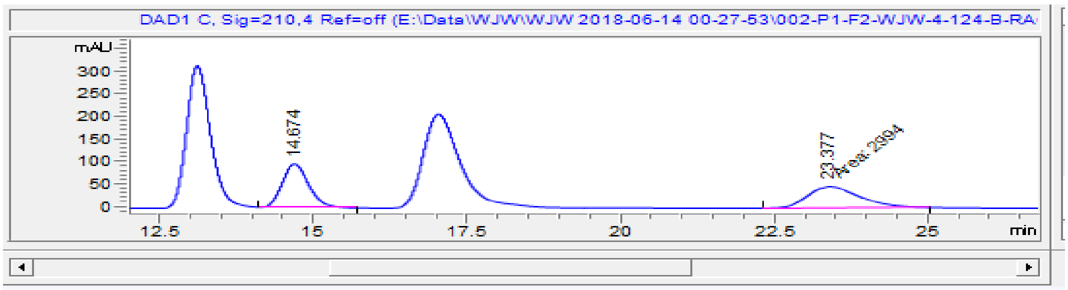

#	Time	Type	Area	Height	Width	Area%	Symmetry
1	14.674	VB	2962.3	98.2	0.3719	49.734	0.823
2	23.377	MM	2994	48.6	1.0262	50.266	0.68

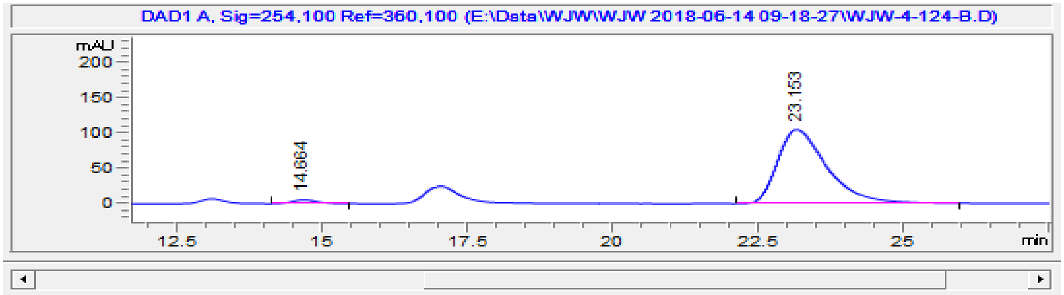

#	Time	Type	Area	Height	Width	Area%	Symmetry
1	14.664	BB	165.5	5.5	0.3536	2.573	0.814
2	23.153	BB	6266	105.9	0.6918	97.427	0.601

The minor diastereomer:

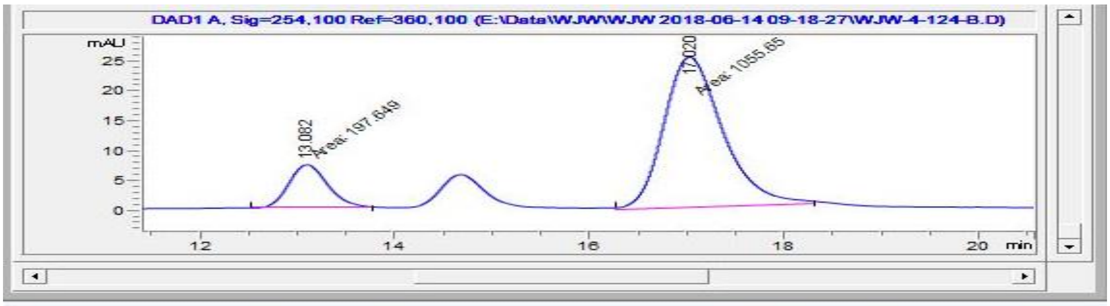

#	Time	Type	Area	Height	Width	Area%	Symmetry
1	13.082	MM	197.6	7.2	0.4602	15.770	0.786
2	17.02	MM	1055.6	25	0.7026	84.230	0.748

(*S*)-4-((*1R*,*3R*)-2-acetyl-1,4-diphenylbuta-2,3-dien-1-yl)-2-(3,5-dimethoxyphenyl)-4-(2-(methylthio)ethyl)oxazol-5(4H)-one **(3x)**

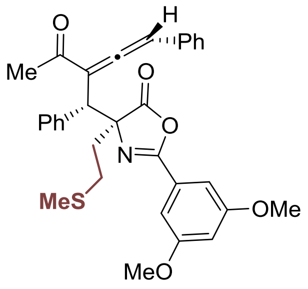

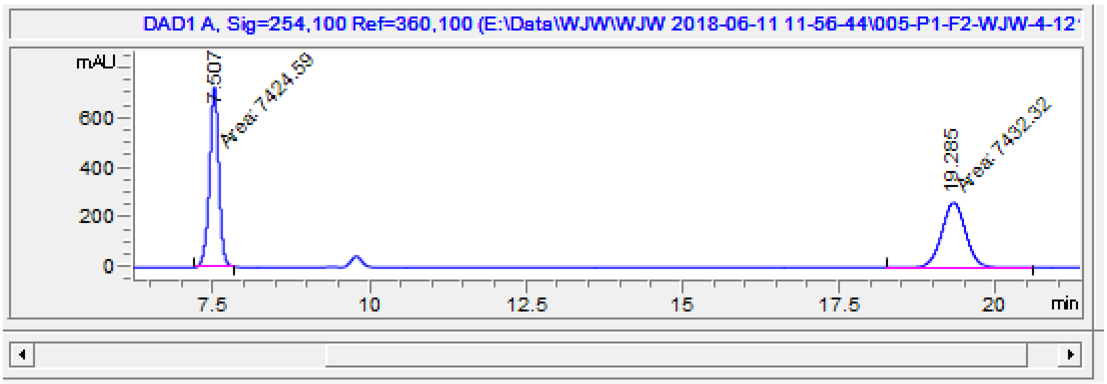

#	Time	Type	Area	Height	Width	Area%	Symmetry
1	7.507	MM	7424.6	721.2	0.1716	49.974	1.052
2	19.285	MM	7432.3	266.2	0.4653	50.026	0.983

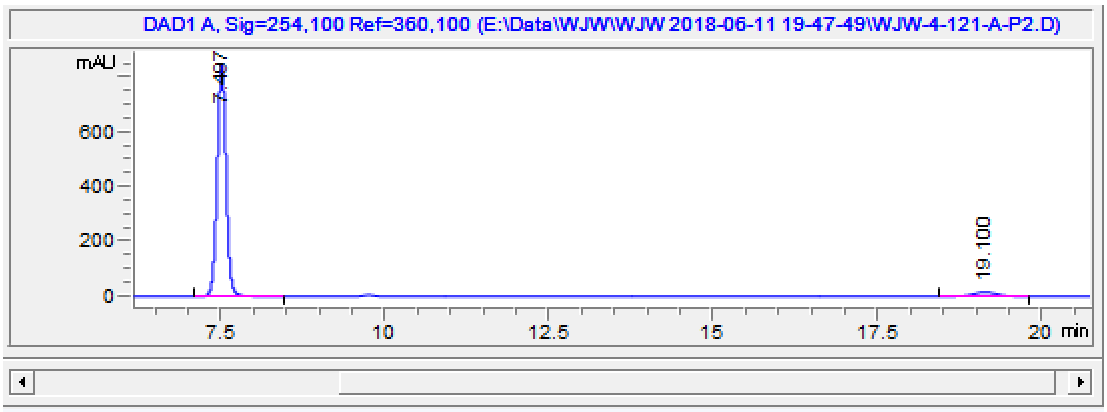

#	Time	Type	Area	Height	Width	Area%	Symmetry
1	7.497	BB	8067.1	852.6	0.1459	95.263	0.952
2	19.1	BB	401.1	15.5	0.3035	4.737	1.004

tert-butyl-(4-((*S*)-4-((*1R*,*3R*)-2-acetyl-1,4-diphenylbuta-2,3-dien-1-yl)-2-(3,5-dimethoxyphenyl)-5-oxo-4,5-dihydrooxazol-4-yl)butyl)carbamate **(3y)**

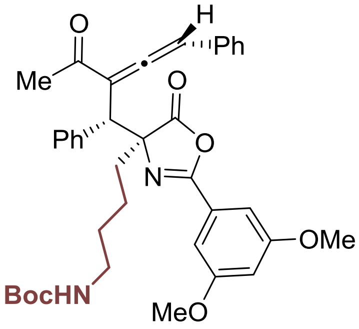

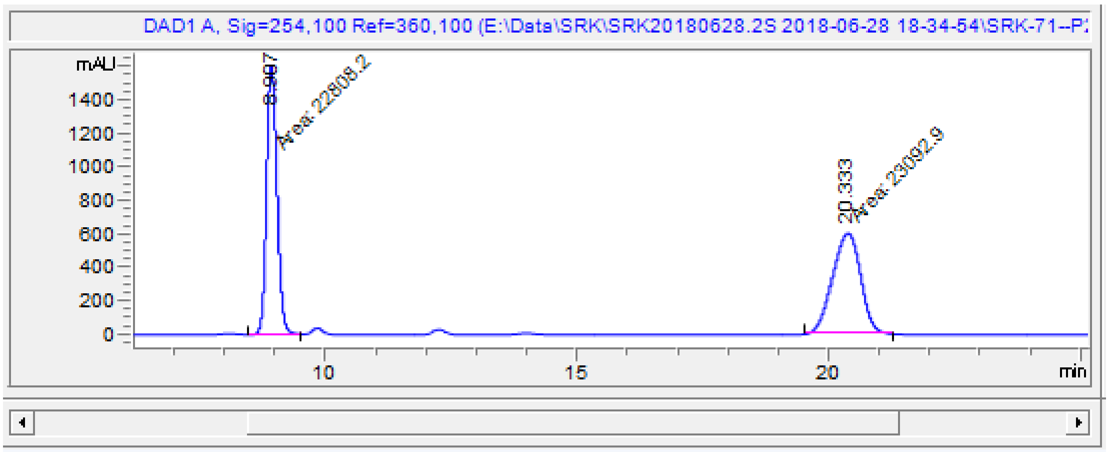

#	Time	Type	Area	Height	Width	Area%	Symmetry
1	8.907	MM	22808.2	1601.9	0.2373	49.690	0.838
2	20.333	MM	23092.9	599.1	0.6424	50.310	1.17

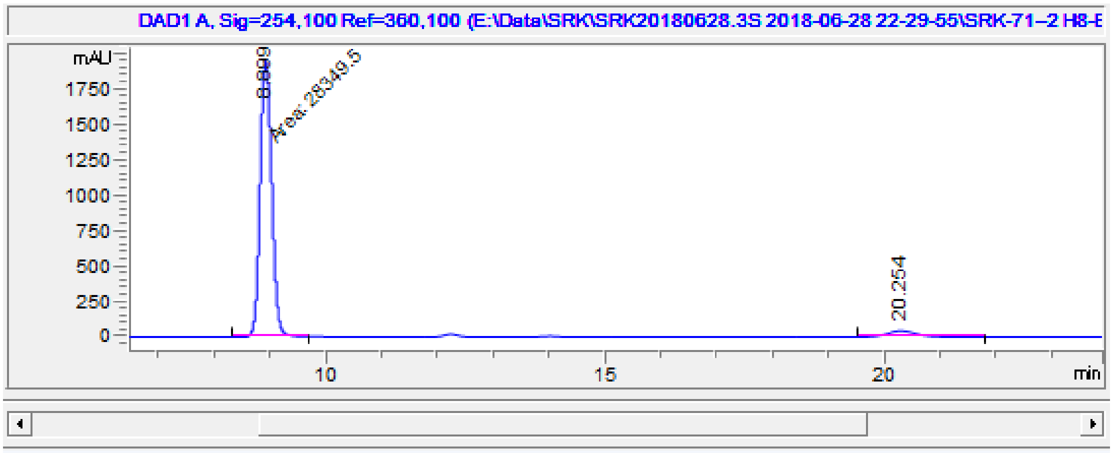

#	Time	Type	Area	Height	Width	Area%	Symmetry
1	8.899	MM	28349.5	1946.9	0.2427	94.492	0.841
2	20.254	BB	1652.5	45.8	0.5156	5.508	0.842

(*S*)-4-((*1R*,*3S*)-2-acetyl-1,4-diphenylbuta-2,3-dien-1-yl)-2-(3,5-dimethoxyphenyl)-4-ethyloxazol-5(4H)-one **(4a)**

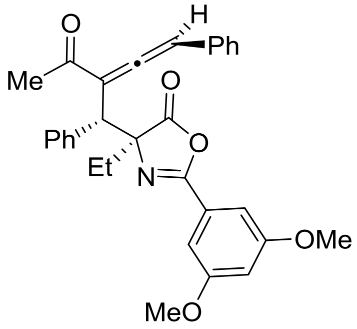

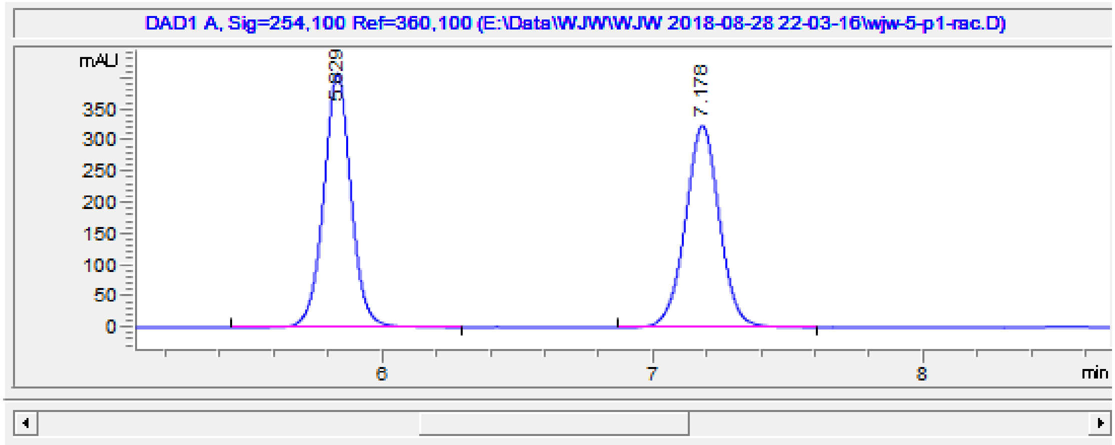

#	Time	Type	Area	Height	Width	Area%	Symmetry
1	5.829	BB	2877.5	407.3	0.1079	50.310	0.967
2	7.178	BB	2842	323.5	0.1345	49.690	0.979

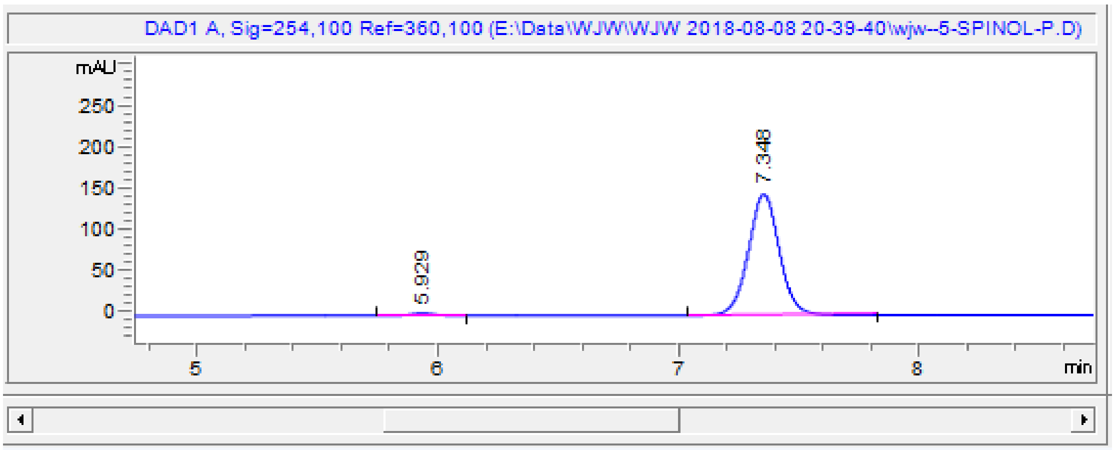

#	Time	Type	Area	Height	Width	Area%	Symmetry
1	5.929	BB	20	2.8	0.0907	1.487	1.105
2	7.348	BB	1325.6	147.8	0.1382	98.513	0.96

(*S*)-4-((*1R*,*3S*)-2-acetyl-1-phenyl-4-(p-tolyl)buta-2,3-dien-1-yl)-2-(3,5-dimethoxyphenyl)-4-ethyloxazol-5(4H)-one **(4b)**

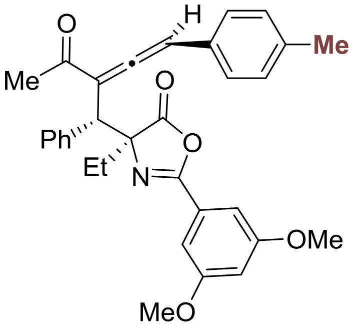

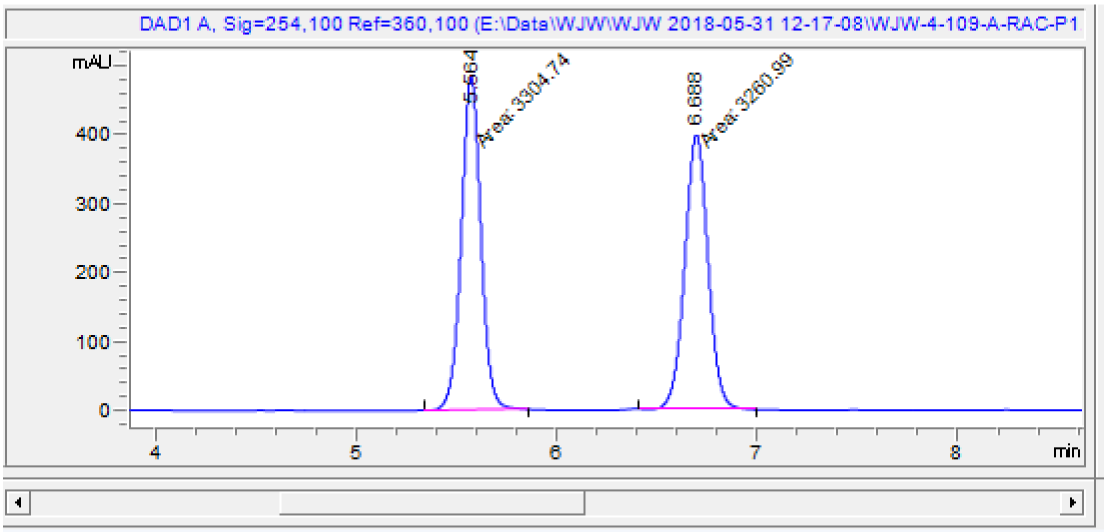

#	Time	Type	Area	Height	Width	Area%	Symmetry
1	5.564	FM	3304.7	483.1	0.114	50.333	0.964
2	6.688	MF	3261	399.7	0.136	49.667	0.97

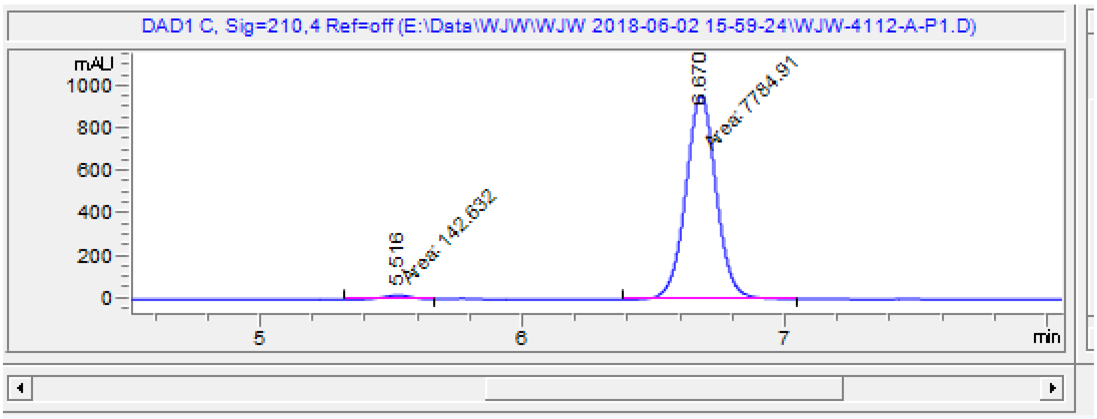

#	Time	Type	Area	Height	Width	Area%	Symmetry
1	5.516	MM	142.6	19.3	0.1234	1.799	1.145
2	6.67	MM	7784.9	959.9	0.1352	98.201	0.968

(*S*)-4-((*1R*,*3S*)-2-acetyl-4-(4-chlorophenyl)-1-phenylbuta-2,3-dien-1-yl)-2-(3,5-dimethoxyphenyl)-4-ethyloxazol-5(4H)-one **(4c)**

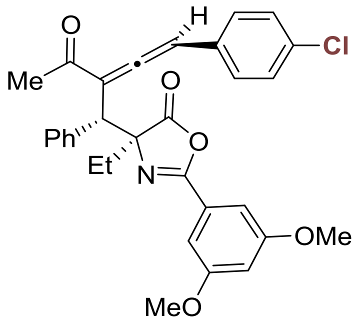

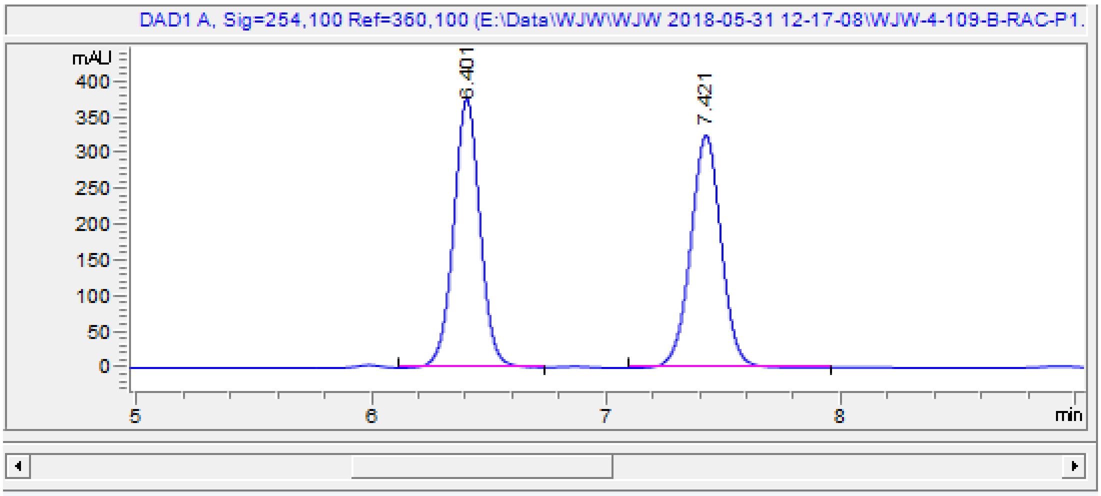

#	Time	Type	Area	Height	Width	Area%	Symmetry
1	6.401	BB	2948.9	376.1	0.1205	49.892	0.975
2	7.421	BB	2961.7	325.7	0.1401	50.108	0.991

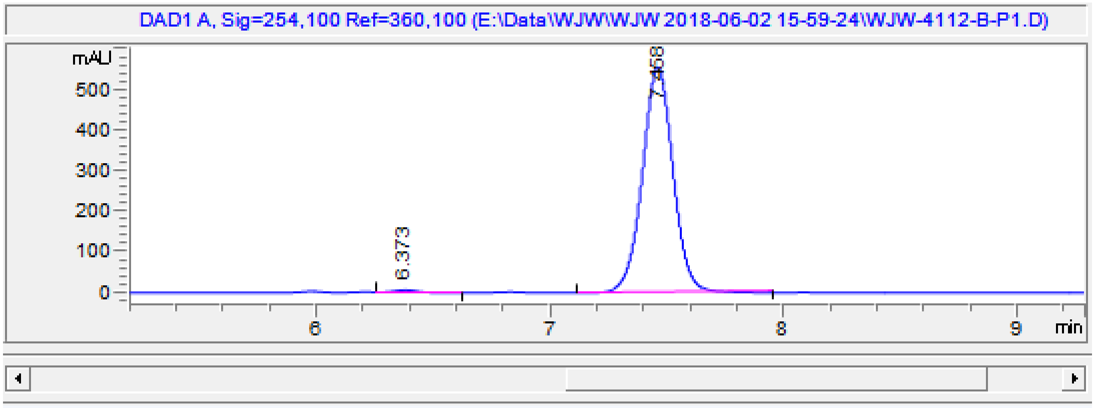

#	Time	Type	Area	Height	Width	Area%	Symmetry
1	6.373	VB	59.1	7.4	0.116	1.127	1.005
2	7.458	BB	5186.5	562.5	0.1411	98.873	0.983

(*S*)-4-((*1R*,*3S*)-2-acetyl-4-(4-methoxyphenyl)-1-phenylbuta-2,3-dien-1-yl)-2-(3,5-dimethoxyphenyl)-4-ethyloxazol-5(4H)-one **(4d)**

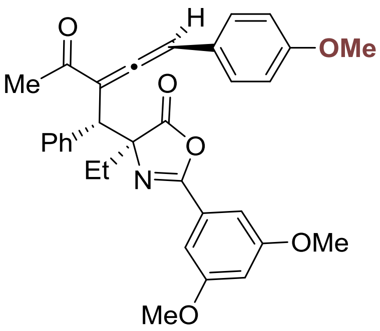

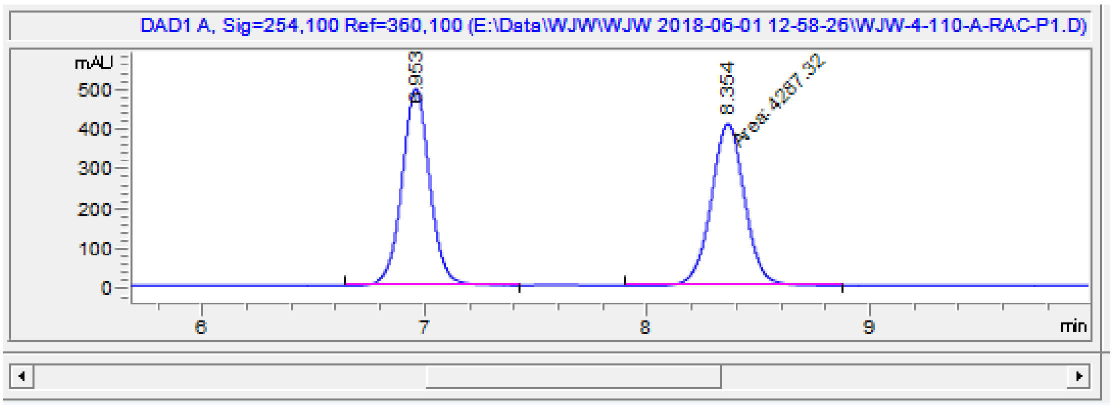

#	Time	Type	Area	Height	Width	Area%	Symmetry
1	6.953	BB	4288.6	491.4	0.1338	50.007	0.954
2	8.354	MM	4287.3	404.4	0.1767	49.993	0.961

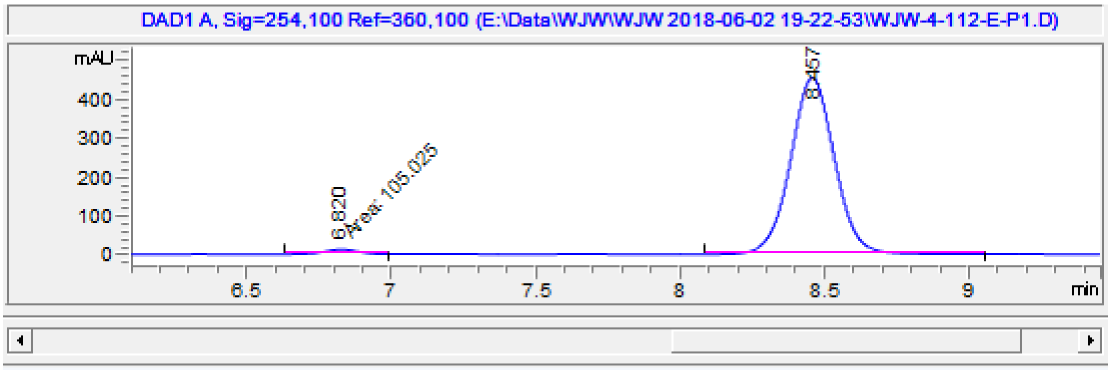

#	Time	Type	Area	Height	Width	Area%	Symmetry
1	6.82	MM	105	12.7	0.1376	2.114	0.988
2	8.457	BB	4863.3	458.9	0.1626	97.886	0.968

(*S*)-4-((*1R*,*3S*)-2-acetyl-4-(4-fluorophenyl)-1-phenylbuta-2,3-dien-1-yl)-2-(3,5-dimethoxyphenyl)-4-ethyloxazol-5(4H)-one **(4e)**

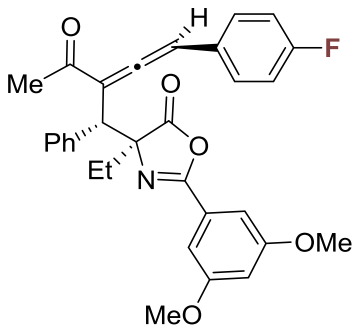

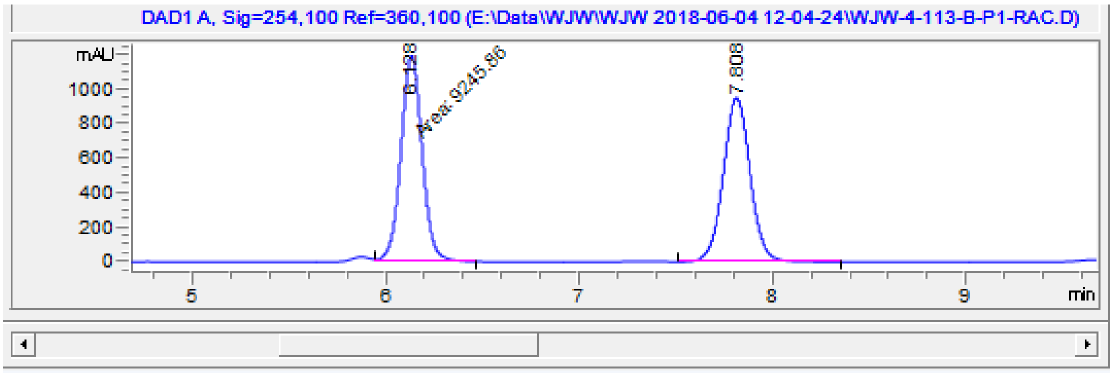

#	Time	Type	Area	Height	Width	Area%	Symmetry
1	6.128	MM	9245.9	1204	0.128	49.969	0.956
2	7.808	BB	9257.4	957	0.1489	50.031	0.945

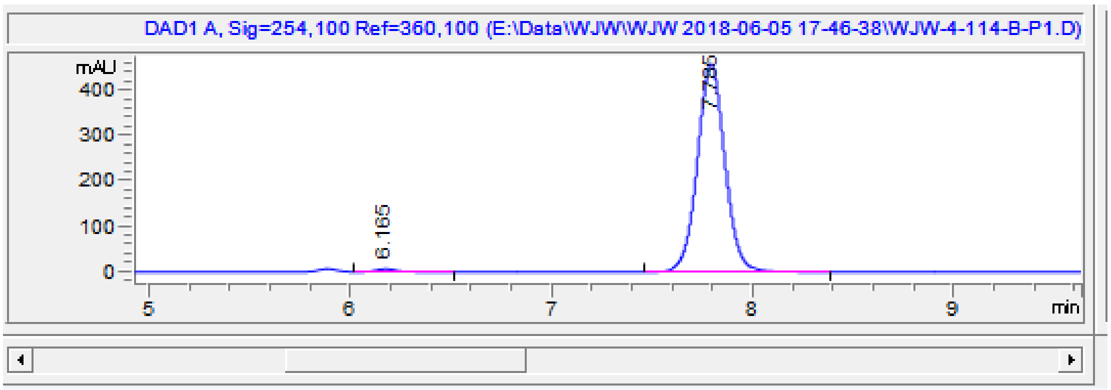

#	Time	Type	Area	Height	Width	Area%	Symmetry
1	6.165	VB	58	7.1	0.1181	1.296	0.83
2	7.785	BB	4418.9	463.3	0.1468	98.704	0.944

(*S*)-4-((*1R,3S*)-2-acetyl-1-phenyl-4-(4-(trifluoromethyl)phenyl)buta-2,3-dien-1-yl)-2-(3,5-dimethoxyphenyl)-4-ethyloxazol-5(4H)-one **(4f)**

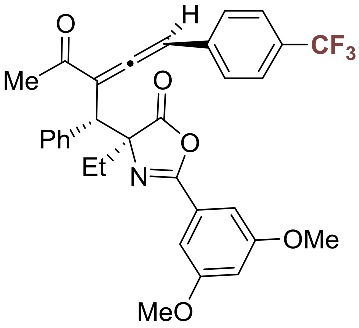

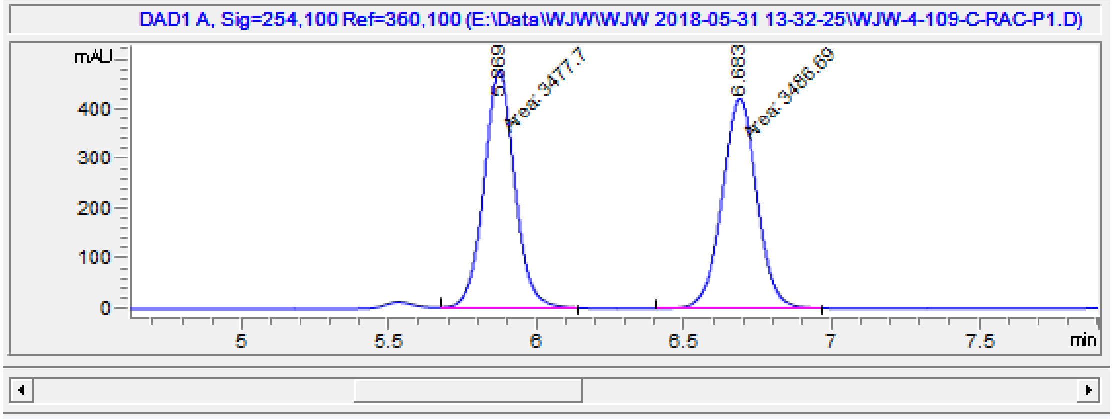

#	Time	Type	Area	Height	Width	Area%	Symmetry
1	5.869	MF	3477.7	474.6	0.1221	49.935	0.947
2	6.683	MM	3486.7	420.9	0.138	50.065	0.988

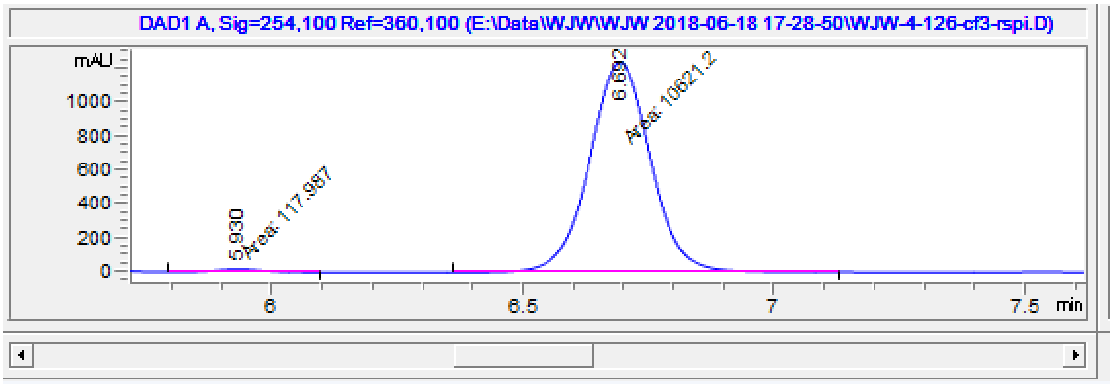

#	Time	Type	Area	Height	Width	Area%	Symmetry
1	5.93	MM	118	16.9	0.1162	1.099	0.91
2	6.692	MM	10621.2	1248.2	0.1418	98.901	0.979

Methyl-3-((*S*)-3-((*R*)-((*S*)-2-(3,5-dimethoxyphenyl)-4-ethyl-5-oxo-4,5-dihydrooxazol-4-yl)(phenyl)methyl)-4-oxopenta-1,2-dien-1-yl)benzoate **(4g)**

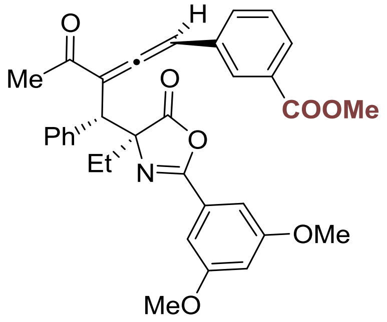

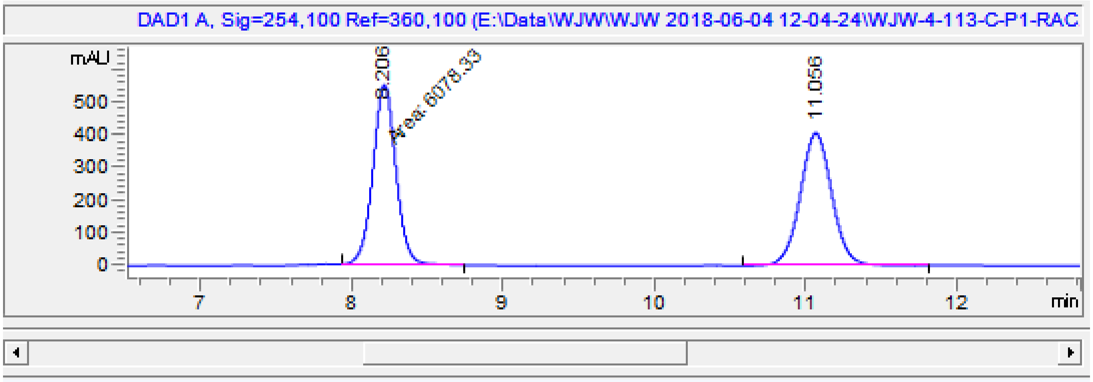

#	Time	Type	Area	Height	Width	Area%	Symmetry
1	8.206	MF	6078.3	563	0.1799	50.220	0.943
2	11.056	BB	6025	412.9	0.2247	49.780	0.946

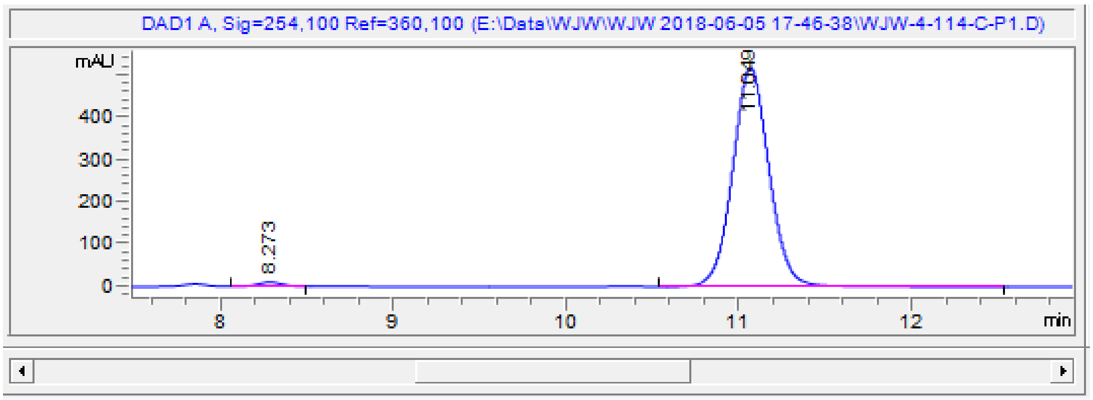

#	Time	Type	Area	Height	Width	Area%	Symmetry
1	8.273	BB	100.8	10.1	0.152	1.300	0.993
2	11.049	BB	7656.3	523	0.2253	98.700	0.919

(*S*)-4-((*1R*,*3S*)-2-acetyl-4-(2-chlorophenyl)-1-phenylbuta-2,3-dien-1-yl)-2-(3,5-dimethoxyphenyl)-4-ethyloxazol-5(4H)-one **(4h)**

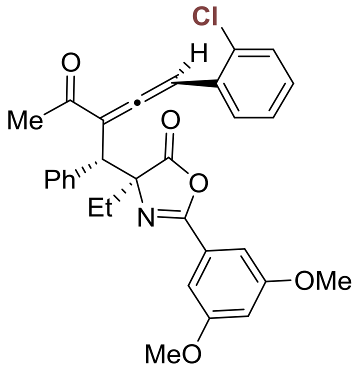

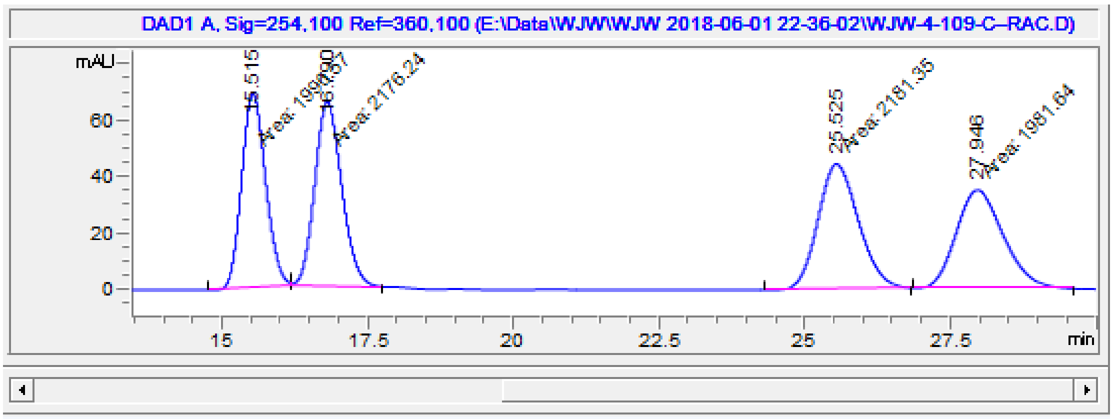

#	Time	Type	Area	Height	Width	Area%	Symmetry
1	15.515	MM	1990.6	69.8	0.4755	23.897	0.843
2	16.79	MM	2176.2	66.5	0.5451	26.126	0.829
3	25.525	MM	2181.4	44.9	0.8097	26.187	0.819
4	27.946	MM	1981.6	35.1	0.9402	23.790	0.804

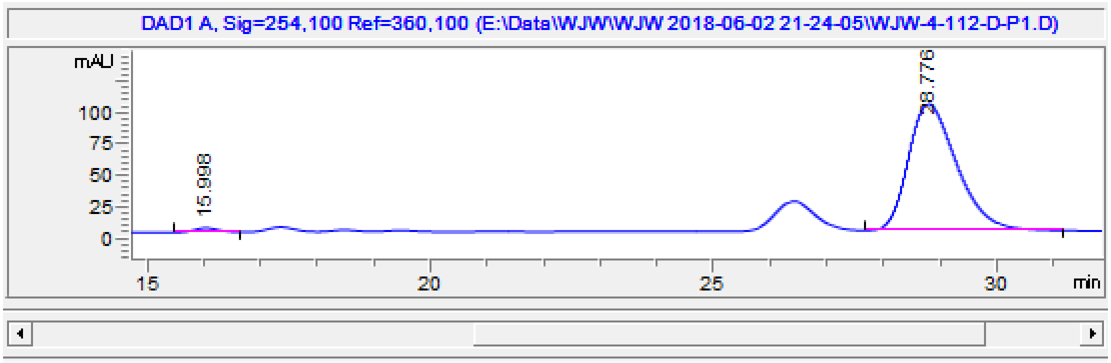

#	Time	Type	Area	Height	Width	Area%	Symmetry
1	15.998	BB	102.1	3.5	0.3422	1.665	0.918
2	28.776	BB	6028.6	101.4	0.6974	98.335	0.681

(*S*)-4-((*1R*,*3S*)-2-acetyl-1-phenyl-4-(thiophen-3-yl)buta-2,3-dien-1-yl)-2-(3,5-dimethoxyphenyl)-4-ethyloxazol-5(4H)-one **(4i)**

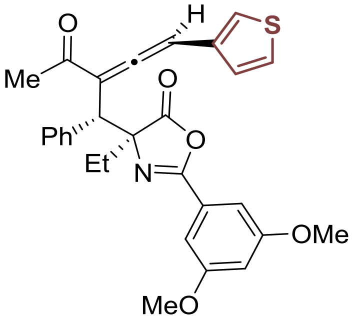

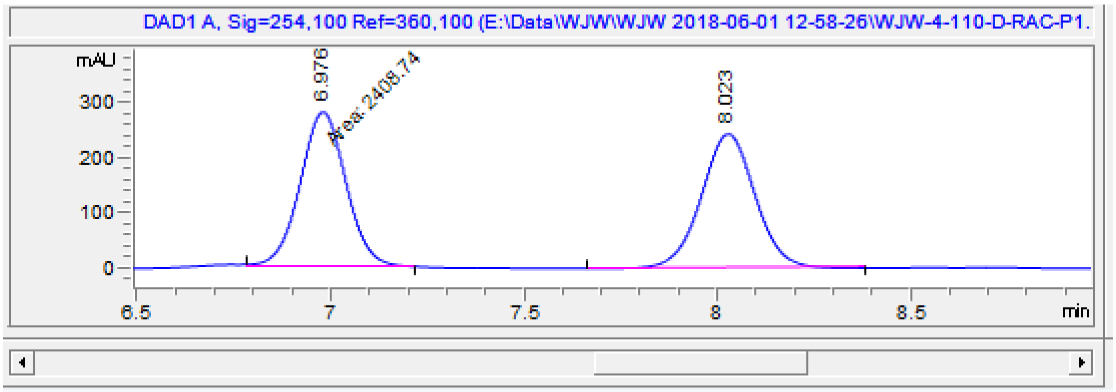

#	Time	Type	Area	Height	Width	Area%	Symmetry
1	6.976	MM	2408.7	285.8	0.1405	50.048	0.974
2	8.023	BB	2404.1	246.6	0.1492	49.952	0.977

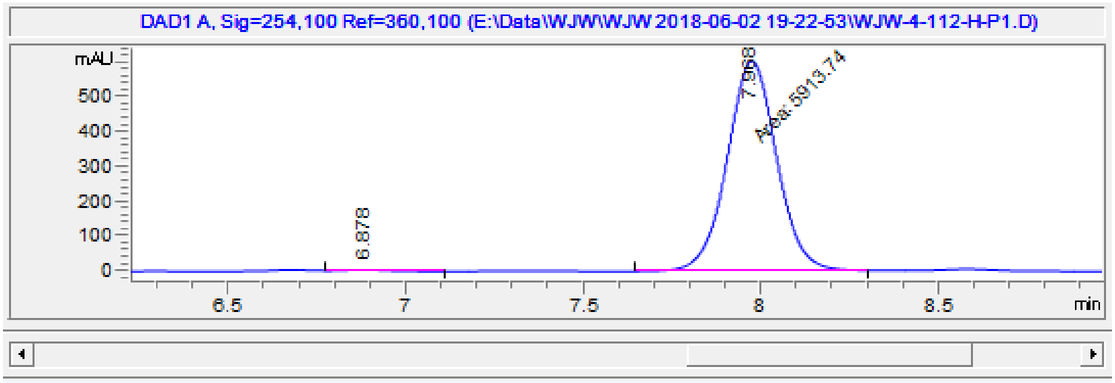

#	Time	Type	Area	Height	Width	Area%	Symmetry
1	6.878	VB	35.4	4.2	0.1209	0.596	0.991
2	7.968	MM	5913.7	608.8	0.1619	99.404	0.975

(*S*)-4-((*1R*,*3S*)-2-acetyl-4-(cyclohex-1-en-1-yl)-1-phenylbuta-2,3-dien-1-yl)-2-(3,5-dimethoxyphenyl)-4-ethyloxazol-5(4H)-one **(4j)**

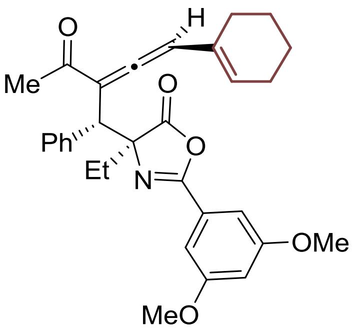

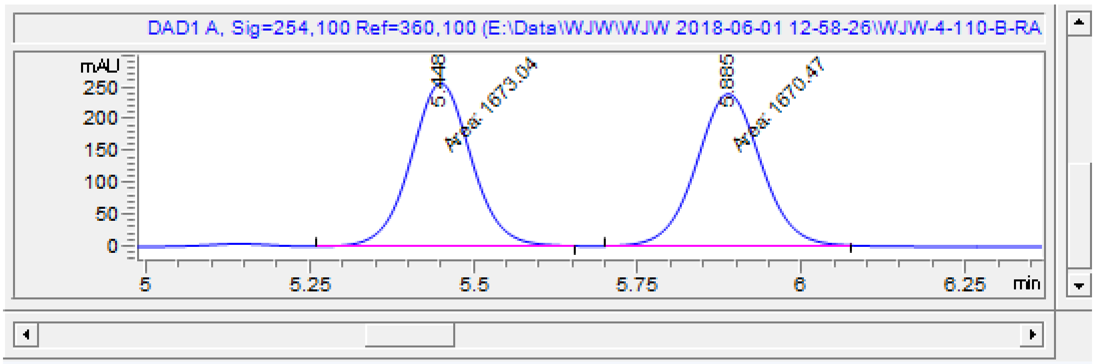

#	Time	Type	Area	Height	Width	Area%	Symmetry
1	5.448	MM	1673	255.7	0.109	50.038	0.946
2	5.885	MM	1670.5	238.5	0.1167	49.962	0.96

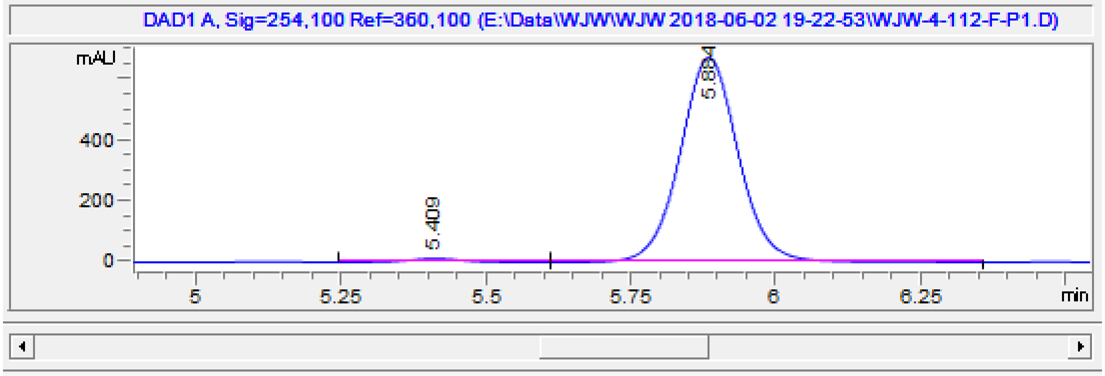

#	Time	Type	Area	Height	Width	Area%	Symmetry
1	5.409	VB	76.4	11.9	0.0978	1.595	0.981
2	5.884	BB	4716.5	670.7	0.108	98.405	0.946

(*S*)-4-((*1R*,*3S*)-2-acetyl-1-phenylocta-2,3-dien-1-yl)-2-(3,5-dimethoxyphenyl)-4-ethyloxazol-5(4H)-one **(4k)**

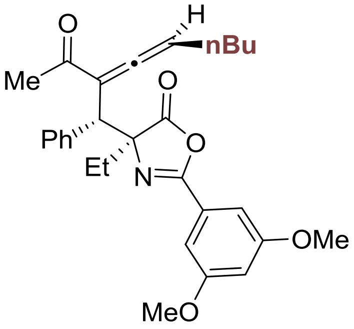

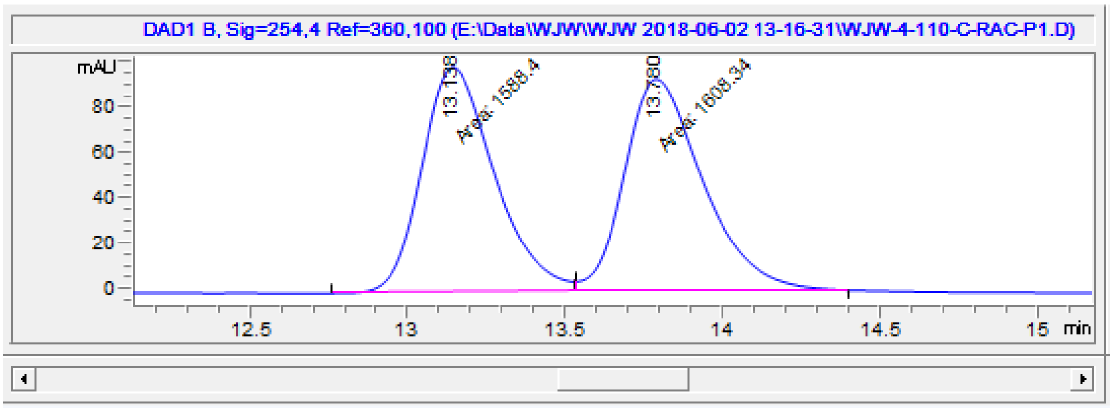

#	Time	Type	Area	Height	Width	Area%	Symmetry
1	13.138	MF	1588.4	99	0.2675	49.688	0.731
2	13.78	FM	1608.3	93.2	0.2875	50.312	0.669

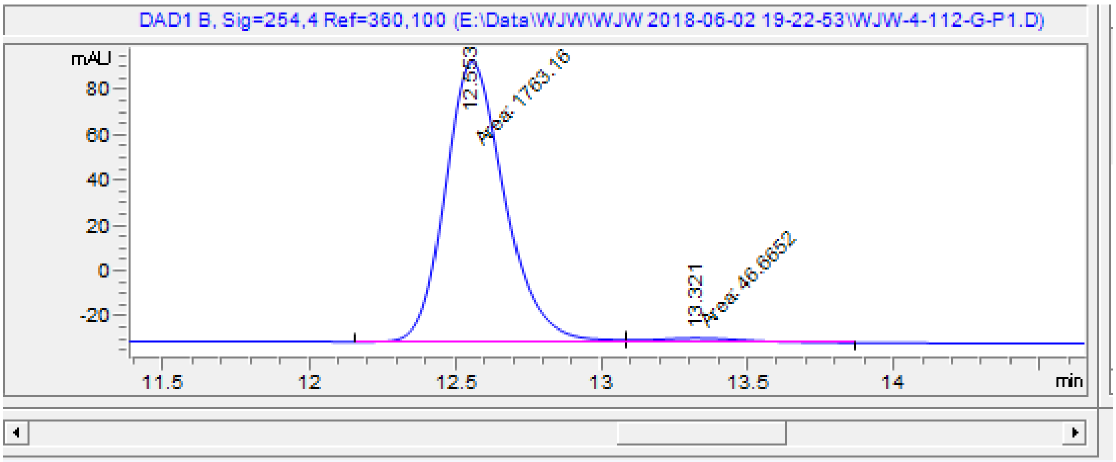

#	Time	Type	Area	Height	Width	Area%	Symmetry
1	12.553	MF	1763.2	123.5	0.2379	97.422	0.787
2	13.321	FM	46.7	2	0.3947	2.578	0.781

(*S*)-4-((*1R*,*3S*)-2-acetyl-4-phenyl-1-(p-tolyl)buta-2,3-dien-1-yl)-2-(3,5-dimethoxyphenyl)-4-ethyloxazol-5(4H)-one **(4l)**

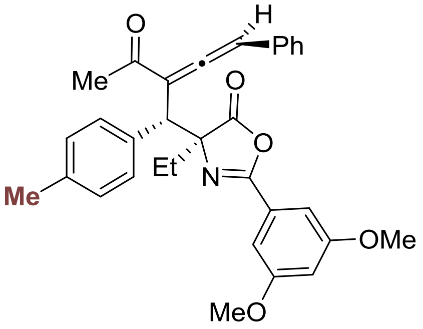

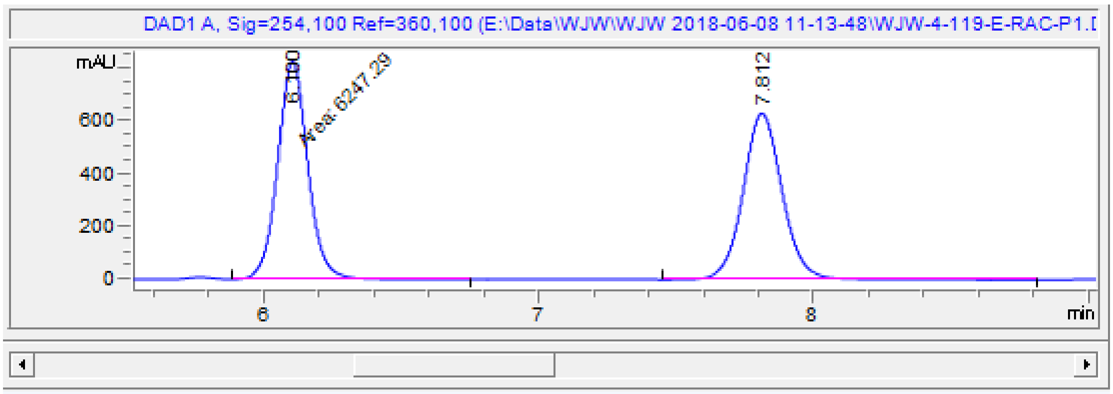

#	Time	Type	Area	Height	Width	Area%	Symmetry
1	6.1	FM	6247.3	824	0.1264	50.167	0.924
2	7.812	BB	6205.7	627	0.1515	49.833	0.904

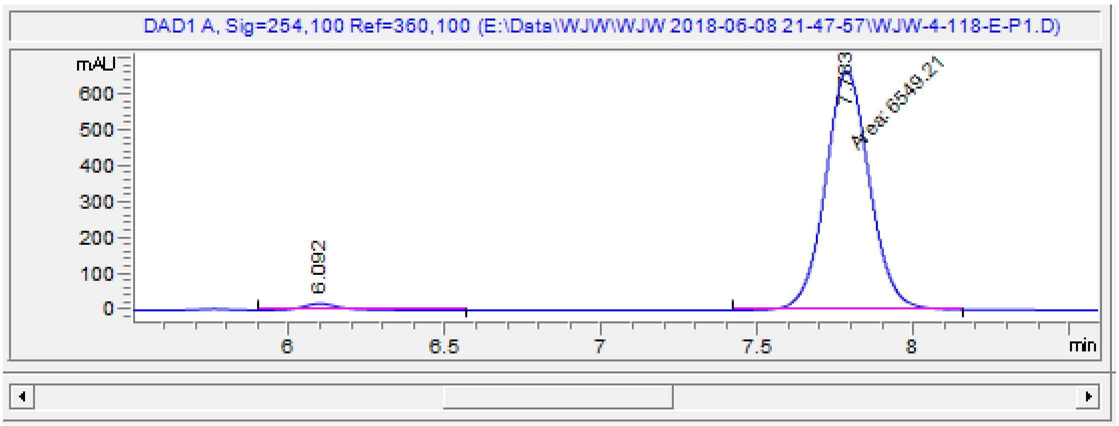

#	Time	Type	Area	Height	Width	Area%	Symmetry
1	6.092	BB	148.5	18.1	0.1221	2.218	0.769
2	7.783	MF	6549.2	662.5	0.1648	97.782	0.915

(*S*)-4-((*1R*,*3S*)-2-acetyl-1-(4-fluorophenyl)-4-phenylbuta-2,3-dien-1-yl)-2-(3,5-dimethoxyphenyl)-4-ethyloxazol-5(4H)-one **(4m)**

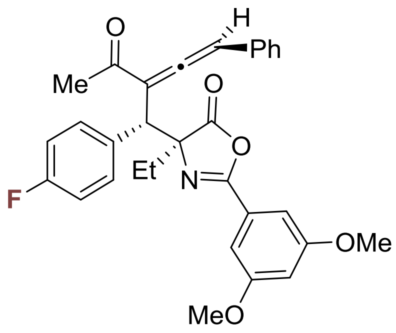

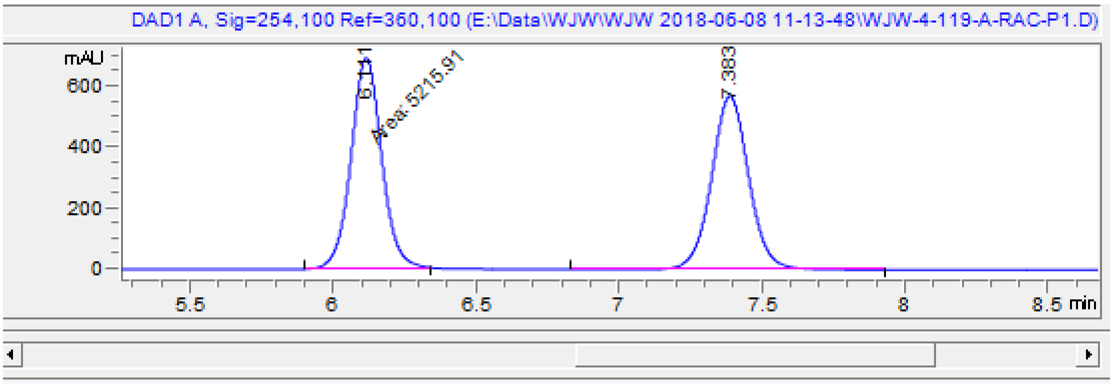

#	Time	Type	Area	Height	Width	Area%	Symmetry
1	6.111	FM	5215.9	692.6	0.1255	50.174	0.934
2	7.383	VB R	5179.6	568	0.1402	49.826	0.952

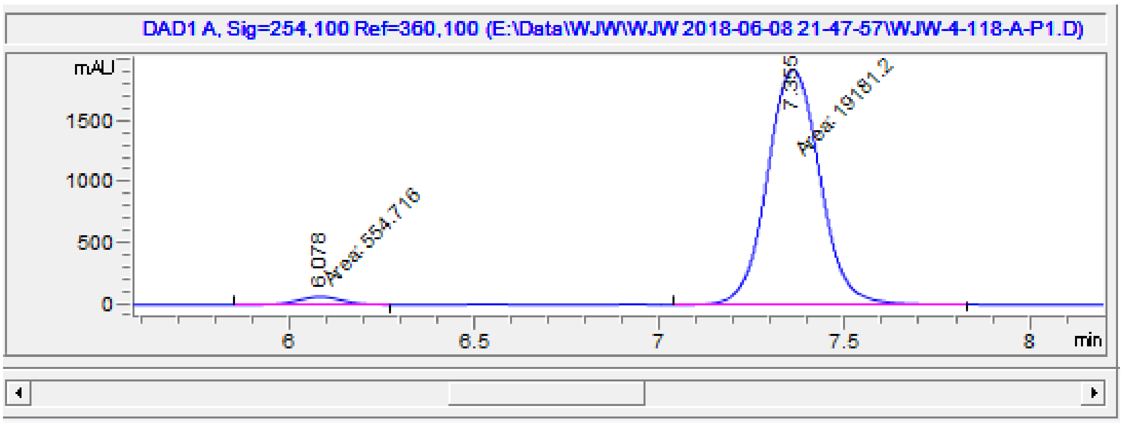

#	Time	Type	Area	Height	Width	Area%	Symmetry
1	6.078	MM	554.7	64.9	0.1424	2.811	0.981
2	7.355	MM	19181.2	1932.6	0.1654	97.189	0.907

(*S*)-4-((*1R*,*3S*)-2-acetyl-1-(4-chlorophenyl)-4-phenylbuta-2,3-dien-1-yl)-2-(3,5-dimethoxyphenyl)-4-ethyloxazol-5(4H)-one **(4n)**

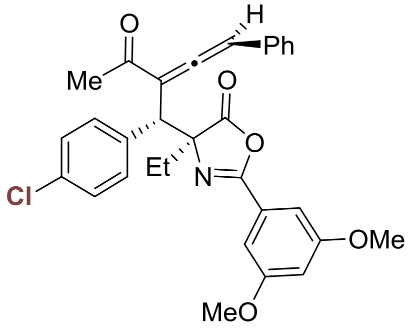

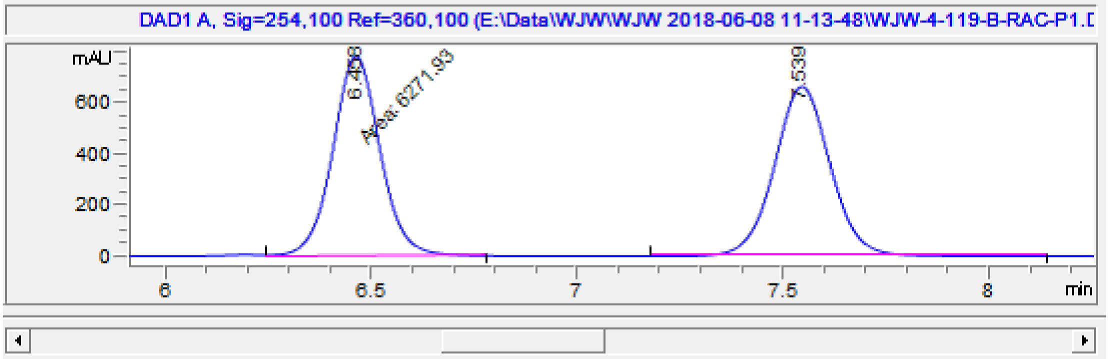

#	Time	Type	Area	Height	Width	Area%	Symmetry
1	6.458	MF	6271.9	778.7	0.1342	50.068	0.943
2	7.539	BB	6255	660.8	0.1454	49.932	0.954

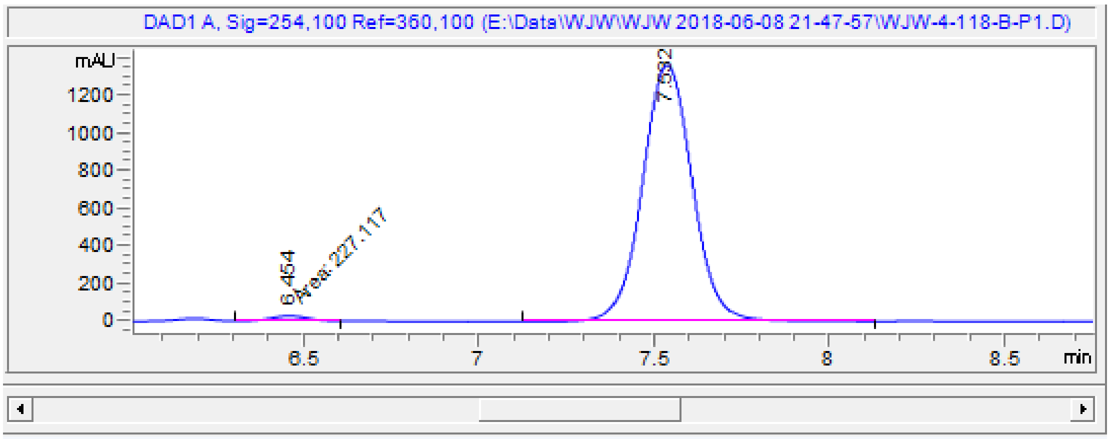

#	Time	Type	Area	Height	Width	Area%	Symmetry
1	6.454	MF	227.1	28.2	0.1341	1.649	0.992
2	7.532	BB	13544.7	1373.8	0.1525	98.351	0.959

(*S*)-4-((*1R*,*3S*)-2-acetyl-1-(4-methoxyphenyl)-4-phenylbuta-2,3-dien-1-yl)-2-(3,5-dimethoxyphenyl)-4-ethyloxazol-5(4H)-one **(4o)**

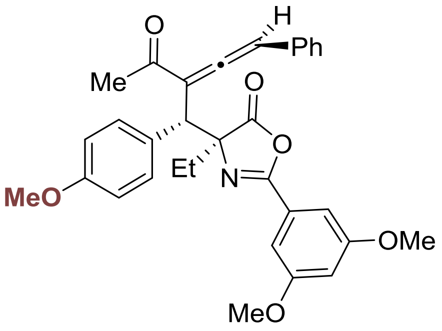

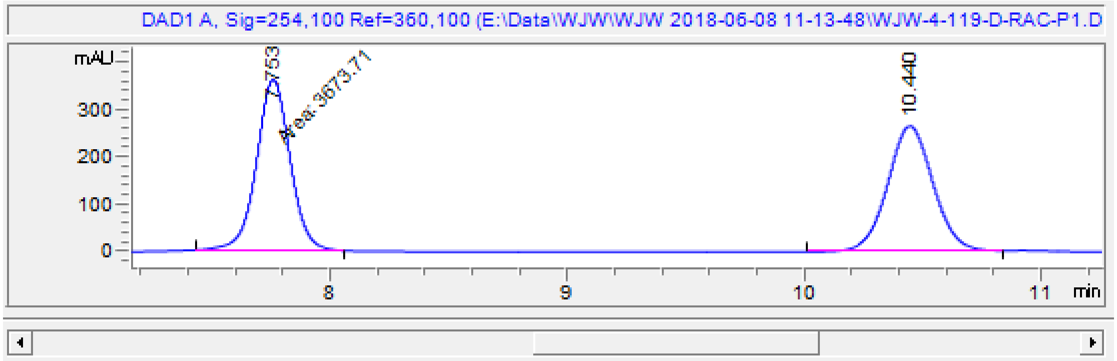

#	Time	Type	Area	Height	Width	Area%	Symmetry
1	7.753	MF	3673.7	363.4	0.1685	50.956	0.985
2	10.44	BB	3535.9	264.2	0.2076	49.044	0.948

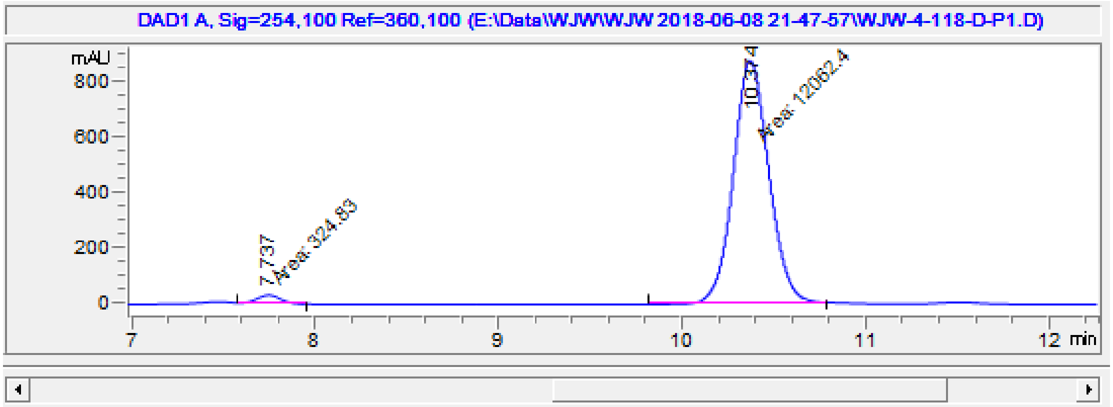

#	Time	Type	Area	Height	Width	Area%	Symmetry
1	7.737	MF	324.8	32.8	0.1652	2.622	0.985
2	10.374	MM	12062.4	880.9	0.2282	97.378	0.874

(*S*)-4-((*1R*,*3S*)-2-acetyl-1-(naphthalen-2-yl)-4-phenylbuta-2,3-dien-1-yl)-2-(3,5-dimethoxyphenyl)-4-ethyloxazol-5(4H)-one **(4p)**

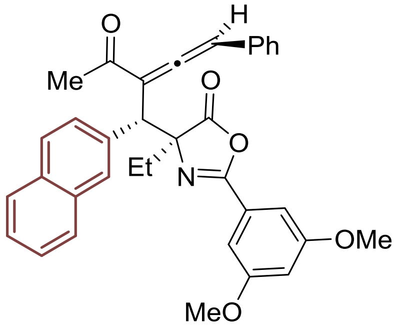

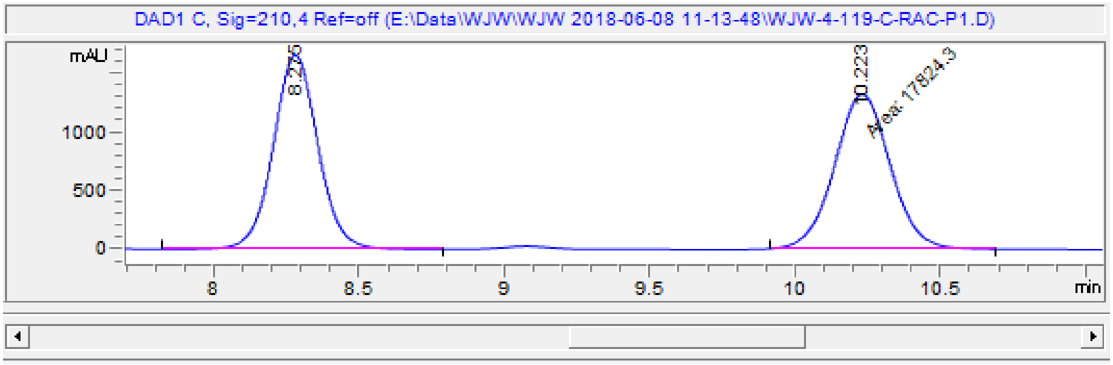

#	Time	Type	Area	Height	Width	Area%	Symmetry
1	8.275	VB	17777	1657.5	0.1661	49.934	0.959
2	10.223	FM	17824.3	1325.4	0.2241	50.066	0.966

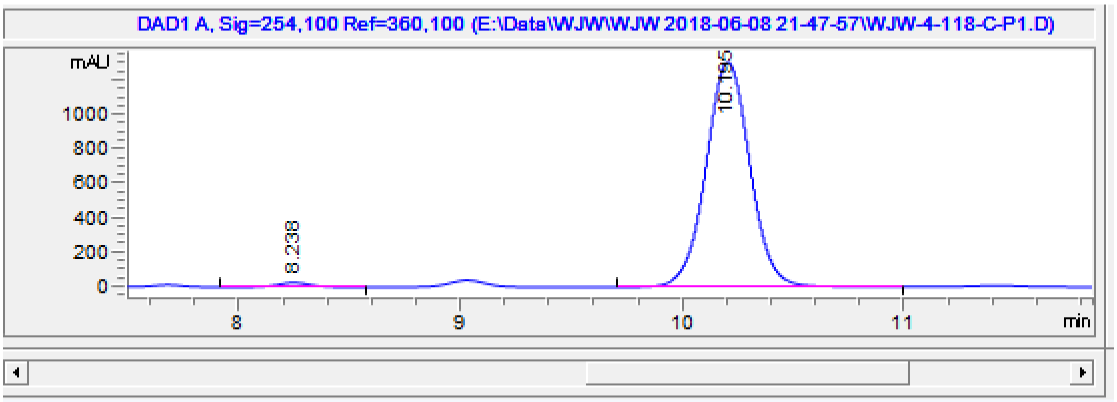

#	Time	Type	Area	Height	Width	Area%	Symmetry
1	8.238	BB	276.1	26.2	0.1621	1.507	0.968
2	10.195	BB	18051.1	1303.1	0.1911	98.493	0.946

(*S*)-4-((*1R*,*3S*)-2-acetyl-1-cyclohexyl-4-phenylbuta-2,3-dien-1-yl)-2-(3,5-dimethoxyphenyl)-4-ethyloxazol-5(4H)-one **(4q)**

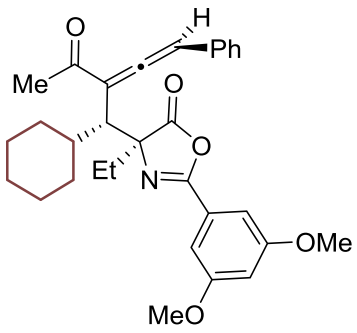

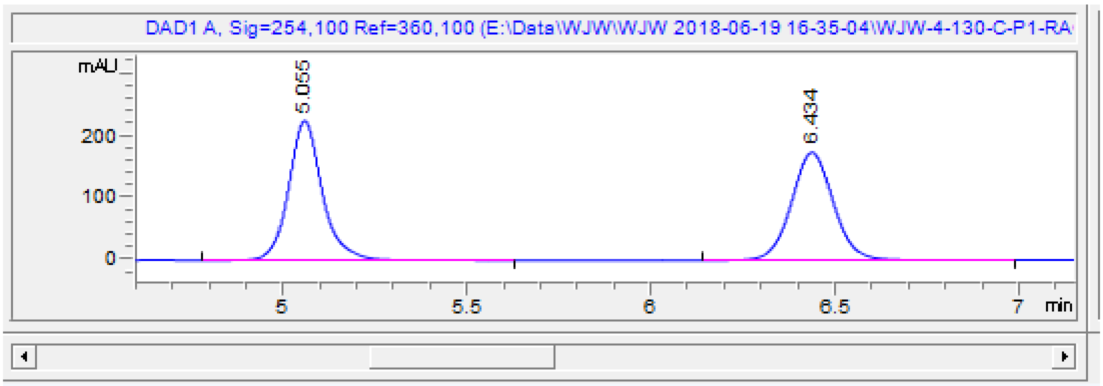

#	Time	Type	Area	Height	Width	Area%	Symmetry
1	5.055	BB	1422.3	223.5	0.0958	51.065	0.856
2	6.434	BB	1362.9	172.7	0.1211	48.935	0.944

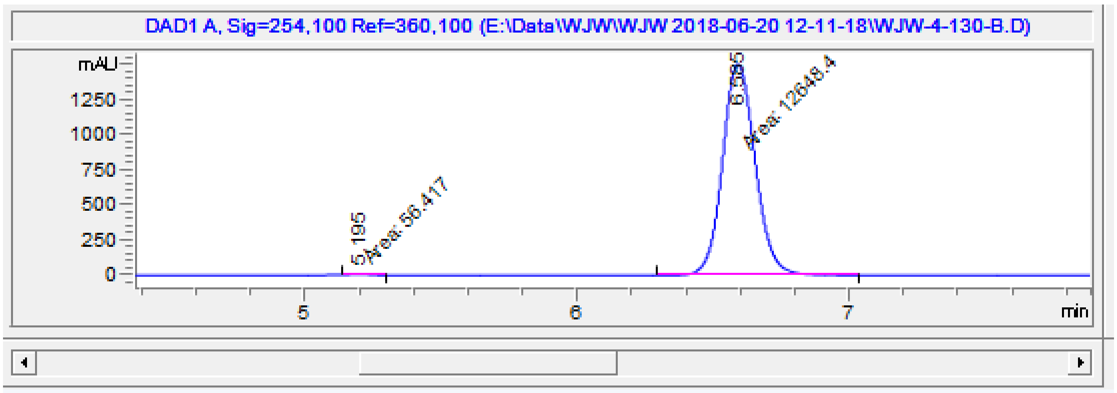

#	Time	Type	Area	Height	Width	Area%	Symmetry
1	5.195	FM	56.4	9	0.1046	0.444	0.843
2	6.585	MM	12648.4	1506.3	0.1399	99.556	0.856

(*S*)-4-((*1R*,*3S*)-2-acetyl-1,4-diphenylbuta-2,3-dien-1-yl)-4-butyl-2-(3,5-dimethoxyphenyl)oxazol-5(4H)-one **(4v)**

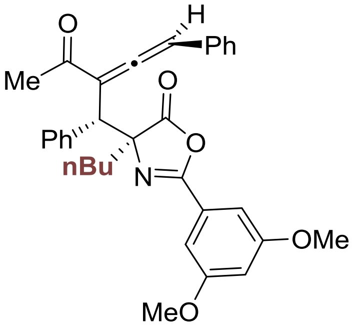

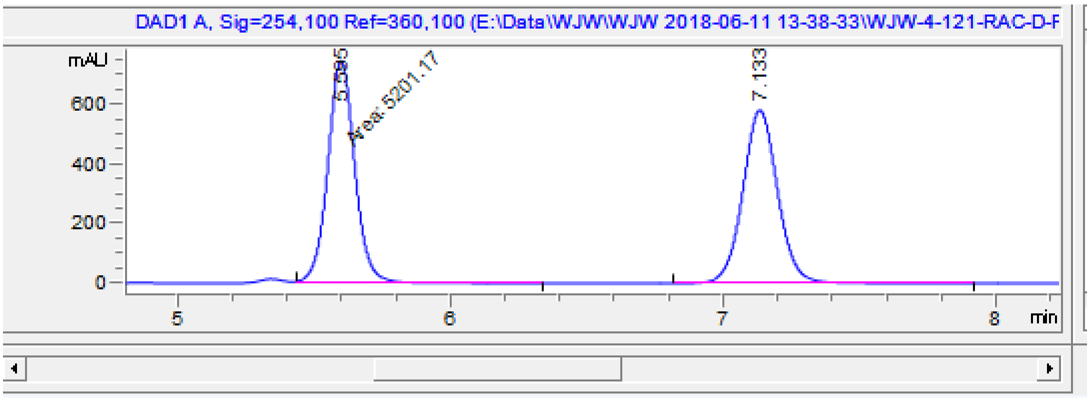

#	Time	Type	Area	Height	Width	Area%	Symmetry
1	5.595	FM	5201.2	749.6	0.1156	50.007	0.922
2	7.133	BB	5199.7	582.6	0.1376	49.993	0.928

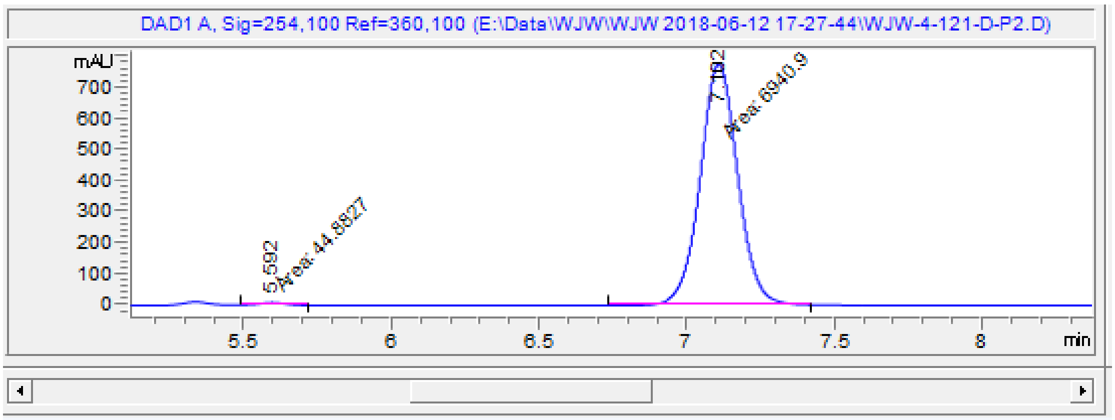

#	Time	Type	Area	Height	Width	Area%	Symmetry
1	5.592	MM	44.9	7.6	0.0986	0.642	0.837
2	7.102	MF	6940.9	778.6	0.1486	99.358	0.96

(*S*)-4-((*1R*,*3S*)-2-acetyl-1,4-diphenylbuta-2,3-dien-1-yl)-4-benzyl-2-(3,5-dimethoxyphenyl)oxazol-5(4H)-one **(4w)**

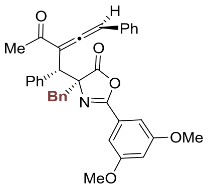

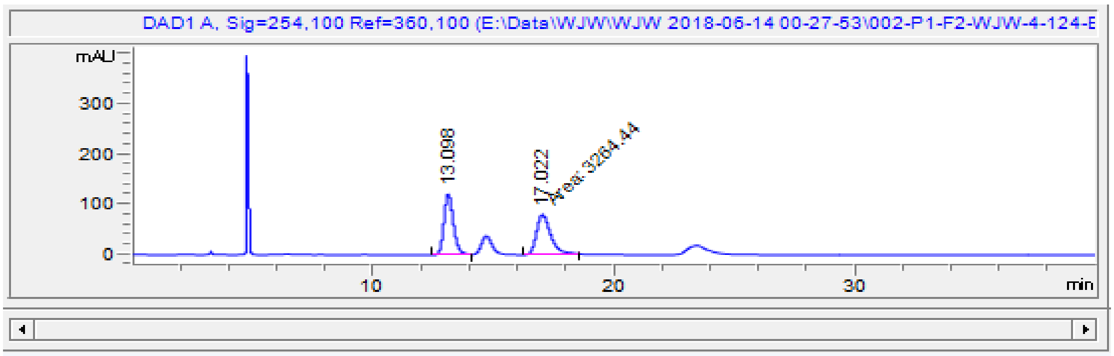

#	Time	Type	Area	Height	Width	Area%	Symmetry
1	13.098	BB	3237	120.5	0.395	49.789	0.791
2	17.022	MM	3264.4	79.3	0.6865	50.211	0.688

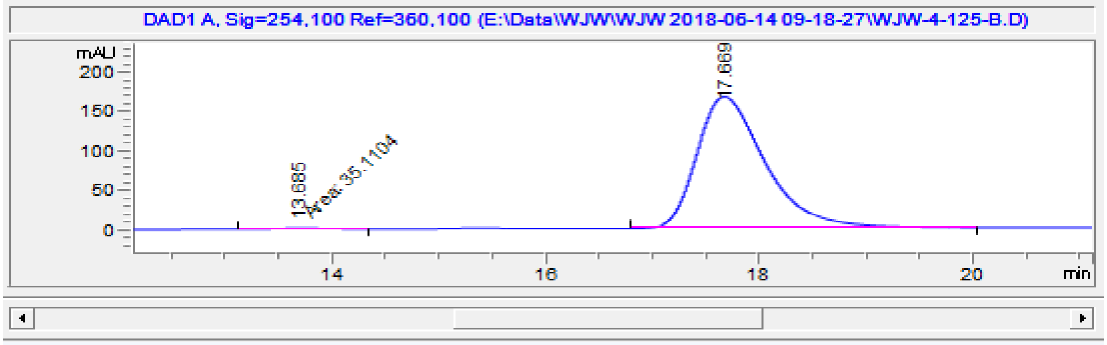

#	Time	Type	Area	Height	Width	Area%	Symmetry
1	13.685	MM	35.1	1.2	0.4826	0.469	0.883
2	17.669	BB	7451.3	167	0.6016	99.531	0.627

The minor diastereomer:

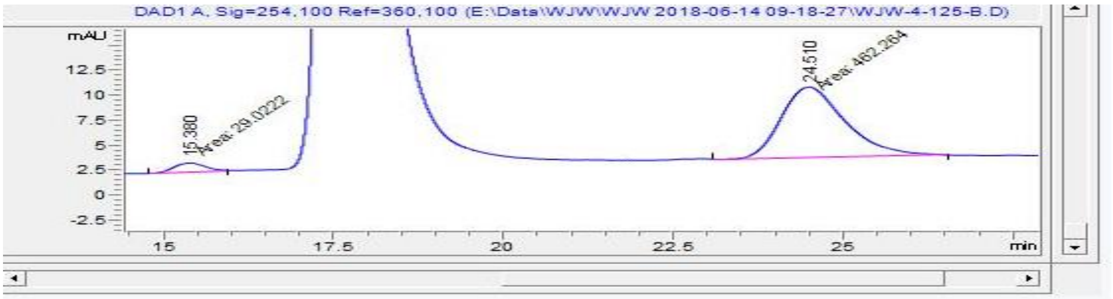

#	Time	Type	Area	Height	Width	Area%	Symmetry
1	15.38	MM	29	9.1E-1	0.5311	5.907	1.116
2	24.51	MM	462.3	7.1	1.0925	94.093	0.794

(*S*)-4-((*1R*,*3S*)-2-acetyl-1,4-diphenylbuta-2,3-dien-1-yl)-2-(3,5-dimethoxyphenyl)-4-(2-(methylthio)ethyl)oxazol-5(4H)-one **(4x)**

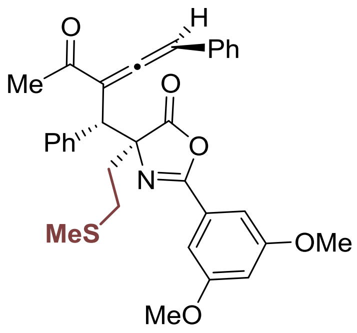

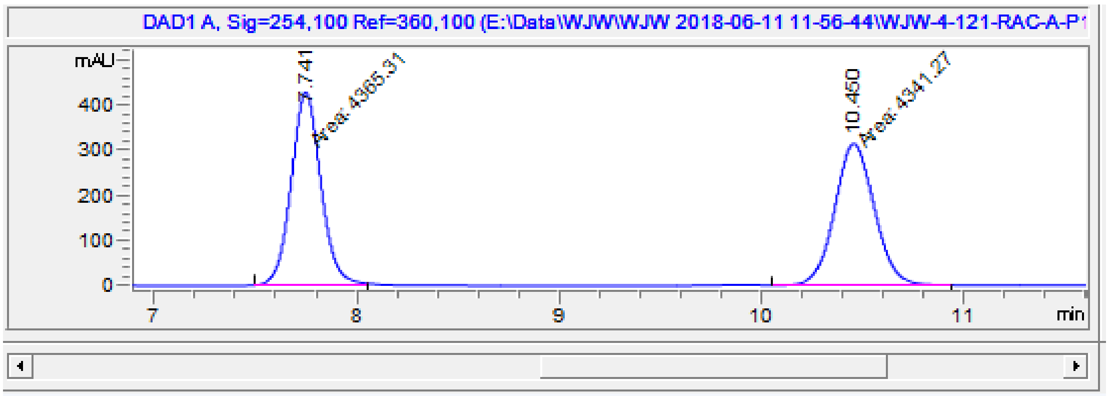

#	Time	Type	Area	Height	Width	Area%	Symmetry
1	7.741	FM	4365.3	426.3	0.1707	50.138	0.904
2	10.45	MM	4341.3	313.5	0.2308	49.862	0.913

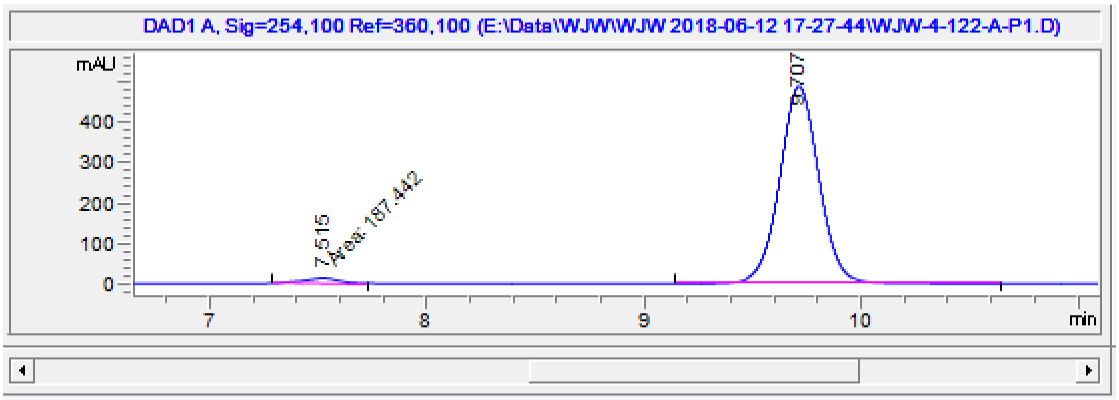

#	Time	Type	Area	Height	Width	Area%	Symmetry
1	7.515	MM	187.4	14.2	0.2198	2.984	0.918
2	9.707	BB	6094	482.8	0.1948	97.016	0.947

tert-butyl-(4-((*S*)-4-((*1R*,*3S*)-2-acetyl-1,4-diphenylbuta-2,3-dien-1-yl)-2-(3,5-dimethoxyphenyl)-5-oxo-4,5-dihydrooxazol-4-yl)butyl)carbamate **(4y)**

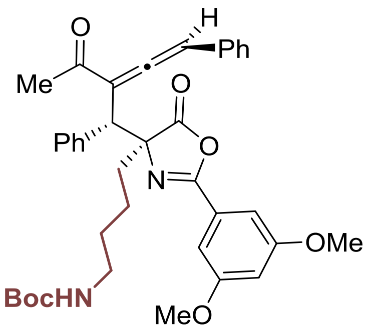

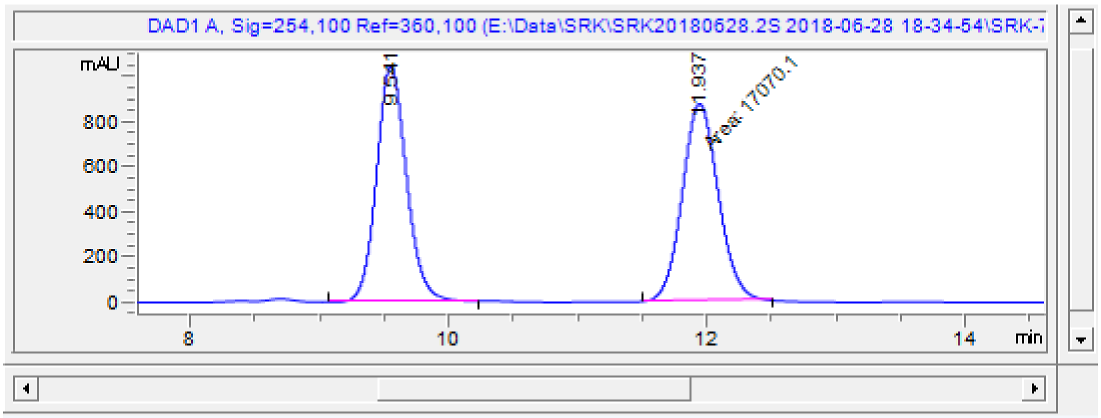

#	Time	Type	Area	Height	Width	Area%	Symmetry
1	9.541	BB	16959.9	1045.2	0.2488	49.838	0.861
2	11.937	MM	17070.1	877.4	0.3243	50.162	0.888

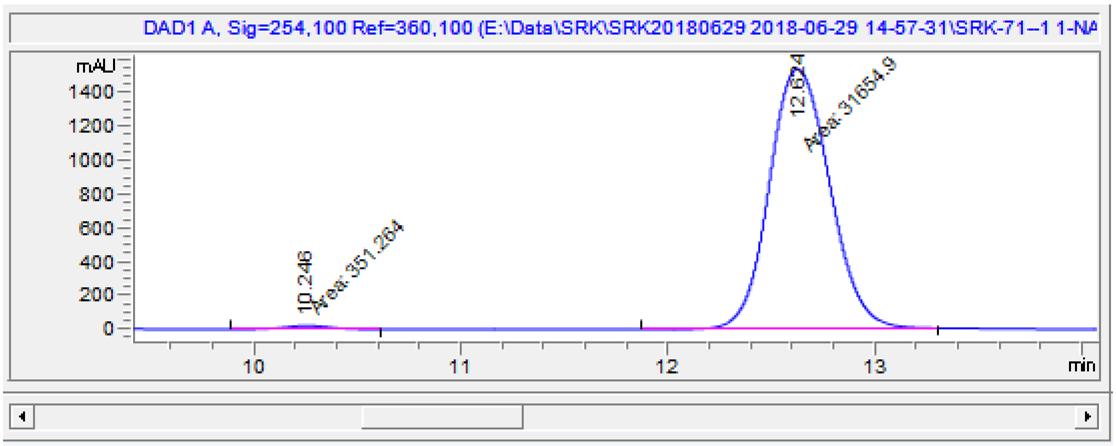

#	Time	Type	Area	Height	Width	Area%	Symmetry
1	10.246	MM	351.3	21.9	0.2675	1.097	0.978
2	12.624	MF	31654.9	1544.8	0.3415	98.903	0.835

3-benzyl-5-phenylpenta-3,4-dien-2-one**(6a)**

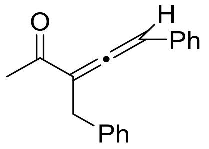

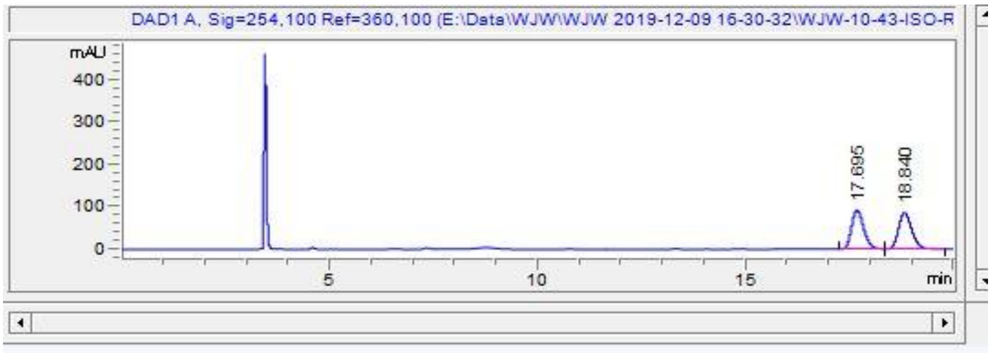

#	Time	Type	Area	Height	Width	Area%	Symmetry
1	17.695	BB	1899.9	93	0.3113	49.947	0.76
2	18.84	BB	1903.9	86.5	0.3288	50.053	0.779

Using (*S*)-**B2** as catalyst

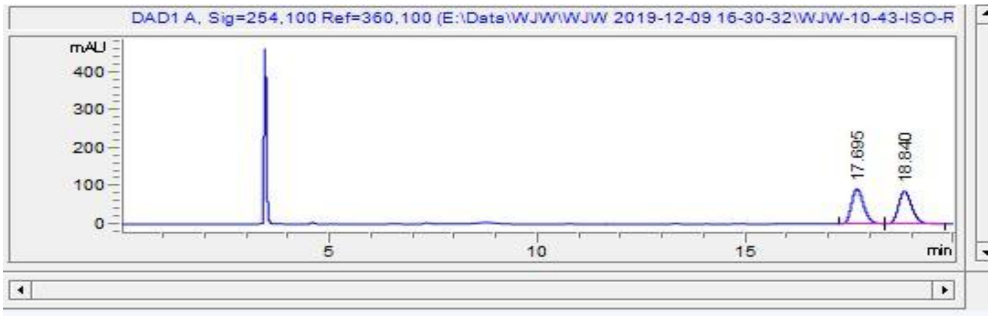

#	Time	Type	Area	Height	Width	Area%	Symmetry
1	17.695	BB	1899.9	93	0.3113	49.947	0.76
2	18.84	BB	1903.9	86.5	0.3288	50.053	0.779

Using (*R*)-**C1** as catalyst

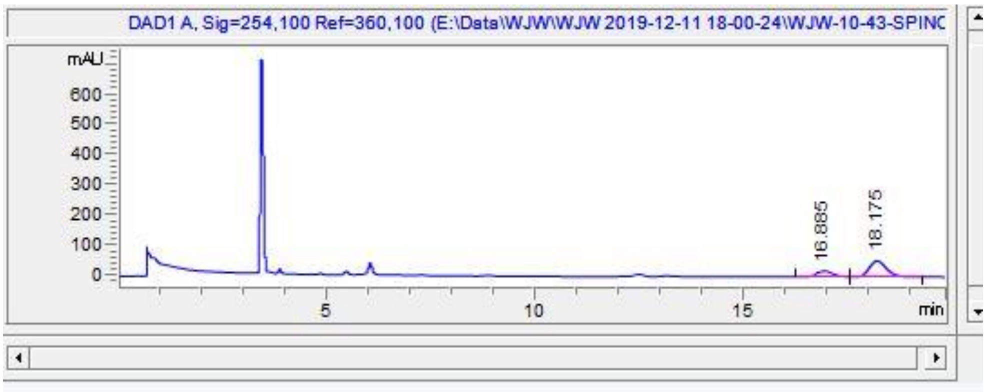

#	Time	Type	Area	Height	Width	Area%	Symmetry
1	16.885	BB	515.7	19.6	0.3708	25.708	0.832
2	18.175	BB	1490.3	52.6	0.4267	74.292	0.811

(*S*)-2-(3,5-dimethoxyphenyl)-4-ethyl-4-((*R*)-(2-methyl-5-phenylfuran-3-yl)(phenyl)methyl)oxazol-5(4H)-one **(7a)**

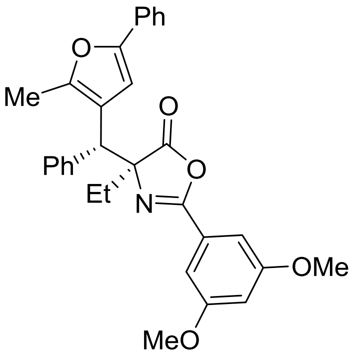

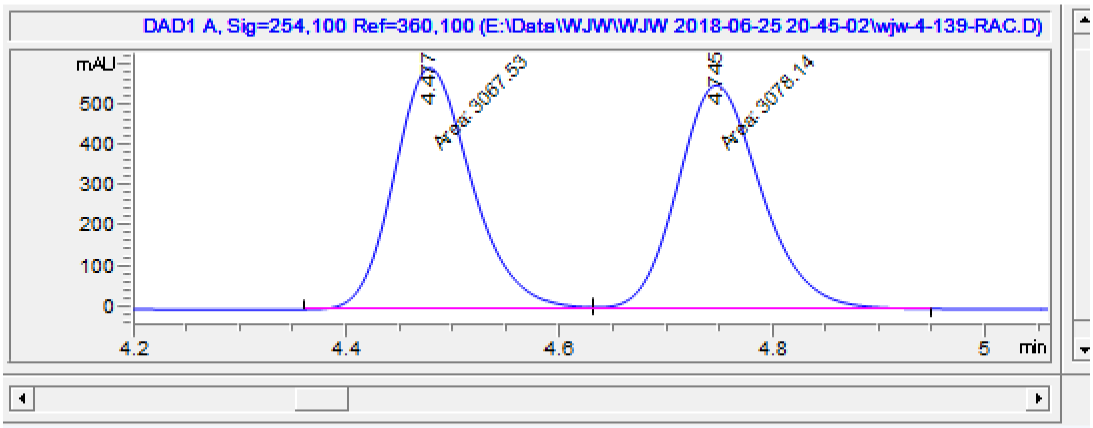

#	Time	Type	Area	Height	Width	Area%	Symmetry
1	4.477	FM	3067.5	601.9	0.0849	49.914	0.828
2	4.745	MF	3078.1	558.7	0.0918	50.086	0.845

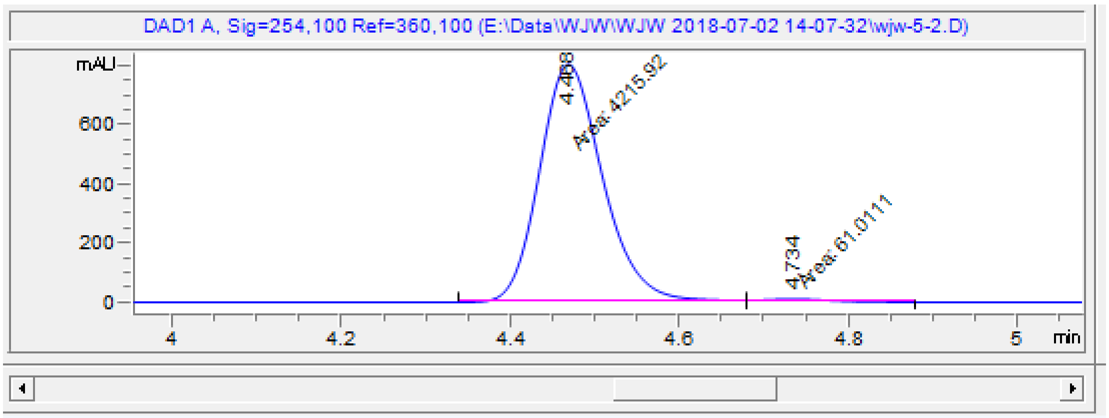

#	Time	Type	Area	Height	Width	Area%	Symmetry
1	4.468	MF	4215.9	802.1	0.0876	98.573	0
2	4.734	FM	61	10.6	0.096	1.427	0.808

Methyl-(*S*)-2-(3,5-dimethoxybenzamido)-2-((*R*)-(2-methyl-5-phenylfuran-3-yl)(phenyl)methyl)butanoate **(8a)**

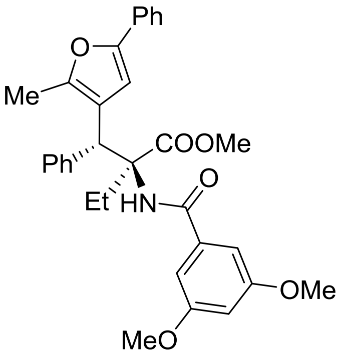

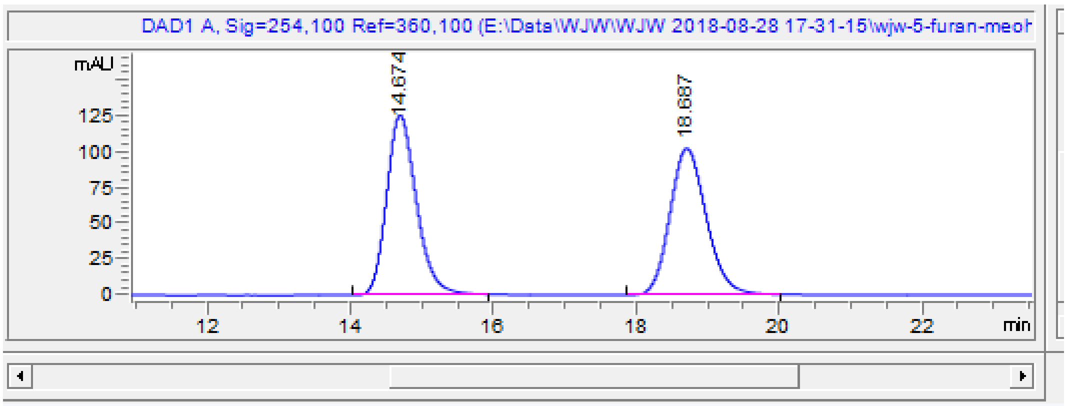

#	Time	Type	Area	Height	Width	Area%	Symmetry
1	14.674	BB	3612.3	124.5	0.3945	50.163	0.778
2	18.687	BB	3588.8	101.6	0.4295	49.837	0.836

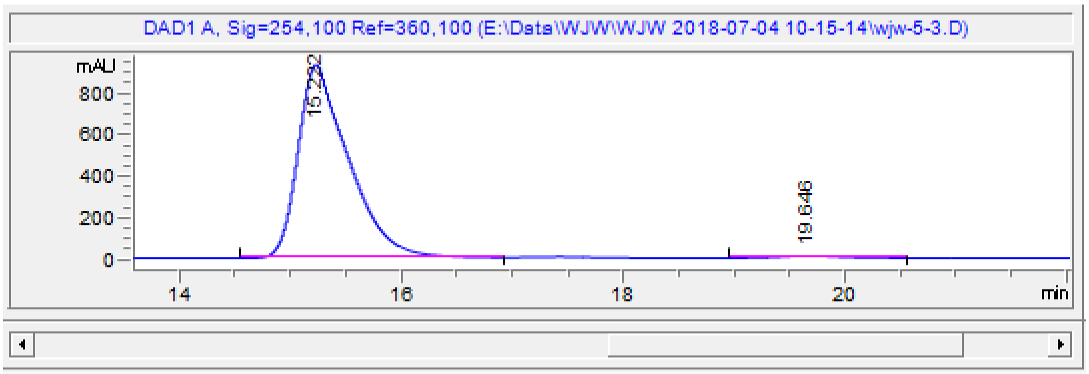

#	Time	Type	Area	Height	Width	Area%	Symmetry
1	15.222	BB	29261.3	925.8	0.3707	98.782	0.54
2	19.646	BB	360.8	9.8	0.4314	1.218	0.889

N-((*3S*,*4R*,*6R*)-3-ethyl-6-methyl-2-oxo-4-phenyl-5-((*R*)-2-phenylvinylidene)tetrahydro-2H-pyran-3-yl)-3,5-dimethoxybenzamide **(9a)**

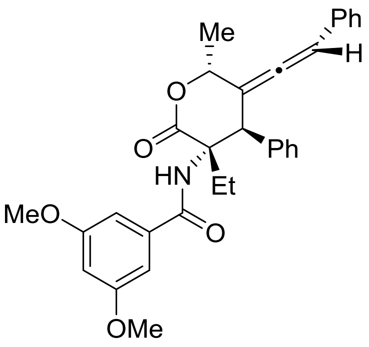

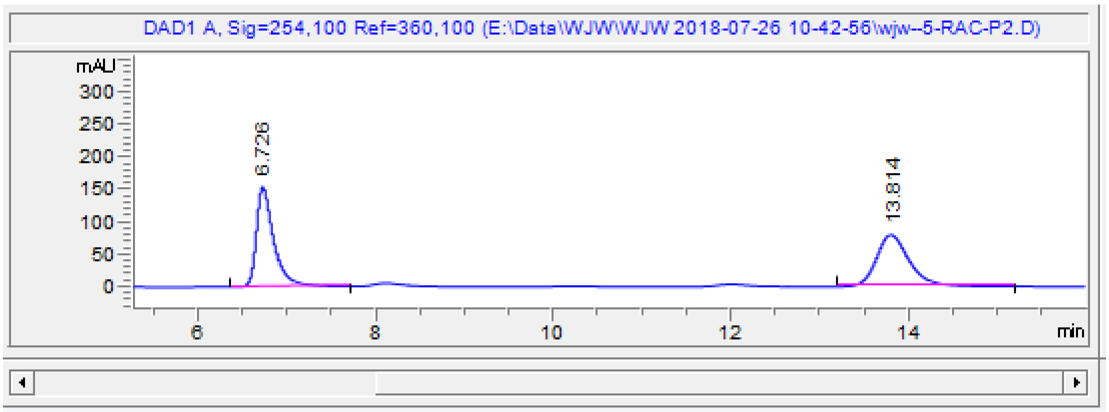

#	Time	Type	Area	Height	Width	Area%	Symmetry
1	6.726	BB	2110.7	155.6	0.2007	50.028	0.54
2	13.814	BB	2108.4	80.9	0.386	49.972	0.739

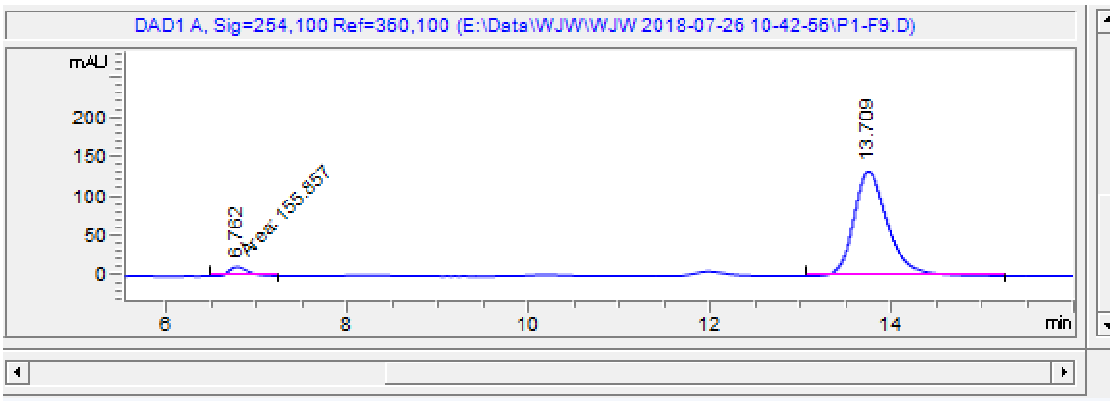

#	Time	Type	Area	Height	Width	Area%	Symmetry
1	6.762	MM	155.9	11.1	0.2335	4.340	0.667
2	13.709	BB	3435.7	132.7	0.3824	95.660	0.727

3,5-dimethoxy-N-((*1R*,*2S*)-1-((*2R*,*5S*)-2-methyl-5-phenyl-2,5-dihydrofuran-3-yl)-1-phenyl-2-(propylcarbamoyl)butan-2-yl)benzamide **(11a)**

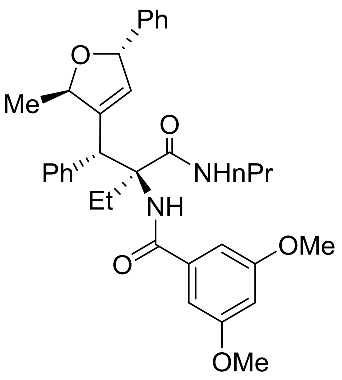

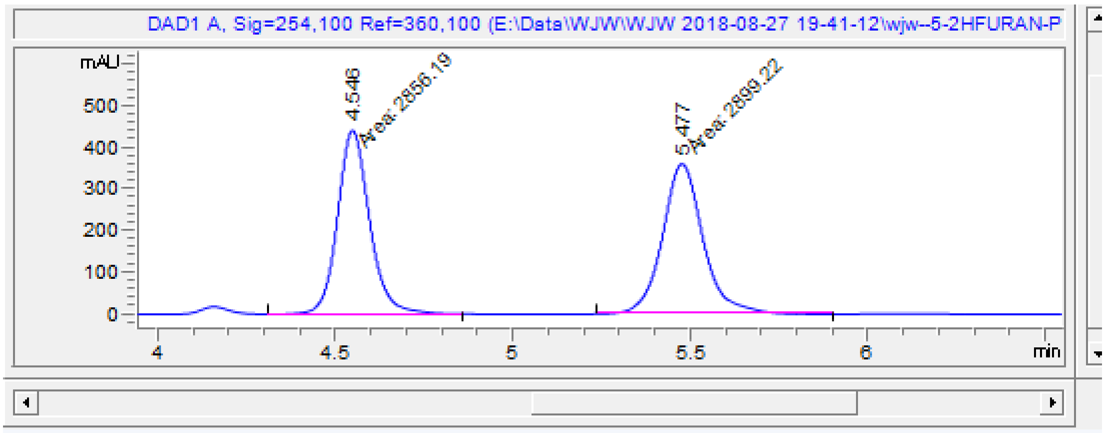

#	Time	Type	Area	Height	Width	Area%	Symmetry
1	4.546	MM	2856.2	442.4	0.1076	49.626	0.851
2	5.477	FM	2899.2	359.9	0.1343	50.374	0.912

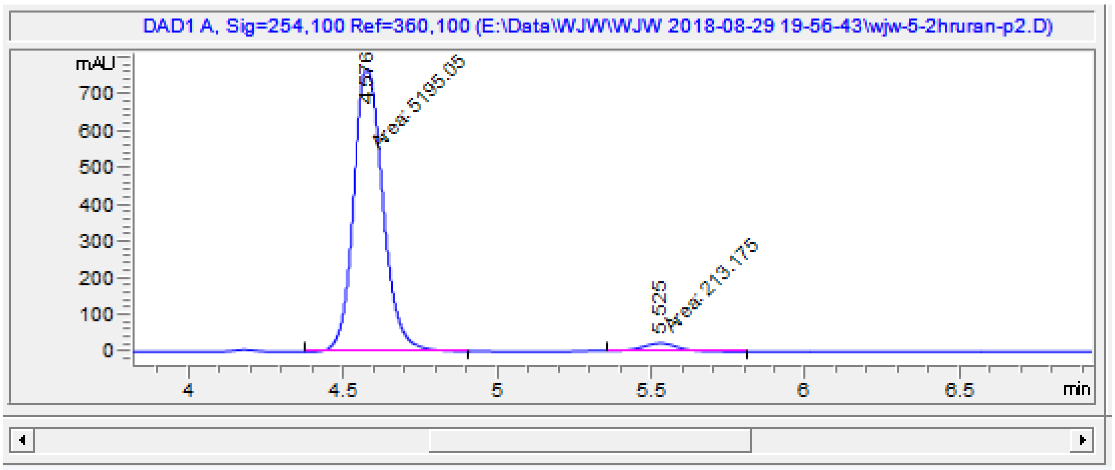

#	Time	Type	Area	Height	Width	Area%	Symmetry
1	4.576	MM	5195	772.2	0.1121	96.058	0.821
2	5.525	FM	213.2	23.3	0.1523	3.942	1.015

N-((*1S*,*4R*,*6R*,*10bR*)-1-ethyl-5-iodo-4-methyl-2-oxo-6-phenyl-1,4,6,10b-tetrahydro-2H-benzo[f]isochromen-1-yl)-3,5-dimethoxybenzamide **(12a)**

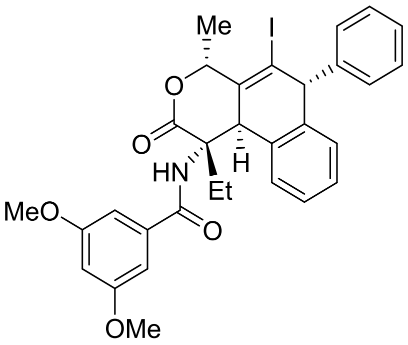

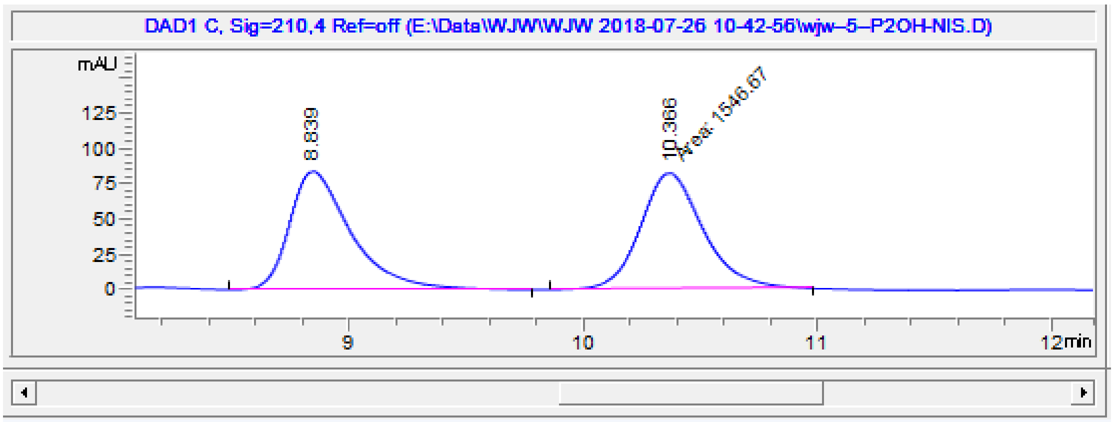

#	Time	Type	Area	Height	Width	Area%	Symmetry
1	8.839	BB	1552.7	84.2	0.2642	50.097	0.566
2	10.366	MM	1546.7	82.4	0.3128	49.903	0.826

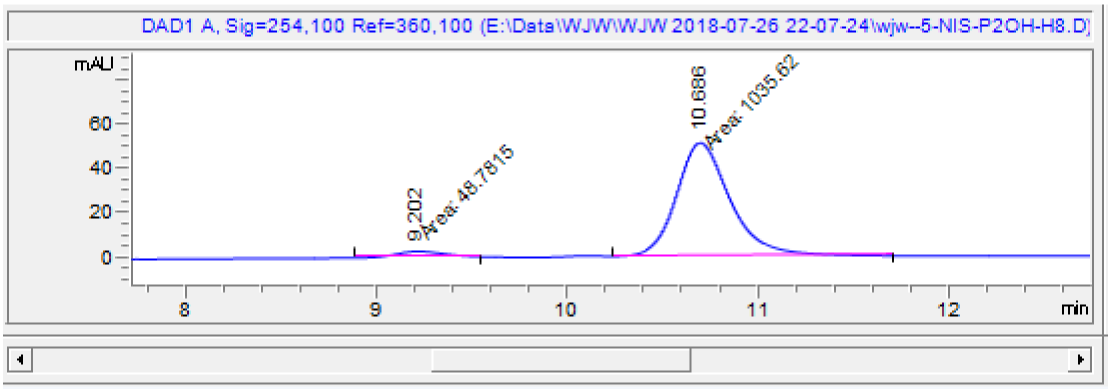

#	Time	Type	Area	Height	Width	Area%	Symmetry
1	9.202	MM	48.8	2.8	0.2929	4.498	0.814
2	10.686	MM	1035.6	52.2	0.3308	95.502	0.77

N-((3*S*,4*S*,6*S*)-3-ethyl-6-methyl-2-oxo-4-phenyl-5-((*S*)-2-phenylvinylidene)tetrahydro-2H-pyran-3-yl)-3,5-dimethoxybenzamide **(13a)**

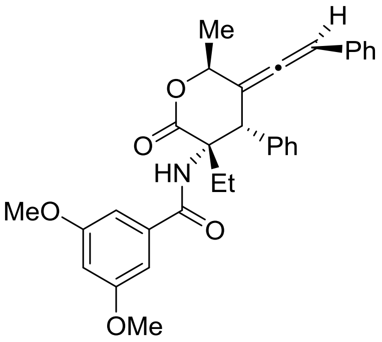

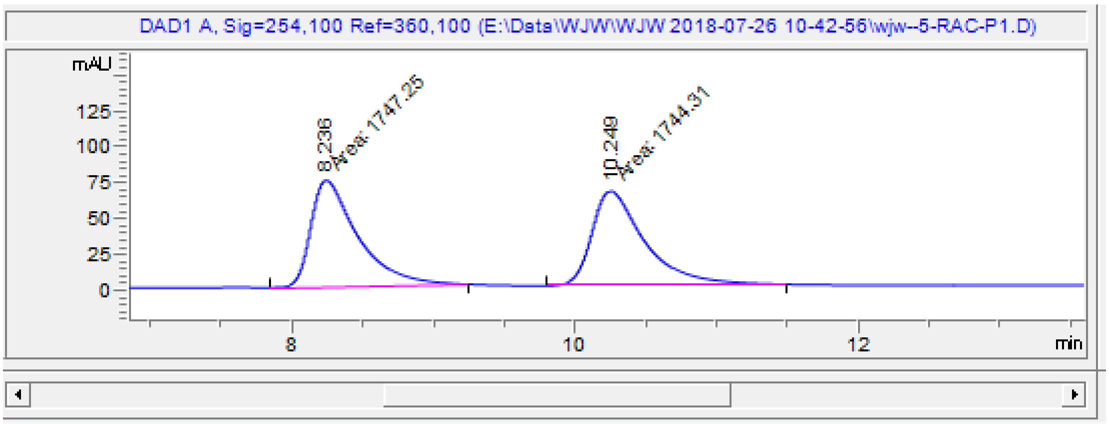

#	Time	Type	Area	Height	Width	Area%	Symmetry
1	8.236	MM	1747.3	74.3	0.3918	50.042	0.475
2	10.249	MM	1744.3	65.8	0.4419	49.958	0.527

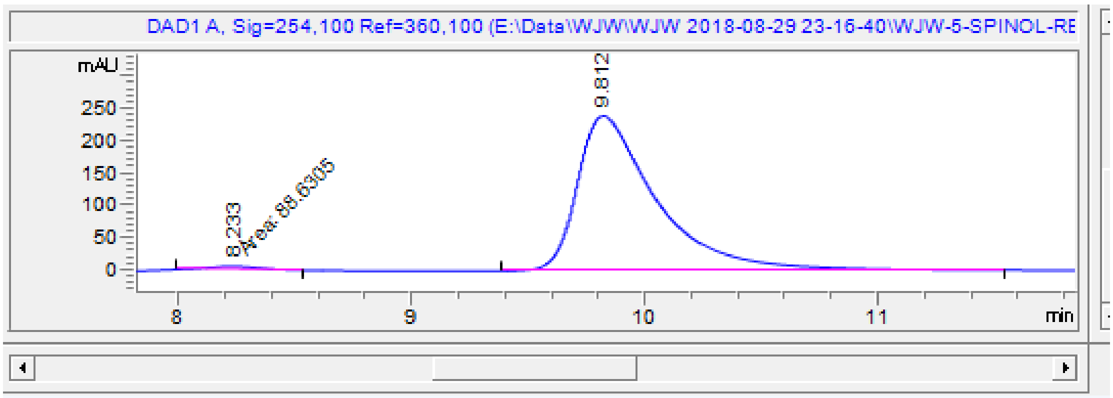

#	Time	Type	Area	Height	Width	Area%	Symmetry
1	8.233	MM	88.6	5.6	0.265	1.520	0.721
2	9.812	BB	5743	242.5	0.3348	98.480	0.449

3,5-dimethoxy-*N*-((*1R*,*2S*)-1-((*2S*,*5R*)-2-methyl-5-phenyl-2,5-dihydrofuran-3-yl)-1-phenyl-2-(propylcarbamoyl)butan-2-yl)benzamide **(15a)**

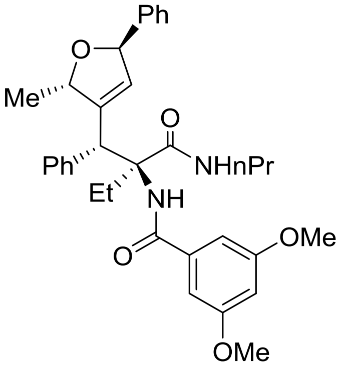

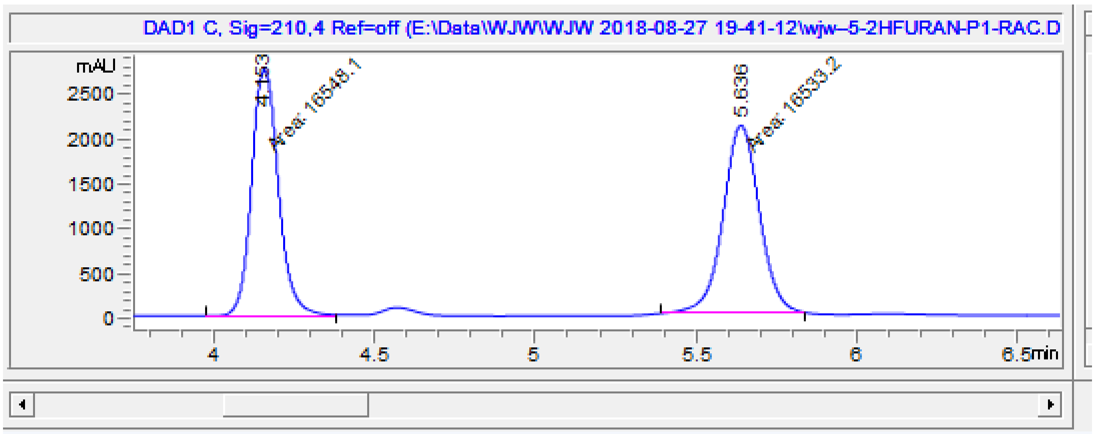

#	Time	Type	Area	Height	Width	Area%	Symmetry
1	4.153	MM	16548.1	2754.4	0.1001	50.023	0.861
2	5.636	MM	16533.2	2081.7	0.1324	49.977	0.981

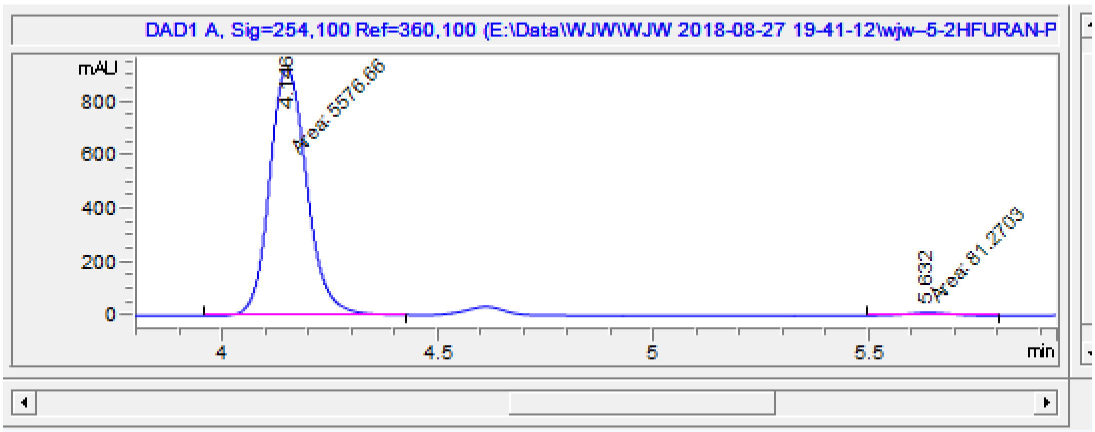

#	Time	Type	Area	Height	Width	Area%	Symmetry
1	4.146	MF	5576.7	919.9	0.101	98.564	0.818
2	5.632	MM	81.3	11.1	0.122	1.436	0.899

(*1R,5S,8S,9S*)-3-(3,5-dimethoxyphenyl)-5-ethyl-1-((*Z*)-1-iodo-2-phenylvinyl)-8-methyl-9-phenyl-2,7-dioxa-4-azabicyclo[3.3.1]non-3-en-6-one (**16a**)

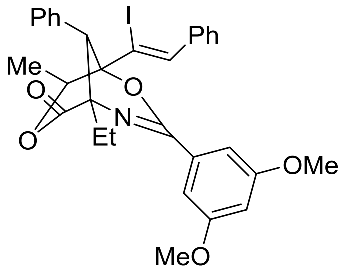

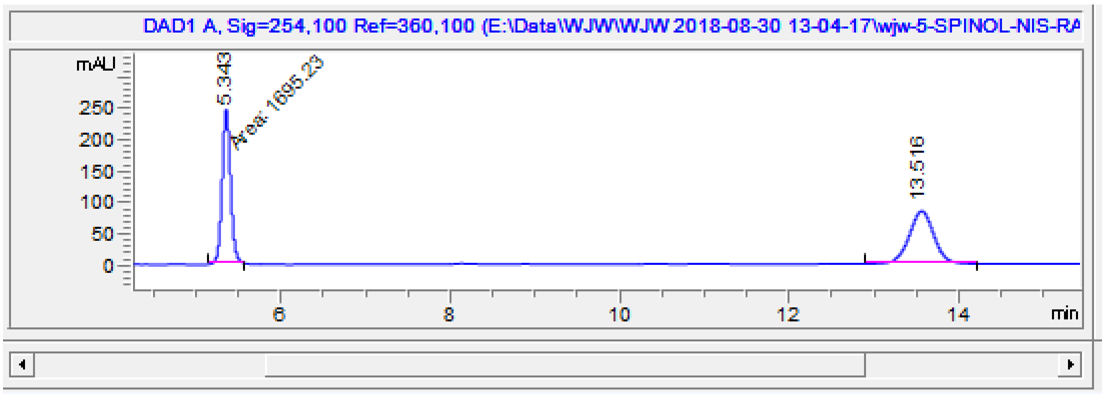

#	Time	Type	Area	Height	Width	Area%	Symmetry
1	5.343	MM	1695.2	245.9	0.1149	49.850	0.936
2	13.516	BB	1705.4	84.3	0.2904	50.150	1.017

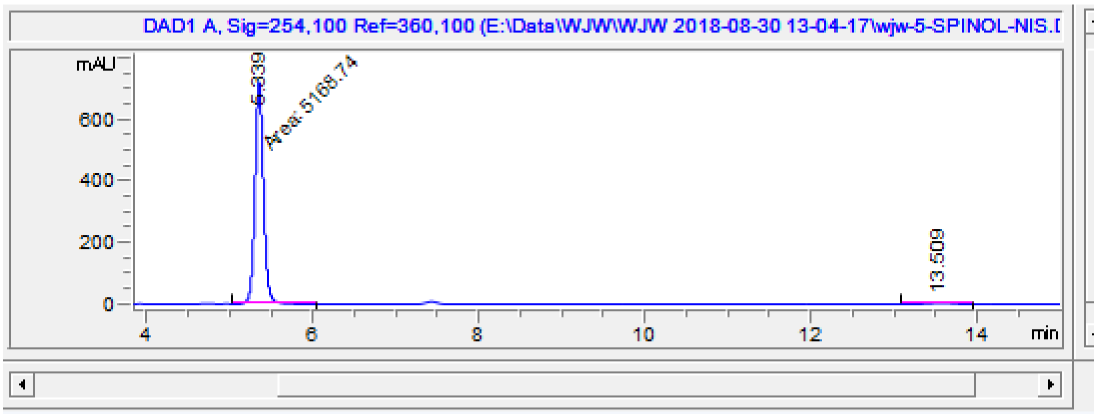

#	Time	Type	Area	Height	Width	Area%	Symmetry
1	5.339	FM	5168.7	727.2	0.1185	98.497	0.912
2	13.509	BB	78.9	3.9	0.2349	1.503	0.987

NMR Spectrums:

4-hydroxy-2,6-bis(4-methylnaphthalen-1-yl)-8,9,10,11,12,13,14,15-octahydrodinaphtho[2,1-d:1',2'-f][1,3,2]dioxaphosphepine 4-oxide **(*S*)-B2**

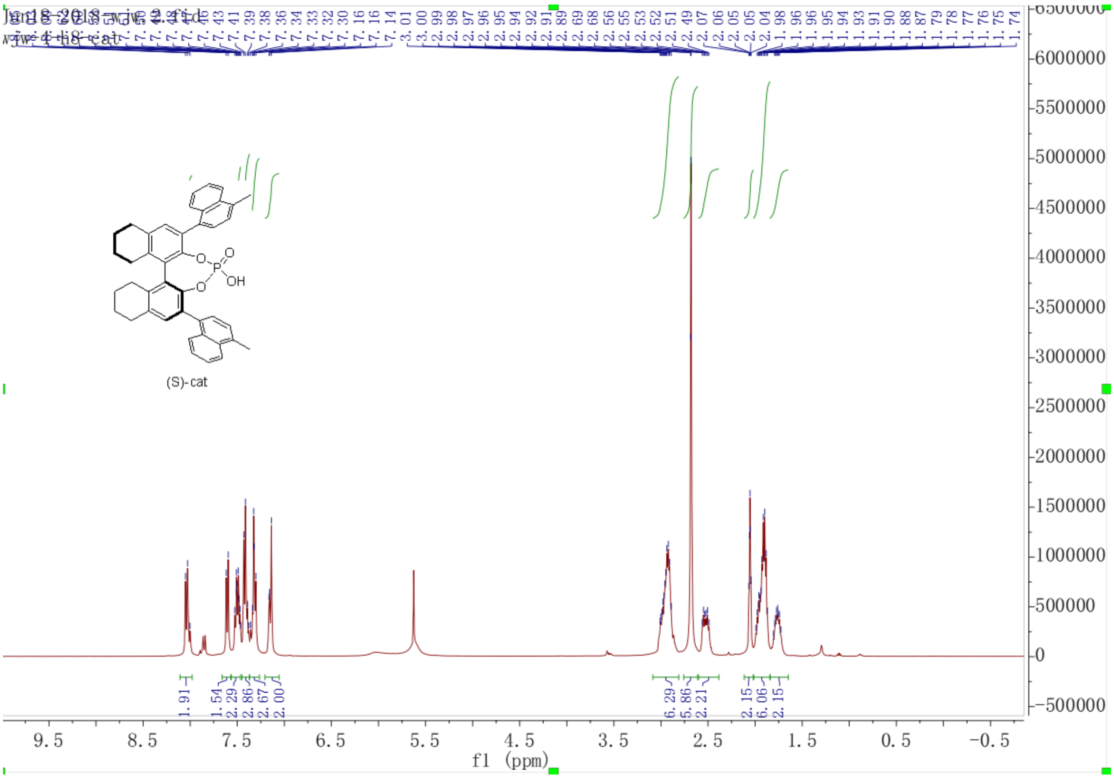

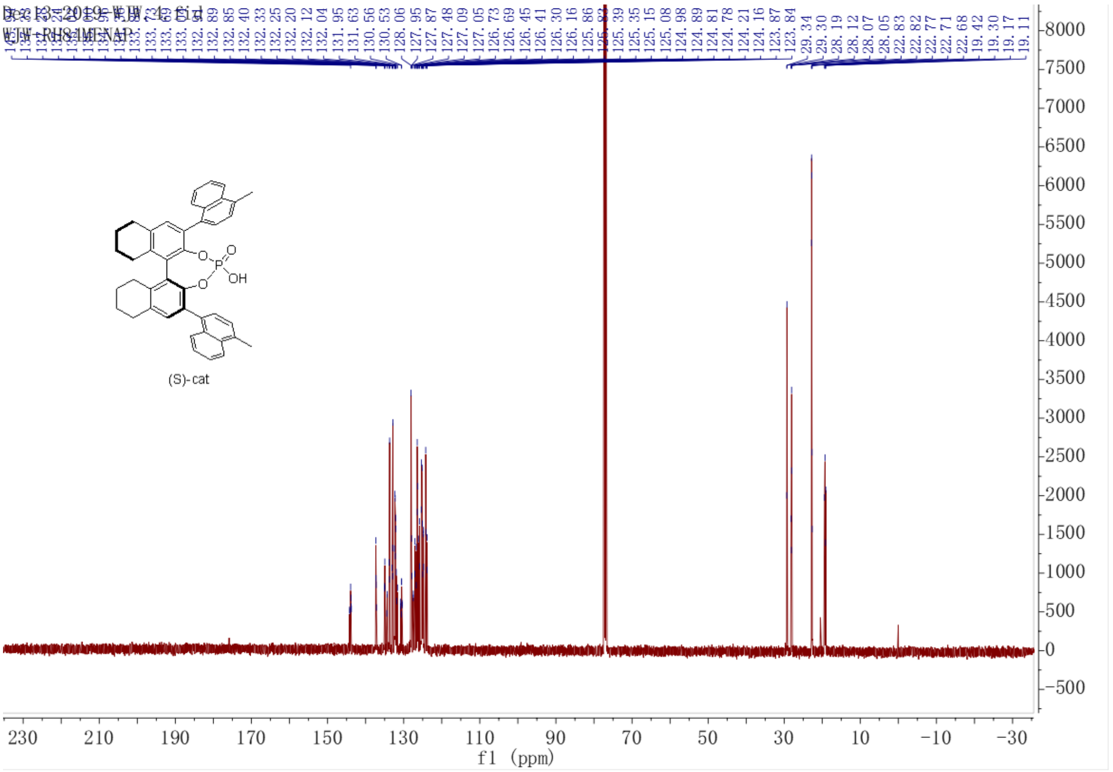

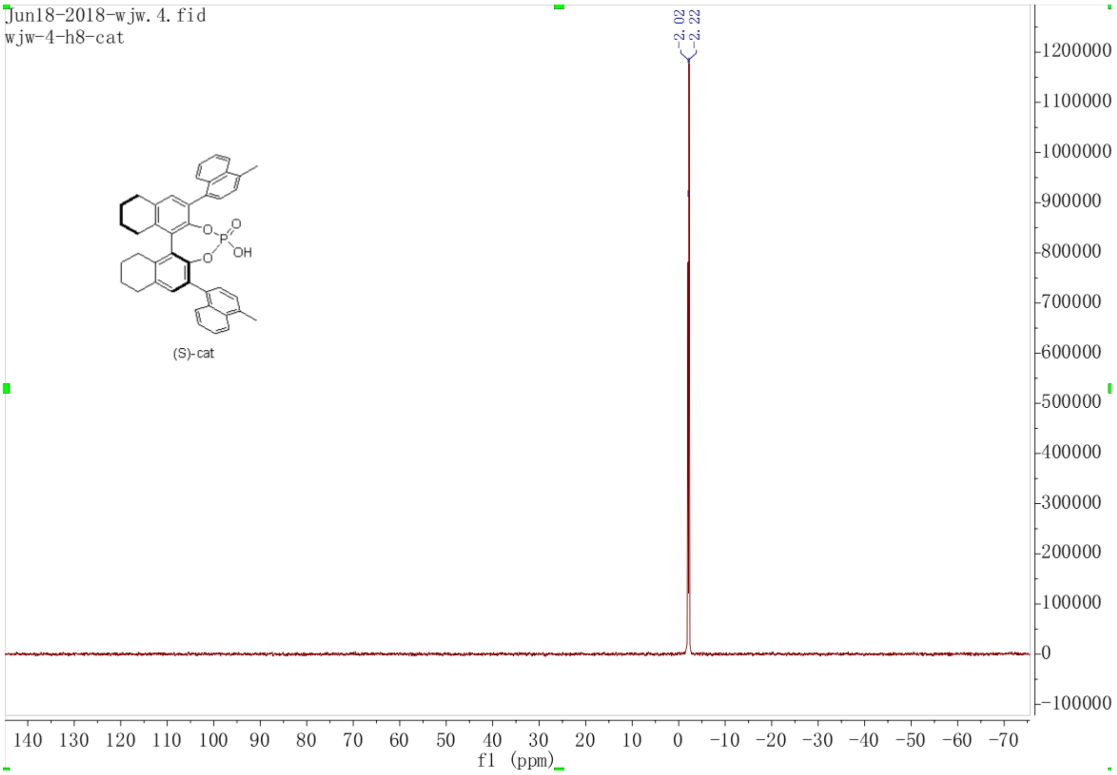
Jun18-2018-wjw.4.fid
wjw-4-h8-cat
-2.02
-2.22
(S)-cat
OH
1200000
1100000
1000000
900000
800000
700000
600000
500000
400000
300000
200000
100000
0
-100000
140 130 120 110 100 90 80 70 60 50 40 30 20 10 0 -10 -20 -30 -40 -50 -60 -70
f1 (ppm)

(*E*)-3-benzylidene-5-(4-chlorophenyl)pent-4-yn-2-one (**1c**)

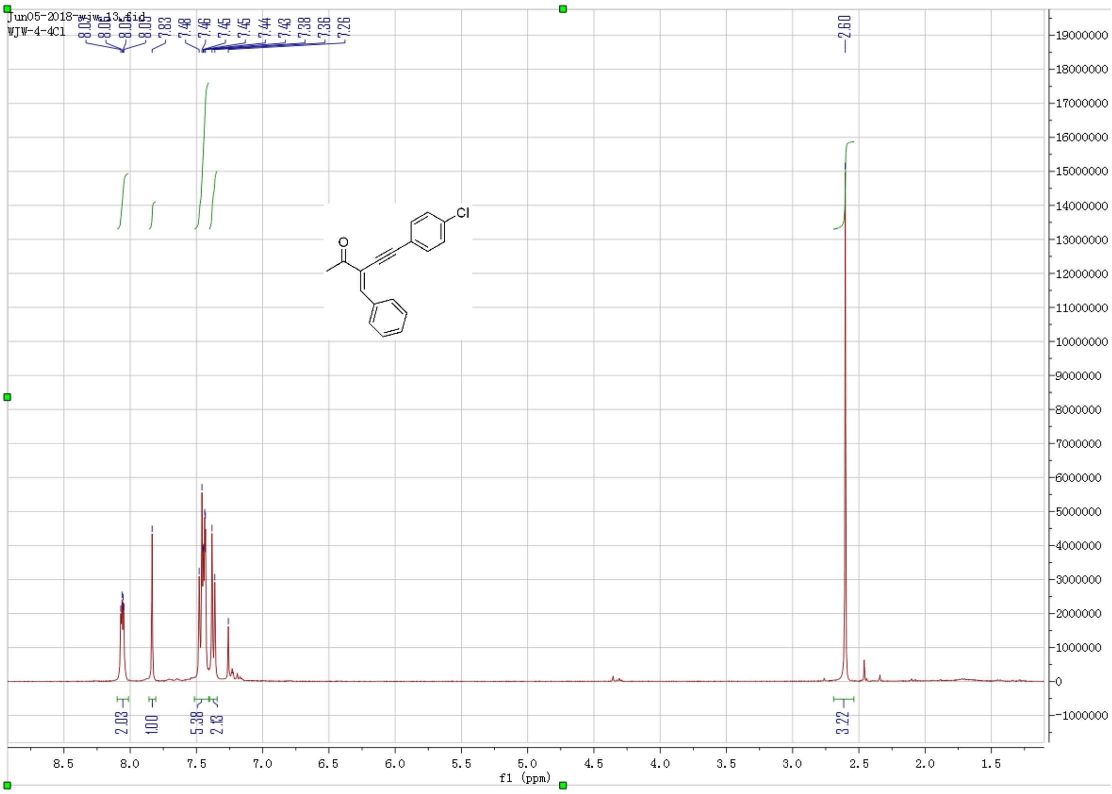

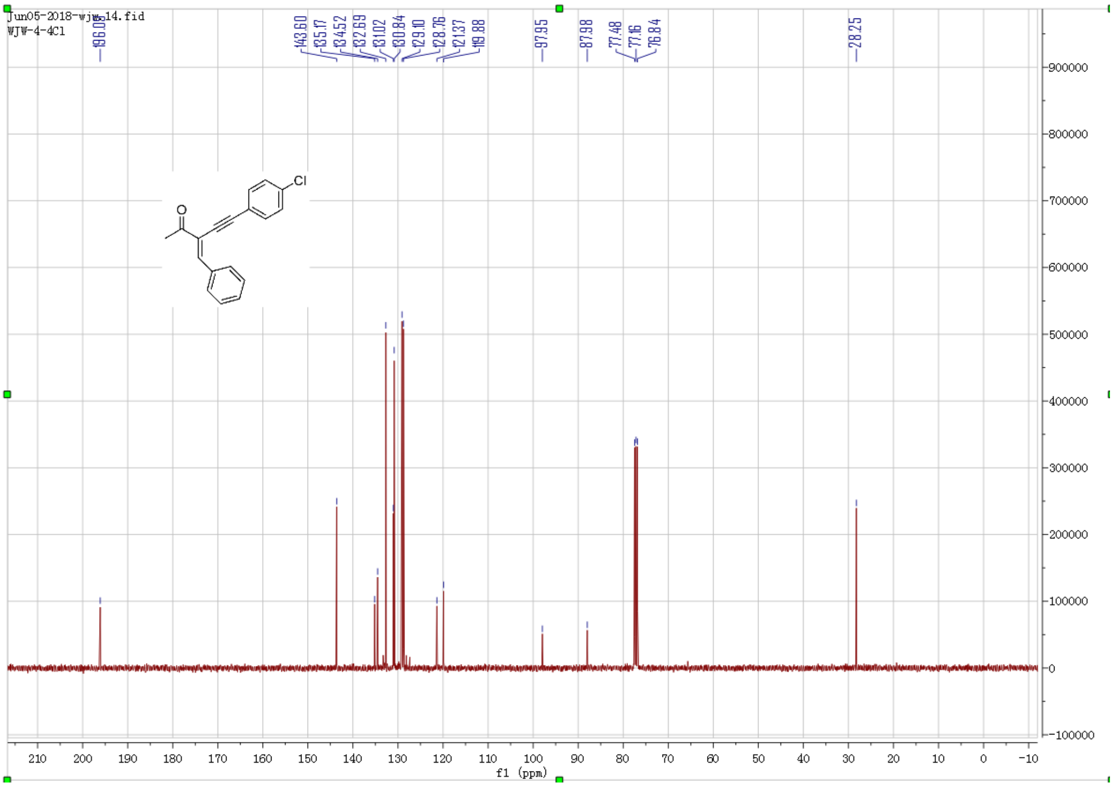

Methyl (*E*)-3-(3-benzylidene-4-oxopent-1-yn-1-yl)benzoate (**1g**)

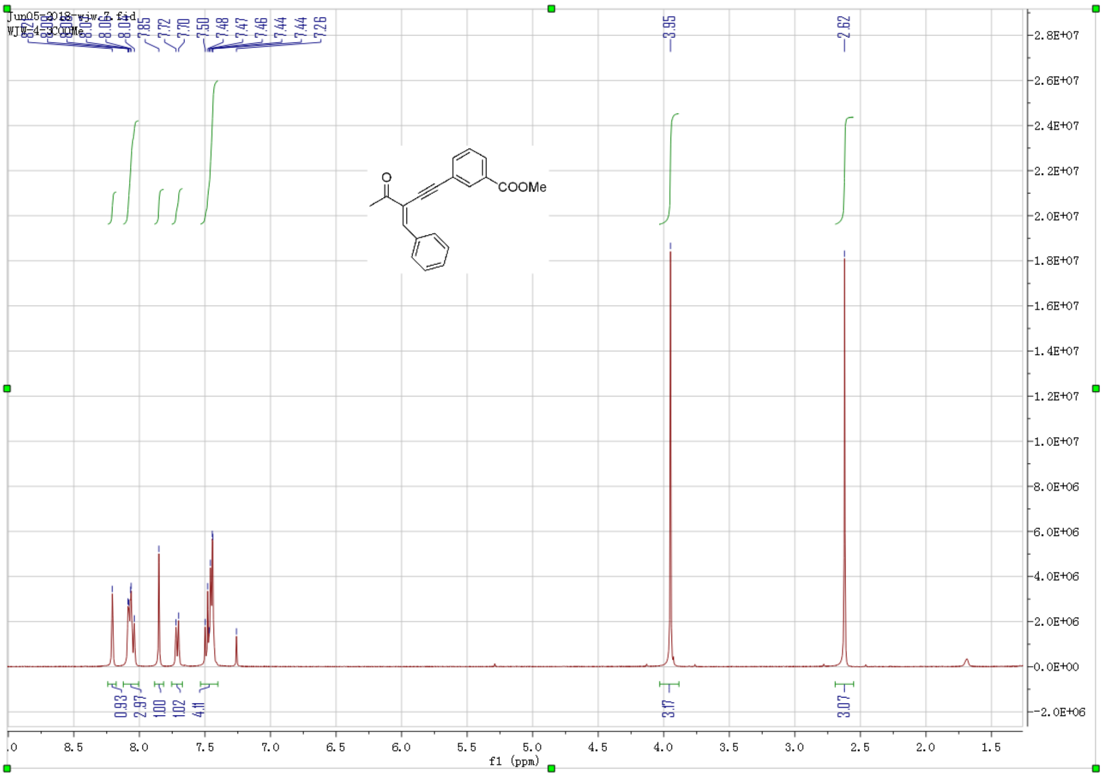

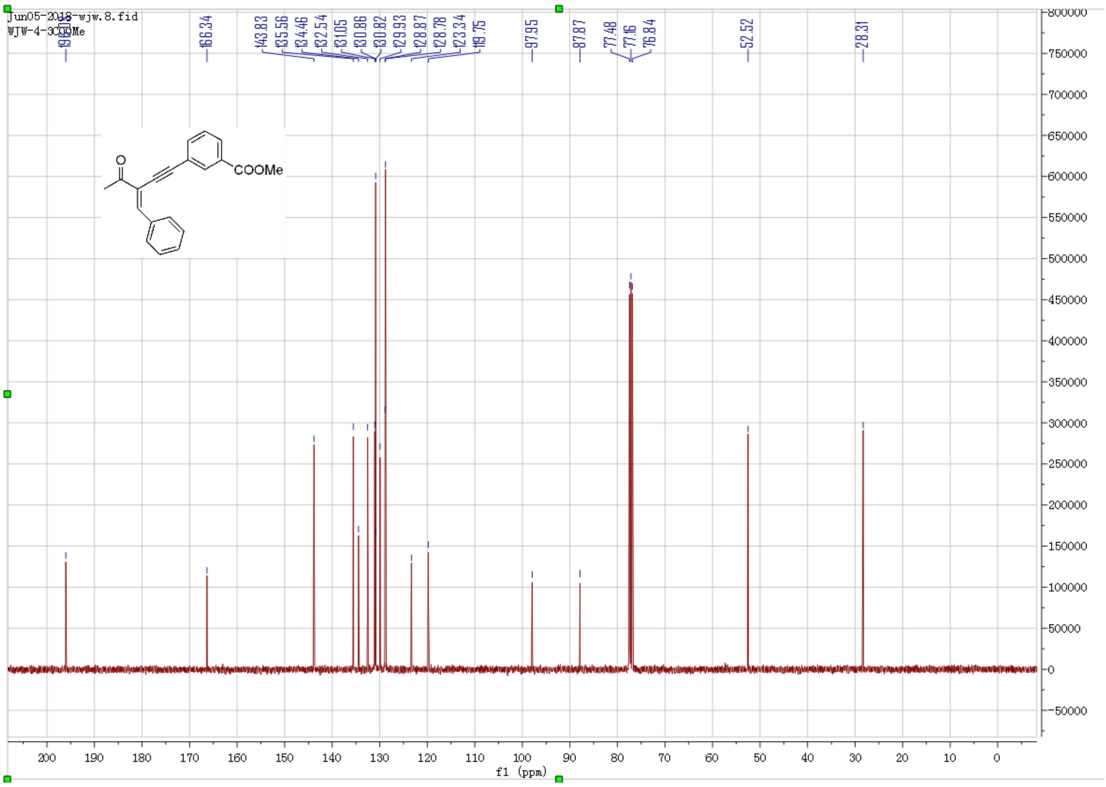

(*E*)-3-benzylidene-5-(2-chlorophenyl)pent-4-yn-2-one (**1h**)

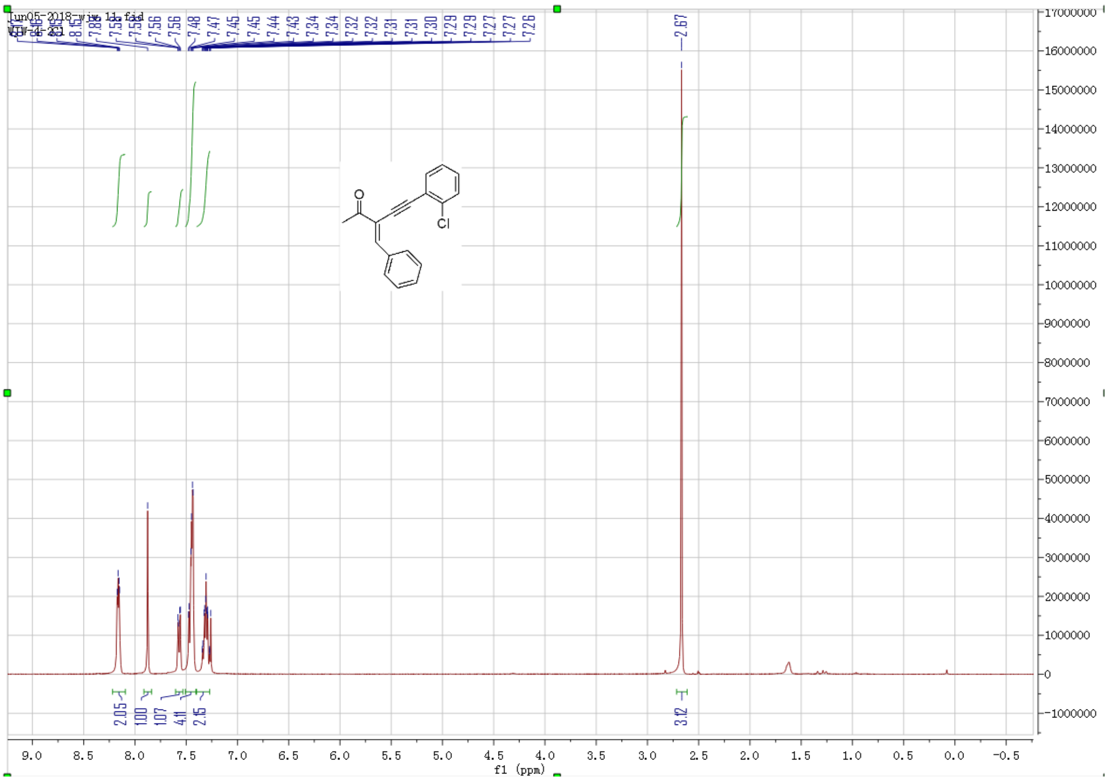

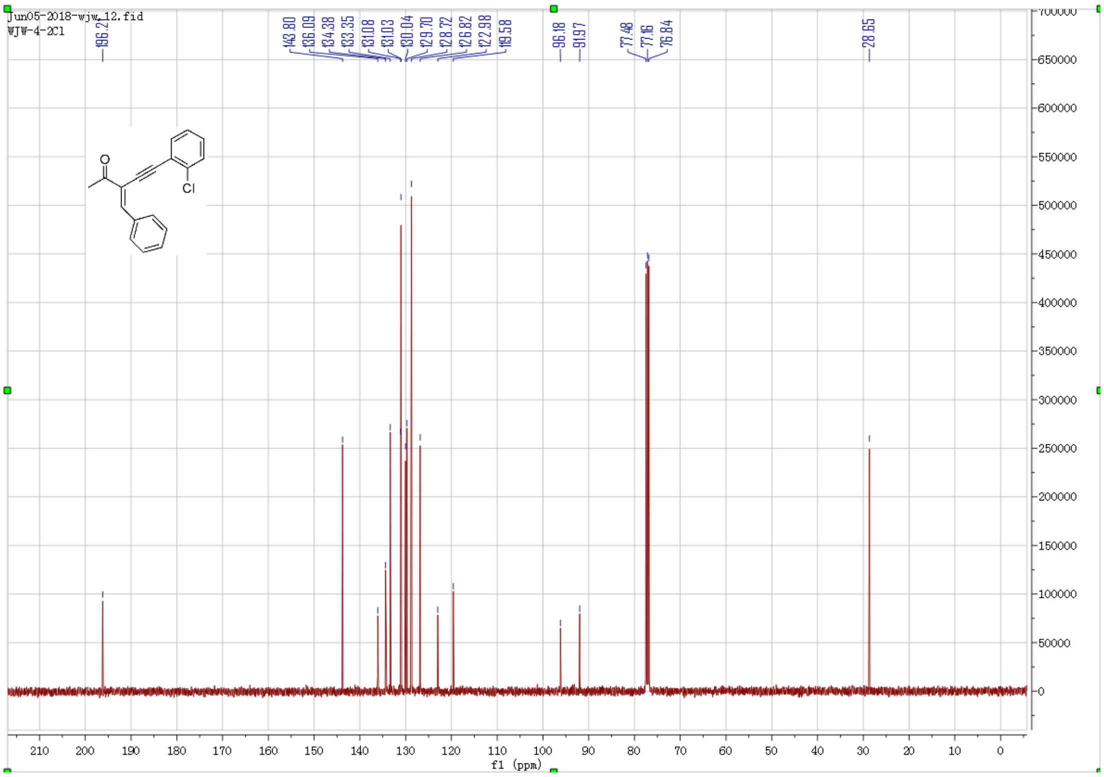

(*E*)-3-benzylidene-5-(thiophen-3-yl)pent-4-yn-2-one (**1i**)

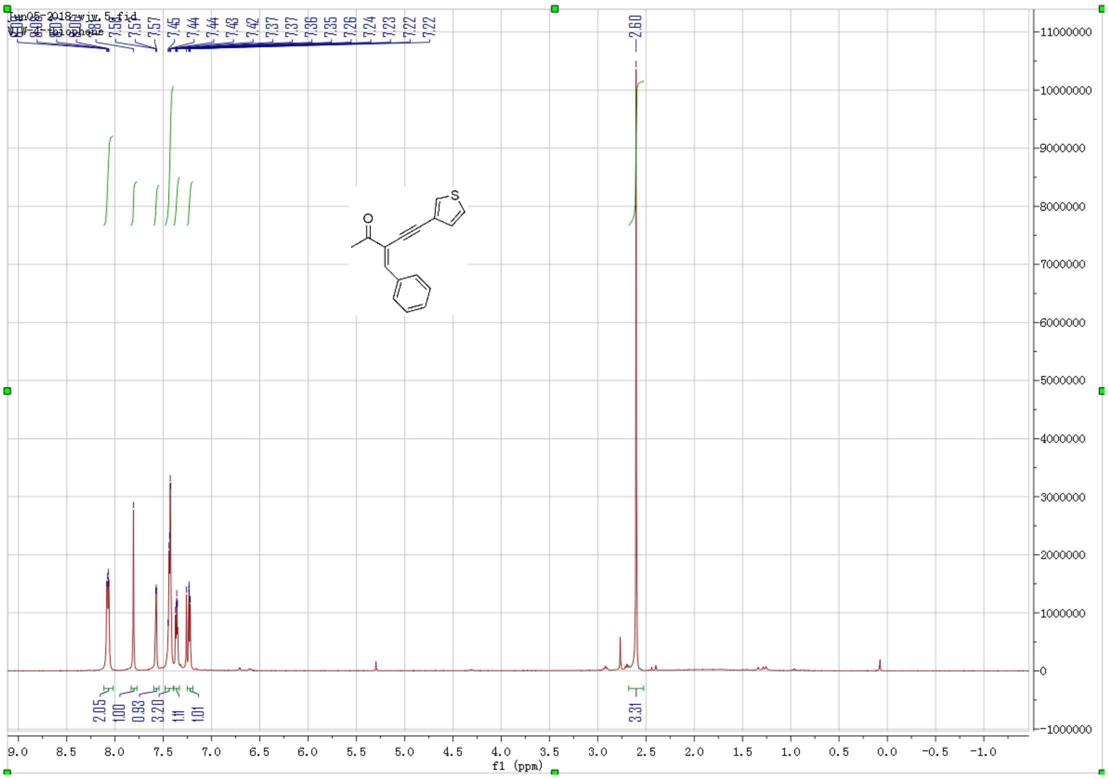

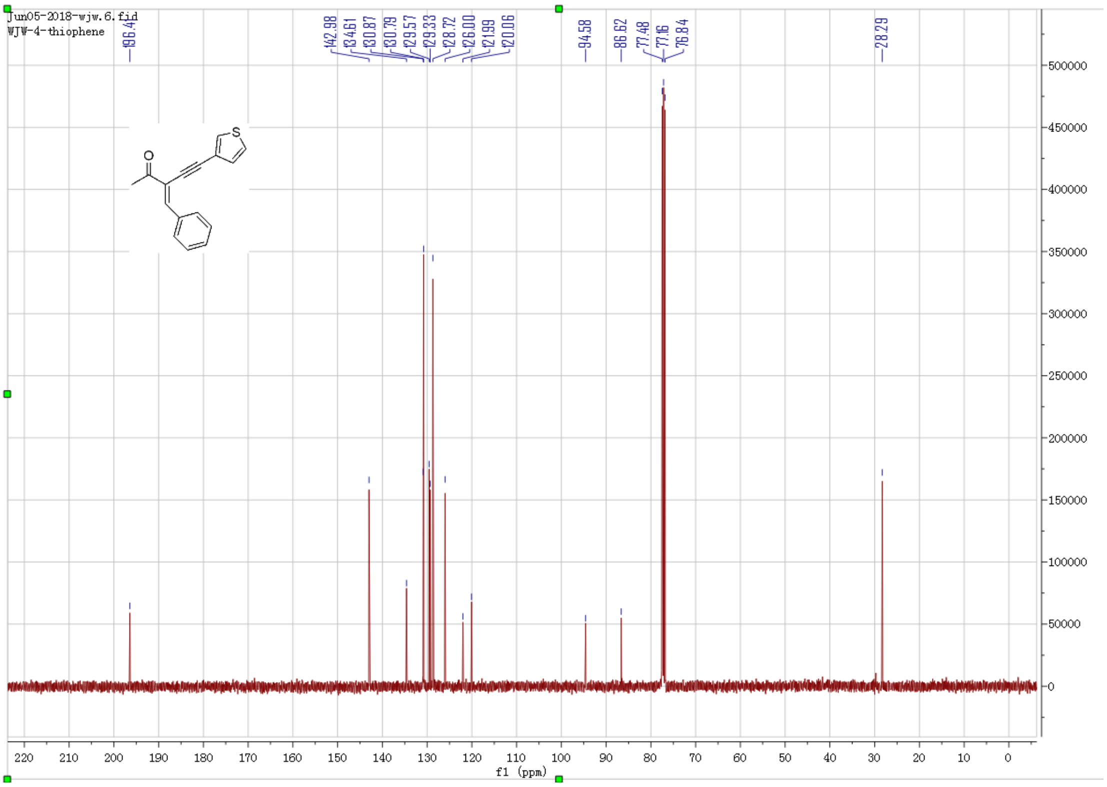

(*E*)-3-benzylidene-5-(cyclohex-1-en-1-yl)pent-4-yn-2-one (**1j**)

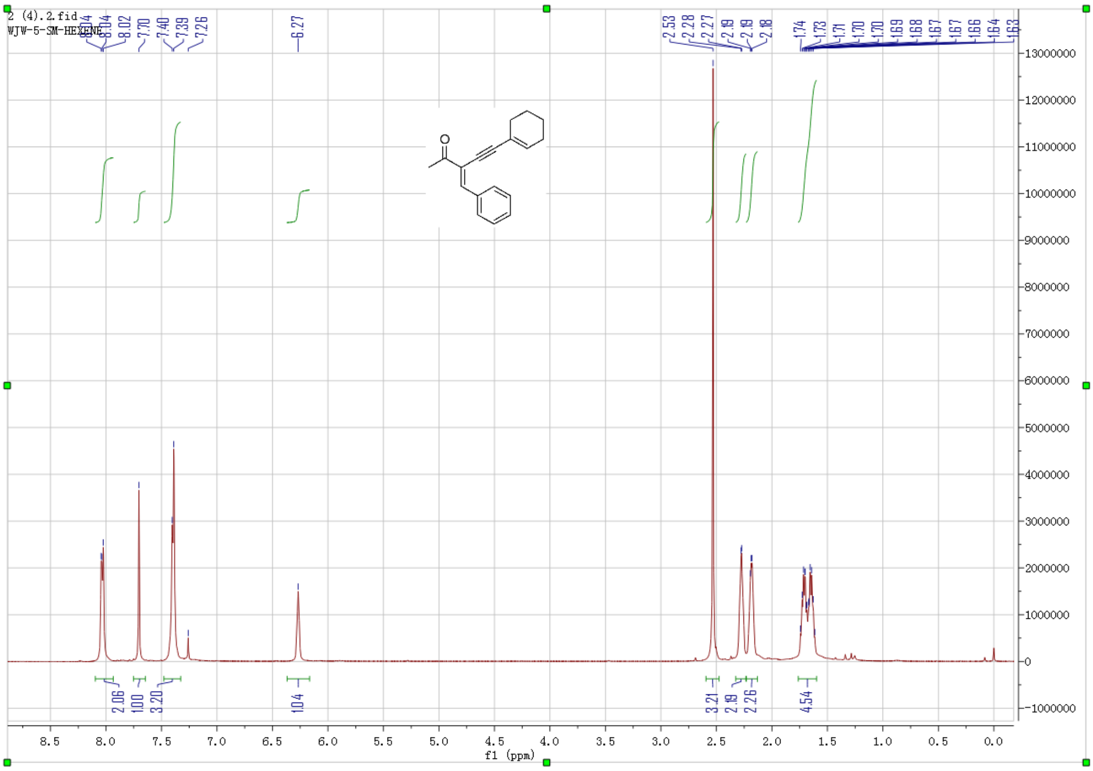

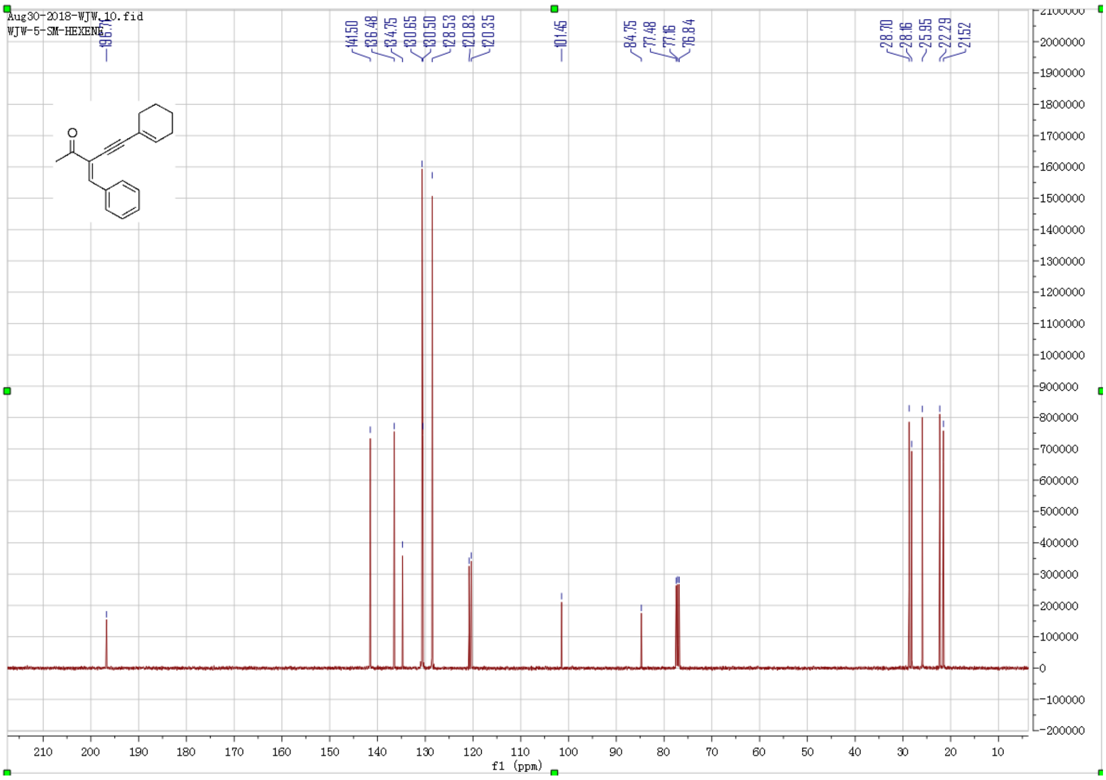

(*E*)-3-benzylidenenon-4-yn-2-one (**1k**)

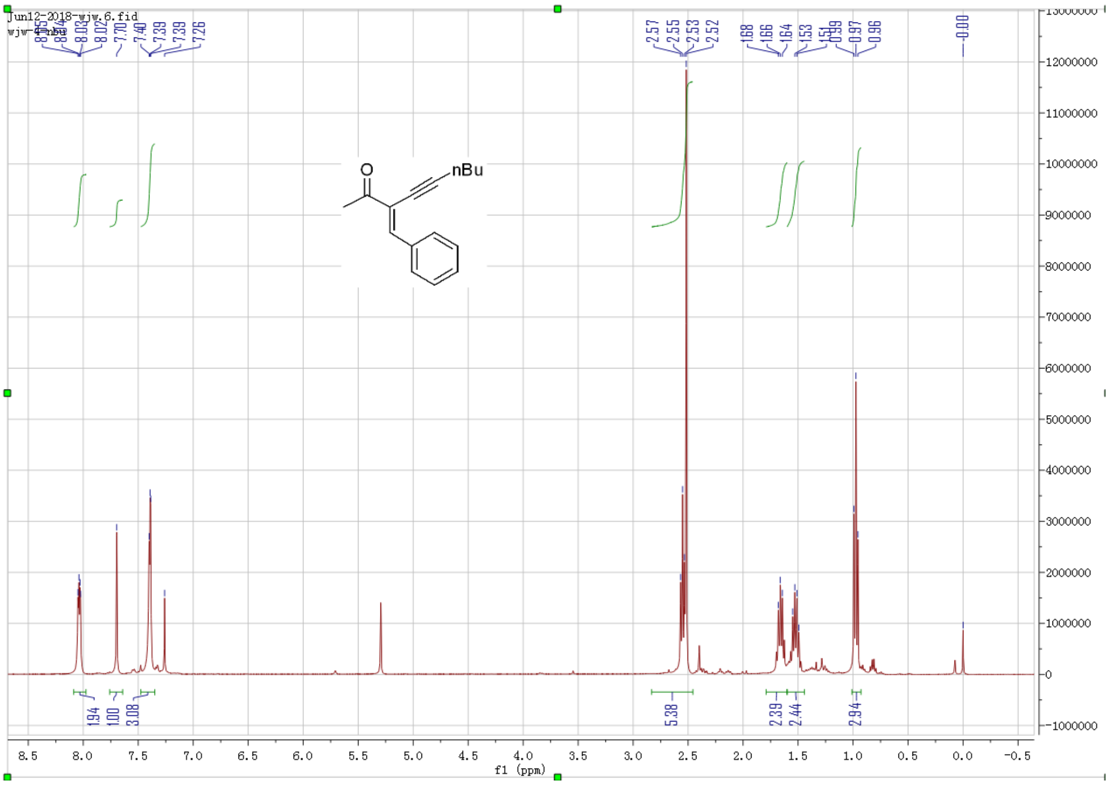

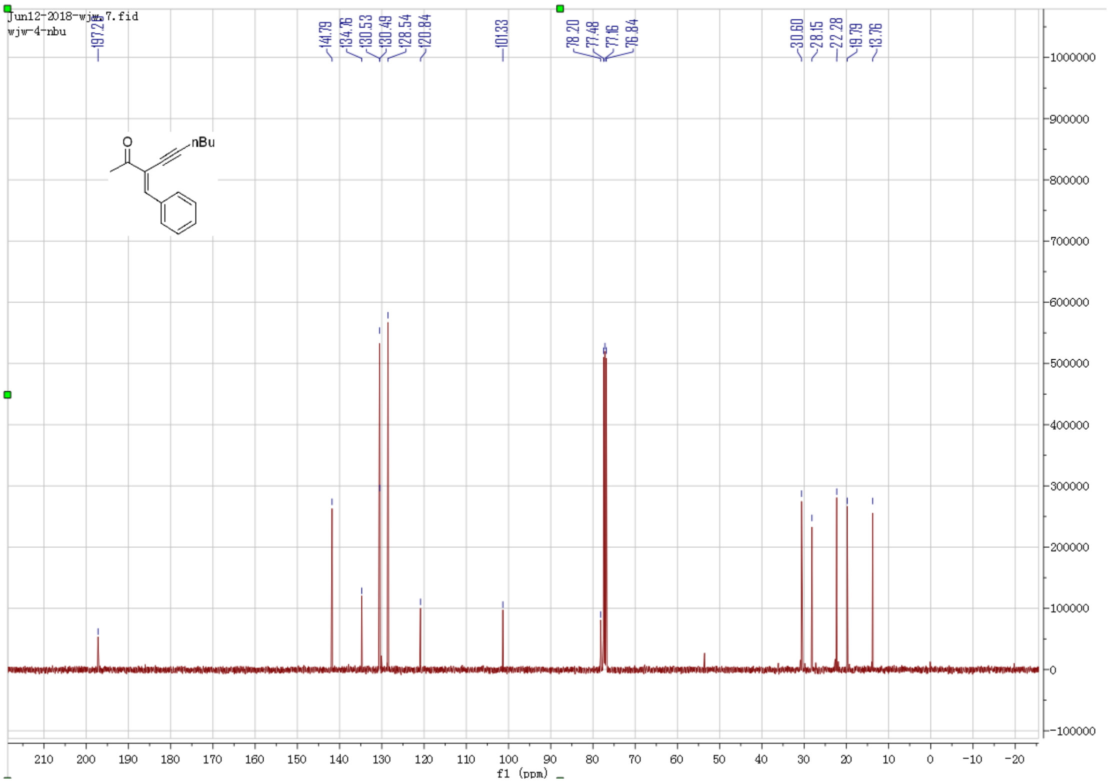

(*E*)-3-(4-fluorobenzylidene)-5-phenylpent-4-yn-2-one (**1m**)

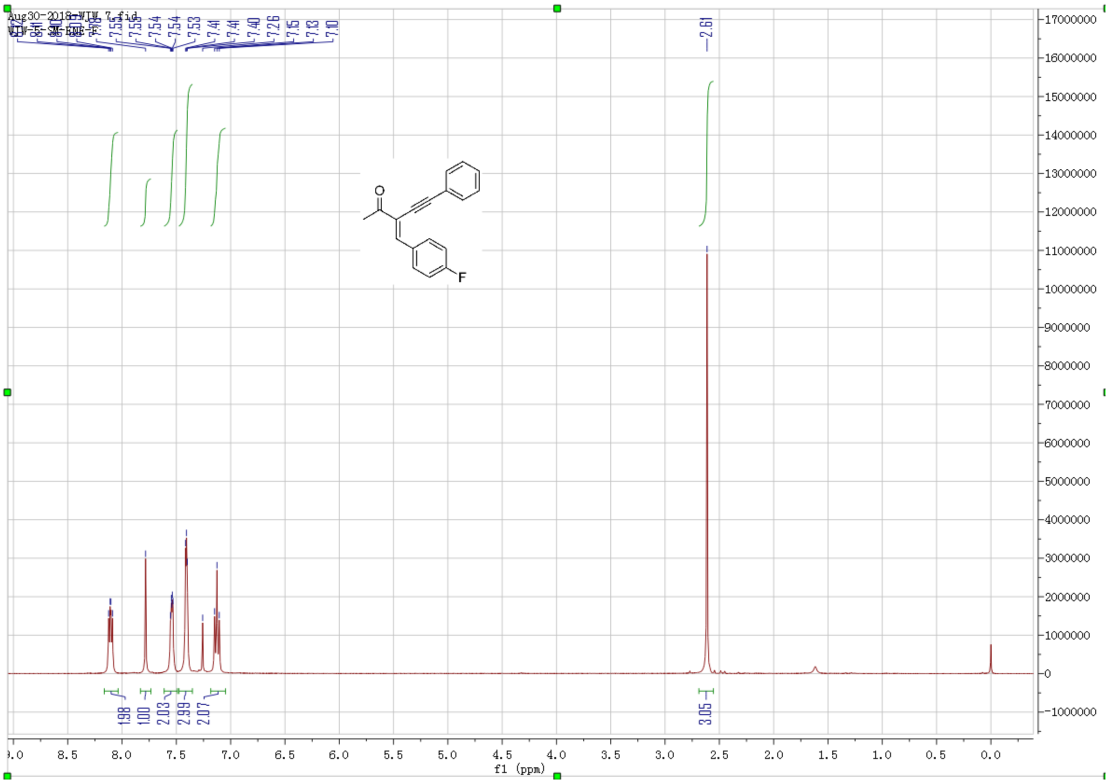

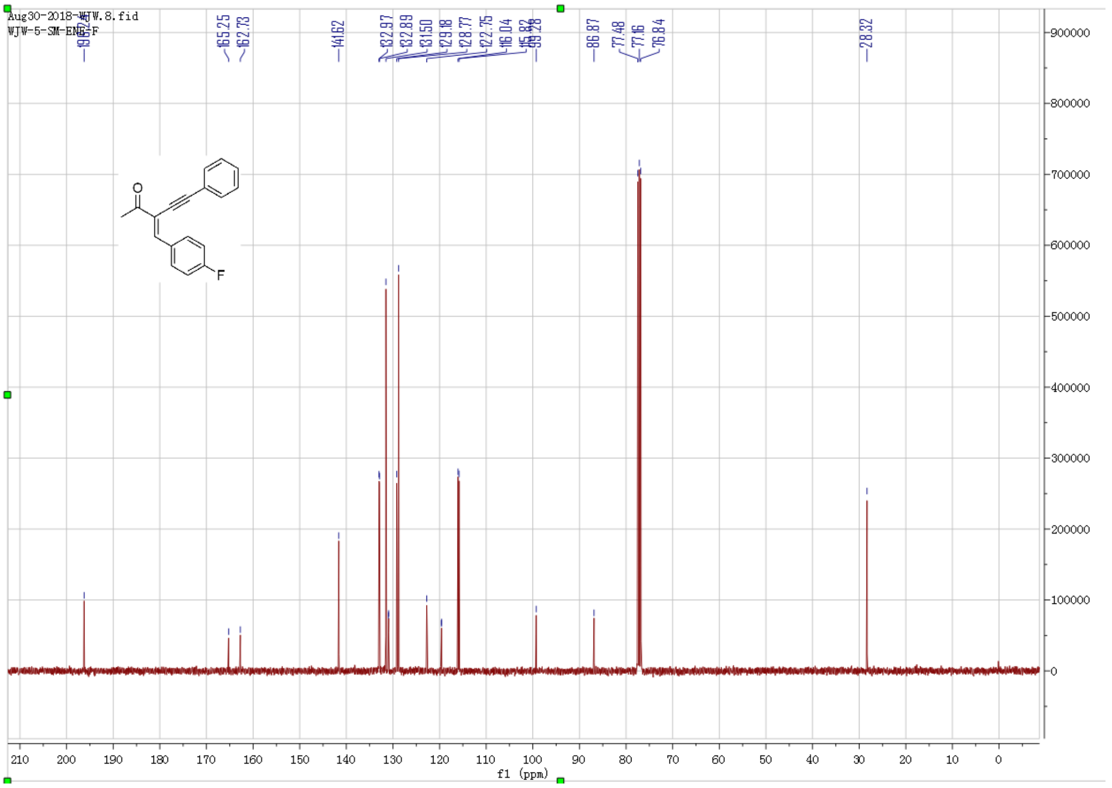

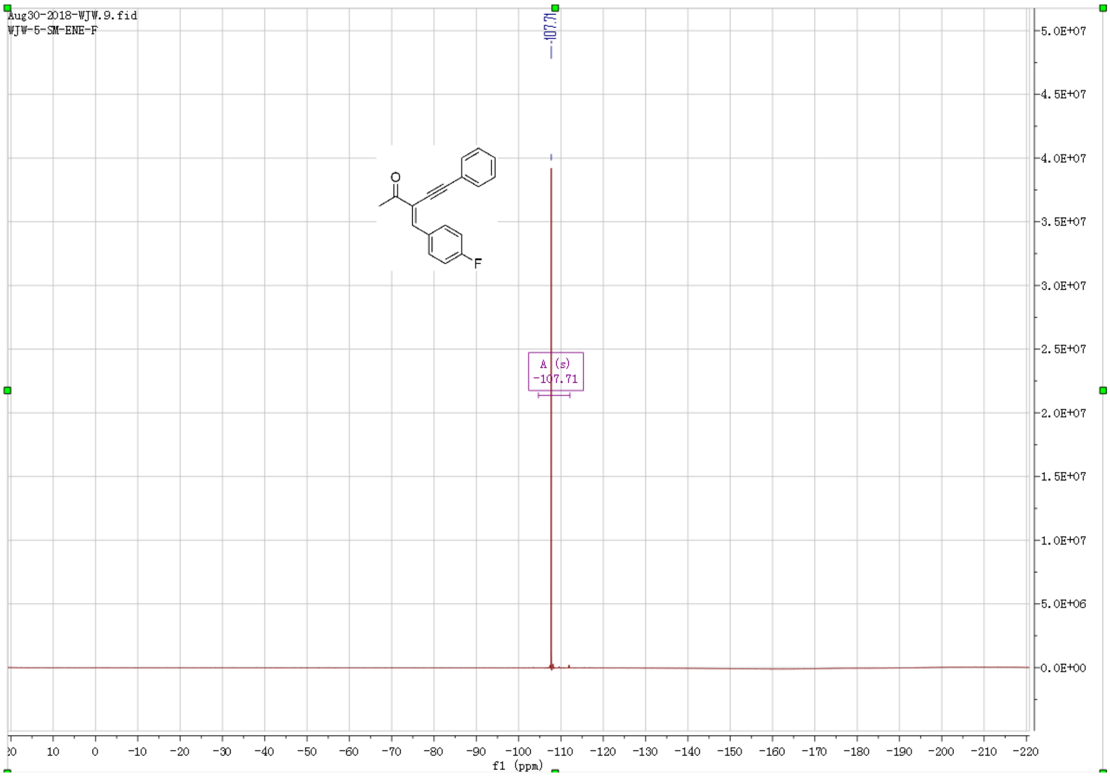

Aug30-2018-WJW.9.fid
WJW-5-SM-ENE-F
-107.71
A (s)
-107.71
F
O
f1 (ppm)

(*E*)-3-(naphthalen-2-ylmethylene)-5-phenylpent-4-yn-2-one (**1p**)

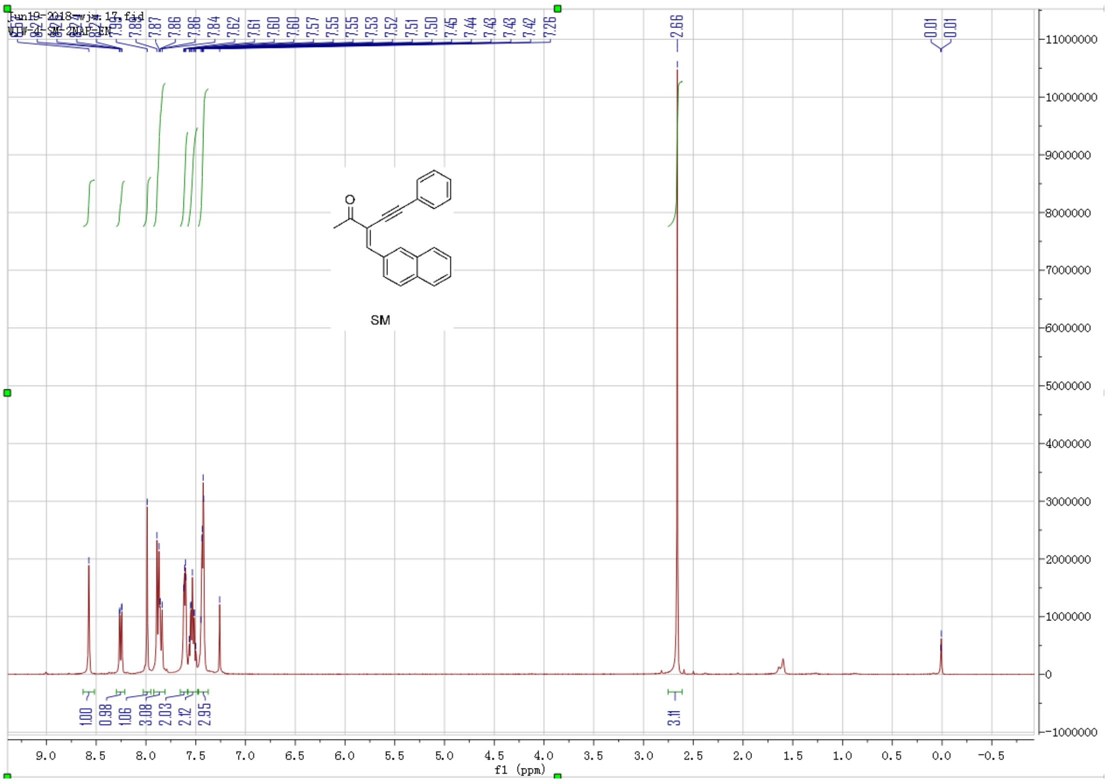

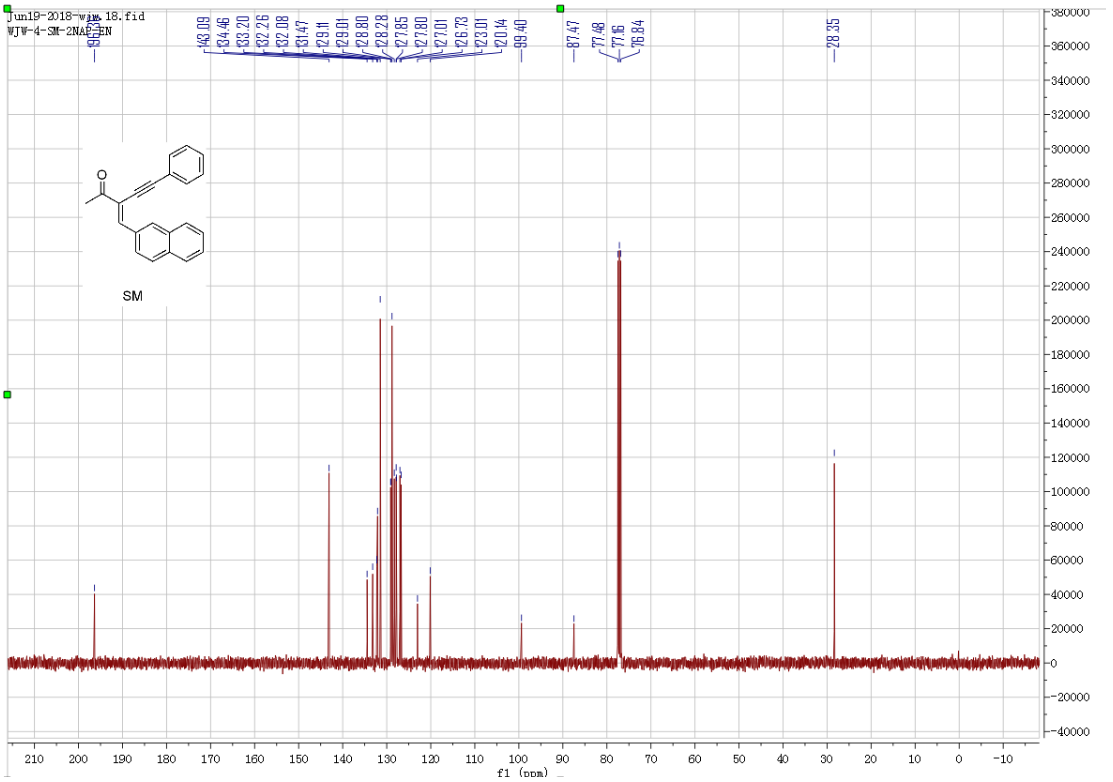

(*E*)-3-benzylidene-1-phenylhept-1-yn-4-one (**1s**)

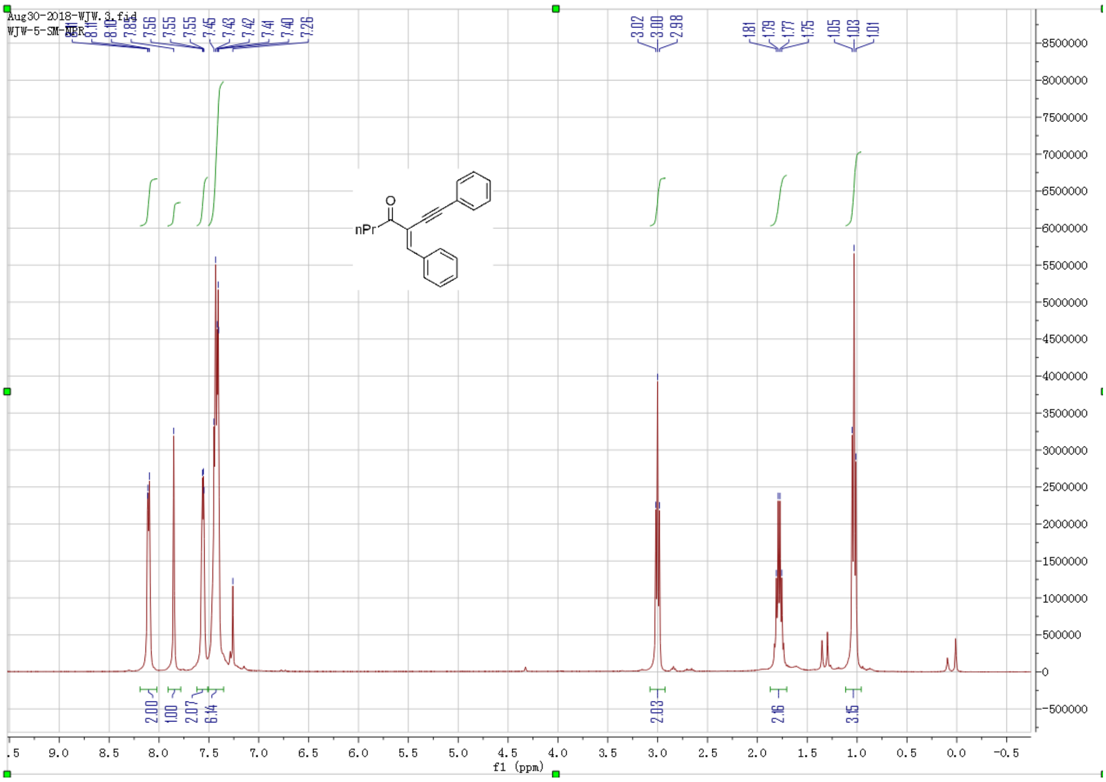

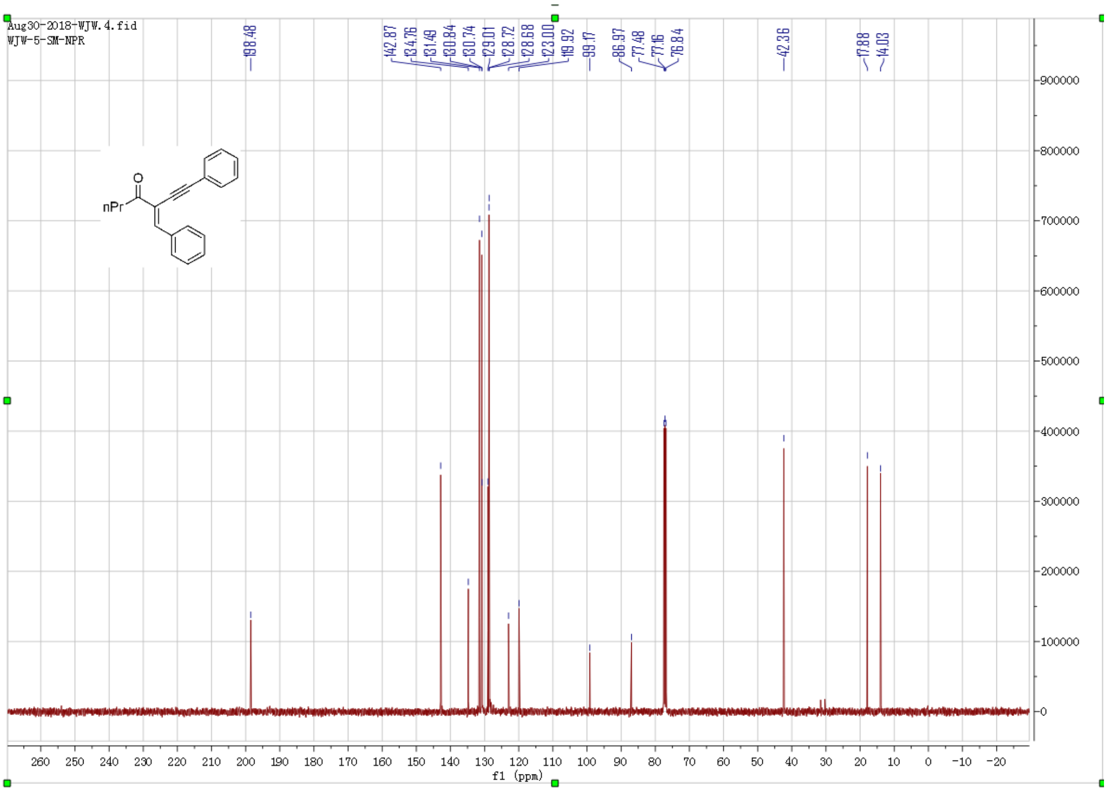

(*E*)-3-benzylidene-6-methyl-1-phenylhept-1-yn-4-one (**1u**)

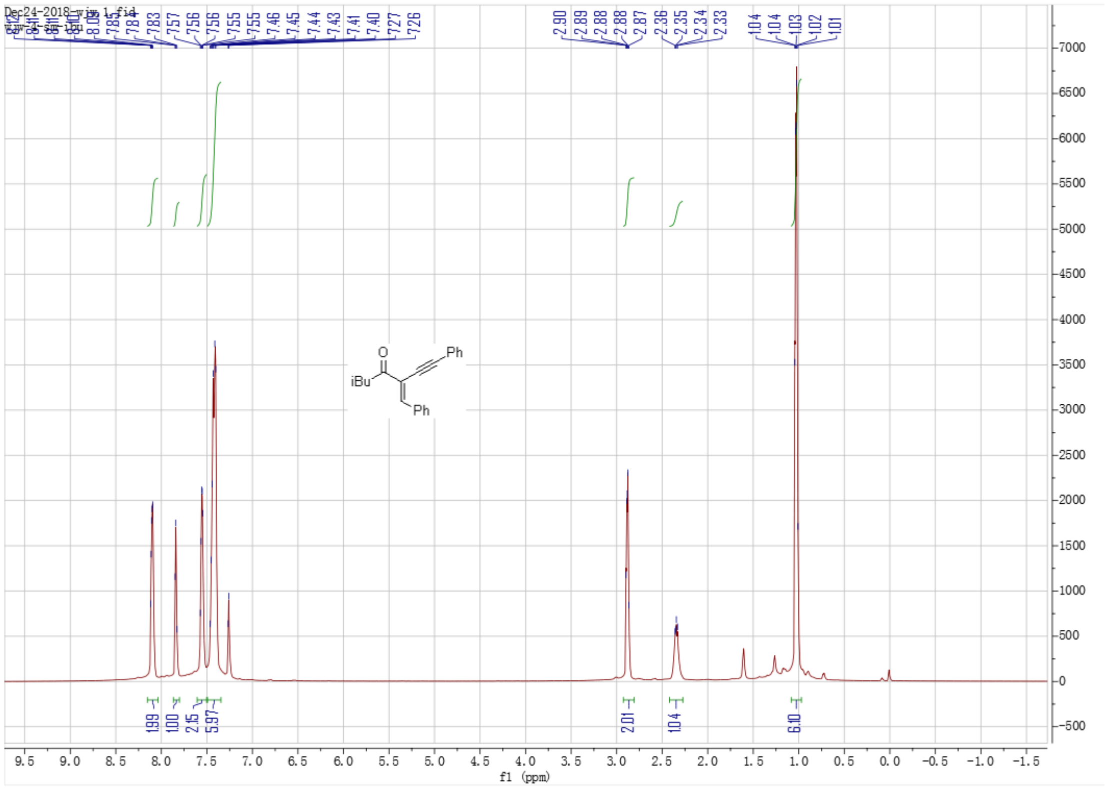

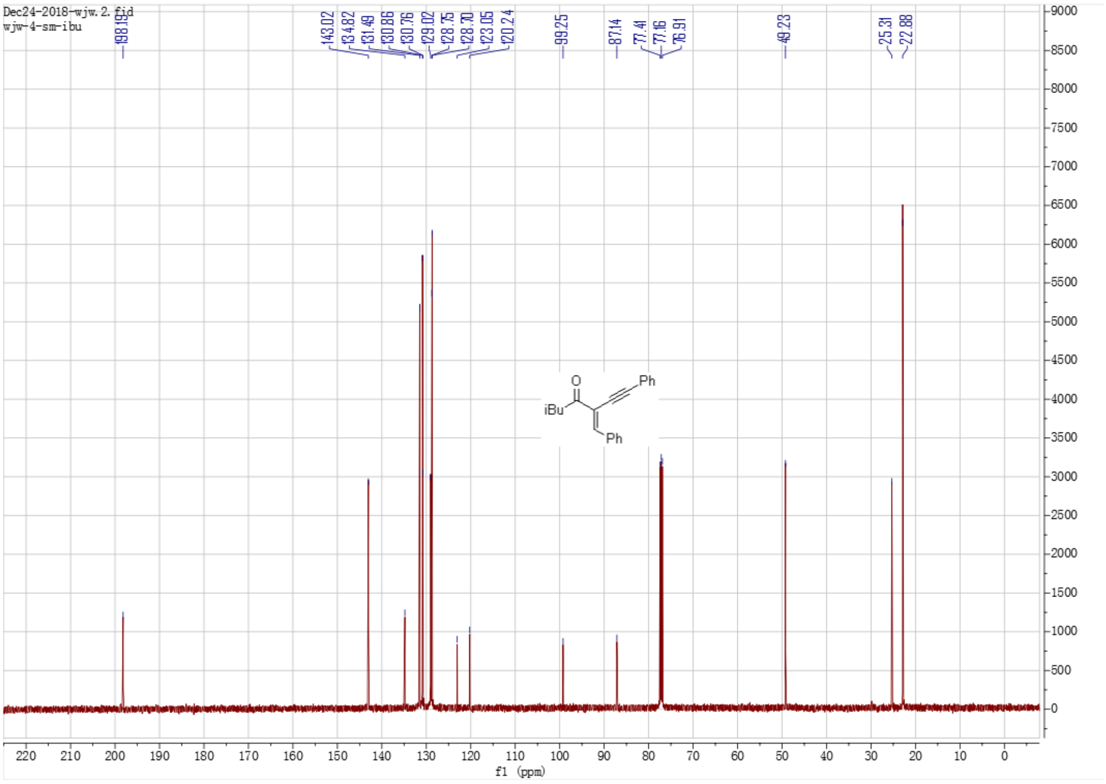

2-(3,5-dimethoxyphenyl)-4-ethyloxazol-5(4H)-one (**2a**)

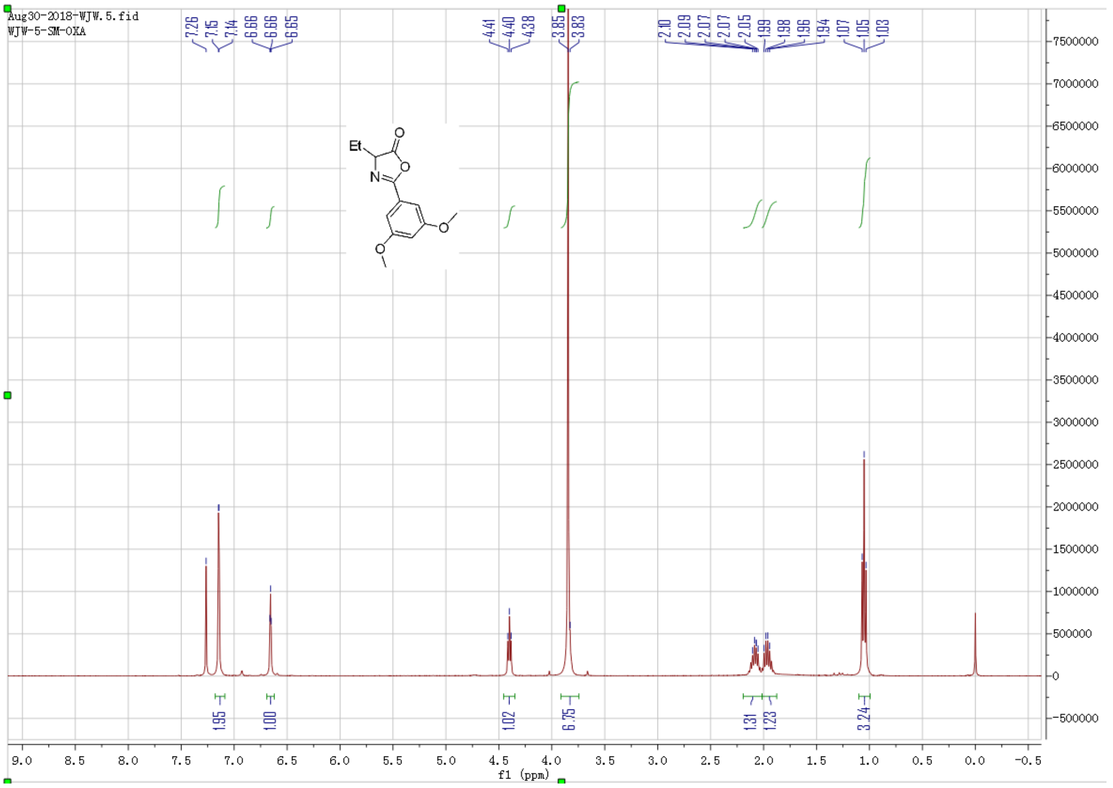

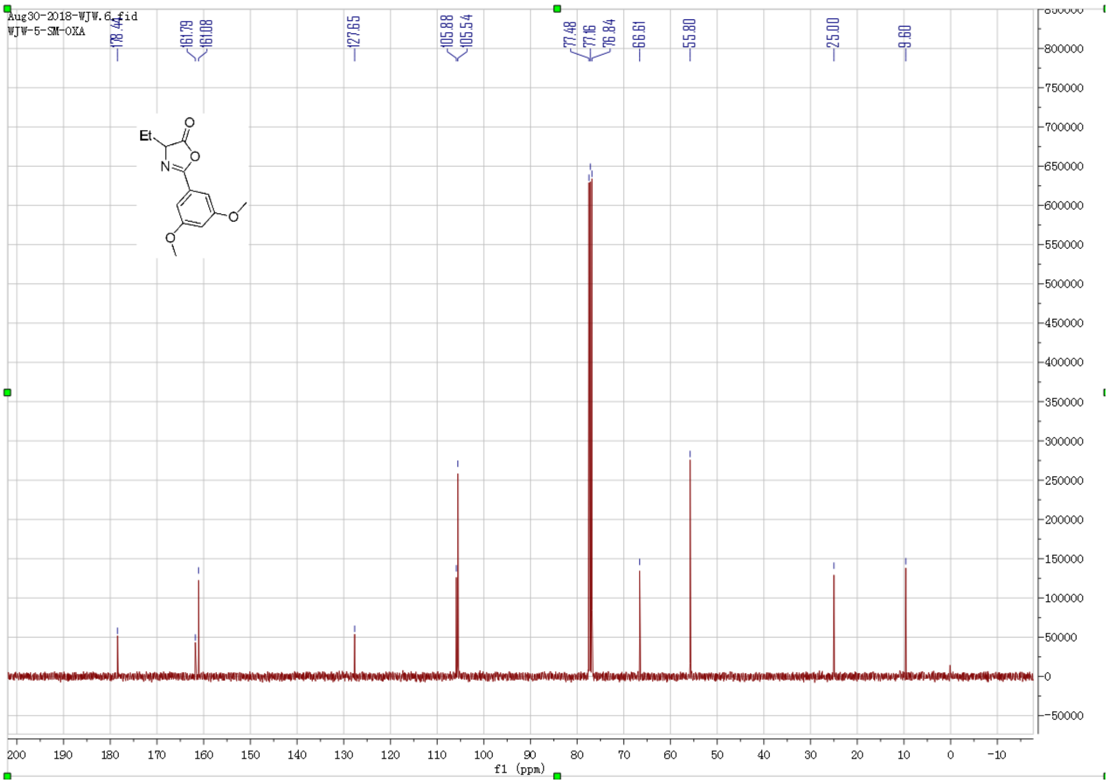

4-Butyl-2-(3,5-dimethoxyphenyl)oxazol-5(4H)-one (**2v**)

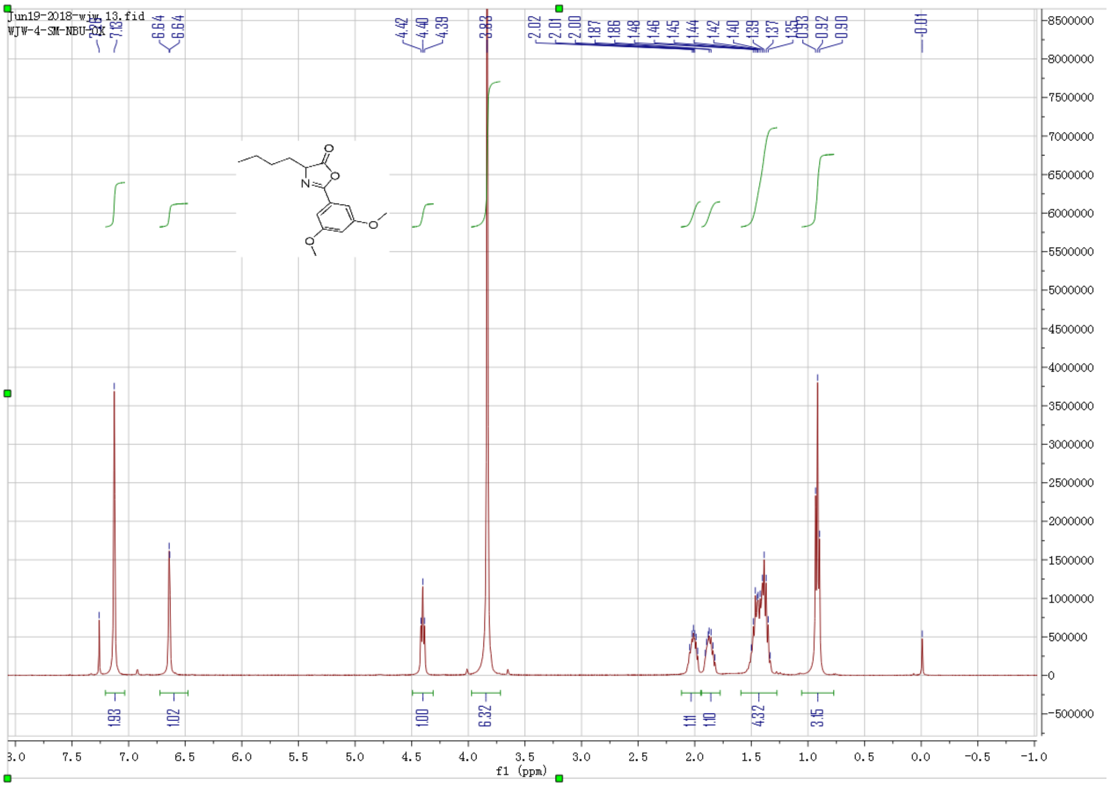

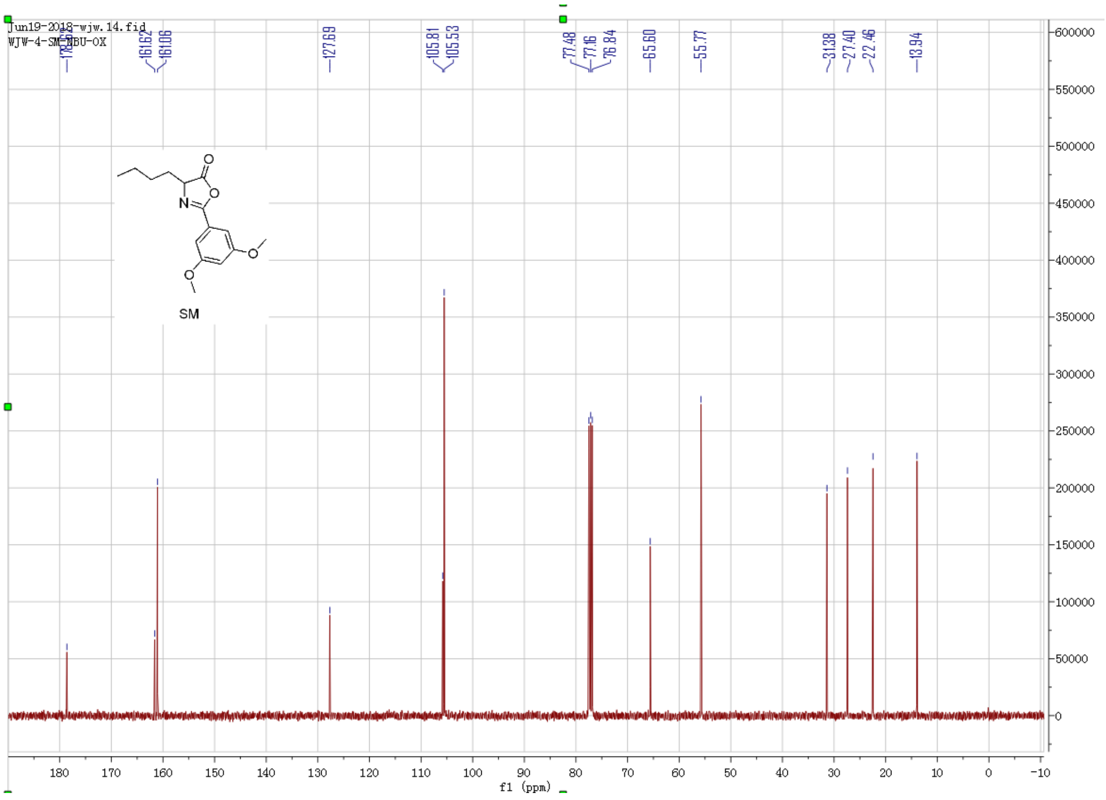

4-benzyl-2-(3,5-dimethoxyphenyl)oxazol-5(4H)-one (**2w**)

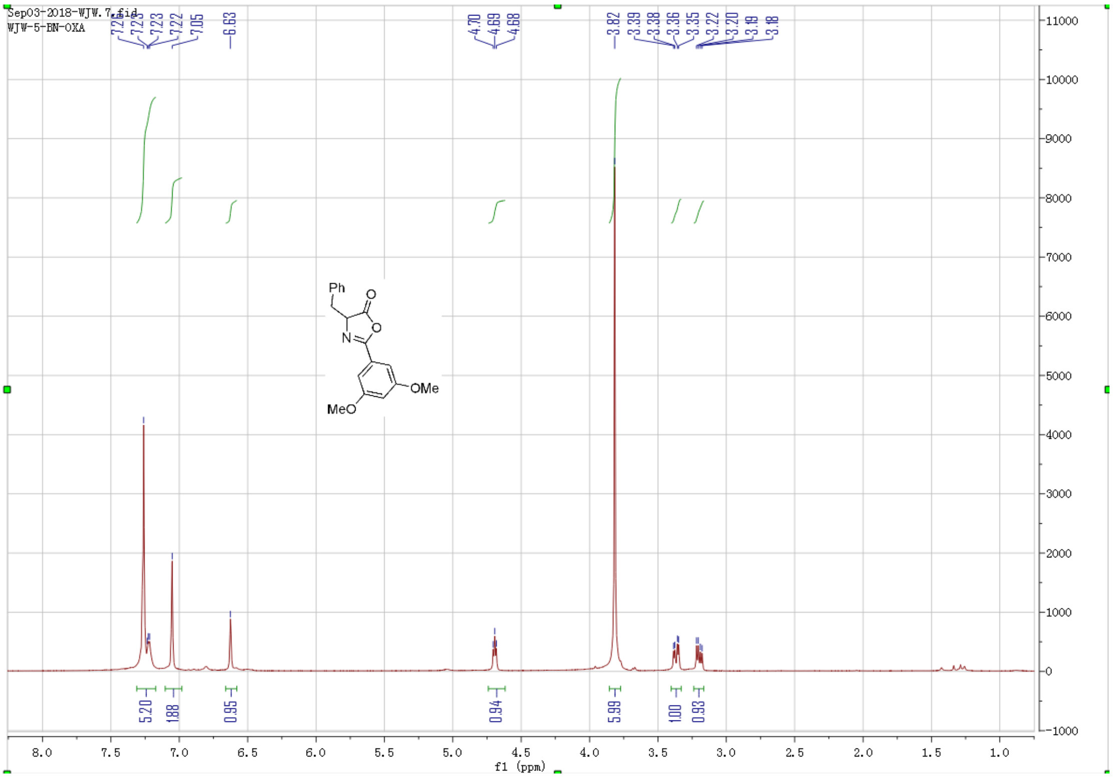

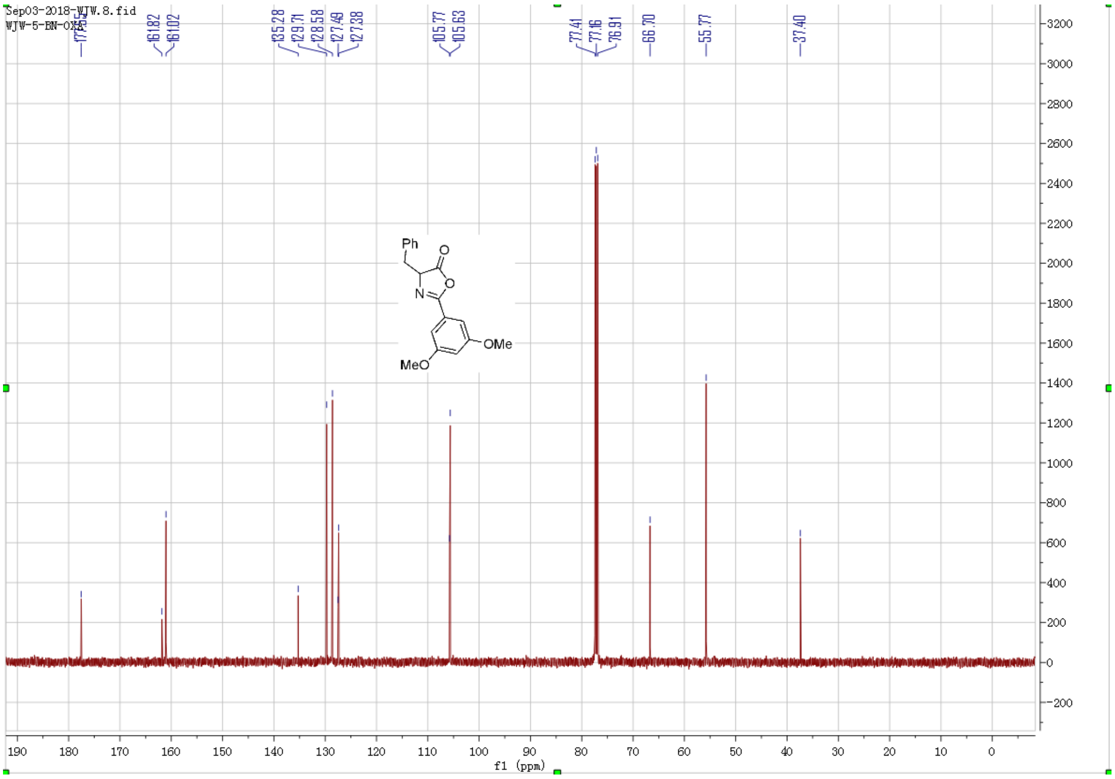

2-(3,5-dimethoxyphenyl)-4-(2-(methylthio)ethyl)oxazol-5(4H)-one (**2x**)

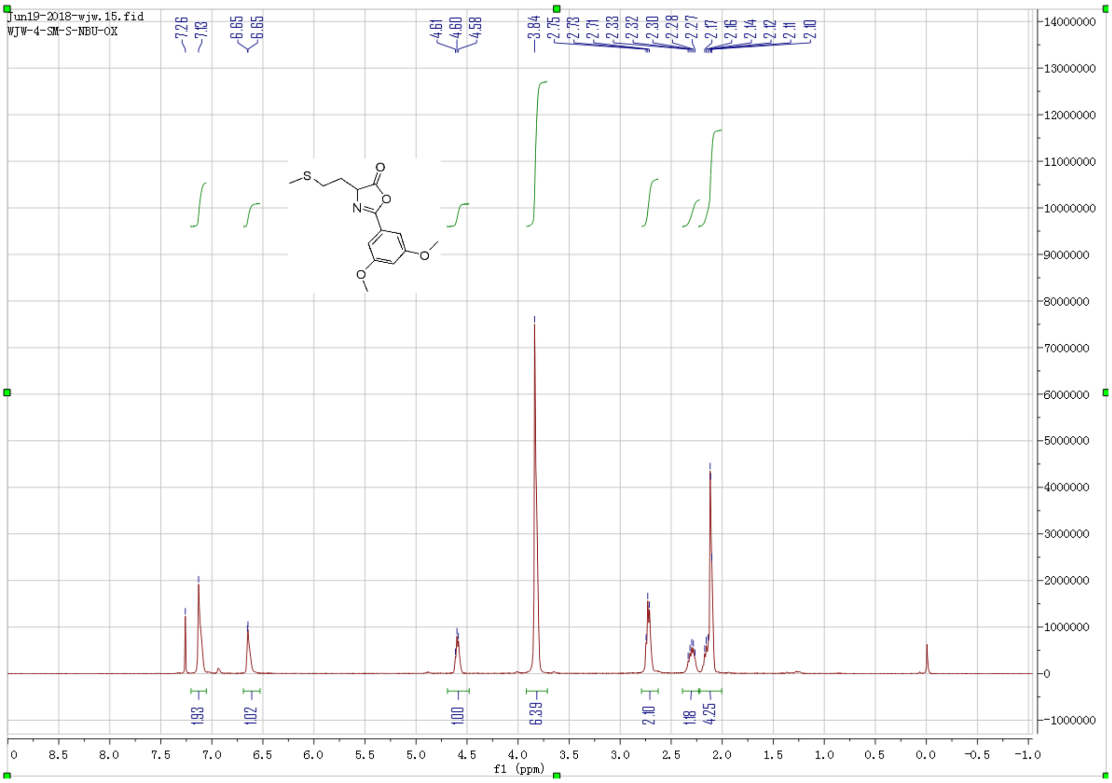

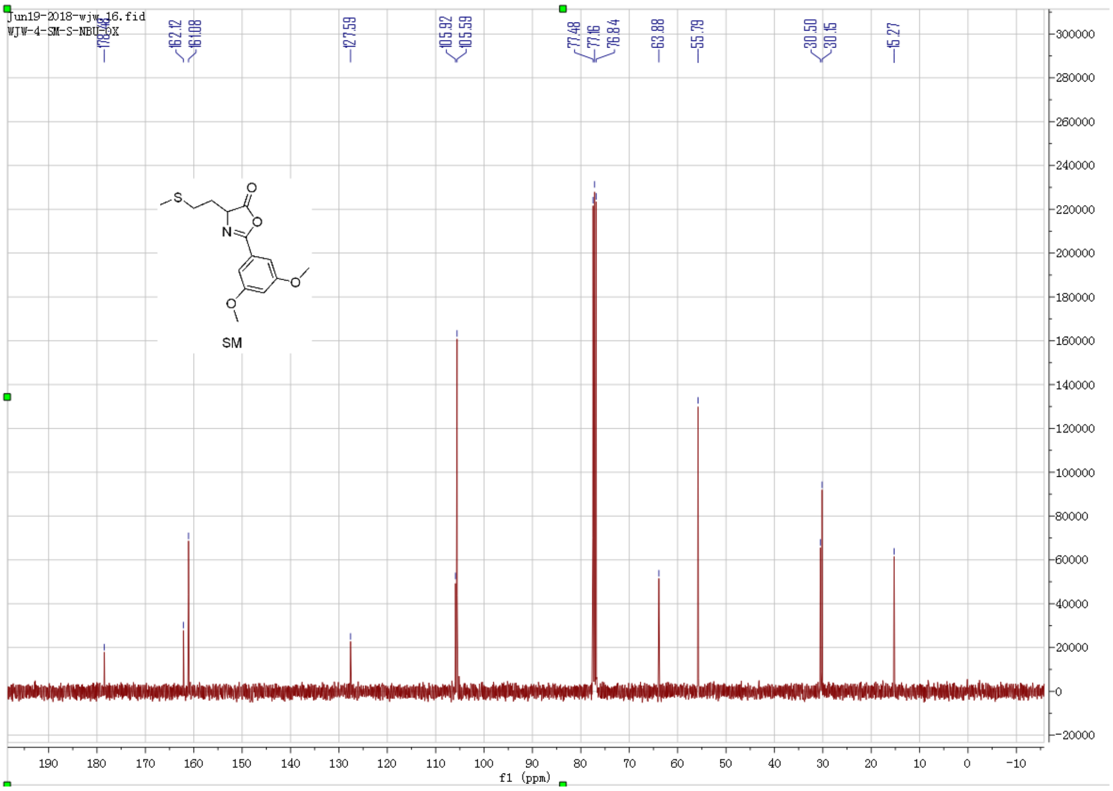

tert-butyl (4-(2-(3,5-dimethoxyphenyl)-5-oxo-4,5-dihydrooxazol-4-yl)butyl)carbamate (**2y**)

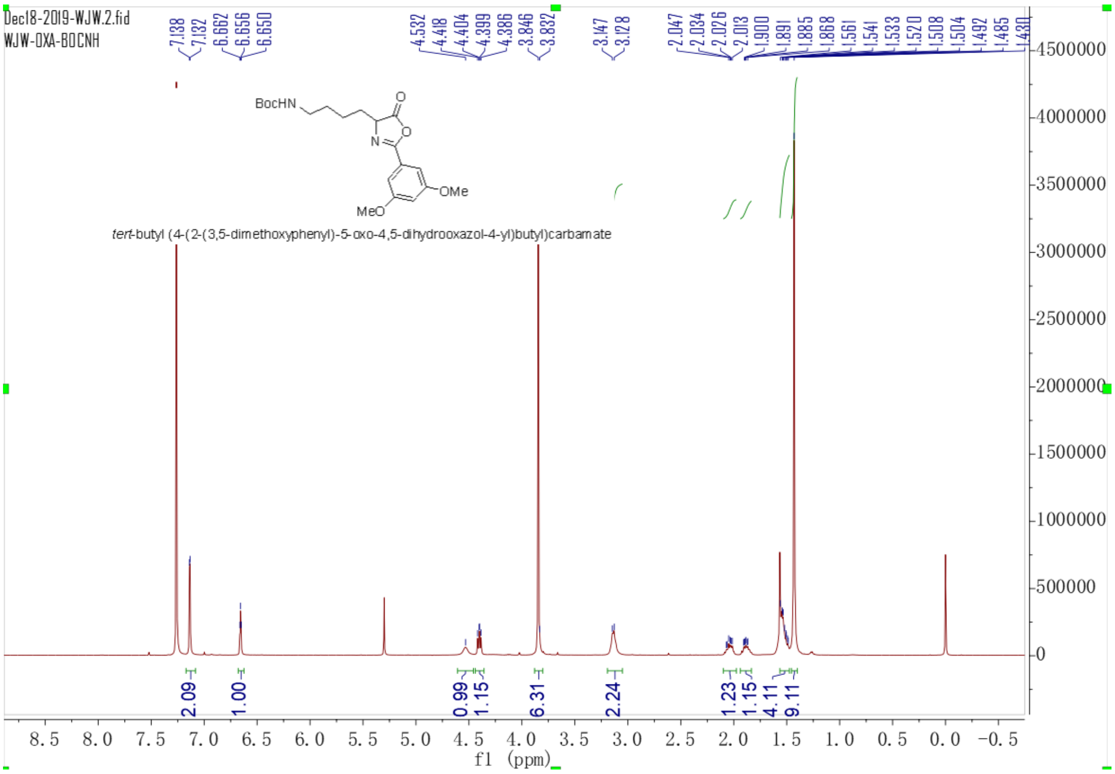

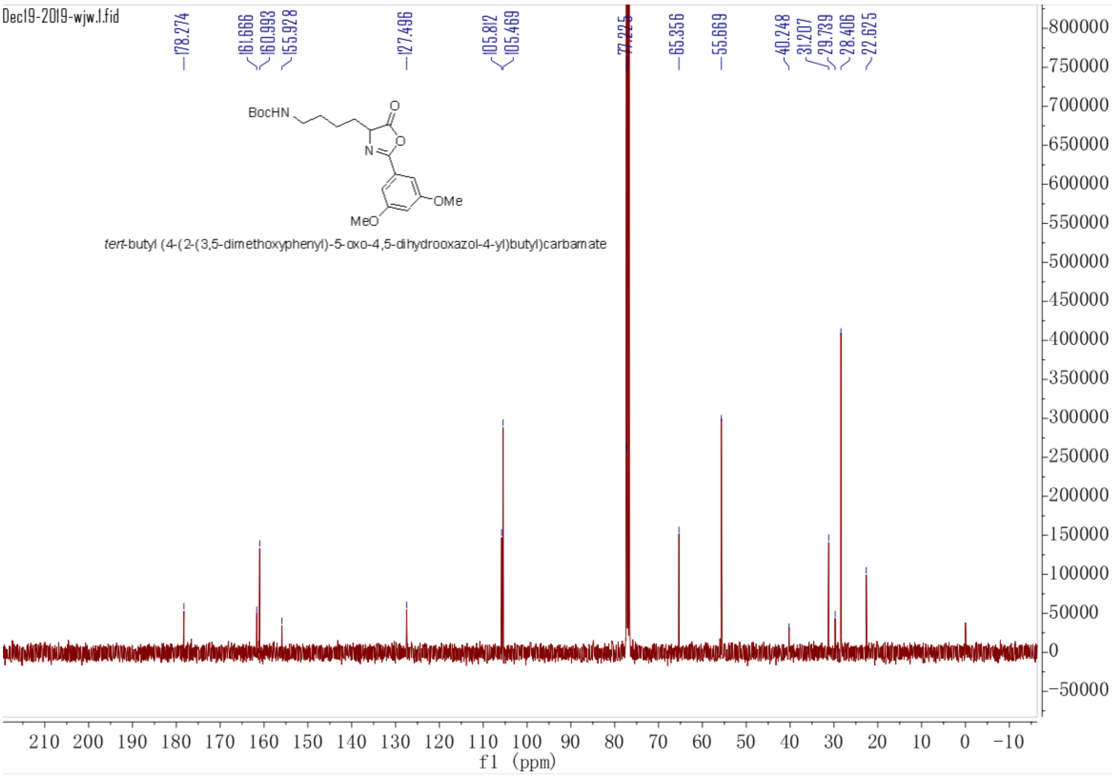

(*S*)-4-((*1R*,*3R*)-2-acetyl-1,4-diphenylbuta-2,3-dien-1-yl)-2-(3,5-dimethoxyphenyl)-4-ethyloxazol-5(4H)-one (**3a**)

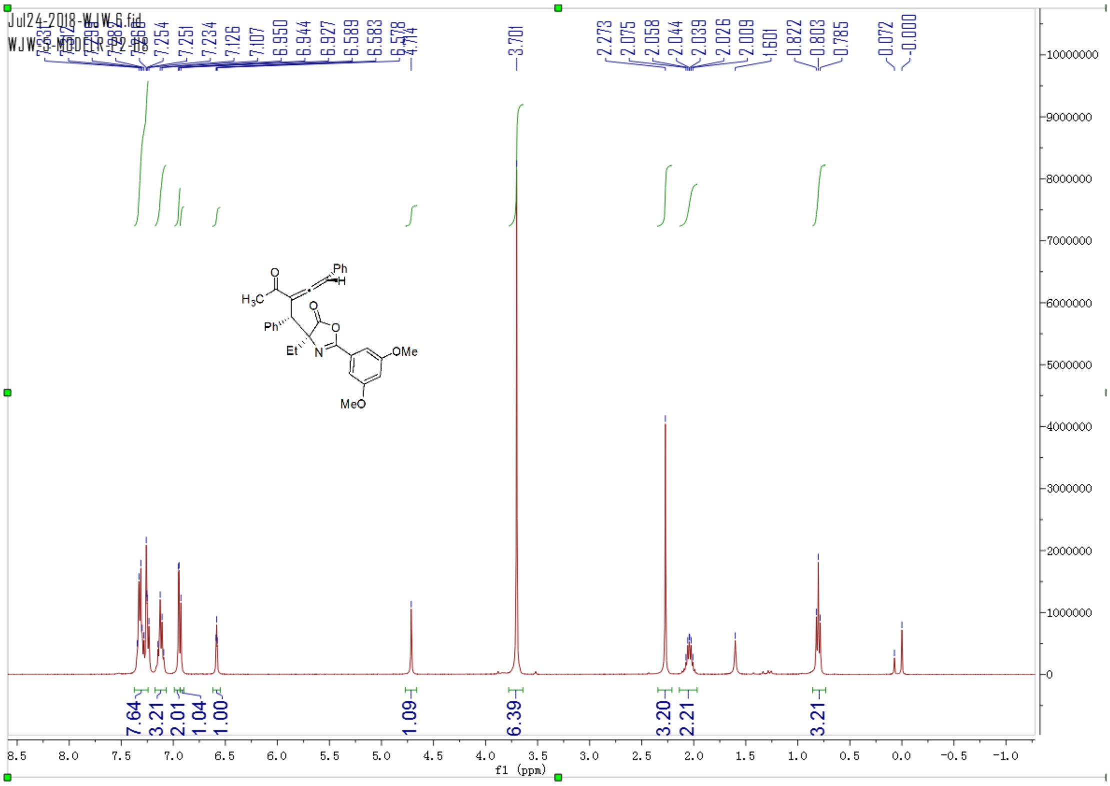

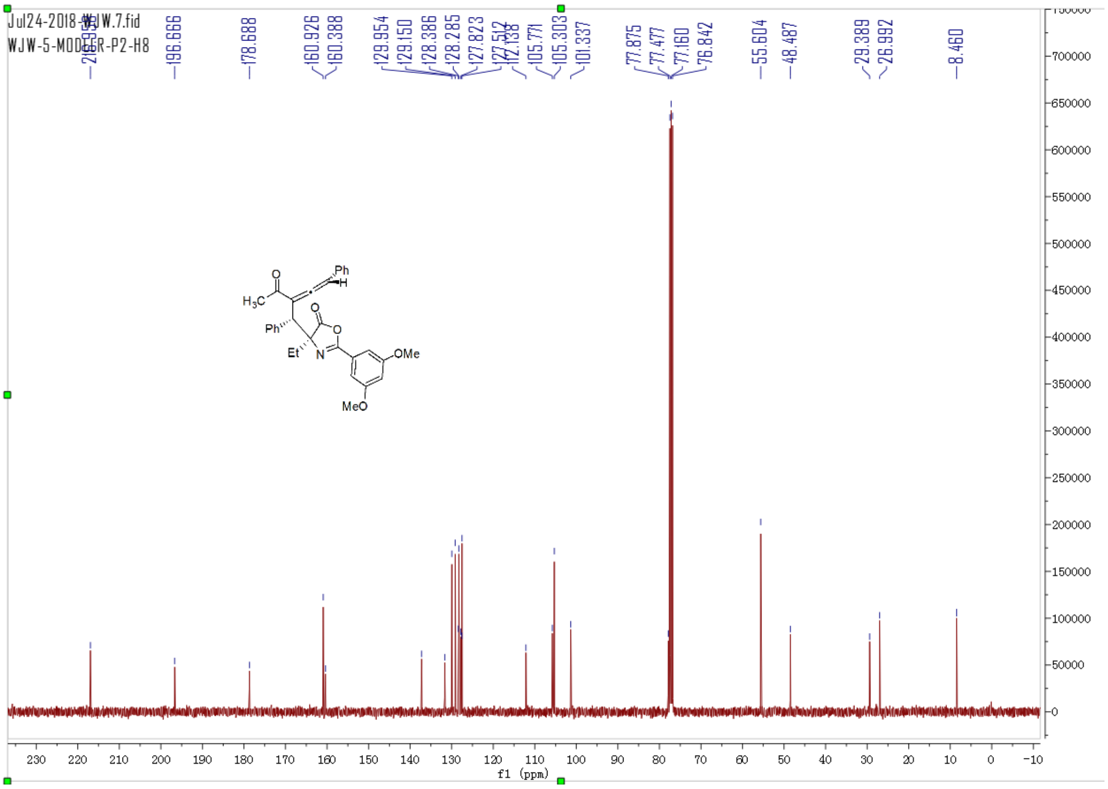

(*S*)-4-((*1R*,*3R*)-2-acetyl-1-phenyl-4-(p-tolyl)buta-2,3-dien-1-yl)-2-(3,5-dimethoxyphenyl)-4-ethyloxazol-5(4H)-one (**3b**)

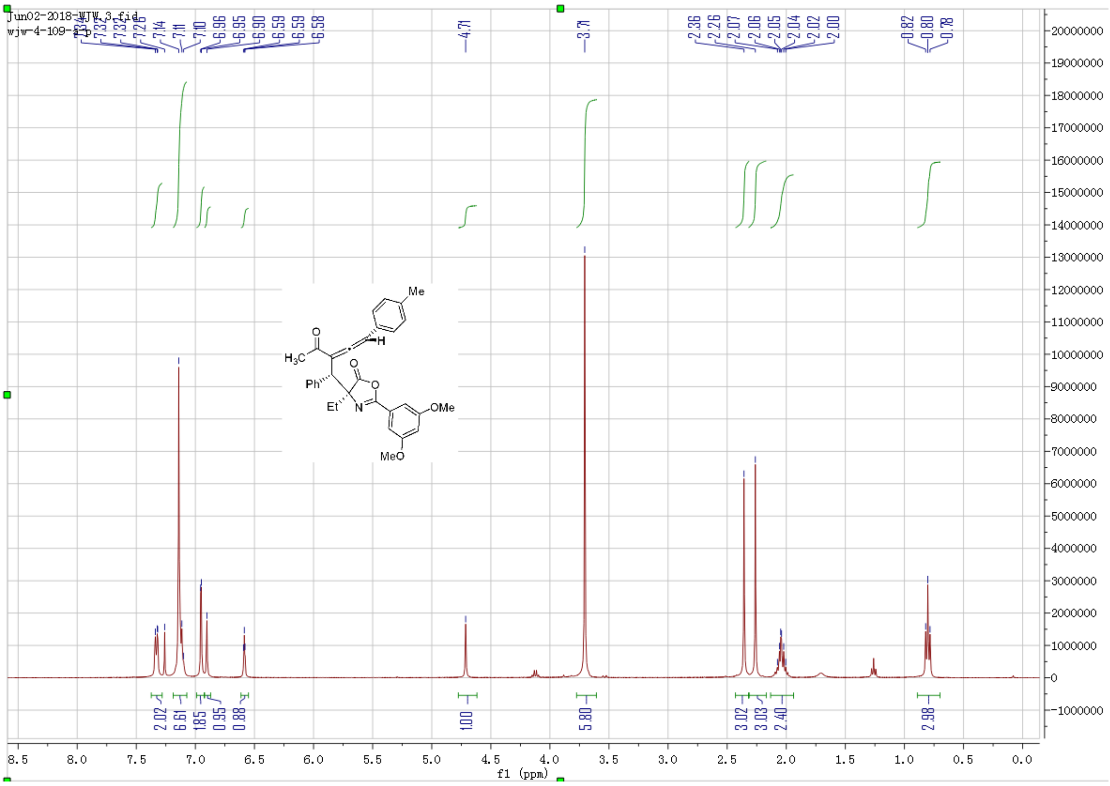

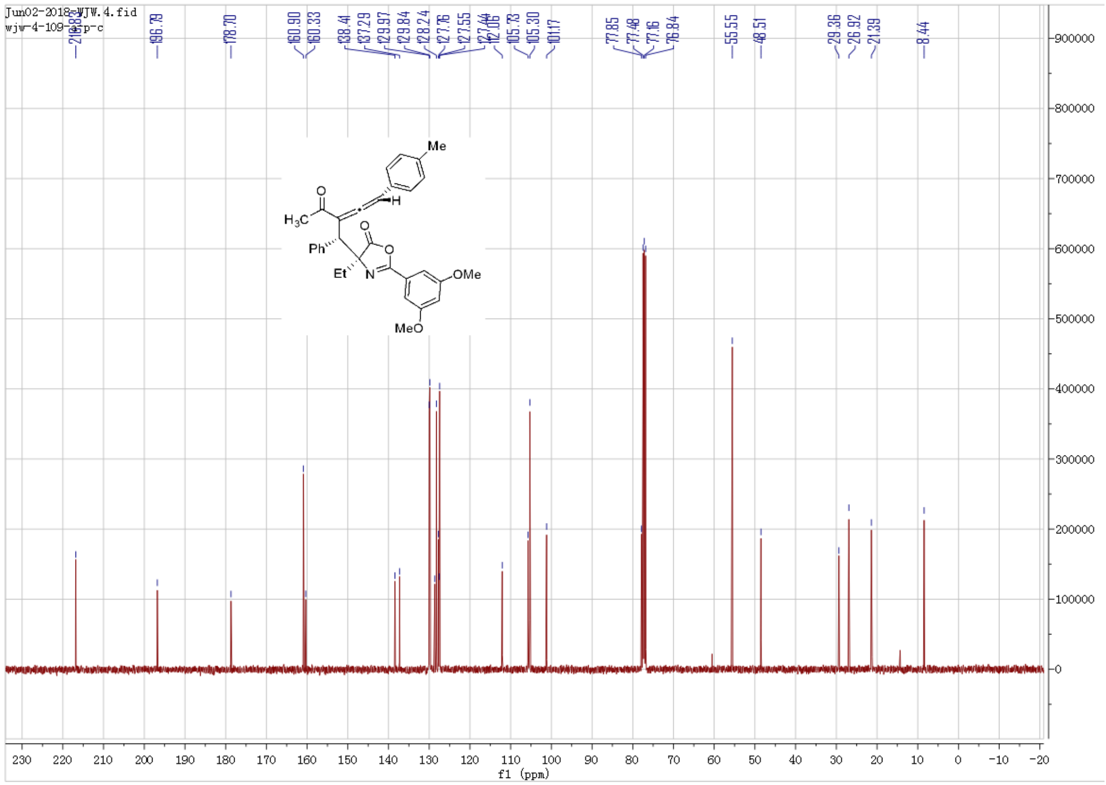

(*S*)-4-((*1R*,*3R*)-2-acetyl-4-(4-chlorophenyl)-1-phenylbuta-2,3-dien-1-yl)-2-(3,5-dimethoxyphenyl)-4-ethyloxazol-5(4H)-one (**3c**)

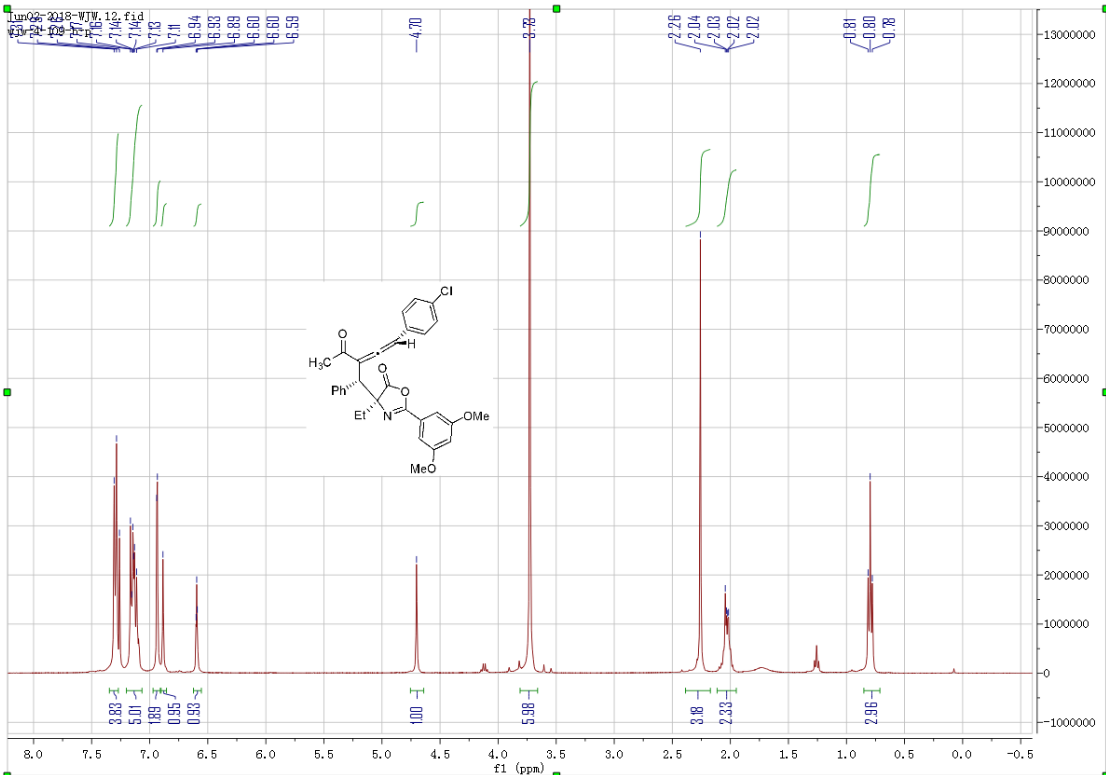

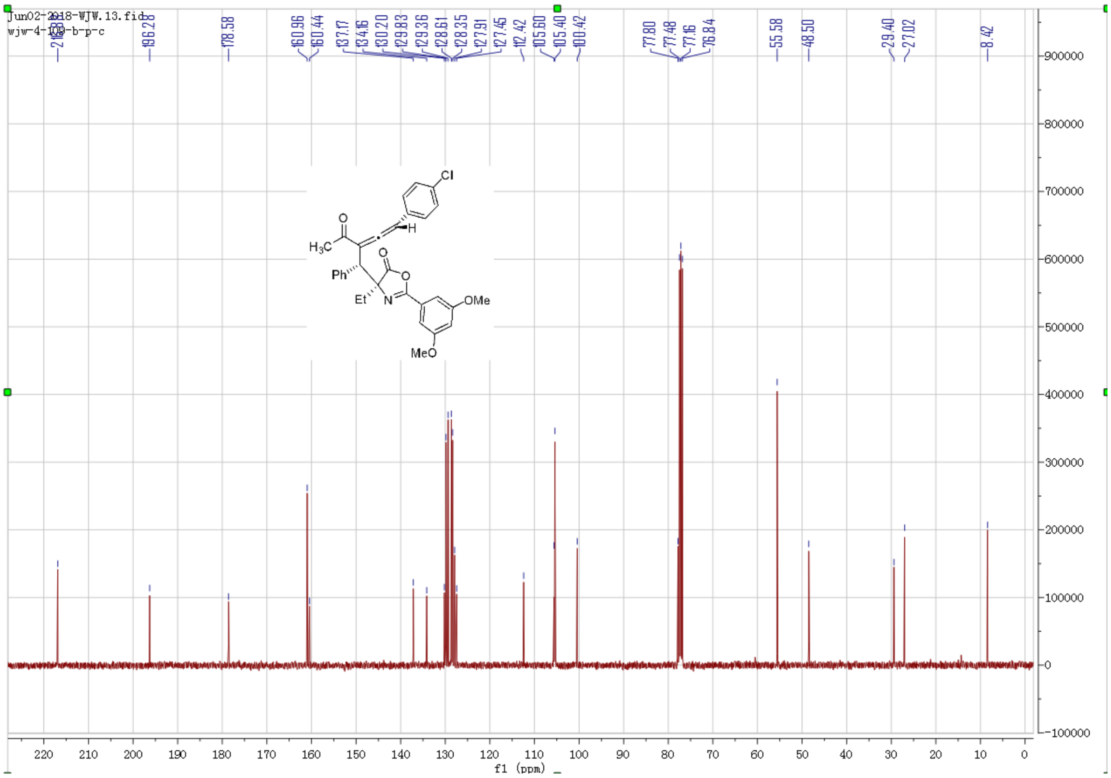

(*S*)-4-((*1R*,*3R*)-2-acetyl-4-(4-methoxyphenyl)-1-phenylbuta-2,3-dien-1-yl)-2-(3,5-dimethoxyphenyl)-4-ethyloxazol-5(4H)-one (**3d**)

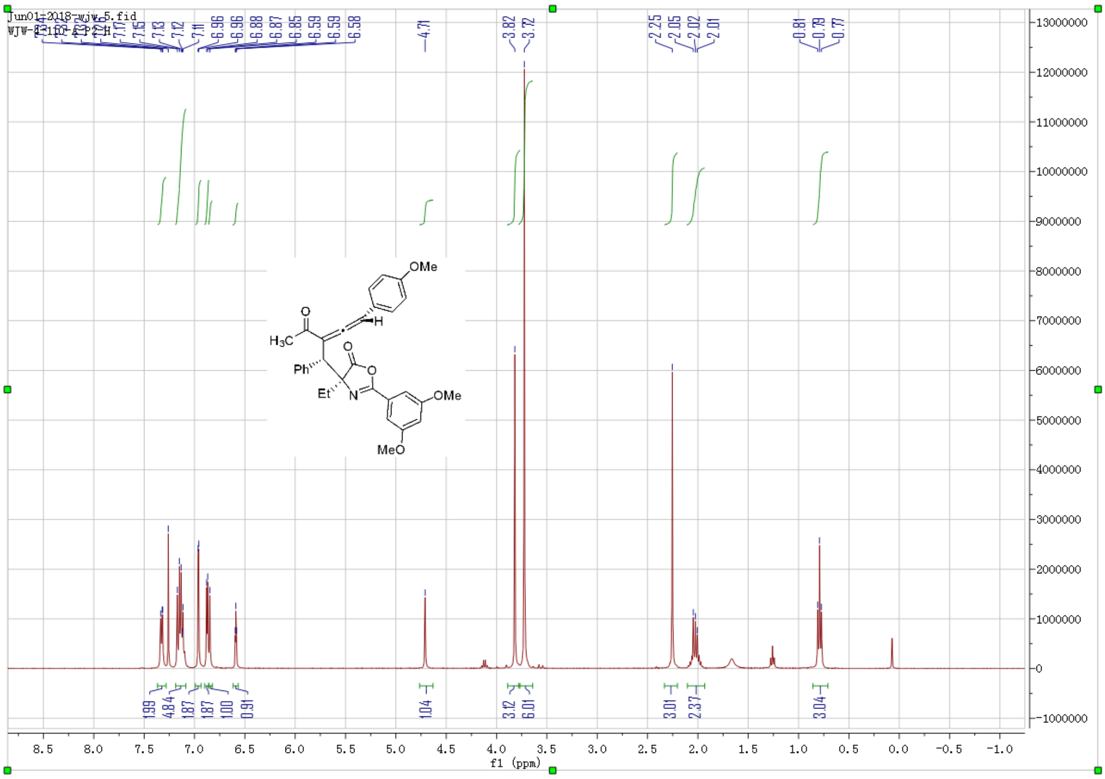

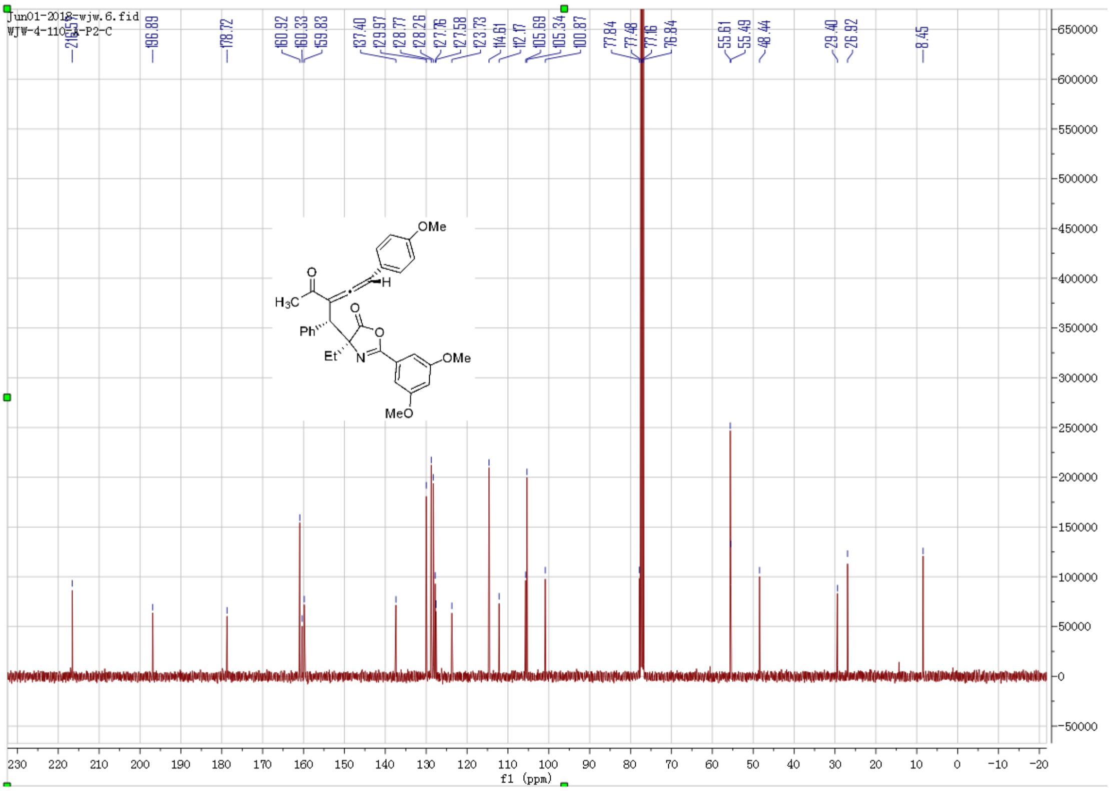

(*S*)-4-((*1R*,*3R*)-2-acetyl-4-(4-fluorophenyl)-1-phenylbuta-2,3-dien-1-yl)-2-(3,5-dimethoxyphenyl)-4-ethyloxazol-5(4H)-one (**3e**)

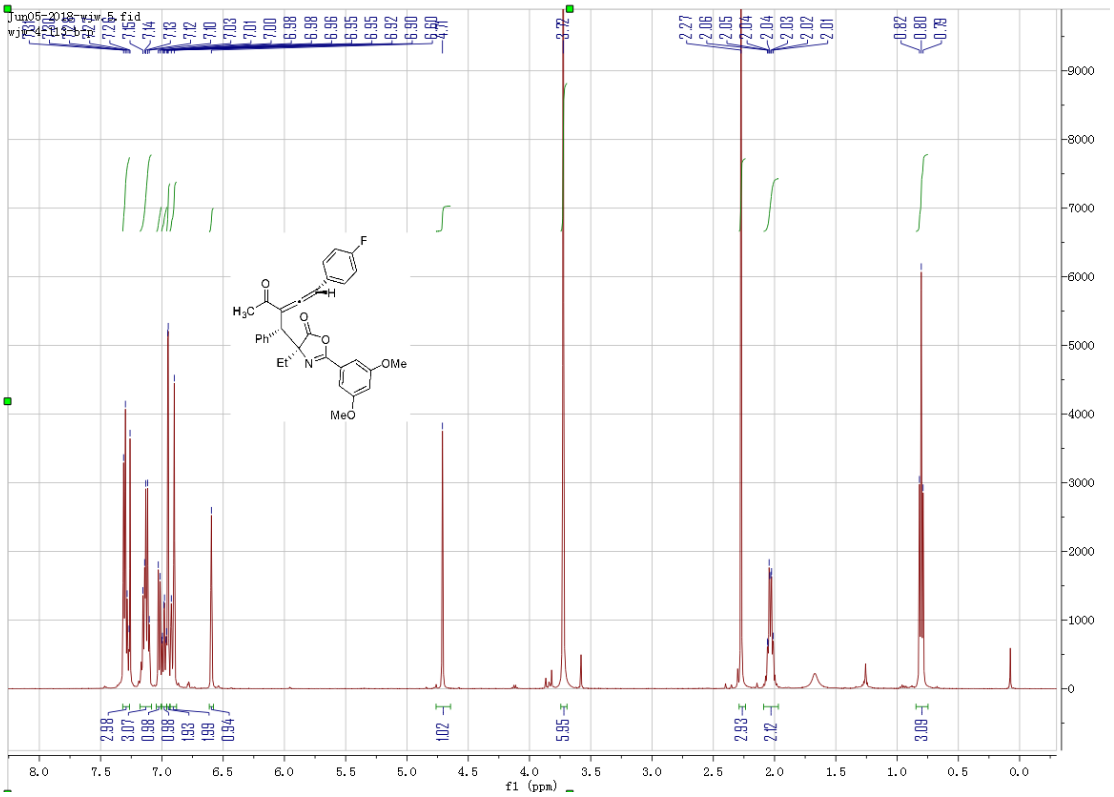

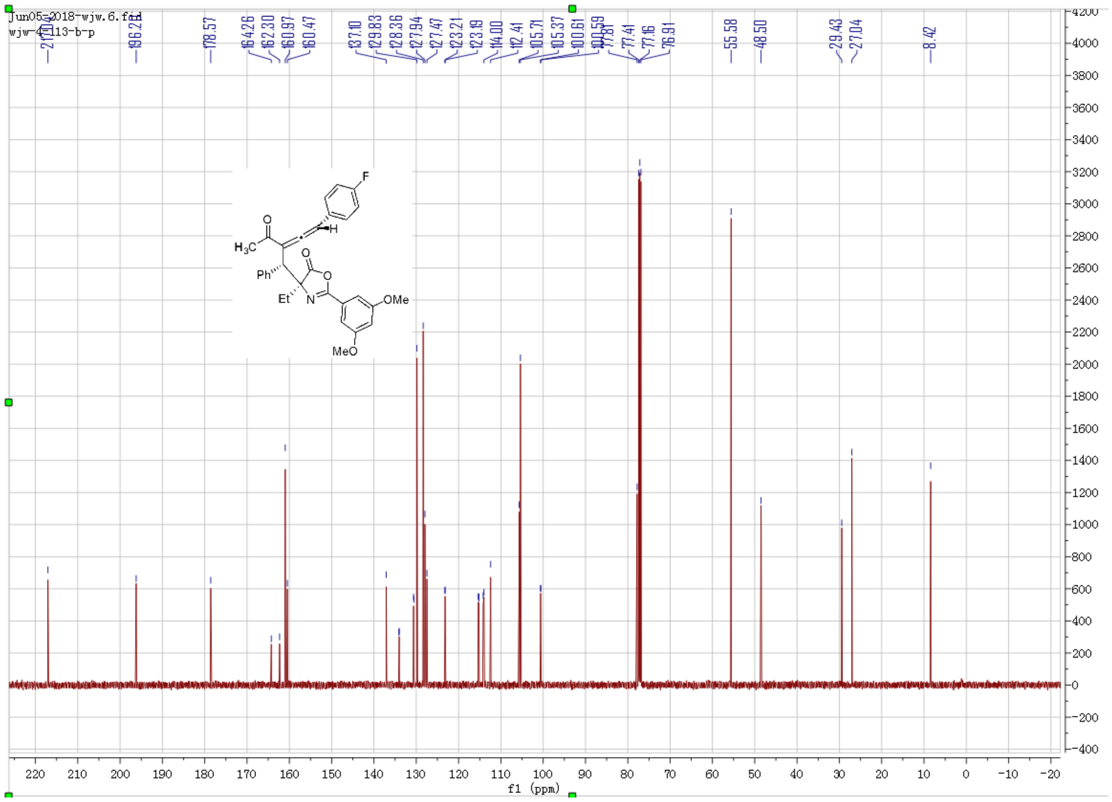

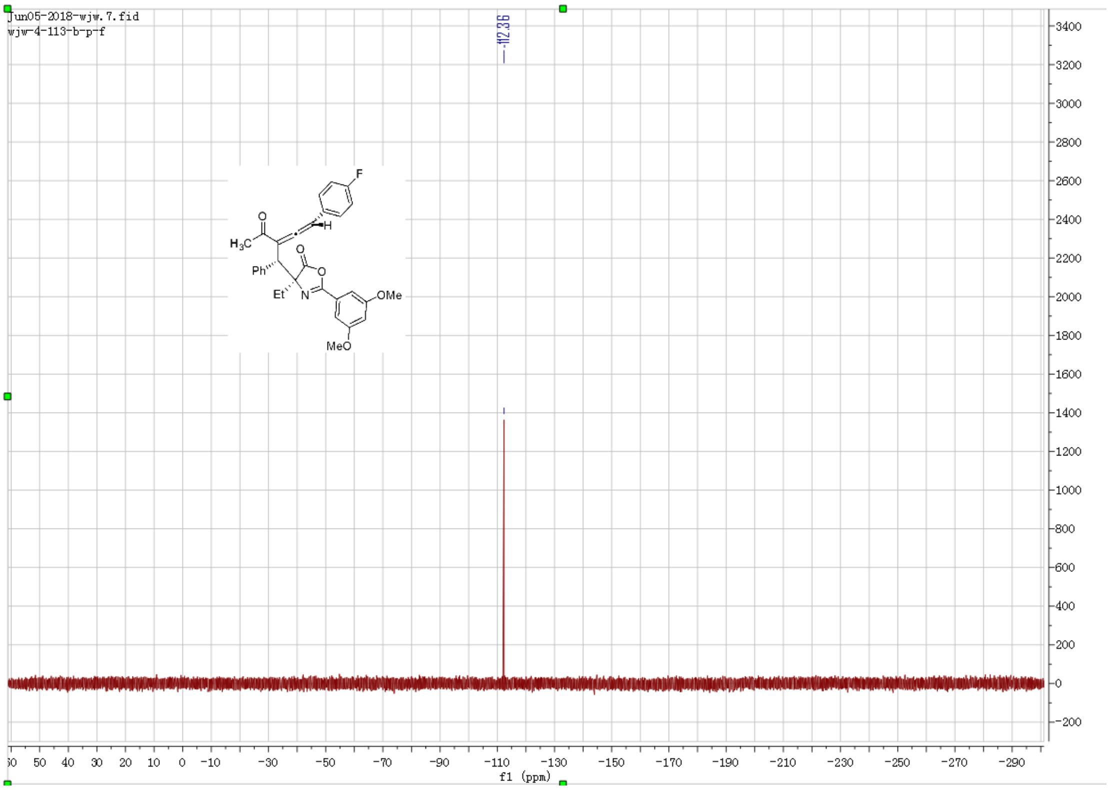

Jun05-2018-wjw.7.fid
wjw-4-113-b-p-f
-112.36
F
O
H3C
H
O
O
Ph
Et
N
OMe
MeO
f1 (ppm)

(*S*)-4-((*1R*,*3R*)-2-acetyl-1-phenyl-4-(4-(trifluoromethyl)phenyl)buta-2,3-dien-1-yl)-2-(3,5-dimethoxyphenyl)-4-ethyloxazol-5(4H)-one (**3f**)

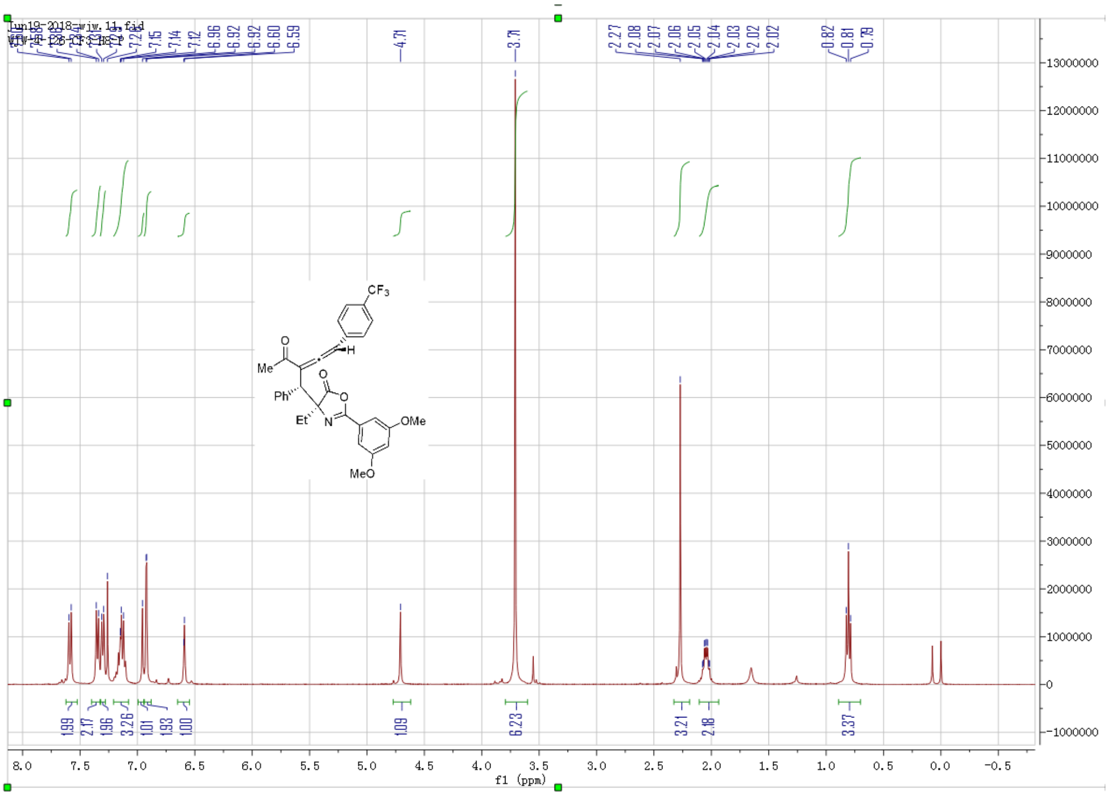

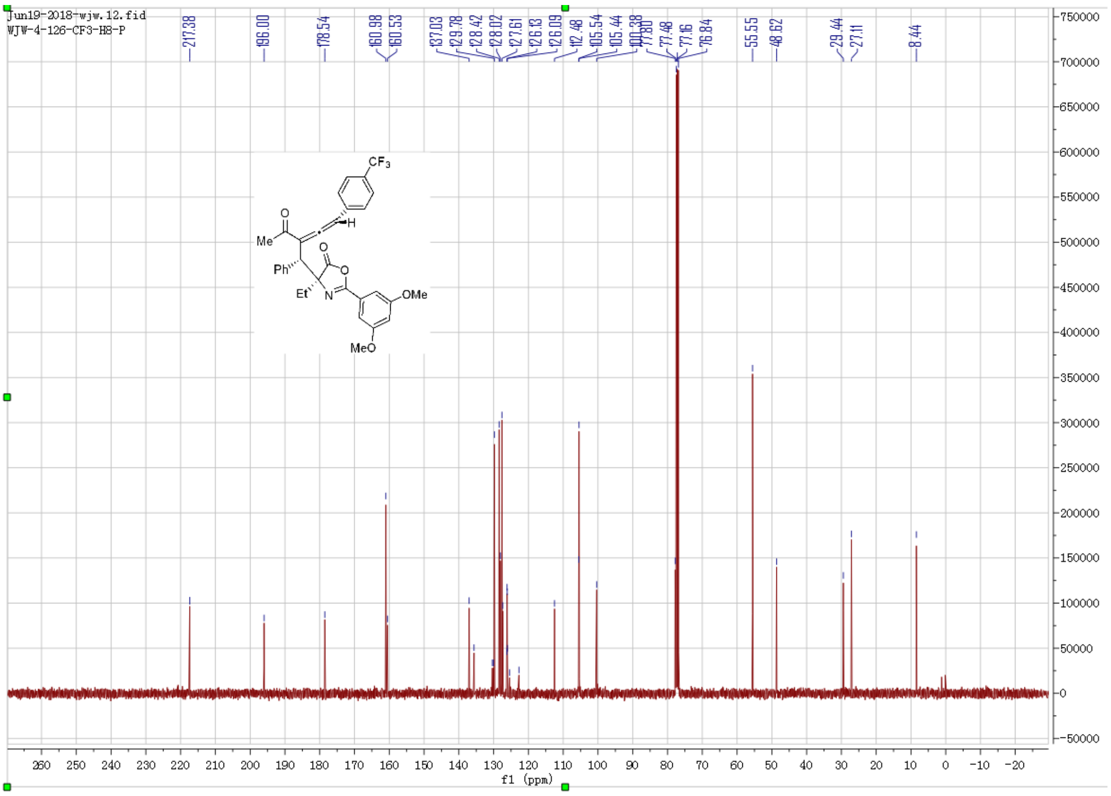

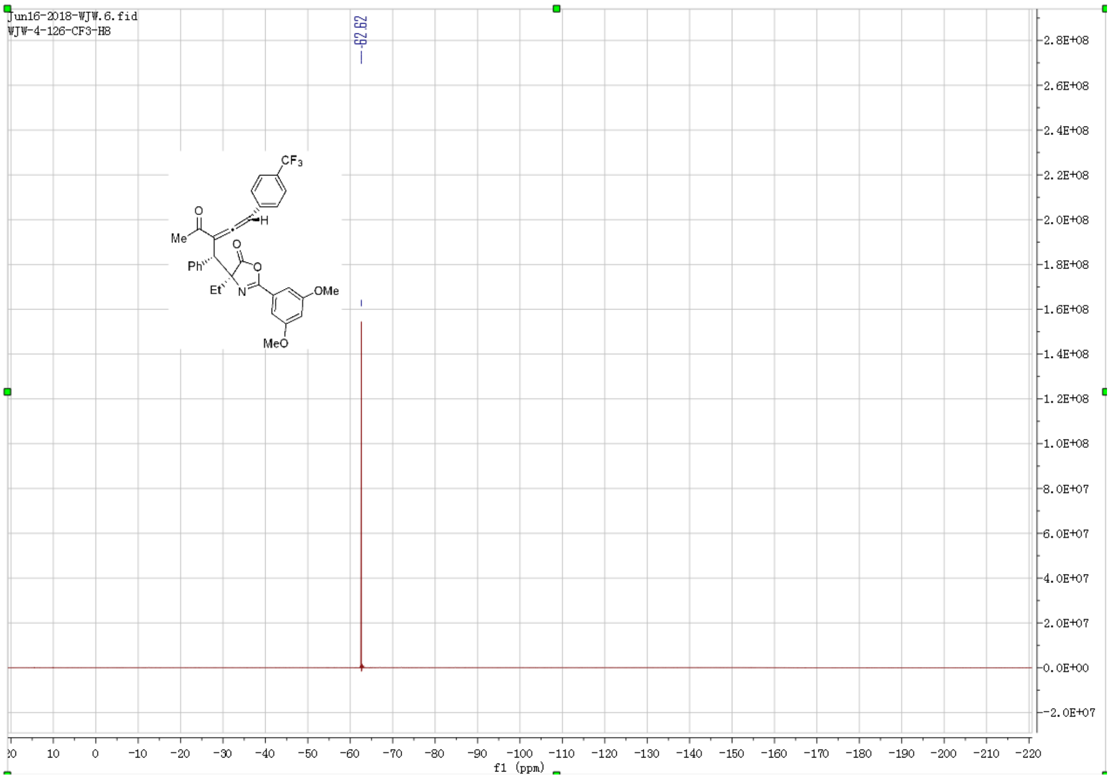
Jun16-2018-WJW.6.fid
WJW-4-126-CF3-H8
-62.62
CF3
O
Me
H
Ph
O
O
Et
N
OMe
MeO
2.8E+08
2.6E+08
2.4E+08
2.2E+08
2.0E+08
1.8E+08
1.6E+08
1.4E+08
1.2E+08
1.0E+08
8.0E+07
6.0E+07
4.0E+07
2.0E+07
0.0E+00
-2.0E+07
20 10 0 -10 -20 -30 -40 -50 -60 -70 -80 -90 -100 -110 -120 -130 -140 -150 -160 -170 -180 -190 -200 -210 -220
f1 (ppm)

Methyl3-((*R*)-3-((*R*)-((*S*)-2-(3,5-dimethoxyphenyl)-4-ethyl-5-oxo-4,5-dihydrooxazol-4-yl)(phenyl)methyl)-4-oxopenta-1,2-dien-1-yl)benzoate (**3g**)

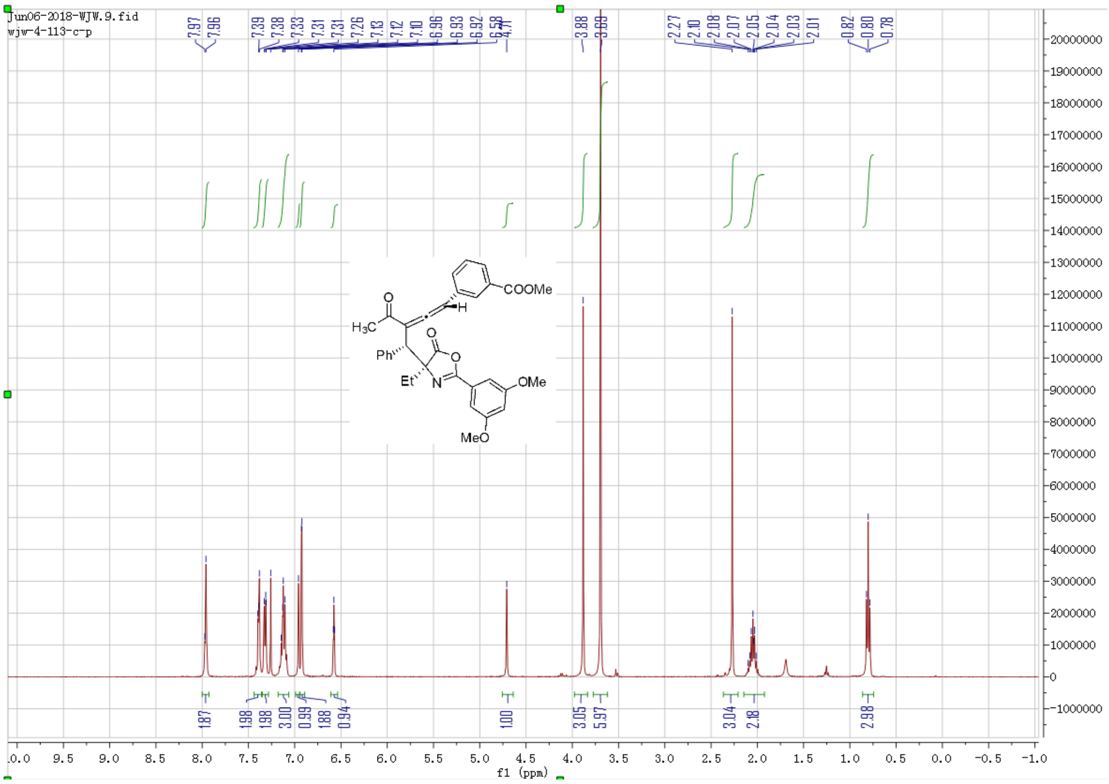

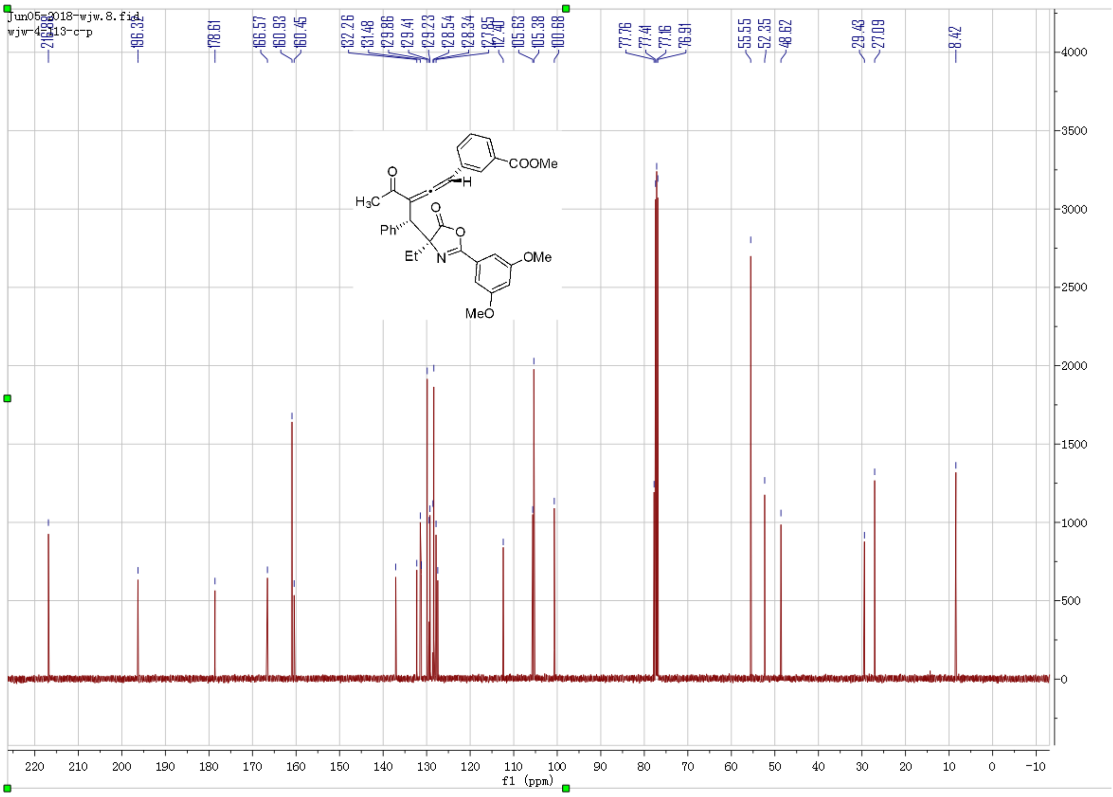

(*S*)-4-((*1R*,*3R*)-2-acetyl-4-(2-chlorophenyl)-1-phenylbuta-2,3-dien-1-yl)-2-(3,5-dimethoxyphenyl)-4-ethyloxazol-5(4H)-one **(3h)**

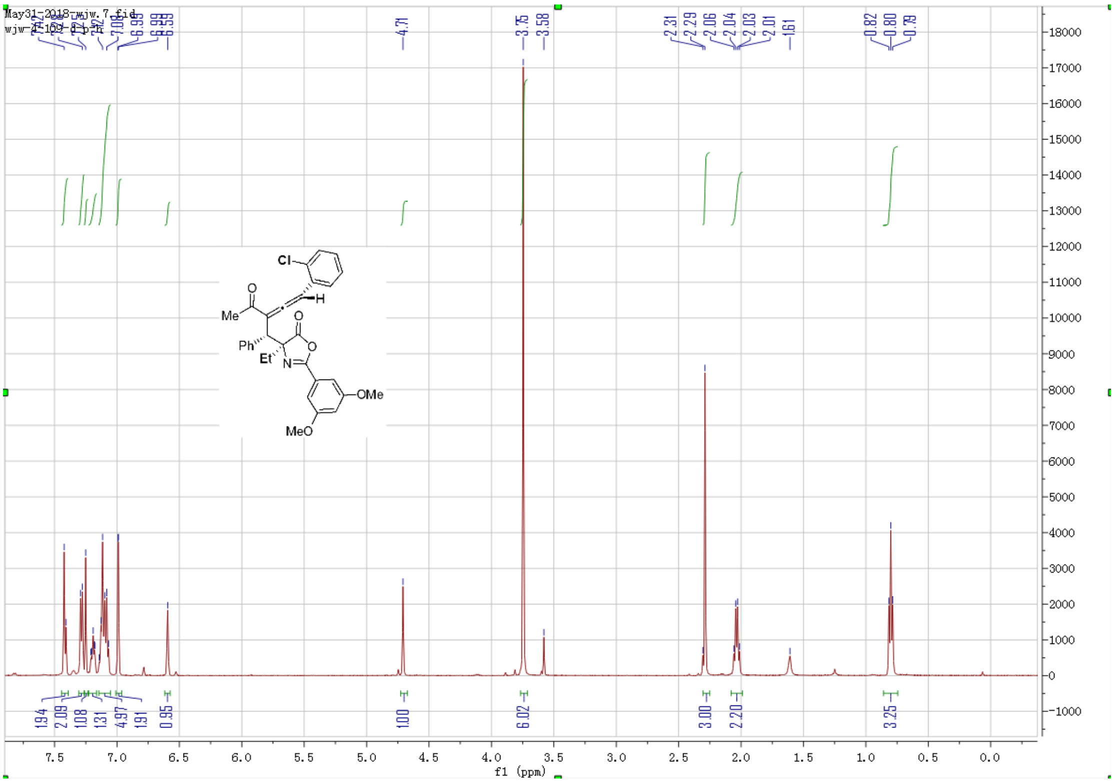

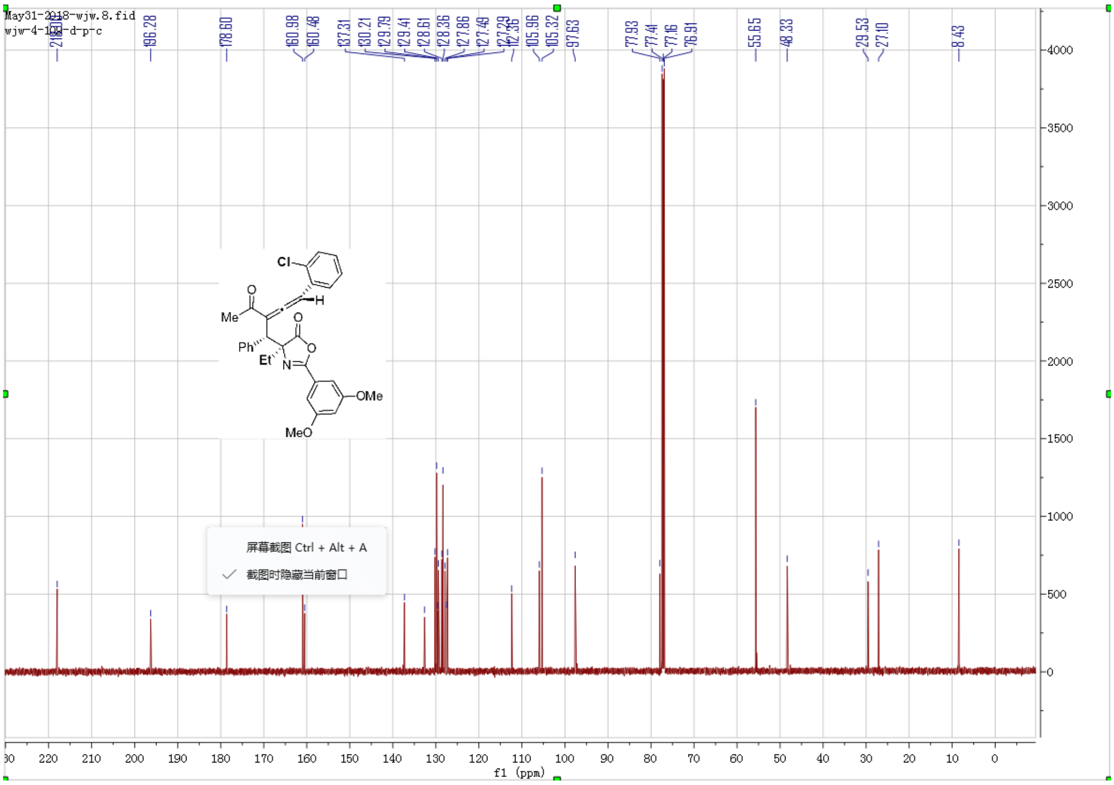

(*S*)-4-((*1R*,*3R*)-2-acetyl-1-phenyl-4-(thiophen-3-yl)buta-2,3-dien-1-yl)-2-(3,5-dimethoxyphenyl)-4-ethyloxazol-5(4H)-one (**3i**)

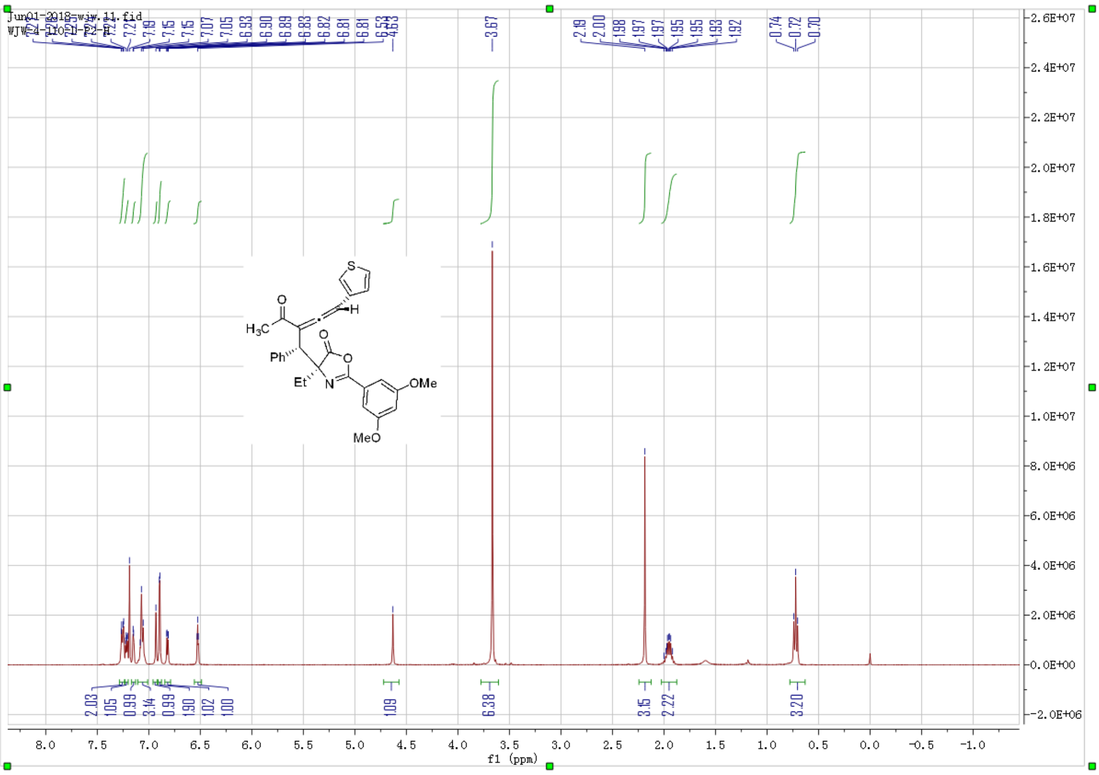

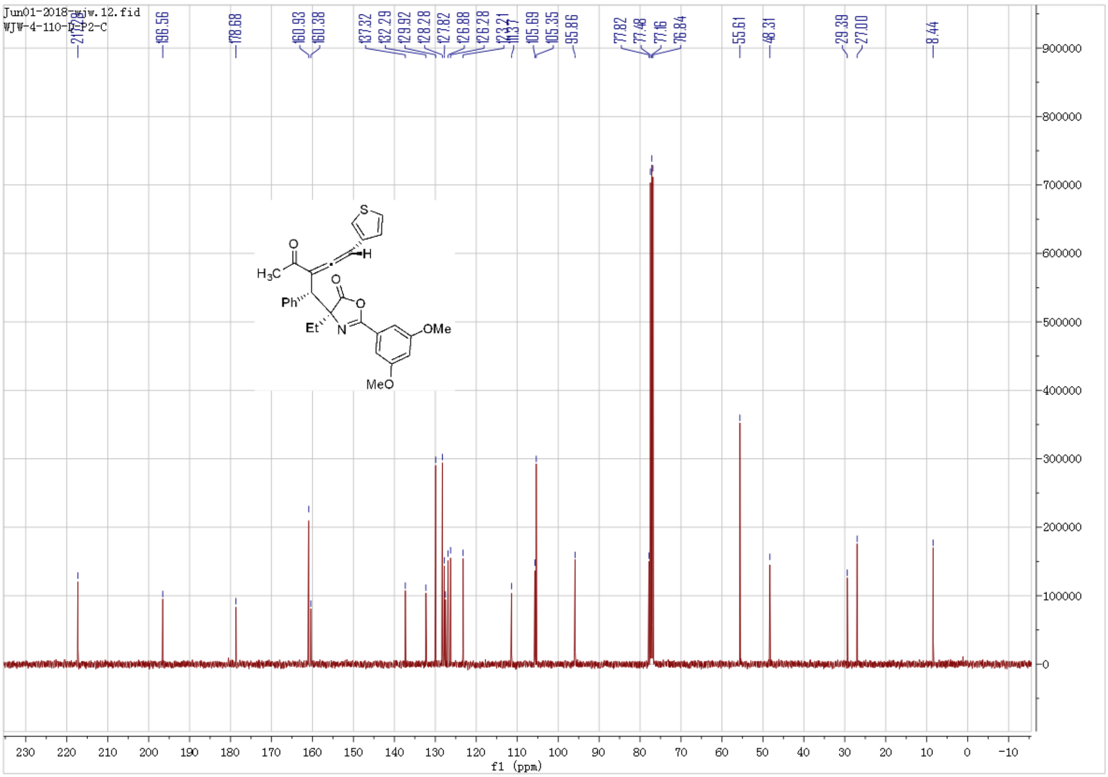

(*S*)-4-((*1R*,*3R*)-2-acetyl-4-(cyclohex-1-en-1-yl)-1-phenylbuta-2,3-dien-1-yl)-2-(3,5-dimethoxyphenyl)-4-ethyloxazol-5(4H)-one (**3j**)

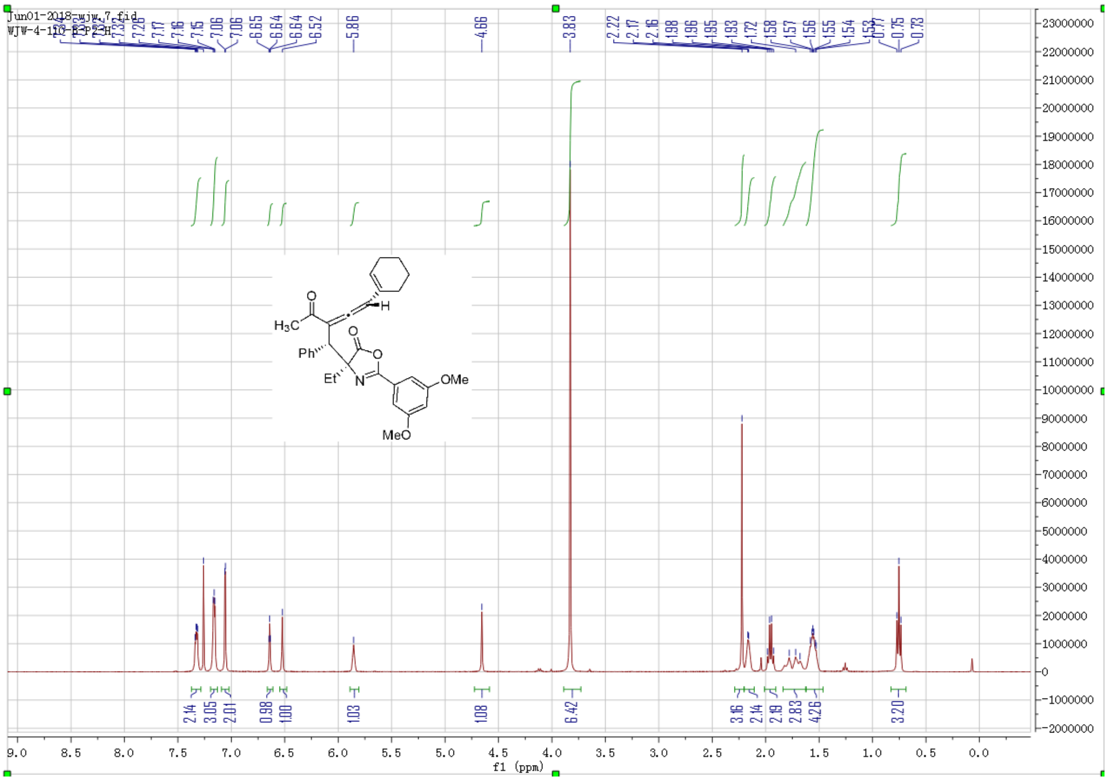

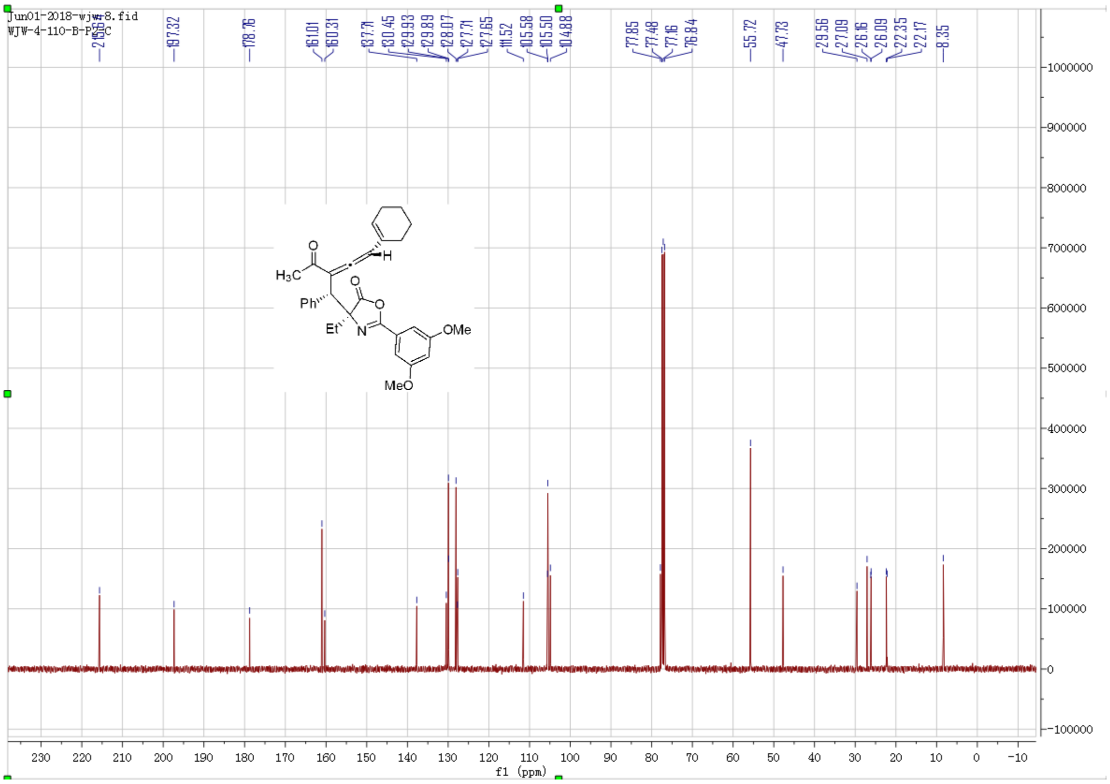

(*S*)-4-((*1R*,*3R*)-2-acetyl-1-phenylocta-2,3-dien-1-yl)-2-(3,5-dimethoxyphenyl)-4-ethyloxazol-5(4H)-one (**3k**)

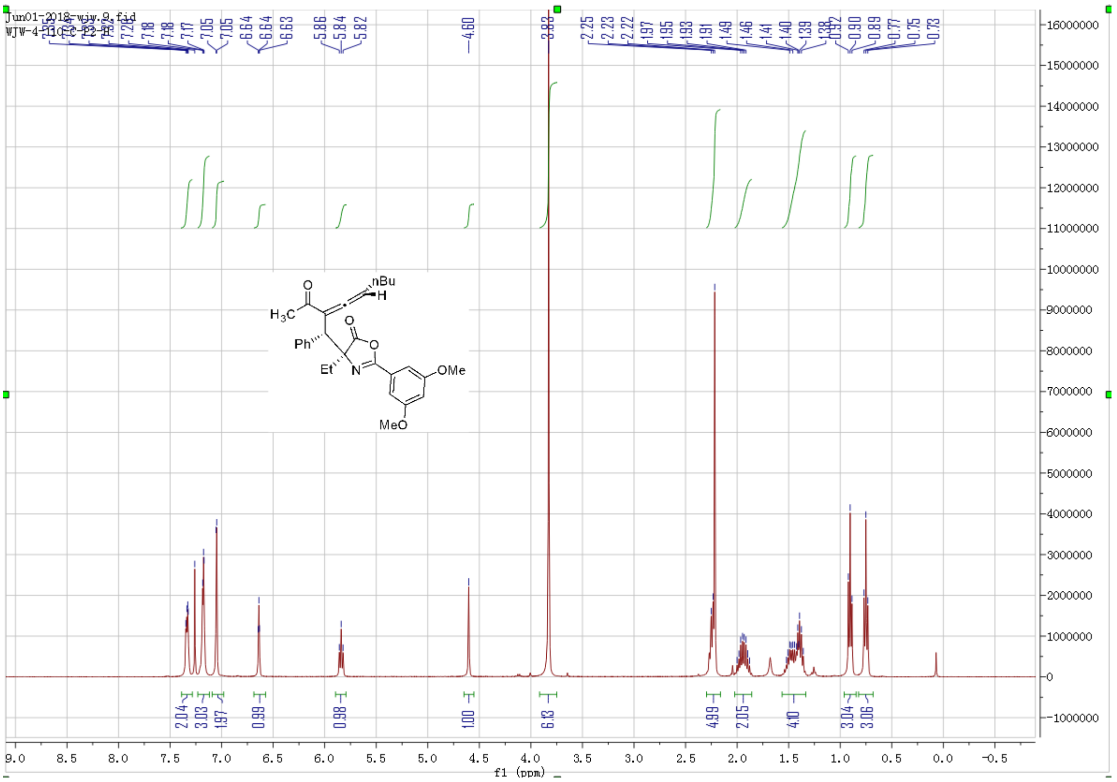

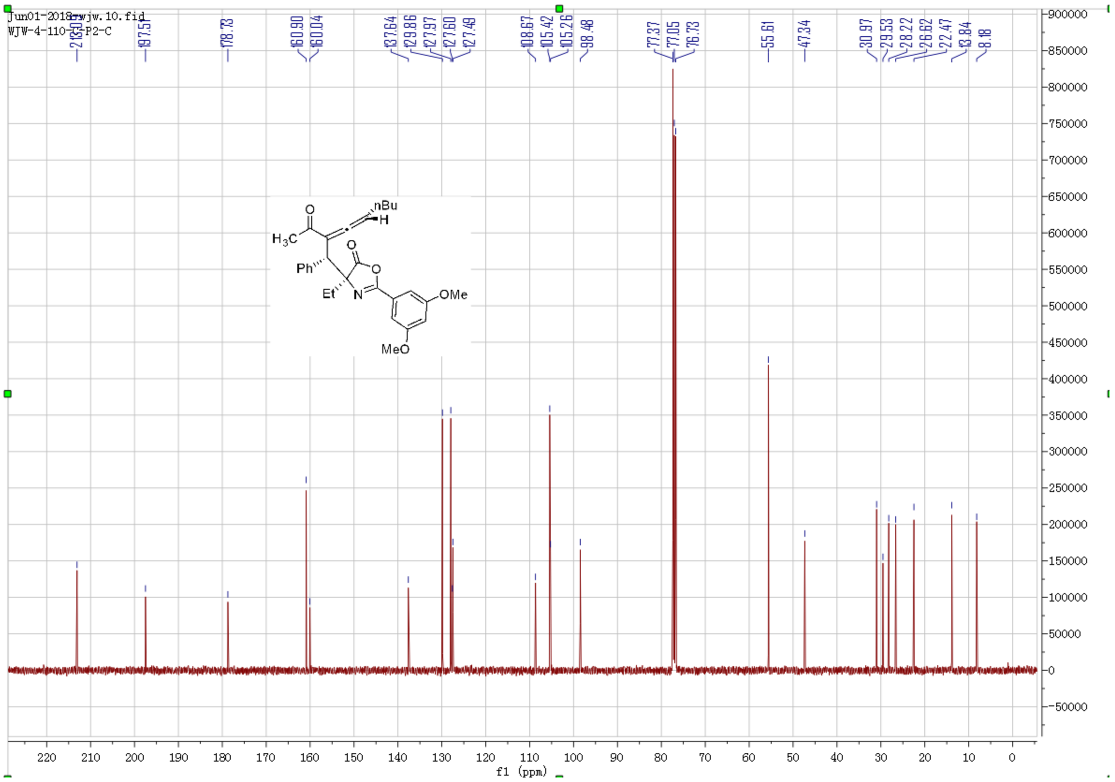

(*S*)-4-((*1R*,*3R*)-2-acetyl-4-phenyl-1-(p-tolyl)buta-2,3-dien-1-yl)-2-(3,5-dimethoxyphenyl)-4-ethyloxazol-5(4H)-one (**3l**)

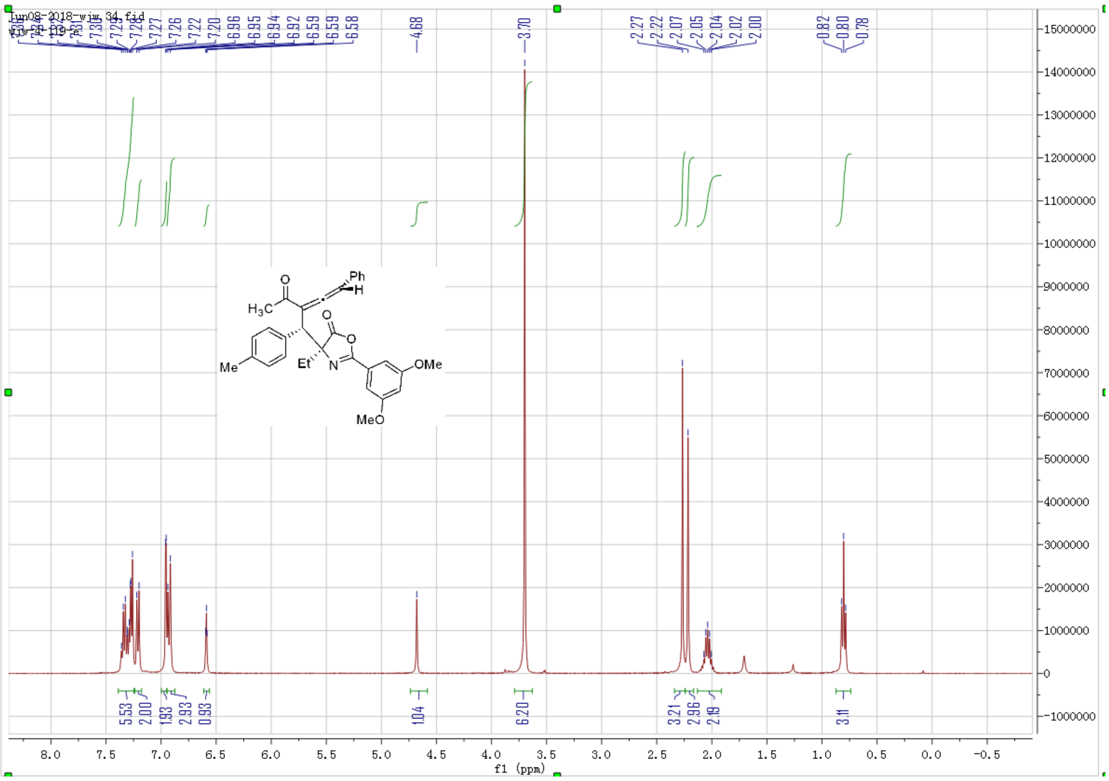

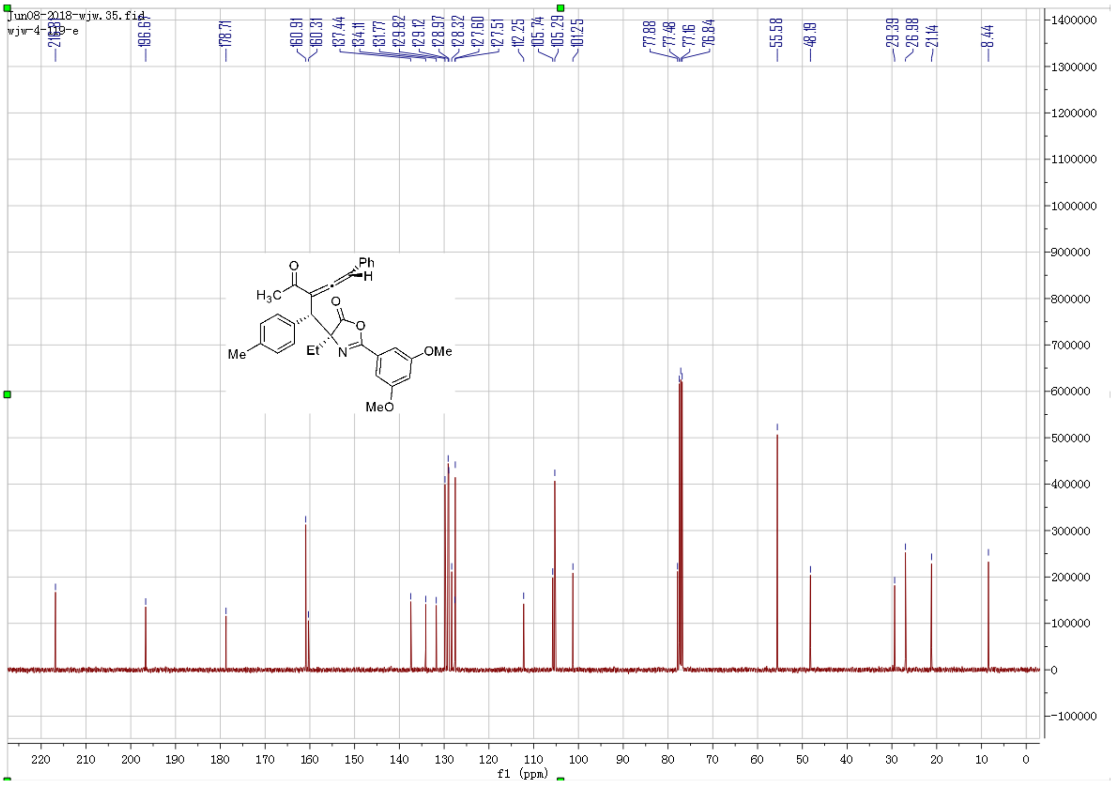

(*S*)-4-((*1R*,*3R*)-2-acetyl-1-(4-fluorophenyl)-4-phenylbuta-2,3-dien-1-yl)-2-(3,5-dimethoxyphenyl)-4-ethyloxazol-5(4H)-one (**3m**)

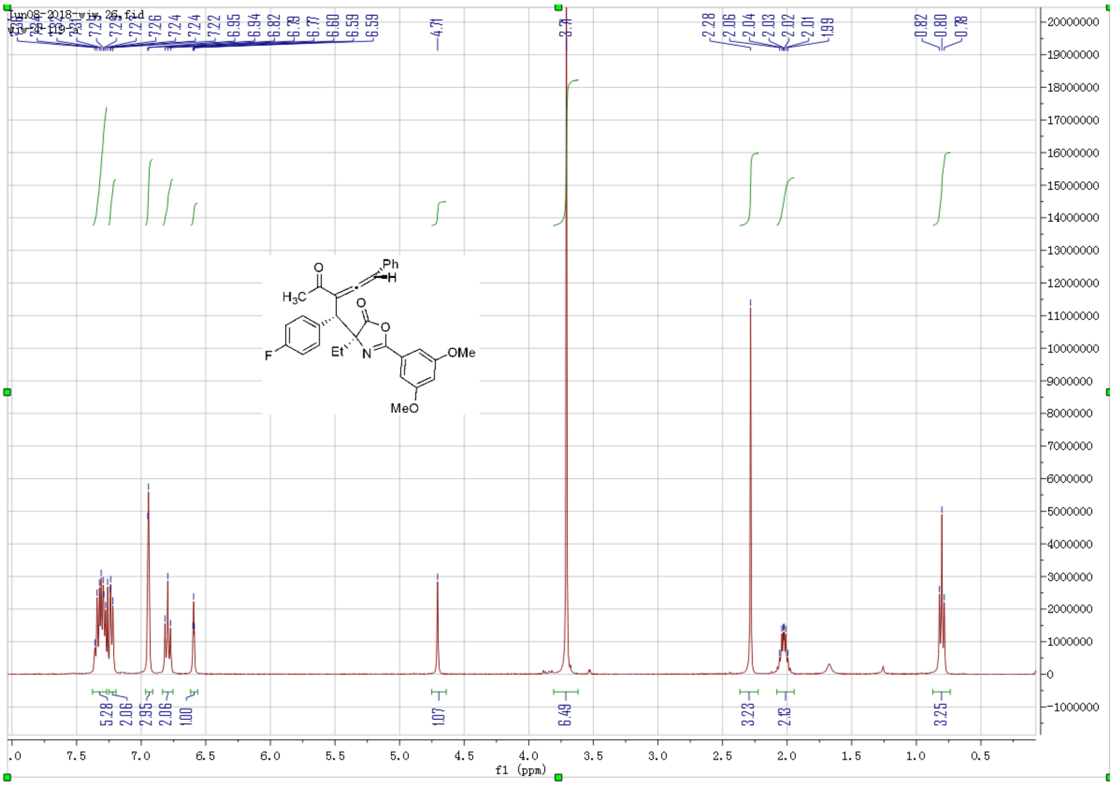

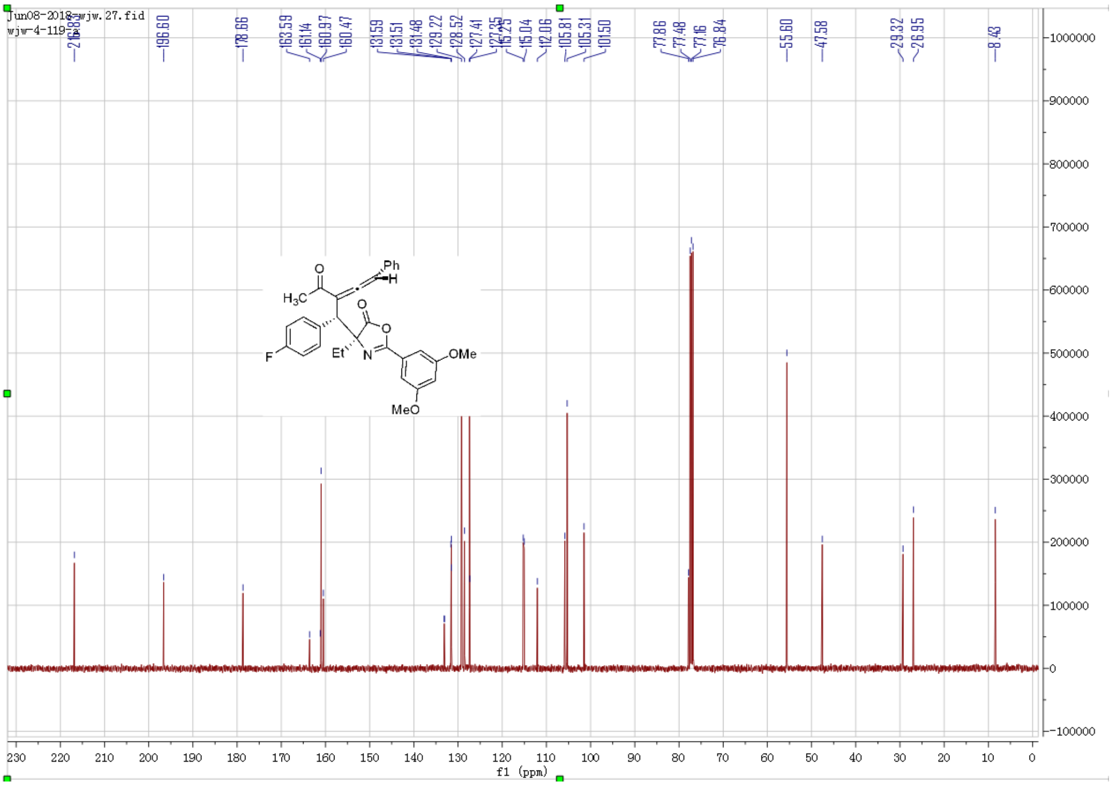

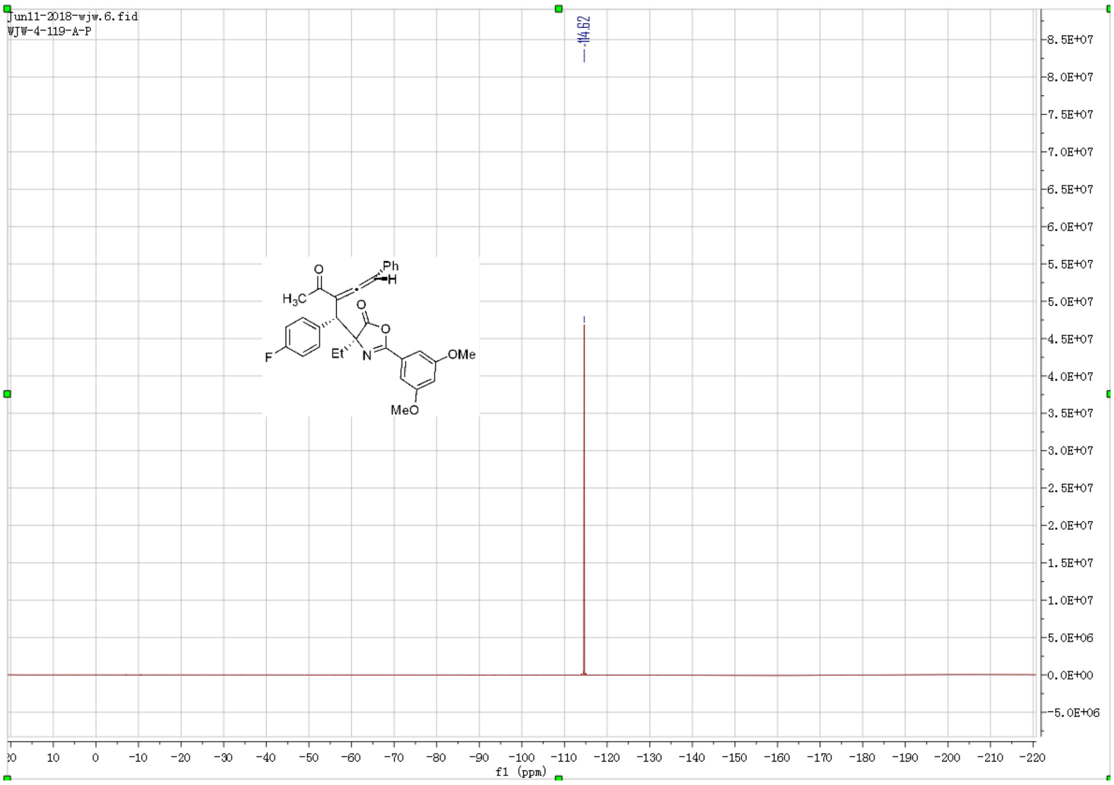
Jun11-2018-wjw.6.fid
WJW-4-119-A-P
-114.62
Ph
H
O
H3C
O
O
F
Et
N
OMe
MeO
f1 (ppm)

(*S*)-4-((*1R*,*3R*)-2-acetyl-1-(4-chlorophenyl)-4-phenylbuta-2,3-dien-1-yl)-2-(3,5-dimethoxyphenyl)-4-ethyloxazol-5(4H)-one (**3n**)

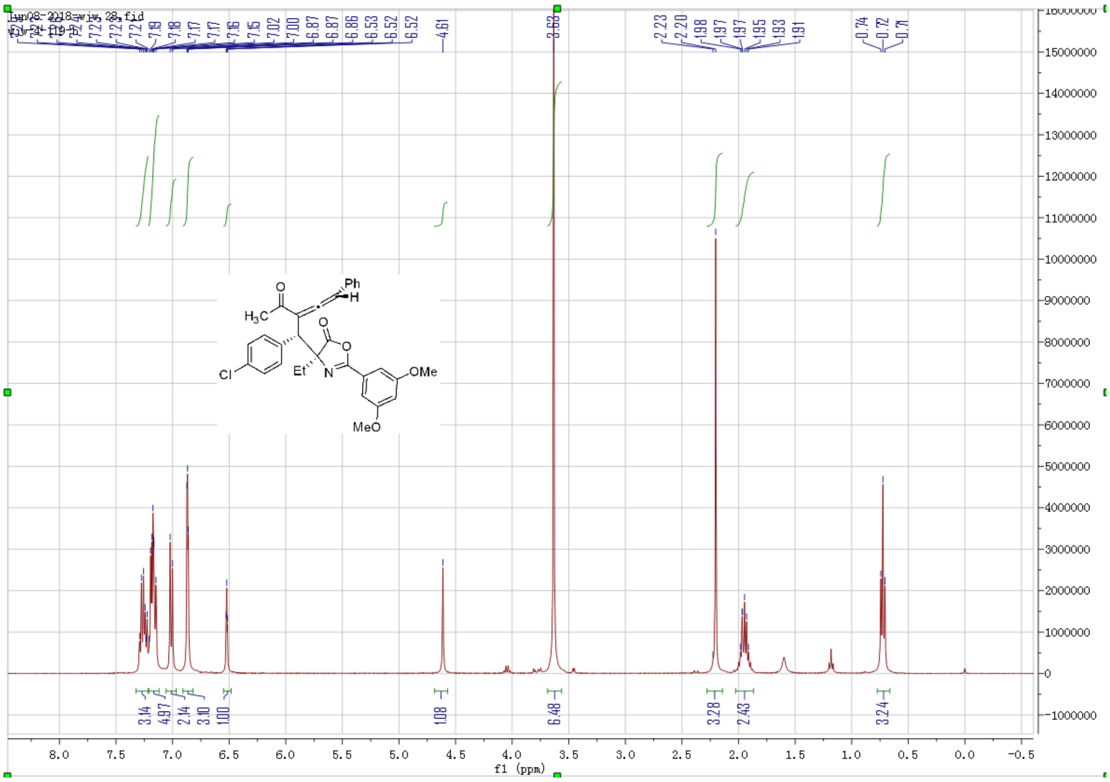

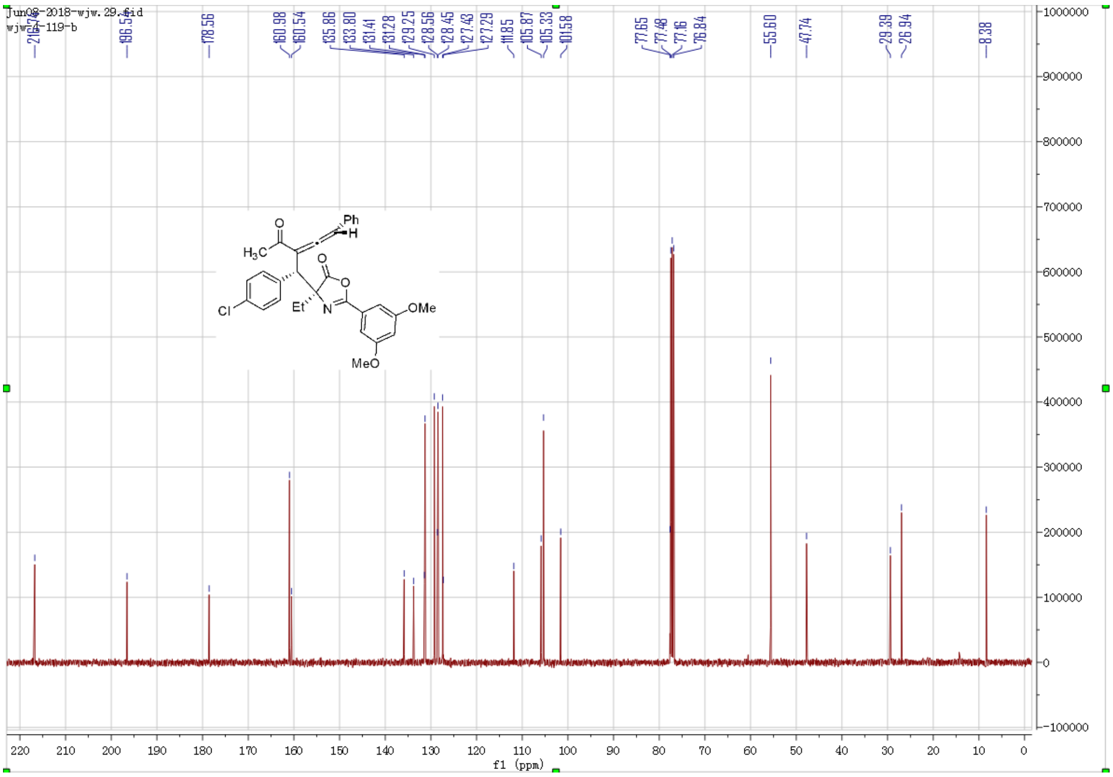

(*S*)-4-((*1R*,*3R*)-2-acetyl-1-(4-methoxyphenyl)-4-phenylbuta-2,3-dien-1-yl)-2-(3,5-dimethoxyphenyl)-4-ethyloxazol-5(4H)-one (**3o**)

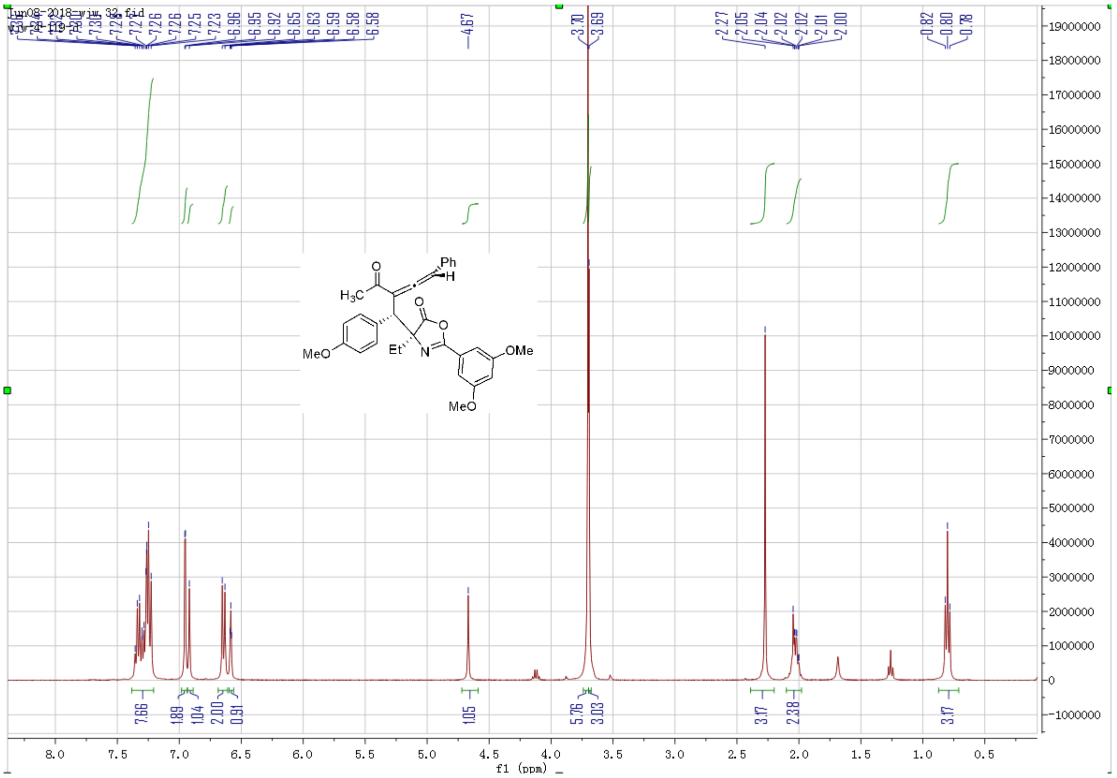

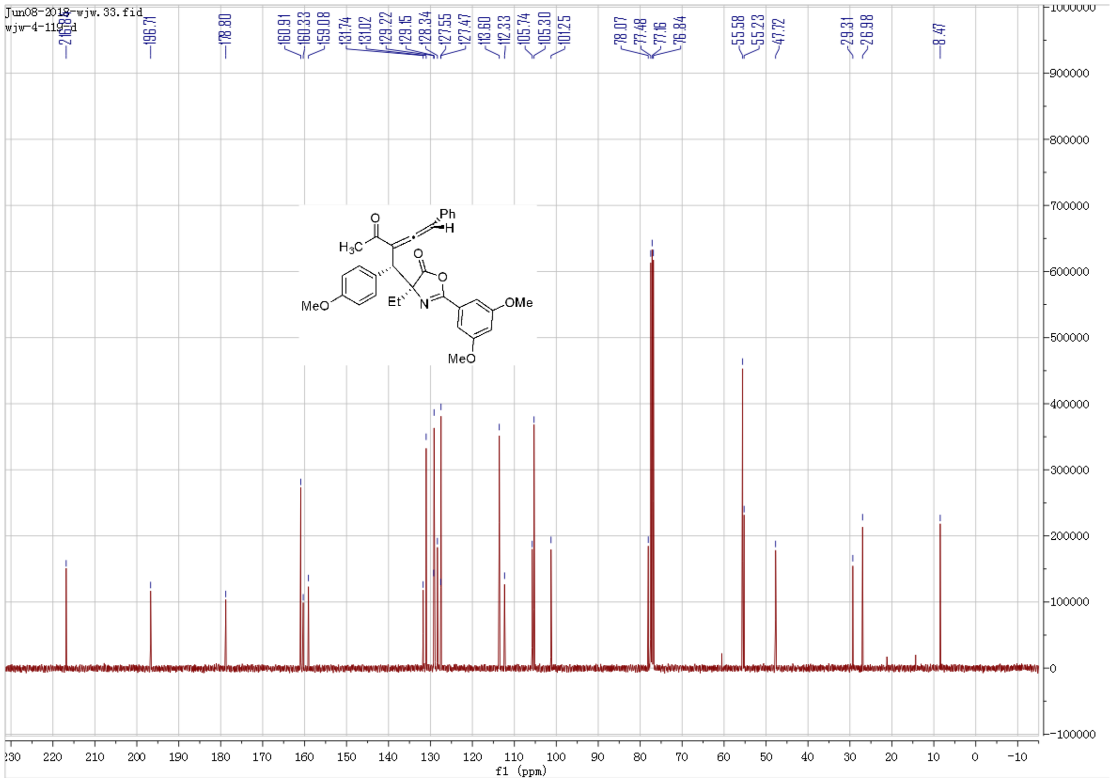

(*S*)-4-((*1R*,*3R*)-2-acetyl-1-(naphthalen-2-yl)-4-phenylbuta-2,3-dien-1-yl)-2-(3,5-dimethoxyphenyl)-4-ethyloxazol-5(4H)-one (**3p**)

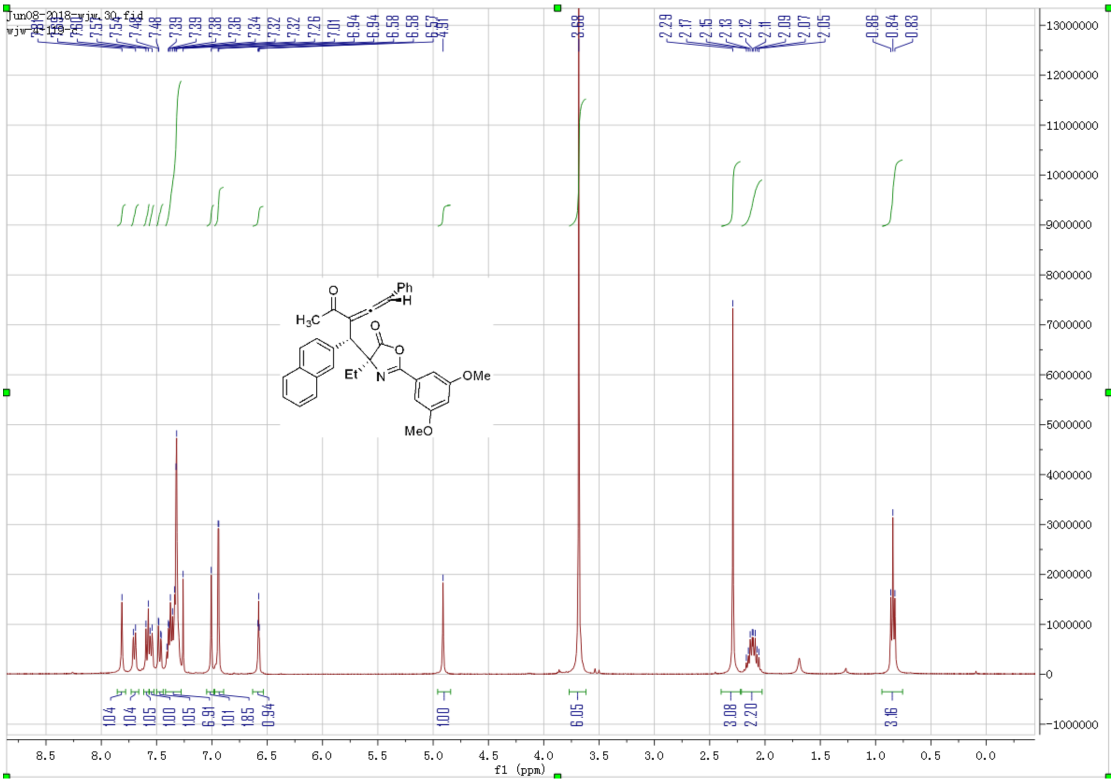

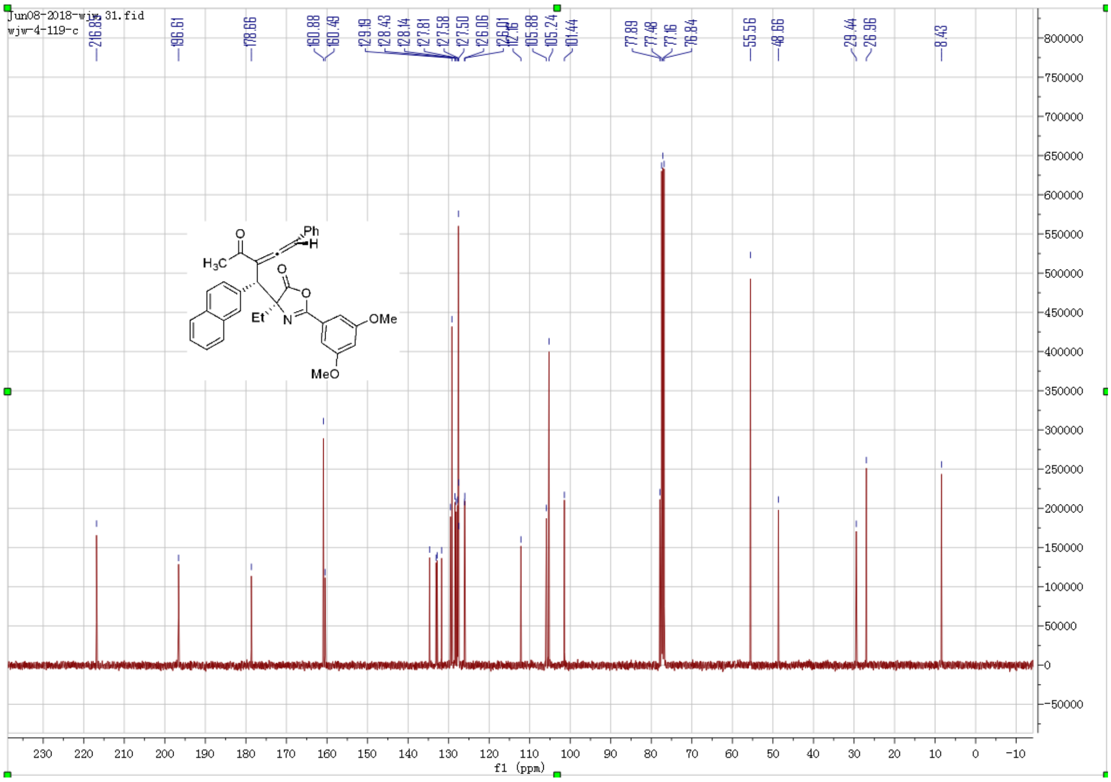

(*S*)-4-((*1R*,*3R*)-2-acetyl-1-cyclohexyl-4-phenylbuta-2,3-dien-1-yl)-2-(3,5-dimethoxyphenyl)-4-ethyloxazol-5(4H)-one (**3q**)

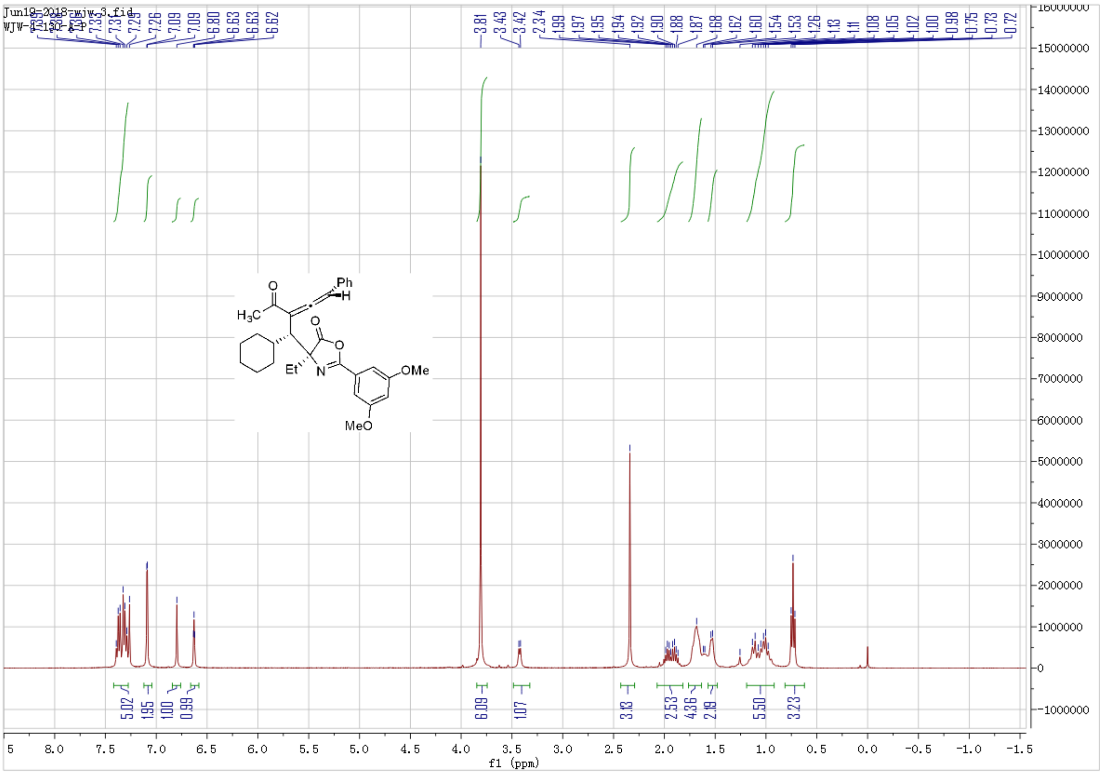

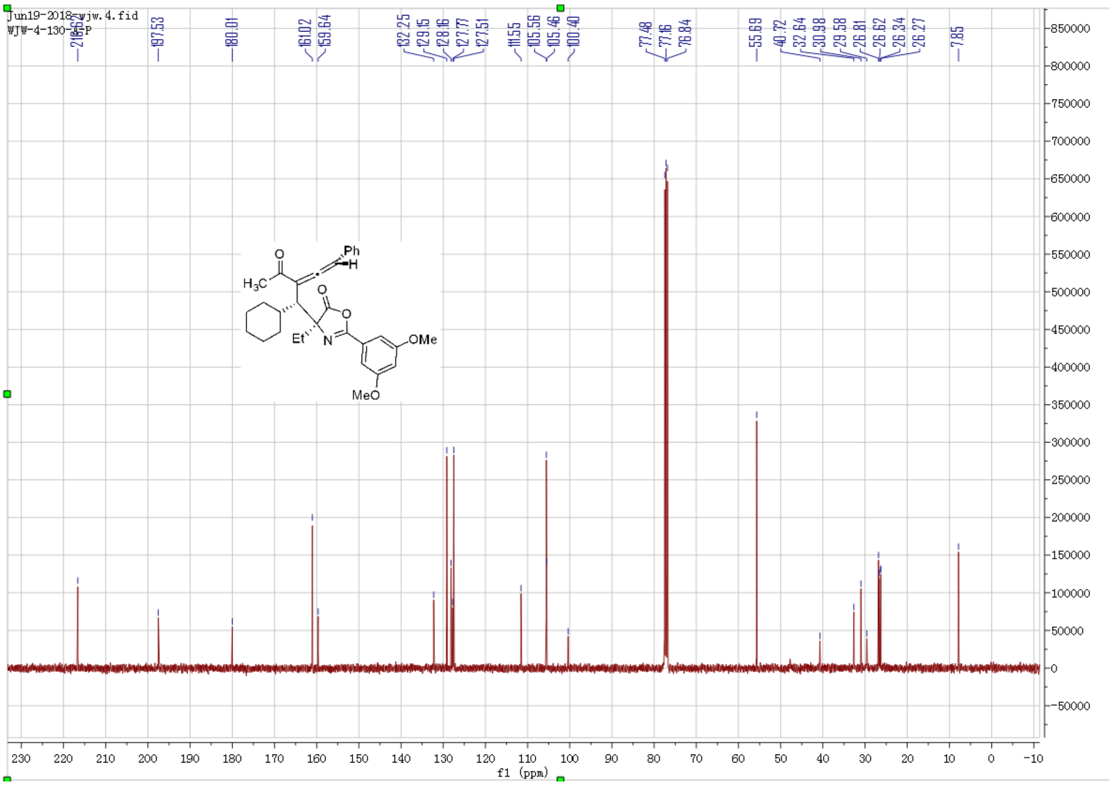

(*S*)-2-(3,5-dimethoxyphenyl)-4-ethyl-4-((*R*)-3-oxo-1-phenyl-2-((*R*)-2-phenylvinylidene)pentyl)oxazol-5(4H)-one (**3r**)

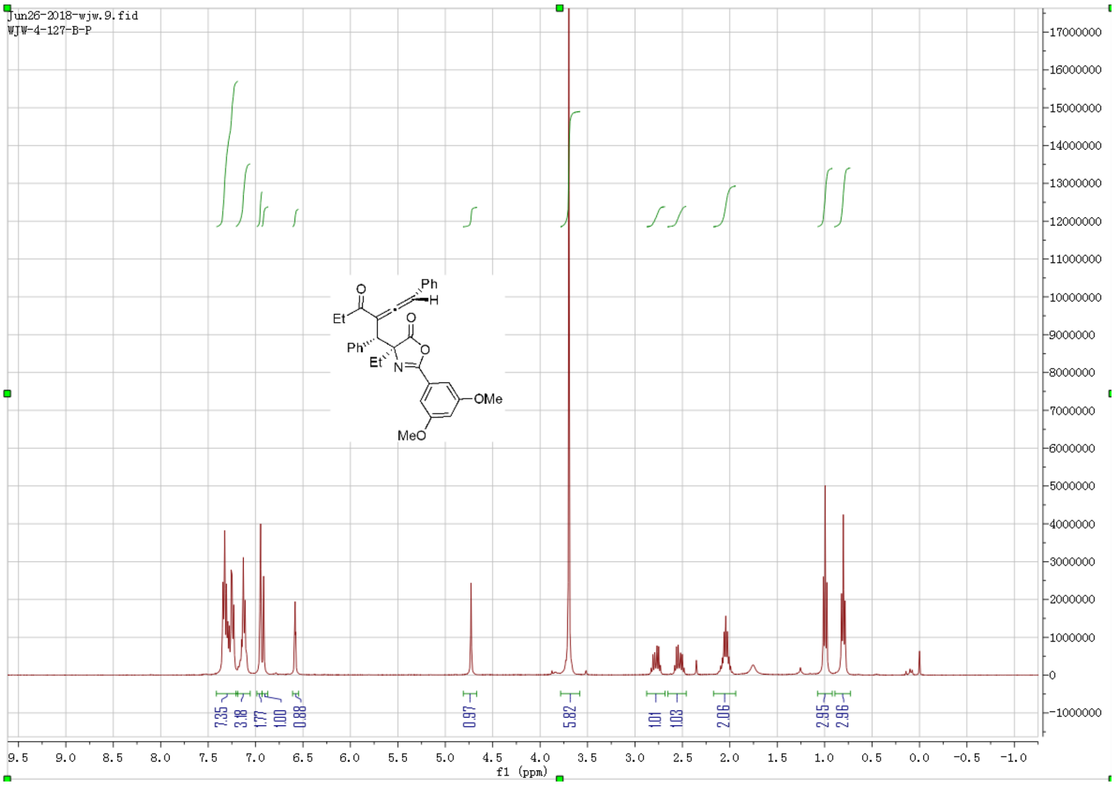

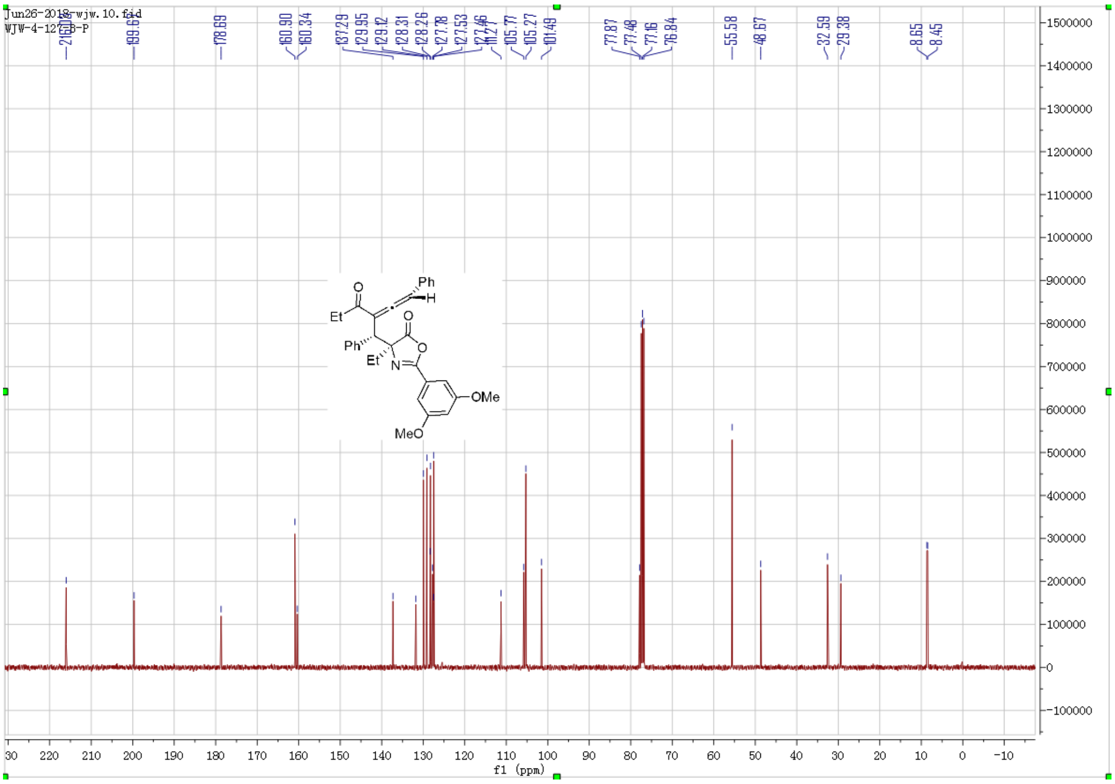

(*S*)-2-(3,5-dimethoxyphenyl)-4-ethyl-4-((*R*)-3-oxo-1-phenyl-2-((*R*)-2-phenylvinylidene)hexyl)oxazol-5(4H)-one (**3s**)

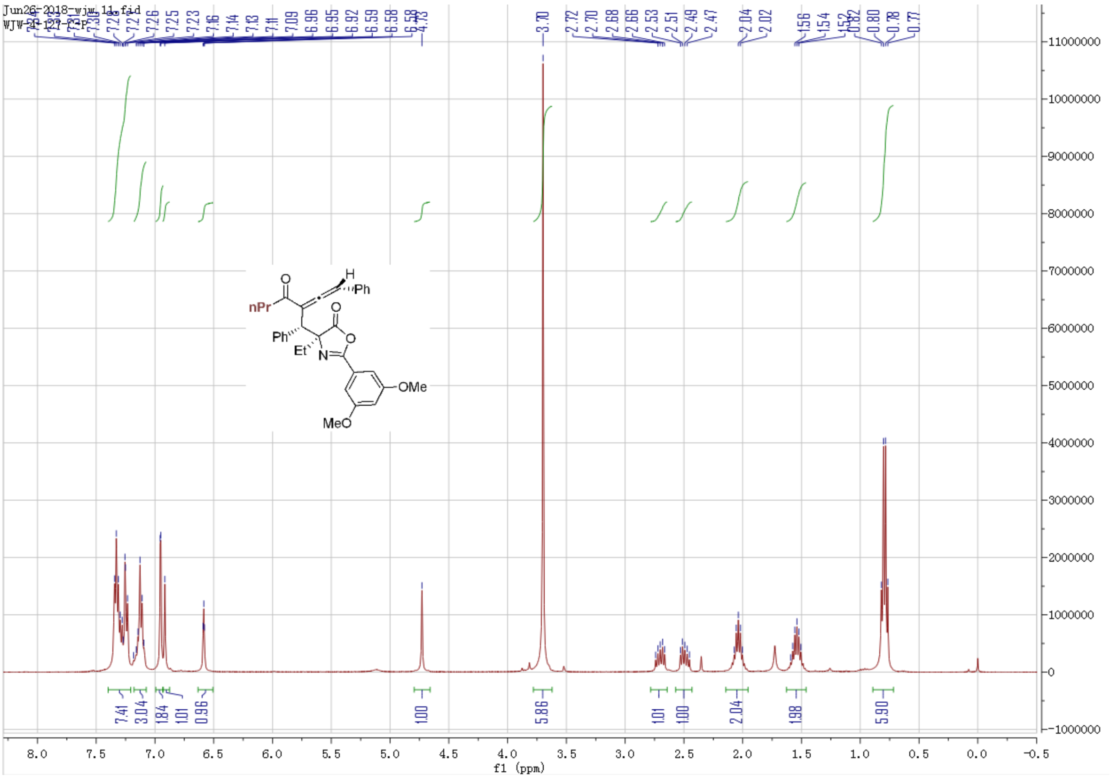

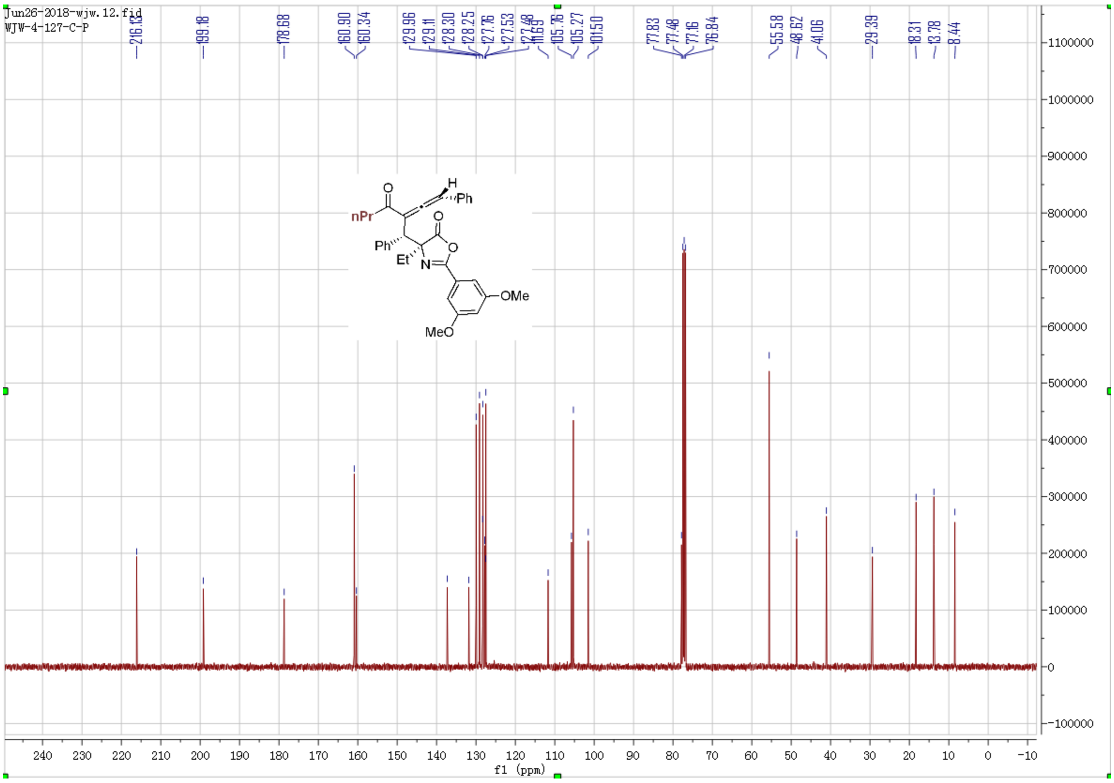

(*S*)-2-(3,5-dimethoxyphenyl)-4-ethyl-4-((*R*)-3-oxo-1-phenyl-2-((*R*)-2-phenylvinylidene)heptyl)oxazol-5(4H)-one (**3t**)

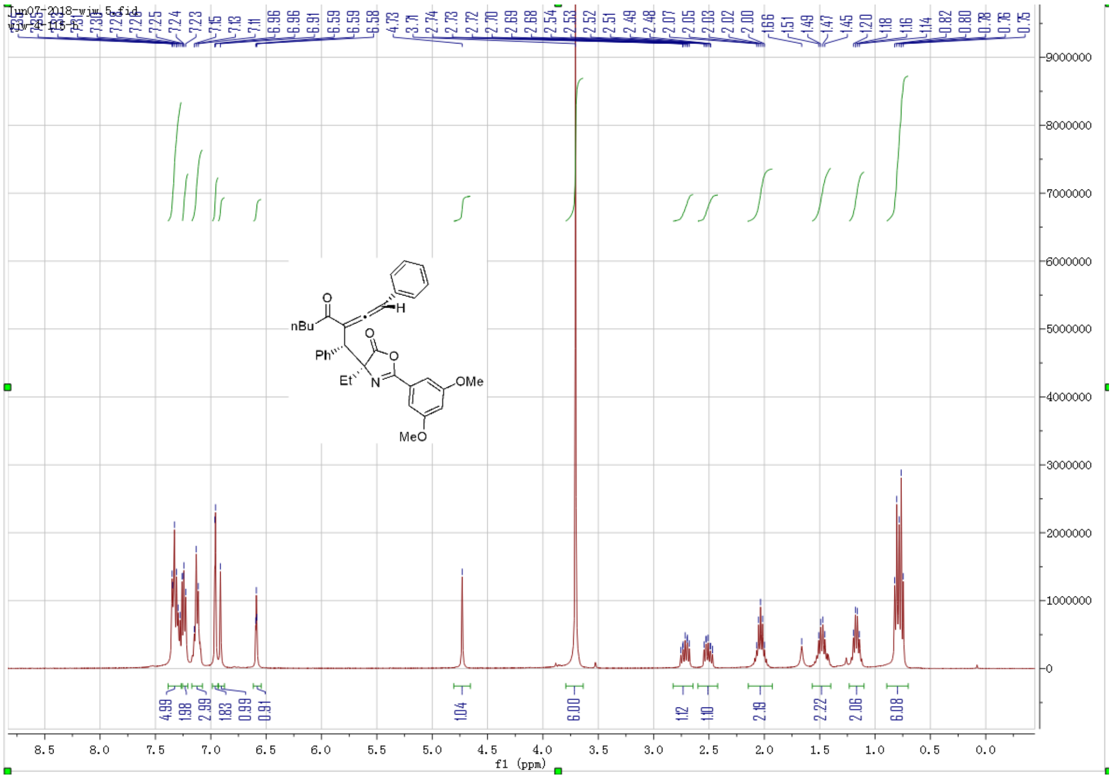

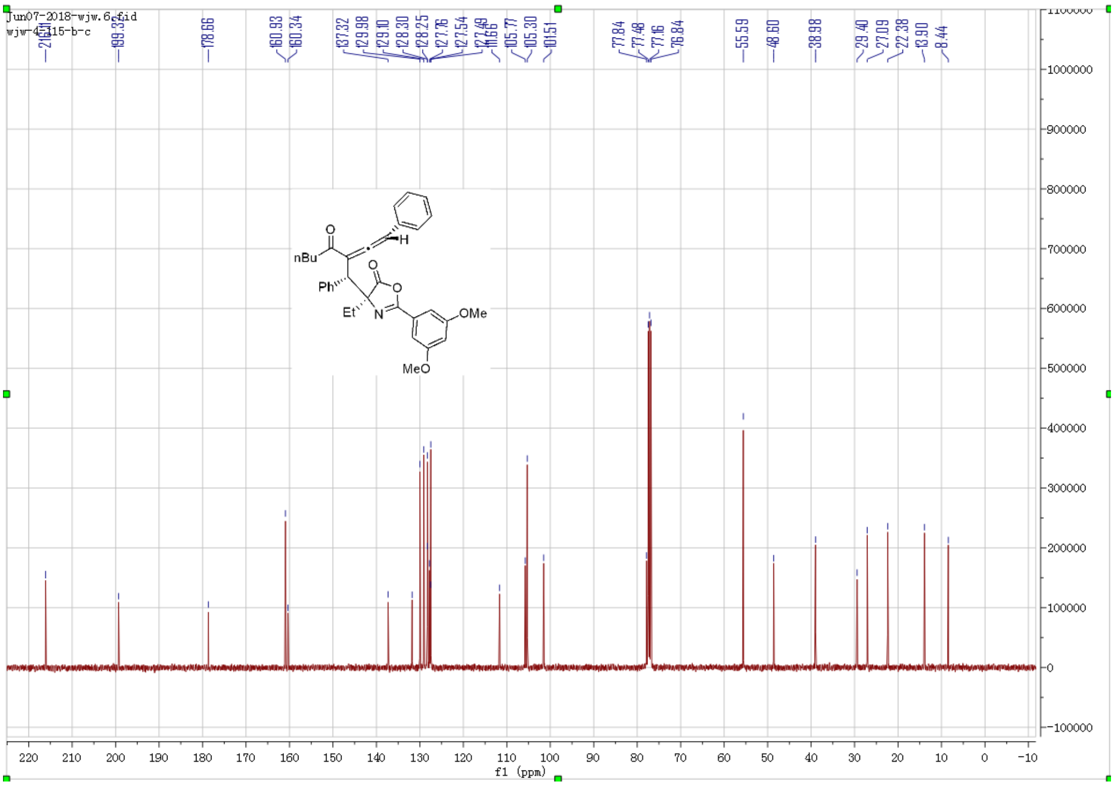

(*S*)-2-(3,5-dimethoxyphenyl)-4-ethyl-4-((*R*)-5-methyl-3-oxo-1-phenyl-2-((*R*)-2-phenylvinylidene)hexyl)oxazol-5(4H)-one (**3u**)

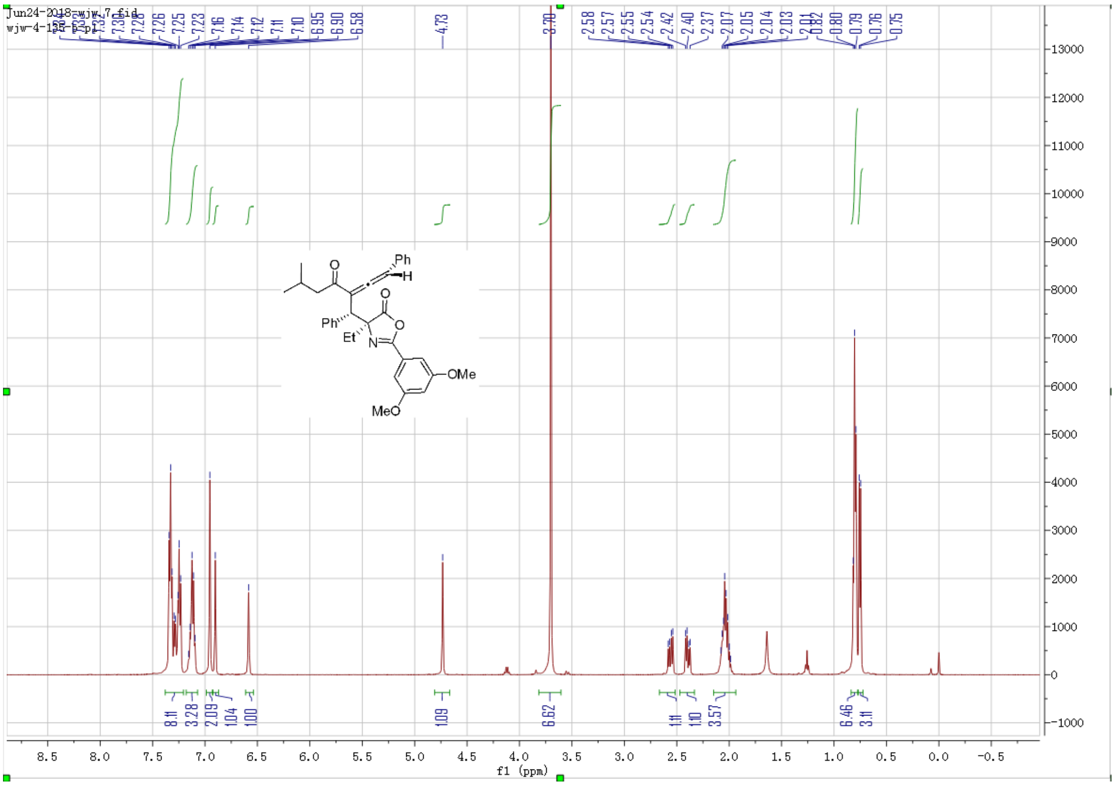

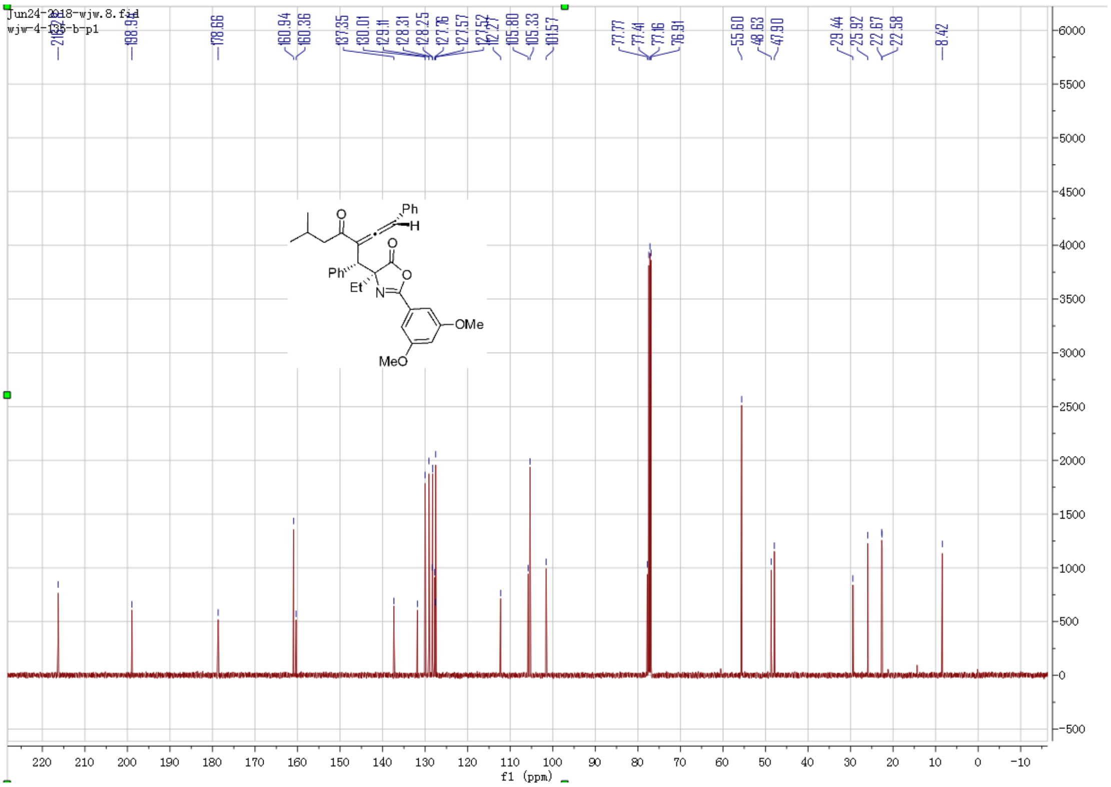

(*S*)-4-((*1R*,*3R*)-2-acetyl-1,4-diphenylbuta-2,3-dien-1-yl)-4-butyl-2-(3,5-dimethoxyphenyl)oxazol-5(4H)-one (**3v**)

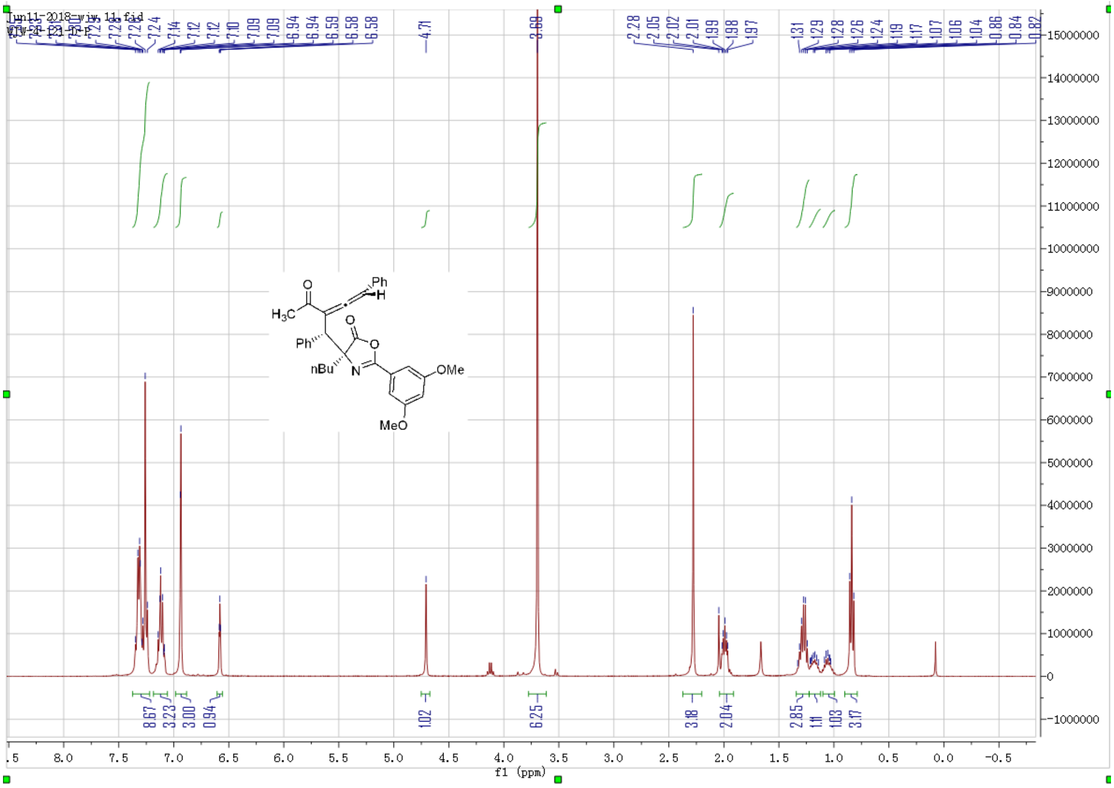

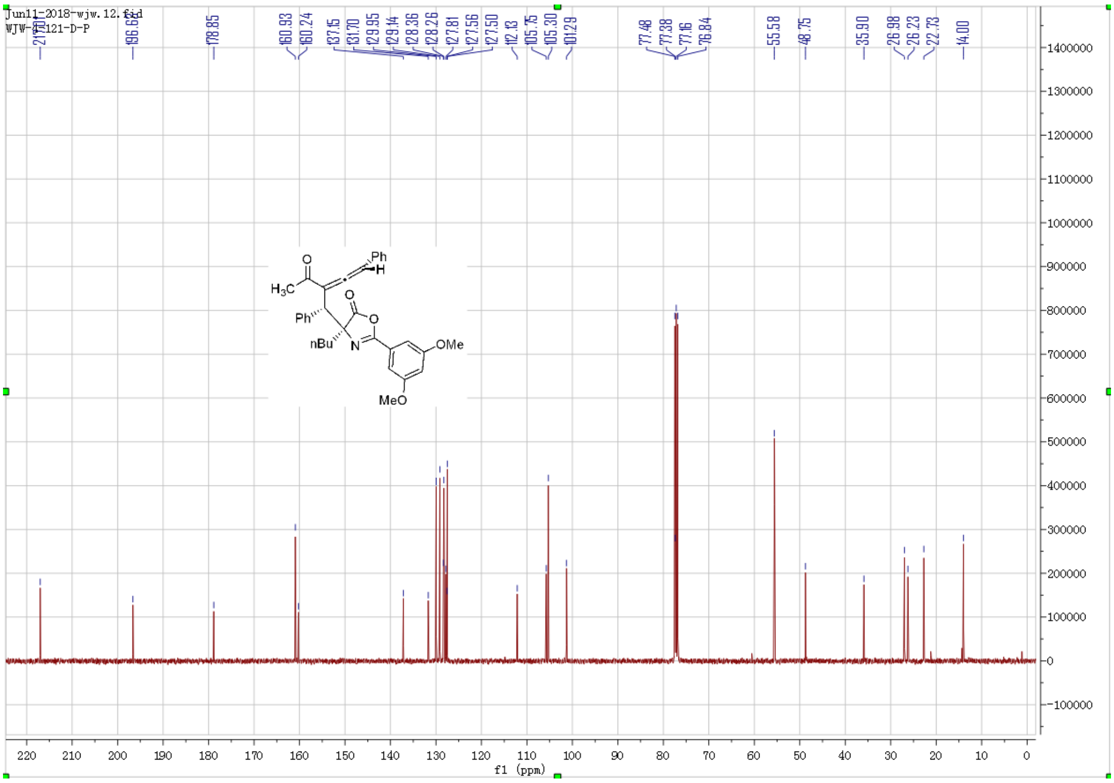

(*S*)-4-((*1R*,*3R*)-2-acetyl-1,4-diphenylbuta-2,3-dien-1-yl)-4-benzyl-2-(3,5-dimethoxyphenyl)oxazol-5(4H)-one (**3w**)

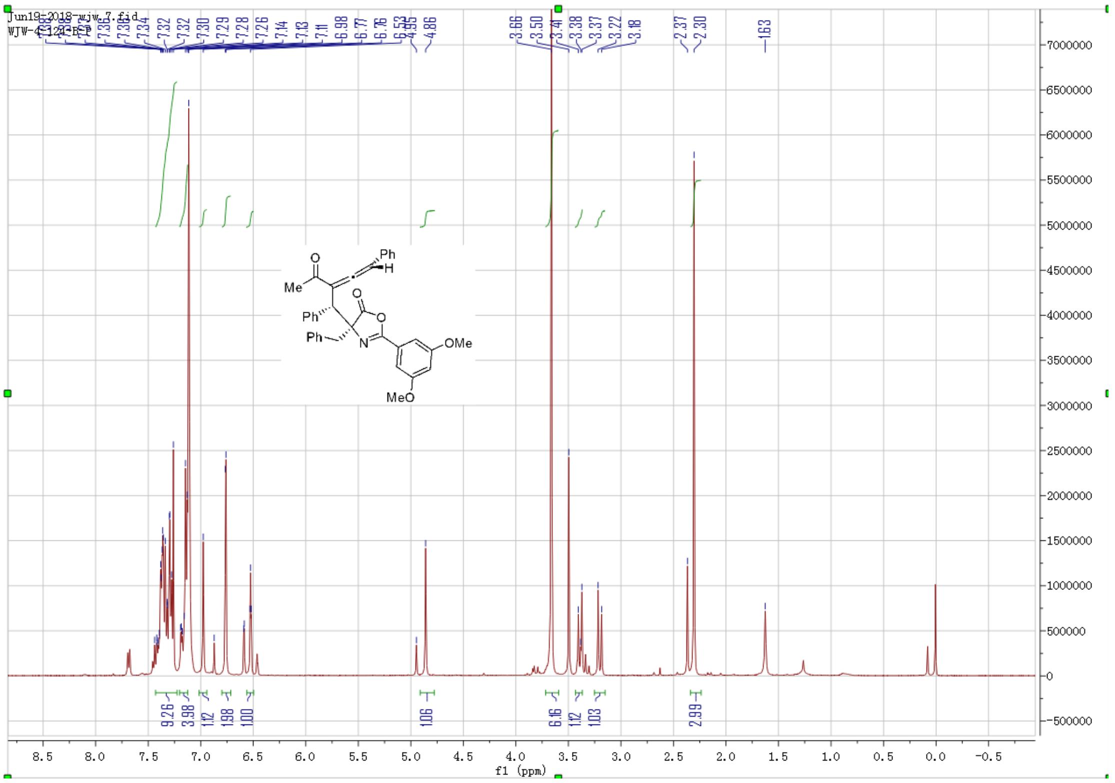

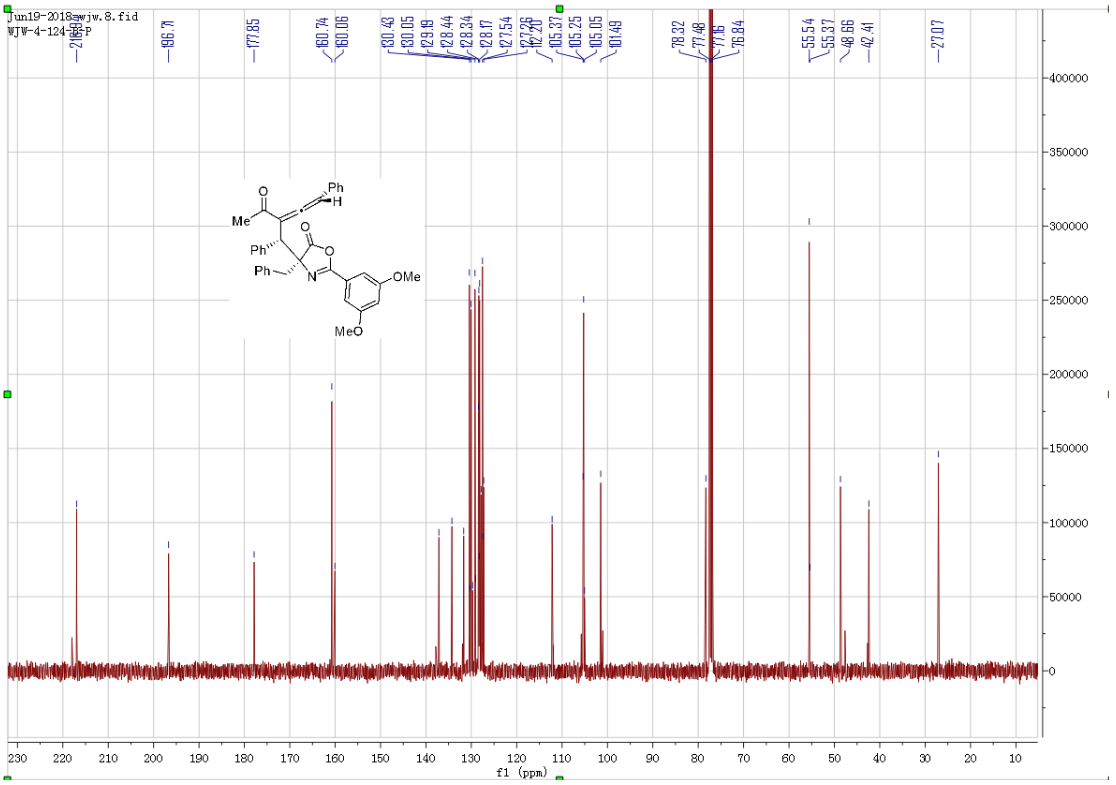

(*S*)-4-((*1R*,*3R*)-2-acetyl-1,4-diphenylbuta-2,3-dien-1-yl)-2-(3,5-dimethoxyphenyl)-4-(2-(methylthio)ethyl)oxazol-5(4H)-one (**3x**)

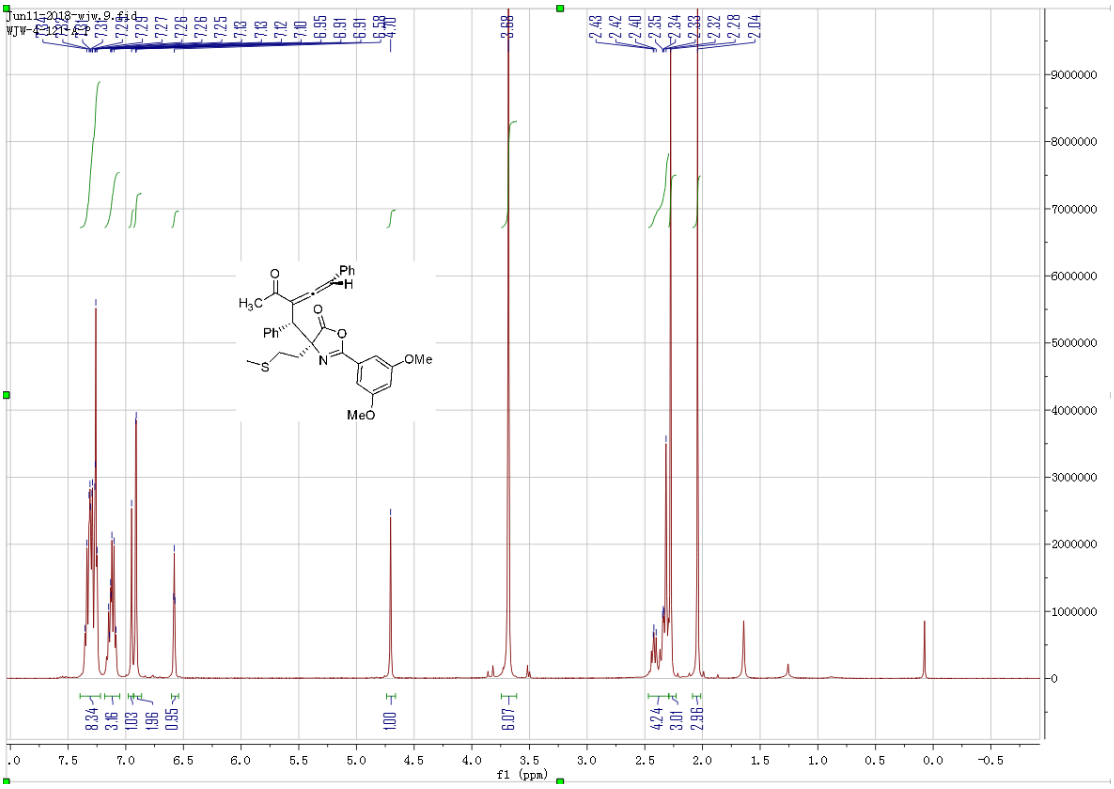

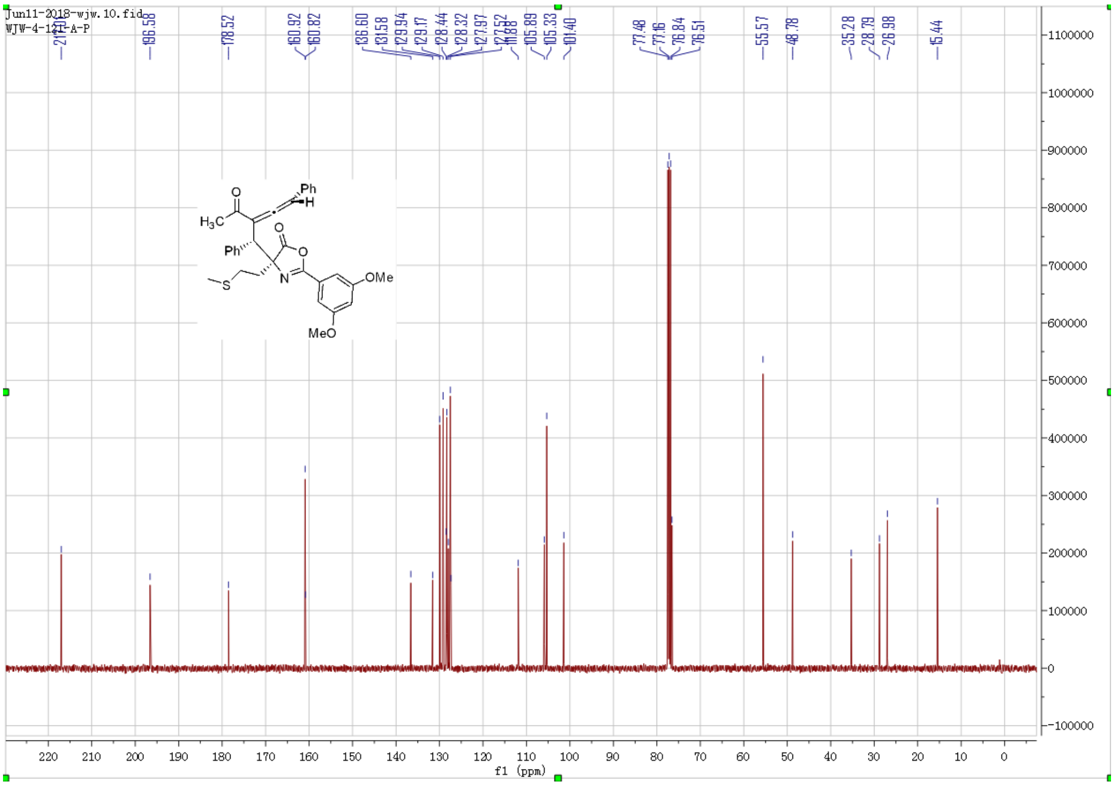

tert-butyl(4-((*S*)-4-((*1R*,*3R*)-2-acetyl-1,4-diphenylbuta-2,3-dien-1-yl)-2-(3,5-dimethoxyphenyl)-5-oxo-4,5-dihydrooxazol-4-yl)butyl)carbamate (**3y**)

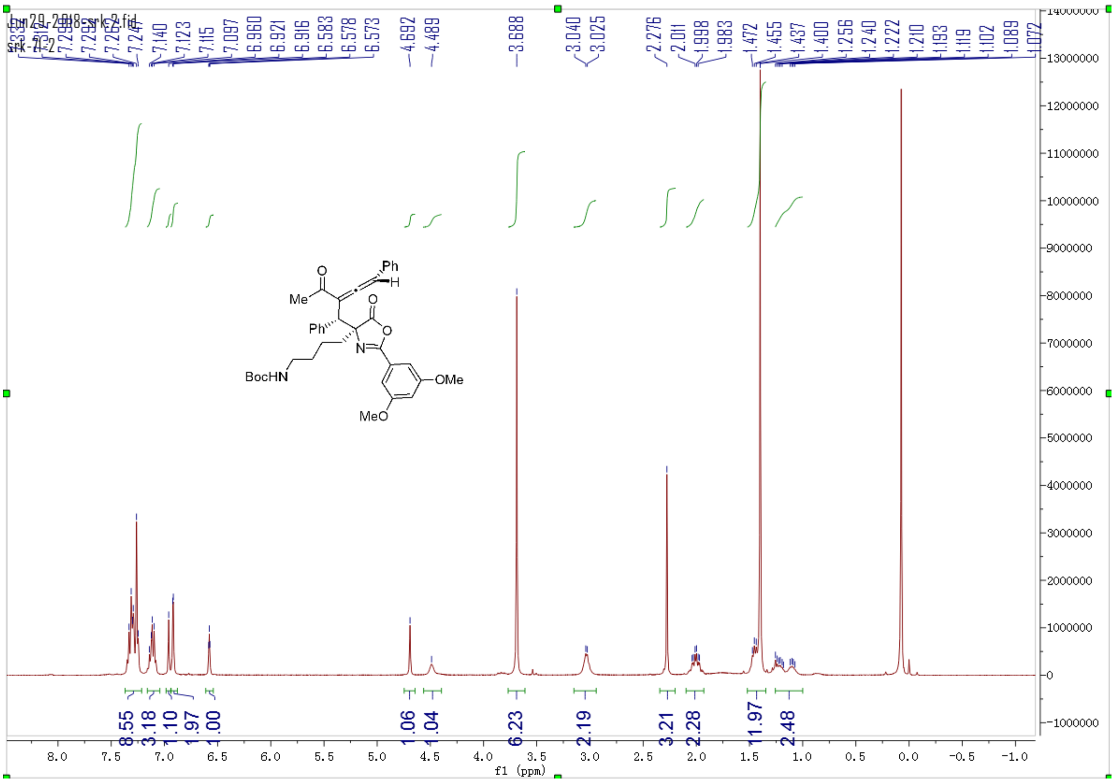

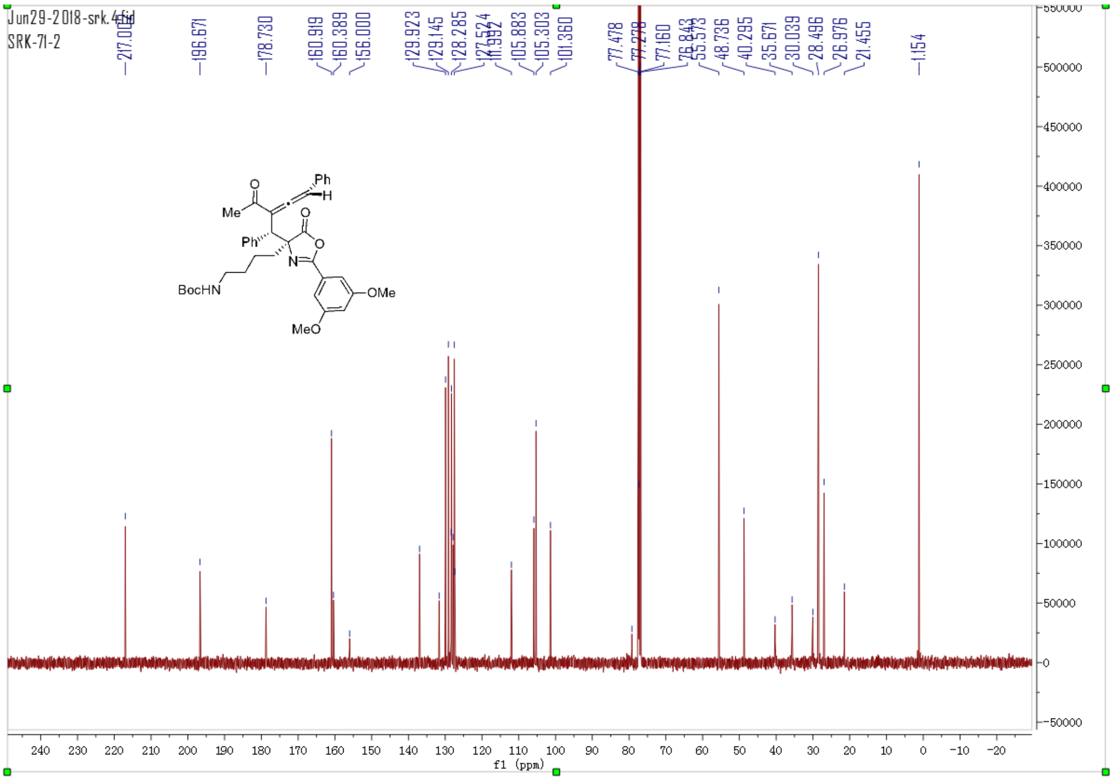

-4-((*1R*,*3S*)-2-acetyl-1,4-diphenylbuta-2,3-dien-1-yl)-2-(3,5-dimethoxyphenyl)-4-ethyloxazol-5(4H)-one (**4a**)

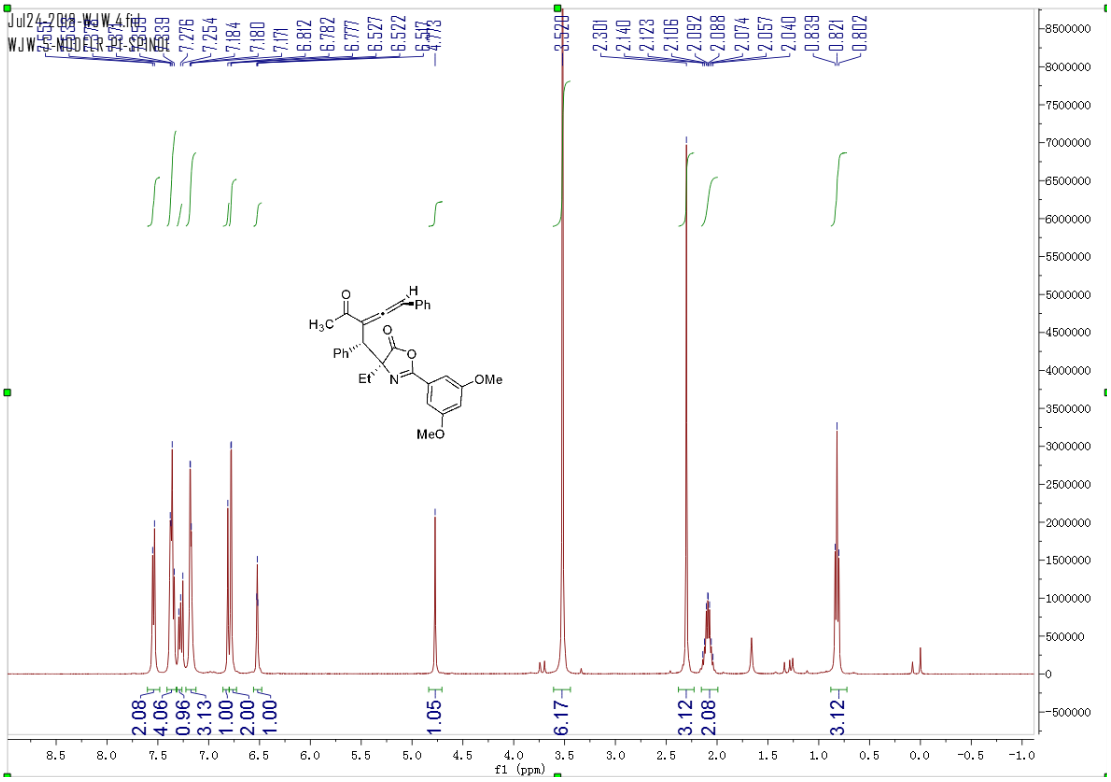

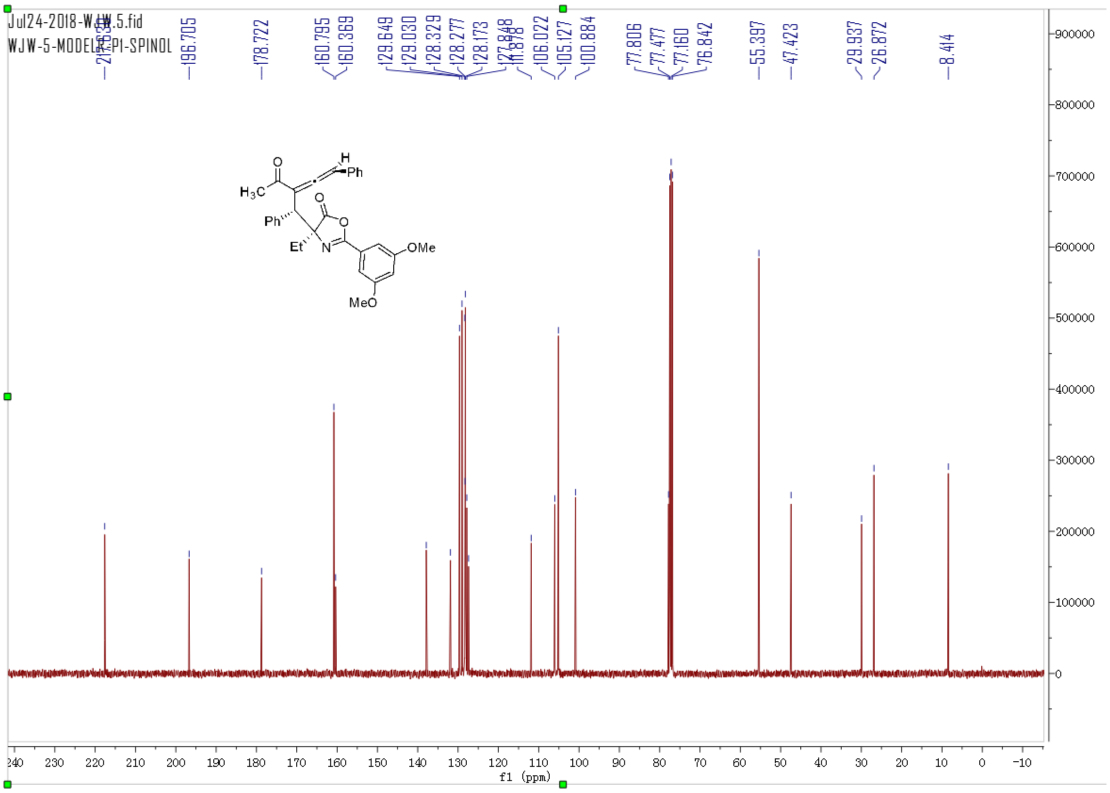

(*S*)-4-((*1R*,*3S*)-2-acetyl-1-phenyl-4-(p-tolyl)buta-2,3-dien-1-yl)-2-(3,5-dimethoxyphenyl)-4-ethyloxazol-5(4H)-one (**4b**)

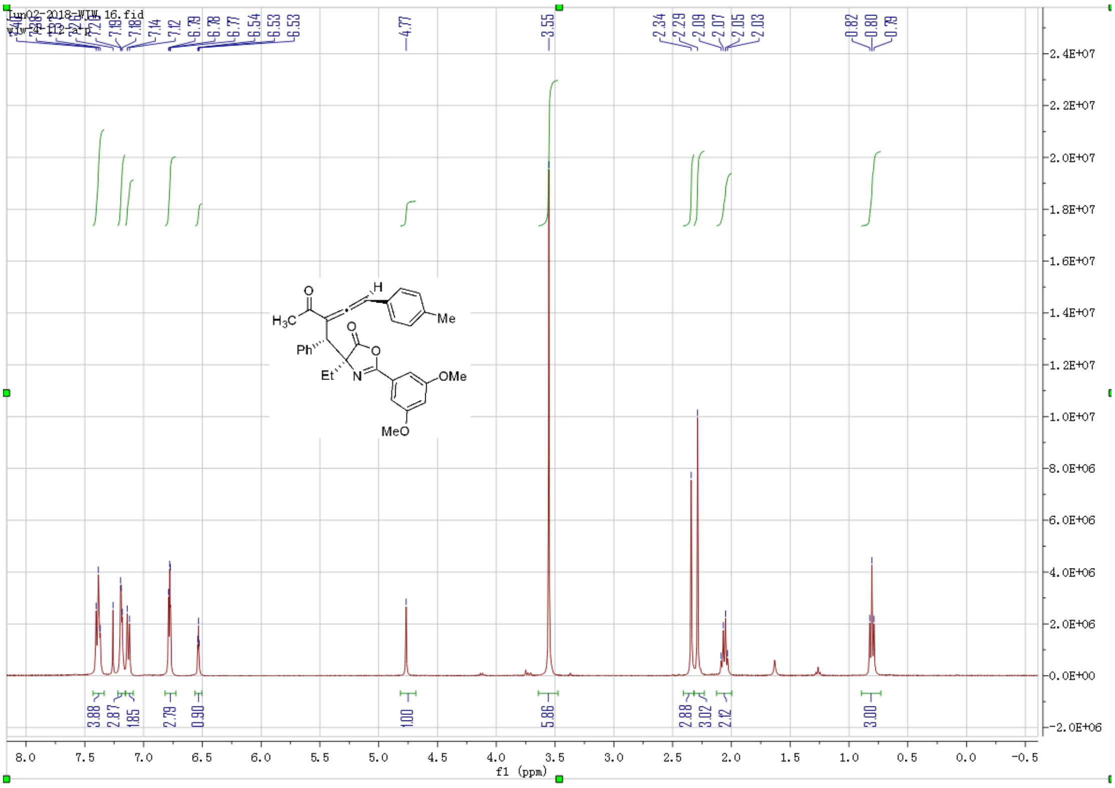

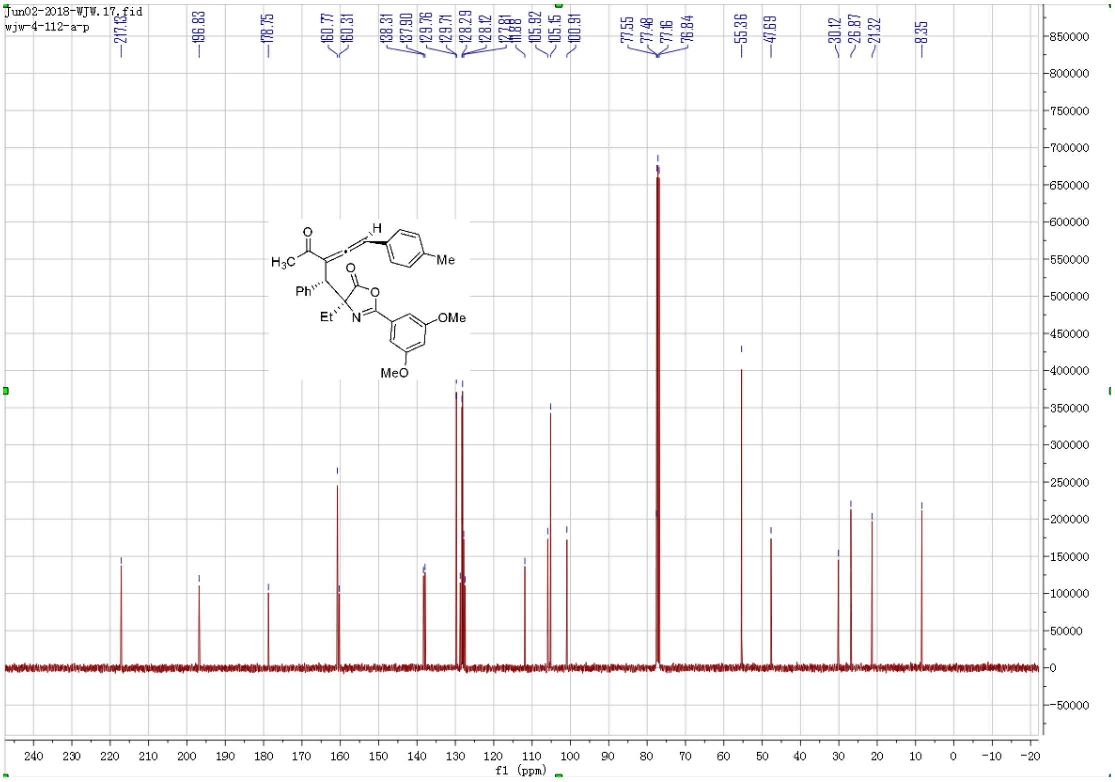

(*S*)-4-((*1R*,*3S*)-2-acetyl-4-(4-chlorophenyl)-1-phenylbuta-2,3-dien-1-yl)-2-(3,5-dimethoxyphenyl)-4-ethyloxazol-5(4H)-one (**4c**)

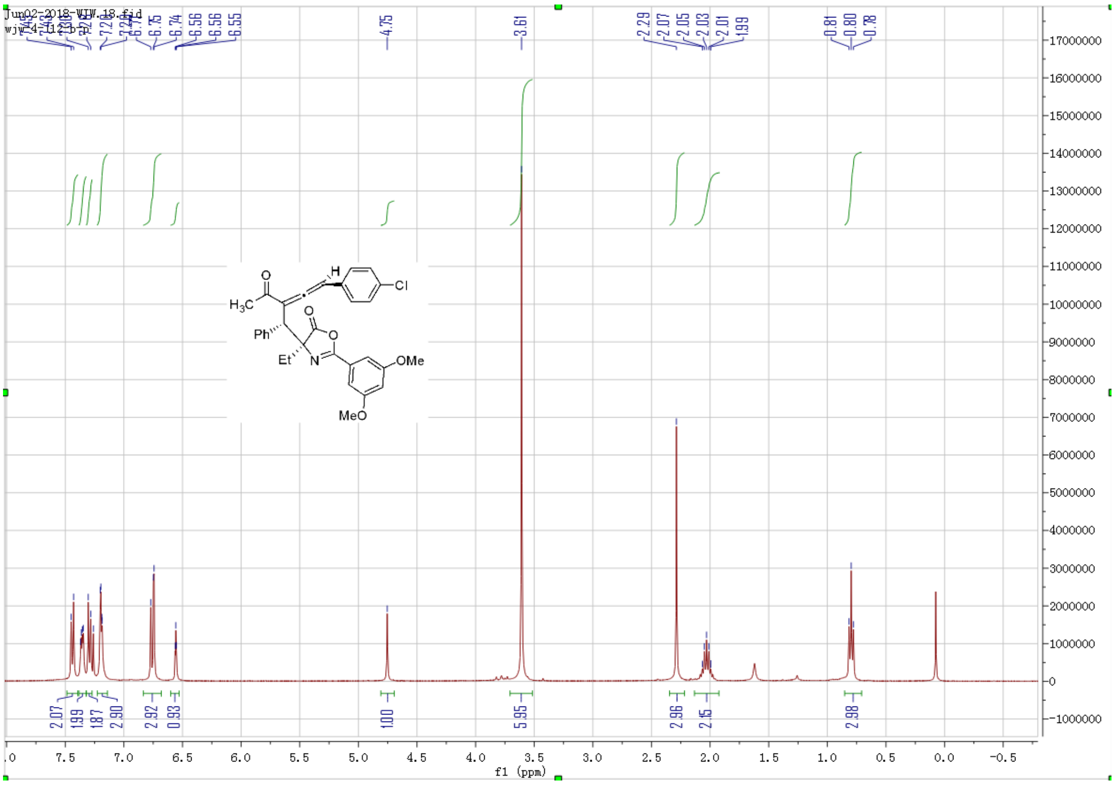

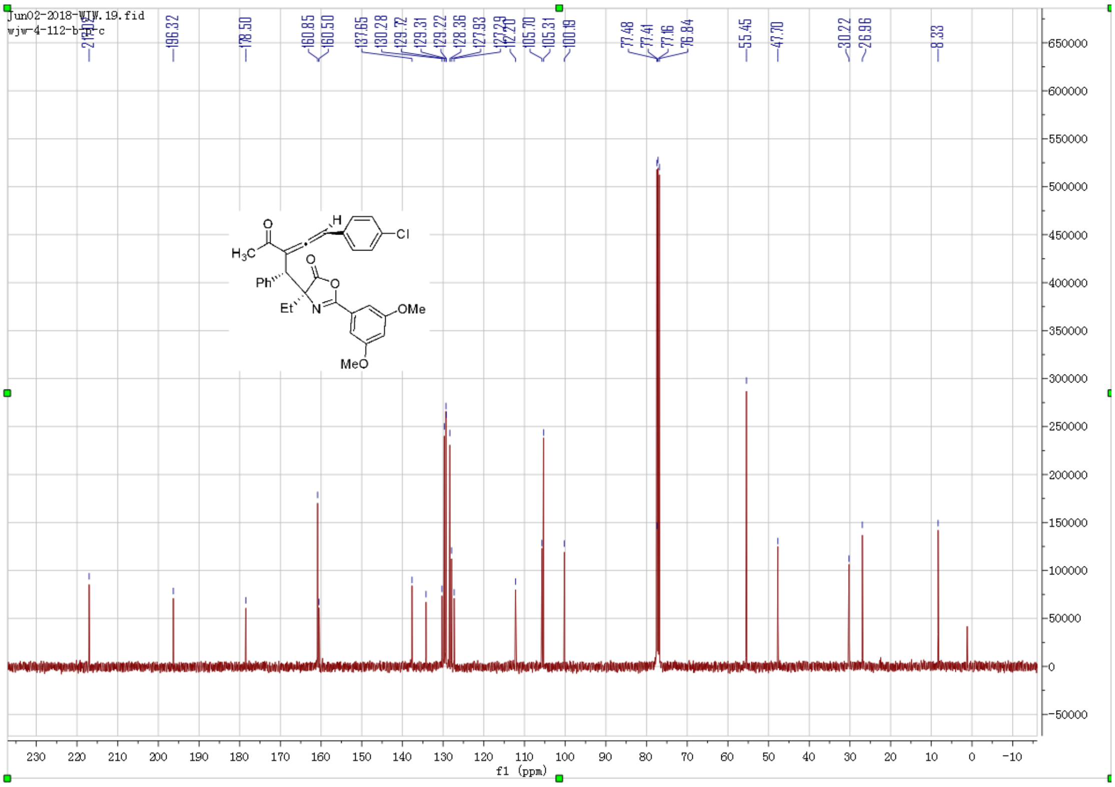

(*S*)-4-((*1R*,*3S*)-2-acetyl-4-(4-methoxyphenyl)-1-phenylbuta-2,3-dien-1-yl)-2-(3,5-dimethoxyphenyl)-4-ethyloxazol-5(4H)-one (**4d**)

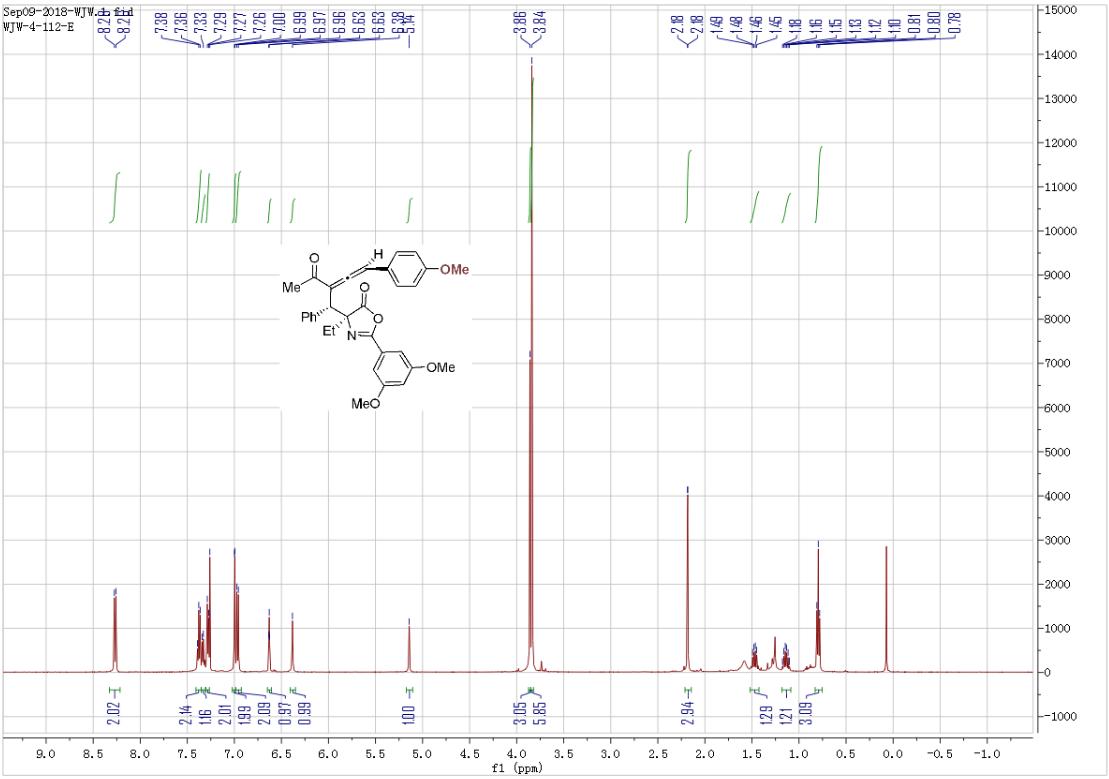

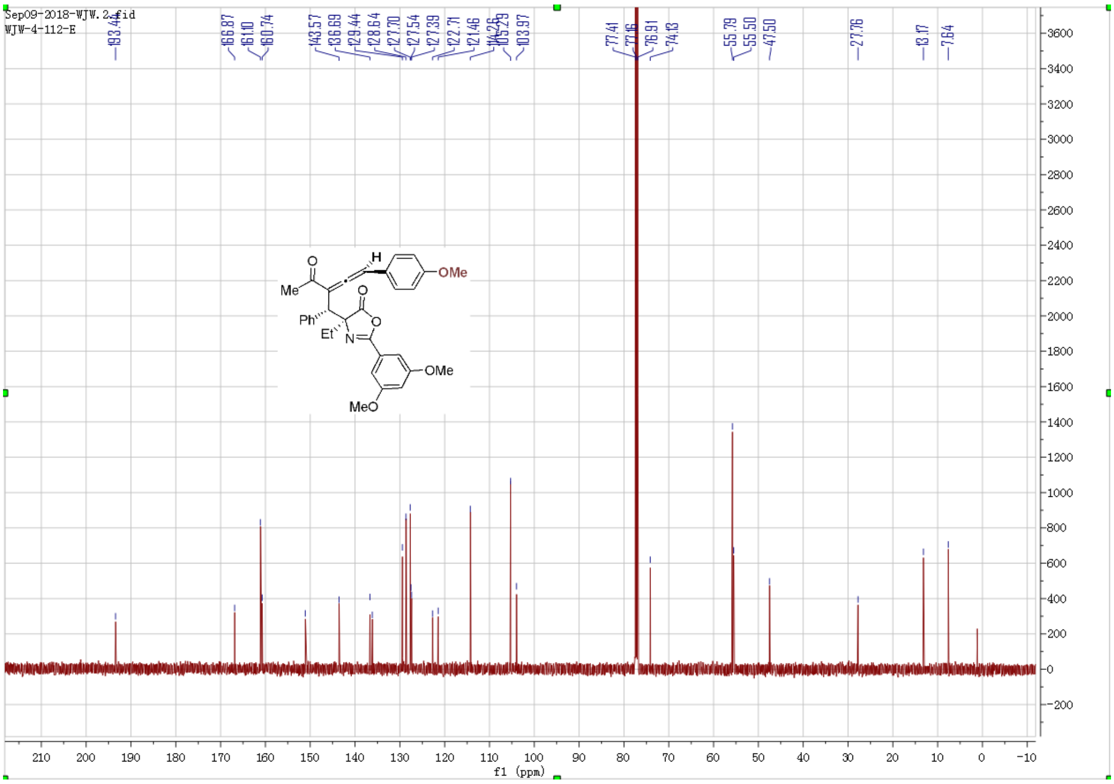

-4-((*1R*,*3S*)-2-acetyl-4-(4-fluorophenyl)-1-phenylbuta-2,3-dien-1-yl)-2-(3,5-dimethoxyphenyl)-4-ethyloxazol-5(4H)-one (**4e**)

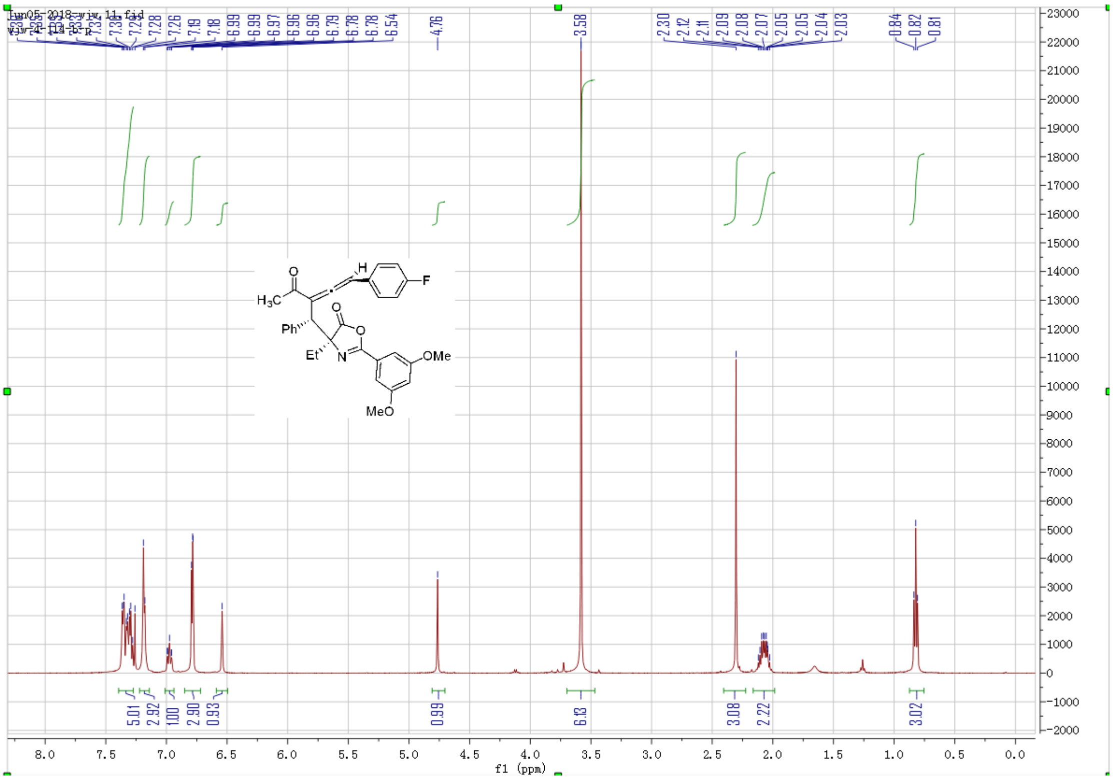

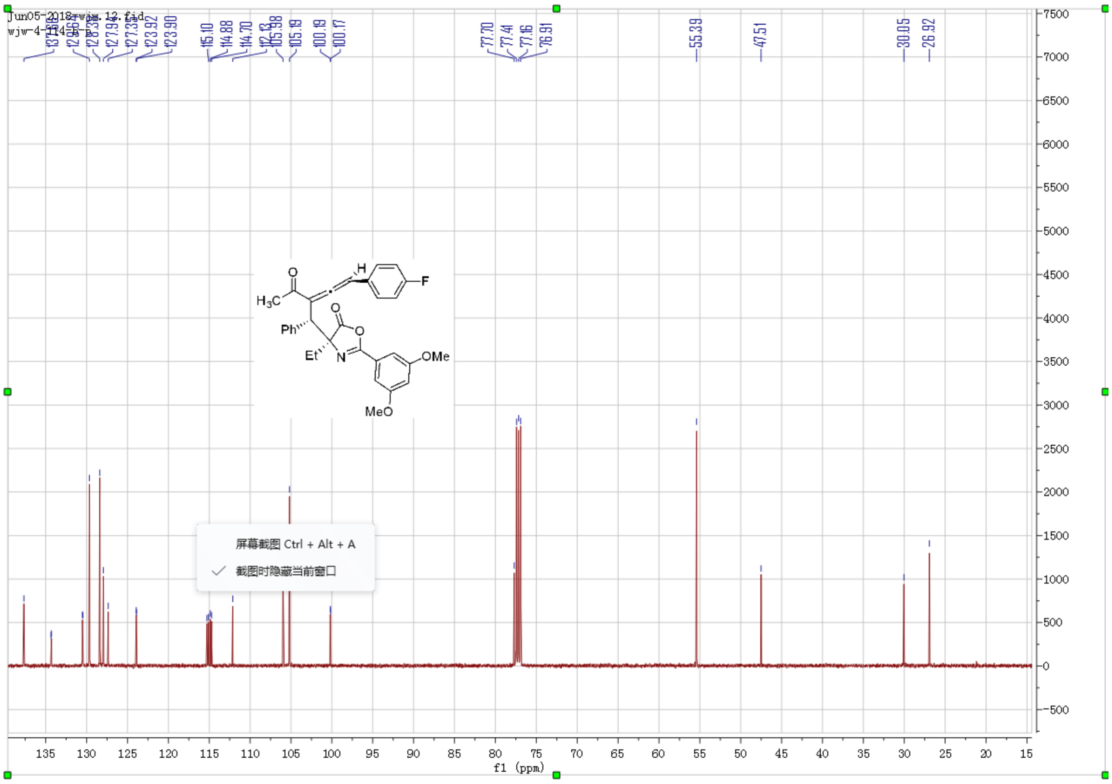

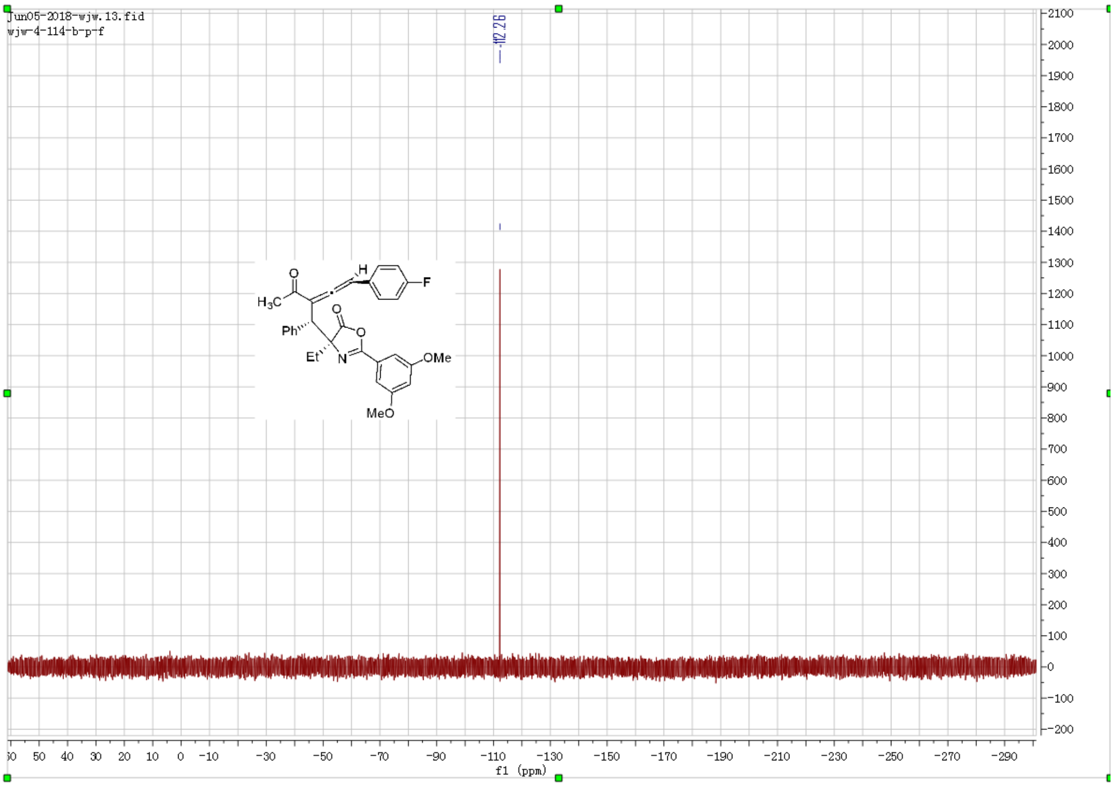

Jun05-2018-wjw.13.fid
wjw-4-114-b-p-f
-112.26
H
F
O
H_3C
O
O
Ph
Et
N
OMe
MeO
f1 (ppm)

(*S*)-4-((*1R*,*3S*)-2-acetyl-1-phenyl-4-(4-(trifluoromethyl)phenyl)buta-2,3-dien-1-yl)-2-(3,5-dimethoxyphenyl)-4-ethyloxazol-5(4H)-one (**4f**)

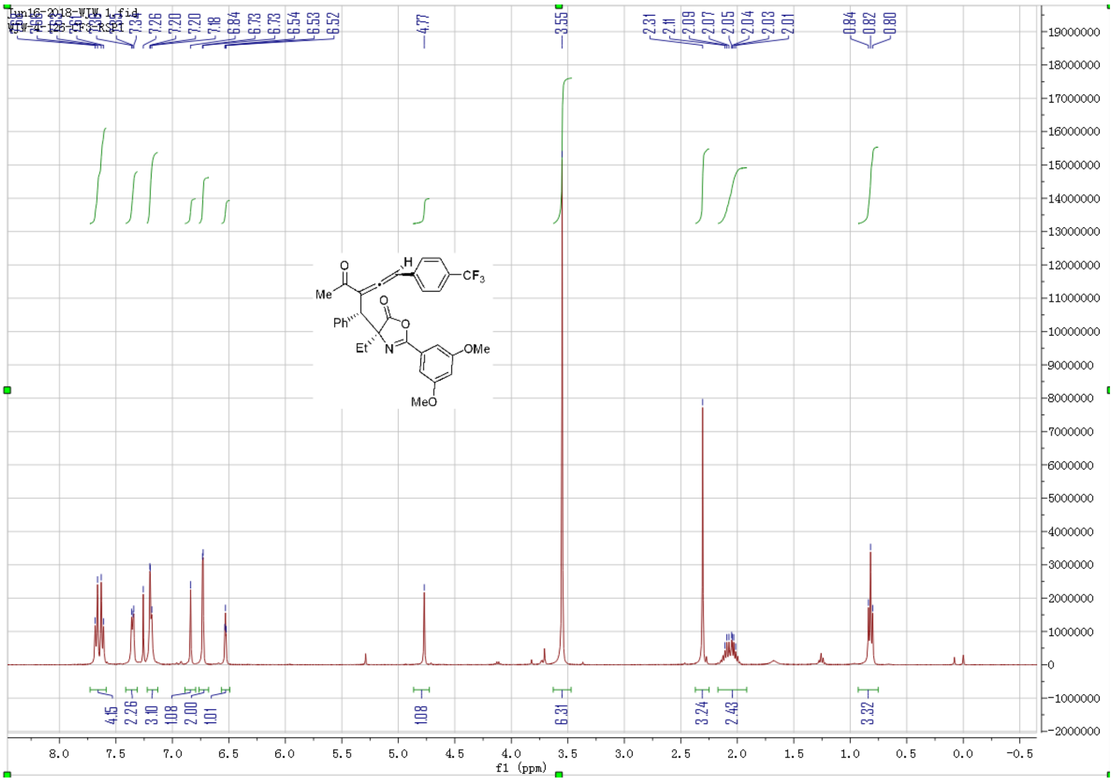

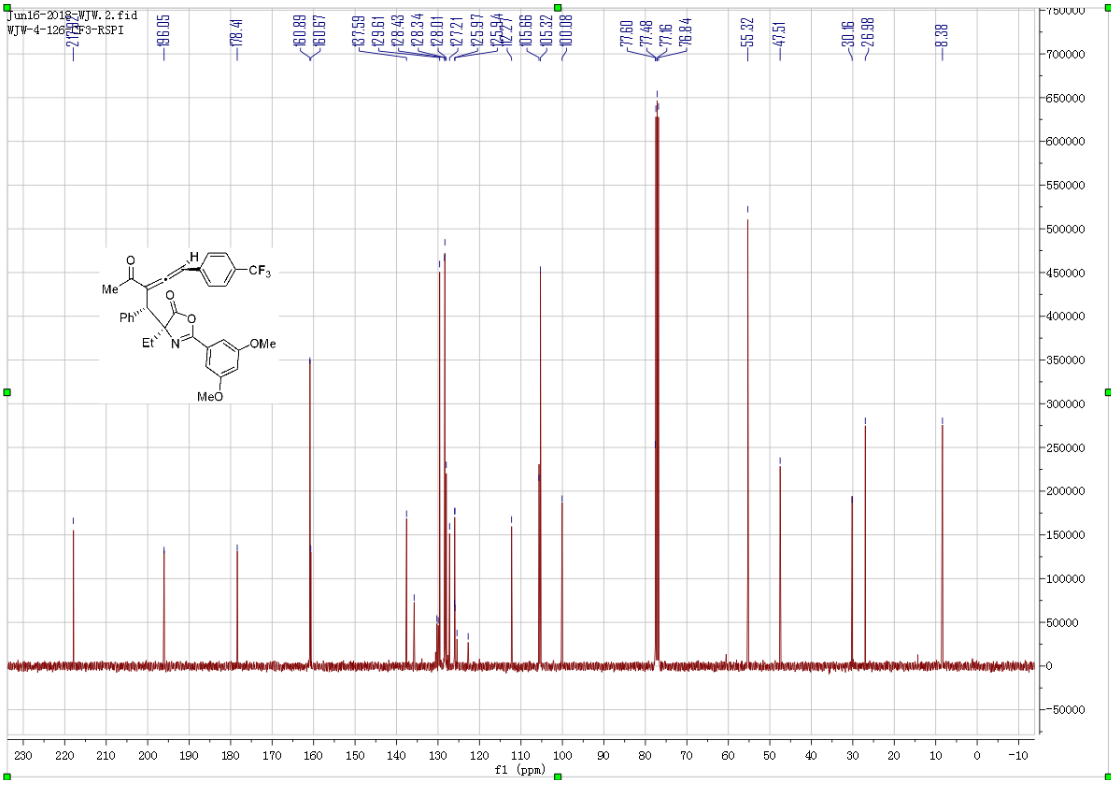

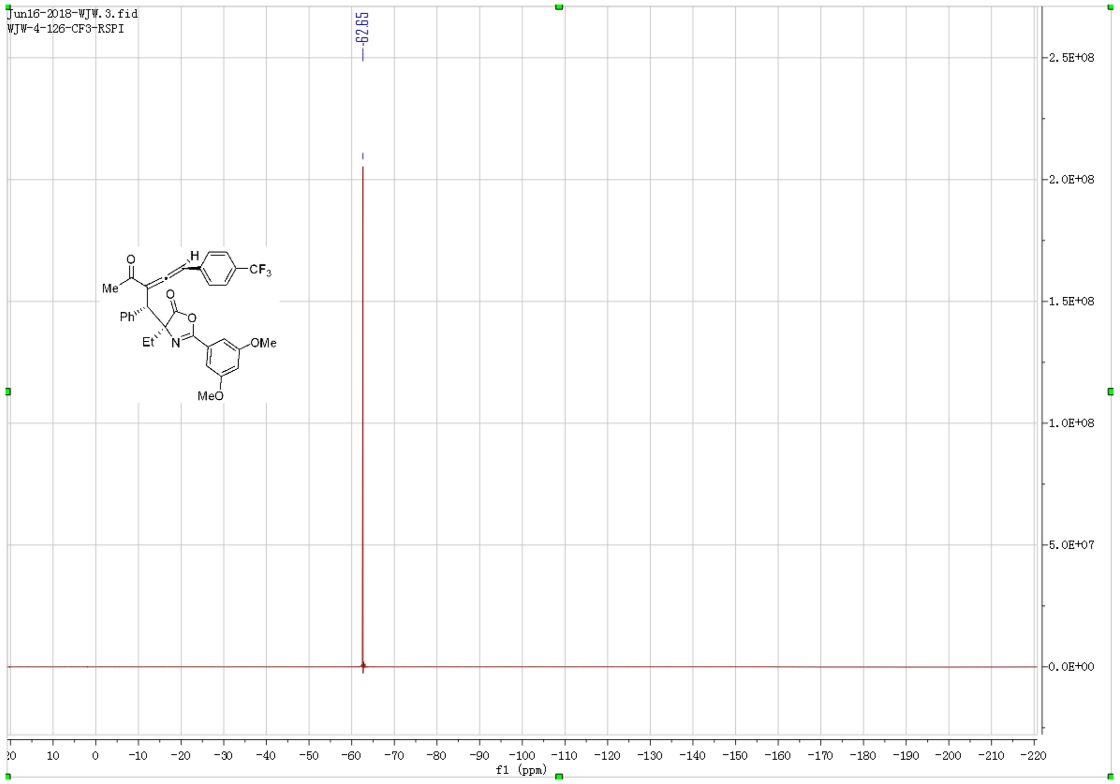
Jun16-2018-WJW.3.fid
WJW-4-126-CF3-RSPI
—62.65
2.5E+08
2.0E+08
1.5E+08
1.0E+08
5.0E+07
0.0E+00
f1 (ppm)
O
Me
H
CF3
Ph
O
O
Et
N
OMe
MeO

Methyl 3-((*S*)-3-((*R*)-((*S*)-2-(3,5-dimethoxyphenyl)-4-ethyl-5-oxo-4,5-dihydrooxazol-4-yl)(phenyl)methyl)-4-oxopenta-1,2-dien-1-yl)benzoate (**4g**)

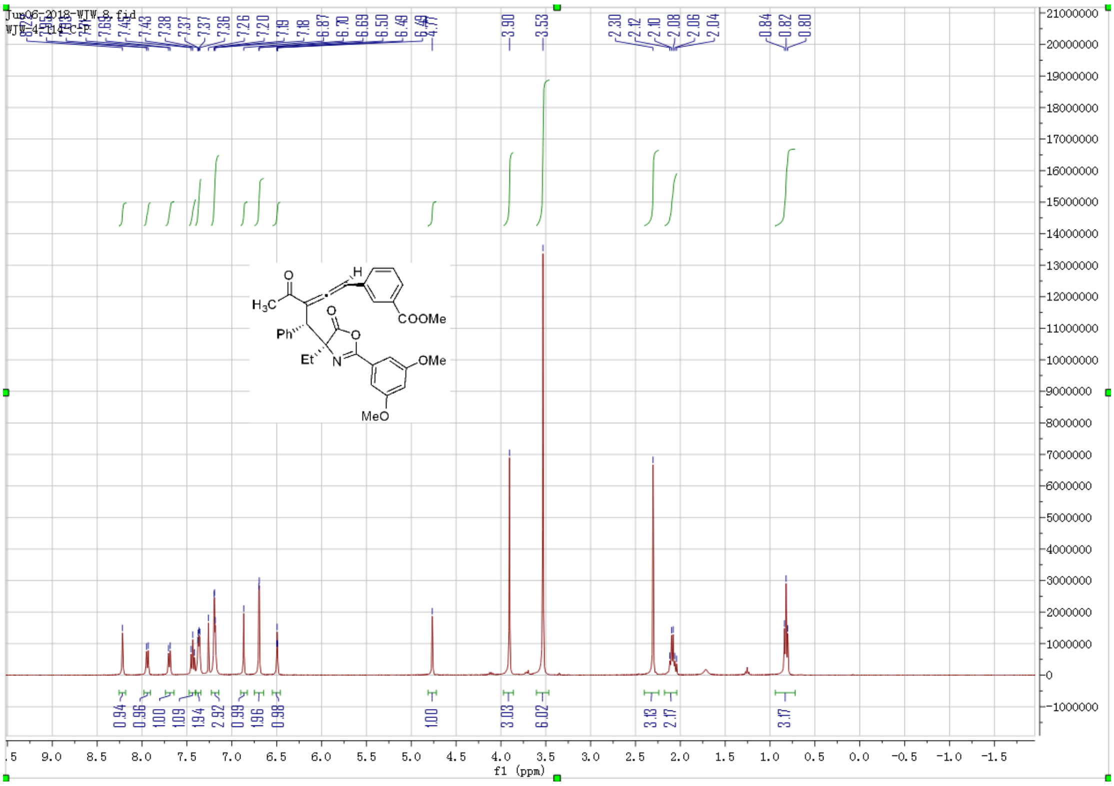

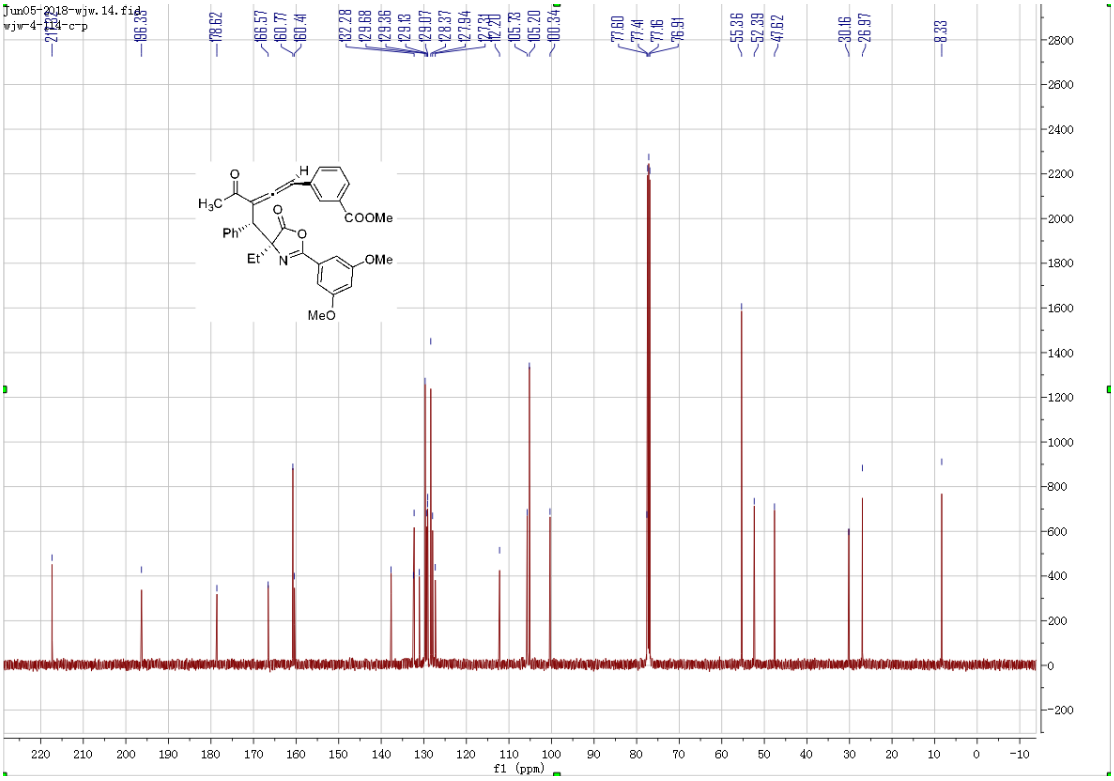

(*S*)-4-((*1R*,*3S*)-2-acetyl-4-(2-chlorophenyl)-1-phenylbuta-2,3-dien-1-yl)-2-(3,5-dimethoxyphenyl)-4-ethyloxazol-5(4H)-one (**4h**)

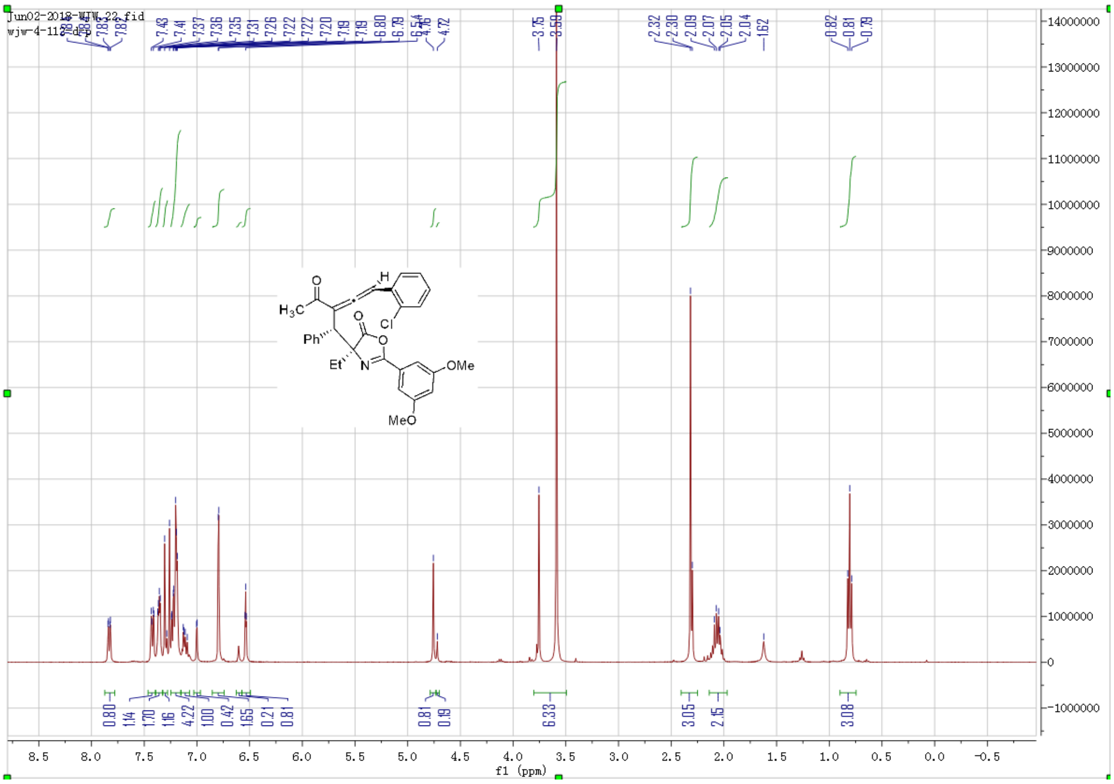

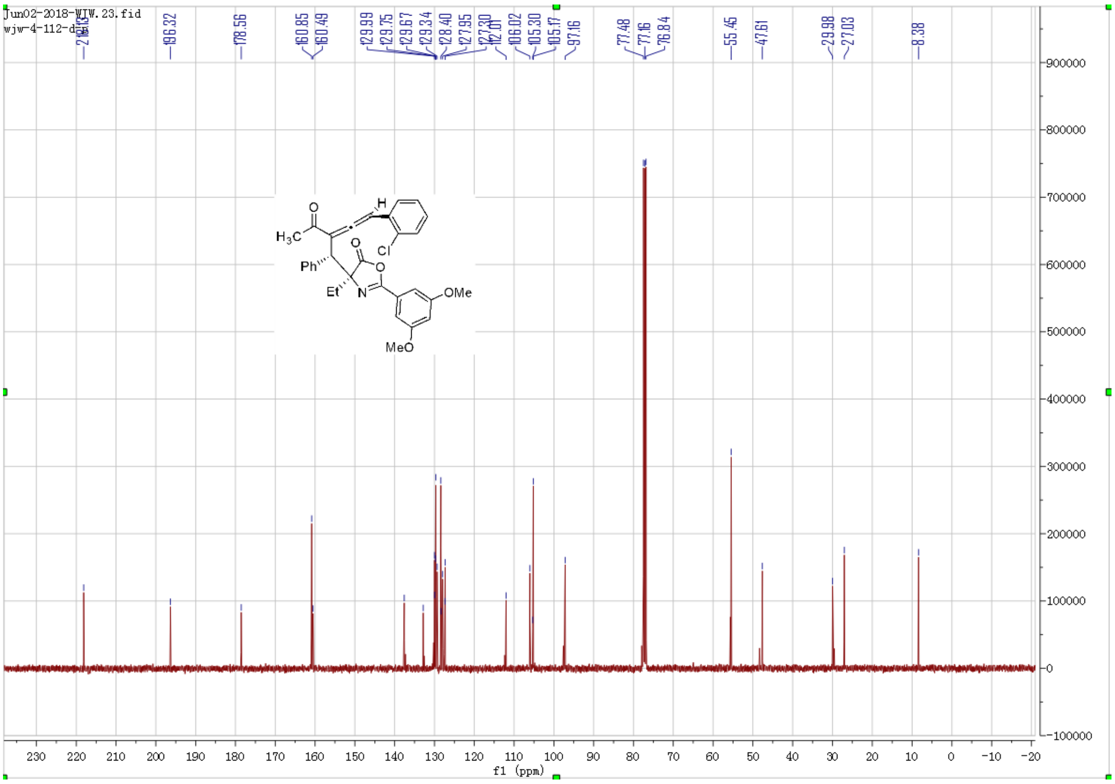

(*S*)-4-((*1R*,*3R*)-2-acetyl-1-phenyl-4-(thiophen-3-yl)buta-2,3-dien-1-yl)-2-(3,5-dimethoxyphenyl)-4-ethyloxazol-5(4H)-one (**4i**)

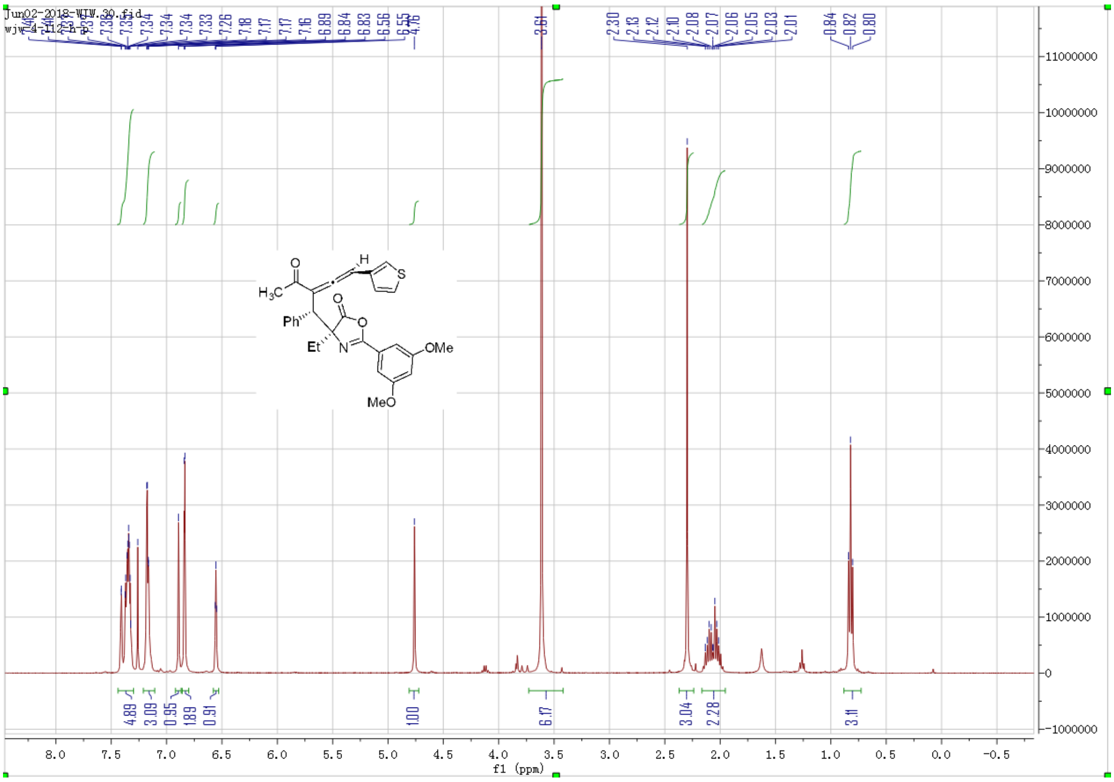

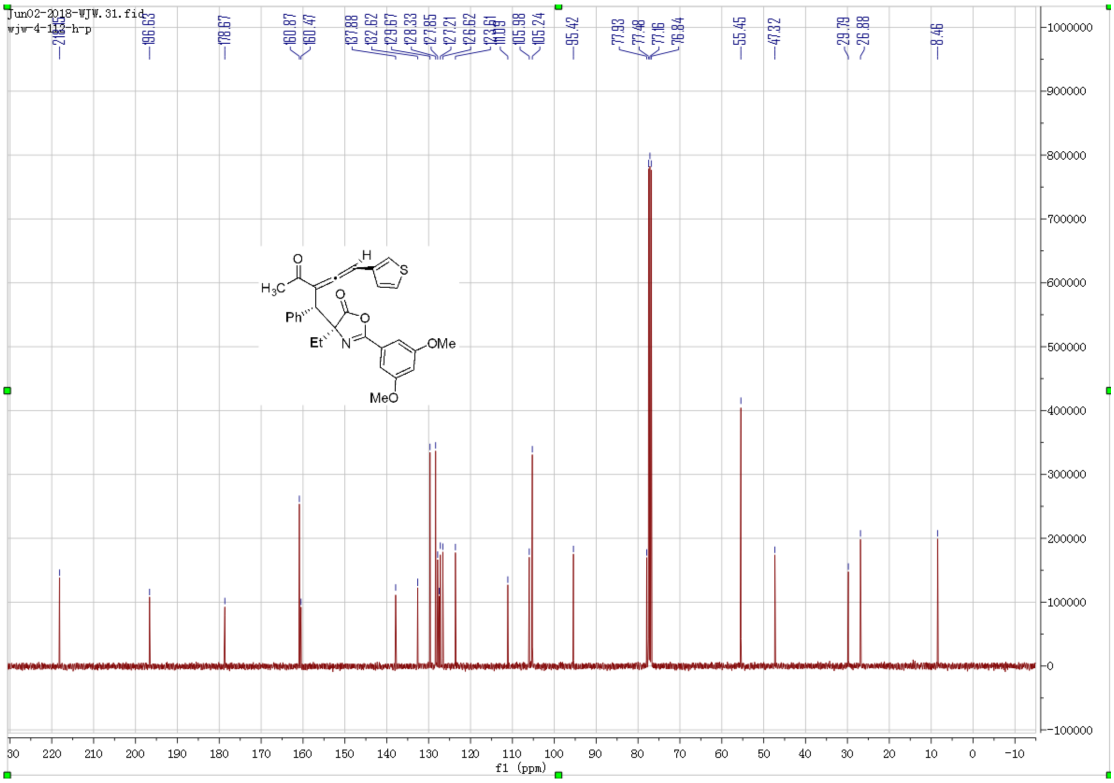

(*S*)-4-((*1R*,*3S*)-2-acetyl-4-(cyclohex-1-en-1-yl)-1-phenylbuta-2,3-dien-1-yl)-2-(3,5-dimethoxyphenyl)-4-ethyloxazol-5(4H)-one (**4j**)

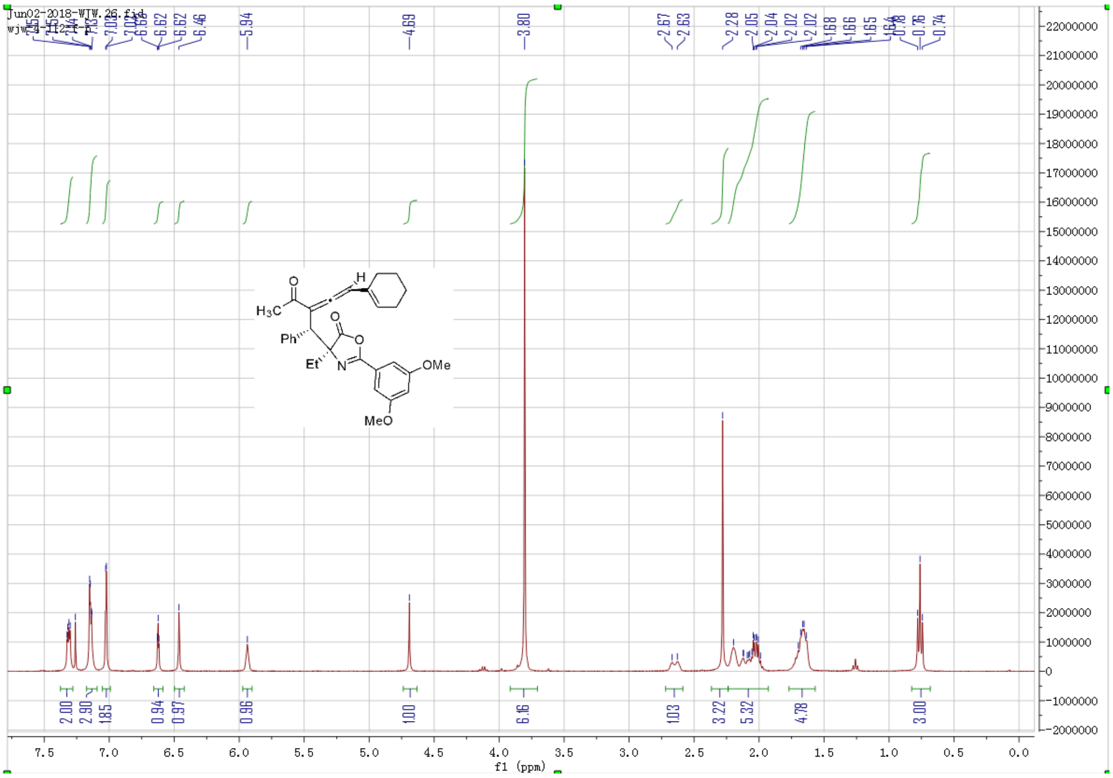

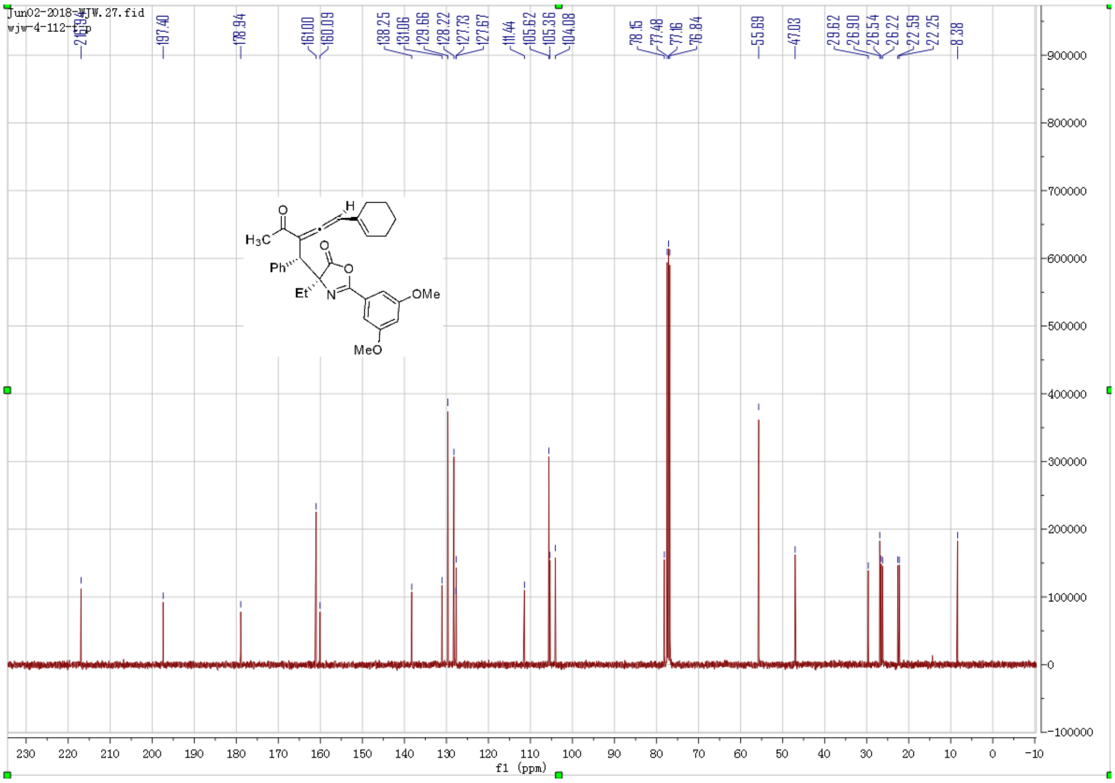

(*S*)-4-((*1R*,*3S*)-2-acetyl-1-phenylocta-2,3-dien-1-yl)-2-(3,5-dimethoxyphenyl)-4-ethyloxazol-5(4H)-one (**4k**)

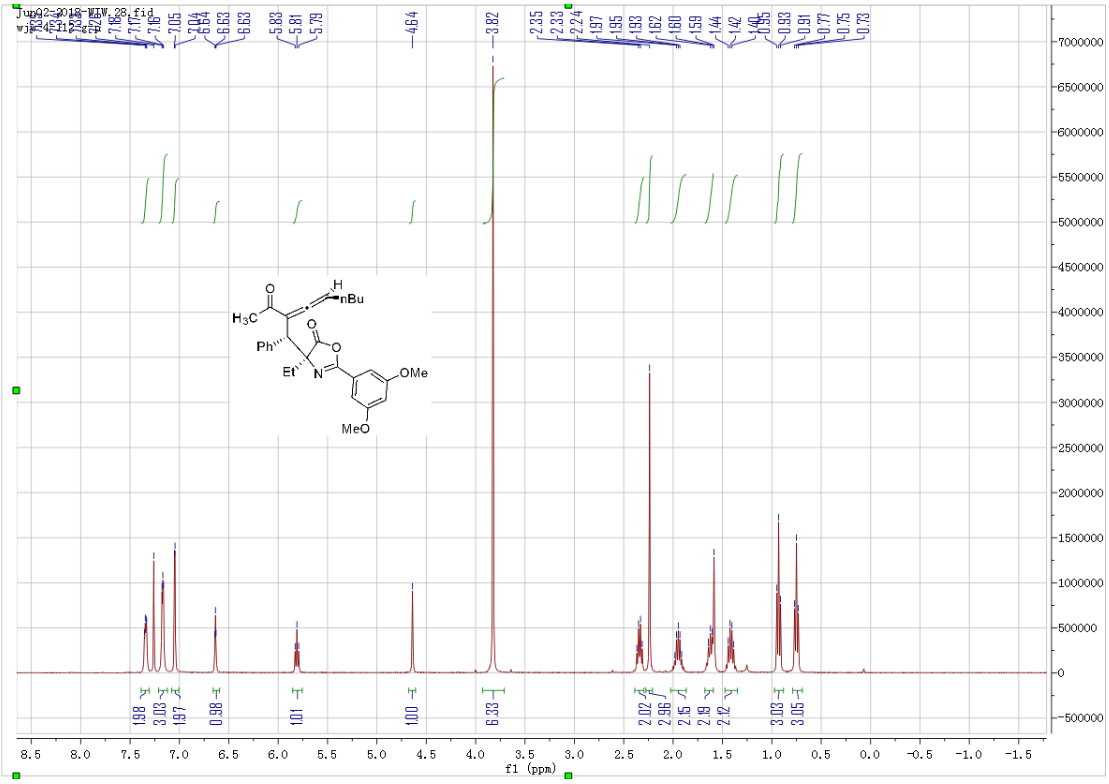

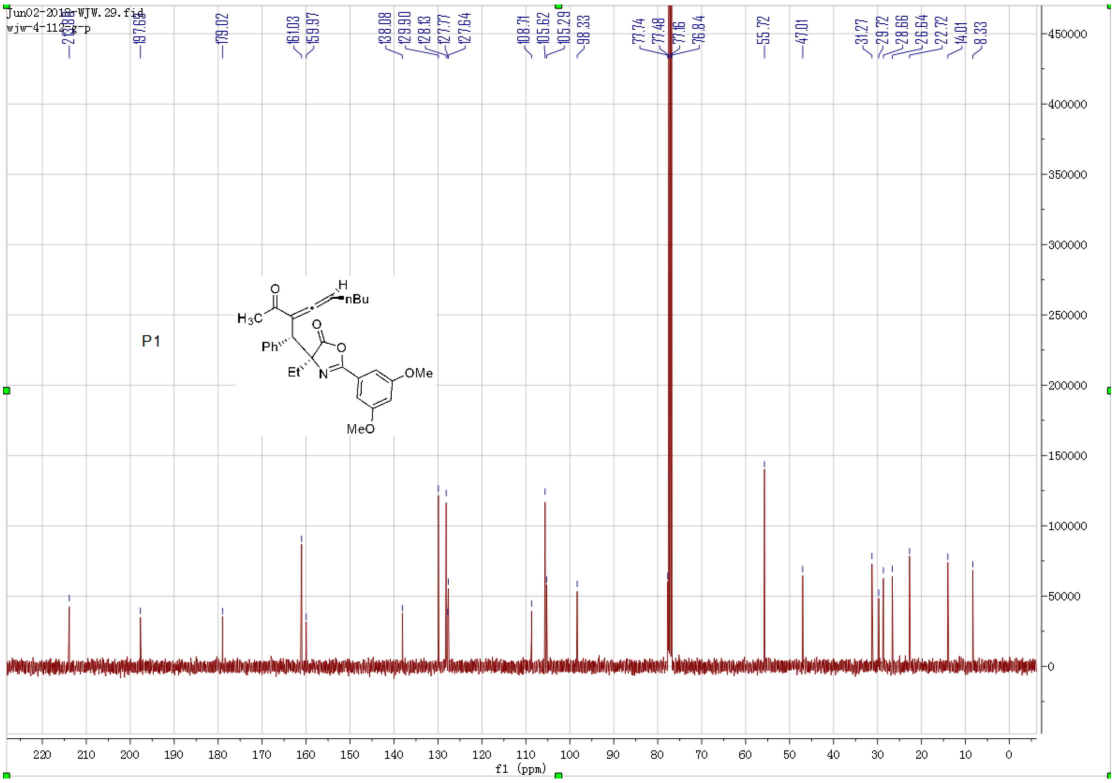

(*S*)-4-((*1R*,*3S*)-2-acetyl-4-phenyl-1-(p-tolyl)buta-2,3-dien-1-yl)-2-(3,5-dimethoxyphenyl)-4-ethyloxazol-5(4H)-one (**4l**)

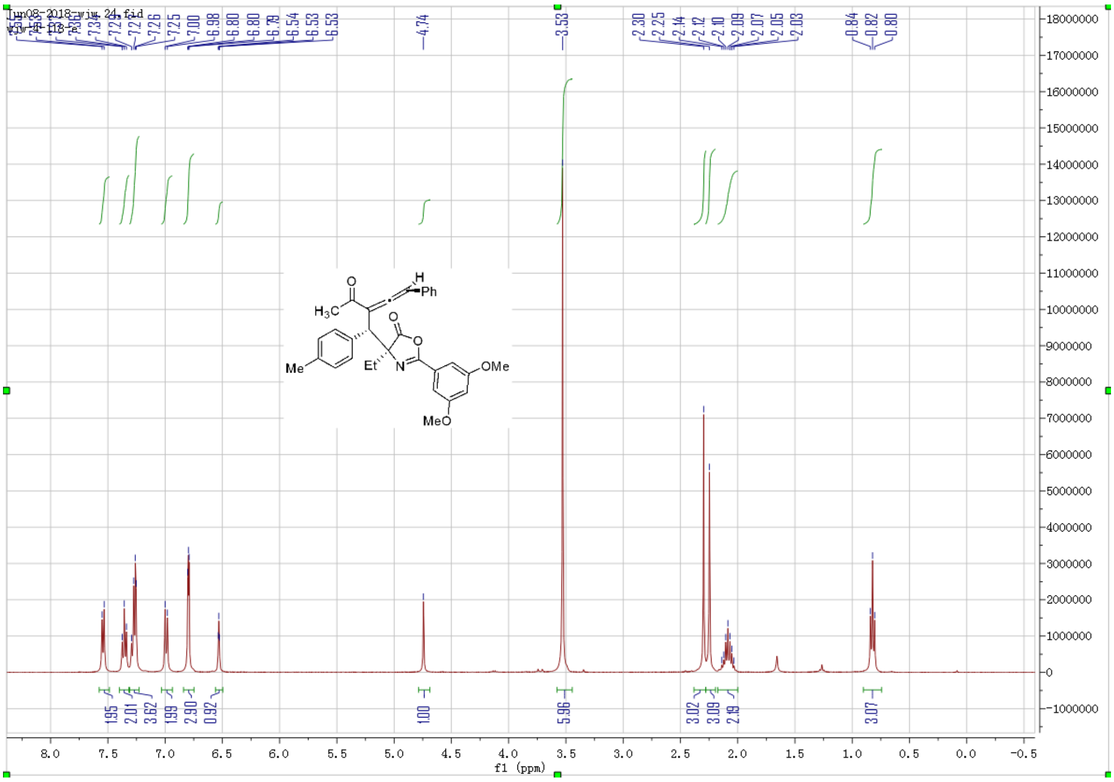

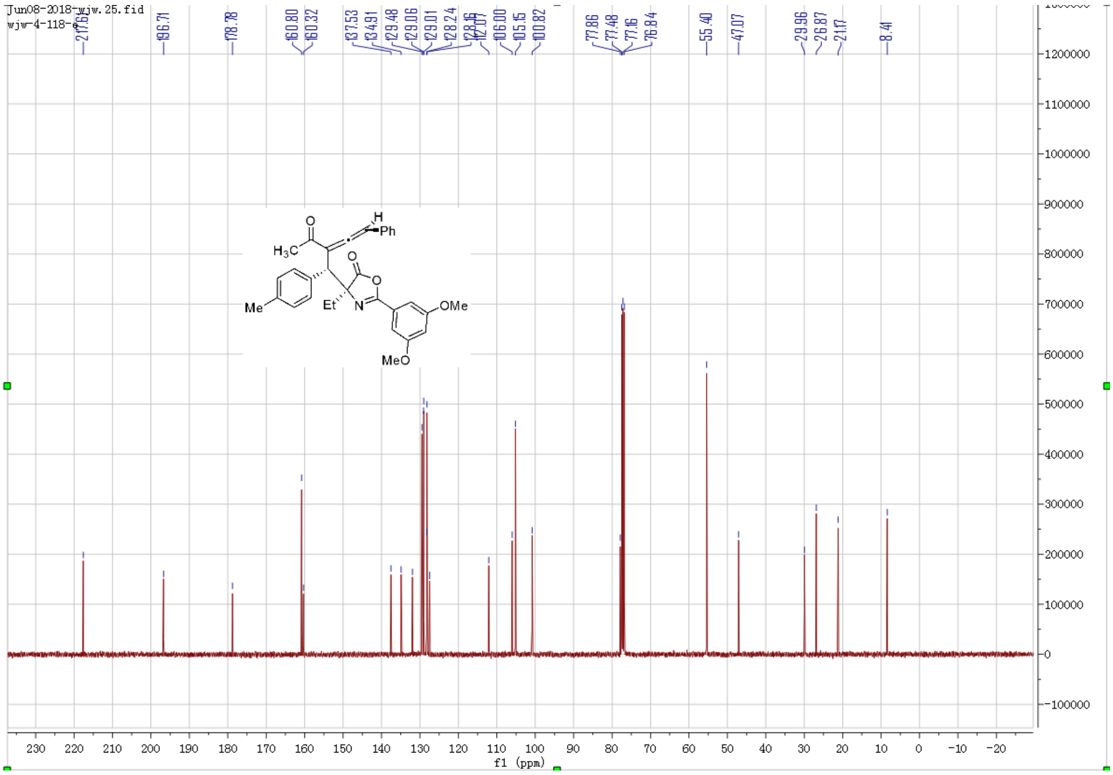

(*S*)-4-((*1R*,*3S*)-2-acetyl-1-(4-fluorophenyl)-4-phenylbuta-2,3-dien-1-yl)-2-(3,5-dimethoxyphenyl)-4-ethyloxazol-5(4H)-one (**4m**)

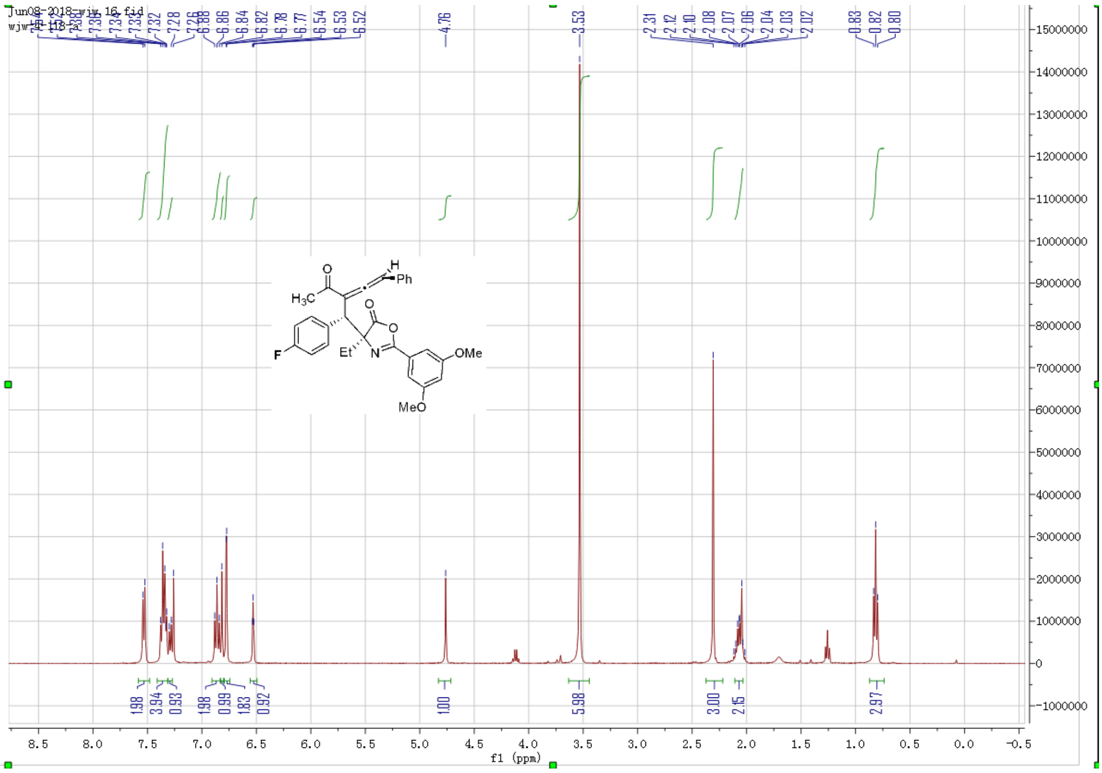

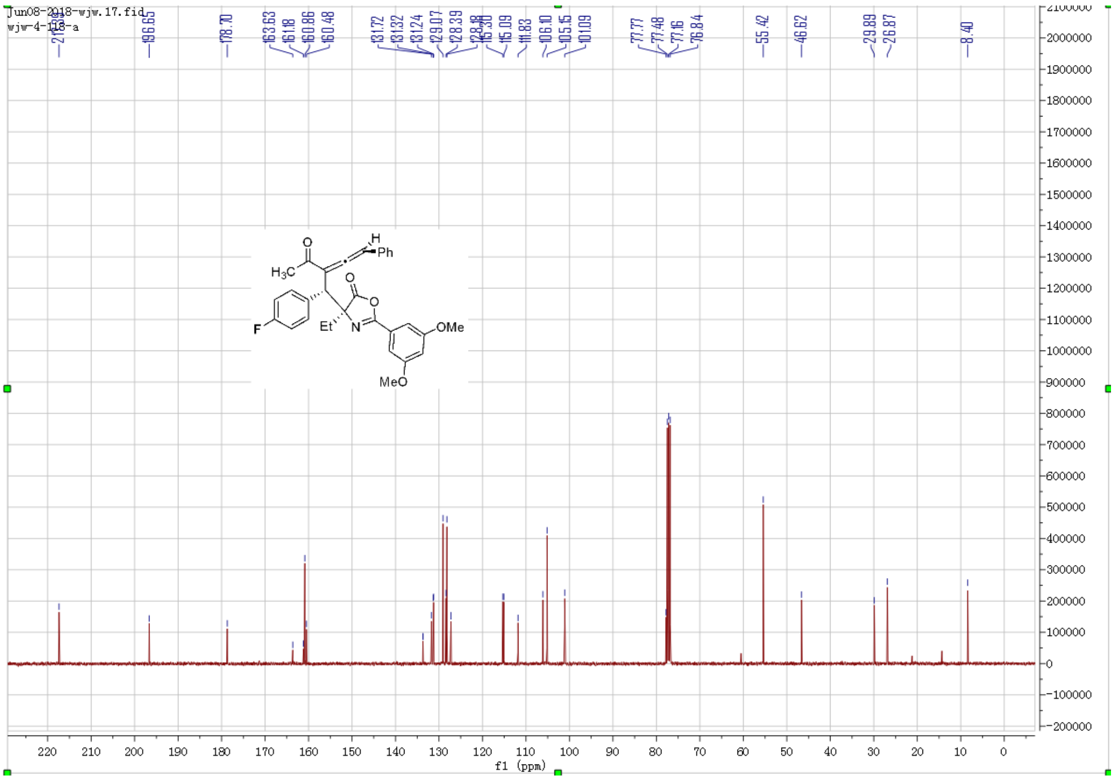

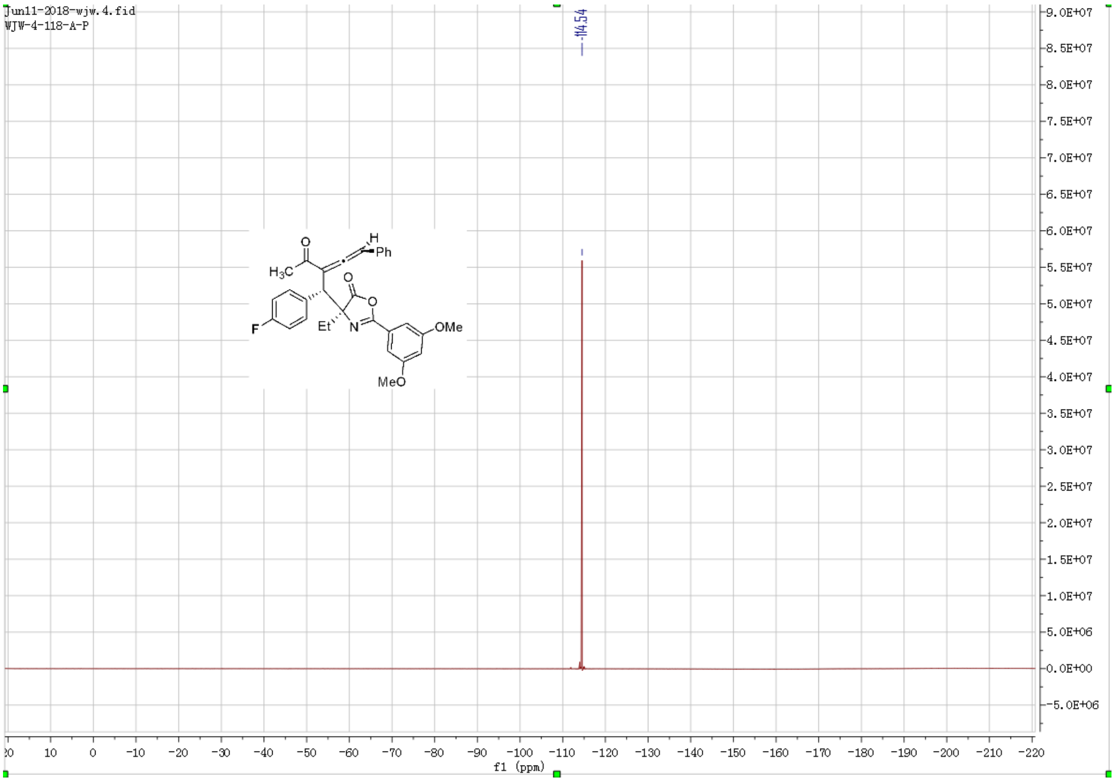

Jun11-2018-wjw.4.fid
WJW-4-118-A-P
-114.54
H
Ph
O
H3C
O
O
F
Et
N
OMe
MeO
f1 (ppm)

(*S*)-4-((*1R*,*3S*)-2-acetyl-1-(4-chlorophenyl)-4-phenylbuta-2,3-dien-1-yl)-2-(3,5-dimethoxyphenyl)-4-ethyloxazol-5(4H)-one (**4n**)

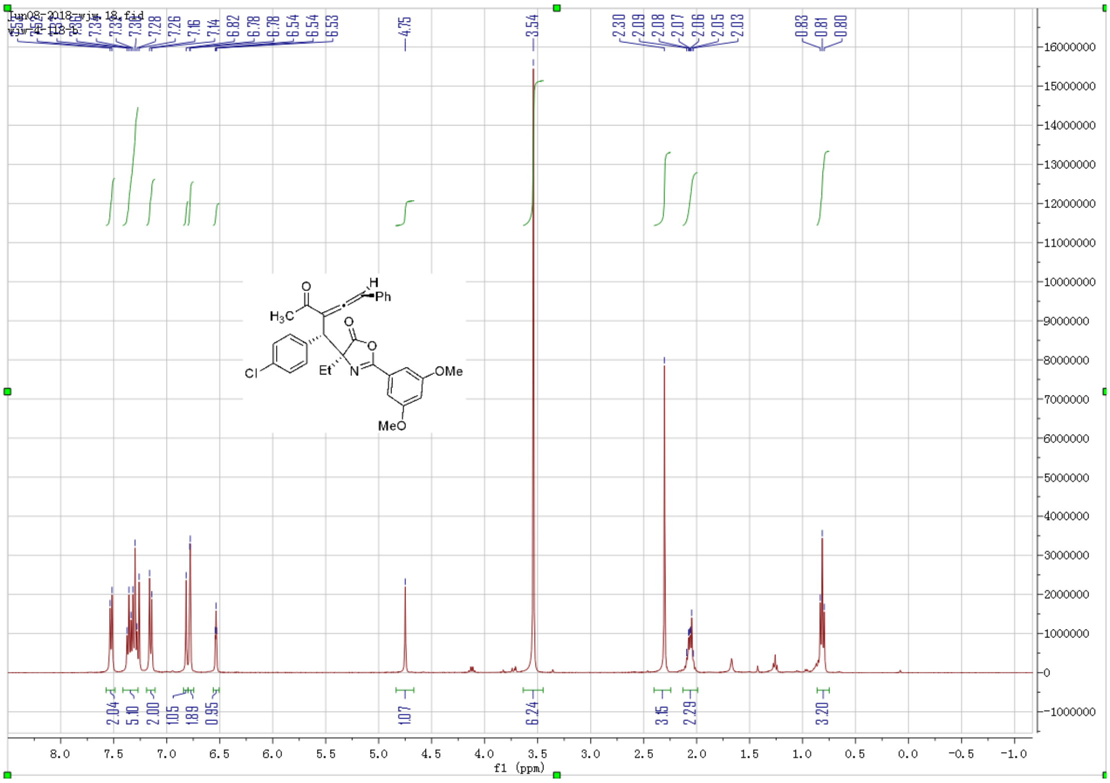

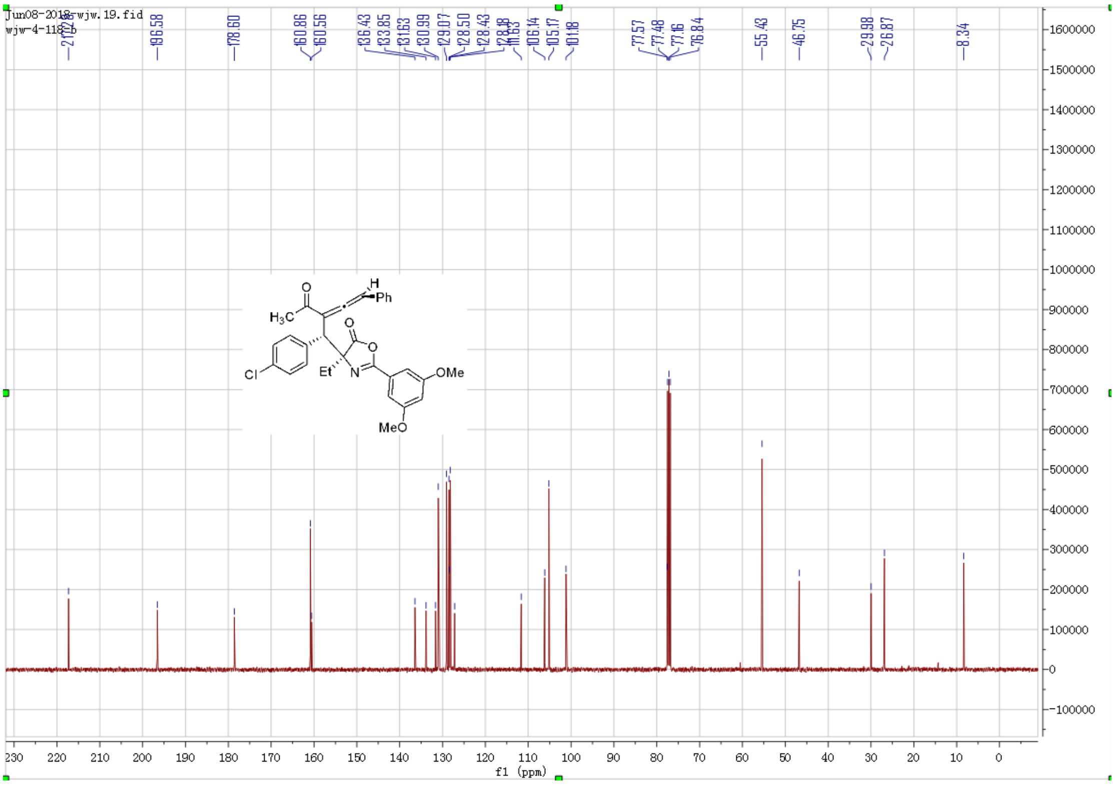

(*S*)-4-((*1R*,*3S*)-2-acetyl-1-(4-methoxyphenyl)-4-phenylbuta-2,3-dien-1-yl)-2-(3,5-dimethoxyphenyl)-4-ethyloxazol-5(4H)-one (**4o**)

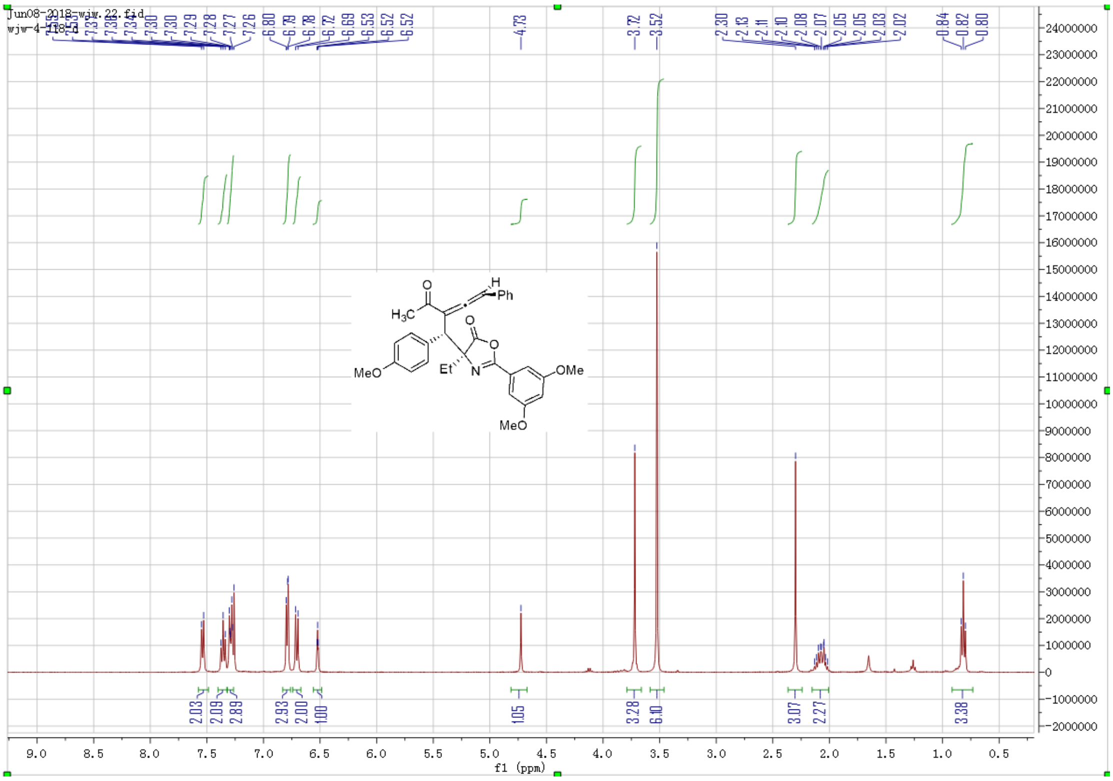

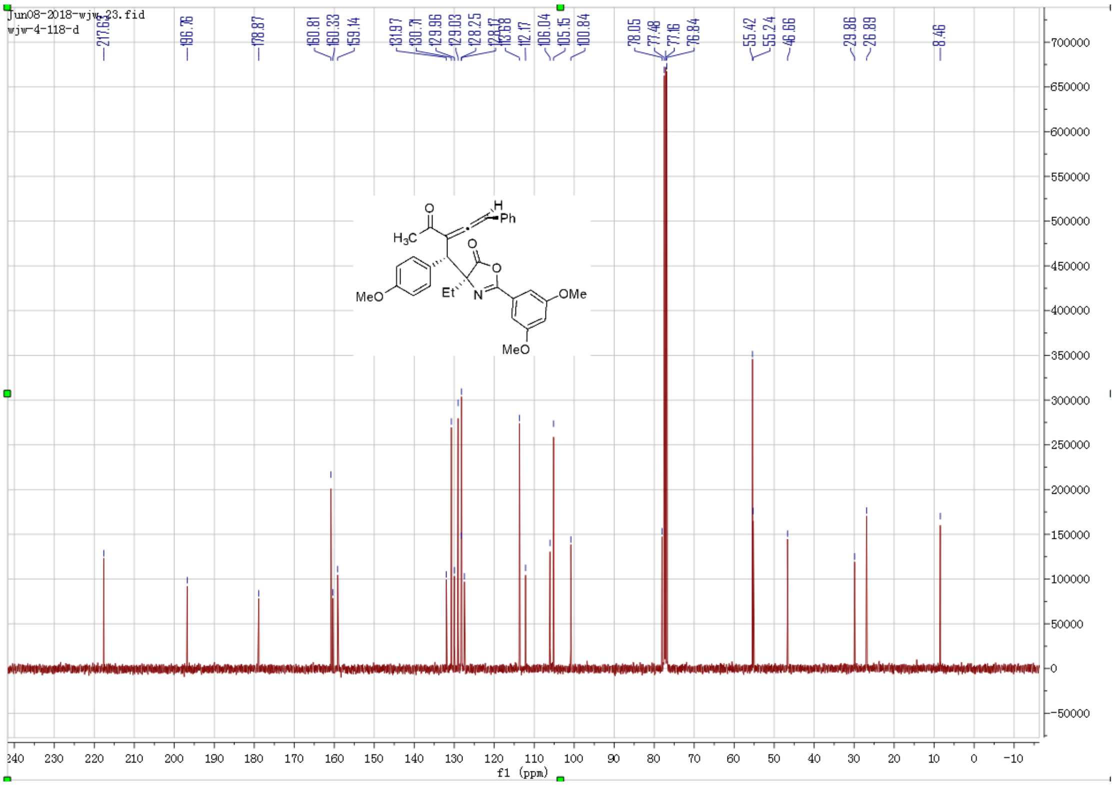

(*S*)-4-((*1R*,*3S*)-2-acetyl-1-(naphthalen-2-yl)-4-phenylbuta-2,3-dien-1-yl)-2-(3,5-dimethoxyphenyl)-4-ethyloxazol-5(4H)-one (**4p**)

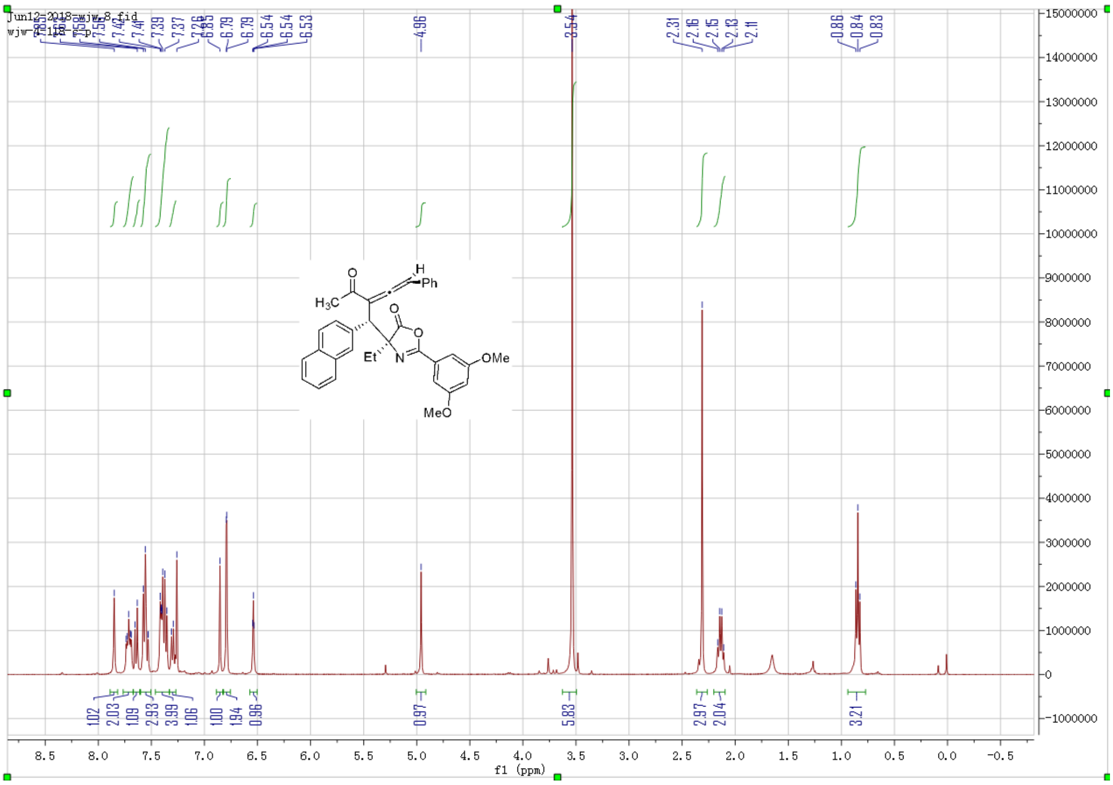

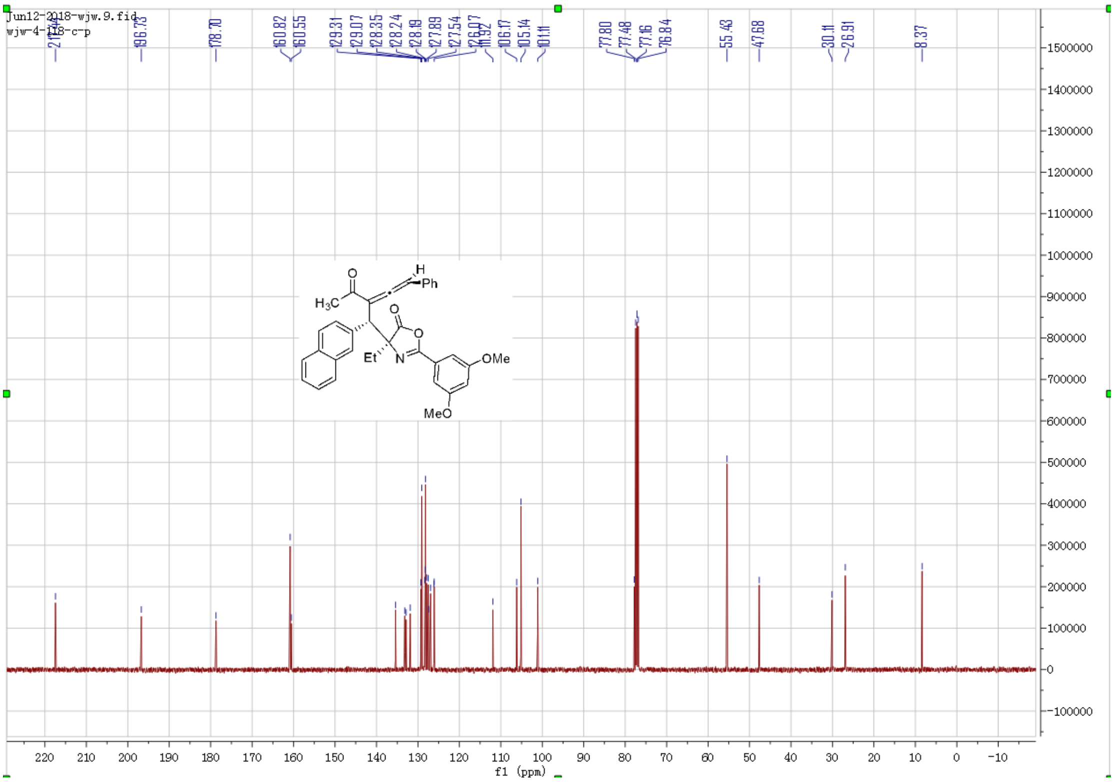

(*S*)-4-((*1R*,*3S*)-2-acetyl-1-cyclohexyl-4-phenylbuta-2,3-dien-1-yl)-2-(3,5-dimethoxyphenyl)-4-ethyloxazol-5(4H)-one (**4q**)

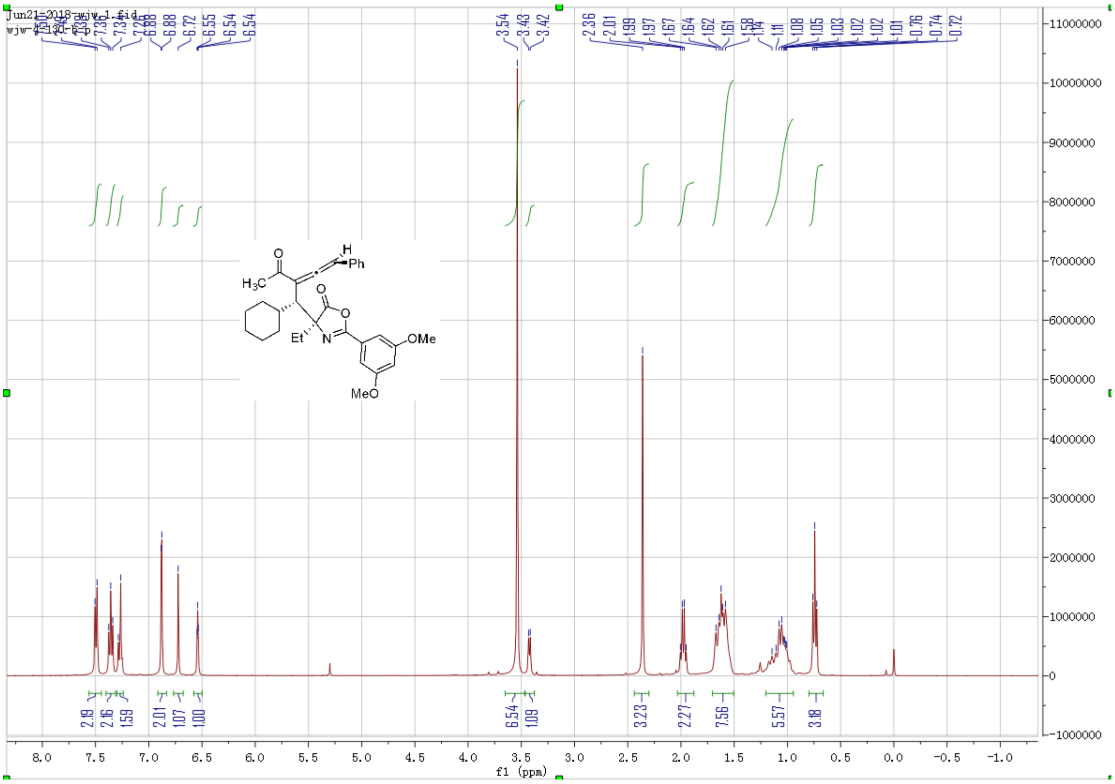

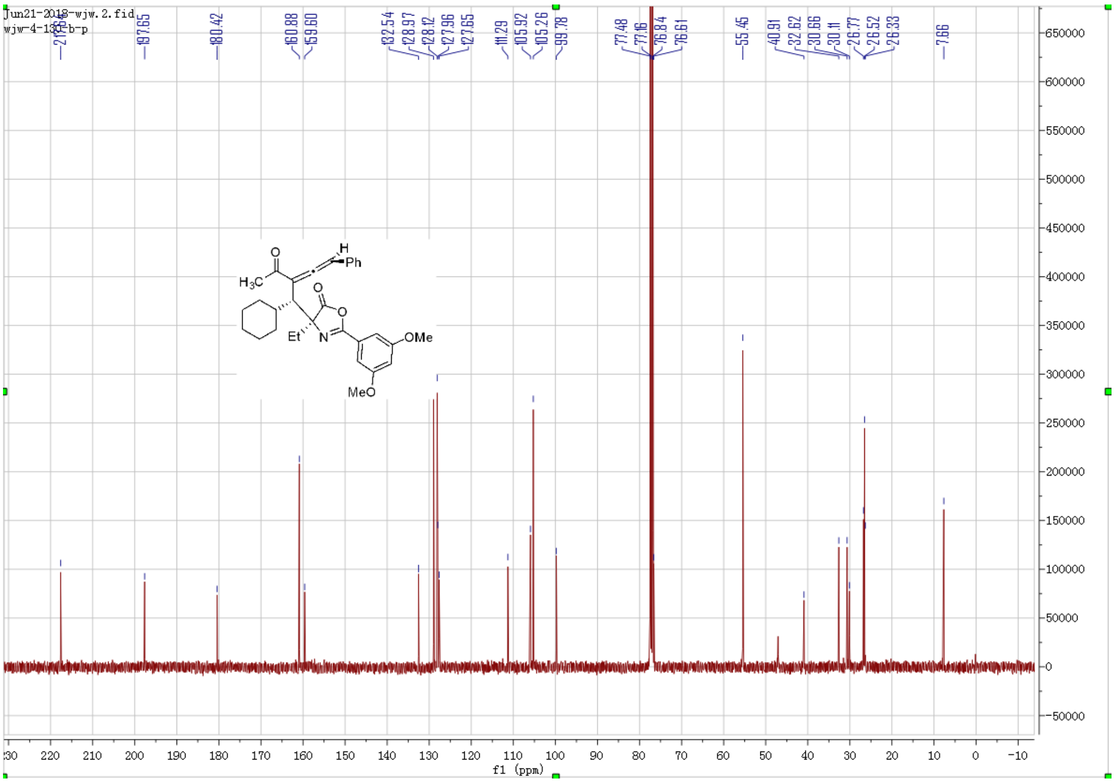

(*S*)-4-((*1R*,*3S*)-2-acetyl-1,4-diphenylbuta-2,3-dien-1-yl)-4-butyl-2-(3,5-dimethoxyphenyl)oxazol-5(4H)-one (**4v**)

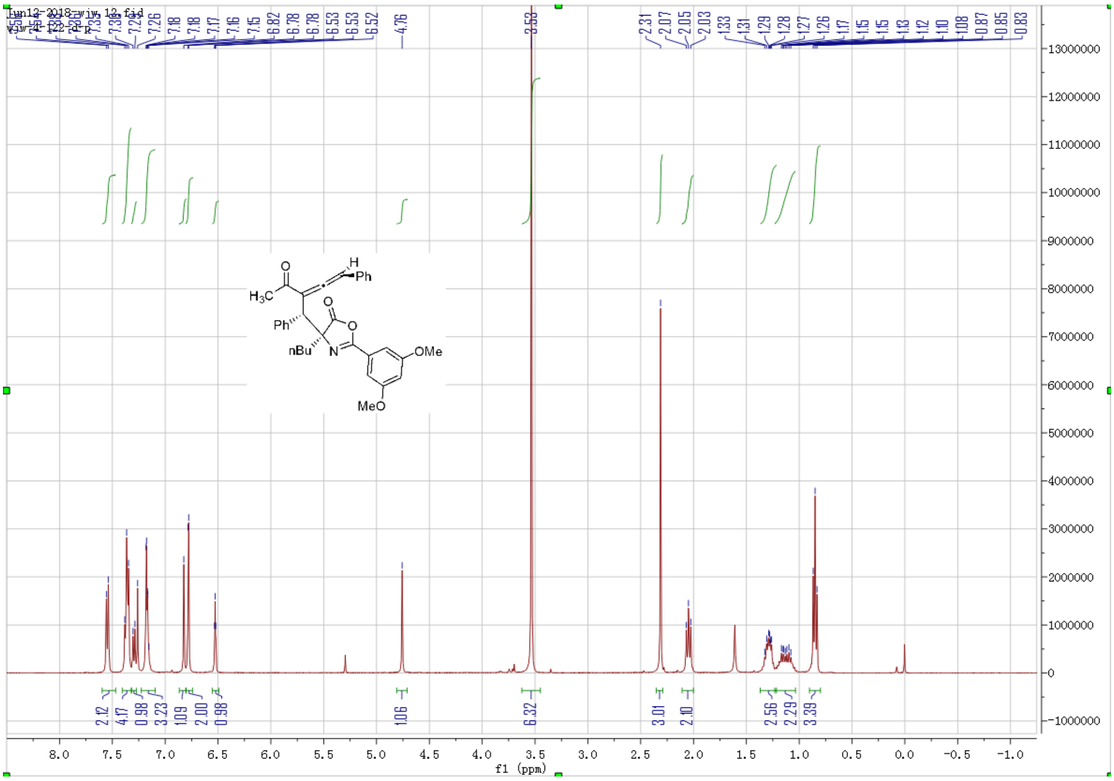

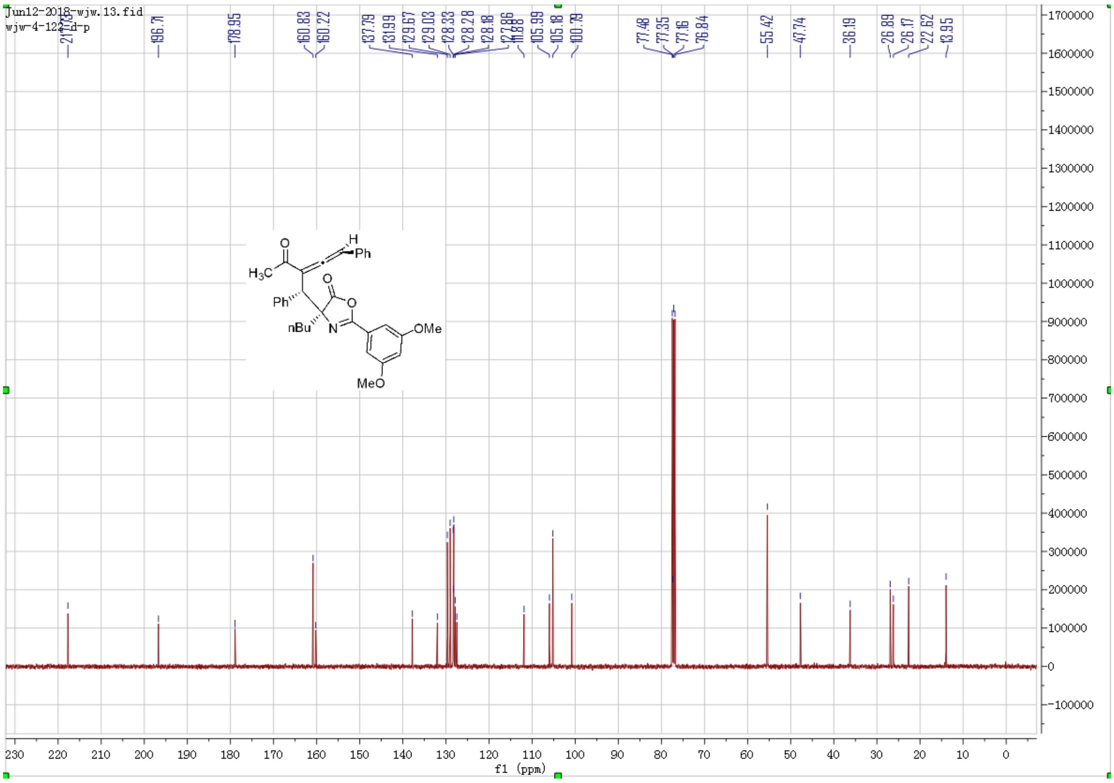

(*S*)-4-((*1R*,*3S*)-2-acetyl-1,4-diphenylbuta-2,3-dien-1-yl)-4-benzyl-2-(3,5-dimethoxyphenyl)oxazol-5(4H)-one (**4w**)

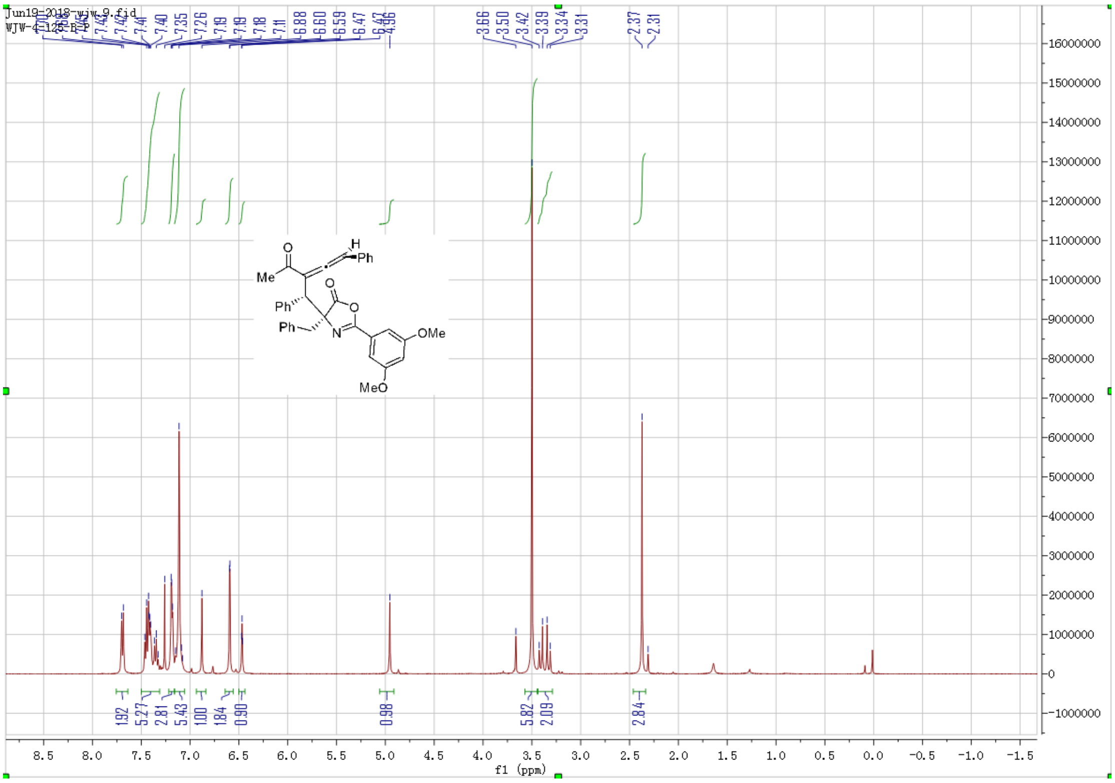

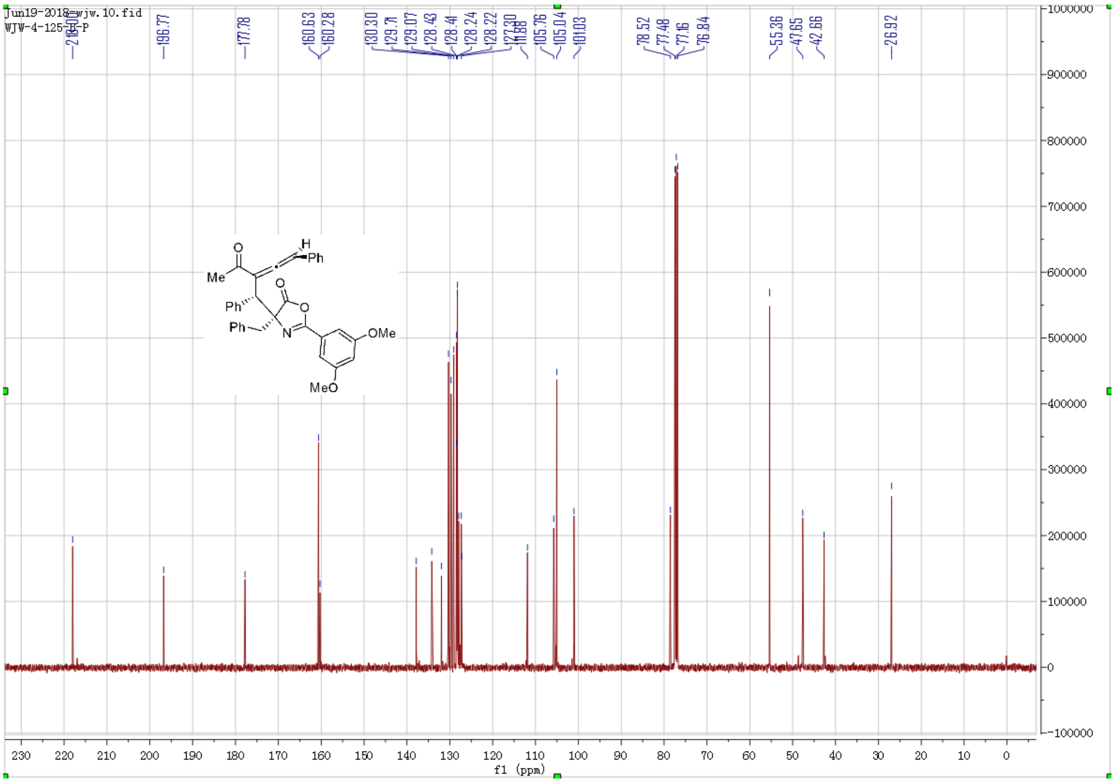

(*S*)-4-((*1R*,*3S*)-2-acetyl-1,4-diphenylbuta-2,3-dien-1-yl)-2-(3,5-dimethoxyphenyl)-4-(2-(methylthio)ethyl)oxazol-5(4H)-one (**4x**)

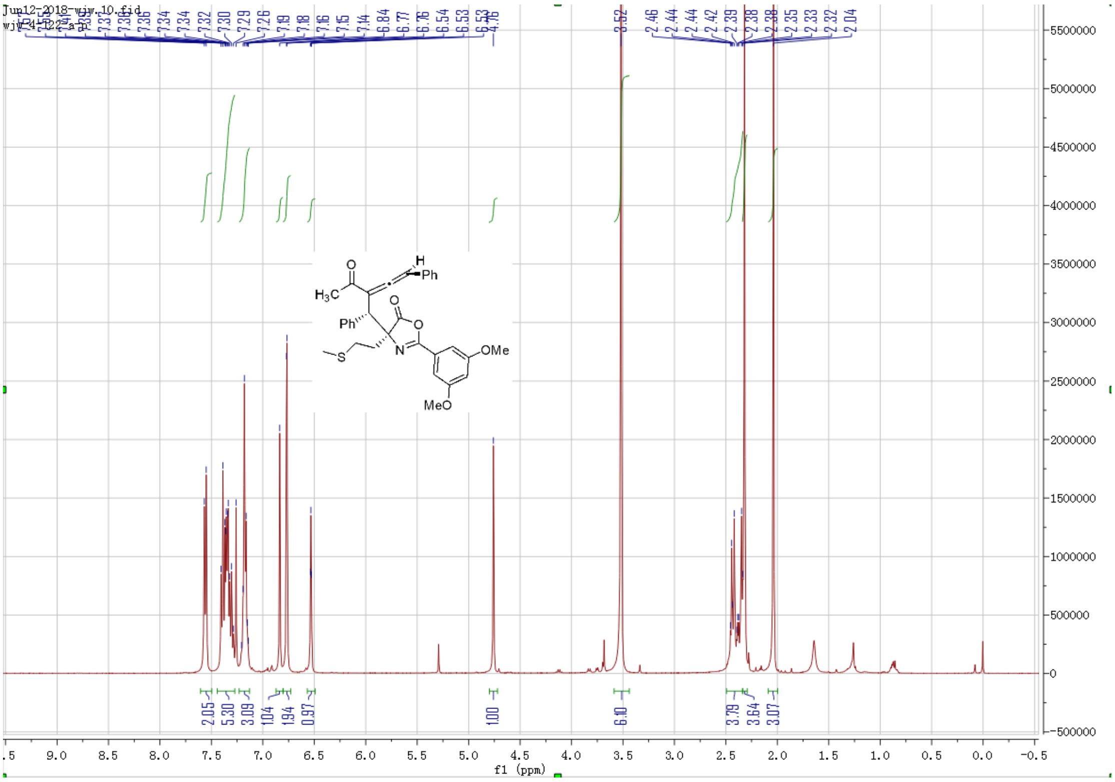

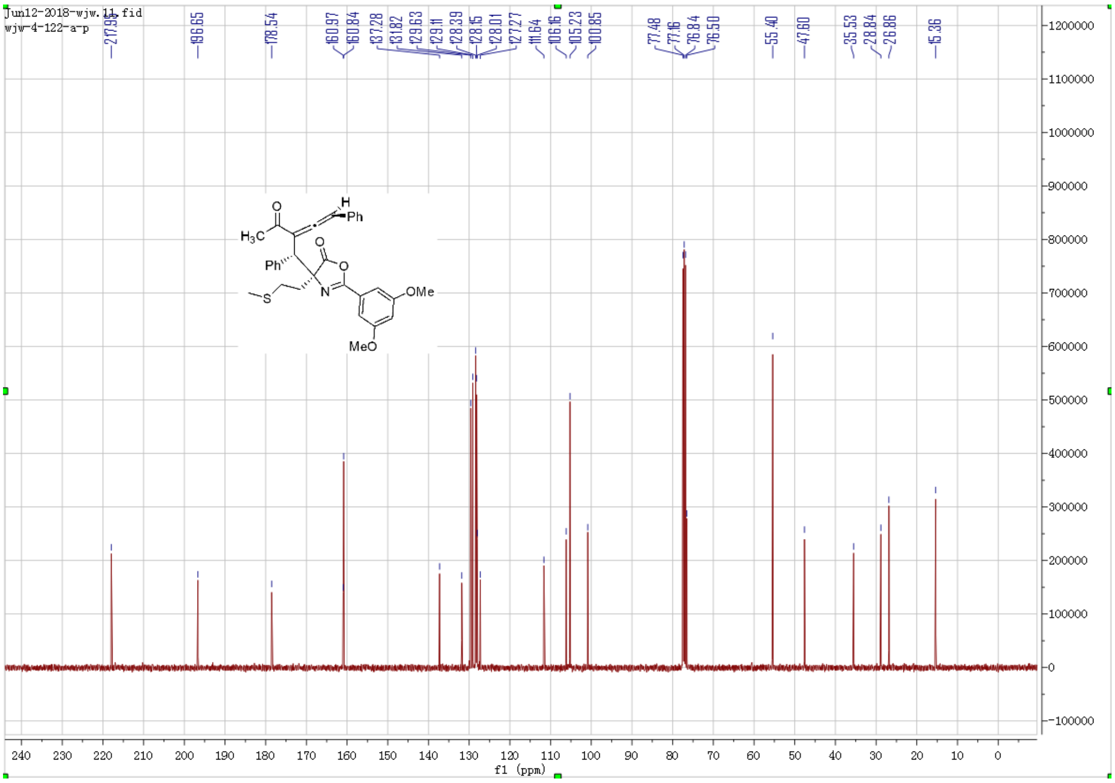

tert-butyl(4-((*S*)-4-((*1R*,*3S*)-2-acetyl-1,4-diphenylbuta-2,3-dien-1-yl)-2-(3,5-dimethoxyphenyl)-5-oxo-4,5-dihydrooxazol-4-yl)butyl)carbamate (**4y**)

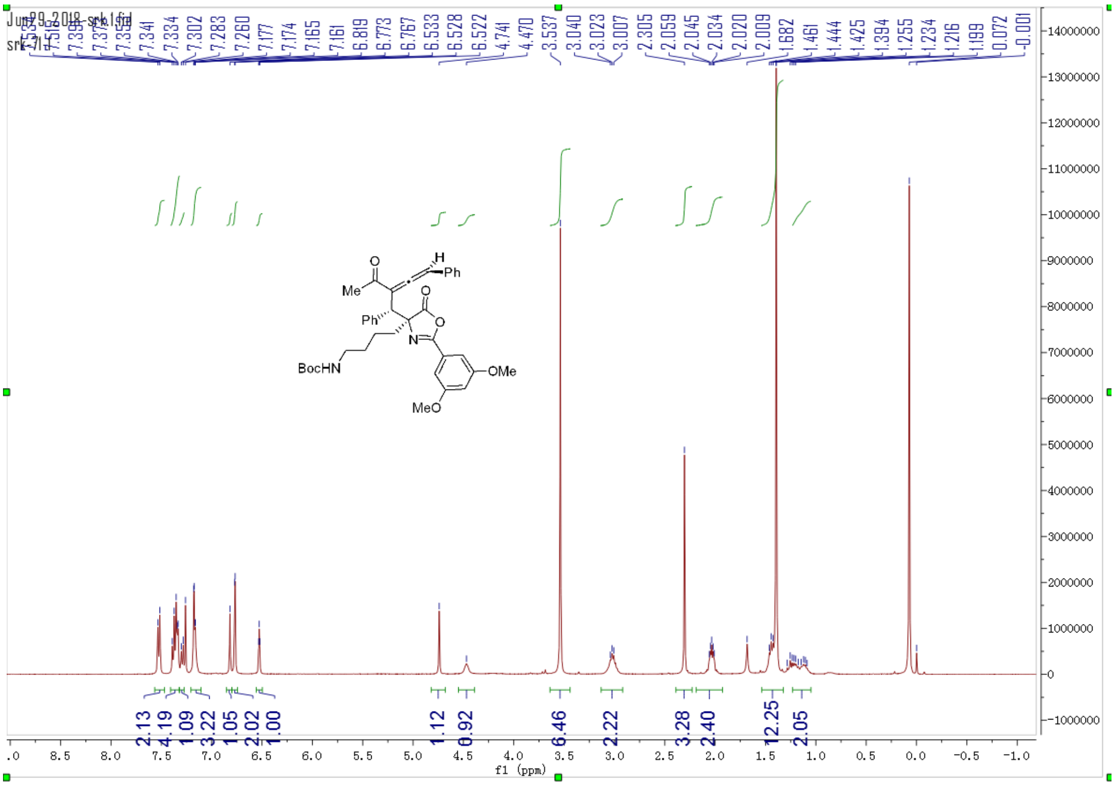

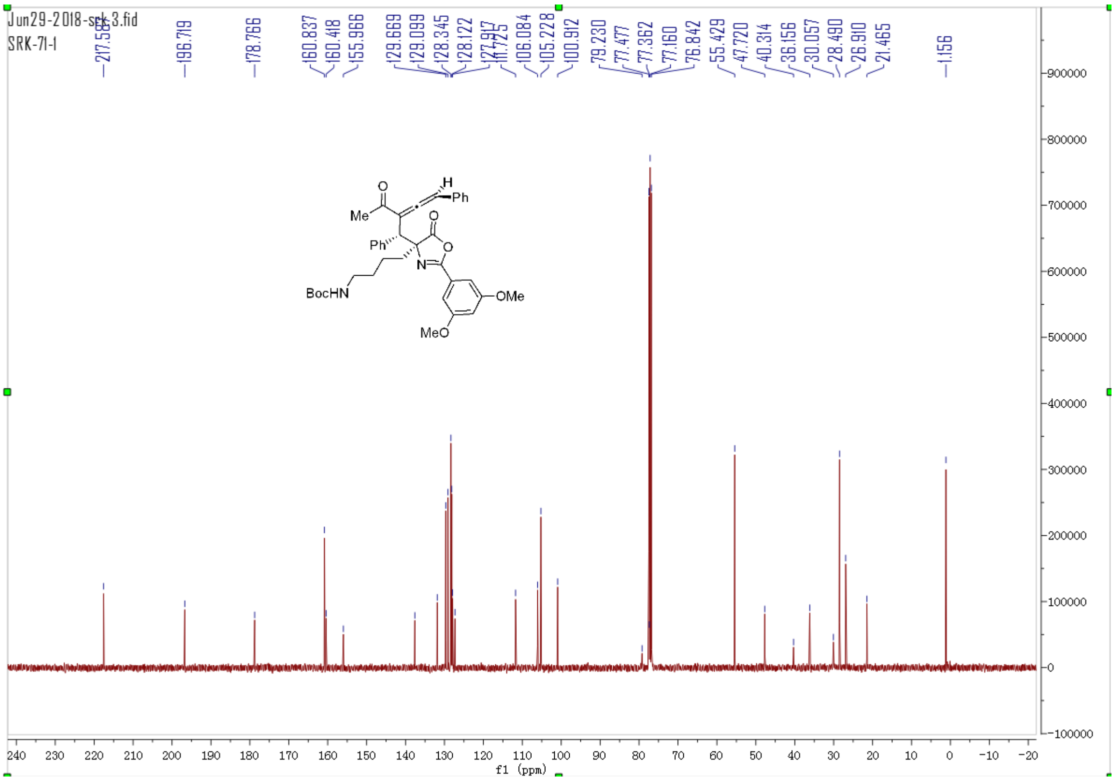

3-benzyl-5-phenylpent-4-yn-2-one (**5a**)

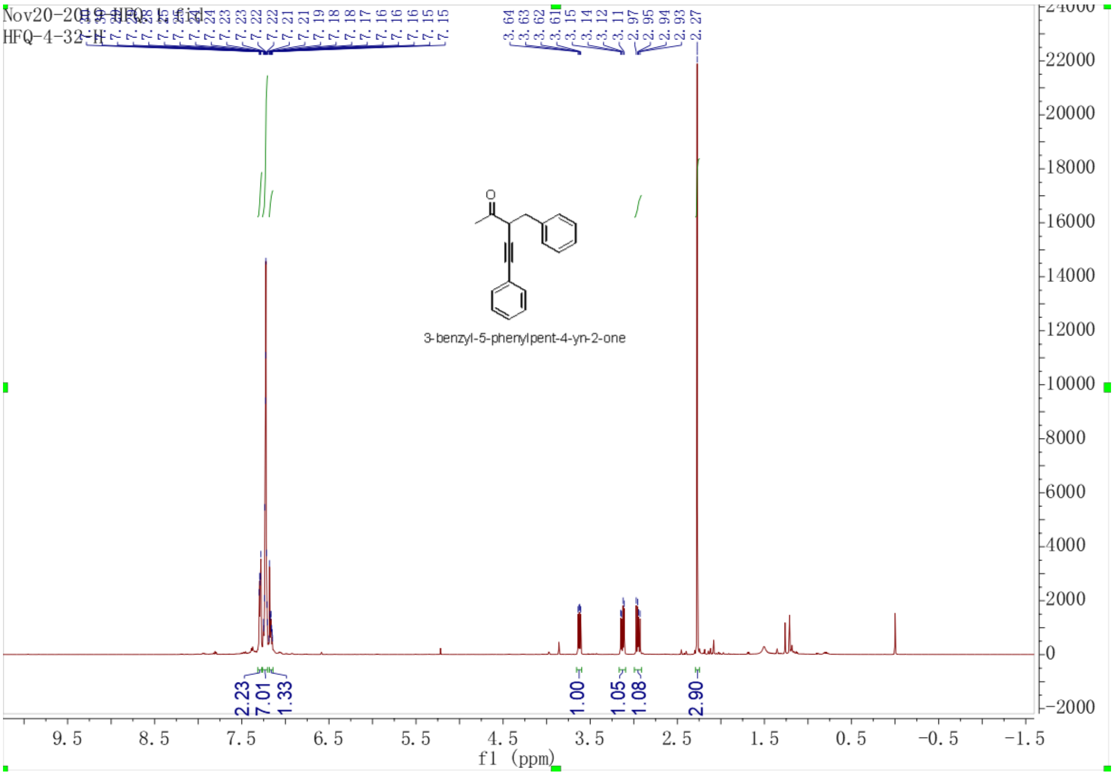

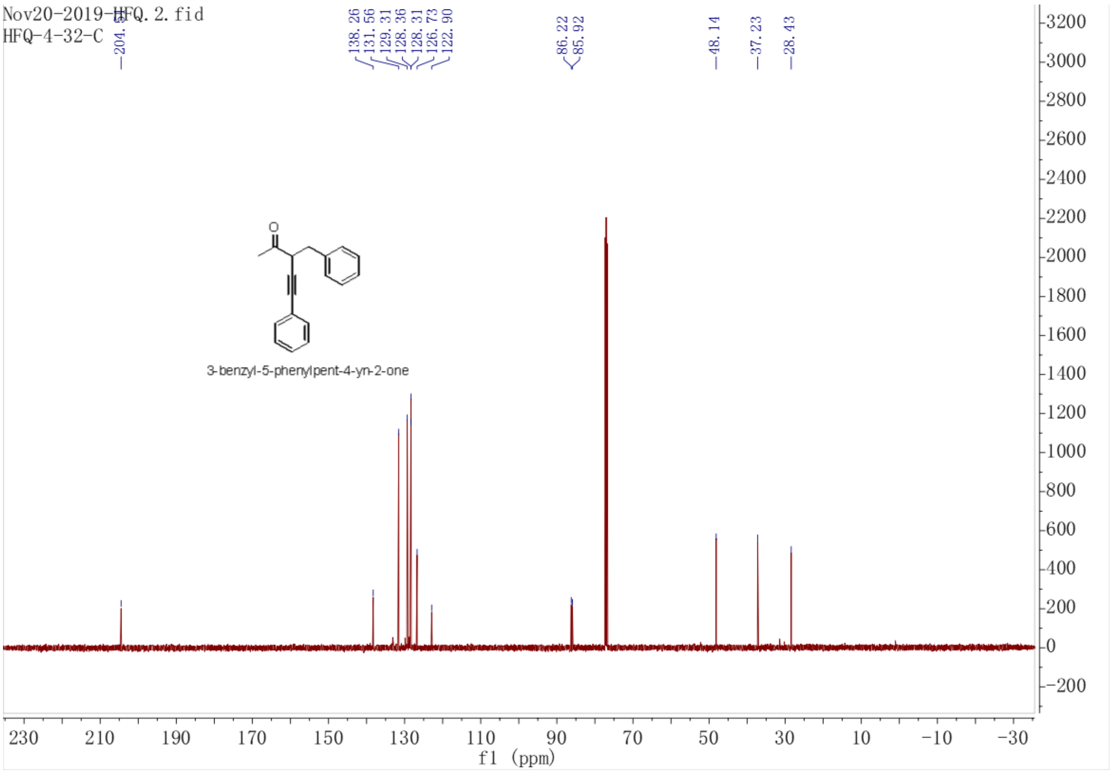

3-benzyl-5-phenylpenta-3,4-dien-2-one (**6a**)

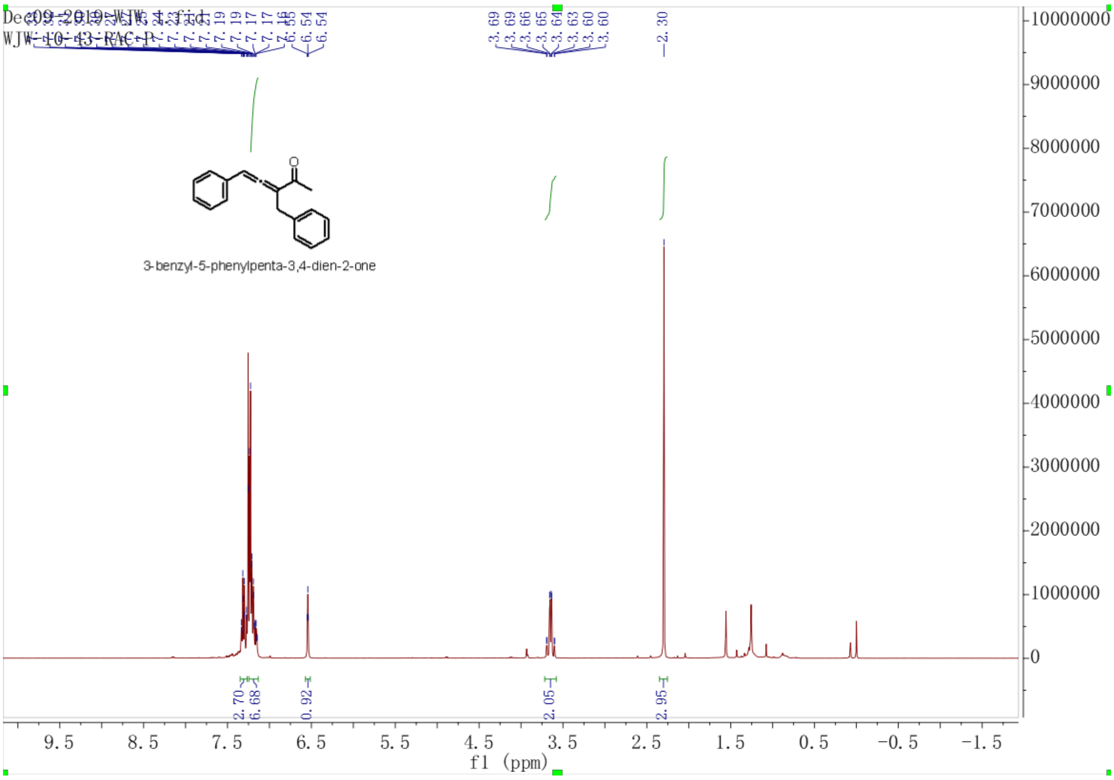

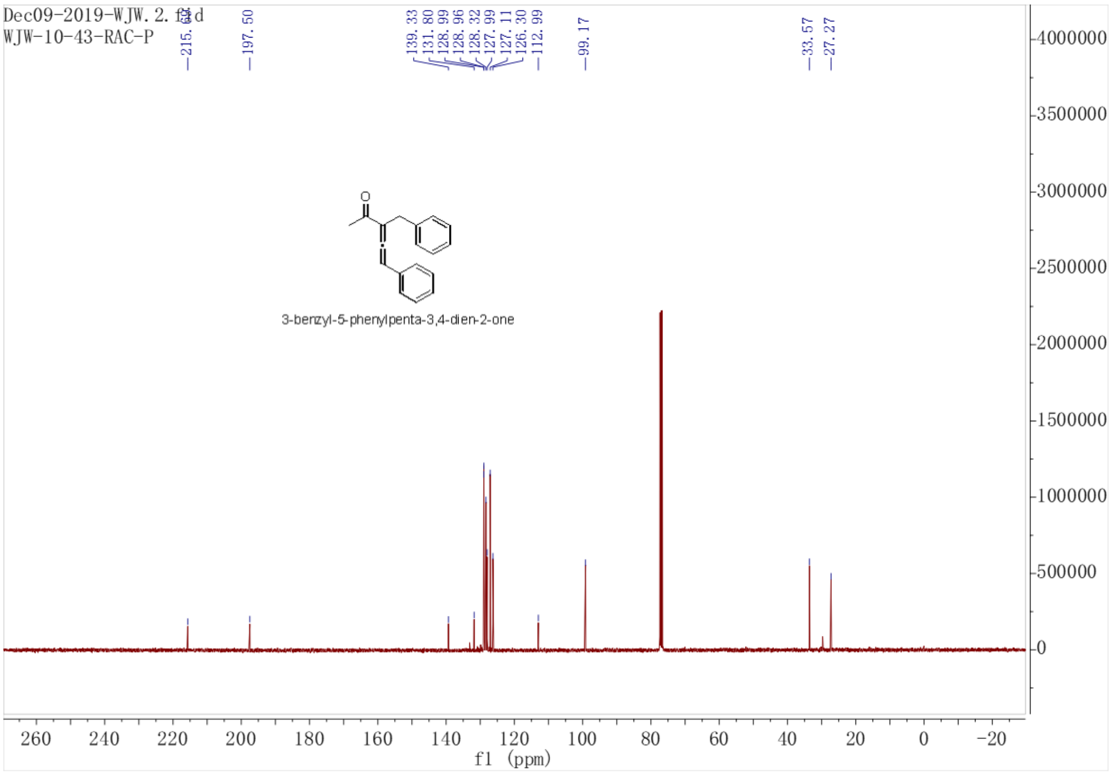

(*S*)-2-(3,5-dimethoxyphenyl)-4-ethyl-4-((*R*)-(2-methyl-5-phenylfuran-3-yl)(phenyl)methyl)oxazol-5(4H)-one (**7a**)

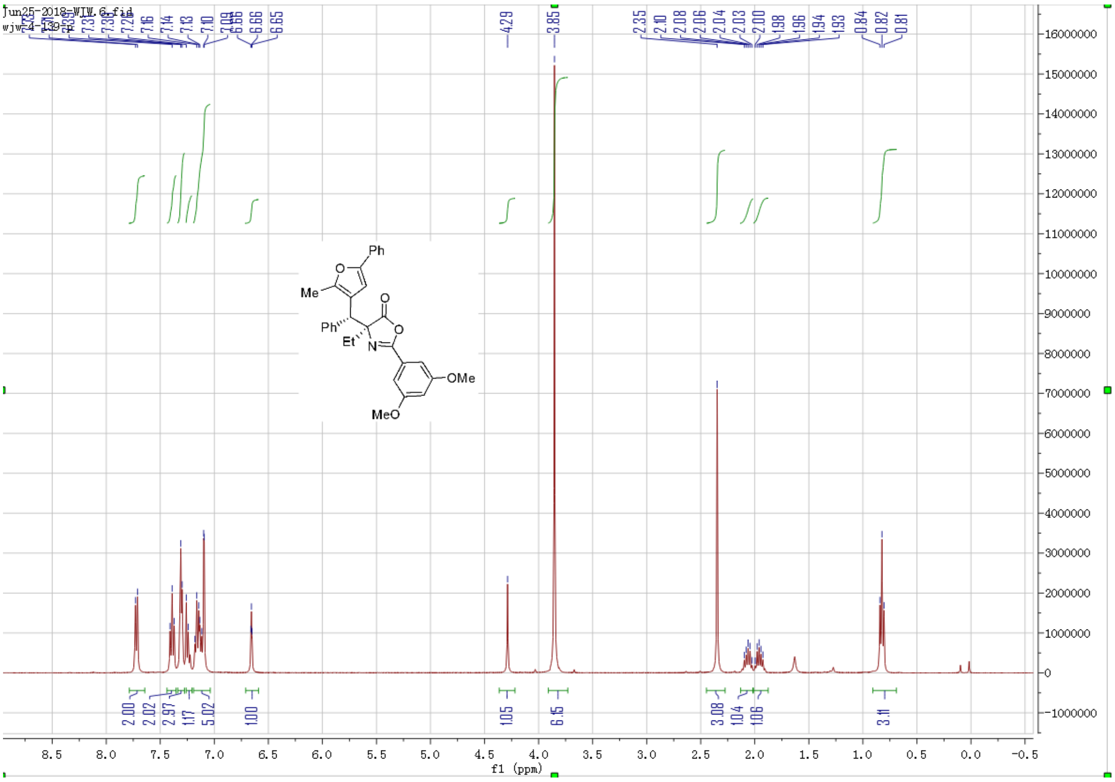

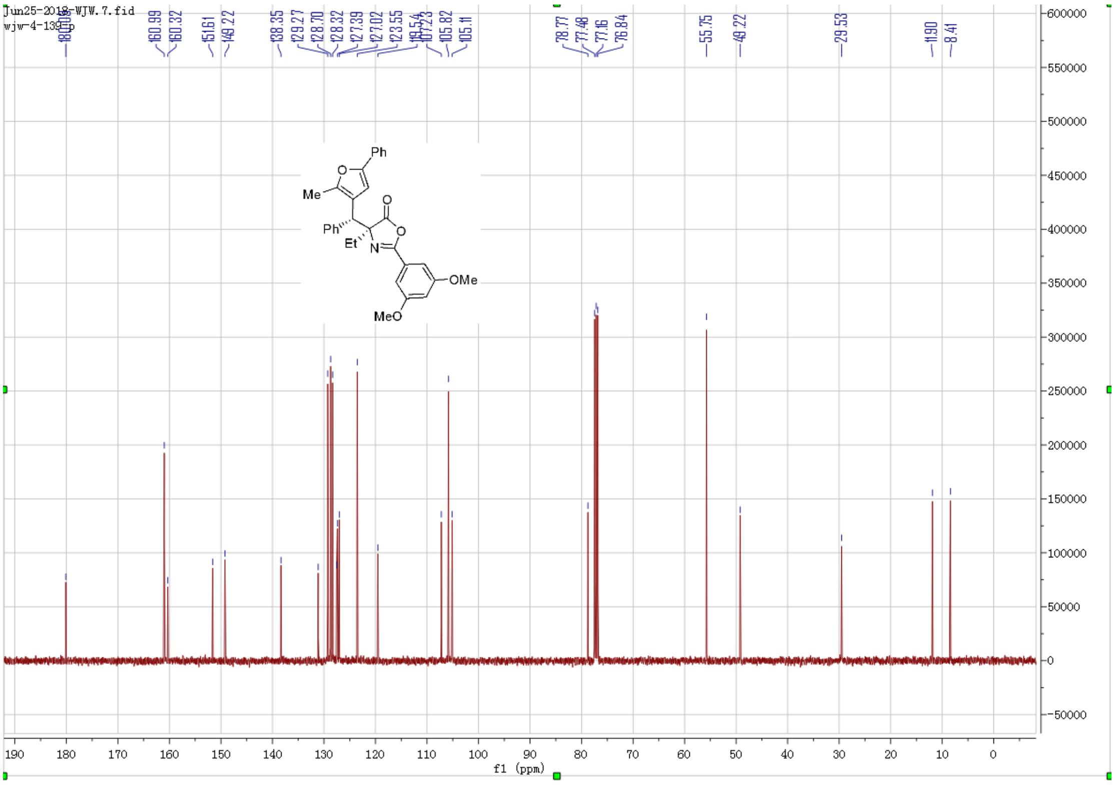

Methyl-(*S*)-2-(3,5-dimethoxybenzamido)-2-((*R*)-(2-methyl-5-phenylfuran-3-yl)(phenyl)methyl)butanoate (**8a**)

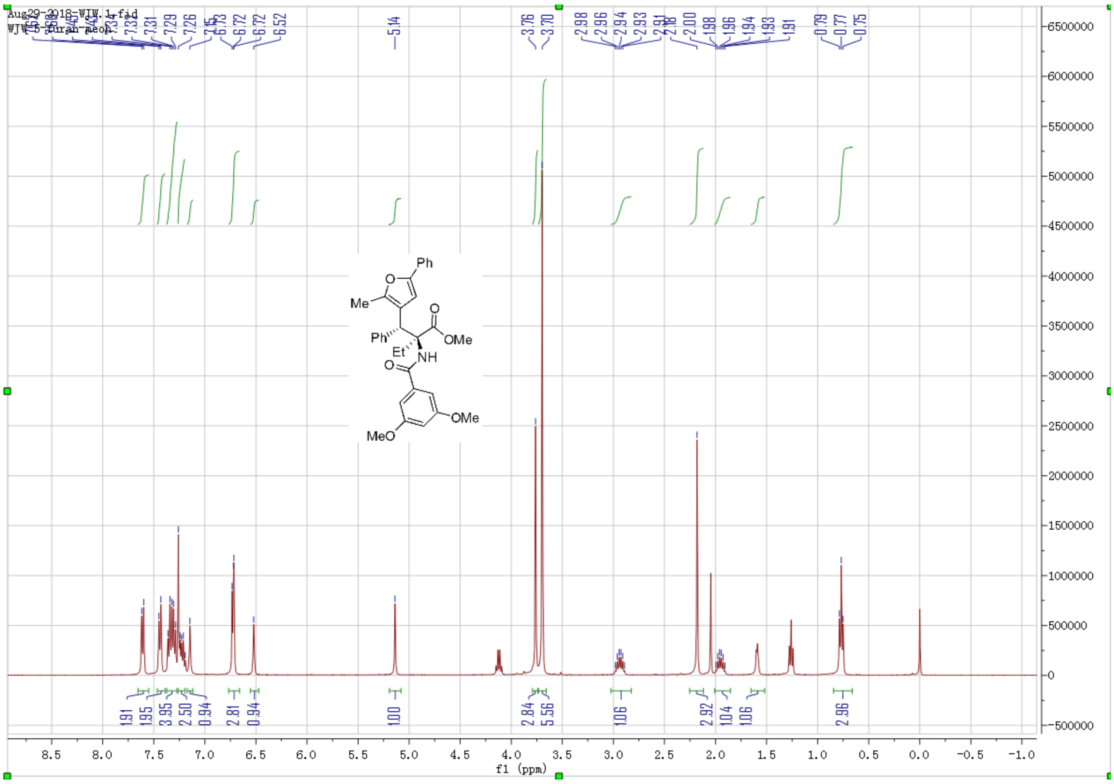

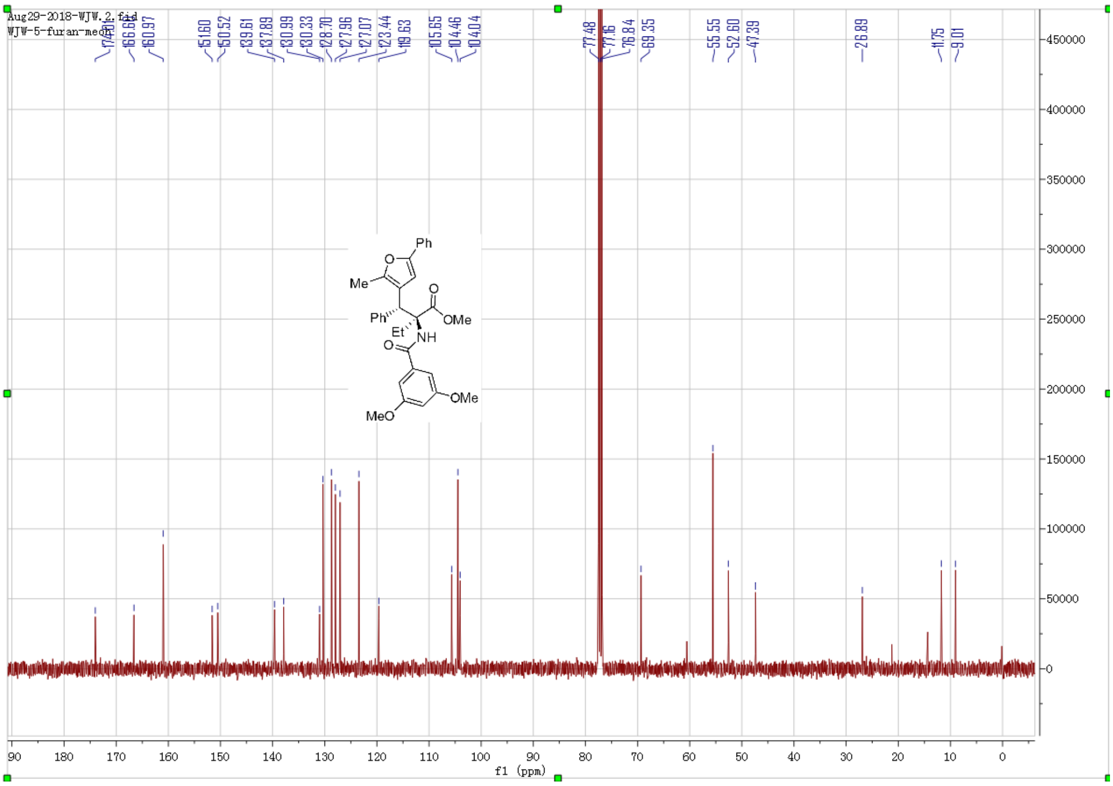

N-((*3S*,*4R*,*6R*)-3-ethyl-6-methyl-2-oxo-4-phenyl-5-((R)-2-phenylvinylidene)tetrahydro-2H-pyran-3-yl)-3,5-dimethoxybenzamide (**9a**)

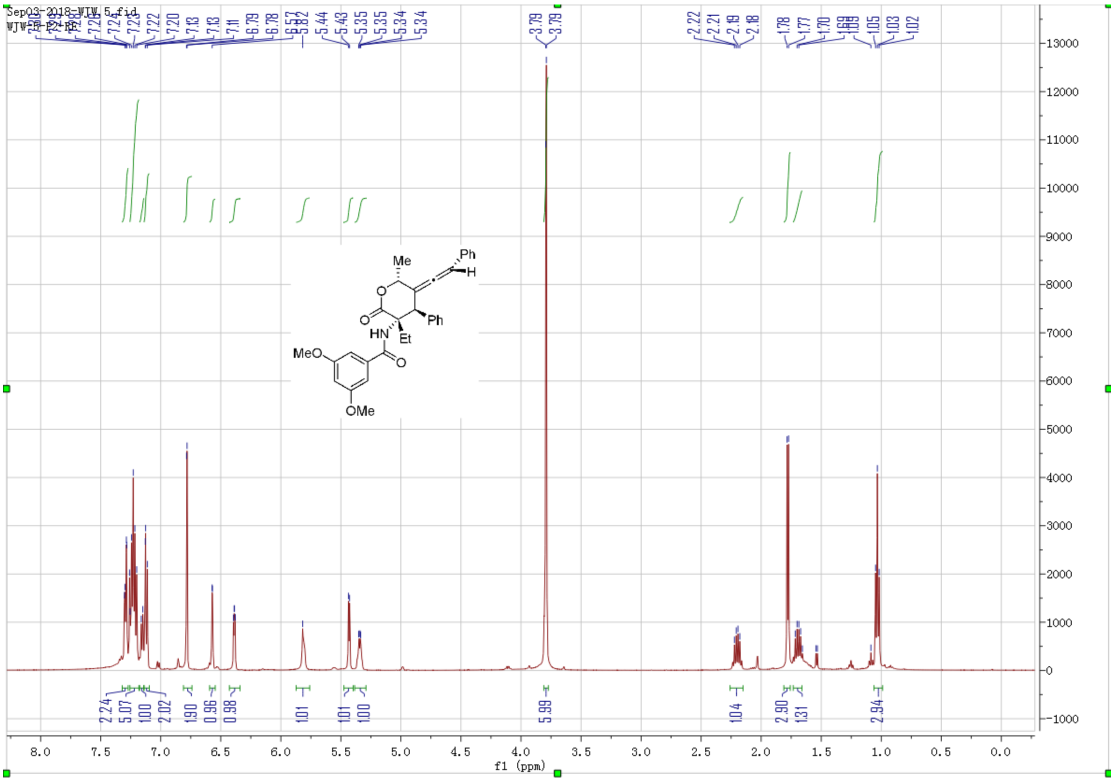

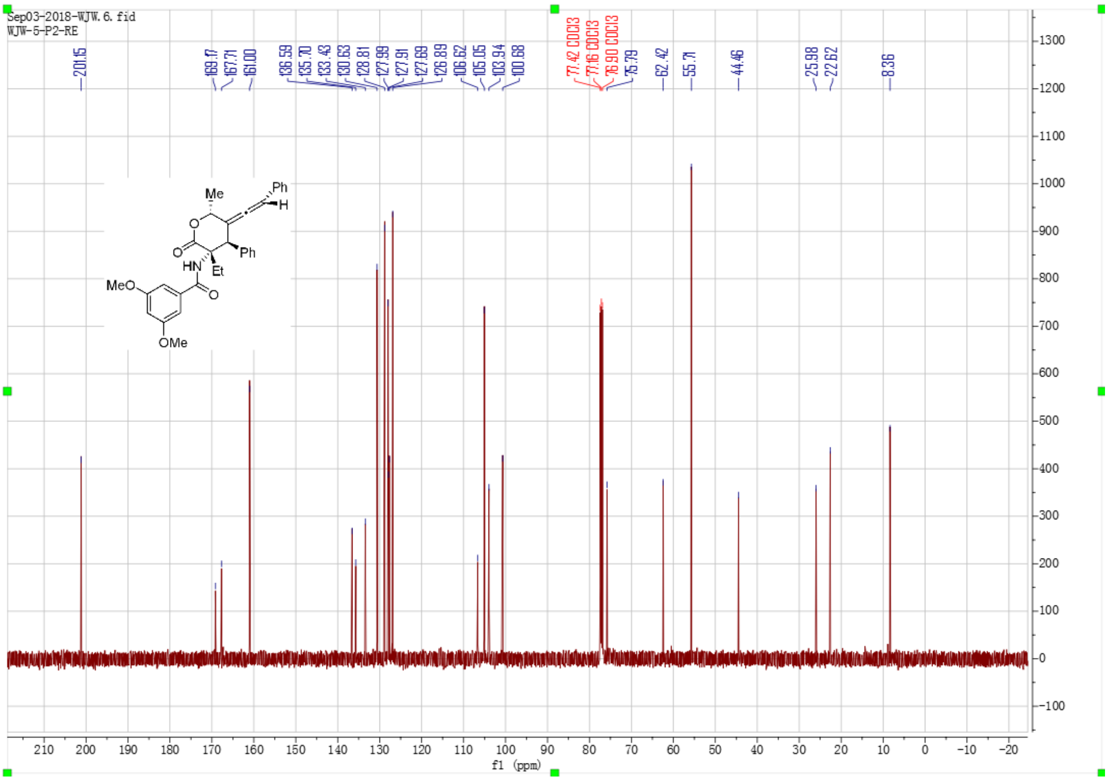

N-((3*S*,4*R*,6*R*)-5-((*R*)-1-hydroxyethyl)-4,7-diphenyl-3-(propylcarbamoyl)hepta-5,6-dien-3-yl)-3,5-dimethoxybenzamide (**10a**)

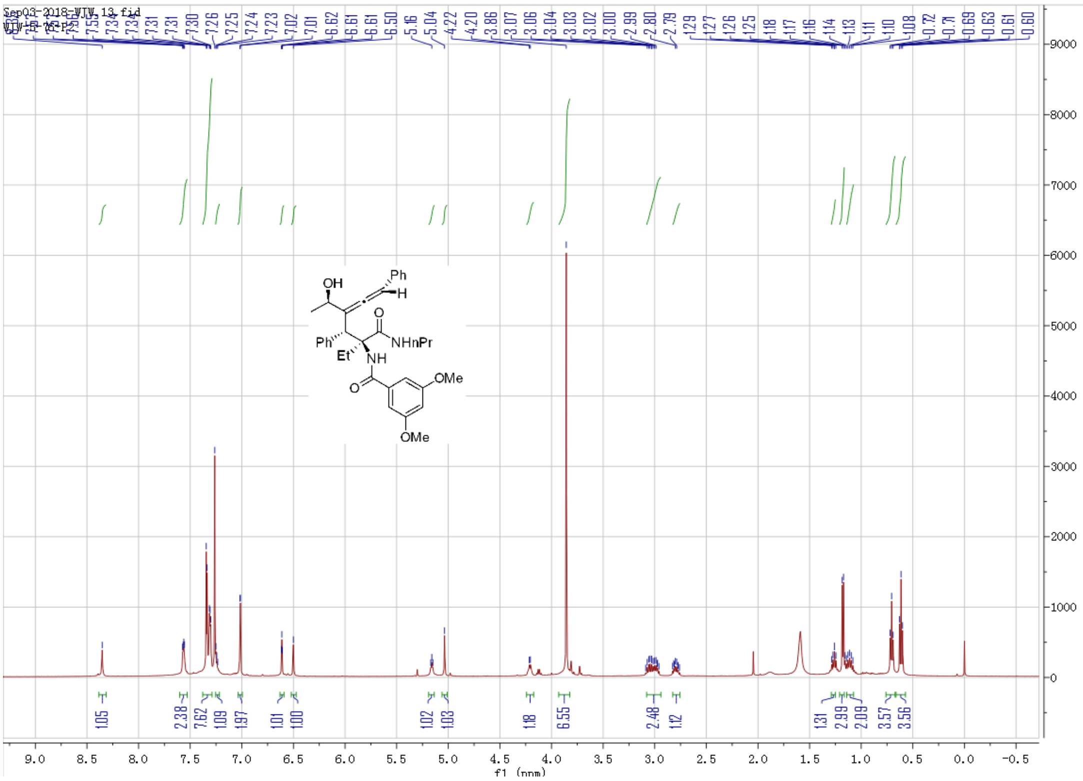

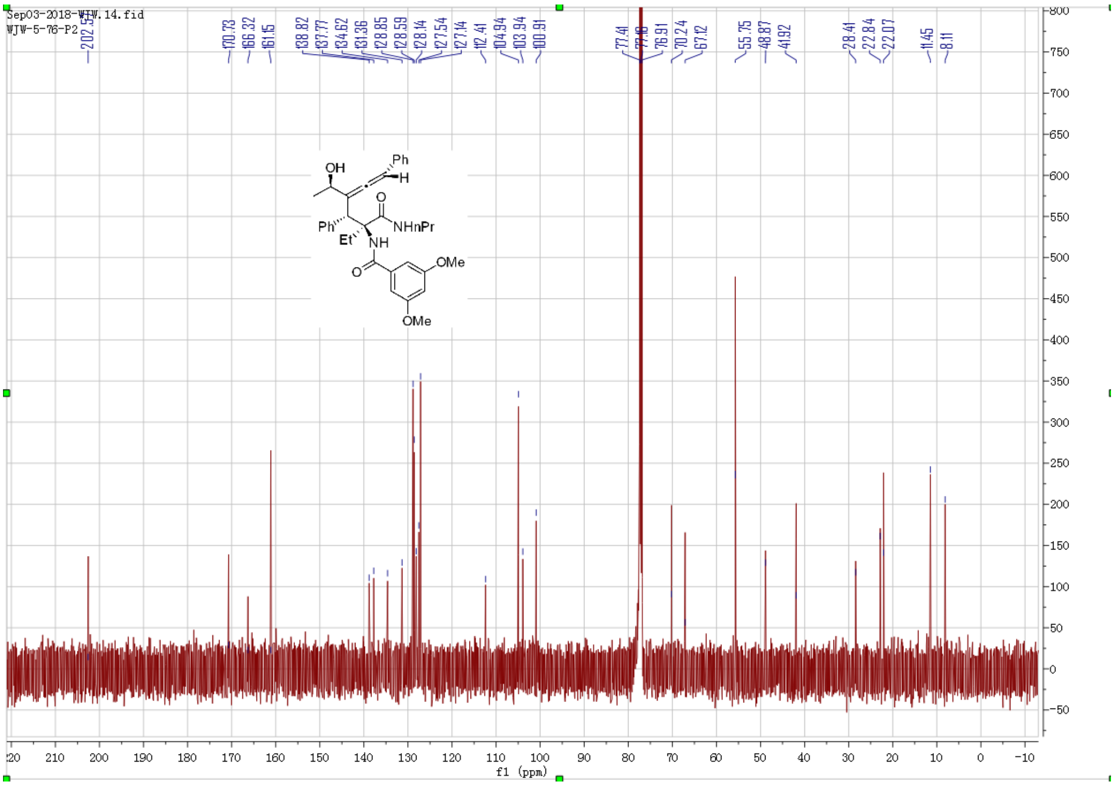

3,5-dimethoxy-*N*-((*1R*,*2S*)-1-((*2R*,*5S*)-2-methyl-5-phenyl-2,5-dihydrofuran-3-yl)-1-phenyl-2-(propylcarbamoyl)butan-2-yl)benzamide (**11a**)

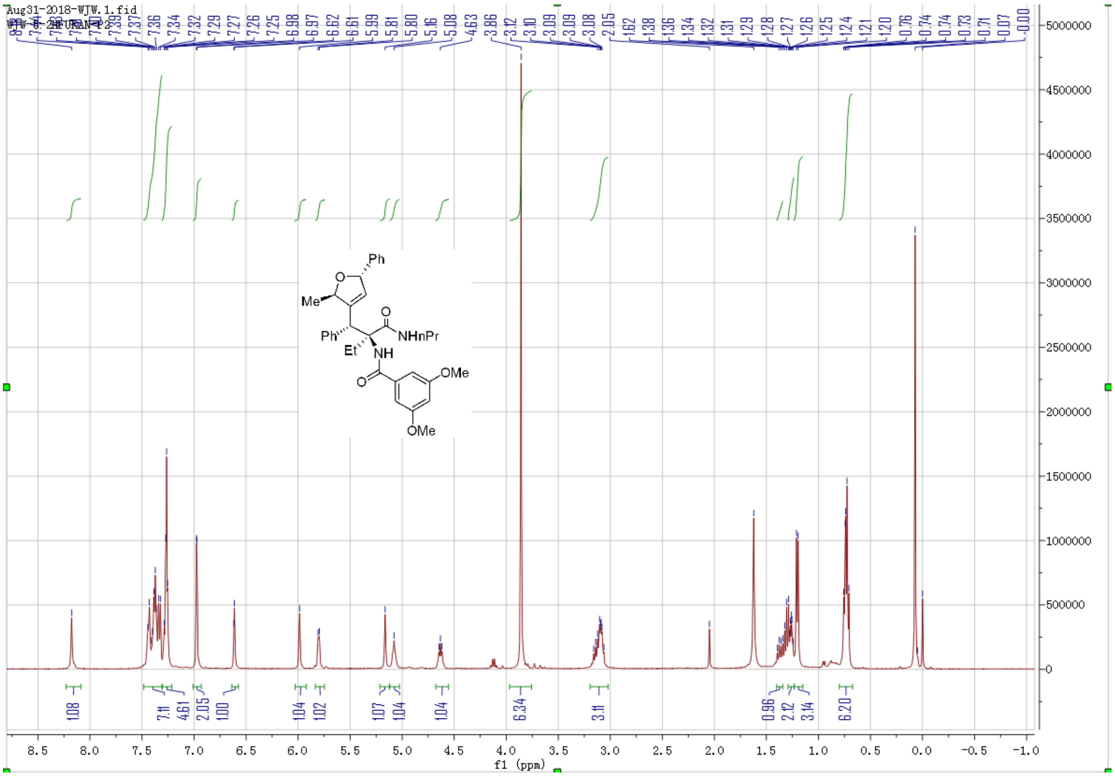

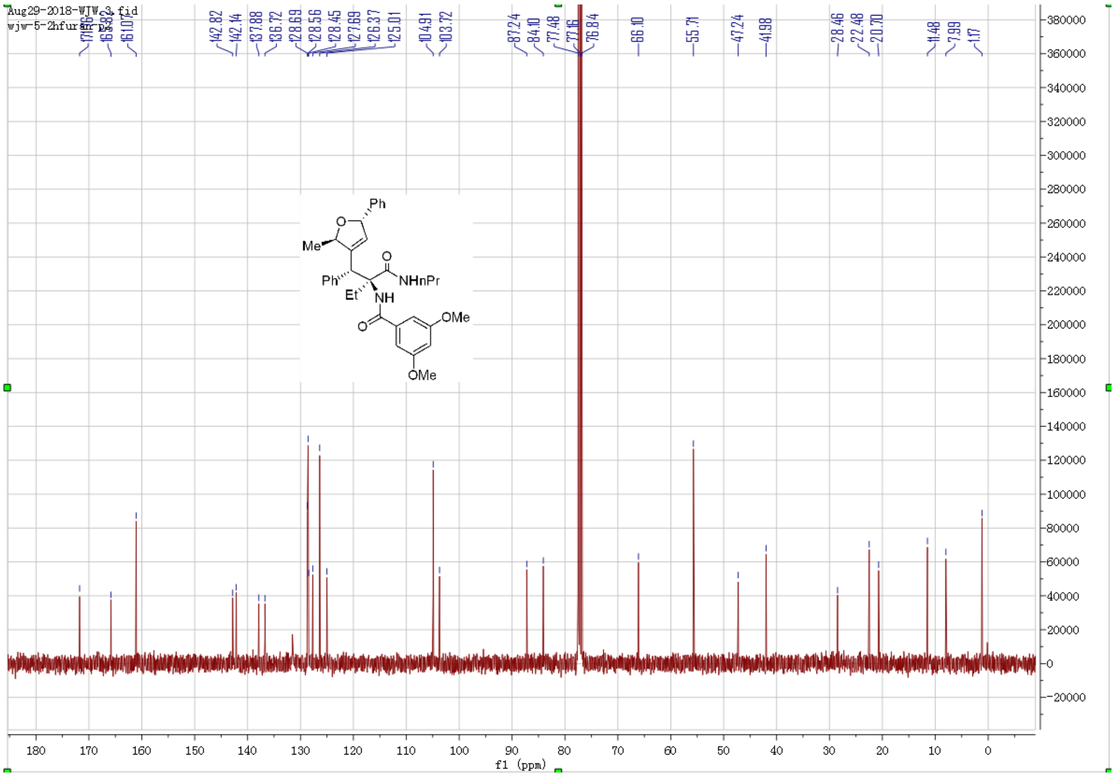

N-((*1S*,*4R*,*6R*,*10bR*)-1-ethyl-5-iodo-4-methyl-2-oxo-6-phenyl-1,4,6,10b-tetrahydro-2H-benzo[f]isochromen-1-yl)-3,5-dimethoxybenzamide (**12a**)

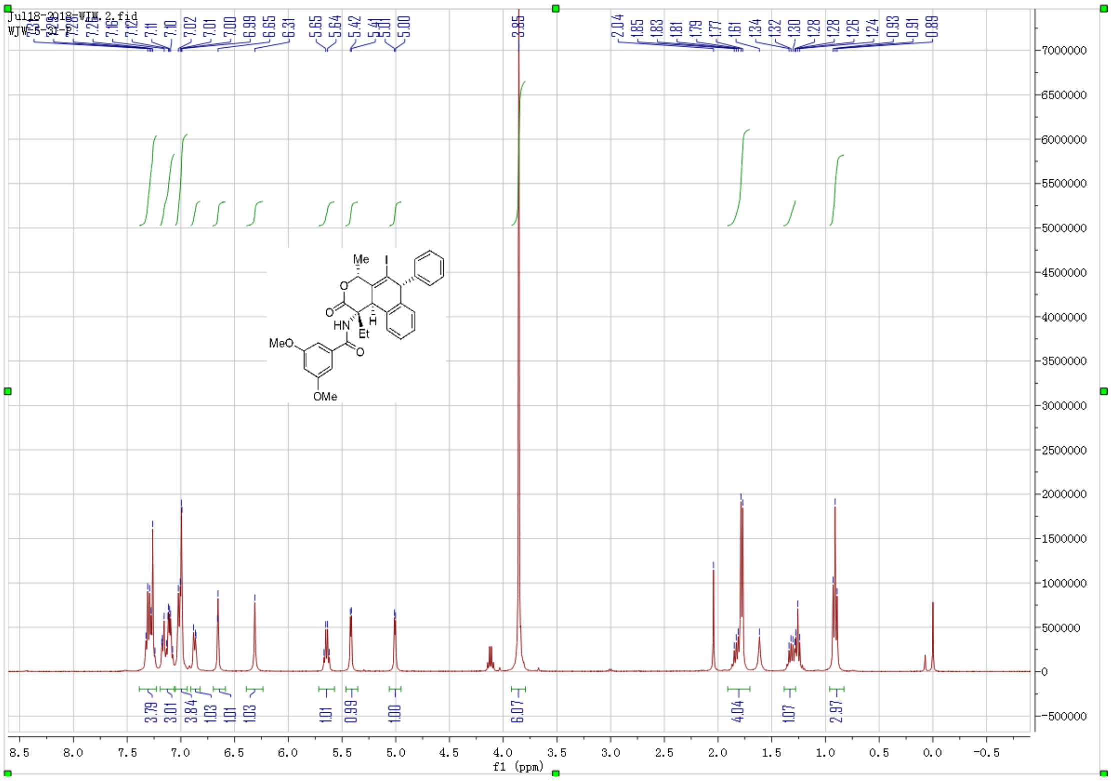

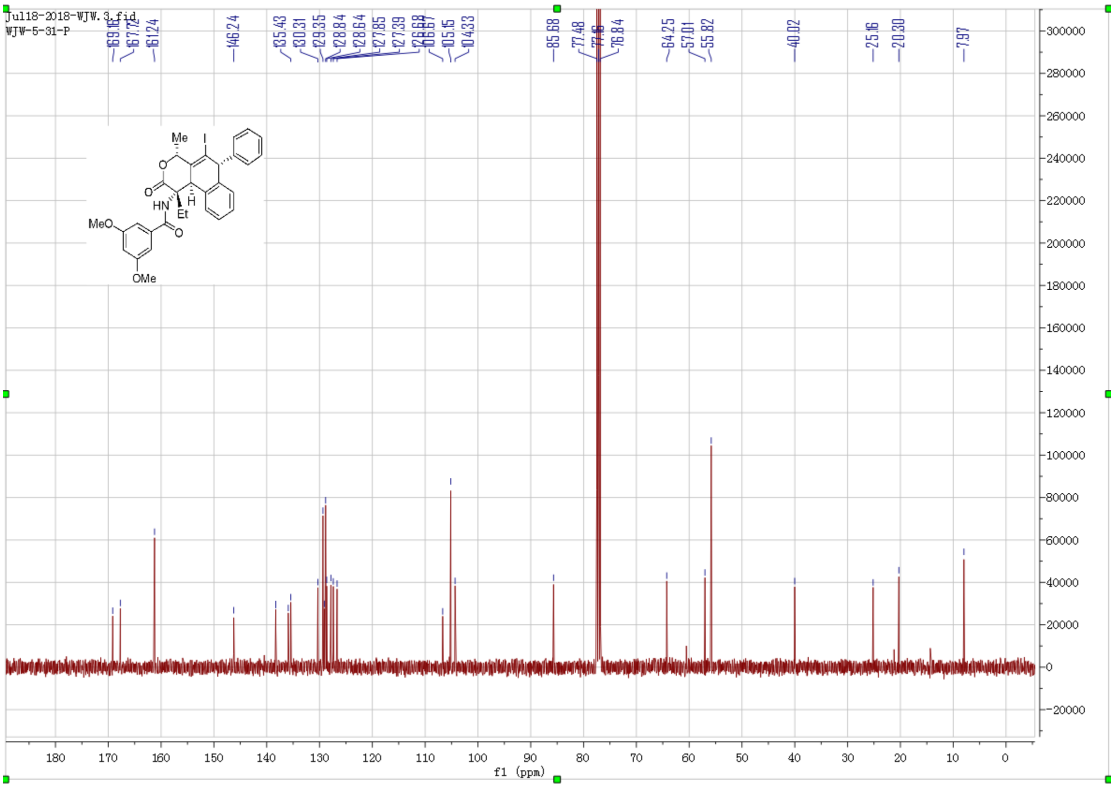

N-((3*S*,4*S*,6*S*)-3-ethyl-6-methyl-2-oxo-4-phenyl-5-((*S*)-2-phenylvinylidene)tetrahydro-2H-pyran-3-yl)-3,5-dimethoxybenzamide (**13a**)

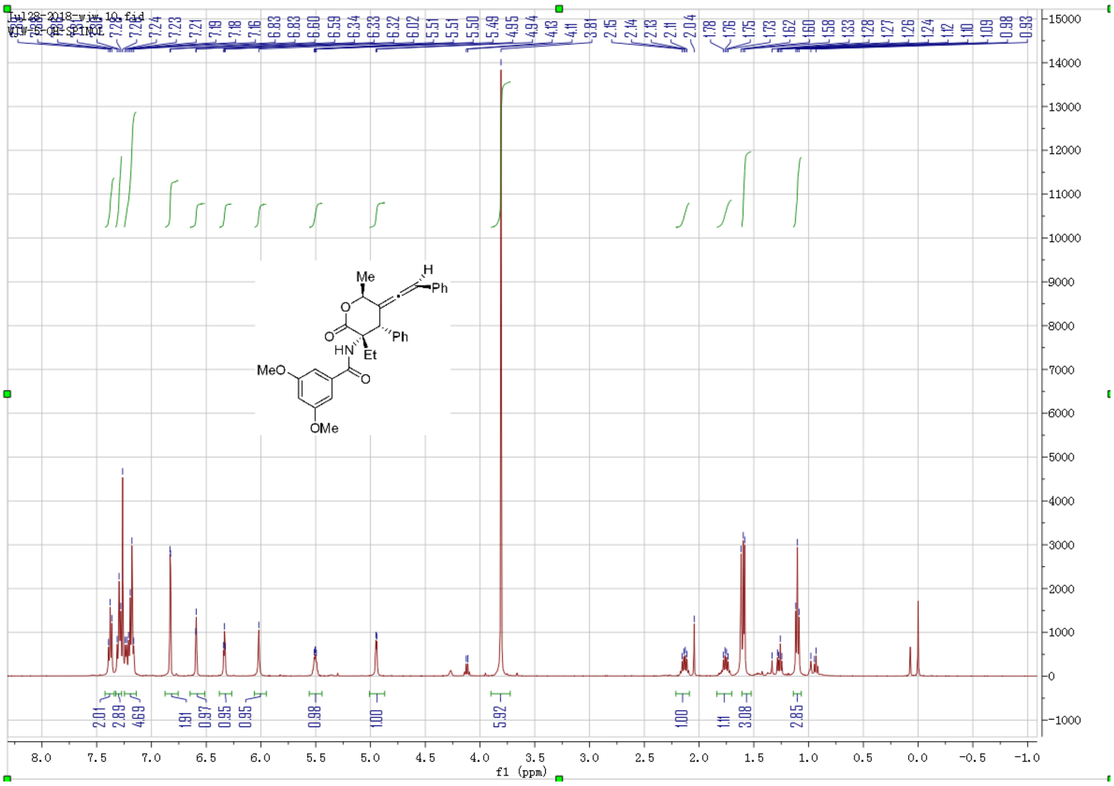

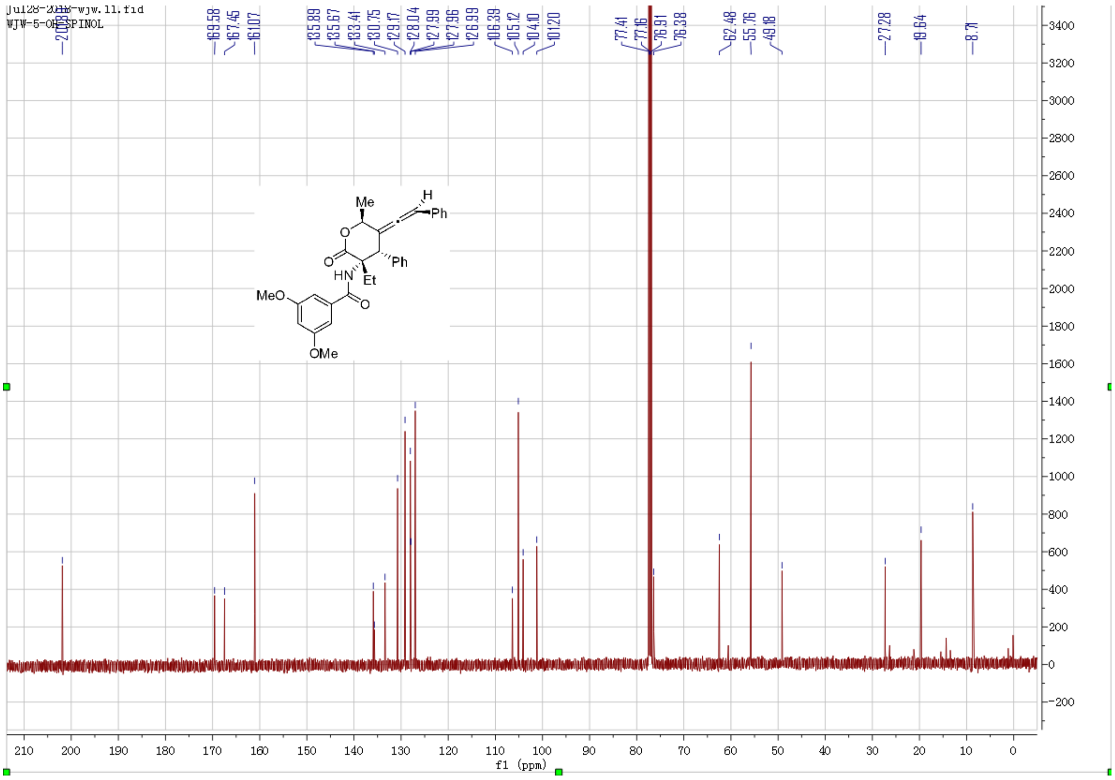

N-((3*S*,4*R*,6*S*)-5-((*S*)-1-hydroxyethyl)-4,7-diphenyl-3-(propylcarbamoyl)hepta-5,6-dien-3-yl)-3,5-dimethoxybenzamide (**14a**)

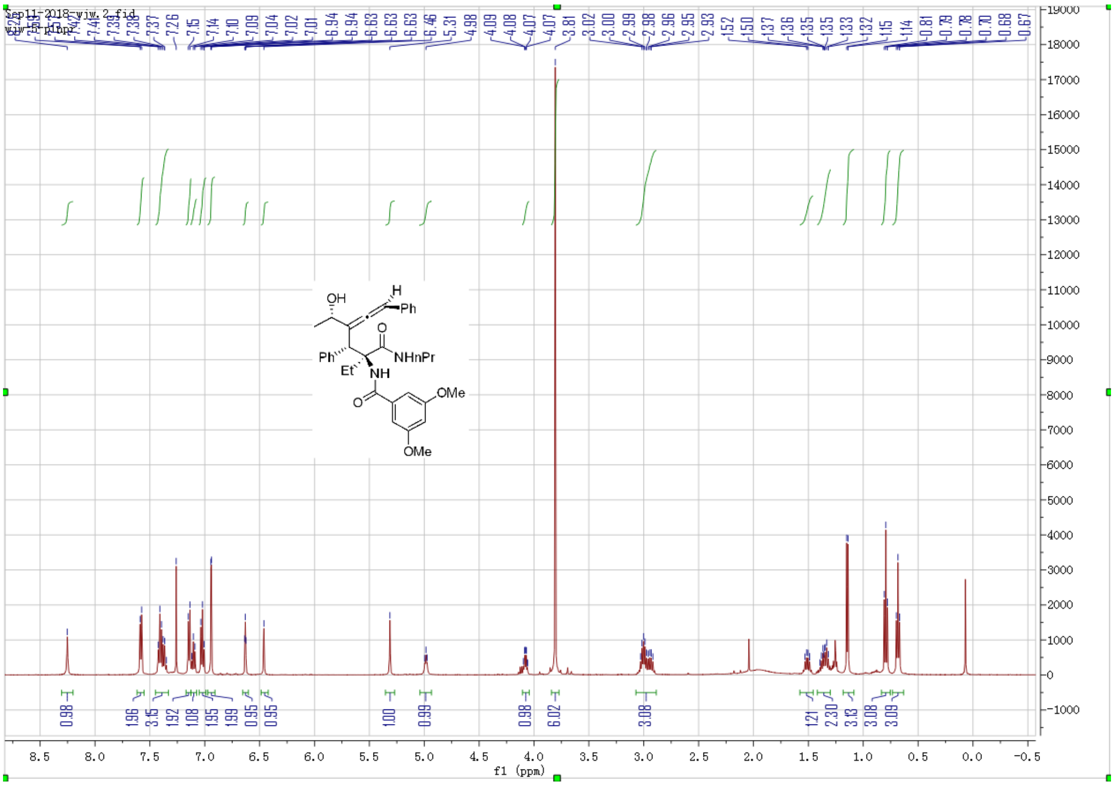

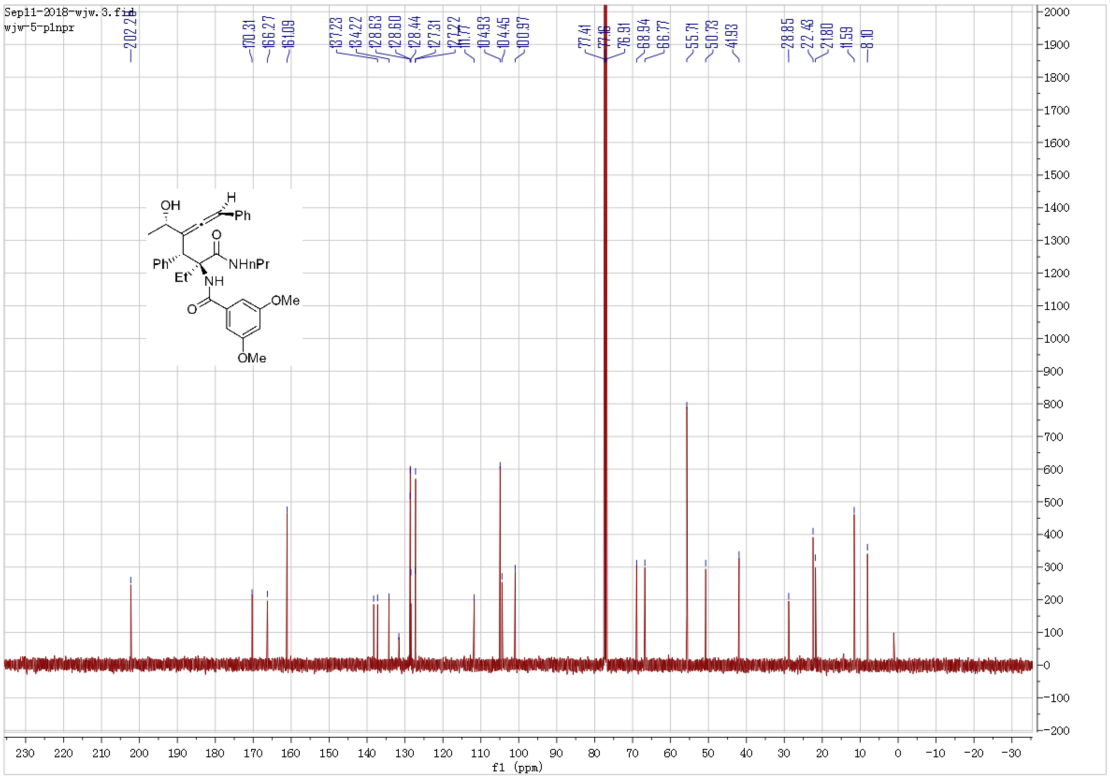

3,5-dimethoxy-*N*-((*1R*,*2S*)-1-((*2S*,*5R*)-2-methyl-5-phenyl-2,5-dihydrofuran-3-yl)-1-phenyl-2-(propylcarbamoyl)butan-2-yl)benzamide (**15a**)

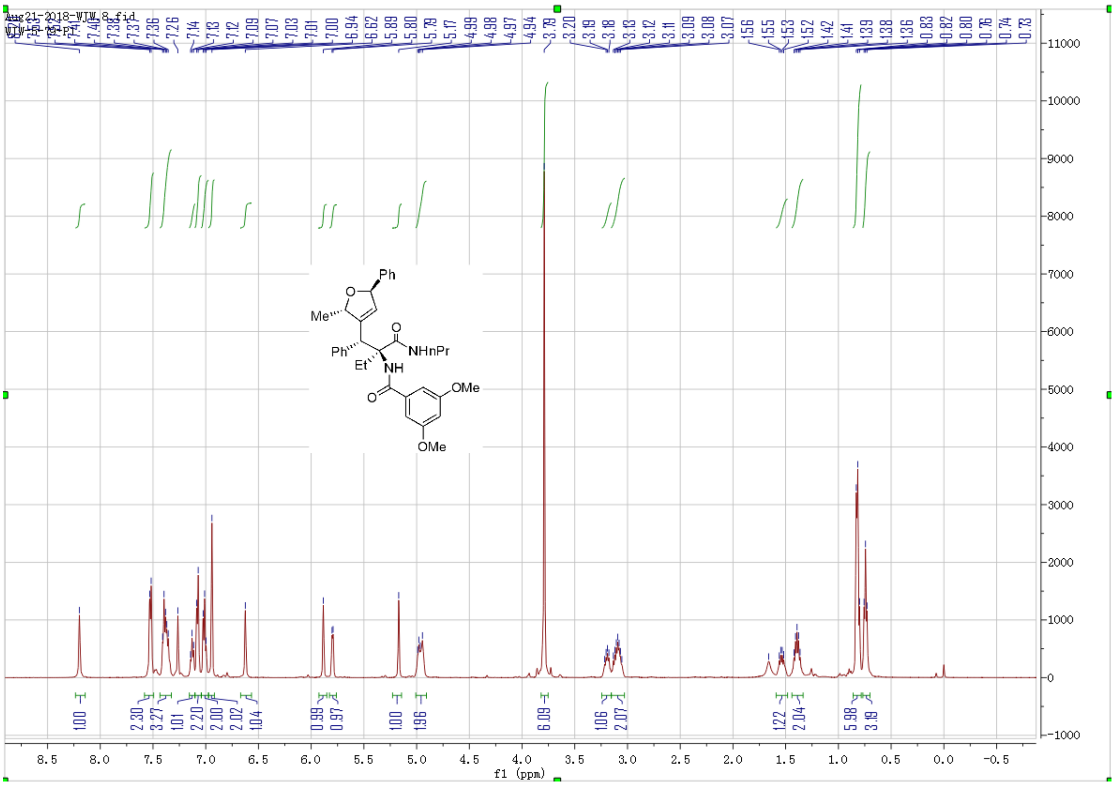

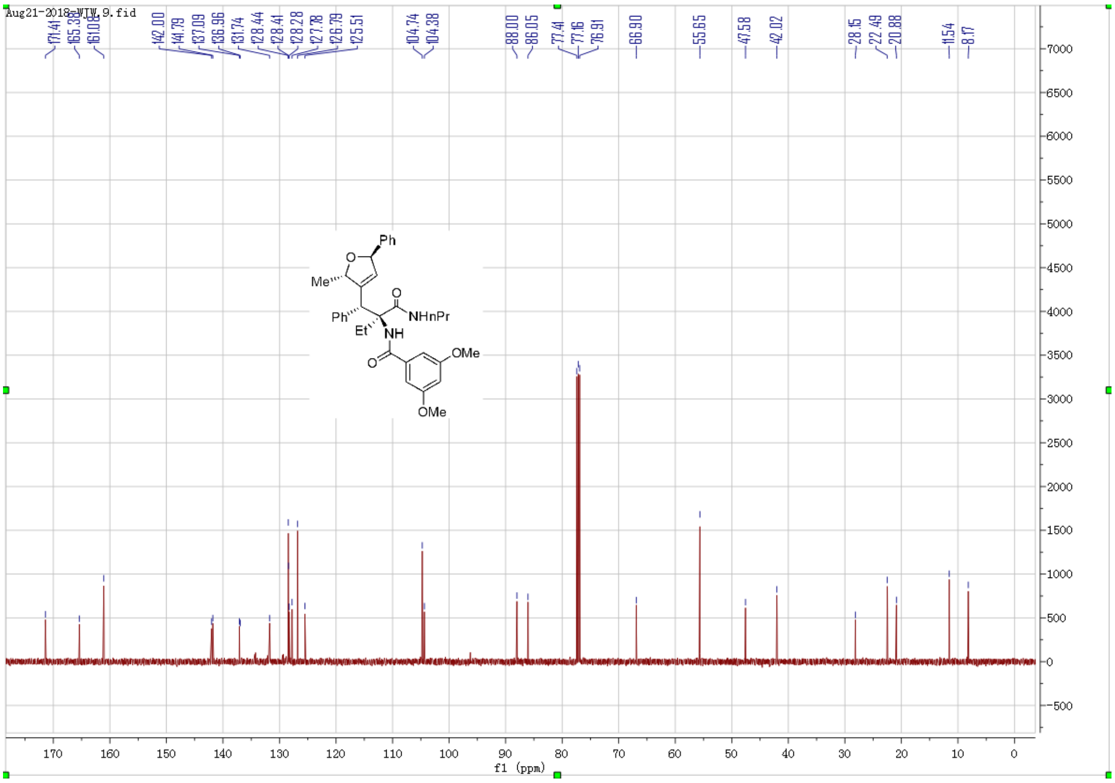

(*1R,5S,8S,9S*)-3-(3,5-dimethoxyphenyl)-5-ethyl-1-((*Z*)-1-iodo-2-phenylvinyl)-8-methyl-9-phenyl-2,7-dioxa-4-azabicyclo[3.3.1]non-3-en-6-one (**16a**)

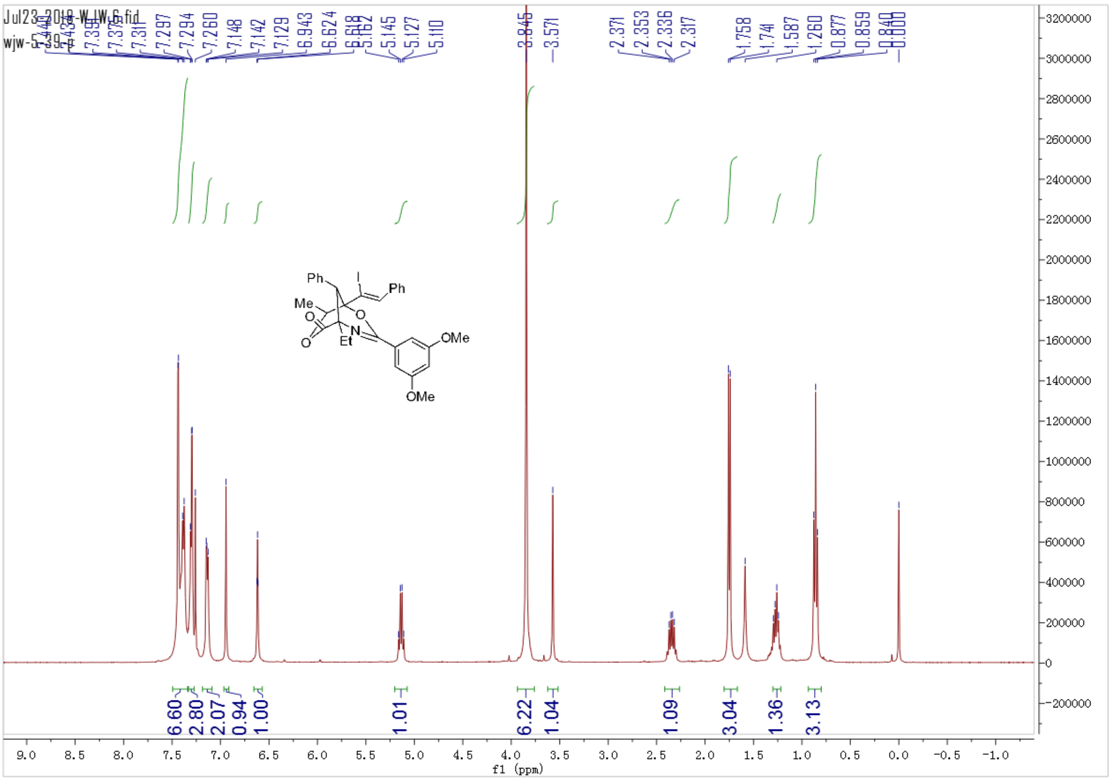

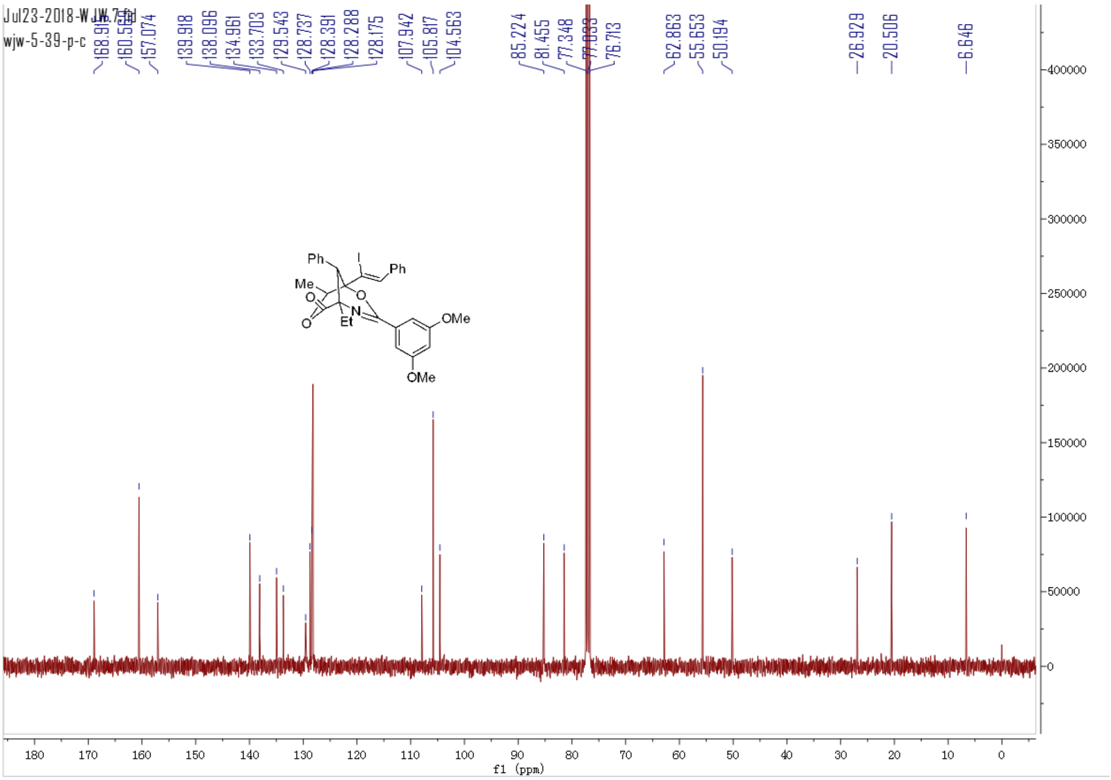

Supplementary References

1 Kashikura, W., Itoh, J., Mori, K. & Akiyama, T. Enantioselective Friedel–Crafts Alkylation of Indoles, Pyrroles, and Furans with Trifluoropyruvate Catalyzed by Chiral Phosphoric Acid. *Chem. Asian J.* **5**, 470-472 (2010).

2 Wang, T. & Zhang, J. Chemoselective C C Bond Cleavage of Epoxide Motifs: Gold(I)-Catalyzed Diastereoselective [4+3] Cycloadditions of 1-(1-Alkynyl)oxiranyl Ketones and Nitrones. *Chem. Eur. J.* **17**, 86-90 (2011).

3 Poulsen, P. H. *et al.* Organocatalytic Formation of Chiral Trisubstituted Allenes and Chiral Furan Derivatives. *Angew. Chem. Int. Ed.* **57**, 10661-10665 (2018).

4 Pathipati, S. R., van der Werf, A., Eriksson, L. & Selander, N. Diastereoselective Synthesis of Cyclopenta[c]furans by a Catalytic Multicomponent Reaction. *Angew. Chem. Int. Ed.* **55**, 11863-11866 (2016).

5 Du, Q., Neudörfl, J.-M. & Schmalz, H.-G. Chiral Phosphine–Phosphite Ligands in Asymmetric Gold Catalysis: Highly Enantioselective Synthesis of Furo[3,4-d]-Tetrahydropyridazine Derivatives through [3+3]-Cycloaddition. *Chem. Eur. J.* **24**, 2379-2383 (2018).

6 Wang, Z. *et al.* Enantioselective synthesis of chiral 4H-pyran derivatives through [3+3] tandem reaction over a squaramide catalyst. *Tetrahedron: Asymmetry* **28**, 1708-1716 (2017).

7 Hu, J., Liu, L., Yang, S. & Liang, Y.-M. Phase-transfer-catalyzed cyclization reaction of nucleophilic addition to electron-deficient 1,3-conjugated enynes for the synthesis of functionalized 4H-pyrans. *Org. Bio. Chem.* **9**, 3375-3379 (2011).

8 Zhang, H. *et al.* Catalytic Asymmetric Epoxidation of Electron-Deficient Enynes Promoted by Chiral N,N′-Dioxide-Scandium(III) Complex. *Adv. Synth. Catal.* **359**, 3454-3459 (2017).

9 Yao, T., Zhang, X. & Larock, R. C. AuCl3-Catalyzed Synthesis of Highly Substituted Furans from 2-(1-Alkynyl)-2-alken-1-ones. *J. Am. Chem. Soc.* **126**, 11164-11165 (2004).

10 de Mello, A. C., Momo, P. B., Burtoloso, A. C. B. & Amarante, G. W. Metal-Free Insertion Reactions of Diazo Carbonyls to Azlactones. *J. Org. Chem.* **83**, 11399-11406 (2018).

11 M. J. Frisch, G. W. Trucks, H. B. Schlegel, G. E. Scuseria, M. A. Robb, J. R. Cheeseman, G. Scalmani, V. Barone, B. Mennucci, G. A. Petersson, H. Nakatsuji, M. Caricato, X. Li, H. P. Hratchian, A. F. Izmaylov, J. Bloino, G. Zheng, J. L. Sonnenberg, M. Hada, M. Ehara, K. Toyota, R. Fukuda, J. Hasegawa, M. Ishida, T. Nakajima, Y. Honda, O. Kitao, H. Nakai, T. Vreven, Jr., J. A. Montgomery, J. E. Peralta, F. Ogliaro, M. Bearpark, J. J. Heyd, E. Brothers, K. N. Kudin, V. N. Staroverov, T. Keith, R. Kobayashi, J. Normand, K. Raghavachari, A. Rendell, J. C. Burant, S. S. Iyengar, J. Tomasi, M. Cossi, N. Rega, J. M. Millam, M. Klene, J. E. Knox, J. B. Cross, V. Bakken, C. Adamo, J. Jaramillo, R. Gomperts, R. E. Stratmann, O. Yazyev, A. J. Austin, R. Cammi, C. Pomelli, J. W. Ochterski, R. L. Martin, K. Morokuma, V. G. Zakrzewski, G. A. Voth, P. Salvador, J. J. Dannenberg, S. Dapprich, A. D. Daniels, O. Farkas, J. B. Foresman, J. V. Ortiz, J. Cioslowski, D. J. Fox, Gaussian 09, Rev. D.01; Gaussian, Inc., Wallingford CT, **2013**.

12 Schwabe, T. & Grimme, S. Double-hybrid density functionals with long-range dispersion corrections: higher accuracy and extended applicability. *Phys. Chem. Chem. Phys.* **9**, 3397-3406 (2007).

13 Schwabe, T. & Grimme, S. Theoretical Thermodynamics for Large Molecules: Walking the Thin Line between Accuracy and Computational Cost. *Acc. Chem. Res.* **41**, 569-579 (2008).

14 Marenich, A. V., Cramer, C. J. & Truhlar, D. G. Universal Solvation Model Based on Solute Electron Density and on a Continuum Model of the Solvent Defined by the Bulk Dielectric Constant and Atomic Surface Tensions. *J. Phys. Chem. B* **113**, 6378-6396 (2009).
15 Hratchian, H. P. & Schlegel, H. B. Accurate reaction paths using a Hessian based predictor–corrector integrator. *J. Chem. Phys.* **120**, 9918-9924 (2004).
16 Hratchian, H. P. & Schlegel, H. B. Using Hessian Updating To Increase the Efficiency of a Hessian Based Predictor-Corrector Reaction Path Following Method. *J. Chem. Theory Comput.* **1**, 61-69 (2005).
17 Peng, Q., Duarte, F. & Paton, R. S. Computing organic stereoselectivity – from concepts to quantitative calculations and predictions. *Chem. Soc. Rev.* **45**, 6093-6107 (2016).
18 Mitoraj, M. & Michalak, A. Donor–Acceptor Properties of Ligands from the Natural Orbitals for Chemical Valence. *Organometallics* **26**, 6576-6580 (2007).
19 Mitoraj, M. P., Michalak, A. & Ziegler, T. A Combined Charge and Energy Decomposition Scheme for Bond Analysis. *J. Chem. Theory Comput.* **5**, 962-975 (2009).
20 SCM, ADF2019, Theoretical Chemistry, Vrije Universiteit, Amsterdam, Netherlands, http://www.scm.com.
21 te Velde, G. *et al.* Chemistry with ADF. *J. Comput. Chem.* **22**, 931-967 (2001).
22 Van Lenthe, E. & Baerends, E. J. Optimized Slater-type basis sets for the elements 1–118. *J. Comput. Chem.* **24**, 1142-1156 (2003).
23 C. Y. Legault, CYLview, 1.0b; Universitéde Sherbrooke: Québec, Montreal, Canada, 2009; http://www.cylview.org.
24 Humphrey, W., Dalke, A. & Schulten, K. VMD: Visual molecular dynamics. *J. Mol. Graphics* **14**, 33-38 (1996).
25 Ess, D. H. & Houk, K. N. Distortion/Interaction Energy Control of 1,3-Dipolar Cycloaddition Reactivity. *J. Am. Chem. Soc.* **129**, 10646-10647 (2007).
26 Legault, C. Y., Garcia, Y., Merlic, C. A. & Houk, K. N. Origin of Regioselectivity in Palladium-Catalyzed Cross-Coupling Reactions of Polyhalogenated Heterocycles. *J. Am. Chem. Soc.* **129**, 12664-12665 (2007).
27 Ess, D. H. & Houk, K. N. Theory of 1,3-Dipolar Cycloadditions: Distortion/Interaction and Frontier Molecular Orbital Models. *J. Am. Chem. Soc.* **130**, 10187-10198 (2008).
28 Hayden, A. E. & Houk, K. N. Transition State Distortion Energies Correlate with Activation Energies of 1,4-Dihydrogenations and Diels−Alder Cycloadditions of Aromatic Molecules. *J. Am. Chem. Soc.* **131**, 4084-4089 (2009).
29 Schoenebeck, F., Ess, D. H., Jones, G. O. & Houk, K. N. Reactivity and Regioselectivity in 1,3-Dipolar Cycloadditions of Azides to Strained Alkynes and Alkenes: A Computational Study. *J. Am. Chem. Soc.* **131**, 8121-8133 (2009).
