# Supplementary material for: Chiral phosphoric acid-catalyzed stereodivergent synthesis of trisubstituted allenes and computational mechanistic studies
Source: Nat Commun. 2020 Nov 2;11:5527. doi: 10.1038/s41467-020-19294-8 (PMC7608664; doi:10.1038/s41467-020-19294-8)
Supplement: Supplementary file 3 — Description of Additional Supplementary Files [file 41467_2020_19294_MOESM3_ESM.pdf]

Description of Additional Supplementary Files

File Name: Supplementary Data 1
Description: The cartesian coordinates for the optimized molecule structures
