# Supplementary material for: Chiral phosphoric acid-catalyzed stereodivergent synthesis of trisubstituted allenes and computational mechanistic studies
Source: Nat Commun. 2020 Nov 2;11:5527. doi: 10.1038/s41467-020-19294-8 (PMC7608664; doi:10.1038/s41467-020-19294-8)
Supplement: Supplementary file 4 — Supplementary Data 1 [file 41467_2020_19294_MOESM4_ESM.zip › 256936_2_data_set_4967612_qhgj1p (3).docx]

### Supplementary Data 1. Cartesian Coordinates of the Optimized Structures

| **INT0-C**  C 2.7781271 2.0283229 -0.3236535  O 3.5293191 2.7331339 -1.2215945  C 2.6355011 3.2693349 -2.1486095  C 1.2503481 2.8570559 -1.6886415  H 0.7887031 2.2010089 -2.4373965  C 3.4990351 1.2887239 0.7173835  C 2.8266341 0.2714779 1.3891045  C 4.8354971 1.5849689 0.9748025  C 3.5212611 -0.4657901 2.3445605  H 1.8027471 0.0034109 1.1481385  C 5.5095571 0.8458769 1.9438605  H 5.3592011 2.3666419 0.4376625  C 4.8602991 -0.1815281 2.6365765  H 5.3910301 -0.7581921 3.3816165  O 3.0109131 3.9126189 -3.0782655  N 1.5160791 2.0692719 -0.4922755  C 0.3057821 4.0326779 -1.4146685  C -0.1482649 4.7090269 -2.7038375  H 0.7990751 4.7454539 -0.7429535  H -0.5563279 3.6206099 -0.8791005  H -0.8104239 5.5498459 -2.4816495  H 0.7026141 5.0828059 -3.2800295  H -0.7012809 3.9965809 -3.3238795  O 6.8072541 1.1868219 2.1487965  O 2.8260471 -1.4693811 2.9341755  C 7.5427991 0.4522019 3.1014095  H 8.5474901 0.8743399 3.0973305  H 7.1102531 0.5528659 4.1043275  H 7.5940571 -0.6105151 2.8333085  C 3.5139661 -2.3114921 3.8300715  H 3.8736021 -1.7590121 4.7069295  H 2.7933351 -3.0635541 4.1511825  H 4.3628621 -2.8050401 3.3392865  H 0.2008441 1.5330429 0.3835145  O -0.6899799 1.1168609 0.6348585  P -1.2202569 0.3434889 -0.6237105  O -2.7689089 0.0201649 -0.3158825  O -0.4799589 -1.0973731 -0.4328665  O -1.0623969 0.9575559 -1.9506585  C -3.1048809 -0.5934951 0.8841115  C -0.7938429 -2.0136721 -1.4294985  C -3.0636649 -1.9769991 0.9737615  C -3.4572959 0.2009809 1.9841185  C -1.9230529 -2.8042671 -1.2908835  C -0.0076769 -2.0595161 -2.5867345  C -2.8245159 -3.0320221 -0.0926775  C -3.2988169 -2.5861081 2.2104035  C -3.7281029 -0.4438931 3.1954565  C -3.4834639 1.6868709 1.9040425  C -2.3467939 -3.5847501 -2.3709665  C -0.4275189 -2.8878791 -3.6311295  C 1.2259841 -1.2352771 -2.7208615  C -2.3030029 -4.2042761 0.7879635  C -4.1378719 -3.4540091 -0.8097315  C -3.6349589 -1.8277461 3.3230055  C -3.0890779 -4.0791721 2.1052295  H -4.0011309 0.1664029 4.0513905  C -4.3328159 2.3796729 0.9835775  C -2.6722389 2.4059059 2.7532355  C -1.6044409 -3.6309851 -3.5430105  C -3.6659659 -4.2485851 -2.0434355  H 0.1748571 -2.9225021 -4.5342495  C 2.3722201 -1.4854051 -1.8977785  C 1.2796891 -0.2550461 -3.6848065  H -1.2360289 -4.0447001 0.9805335  H -2.4195339 -5.1752951 0.2969195  H -4.6640319 -2.5489651 -1.1333965  H -4.8051729 -4.0207211 -0.1528025  H -3.8151629 -2.3026351 4.2832925  H -2.5486299 -4.4887001 2.9638485  H -4.0500949 -4.6064671 2.0463335  C -5.2370209 1.7098489 0.1158575  C -4.2915809 3.8030209 0.9496595  C -2.6388719 3.8173649 2.7196735  H -2.0164789 1.8672199 3.4313815  H -1.9352729 -4.2308841 -4.3860825  H -4.3753249 -4.1994071 -2.8747965  H -3.5229729 -5.3098951 -1.8033995  C 2.3913501 -2.4874671 -0.8898395  C 3.5551941 -0.7212431 -2.1158265  C 2.4487831 0.5174689 -3.8815345  H 0.3919061 -0.0549831 -4.2772495  C -6.0381279 2.4118579 -0.7470085  H -5.2922849 0.6263629 0.1450425  C -5.1295709 4.5011589 0.0402175  C -3.4255639 4.5008509 1.8304745  H -1.9779259 4.3532249 3.3940105  C 3.5239361 -2.7241771 -0.1530925  H 1.4947981 -3.0732641 -0.7121315  C 4.7122771 -0.9920541 -1.3370325  C 3.5622471 0.2889509 -3.1149265  H 2.4572811 1.2982009 -4.6372615  C -5.9819149 3.8251769 -0.7919155  H -6.7215009 1.8807229 -1.4024355  H -5.0826539 5.5871489 0.0223795  H -3.4034059 5.5869009 1.7881445  C 4.7022861 -1.9748671 -0.3822105  H 3.5157091 -3.4932961 0.6142725  H 5.6066701 -0.4000261 -1.5152575  H 4.4640991 0.8796499 -3.2574985  H -6.6182339 4.3692419 -1.4831825  H 5.5927731 -2.1738611 0.2070815 | **TS1-C**  C 2.6214079 2.2229546 -0.4960012  O 2.9225579 2.6964596 -1.6904432  C 1.6824859 3.1751866 -2.3098332  C 0.6390659 2.8471326 -1.3633732  H -0.0530481 1.6095996 -1.6922602  C 3.6141709 1.5731326 0.3539848  C 3.1779859 0.6836536 1.3350308  C 4.9638419 1.8506316 0.1519688  C 4.1318339 0.0760896 2.1486948  H 2.1335789 0.4055506 1.4523388  C 5.9002009 1.2382786 0.9833578  H 5.2942529 2.5291836 -0.6259742  C 5.4935679 0.3586586 1.9914188  H 6.2269089 -0.1201914 2.6254018  O 1.7354049 3.6705786 -3.3951412  N 1.3634289 2.3827526 -0.2192902  C -0.5438071 3.7667836 -1.1103742  C -1.4223741 3.9076736 -2.3515292  H -0.2010521 4.7521836 -0.7673862  H -1.1274981 3.3293896 -0.2889752  H -2.2459091 4.5992876 -2.1554272  H -0.8411461 4.2814646 -3.1991412  H -1.8524481 2.9390106 -2.6245062  O 7.1962929 1.5483716 0.7351558  O 3.6508009 -0.7975224 3.0619838  C 8.1886089 0.9402636 1.5329958  H 9.1421709 1.3213796 1.1690548  H 8.0690069 1.2066726 2.5901168  H 8.1688919 -0.1516964 1.4284898  C 4.5739139 -1.5094184 3.8544968  H 5.1685909 -0.8353114 4.4830418  H 3.9795339 -2.1643534 4.4909138  H 5.2447679 -2.1149804 3.2326328  H 0.8509719 1.9567026 0.5721338  O -0.5072101 0.8993396 0.9440898  P -1.0206711 0.1386906 -0.2277902  O -2.6321681 -0.0372754 -0.2561702  O -0.4812051 -1.3878844 -0.0860612  O -0.7238021 0.6548756 -1.6467112  C -3.1887091 -0.5298054 0.9156078  C -0.8401291 -2.2618304 -1.1029202  C -3.2993031 -1.9008504 1.0831518  C -3.5659011 0.3661116 1.9242558  C -2.0444731 -2.9446594 -1.0238732  C 0.0013359 -2.3890354 -2.2152702  C -3.0589941 -3.0341504 0.1026658  C -3.7175681 -2.4060234 2.3174838  C -4.0288901 -0.1697964 3.1305528  C -3.4150881 1.8374776 1.7528718  C -2.4605641 -3.7170354 -2.1133702  C -0.4226571 -3.2030734 -3.2692192  C 1.2886389 -1.6433754 -2.3057702  C -2.7374731 -4.1910384 1.0910358  C -4.3431201 -3.3729144 -0.7074362  C -4.0894361 -1.5464604 3.3422318  C -3.6343471 -3.9159204 2.3137148  H -4.3238391 0.5167796 3.9189818  C -4.0772151 2.5481926 0.6997848  C -2.6219831 2.5279356 2.6420778  C -1.6544821 -3.8546824 -3.2347372  C -3.8484431 -4.2622104 -1.8630422  H 0.2238069 -3.2996904 -4.1367782  C 2.3821239 -1.9365254 -1.4303772  C 1.4326979 -0.6727104 -3.2722312  H -1.6840341 -4.1155904 1.3834748  H -2.8973011 -5.1764974 0.6423468  H -4.7540611 -2.4410614 -1.1117302  H -5.1125031 -3.8447794 -0.0882482  H -4.4145361 -1.9367914 4.3025238  H -3.2180481 -4.3146434 3.2434678  H -4.6281631 -4.3637394 2.1864038  C -4.9378541 1.9140966 -0.2371452  C -3.8868371 3.9571106 0.5957038  C -2.4369101 3.9241276 2.5344878  H -2.0986601 1.9739596 3.4156348  H -1.9815541 -4.4495314 -4.0828562  H -4.4882531 -4.2039004 -2.7484082  H -3.8071551 -5.3171394 -1.5623692  C 2.3042059 -2.9159404 -0.4030992  C 3.6092749 -1.2332054 -1.6050742  C 2.6352059 0.0576106 -3.4089862  H 0.5848039 -0.4386604 -3.9088972  C -5.5575651 2.6329666 -1.2262512  H -5.1063541 0.8448626 -0.1590862  C -4.5450511 4.6737036 -0.4397972  C -3.0531621 4.6238336 1.5306568  H -1.7963301 4.4344786 3.2472168  C 3.3881529 -3.1930364 0.3903888  H 1.3714039 -3.4510594 -0.2552462  C 4.7176739 -1.5535774 -0.7780202  C 3.7028599 -0.2219194 -2.5954002  H 2.7009879 0.8506506 -4.1484812  C -5.3584121 4.0297816 -1.3343732  H -6.2093591 2.1283606 -1.9327862  H -4.3887891 5.7479316 -0.5054962  H -2.9142901 5.6977976 1.4327158  C 4.6143829 -2.5152184 0.1923118  H 3.3079339 -3.9436034 1.1711608  H 5.6501919 -1.0146634 -0.9303952  H 4.6315489 0.3368986 -2.6856352  H -5.8551171 4.5858846 -2.1234612  H 5.4737849 -2.7575074 0.8124408 |
| --- | --- |
| **INT1-C**  C 2.7636987 2.1166066 -0.5898121  O 3.1135897 2.5088666 -1.7978401  C 1.9264987 3.0517856 -2.4755151  C 0.8858907 2.8898326 -1.5262301  H -0.0664113 1.2822066 -1.8214281  C 3.6762957 1.4338256 0.3173259  C 3.1544587 0.6305416 1.3320699  C 5.0499817 1.5916086 0.1408099  C 4.0391287 -0.0105214 2.1952919  H 2.0894147 0.4421646 1.4399989  C 5.9165537 0.9482436 1.0219399  H 5.4503467 2.2074616 -0.6563741  C 5.4227427 0.1507036 2.0592619  H 6.1007607 -0.3529774 2.7341409  O 2.0397627 3.4664776 -3.6019331  N 1.5018227 2.3816606 -0.3714781  C -0.3989483 3.6715406 -1.4457101  C -1.1168093 3.7353256 -2.7943821  H -0.2046793 4.6917616 -1.0830991  H -1.0543753 3.2058436 -0.6964391  H -2.0145353 4.3537576 -2.7124991  H -0.4597933 4.1546766 -3.5601981  H -1.4229613 2.7350126 -3.1159911  O 7.2397487 1.1454936 0.7935009  O 3.4692327 -0.7999034 3.1372649  C 8.1616277 0.4919196 1.6370449  H 9.1509757 0.7795256 1.2821909  H 8.0432187 0.8091576 2.6802929  H 8.0547597 -0.5983804 1.5747709  C 4.3175227 -1.5497024 3.9755939  H 4.9433707 -0.9002854 4.6000799  H 3.6633257 -2.1423804 4.6146739  H 4.9602667 -2.2199034 3.3911589  H 0.9616407 2.0659196 0.4418499  O -0.4060573 0.9194226 0.8731629  P -1.0357273 0.1408586 -0.2145281  O -2.6452753 0.1005576 -0.2267191  O -0.6084763 -1.4079874 -0.0798491  O -0.7853663 0.5857466 -1.6981271  C -3.2364213 -0.3595364 0.9481069  C -1.0335293 -2.2619374 -1.0969441  C -3.4656143 -1.7186584 1.0926909  C -3.5208533 0.5514536 1.9743989  C -2.2882173 -2.8455084 -1.0190441  C -0.1991463 -2.4509374 -2.2055911  C -3.3132263 -2.8587684 0.1018279  C -3.9279123 -2.2030454 2.3201239  C -4.0365543 0.0381646 3.1697879  C -3.1915443 1.9983676 1.8521449  C -2.7583483 -3.5839134 -2.1104841  C -0.6810803 -3.2329864 -3.2587291  C 1.1293117 -1.7825004 -2.2968571  C -3.0881463 -4.0467664 1.0802329  C -4.6160443 -3.0909434 -0.7163871  C -4.2265023 -1.3295174 3.3567979  C -3.9668843 -3.7140644 2.3012379  H -4.2627613 0.7351636 3.9715249  C -3.7059713 2.8157156 0.7948909  C -2.3459023 2.5531076 2.7873439  C -1.9600673 -3.7866144 -3.2271801  C -4.1866223 -4.0161724 -1.8694531  H -0.0414653 -3.3806174 -4.1240941  C 2.1916767 -2.1020374 -1.3930311  C 1.3326657 -0.8399234 -3.2815701  H -2.0337173 -4.0576974 1.3781669  H -3.3222323 -5.0118004 0.6203399  H -4.9490793 -2.1288924 -1.1215371  H -5.4242593 -3.5004014 -0.1024141  H -4.5869583 -1.7047994 4.3103269  H -3.5872433 -4.1525124 3.2287429  H -4.9924103 -4.0806354 2.1664749  C -4.6164423 2.3312296 -0.1827711  C -3.3018123 4.1806346 0.7235539  C -1.9532663 3.9076106 2.7156429  H -1.9366093 1.9169126 3.5666739  H -2.3295173 -4.3555694 -4.0756991  H -4.8132573 -3.9051464 -2.7591751  H -4.2321313 -5.0713474 -1.5707671  C 2.0560437 -3.0599594 -0.3518691  C 3.4466057 -1.4459484 -1.5522951  C 2.5622107 -0.1530234 -3.4027291  H 0.5095217 -0.5894024 -3.9450901  C -5.0779233 3.1460446 -1.1842951  H -4.9501603 1.3000276 -0.1277171  C -3.7924423 4.9978796 -0.3293341  C -2.4148153 4.7027336 1.7002959  H -1.2725183 4.3067956 3.4610129  C 3.1099187 -3.3569954 0.4740209  H 1.1032447 -3.5629424 -0.2186461  C 4.5223687 -1.7856254 -0.6906691  C 3.5985387 -0.4568464 -2.5576221  H 2.6701987 0.6320326 -4.1456631  C -4.6582693 4.4951596 -1.2649721  H -5.7716813 2.7549236 -1.9218801  H -3.4667493 6.0342606 -0.3750611  H -2.1103203 5.7436546 1.6252969  C 4.3617227 -2.7210344 0.2971489  H 2.9862987 -4.0914944 1.2642379  H 5.4751497 -1.2800464 -0.8284471  H 4.5449097 0.0736036 -2.6302811  H -5.0259993 5.1283536 -2.0666601  H 5.1950197 -2.9737194 0.9476429 | **INT2-C**  C -2.0539916 2.3957960 -0.6771138  O -3.3101706 2.8046040 -0.7477998  C -4.0204066 2.3636730 0.4380172  C -3.0459686 1.6760330 1.1779172  N -1.8758176 1.7344320 0.4474952  C -1.0930256 2.6631300 -1.7311528  C 0.2100934 2.1664320 -1.6414198  C -1.5077106 3.3855320 -2.8525778  C 1.0837284 2.3663540 -2.7027788  H 0.5500644 1.5675890 -0.8028878  C -0.6103886 3.5899780 -3.8962478  H -2.5192656 3.7658870 -2.9325158  C 0.6939394 3.0838420 -3.8390048  H 1.3838074 3.2446780 -4.6554928  C -3.1059476 1.0875060 2.5494982  C -4.5058986 1.1651690 3.1574512  H -2.3909586 1.6088940 3.2023542  H -2.7740886 0.0395900 2.5239332  H -4.4975146 0.7509280 4.1701942  H -4.8582476 2.1982540 3.2020462  H -5.2250686 0.6033730 2.5556892  O -5.2030896 2.6407750 0.5579812  C -2.3058766 -2.5931850 2.1011172  O -1.1284416 -2.7164610 2.4213402  C -3.3803286 -3.1112930 3.0217202  H -2.9163216 -3.6362890 3.8562222  H -3.9777666 -2.2702160 3.3910122  H -4.0676726 -3.7678490 2.4797272  C -2.7367466 -1.9320090 0.8365022  C -1.7931456 -1.6742810 -0.1074648  C -1.9056646 -1.0091750 -1.3982888  C -0.7576286 -0.9916860 -2.2054078  C -3.0698356 -0.3674780 -1.8654578  C -0.7767736 -0.3968180 -3.4615888  H 0.1597754 -1.4413380 -1.8326748  C -3.0762446 0.2425490 -3.1109498  H -3.9566316 -0.3233170 -1.2445408  C -1.9361906 0.2206490 -3.9184798  H 0.1241774 -0.3999060 -4.0688248  H -3.9718336 0.7556310 -3.4477268  H -1.9505116 0.7092120 -4.8887408  H -0.7946416 -2.0423590 0.1258842  C -4.1299676 -1.6326330 0.7289312  C -5.3256526 -1.4263300 0.7194192  C -6.7376546 -1.1780610 0.6816502  C -7.2206046 0.1285570 0.5142282  C -7.6367856 -2.2464870 0.8139332  C -8.5939966 0.3474680 0.4781812  H -6.5242746 0.9616350 0.4275992  C -9.0050826 -2.0124480 0.7758232  H -7.2499866 -3.2527990 0.9422082  C -9.4856476 -0.7147910 0.6065802  H -8.9659986 1.3593060 0.3514322  H -9.6980356 -2.8421050 0.8770962  H -10.5558716 -0.5332090 0.5769072  O -1.0932916 4.2910050 -4.9572418  O 2.3081264 1.7913170 -2.5581258  C -0.2346976 4.5062280 -6.0525738  H 0.0912984 3.5581000 -6.4989758  H -0.8136806 5.0693560 -6.7846468  H 0.6479564 5.0896520 -5.7622468  C 3.2165504 1.9081320 -3.6241488  H 4.0882524 1.3160950 -3.3446998  H 2.7960024 1.5100140 -4.5567478  H 3.5212654 2.9513820 -3.7834128  H 0.3945564 -2.1161020 2.2282402  O 0.4070134 0.1763510 0.8118862  P 1.5773404 -0.6317240 1.2010972  O 2.7775194 0.1581060 1.9194612  O 2.2552844 -1.2705800 -0.1252478  O 1.3404154 -1.7927000 2.2219502  C 3.3377814 1.1997770 1.1732132  C 3.2814074 -2.1910460 0.1003172  C 4.4047954 0.9094590 0.3386622  C 2.8036034 2.4938910 1.2575082  C 4.5735124 -1.7465620 0.3315842  C 2.9598434 -3.5535120 0.1620982  C 5.1980864 -0.3719940 0.1779262  C 4.9238504 1.9098060 -0.4876388  C 3.3567634 3.4752180 0.4243772  C 1.6704454 2.8440070 2.1576892  C 5.5429724 -2.6650620 0.7520322  C 3.9625304 -4.4531880 0.5295842  C 1.5823734 -4.0321760 -0.1364608  C 5.7710824 -0.1659290 -1.2547668  C 6.3633404 -0.4797000 1.2006962  C 4.3986024 3.1934070 -0.4550068  C 6.0362194 1.3471370 -1.3420728  H 2.9476884 4.4796170 0.4796812  C 1.7196004 2.5968370 3.5691942  C 0.5664494 3.4667530 1.6162472  C 5.2460804 -4.0159540 0.8536662  C 6.8270394 -1.9449280 1.0971672  H 3.7129444 -5.5088260 0.5828522  C 1.0539704 -3.9437470 -1.4659098  C 0.8230074 -4.5948220 0.8627852  H 4.9972064 -0.4493930 -1.9789328  H 6.6563574 -0.7811520 -1.4426808  H 5.9639064 -0.2894480 2.2032362  H 7.1576124 0.2473910 1.0064372  H 4.7922864 3.9715990 -1.1032428  H 6.0289684 1.7322210 -2.3662688  H 7.0164224 1.5985990 -0.9165098  C 2.8518684 2.0364560 4.2198932  C 0.5980874 2.9659910 4.3675862  C -0.5412126 3.8343590 2.4137002  H 0.5382184 3.6534940 0.5452022  H 5.9977474 -4.7248900 1.1889332  H 7.2731694 -2.3147350 2.0249022  H 7.5761094 -2.0717690 0.3052872  C 1.8009814 -3.4157460 -2.5535258  C -0.2672506 -4.4135600 -1.7121778  C -0.4852786 -5.0714620 0.6097132  H 1.2287994 -4.6444810 1.8695802  C 2.8573034 1.8271040 5.5750022  H 3.7279324 1.7800050 3.6338002  C 0.6307794 2.7275570 5.7668652  C -0.5276666 3.5813740 3.7597352  H -1.4059396 4.2981010 1.9474092  C 1.2565084 -3.3463620 -3.8116338  H 2.8152134 -3.0695640 -2.3784888  C -0.8048376 -4.3162400 -3.0219288  C -1.0223346 -4.9722430 -0.6455088  H -1.0617986 -5.4950380 1.4264282  C 1.7309474 2.1673450 6.3602472  H 3.7336144 1.3977330 6.0506952  H -0.2370776 3.0065250 6.3588372  H -1.3765176 3.8516390 4.3830432  C -0.0633256 -3.7949100 -4.0498378  H 1.8421854 -2.9426950 -4.6324748  H -1.8214816 -4.6608170 -3.1920518  H -2.0328606 -5.3175240 -0.8484998  H 1.7458994 1.9907020 7.4312612  H -0.4882306 -3.7175030 -5.0456928  H -1.0024296 1.2633060 0.7107092 |
| **1a**  C -0.2367658 3.1625913 1.1315990  O 0.8187022 3.3243793 1.7020490  C -1.2775288 4.2656063 1.0953350  H -0.8744418 5.1225043 1.6346500  H -1.5076618 4.5583523 0.0665020  H -2.2092548 3.9439503 1.5708280  C -0.5310758 1.8543313 0.4411460  C 0.4986302 0.8752203 0.5575610  C 1.3867672 0.0633953 0.6982340  C 2.4054152 -0.9309327 0.8334810  C 2.1316592 -2.2633497 0.4911670  C 3.6803512 -0.5862207 1.3037000  C 3.1196162 -3.2315957 0.6159620  H 1.1387412 -2.5240837 0.1368010  C 4.6625582 -1.5611587 1.4251220  H 3.8822142 0.4464153 1.5688200  C 4.3858142 -2.8833517 1.0822020  H 2.9016542 -4.2615597 0.3510250  H 5.6484992 -1.2885797 1.7886930  H 5.1559652 -3.6422407 1.1802960  C -1.7088888 1.6576993 -0.1978800  C -2.1920948 0.4865523 -0.9325220  C -3.5752628 0.3803533 -1.1471930  C -1.3561588 -0.5135847 -1.4543950  C -4.1149418 -0.7031347 -1.8277250  H -4.2312278 1.1572863 -0.7619770  C -1.8976448 -1.5902297 -2.1461940  H -0.2828798 -0.4333097 -1.3347860  C -3.2752418 -1.6951387 -2.3285320  H -5.1886208 -0.7712567 -1.9719170  H -1.2374828 -2.3503207 -2.5529950  H -3.6914158 -2.5410077 -2.8669810  H -2.4279998 2.4724193 -0.1520760 |  |
| **TS2-C-*SR***  C 2.1437110 1.1866559 0.9892849  O 3.3979520 1.1223189 1.4261039  C 3.5209000 -0.0090721 2.2764709  C 2.2232620 -0.6567891 2.2177879  N 1.4055340 0.2562589 1.5522659  C 1.7213640 2.2125179 0.0540079  C 0.3607860 2.3822059 -0.1956721  C 2.6932870 2.9408409 -0.6344311  C -0.0362200 3.2942669 -1.1702841  H -0.4025220 1.8612099 0.3729079  C 2.2762400 3.8561809 -1.5986741  H 3.7514400 2.7800329 -0.4596271  C 0.9161870 4.0370059 -1.8802531  H 0.6009380 4.7454699 -2.6339081  C 1.6361840 -1.5630741 3.2688009  C 2.6485920 -2.0262061 4.3178979  H 0.8166900 -1.0238711 3.7608989  H 1.1746610 -2.4333351 2.7852989  H 2.1575900 -2.7056351 5.0200049  H 3.0494460 -1.1775481 4.8779529  H 3.4924570 -2.5485241 3.8611539  O 4.5747160 -0.2663831 2.7964399  C 1.8517630 -0.5994231 -1.3646731  O 0.6773610 -0.9298671 -1.0645651  C 2.0618060 0.2639699 -2.5801241  H 3.0768640 0.6526649 -2.6576531  H 1.3450960 1.0923839 -2.5481161  H 1.8327310 -0.3421471 -3.4632631  C 2.9624160 -1.0571541 -0.5804351  C 2.6802610 -1.9235491 0.5085329  C 3.5883380 -2.9091031 1.1262319  C 4.9712110 -2.7326671 1.2864219  C 3.0057320 -4.0930601 1.6106889  C 5.7331940 -3.7102801 1.9188589  H 5.4491460 -1.8291931 0.9328389  C 3.7709180 -5.0694501 2.2345379  H 1.9375770 -4.2425121 1.4802509  C 5.1416680 -4.8771061 2.3953649  H 6.7999680 -3.5521971 2.0433949  H 3.2983310 -5.9772781 2.5982939  H 5.7451690 -5.6333841 2.8884129  H 1.6394420 -2.2497841 0.5010839  C 4.2681880 -0.5534081 -0.8340181  C 5.3751660 -0.0668451 -0.9490691  C 6.6826130 0.5124599 -0.9054691  C 7.2392660 0.8191779 0.3474749  C 7.4150680 0.7776149 -2.0702461  C 8.5107420 1.3742799 0.4233739  H 6.6559500 0.6197069 1.2436049  C 8.6834070 1.3388369 -1.9829511  H 6.9805010 0.5381929 -3.0356161  C 9.2352280 1.6359549 -0.7384771  H 8.9370660 1.6071599 1.3945099  H 9.2449340 1.5432219 -2.8895801  H 10.2276310 2.0716209 -0.6733781  O 3.2668360 4.5299959 -2.2358141  O -1.3686000 3.4051539 -1.3534711  C 2.9020140 5.4643169 -3.2261901  H 2.3618260 4.9829369 -4.0506341  H 3.8350760 5.8832689 -3.6022421  H 2.2839610 6.2688849 -2.8087951  C -1.8278940 4.1863179 -2.4316851  H -2.9129130 4.0859869 -2.4281121  H -1.4225410 3.8208899 -3.3835051  H -1.5654360 5.2434469 -2.2975311  H -0.5513920 -0.3081591 -1.4617491  H 0.4066340 0.0645809 1.3210529  O -1.5331790 0.0722189 -1.4096581  P -2.1282310 -0.2748341 -0.0206341  O -2.8914600 -1.6993521 -0.1141041  O -3.3322340 0.8037809 0.0786949  O -1.2409020 -0.3043251 1.1670459  C -3.7512060 -1.8806721 -1.1904711  C -4.0892080 0.6866509 1.2420359  C -5.0713590 -1.4747181 -1.0656671  C -3.2611620 -2.4172631 -2.3902761  C -5.1722360 -0.1795151 1.2628669  C -3.6964030 1.3895539 2.3888179  C -5.8336990 -0.9626851 0.1428469  C -5.9159420 -1.5344361 -2.1776281  C -4.1509680 -2.5166281 -3.4669021  C -1.8304380 -2.7851441 -2.5802971  C -5.8391030 -0.4102031 2.4706739  C -4.4129130 1.1727499 3.5692259  C -2.5291010 2.3161539 2.3839849  C -6.9462050 -0.1339531 -0.5602331  C -6.4734080 -2.1245941 0.9545209  C -5.4653590 -2.0654281 -3.3780741  C -7.2565550 -0.9182651 -1.8489451  H -3.7823540 -2.9372451 -4.3979511  C -1.1516190 -3.7182761 -1.7315791  C -1.1529680 -2.2221701 -3.6409351  C -5.4691940 0.2651389 3.6248509  C -6.8920750 -1.4798821 2.2884129  H -4.1110270 1.7156639 4.4601639  C -2.5571810 3.5366209 1.6393589  C -1.4259180 2.0124089 3.1512539  H -6.5359090 0.8489949 -0.8179731  H -7.8191750 0.0186989 0.0821119  H -5.7050070 -2.8839131 1.1378289  H -7.3000490 -2.6001391 0.4175459  H -6.1204590 -2.1158311 -4.2431741  H -7.6317090 -0.2796611 -2.6541671  H -8.0135640 -1.6929041 -1.6720141  C -1.8001590 -4.4088081 -0.6728881  C 0.2229280 -4.0008001 -1.9826151  C 0.2006100 -2.5291591 -3.9021951  H -1.6717860 -1.5052211 -4.2712761  H -5.9833910 0.0813179 4.5639259  H -6.9109320 -2.1930191 3.1176469  H -7.8939110 -1.0371761 2.2196859  C -3.6742920 3.9261129 0.8516249  C -1.4367940 4.4130809 1.7115489  C -0.3109710 2.8788159 3.2103019  H -1.4099420 1.0711909 3.6932379  C -1.1198460 -5.3137171 0.1013129  H -2.8520020 -4.2204571 -0.4852481  C 0.9062410 -4.9243831 -1.1494451  C 0.8815280 -3.3853421 -3.0782771  H 0.6958890 -2.0731951 -4.7546811  C -3.6806610 5.1220869 0.1823539  H -4.5354000 3.2670429 0.7986069  C -1.4640770 5.6341659 0.9863949  C -0.3166570 4.0546239 2.5049889  H 0.5500730 2.6020049 3.8118789  C 0.2509450 -5.5721661 -0.1339461  H -1.6359280 -5.8364781 0.9006749  H 1.9606000 -5.1135411 -1.3368311  H 1.9301080 -3.6138001 -3.2526311  C -2.5621010 5.9883109 0.2474439  H -4.5497260 5.4107909 -0.4020161  H -0.5978910 6.2889849 1.0424129  H 0.5392470 4.7241529 2.5336899  H 0.7827370 -6.2864651 0.4887709  H -2.5804790 6.9334589 -0.2879961 | **TS2-C-*RS***  C -2.0833088 1.2912940 -0.6678925  O -3.0099028 2.0960220 -0.1541955  C -2.4060508 2.8769160 0.8690505  C -1.0901758 2.2780430 1.0524495  N -0.9155058 1.5116580 -0.1100195  C -2.4494038 0.3458730 -1.7068815  C -1.4851478 -0.4705170 -2.3062955  C -3.8096098 0.2020680 -1.9791015  C -1.9175998 -1.4555810 -3.1897995  H -0.4242708 -0.3817560 -2.0842995  C -4.2206378 -0.8028960 -2.8520745  H -4.5551568 0.8175070 -1.4912565  C -3.2812138 -1.6385750 -3.4640555  H -3.6030868 -2.4184610 -4.1403465  C 0.0910472 2.9698250 1.6906845  C -0.2597248 4.2976480 2.3621335  H 0.5537232 2.3006430 2.4250195  H 0.8523722 3.1301620 0.9163115  H 0.6539052 4.7506630 2.7583565  H -0.9685088 4.1552580 3.1817155  H -0.7114938 4.9939060 1.6523905  O -3.0292978 3.7381100 1.4202005  C -1.4637868 -1.2335030 1.0345695  O -0.2146108 -1.1634800 1.0784355  C -2.0611398 -2.4171270 0.3105615  H -1.2649178 -2.9410610 -0.2206015  H -2.8496678 -2.1055850 -0.3814225  H -2.5189528 -3.0948320 1.0422165  C -2.3312978 -0.2549850 1.6382695  C -1.7202528 0.7980000 2.3825015  C -2.3627278 1.5233310 3.5011175  C -1.5656238 1.8753920 4.6005235  C -3.7192428 1.8732780 3.5190785  C -2.1037488 2.5507010 5.6892445  H -0.5117448 1.6045410 4.5982085  C -4.2573988 2.5448710 4.6111265  H -4.3509738 1.6226630 2.6740765  C -3.4556948 2.8864970 5.6968715  H -1.4706508 2.8107020 6.5321405  H -5.3100598 2.8101870 4.6084375  H -3.8824968 3.4128860 6.5453175  H -0.6780598 0.5736600 2.6015875  C -3.7196318 -0.4100280 1.3853255  C -4.8404248 -0.6379340 0.9700465  C -6.0875658 -0.9361960 0.3402865  C -6.3575418 -2.2493190 -0.0790315  C -7.0194348 0.0732730 0.0565075  C -7.5313118 -2.5405660 -0.7618655  H -5.6336008 -3.0292390 0.1381975  C -8.1889168 -0.2256510 -0.6317925  H -6.8063998 1.0907070 0.3710685  C -8.4504258 -1.5305600 -1.0439735  H -7.7314258 -3.5615660 -1.0756845  H -8.9002678 0.5655800 -0.8485535  H -9.3681938 -1.7608870 -1.5768865  O -5.5579928 -0.8939900 -3.0455945  O -0.9399938 -2.2187740 -3.7410555  C -6.0469898 -1.9879200 -3.7871525  H -5.7031128 -1.9522040 -4.8287625  H -7.1332978 -1.9069590 -3.7560175  H -5.7436338 -2.9397300 -3.3323695  C -1.3197628 -3.2573420 -4.6139195  H -0.3941628 -3.7405700 -4.9264465  H -1.8423208 -2.8686560 -5.4970405  H -1.9595958 -3.9915060 -4.1085405  O 1.4269412 0.3744200 -0.9801145  P 2.3668012 0.1729400 0.1477095  O 3.5470832 1.2727200 0.2360065  O 3.1256872 -1.2360810 -0.0449885  O 1.7802352 0.2454690 1.5921525  C 4.1577202 1.4708810 -1.0067245  C 4.1271002 -1.5483430 0.8708845  C 5.1880252 0.6253640 -1.3876785  C 3.6443952 2.4385580 -1.8795065  C 5.4185892 -1.1075940 0.6213765  C 3.8059982 -2.2564230 2.0356085  C 5.9926022 -0.3766790 -0.5794535  C 5.6568912 0.6701840 -2.7034825  C 4.1685312 2.4932240 -3.1747025  C 2.6258912 3.4268870 -1.4184555  C 6.4153402 -1.3398070 1.5732875  C 4.8360172 -2.5009210 2.9516345  C 2.4201452 -2.7024820 2.3595335  C 6.4519772 -1.3405740 -1.7078405  C 7.2301772 0.3068380 0.0738145  C 5.1506552 1.6005010 -3.6016965  C 6.6765722 -0.4250620 -2.9274885  H 3.7741082 3.2392270 -3.8590815  C 1.2976742 3.4646640 -1.9502305  C 3.0091222 4.3600790 -0.4789845  C 6.1330232 -2.0421550 2.7365835  C 7.7094002 -0.6897080 1.1435175  H 4.5940302 -3.0503410 3.8565835  C 1.7454742 -3.6967560 1.5845595  C 1.8105762 -2.1874950 3.4830995  H 5.6382512 -2.0433240 -1.9188435  H 7.3374212 -1.9180970 -1.4244905  H 6.9003672 1.2326740 0.5576885  H 7.9969472 0.5631520 -0.6637205  H 5.5089132 1.6329130 -4.6266985  H 6.5284862 -0.9434120 -3.8793085  H 7.6951542 -0.0166670 -2.9375645  C 0.8237322 2.5237950 -2.9065675  C 0.4030102 4.4758760 -1.4885215  C 2.1327382 5.3838970 -0.0562155  H 4.0163442 4.3149530 -0.0744315  H 6.9059322 -2.2177290 3.4793585  H 8.2317132 -0.2035830 1.9726115  H 8.3970212 -1.4317230 0.7179685  C 2.3142712 -4.2802340 0.4213915  C 0.4644422 -4.1477810 2.0147805  C 0.5254912 -2.6134880 3.8863595  H 2.3276532 -1.4204000 4.0532155  C -0.4676338 2.5796100 -3.3649655  H 1.4925842 1.7392560 -3.2441475  C -0.9383948 4.4864950 -1.9606435  C 0.8589602 5.4450850 -0.5584085  H 2.4757052 6.1179690 0.6663665  C 1.6480562 -5.2557950 -0.2751315  H 3.2917332 -3.9467420 0.0874155  C -0.1935098 -5.1679160 1.2789105  C -0.1302768 -3.5797190 3.1703195  H 0.0655302 -2.1718490 4.7651485  C -1.3656108 3.5622800 -2.8781965  H -0.8177308 1.8457790 -4.0845245  H -1.6188218 5.2427250 -1.5763945  H 0.1737962 6.2271160 -0.2415345  C 0.3798922 -5.7102620 0.1587725  H 2.0979702 -5.6886680 -1.1633515  H -1.1673388 -5.5081510 1.6231285  H -1.1153788 -3.9246950 3.4756295  H -2.3939468 3.5725460 -3.2277575  H -0.1327688 -6.4906540 -0.3954165  H -0.0430218 1.0222000 -0.3893225  H 0.9036332 -0.3028810 1.5900675 |
| **TS2-C-*RR***  C -1.4966612 2.6809177 0.3840929  O -2.8141242 2.7868087 0.3498939  C -3.3725112 1.7425907 1.1770899  C -2.2310242 0.9714667 1.6284799  N -1.1252092 1.7156537 1.1848419  C -0.6290962 3.5853797 -0.3540651  C 0.7284658 3.2793587 -0.4576511  C -1.1701812 4.7314577 -0.9327091  C 1.5652518 4.1779107 -1.1107061  H 1.1495968 2.3541427 -0.0749801  C -0.3176722 5.6033457 -1.6090811  H -2.2284852 4.9548697 -0.8677771  C 1.0552438 5.3434807 -1.6943891  H 1.7134608 6.0314127 -2.2070581  C -2.1324202 0.2824087 2.9694499  C -3.4846002 0.0879747 3.6568049  H -1.4806452 0.8879467 3.6123229  H -1.6317902 -0.6840143 2.8460079  H -3.3430232 -0.4776283 4.5820879  H -3.9409382 1.0499477 3.9062229  H -4.1898002 -0.4494893 3.0182899  O -4.5619252 1.6416687 1.2795189  C -2.5188152 -2.7049173 1.5631539  O -1.2862452 -2.8819863 1.7524389  C -3.4390022 -3.7194043 2.2035309  H -2.8394352 -4.5223173 2.6325759  H -4.0318722 -3.2396553 2.9902089  H -4.1508162 -4.1187153 1.4749829  C -3.1067162 -1.6536513 0.7958809  C -2.2725072 -0.6624293 0.2220229  C -2.4892672 0.0803747 -1.0348531  C -1.3532732 0.6045647 -1.6742931  C -3.7544912 0.3423577 -1.5889231  C -1.4769412 1.4073167 -2.8044731  H -0.3657832 0.3781337 -1.2757251  C -3.8680142 1.1363627 -2.7228691  H -4.6452572 -0.0549593 -1.1189041  C -2.7352972 1.6856967 -3.3251001  H -0.5830502 1.8115177 -3.2711491  H -4.8530092 1.3395427 -3.1326731  H -2.8364602 2.3190237 -4.2018371  H -1.2152892 -0.8459303 0.4084309  C -4.5254122 -1.6105293 0.6993709  C -5.7387812 -1.5575713 0.6575089  C -7.1462102 -1.3144733 0.5907229  C -7.5972332 0.0120907 0.4866419  C -8.0811082 -2.3584863 0.6209319  C -8.9582952 0.2782397 0.4071779  H -6.8644592 0.8143027 0.4850499  C -9.4409212 -2.0803833 0.5473799  H -7.7293552 -3.3823563 0.7006199  C -9.8836742 -0.7639043 0.4369149  H -9.3000222 1.3059687 0.3279219  H -10.1582552 -2.8952663 0.5733959  H -10.9466382 -0.5509073 0.3766779  O -0.9073492 6.6961767 -2.1529651  O 2.8785388 3.8371577 -1.1238391  C -0.0911752 7.6162347 -2.8440101  H 0.4077778 7.1431727 -3.6984631  H -0.7586032 8.3989817 -3.2033531  H 0.6627918 8.0568147 -2.1802971  C 3.7842968 4.6880157 -1.7901111  H 4.7635858 4.2225137 -1.6802011  H 3.5386408 4.7759347 -2.8554581  H 3.8018368 5.6871607 -1.3372211  H -0.0667122 -2.4217313 1.3289589  O 1.0318968 0.2348577 0.6420339  P 1.7018688 -1.0883383 0.5950369  O 3.1098638 -1.1283873 1.3883829  O 2.1342208 -1.3696263 -0.9424961  O 0.9920868 -2.3286253 1.1531269  C 4.0540698 -0.1764023 1.0207939  C 2.7030028 -2.6170593 -1.1910711  C 4.9534078 -0.4975583 0.0141749  C 4.0613698 1.0905647 1.6280009  C 4.0635168 -2.8018323 -0.9944891  C 1.8693538 -3.6782223 -1.5656141  C 5.1756708 -1.8044283 -0.7242951  C 5.8491898 0.4719547 -0.4445391  C 5.0116138 2.0185737 1.1812899  C 3.0253778 1.5120597 2.6092729  C 4.5943858 -4.0937113 -1.0768281  C 2.4412388 -4.9480733 -1.6912051  C 0.4074488 -3.4919323 -1.7767821  C 5.7941138 -1.2728913 -2.0493051  C 6.2017588 -2.7176883 0.0038099  C 5.8951758 1.7264997 0.1469479  C 6.6350338 -0.0566013 -1.6213971  H 5.0300628 2.9978947 1.6508159  C 2.6849578 0.7399517 3.7655589  C 2.3492978 2.6882817 2.3590409  C 3.7914858 -5.1691763 -1.4273831  C 6.0561038 -4.0845213 -0.6904101  H 1.7972668 -5.7743223 -1.9775811  C -0.0975052 -2.6564603 -2.8257331  C -0.4721632 -4.1863503 -0.9771581  H 4.9798328 -0.9452923 -2.7054271  H 6.3678178 -2.0416493 -2.5756181  H 5.9057628 -2.8040863 1.0552049  H 7.2178738 -2.3123303 -0.0329121  H 6.6021908 2.4764197 -0.1982181  H 6.7474798 0.6853567 -2.4175891  H 7.6451608 -0.3585923 -1.3163021  C 3.4134848 -0.4139383 4.1614389  C 1.5917088 1.1611957 4.5766339  C 1.2744578 3.1100597 3.1722699  H 2.6208918 3.2726367 1.4842089  H 4.2038078 -6.1724763 -1.4868881  H 6.3201538 -4.9220283 -0.0381551  H 6.6991738 -4.1506343 -1.5774131  C 0.7481038 -1.9365153 -3.7116631  C -1.5073332 -2.5654733 -3.0123791  C -1.8690882 -4.0831223 -1.1628391  H -0.0816732 -4.7957033 -0.1668171  C 3.0541228 -1.1279893 5.2743549  H 4.2702198 -0.7235573 3.5725249  C 1.2325808 0.3895067 5.7143209  C 0.8898098 2.3501727 4.2473309  H 0.7461238 4.0265387 2.9246459  C 0.2222668 -1.1789953 -4.7274661  H 1.8243908 -2.0034033 -3.5857811  C -2.0205852 -1.7651623 -4.0658931  C -2.3770342 -3.2829253 -2.1504031  H -2.5346062 -4.6295943 -0.5003831  C 1.9417348 -0.7317183 6.0546569  H 3.6243448 -2.0063493 5.5596339  H 0.3863438 0.7138307 6.3150029  H 0.0552738 2.6551487 4.8744239  C -1.1778332 -1.0928773 -4.9106721  H 0.8855988 -0.6407203 -5.3983101  H -3.0989582 -1.6868193 -4.1788121  H -3.4511022 -3.1739413 -2.2815401  H 1.6602888 -1.3136153 6.9267309  H -1.5815472 -0.4777113 -5.7094421  H -0.1600432 1.3622407 1.2630709 | **TS2-C-*SS***  C -3.3195420 0.8506311 -0.1390240  O -4.2477690 1.7666151 0.1109070  C -3.7513310 2.6657861 1.1226620  C -2.3678750 2.2987471 1.2481750  N -2.2399130 1.0916391 0.5743790  C -3.6251370 -0.2020579 -1.0966880  C -2.6983540 -1.1946589 -1.3936180  C -4.8803320 -0.1614419 -1.7185870  C -3.0418330 -2.1709569 -2.3291280  H -1.7155180 -1.2527319 -0.9423950  C -5.1964060 -1.1367339 -2.6571750  H -5.6068700 0.6075301 -1.4859550  C -4.2815490 -2.1468579 -2.9750460  H -4.5340120 -2.9060769 -3.7021710  C -1.4033280 2.6586401 2.3393800  C -1.9791270 3.6322911 3.3667990  H -1.0884960 1.7258361 2.8200330  H -0.4909590 3.0685721 1.8897360  H -1.2280420 3.8322291 4.1354320  H -2.8685900 3.2173731 3.8504250  H -2.2684140 4.5827001 2.9110230  O -4.4655260 3.5228601 1.5662210  C -1.4835130 2.1048981 -2.2749030  O -0.7702040 1.1077591 -2.7375880  C -2.6935530 2.3501501 -3.1237480  H -3.2913320 1.4365791 -3.1935130  H -2.3339710 2.5782201 -4.1336230  H -3.3131250 3.1791421 -2.7866640  C -1.0565450 2.9158321 -1.2192000  C -1.9805340 3.7380241 -0.5105030  C -1.7446040 4.9724181 0.2419700  C -2.8721310 5.7795161 0.4884480  C -0.4988060 5.3981941 0.7396020  C -2.7674310 6.9535861 1.2224430  H -3.8402120 5.4684911 0.1094580  C -0.4011470 6.5751841 1.4708190  H 0.3907280 4.8115591 0.5549270  C -1.5304620 7.3525511 1.7217220  H -3.6528570 7.5534701 1.4067130  H 0.5678950 6.8857001 1.8490590  H -1.4450990 8.2684411 2.2986940  H -2.9891400 3.7058291 -0.9141100  C 0.2927180 2.7885461 -0.7631450  C 1.4279760 2.7947271 -0.3375580  C 2.7481720 2.9595941 0.1941910  C 3.4423960 4.1517551 -0.0682050  C 3.3367770 1.9704531 0.9881220  C 4.7151190 4.3424251 0.4509110  H 2.9751000 4.9097741 -0.6906010  C 4.6209010 2.1644161 1.4879310  H 2.7845650 1.0631781 1.2011120  C 5.3097690 3.3440231 1.2222980  H 5.2521110 5.2630221 0.2421860  H 5.0852170 1.3857431 2.0849670  H 6.3153450 3.4834741 1.6078150  O -6.4284140 -1.0353209 -3.2200730  O -2.1059760 -3.1205059 -2.5431680  C -6.8062340 -2.0124339 -4.1637850  H -6.1421000 -2.0049459 -5.0366600  H -7.8173060 -1.7531279 -4.4771730  H -6.8098990 -3.0158249 -3.7196190  C -2.4043580 -4.1563019 -3.4494770  H -1.5306670 -4.8070549 -3.4590360  H -2.5774030 -3.7670009 -4.4605270  H -3.2839280 -4.7283489 -3.1255000  H -0.1691000 0.6481631 -2.0618860  H -1.3323580 0.5593071 0.6580150  O 0.3760450 -0.6299089 -1.2496120  P 0.7055540 -0.8209219 0.2003020  O 2.3178240 -1.0062929 0.4278250  O 0.1253370 -2.3209209 0.5738230  O 0.2078290 0.1636921 1.2148380  C 2.9630250 -1.9106399 -0.4023570  C 0.3414610 -2.7389309 1.8744040  C 2.9739190 -3.2569409 -0.0551130  C 3.5862720 -1.4786019 -1.5793460  C 1.5179830 -3.4122519 2.1724710  C -0.5948990 -2.4666219 2.8848110  C 2.5927030 -3.9382269 1.2440520  C 3.4811510 -4.2018579 -0.9505840  C 4.1461820 -2.4424309 -2.4260540  C 3.7314970 -0.0400369 -1.9534560  C 1.8229080 -3.7383979 3.4970340  C -0.2785700 -2.8389889 4.1970140  C -1.9090580 -1.8135459 2.6361890  C 2.2642050 -5.3662079 0.7285890  C 3.8054900 -3.9923709 2.2177700  C 4.0683930 -3.8029299 -2.1428810  C 3.2688500 -5.6004659 -0.4152260  H 4.6288440 -2.1004299 -3.3373110  C 4.9638360 0.6333731 -1.6684880  C 2.7583430 0.5989061 -2.6838330  C 0.9292170 -3.4517229 4.5180390  C 3.1945140 -4.3680359 3.5790310  H -1.0064800 -2.6330939 4.9765820  C -2.8858710 -2.4068169 1.7720780  C -2.2419410 -0.6848909 3.3560730  H 1.2435780 -5.3613799 0.3291120  H 2.3183070 -6.1179189 1.5225180  H 4.2453640 -2.9905439 2.2767220  H 4.5809230 -4.6850869 1.8760560  H 4.4647950 -4.5339269 -2.8420680  H 2.8892050 -6.2861219 -1.1789010  H 4.2085390 -6.0258149 -0.0403870  C 6.0107110 0.0295961 -0.9212100  C 5.1586590 1.9524321 -2.1676510  C 2.9457930 1.9209991 -3.1521040  H 1.8416220 0.0662631 -2.9080120  H 1.1619050 -3.7002249 5.5496640  H 3.7782500 -3.9945689 4.4257830  H 3.1179700 -5.4570469 3.6950790  C -2.6375870 -3.6007659 1.0395180  C -4.1754170 -1.8068409 1.6751510  C -3.5189930 -0.0880439 3.2507900  H -1.4957430 -0.2508799 4.0160290  C 7.1928810 0.6919801 -0.7007400  H 5.8638270 -0.9711839 -0.5262640  C 6.3932530 2.6080801 -1.9249640  C 4.1211310 2.5791811 -2.9077730  H 2.1465300 2.4021461 -3.7080350  C -3.6062110 -4.1513199 0.2418540  H -1.6678770 -4.0791369 1.1202700  C -5.1542070 -2.3977229 0.8309560  C -4.4659610 -0.6344759 2.4221660  H -3.7447050 0.8041771 3.8291060  C 7.3927320 1.9934751 -1.2155240  H 7.9834280 0.2120561 -0.1312110  H 6.5288310 3.6144181 -2.3146030  H 4.2800700 3.5915141 -3.2721500  C -4.8778340 -3.5394779 0.1267850  H -3.3943770 -5.0625199 -0.3102580  H -6.1278750 -1.9202889 0.7523120  H -5.4514560 -0.1838519 2.3287300  H 8.3348760 2.5046001 -1.0407310  H -5.6329540 -3.9784809 -0.5192030 |
| **INT3-C-*SR***  C 2.0061871 1.5283346 0.8001359  O 3.3138061 1.6112916 1.1807529  C 3.5624601 0.5107476 1.9881819  C 2.3026761 -0.3458984 1.9391399  N 1.3499521 0.5368636 1.2660249  C 1.5237651 2.5305636 -0.1521461  C 0.1537701 2.6483686 -0.3550091  C 2.4445701 3.2634946 -0.8997221  C -0.3117579 3.5190026 -1.3362931  H -0.5647719 2.1096946 0.2519659  C 1.9632531 4.1318686 -1.8786671  H 3.5129541 3.1550126 -0.7535131  C 0.5887811 4.2646426 -2.1071611  H 0.2218211 4.9391086 -2.8684691  C 1.7554361 -0.7648544 3.3170939  C 2.7579771 -1.2349664 4.3726549  H 1.2131051 0.1040586 3.7094529  H 0.9993211 -1.5386324 3.1330289  H 2.2105871 -1.4734874 5.2894929  H 3.4861951 -0.4552784 4.6052349  H 3.3067961 -2.1265094 4.0649639  O 4.6050721 0.3682176 2.5498179  C 2.0373161 -0.8125744 -1.3765421  O 0.7768941 -1.2032084 -1.1437771  C 2.2799311 -0.0897374 -2.6641381  H 3.3085161 0.2630896 -2.7363411  H 1.6013641 0.7727586 -2.7148041  H 2.0515401 -0.7430964 -3.5120041  C 2.9754311 -1.0588444 -0.4240351  C 2.5220461 -1.5682254 0.9380469  C 3.3193941 -2.7009164 1.5508109  C 4.7043991 -2.6615614 1.7467739  C 2.6143991 -3.8268454 1.9890179  C 5.3557641 -3.7209414 2.3734099  H 5.2777921 -1.8010134 1.4253859  C 3.2652291 -4.8845824 2.6171869  H 1.5388591 -3.8703404 1.8347699  C 4.6421691 -4.8323404 2.8147049  H 6.4302741 -3.6713424 2.5218479  H 2.6952981 -5.7466334 2.9526439  H 5.1563271 -5.6521674 3.3073279  H 1.5025191 -1.9394774 0.7979769  C 4.3226261 -0.6463144 -0.6320861  C 5.4668361 -0.2554274 -0.7444491  C 6.8117401 0.2312296 -0.7849301  C 7.3870251 0.7520806 0.3851319  C 7.5648371 0.2004596 -1.9672711  C 8.6932261 1.2253446 0.3660989  H 6.7909241 0.7841156 1.2927269  C 8.8681571 0.6822746 -1.9770351  H 7.1180261 -0.2046044 -2.8696801  C 9.4370441 1.1933246 -0.8121861  H 9.1311201 1.6260386 1.2753429  H 9.4441261 0.6565166 -2.8971771  H 10.4565541 1.5664506 -0.8234161  O 2.9084571 4.8131186 -2.5756751  O -1.6531049 3.5858406 -1.4652051  C 2.4786671 5.7102146 -3.5743841  H 1.9227761 5.1924856 -4.3658061  H 3.3839251 6.1466546 -3.9959091  H 1.8523421 6.5069066 -3.1540391  C -2.1818549 4.3673746 -2.5100091  H -3.2627539 4.2382046 -2.4588641  H -1.8139059 4.0249786 -3.4851431  H -1.9402929 5.4294456 -2.3726931  H 0.1174011 -0.7512874 -1.7155741  H -0.1601289 0.0727606 0.9689399  O -1.4431709 0.1414186 -1.5878621  P -2.0210509 -0.2283184 -0.2779671  O -2.6587739 -1.7154904 -0.1994391  O -3.2763799 0.7463856 0.0416029  O -1.1385869 -0.2503164 1.0045749  C -3.5754379 -2.0168634 -1.1995271  C -3.9412449 0.5527036 1.2471429  C -4.9086249 -1.6951454 -0.9997611  C -3.1337979 -2.5863374 -2.4016421  C -4.9544439 -0.3924524 1.3284079  C -3.5456199 1.2882616 2.3725899  C -5.6262279 -1.2224854 0.2489119  C -5.8182719 -1.8473544 -2.0493391  C -4.0820419 -2.7793694 -3.4139121  C -1.7054559 -2.9250034 -2.6515171  C -5.5295099 -0.6726254 2.5731039  C -4.1715059 1.0160186 3.5926369  C -2.4677639 2.3154536 2.3146859  C -6.8379689 -0.4859044 -0.3880031  C -6.1265559 -2.4285784 1.0942109  C -5.4118739 -2.3946974 -3.2582961  C -7.1737759 -1.3127294 -1.6446581  H -3.7499189 -3.2237004 -4.3477661  C -0.9696149 -3.8175114 -1.8054191  C -1.0941819 -2.3905304 -3.7655501  C -5.1474519 0.0286006 3.7072139  C -6.5115019 -1.8153804 2.4513929  H -3.8637379 1.5845056 4.4654039  C -2.6491659 3.5465556 1.6075839  C -1.3052569 2.1019776 3.0221999  H -6.5170979 0.5194906 -0.6830401  H -7.6760299 -0.3863624 0.3087119  H -5.2926379 -3.1266874 1.2291439  H -6.9445679 -2.9653744 0.6043209  H -6.1161379 -2.5178674 -4.0761591  H -7.6399989 -0.7150544 -2.4335171  H -7.8650729 -2.1322194 -1.4095981  C -1.5415019 -4.4540844 -0.6718571  C 0.3907751 -4.1000184 -2.1234581  C 0.2442731 -2.7024284 -4.0940921  H -1.6544329 -1.6998884 -4.3893111  H -5.5924199 -0.1928624 4.6731639  H -6.4296569 -2.5268134 3.2783769  H -7.5449889 -1.4458394 2.4432119  C -3.8349809 3.8479666 0.8850569  C -1.6133739 4.5231986 1.6539279  C -0.2781209 3.0718736 3.0602439  H -1.1763279 1.1554326 3.5401599  C -0.7980109 -5.2970624 0.1146919  H -2.5842999 -4.2728214 -0.4331211  C 1.1412381 -4.9563894 -1.2770821  C 0.9754971 -3.5301234 -3.2833691  H 0.6880311 -2.2727034 -4.9877011  C -3.9830939 5.0531036 0.2480399  H -4.6332929 3.1128306 0.8534779  C -1.7875219 5.7517726 0.9634459  C -0.4293179 4.2573916 2.3897469  H 0.6311391 2.8671036 3.6186669  C 0.5626451 -5.5456184 -0.1832591  H -1.2566419 -5.7767904 0.9741229  H 2.1868291 -5.1359594 -1.5134051  H 2.0126781 -3.7625134 -3.5127351  C -2.9468709 6.0170966 0.2829199  H -4.9023999 5.2729636 -0.2873181  H -0.9851729 6.4846276 1.0010959  H 0.3572481 5.0078596 2.4020049  H 1.1491541 -6.1986144 0.4565369  H -3.0778169 6.9684706 -0.2249781 | **INT3-C-*RS***  C -2.0309542 0.7137707 -1.2756789  O -3.1062482 1.4834087 -1.0759029  C -2.7528932 2.4688047 -0.1280889  C -1.3943742 2.0619507 0.4123911  N -0.9884622 1.1162347 -0.6266869  C -2.1807342 -0.5073643 -2.0599619  C -1.1042412 -1.3891833 -2.1559149  C -3.4539822 -0.8287903 -2.5317459  C -1.3253942 -2.6344683 -2.7414519  H -0.1184782 -1.1582643 -1.7595779  C -3.6508272 -2.0805273 -3.1121299  H -4.2904742 -0.1487083 -2.4219949  C -2.5936402 -2.9904703 -3.2193819  H -2.7547752 -3.9646103 -3.6608869  C -0.3570162 3.1716087 0.6477381  C -0.9145172 4.5407907 1.0314721  H 0.3265198 2.8080917 1.4225051  H 0.2383828 3.2602327 -0.2631879  H -0.0751032 5.2114017 1.2370731  H -1.5463662 4.4951977 1.9223721  H -1.5023762 4.9761447 0.2194181  O -3.4970442 3.3542837 0.1320001  C -1.7612662 -1.2384733 1.1565571  O -0.4429982 -1.3920023 1.0538451  C -2.4965782 -2.4968373 0.7896431  H -1.7711442 -3.2496803 0.4734301  H -3.2101822 -2.3060993 -0.0201769  H -3.0665252 -2.8717813 1.6458891  C -2.3979372 -0.0766073 1.4962641  C -1.6141322 1.1897367 1.7537871  C -2.0505762 2.0514807 2.9213001  C -1.0808432 2.3681237 3.8792291  C -3.3433132 2.5635777 3.0778801  C -1.3846662 3.1863807 4.9626151  H -0.0760162 1.9657887 3.7653131  C -3.6454292 3.3831587 4.1623511  H -4.1153572 2.3328427 2.3536731  C -2.6705862 3.7000147 5.1044501  H -0.6169412 3.4200267 5.6940731  H -4.6509742 3.7794497 4.2674811  H -2.9137292 4.3409077 5.9465991  H -0.5852162 0.9092947 1.9776581  C -3.8203802 -0.0685003 1.4279651  C -5.0260882 -0.0824533 1.2809231  C -6.4445252 -0.0926223 1.0941861  C -7.0096822 -0.7909423 0.0160611  C -7.2857932 0.5996937 1.9772951  C -8.3867672 -0.7962503 -0.1679509  H -6.3568262 -1.3204013 -0.6723109  C -8.6614362 0.5946517 1.7825011  H -6.8448392 1.1347757 2.8128181  C -9.2161192 -0.1037863 0.7119801  H -8.8154672 -1.3381593 -1.0055919  H -9.3038322 1.1366677 2.4698451  H -10.2914192 -0.1067303 0.5627591  O -4.9140422 -2.3385413 -3.5337329  O -0.2498622 -3.4504953 -2.7940399  C -5.1812982 -3.6061093 -4.0940949  H -4.5852002 -3.7763363 -4.9988429  H -6.2391642 -3.6021053 -4.3550209  H -4.9867392 -4.4090213 -3.3727389  C -0.4386452 -4.7870903 -3.1998749  H 0.5318208 -5.2702423 -3.0888729  H -0.7585622 -4.8491213 -4.2476219  H -1.1722492 -5.2946793 -2.5609479  O 1.3907298 0.2507667 -0.9988149  P 2.1866518 0.3547107 0.2787391  O 3.2400458 1.6044017 0.1667781  O 3.1599888 -0.9550423 0.2691221  O 1.4516508 0.4993017 1.5753101  C 3.9899208 1.6178587 -0.9998939  C 4.0239858 -1.0549913 1.3450171  C 5.1830588 0.9056657 -1.0396839  C 3.5141418 2.2821947 -2.1401209  C 5.2645598 -0.4411643 1.2597211  C 3.6214388 -1.7208443 2.5105081  C 5.9484988 0.2076347 0.0718931  C 5.8720728 0.7816847 -2.2491779  C 4.2467568 2.1704917 -3.3268999  C 2.3032088 3.1570667 -2.1156499  C 6.0978188 -0.4061233 2.3796681  C 4.4989148 -1.7083133 3.6018631  C 2.2967658 -2.3912493 2.6432011  C 6.7515708 -0.8380493 -0.7541159  C 6.9254858 1.1709397 0.8037401  C 5.4105578 1.4096487 -3.3976369  C 7.0692398 -0.1249173 -2.0802979  H 3.8754188 2.6824217 -4.2107979  C 1.1031448 2.8074907 -2.8141649  C 2.3824108 4.3771377 -1.4787639  C 5.7217198 -1.0418583 3.5551181  C 7.3337538 0.4124087 2.0818861  H 4.1955278 -2.2235983 4.5087881  C 1.8961798 -3.4663913 1.7850191  C 1.4671738 -2.0081983 3.6752861  H 6.1010508 -1.6986733 -0.9471819  H 7.6393468 -1.1936053 -0.2215869  H 6.3774258 2.0799607 1.0748921  H 7.7742648 1.4588357 0.1753181  H 5.9421878 1.3069647 -4.3394429  H 7.1917448 -0.8187583 -2.9173699  H 7.9954828 0.4596437 -2.0064729  C 0.9453488 1.5629317 -3.4854949  C 0.0146828 3.7295167 -2.8239379  C 1.3192808 5.3071777 -1.5308979  H 3.2942968 4.6338107 -0.9474309  H 6.3643388 -1.0181743 4.4307051  H 7.6015278 1.0819547 2.9046731  H 8.1996068 -0.2371843 1.8998831  C 2.6911768 -3.9235613 0.7000641  C 0.6570618 -4.1233083 2.0379511  C 0.2405828 -2.6643493 3.9229421  H 1.7648828 -1.1706403 4.2996631  C -0.2341012 1.2470517 -4.1089729  H 1.7623288 0.8504787 -3.4578529  C -1.2048972 3.3591507 -3.4554169  C 0.1610978 4.9929457 -2.1936209  H 1.4265948 6.2698307 -1.0401779  C 2.2683508 -4.9515693 -0.1035029  H 3.6464218 -3.4471213 0.5075511  C 0.2501898 -5.1892693 1.1929511  C -0.1522272 -3.7064563 3.1255641  H -0.3863122 -2.3332233 4.7452731  C -1.3287732 2.1470457 -4.0839149  H -0.3436562 0.2859977 -4.6025039  H -2.0354762 4.0608327 -3.4339099  H -0.6661422 5.6973157 -2.2339349  C 1.0313138 -5.5927103 0.1413471  H 2.8906138 -5.2797603 -0.9308289  H -0.6972402 -5.6815083 1.4015981  H -1.0927342 -4.2221483 3.3057641  H -2.2646352 1.8704907 -4.5615549  H 0.7121078 -6.4141053 -0.4943359  H 0.0233758 0.7058757 -0.7465609  H 0.1216518 -0.6538103 1.3961971 |
| **TS3-C-*SRS***  C -0.7353281 1.7680630 3.1367383  O 0.1624959 0.8672060 3.3578663  C -0.9580141 2.6724360 4.3073403  H -0.2824551 3.5316800 4.2165103  H -1.9795381 3.0522740 4.3537283  H -0.7073031 2.1315510 5.2213743  C -1.4345761 1.9033340 1.9214623  C -1.3733511 0.8480810 1.0601923  C -1.2258311 -0.1172670 0.2564003  C -1.9387291 -1.3559960 -0.0514197  C -1.4162601 -2.2476280 -0.9942157  C -3.1623441 -1.6406960 0.5694273  C -2.1333591 -3.3906630 -1.3386357  H -0.4491551 -2.0387760 -1.4441137  C -3.8794351 -2.7739470 0.2127333  H -3.5583171 -0.9425390 1.3030743  C -3.3720321 -3.6426380 -0.7533067  H -1.7207011 -4.0897920 -2.0599557  H -4.8422231 -2.9709170 0.6763903  H -3.9357521 -4.5265210 -1.0381247  H 0.6061969 0.4406140 2.5297643  H -0.0889441 0.0856420 -0.4026427  C -2.2428421 3.1723040 1.6503033  C -3.0824431 3.1179940 0.3461823  C -1.3476091 4.4006840 1.7555703  C -2.3076491 2.8068930 -0.9339427  N -4.0840061 2.0560330 0.4096743  C -3.7975721 4.4727880 0.1128073  C -0.0703071 4.4177830 1.1878523  C -1.7759081 5.5106260 2.4885213  O -2.9957071 1.7867540 -1.5555687  O -1.3702371 3.3489850 -1.4340437  C -3.9979451 1.4130060 -0.6855677  C -4.7276541 4.4858990 -1.0975917  H -4.3642621 4.7017710 1.0231603  H -3.0237991 5.2377660 -0.0000977  C 0.7599179 5.5213410 1.3622073  H 0.2854749 3.5636470 0.6169723  C -0.9507011 6.6193240 2.6542883  H -2.7626981 5.4976230 2.9481293  C -4.8547991 0.3103520 -1.1439637  H -5.1926911 5.4699340 -1.1971857  H -5.5238871 3.7435540 -0.9968797  H -4.1829691 4.2857980 -2.0267897  C 0.3238609 6.6231340 2.0946403  H 1.7584189 5.5045560 0.9364893  H -1.2972301 7.4701630 3.2332393  C -6.0048211 0.0242660 -0.4247057  C -4.4671511 -0.4451710 -2.2580737  H 0.9793389 7.4771470 2.2367533  C -6.7966751 -1.0589150 -0.8212967  H -6.2863391 0.6171520 0.4379153  C -5.2562651 -1.5298980 -2.6222307  H -3.5502681 -0.1992770 -2.7768467  C -6.4275391 -1.8350230 -1.9142637  O -7.9044481 -1.2853010 -0.0697557  O -4.9730041 -2.3687080 -3.6475017  H -7.0043021 -2.6896490 -2.2458307  C -8.7308591 -2.3674660 -0.4382577  C -3.7963871 -2.1096460 -4.3878327  H -9.5538441 -2.3764950 0.2762523  H -8.1886641 -3.3195670 -0.3827067  H -9.1282181 -2.2382500 -1.4521227  H -3.8497901 -1.1327260 -4.8830577  H -3.7356351 -2.8959080 -5.1403587  H -2.9106801 -2.1447570 -3.7434077  H -3.0138841 3.2576630 2.4287413  O 1.0871949 0.0682620 -0.8900917  P 2.0504019 -0.3556850 0.2149503  O 3.5425199 0.1949050 -0.1043577  O 2.1880119 -1.9697620 0.0110133  O 1.7469539 0.0090380 1.6293143  C 4.0486429 -0.0705060 -1.3694437  C 3.0336359 -2.6143110 0.9022023  C 4.7025889 -1.2698970 -1.6078167  C 3.8702769 0.8711570 -2.3899057  C 4.3897259 -2.6892910 0.6216033  C 2.5147599 -3.1419250 2.0898533  C 5.1487589 -2.3532570 -0.6463497  C 5.0927489 -1.5903330 -2.9119477  C 4.3044649 0.5388850 -3.6765067  C 3.2478569 2.1999370 -2.1356387  C 5.2660089 -3.1904400 1.5888973  C 3.4072649 -3.6882030 3.0167043  C 1.0497459 -3.1533090 2.3708223  C 5.2156459 -3.5734090 -1.6090457  C 6.5584609 -2.0634210 -0.0593817  C 4.8927349 -0.6940190 -3.9521617  C 5.6826309 -2.9819270 -2.9516547  H 4.1609959 1.2673480 -4.4692137  C 3.9340959 3.1900080 -1.3619657  C 2.0364799 2.5033620 -2.7108477  C 4.7827699 -3.6909580 2.7896333  C 6.6961389 -3.0493500 1.1169523  H 3.0043569 -4.0935520 3.9403013  C 0.2165629 -4.1589460 1.7846813  C 0.5113929 -2.2580310 3.2641183  H 4.2055839 -3.9837950 -1.7189647  H 5.8664289 -4.3666660 -1.2280647  H 6.5707039 -1.0349150 0.3186993  H 7.3490169 -2.1616680 -0.8099937  H 5.1893549 -0.9455370 -4.9666007  H 5.3375619 -3.5601660 -3.8140177  H 6.7781379 -2.9406360 -3.0084677  C 5.1887619 2.9472550 -0.7404487  C 3.3485709 4.4819860 -1.2265167  C 1.4502709 3.7809140 -2.5587247  H 1.5131769 1.7326360 -3.2681267  H 5.4625699 -4.0765880 3.5441273  H 7.3611359 -2.6799350 1.9031803  H 7.0967979 -4.0148380 0.7824363  C 0.7088939 -5.1112300 0.8525373  C -1.1504231 -4.2337460 2.1760783  C -0.8613391 -2.2998670 3.6058913  H 1.1564729 -1.5036210 3.7035053  C 5.8242869 3.9285320 -0.0232887  H 5.6459199 1.9678300 -0.8420417  C 4.0391469 5.4804120 -0.4884827  C 2.0942729 4.7495550 -1.8359117  H 0.4799259 3.9763770 -2.9995187  C -0.1053551 -6.1006230 0.3605093  H 1.7479079 -5.0534360 0.5421623  C -1.9616611 -5.2762700 1.6591093  C -1.6706081 -3.2736400 3.0837163  H -1.2576401 -1.5636580 4.2988763  C 5.2456489 5.2132530 0.1043173  H 6.7807369 3.7221490 0.4472033  H 3.5902309 6.4685070 -0.4091217  H 1.6510089 5.7348900 -1.7119907  C -1.4533911 -6.1948040 0.7776023  H 0.2898869 -6.8238390 -0.3467277  H -3.0012751 -5.3264510 1.9742053  H -2.7231361 -3.3284560 3.3514913  H 5.7628399 5.9854070 0.6657703  H -2.0846091 -6.9893780 0.3904603 | **TS3-C-*SRR***  C -1.8048448 -1.0695144 2.5374464  O -2.2837828 -2.3267214 2.2191254  C -3.5340948 -2.1341584 1.6775424  C -3.8396818 -0.6402324 1.8305914  N -2.6014418 -0.0919374 2.3696794  C -0.4080868 -1.0066324 2.9769314  C 0.1498222 0.2445386 3.1782224  C 0.3399972 -2.1827524 3.1117264  C 1.5074602 0.3412396 3.4898084  H -0.4508748 1.1397136 3.0729234  C 1.6846572 -2.0640344 3.4420314  H -0.1300508 -3.1421874 2.9387214  C 2.2791822 -0.8090614 3.6153084  H 3.3366892 -0.7833744 3.8476274  C -4.9829188 -0.5056514 2.8577234  C -4.6733548 -1.1554974 4.2034874  H -5.1723308 0.5663426 2.9919824  H -5.8800828 -0.9536694 2.4189634  H -5.5038588 -1.0008674 4.8969094  H -3.7726578 -0.7256744 4.6503054  H -4.5282178 -2.2365324 4.1008044  O -4.1999678 -3.0384634 1.2710044  C -3.0133318 1.0953466 -1.5267596  O -1.9984148 1.3096136 -2.2839156  C -4.2319928 1.8645076 -1.9222336  H -4.7698958 2.2397816 -1.0489906  H -4.9097818 1.1853666 -2.4542546  H -3.9460398 2.6934686 -2.5700106  C -3.0373198 0.1466346 -0.4753476  C -4.1989938 0.1086856 0.5125664  C -5.4860478 -0.3866754 -0.1315686  C -5.4930408 -1.4956064 -0.9817866  C -6.6863038 0.2888386 0.1019344  C -6.6772138 -1.9212344 -1.5751036  H -4.5680508 -2.0299424 -1.1817856  C -7.8723748 -0.1337464 -0.4917516  H -6.6873838 1.1624666 0.7515104  C -7.8693338 -1.2422374 -1.3336396  H -6.6666478 -2.7870694 -2.2296776  H -8.7948138 0.4066086 -0.3018026  H -8.7906718 -1.5736174 -1.8027686  C -2.1218268 -0.8527604 -0.5616036  C -1.2266058 -1.7402824 -0.6683826  C -1.1467778 -2.9566704 -1.4983366  C -1.7349718 -4.1535684 -1.0813676  C -0.4945788 -2.8878024 -2.7324526  C -1.6869248 -5.2693584 -1.9131446  H -2.2428738 -4.1970614 -0.1214496  C -0.4710318 -4.0022454 -3.5643056  H -0.0235878 -1.9557284 -3.0345736  C -1.0659348 -5.1950254 -3.1584546  H -2.1526118 -6.1962454 -1.5908546  H 0.0261522 -3.9364384 -4.5277136  H -1.0451528 -6.0647074 -3.8088836  O 2.5248322 -3.1237854 3.6081104  O 1.9880552 1.5976406 3.6713154  C 1.9469912 -4.4120914 3.6197614  H 1.1678872 -4.4869694 4.3877704  H 2.7571572 -5.1050244 3.8467594  H 1.5161322 -4.6696734 2.6445504  C 3.2986722 1.7051116 4.1837884  H 3.4969992 2.7719676 4.2953874  H 4.0289162 1.2634856 3.4960454  H 3.3796002 1.2116766 5.1606974  H -1.0780168 0.9370606 -1.9880506  H -0.1115418 -1.2774614 -0.0729416  H -4.3635728 1.1401376 0.8533624  O 0.3886042 0.7573866 -1.8036226  P 1.2930672 0.4362366 -0.6589366  O 1.5251742 1.7052626 0.3331434  O 2.7709432 0.1832676 -1.2876216  O 0.9388662 -0.7027564 0.2861364  C 1.8730892 2.9065946 -0.2677276  C 3.7551422 -0.1177514 -0.3547376  C 3.2113532 3.1425346 -0.5512886  C 0.8850242 3.8547186 -0.5907746  C 4.4686952 0.9225596 0.2196694  C 3.9729382 -1.4475494 0.0299624  C 4.4595782 2.4019846 -0.1066516  C 3.5796612 4.2718156 -1.2868266  C 1.3111692 5.0319226 -1.2259096  C -0.5375538 3.7049586 -0.1737766  C 5.3771632 0.6513296 1.2474674  C 4.8883442 -1.6881744 1.0605794  C 3.2651342 -2.5933224 -0.6045986  C 5.4319152 2.7765416 -1.2576056  C 4.9847422 2.9894266 1.2363614  C 2.6325732 5.2303906 -1.6138686  C 5.0562952 4.2296426 -1.6109436  H 0.5781102 5.8062436 -1.4259666  C -1.6158568 4.2299086 -0.9702026  C -0.8275308 3.1869826 1.0730284  C 5.5825102 -0.6510314 1.6823414  C 5.9827522 1.9406166 1.7552534  H 5.0416892 -2.7151264 1.3790714  C 3.4304282 -2.8976004 -1.9936216  C 2.4971842 -3.4182204 0.1851934  H 5.2284332 2.1220776 -2.1125846  H 6.4816922 2.6524916 -0.9733876  H 4.1364792 3.0704316 1.9255534  H 5.4183722 3.9863276 1.1124874  H 2.9126092 6.1298706 -2.1547946  H 5.2571002 4.4749356 -2.6581306  H 5.6171072 4.9482036 -0.9999026  C -1.4756208 4.5979776 -2.3377636  C -2.9042148 4.3725676 -0.3756866  C -2.1373918 3.2079026 1.6021144  H -0.0182588 2.7997496 1.6823694  H 6.2742672 -0.8639904 2.4931824  H 6.1020732 1.9616286 2.8436154  H 6.9797132 2.0957316 1.3227624  C 4.2081312 -2.0970304 -2.8742026  C 2.8108372 -4.0696524 -2.5151706  C 1.8719562 -4.5698154 -0.3421026  H 2.3691242 -3.1535484 1.2294834  C -2.5068328 5.1860746 -3.0266836  H -0.5413428 4.3823926 -2.8447816  C -3.9393278 5.0246206 -1.0989586  C -3.1451878 3.8318646 0.9145444  H -2.3260088 2.7589396 2.5721664  C 4.3419252 -2.4287454 -4.1977566  H 4.7019482 -1.2116454 -2.4871076  C 2.9710552 -4.3867324 -3.8900616  C 2.0380722 -4.8974974 -1.6608006  H 1.2511172 -5.1906584 0.2979264  C -3.7438668 5.4437606 -2.3881726  H -2.3745558 5.4507096 -4.0712106  H -4.9013248 5.1638396 -0.6109786  H -4.1413808 3.9231406 1.3430624  C 3.7132902 -3.5854434 -4.7170836  H 4.9368262 -1.8007064 -4.8538546  H 2.4881472 -5.2836174 -4.2715756  H 1.5595012 -5.7784444 -2.0791886  H -4.5422988 5.9393486 -2.9319546  H 3.8273332 -3.8360934 -5.7674166 |
| **TS3-C-*RSS***  C -4.3636895 0.9452154 0.6964523  C -5.6910815 0.8940534 1.4894253  C -5.7827585 1.9268914 2.6092113  H -5.7831725 -0.1181516 1.9028343  H -6.5102705 1.0408514 0.7796843  H -6.7424935 1.8325584 3.1235973  H -4.9869645 1.7888494 3.3454023  H -5.7198825 2.9485774 2.2187983  C -2.7914665 -1.3316386 -2.0607027  O -1.6829035 -1.5478386 -2.6892257  C -3.8305575 -2.3617616 -2.3824167  H -4.4350305 -2.6065236 -1.5072767  H -4.5101085 -1.9678666 -3.1470347  H -3.3330145 -3.2581076 -2.7538477  C -3.0250605 -0.2559806 -1.1814887  C -4.3320815 -0.1828826 -0.3805807  C -5.5539265 -0.1501486 -1.2921227  C -5.5869675 0.6366414 -2.4454197  C -6.6545345 -0.9618906 -1.0049337  C -6.6967105 0.6151914 -3.2850517  H -4.7362415 1.2649114 -2.6891567  C -7.7664295 -0.9862526 -1.8414917  H -6.6337775 -1.5927046 -0.1176207  C -7.7891165 -0.1959676 -2.9874337  H -6.7055555 1.2340854 -4.1768877  H -8.6090915 -1.6277226 -1.6021617  H -8.6511445 -0.2151906 -3.6469347  C -2.0323905 0.6778014 -1.0447157  C -1.0382665 1.3788534 -0.7128517  C -0.3413335 2.6552574 -0.7375367  C 0.9392625 2.7307404 -0.1762147  C -0.9816775 3.8183204 -1.1835617  C 1.5750135 3.9609424 -0.0664497  H 1.4229055 1.8278774 0.1877293  C -0.3451395 5.0467234 -1.0516387  H -1.9880375 3.7543254 -1.5881127  C 0.9302905 5.1202164 -0.4913347  H 2.5799925 4.0105274 0.3454383  H -0.8505885 5.9509574 -1.3771507  H 1.4240915 6.0827264 -0.3890527  H -4.3914695 -1.1023996 0.2157633  H -0.8248285 -1.0762486 -2.3821867  H -0.4834645 0.4817024 0.0128143  N -3.2372955 0.8397514 1.6268803  C -4.2341375 2.3600304 0.1304923  O -3.2374825 2.9650564 0.8483353  C -2.6843745 1.9857334 1.6564343  O -4.8587695 2.9378114 -0.7091947  C -1.4576575 2.4123794 2.3428093  C -1.1546485 3.7658524 2.3890843  C -0.5715425 1.4342904 2.8142773  C 0.0779325 4.1651854 2.9147143  H -1.8215555 4.5144294 1.9799473  C 0.6469195 1.8541934 3.3385663  H -0.8225615 0.3879424 2.6919123  C 0.9730095 3.2183794 3.3939603  H 1.9449765 3.4824244 3.7923693  O 0.3208315 5.5002944 2.8901713  O 1.6062465 1.0284804 3.8142543  C 1.6165885 5.9278334 3.2434973  H 2.3736475 5.4605074 2.6009753  H 1.6296625 7.0081864 3.0979473  H 1.8433235 5.7005754 4.2923403  C 1.3939025 -0.3670576 3.6913013  H 2.2977455 -0.8412966 4.0736823  H 0.5358435 -0.6870036 4.2984323  H 1.2341805 -0.6543016 2.6458343  O 0.6633185 -0.8898606 -2.0198197  P 1.1489385 -1.0581996 -0.6164307  O 2.6147385 -0.3906706 -0.3778827  O 1.4095925 -2.6482756 -0.3881317  O 0.3064745 -0.5037396 0.5218953  C 3.6513335 -0.7930476 -1.2067067  C 1.7865145 -3.0253606 0.8896883  C 4.3684965 -1.9387066 -0.8839557  C 3.9623105 -0.0454276 -2.3491847  C 3.1286655 -3.0017986 1.2332813  C 0.8029855 -3.3751386 1.8246723  C 4.3583955 -2.7944386 0.3716693  C 5.3342305 -2.4162916 -1.7745057  C 4.9755905 -0.5178666 -3.1896357  C 3.2911765 1.2512304 -2.6696517  C 3.5018475 -3.2399166 2.5597703  C 1.2113535 -3.6631796 3.1314113  C -0.6428355 -3.4073536 1.4717153  C 4.8785115 -4.1417226 -0.2056057  C 5.3542665 -2.2587866 1.4400713  C 5.6440285 -1.7117216 -2.9296417  C 5.8985725 -3.7297566 -1.2822357  H 5.2191885 0.0628044 -4.0746537  C 3.9002605 2.4770644 -2.2488967  C 2.1464745 1.2857364 -3.4301047  C 2.5506205 -3.5805456 3.5116933  C 4.9891945 -3.0241306 2.7267443  H 0.4525925 -3.9380786 3.8590133  C -1.1537225 -4.3285896 0.5012283  C -1.5092805 -2.5677676 2.1336473  H 4.0377785 -4.6648546 -0.6751267  H 5.2956355 -4.7932706 0.5686493  H 5.1662295 -1.1886296 1.5822613  H 6.3976215 -2.3855696 1.1351633  H 6.3941065 -2.0830936 -3.6221077  H 5.9939885 -4.4702006 -2.0818877  H 6.8979545 -3.5900346 -0.8505227  C 5.0560525 2.5114324 -1.4214047  C 3.3250765 3.7113654 -2.6634907  C 1.5551335 2.5173814 -3.8008837  H 1.6856335 0.3518554 -3.7296237  H 2.8385385 -3.7692806 4.5424443  H 5.2307025 -2.4651386 3.6358353  H 5.5207375 -3.9823276 2.7861023  C -0.3213605 -5.2262166 -0.2204897  C -2.5587905 -4.3710816 0.2679723  C -2.9004685 -2.6036456 1.8931183  H -1.1082935 -1.8564496 2.8499203  C 5.6110925 3.7052064 -1.0340557  H 5.4937525 1.5733794 -1.0931767  C 3.9325685 4.9289964 -2.2601277  C 2.1362695 3.7023594 -3.4383447  H 0.6376255 2.5122704 -4.3812617  C -0.8519045 -6.1044146 -1.1295997  H 0.7490005 -5.2113436 -0.0429017  C -3.0782485 -5.2984466 -0.6747977  C -3.4140575 -3.4954836 0.9877383  H -3.5450655 -1.9103886 2.4252443  C 5.0480555 4.9313204 -1.4633297  H 6.4912065 3.7117644 -0.3981077  H 3.4841385 5.8636784 -2.5891527  H 1.6892205 4.6524384 -3.7201137  C -2.2471375 -6.1429606 -1.3627537  H -0.1986445 -6.7780156 -1.6753927  H -4.1534985 -5.3246606 -0.8381967  H -4.4870285 -3.5520326 0.8091213  H 5.5020805 5.8694064 -1.1579857  H -2.6546515 -6.8469836 -2.0817687 | **TS3-C-*RSR***  C 0.5239430 2.9014390 2.4446999  O -0.7102590 2.6163650 2.6864009  C 0.9418420 4.2606420 2.9153279  H 1.1658640 4.8925890 2.0481449  H 1.8479990 4.1866920 3.5237599  H 0.1347290 4.7115960 3.4894179  C 1.4519140 2.0279940 1.8456069  C 1.1137960 0.7088400 1.8510289  C 0.6915960 -0.4781420 1.8249769  C 0.6962420 -1.5849160 2.7900179  C 1.9076410 -2.1254200 3.2324969  C -0.5141510 -2.0485160 3.3084119  C 1.9036130 -3.1215600 4.2025259  H 2.8362540 -1.7564070 2.8035389  C -0.5058700 -3.0292310 4.2952849  H -1.4476370 -1.6224320 2.9488819  C 0.6978550 -3.5703200 4.7404789  H 2.8442510 -3.5417390 4.5472139  H -1.4480030 -3.3917700 4.6951339  H 0.6971010 -4.3425020 5.5047079  C 2.7754450 2.5396750 1.2970259  C 3.9613720 1.5678500 1.6049509  C 2.6877730 2.8567170 -0.1913721  C 4.1627060 1.3688010 3.1118689  C 3.3673060 3.9676510 -0.7009321  C 1.9942680 2.0231130 -1.0738021  C 5.3637410 0.4867710 3.4412969  H 3.2474150 0.9240610 3.5163999  H 4.2853690 2.3602450 3.5649999  C 3.3771400 4.2275670 -2.0688581  H 3.9141920 4.6188690 -0.0227541  C 1.9979240 2.2890100 -2.4400641  H 1.4415930 1.1636330 -0.7028791  H 5.4511490 0.3461050 4.5216229  H 5.2597030 -0.4989070 2.9778849  H 6.2976320 0.9380580 3.0884339  C 2.6944280 3.3855350 -2.9425031  H 3.9174560 5.0898790 -2.4487021  H 1.4503120 1.6321970 -3.1089511  H 2.6997990 3.5868780 -4.0099091  H 3.0456800 3.4665560 1.8154049  H 0.0163650 -0.4811120 0.6911409  N 3.7840520 0.3068790 0.9078509  C 5.2238340 2.1540720 0.9691649  C 4.6692710 0.2571910 0.0001309  O 5.8342810 3.1441550 1.2374659  O 5.5833470 1.2945820 -0.0451021  C 4.7963570 -0.7906700 -1.0185831  C 5.8943260 -0.8111170 -1.8875051  C 3.7808670 -1.7315620 -1.0905881  C 5.9533590 -1.8204530 -2.8429831  H 6.6590040 -0.0505000 -1.8019031  C 3.8606160 -2.7361840 -2.0594771  H 2.9282840 -1.6839120 -0.4193601  C 4.9407150 -2.7867610 -2.9335761  H 5.0378890 -3.5474070 -3.6985381  O 6.9573140 -1.9592000 -3.7445361  O 2.8283700 -3.6154930 -2.0679051  C 2.8320290 -4.6383100 -3.0402621  H 1.9194330 -5.2104330 -2.8718721  H 2.8246960 -4.2199870 -4.0539011  H 3.7033980 -5.2938910 -2.9239631  C 7.9907830 -0.9971470 -3.7187081  H 8.5187770 -1.0072710 -2.7577421  H 8.6806540 -1.2745460 -4.5154481  H 7.5998000 0.0098540 -3.9060981  O -2.1586230 0.7077810 1.6717279  P -2.1255900 0.1441700 0.2868759  O -2.7798790 1.1513150 -0.8100001  O -3.1166090 -1.1536990 0.2385619  O -0.7774900 -0.2446670 -0.3102681  C -4.0772670 1.5927900 -0.5875631  C -3.1222330 -1.8249780 -0.9826411  C -5.1305350 0.8090890 -1.0415221  C -4.3222720 2.8025620 0.0811169  C -3.9831350 -1.4022100 -1.9863331  C -2.2293080 -2.8809360 -1.2090171  C -5.1246100 -0.4086390 -1.9427801  C -6.4471420 1.1566440 -0.7304251  C -5.6607680 3.1543010 0.3161269  C -3.2694660 3.7260260 0.5874199  C -3.8909690 -1.9637900 -3.2642971  C -2.1879760 -3.4482640 -2.4873321  C -1.3373240 -3.4594040 -0.1655641  C -6.4577040 -1.0838730 -1.5159661  C -5.1974830 0.0084600 -3.4397411  C -6.7211300 2.3352430 -0.0533701  C -7.4035960 0.0929800 -1.2175581  H -5.8580880 4.0985520 0.8140269  C -2.1965120 4.2168090 -0.2288171  C -3.4130920 4.2117950 1.8716359  C -2.9909380 -2.9847610 -3.5253581  C -4.8434270 -1.2733740 -4.2126791  H -1.4911900 -4.2638260 -2.6578781  C -1.8846110 -4.2225130 0.9152779  C 0.0280560 -3.4113020 -0.3430951  H -6.2771200 -1.6547250 -0.5979991  H -6.8381370 -1.7726090 -2.2765821  H -4.4331500 0.7721930 -3.6227351  H -6.1710780 0.4306080 -3.7078101  H -7.7427980 2.6198310 0.1817829  H -8.1670750 -0.1527020 -0.4732831  H -7.9299410 0.4218780 -2.1227541  C -1.9942950 3.8181990 -1.5788821  C -1.3070790 5.1882560 0.3203299  C -2.5653640 5.2137240 2.3877919  H -4.2063240 3.8130030 2.4966119  H -2.9102550 -3.4167620 -4.5189201  H -4.3903950 -1.0714700 -5.1879201  H -5.7344240 -1.8889080 -4.3918551  C -3.2810700 -4.3014880 1.1719119  C -0.9985860 -4.9583790 1.7522479  C 0.9044670 -4.1100140 0.5191279  H 0.4368380 -2.8320980 -1.1670571  C -0.9282040 4.2852910 -2.3031571  H -2.6914140 3.1288080 -2.0384411  C -0.2106400 5.6508020 -0.4560681  C -1.5394250 5.6986630 1.6228409  H -2.7324000 5.5908260 3.3924559  C -3.7666950 -5.0639490 2.2028869  H -3.9659590 -3.7462160 0.5389079  C -1.5342960 -5.7503820 2.8020699  C 0.4007440 -4.8775440 1.5355939  H 1.9751010 -4.0420660 0.3517649  C -0.0127540 5.2005470 -1.7337321  H -0.7820910 3.9496290 -3.3254351  H 0.4651050 6.3828470 -0.0179141  H -0.8817140 6.4785530 2.0004089  C -2.8845780 -5.8036230 3.0272369  H -4.8359030 -5.1064120 2.3869739  H -0.8439740 -6.3044570 3.4341559  H 1.0636550 -5.4246790 2.2010669  H 0.8368460 5.5463940 -2.3128961  H -3.2824960 -6.4103780 3.8352609  H -1.1111560 1.7722890 2.2665289 |
| **INT4-C-*SRS***  C -1.0024433 1.9919433 2.8533907  O 0.1383297 1.5630043 2.8242877  C -1.5313423 2.6168463 4.1237207  H -1.8083263 3.6617243 3.9526457  H -2.4274433 2.0859733 4.4630367  H -0.7569263 2.5616053 4.8887447  C -1.9358513 1.8726903 1.6817307  C -1.5939363 1.0016493 0.7561787  C -1.1806873 0.0814843 -0.0768473  C -1.4484693 -1.3644447 0.0640397  C -0.8461213 -2.2471747 -0.8366613  C -2.2952463 -1.8654347 1.0613447  C -1.1052353 -3.6128077 -0.7533623  H -0.1657083 -1.8497577 -1.5835753  C -2.5570543 -3.2262417 1.1356427  H -2.7792353 -1.1748117 1.7487957  C -1.9627313 -4.1042737 0.2267287  H -0.6303043 -4.2932497 -1.4545593  H -3.2375833 -3.6034017 1.8944177  H -2.1664293 -5.1706097 0.2914277  H 1.3675227 0.9896063 1.6999787  H -0.5570683 0.3872353 -0.9173613  C -3.1767153 2.7554803 1.6428327  C -4.2245313 2.3382273 0.5743167  C -2.7714443 4.2219183 1.5376567  C -3.7188383 2.3460283 -0.8688583  N -4.6510123 0.9571683 0.7827347  C -5.4546513 3.2746003 0.6241047  C -1.7542503 4.6309393 0.6711927  C -3.4094243 5.1818723 2.3291057  O -3.9905823 1.1010583 -1.3782843  O -3.2805323 3.2335023 -1.5393903  C -4.5136183 0.3632323 -0.3333143  C -6.5734063 2.8744193 -0.3342493  H -5.8249363 3.2714483 1.6561107  H -5.1087103 4.2866903 0.3942947  C -1.4000733 5.9745393 0.5883577  H -1.2374173 3.8984163 0.0572317  C -3.0535823 6.5250603 2.2491387  H -4.1923003 4.8698143 3.0179187  C -4.8170403 -1.0382167 -0.6441093  H -7.4071163 3.5756403 -0.2472453  H -6.9483413 1.8718853 -0.1122043  H -6.2358603 2.8953163 -1.3762143  C -2.0468443 6.9246583 1.3740927  H -0.6095723 6.2778003 -0.0911763  H -3.5582263 7.2558973 2.8734947  C -5.6634993 -1.7508447 0.2003477  C -4.1697303 -1.6430087 -1.7160323  H -1.7631573 7.9705483 1.3105317  C -5.8601163 -3.1076147 -0.0423243  H -6.1471403 -1.2727447 1.0442377  C -4.3515723 -3.0075057 -1.9243163  H -3.4718003 -1.0947467 -2.3380963  C -5.2088023 -3.7473217 -1.1028893  O -6.6958283 -3.7473837 0.8183267  O -3.6456083 -3.5429577 -2.9516313  H -5.3489433 -4.8057637 -1.2740203  C -6.8850593 -5.1329427 0.6452367  C -3.6293263 -4.9465997 -3.0733403  H -7.5658183 -5.4464427 1.4364177  H -5.9386513 -5.6800187 0.7416097  H -7.3342613 -5.3577877 -0.3299053  H -3.2780563 -5.4154857 -2.1454413  H -2.9342333 -5.1725277 -3.8820173  H -4.6205193 -5.3410127 -3.3297273  H -3.7138523 2.6248703 2.5920107  O 1.5673177 -0.2258057 -0.8342413  P 2.6401697 0.2837633 0.0280337  O 3.6222097 1.4005053 -0.6141123  O 3.6612647 -0.9207697 0.3760607  O 2.2977587 1.0264823 1.3655597  C 4.1702267 1.0189073 -1.8327673  C 4.7129617 -0.6569647 1.2389697  C 5.3881667 0.3562063 -1.8384133  C 3.4430117 1.2340623 -3.0122503  C 5.8997597 -0.1525477 0.7305727  C 4.5279197 -0.8627547 2.6144267  C 6.3549117 0.0584313 -0.7044433  C 5.8799187 -0.1510867 -3.0442743  C 3.9901397 0.7627173 -4.2102643  C 2.1286767 1.9356283 -2.9638623  C 6.9066387 0.2372093 1.6194407  C 5.5757607 -0.5134867 3.4715837  C 3.2130877 -1.3260287 3.1368917  C 7.0669347 -1.2016257 -1.2761713  C 7.3915987 1.1993043 -0.4992273  C 5.1943757 0.0607653 -4.2337163  C 7.1386567 -0.9508177 -2.7946573  H 3.4366077 0.9270843 -5.1306463  C 0.9172787 1.2814993 -3.3604303  C 2.0775337 3.2260313 -2.4876343  C 6.7556177 0.0500913 2.9869797  C 8.0423767 0.8889003 0.8627707  H 5.4415187 -0.6660787 4.5386217  C 2.6010187 -2.5383367 2.6790697  C 2.5390607 -0.5222467 4.0293367  H 6.4375687 -2.0763597 -1.0780893  H 8.0435807 -1.3719377 -0.8127253  H 6.8540227 2.1519403 -0.4414023  H 8.1083687 1.2610523 -1.3239273  H 5.5778907 -0.3332777 -5.1706053  H 7.1691297 -1.8765597 -3.3765873  H 8.0324307 -0.3718937 -3.0602433  C 0.8794437 -0.0747977 -3.7868953  C -0.3125453 1.9947633 -3.2642933  C 0.8553207 3.9300313 -2.3982273  H 2.9978637 3.7060673 -2.1676843  H 7.5384097 0.3537223 3.6762757  H 8.4200567 1.7839513 1.3656157  H 8.8887867 0.1992003 0.7514027  C 3.2563467 -3.4514987 1.8099967  C 1.2785857 -2.8450317 3.1096777  C 1.2380477 -0.8468337 4.4763007  H 2.9928827 0.4145303 4.3398707  C -0.3065093 -0.6726637 -4.1287683  H 1.8017527 -0.6454237 -3.8027373  C -1.5269633 1.3483583 -3.6270813  C -0.3165493 3.3309393 -2.7803553  H 0.8551577 4.9506913 -2.0268703  C 2.6231947 -4.5846387 1.3697097  H 4.2732877 -3.2400407 1.4947737  C 0.6443347 -4.0190497 2.6250057  C 0.6138257 -1.9728337 4.0106497  H 0.7270897 -0.1726227 5.1561417  C -1.5246153 0.0465263 -4.0567923  H -0.3184803 -1.7132667 -4.4399543  H -2.4540953 1.9068073 -3.5362553  H -1.2658253 3.8535163 -2.7059583  C 1.2958937 -4.8674317 1.7701597  H 3.1406787 -5.2681397 0.7035227  H -0.3757873 -4.2235327 2.9386267  H -0.4003583 -2.2169867 4.3166707  H -2.4538013 -0.4411257 -4.3378513  H 0.7963957 -5.7568517 1.3974427 | **Pro-*SRS***  C -3.0158149 2.0265678 1.7159547  O -3.7153349 1.4931078 2.5488797  C -2.7368699 3.5119248 1.7246877  H -1.6593409 3.7006508 1.7597427  H -3.1165499 3.9687698 0.8054787  H -3.2248349 3.9591678 2.5905157  C -2.3770759 1.1938158 0.6340967  C -1.5011929 1.7360138 -0.1776943  C -0.5823849 2.2680748 -0.9475213  C 0.8516581 2.3354568 -0.6038483  C 1.7761631 2.7269878 -1.5770853  C 1.3090021 2.0121538 0.6794537  C 3.1333201 2.7883968 -1.2776013  H 1.4261211 2.9753338 -2.5757633  C 2.6635411 2.0730768 0.9774137  H 0.5947801 1.6860148 1.4325367  C 3.5787081 2.4644838 0.0011827  H 3.8429661 3.0808188 -2.0454503  H 3.0074961 1.8010638 1.9708537  H 4.6384871 2.5073188 0.2367147  H -0.8831189 2.6708438 -1.9146733  C -2.8242599 -0.2567452 0.5911067  C -1.8605569 -1.2003752 -0.1723263  C -4.2768869 -0.3406482 0.1443837  C -1.4903039 -0.7816612 -1.5983863  N -0.5707559 -1.2471712 0.5133427  C -2.4598749 -2.6233912 -0.2547843  C -4.7149579 0.2620448 -1.0366173  C -5.2004479 -1.0263952 0.9355817  O -0.1107629 -0.7732912 -1.6498473  O -2.1630199 -0.5742402 -2.5621643  C 0.3215631 -1.0342322 -0.3660833  C -1.5158689 -3.6591042 -0.8588733  H -2.7432059 -2.9155722 0.7632167  H -3.3777839 -2.5551002 -0.8468323  C -6.0467539 0.1575618 -1.4278773  H -4.0095579 0.8047468 -1.6574153  C -6.5321439 -1.1307092 0.5467847  H -4.8708419 -1.4678402 1.8727767  C 1.7783181 -1.0396372 -0.1676253  H -2.0063539 -4.6352542 -0.8950213  H -0.6033489 -3.7591582 -0.2650633  H -1.2333039 -3.3968072 -1.8846233  C -6.9582099 -0.5401312 -0.6398233  H -6.3726519 0.6253378 -2.3520663  H -7.2377369 -1.6648592 1.1759367  C 2.2766901 -1.4179792 1.0777167  C 2.6193591 -0.6346312 -1.1972373  H -7.9973469 -0.6163752 -0.9454643  C 3.6511801 -1.3869972 1.2873427  H 1.6100931 -1.7253782 1.8750277  C 3.9929931 -0.5947772 -0.9649653  H 2.2329271 -0.3192412 -2.1585963  C 4.5225551 -0.9744612 0.2708887  O 4.0705781 -1.7671982 2.5226657  O 4.7486511 -0.1506462 -2.0022803  H 5.5894831 -0.9431402 0.4434017  C 5.4556421 -1.7682812 2.7822127  C 6.1368091 -0.0231132 -1.7990493  H 5.5697981 -2.1156552 3.8089507  H 5.8825311 -0.7615542 2.6906797  H 5.9890671 -2.4491932 2.1073467  H 6.3568331 0.6769458 -0.9825223  H 6.5447881 0.3675728 -2.7311353  H 6.6016961 -0.9920172 -1.5784023  H -2.7843439 -0.6112862 1.6278447 |
|  | **(*R*)-C1**  O 0.2764857 -1.8943738 1.3323349  P -0.0357733 -1.0461668 0.1826099  O 1.1287417 -0.1006728 -0.4132291  O -1.2295453 -0.0097838 0.5540109  O -0.4536203 -1.7237068 -1.1962141  C 1.7068087 0.7390342 0.5405259  C -1.7130773 0.8218782 -0.4464011  C 1.1318917 1.9767772 0.7867559  C 2.8087457 0.2851352 1.2758239  C -1.0829173 2.0312662 -0.6938211  C -2.8066373 0.4013952 -1.2176681  C 0.0396727 2.7299292 0.0500119  C 1.6041797 2.7459032 1.8547459  C 3.2885357 1.1010752 2.3050939  C 3.4312867 -1.0407388 1.0131639  C -1.5019403 2.8110102 -1.7764101  C -3.2320493 1.2262882 -2.2636541  C -3.4487163 -0.9248358 -1.0020471  C -0.4960413 3.6370492 1.1937819  C 0.6248227 3.6110132 -1.0915311  C 2.6812337 2.3158652 2.6161059  C 0.7540247 3.9837902 2.0239759  H 4.1413377 0.7527562 2.8803489  C 4.0560157 -1.3341508 -0.2407551  C 3.4231287 -1.9877318 2.0109939  C -2.5793813 2.4208132 -2.5589071  C -0.5994363 4.0124112 -1.9343331  H -4.0794703 0.9048342 -2.8622601  C -4.0524413 -1.2857248 0.2459829  C -3.4672983 -1.8239398 -2.0501771  H -1.1930063 3.0519312 1.8042709  H -1.0281693 4.5142562 0.8127579  H 1.3009087 2.9952302 -1.6951291  H 1.1920757 4.4641072 -0.7068981  H 3.0425797 2.9112912 3.4498309  H 0.5225857 4.1914802 3.0727039  H 1.2639867 4.8694602 1.6239639  C 4.1211107 -0.3984638 -1.3079701  C 4.6567947 -2.6130228 -0.4231931  C 4.0198967 -3.2546468 1.8237719  H 2.9120877 -1.7643158 2.9424999  H -2.9020513 3.0276932 -3.3999551  H -0.3496553 4.2135892 -2.9800981  H -1.0775653 4.9164152 -1.5359791  C -4.1132463 -0.3982018 1.3538249  C -4.6381953 -2.5783798 0.3773609  C -4.0503213 -3.1056728 -1.9136261  H -2.9992213 -1.5417668 -2.9900571  C 4.7362957 -0.7189348 -2.4908781  H 3.6792837 0.5844472 -1.1758781  C 5.2849867 -2.9144778 -1.6602051  C 4.6225897 -3.5601758 0.6328469  H 3.9844147 -3.9845428 2.6263479  C -4.7079603 -0.7800598 2.5279859  H -3.6837203 0.5936002 1.2589609  C -5.2412653 -2.9454118 1.6095459  C -4.6173623 -3.4750838 -0.7222101  H -4.0385243 -3.7905988 -2.7555241  C 5.3242467 -1.9931388 -2.6732931  H 4.7757987 0.0098122 -3.2948341  H 5.7366307 -3.8953928 -1.7858121  H 5.0806857 -4.5333038 0.4750179  C -5.2751883 -2.0699178 2.6618909  H -4.7441003 -0.0895348 3.3646339  H -5.6781153 -3.9366068 1.6985349  H -5.0626353 -4.4590108 -0.6007691  H 5.8075317 -2.2342828 -3.6151341  H -5.7381573 -2.3602168 3.5998919  H -1.2445943 -2.2845508 -1.1080571 |
| **INT0-B**  C 2.9614802 2.7672981 0.3448840  O 3.7851092 3.6491231 -0.2996680  C 2.9645802 4.4844891 -1.0463210  C 1.5396162 4.0479781 -0.7556510  H 1.0893142 3.6309141 -1.6679110  C 3.6075932 1.6919041 1.1046900  C 2.8425012 0.5828921 1.4570090  C 4.9678732 1.7667531 1.3906660  C 3.4640592 -0.4772299 2.1104980  H 1.7942792 0.5023531 1.1931590  C 5.5681222 0.7035381 2.0621240  H 5.5671792 2.6183481 1.0911510  C 4.8275102 -0.4241829 2.4254300  H 5.3065182 -1.2566519 2.9223120  O 3.4088972 5.3438141 -1.7427030  N 1.7098862 2.9538171 0.1906570  C 0.6459222 5.1680201 -0.2128470  C 0.3530762 6.2364801 -1.2620400  H 1.1204332 5.6075741 0.6725030  H -0.2839618 4.6937901 0.1202540  H -0.2930478 7.0128961 -0.8443120  H 1.2731632 6.7064231 -1.6177810  H -0.1600418 5.7969511 -2.1230710  O 6.9008952 0.8367111 2.2966670  O 2.6716062 -1.5403089 2.3915150  C 7.5777882 -0.2480689 2.8904370  H 8.6234872 0.0507101 2.9607520  H 7.1917402 -0.4587839 3.8952990  H 7.4976022 -1.1520559 2.2732820  C 3.2779892 -2.6914989 2.9308580  H 3.7033872 -2.4960969 3.9233610  H 2.4863052 -3.4357029 3.0199680  H 4.0641262 -3.0723669 2.2666750  H 0.2310792 2.3038691 0.7595390  C -1.3738628 -2.0714859 -0.8535880  C -0.1229108 -1.4543179 -0.9308600  C 0.9539922 -2.0481609 -1.6024620  C 0.6703022 -3.1875739 -2.3499150  C -0.6183408 -3.7158869 -2.4640060  C -1.6529428 -3.1652999 -1.7004120  H 1.4883592 -3.6671449 -2.8834810  C -2.3432898 -1.6154259 0.1907660  C -2.7593918 -2.4752989 1.2327840  C -2.7734138 -0.2935369 0.2031770  C -3.6775228 -2.0004729 2.1794390  C -3.6788638 0.2093641 1.1389750  C -4.1307598 -0.6812689 2.1063120  H -4.8441328 -0.3221799 2.8450800  C -0.8336438 -4.9169259 -3.3659140  C -2.3036538 -5.1674269 -3.6923650  C -3.1361478 -5.0979879 -2.4148890  C -3.0720088 -3.6907999 -1.8264690  H -0.2515548 -4.7875399 -4.2851480  H -2.6628338 -4.4053049 -4.3960380  H -2.4160858 -6.1388469 -4.1844620  H -4.1787468 -5.3686749 -2.6103660  H -2.7458148 -5.8226349 -1.6863340  H -3.6229928 -3.0026469 -2.4840470  H -3.5888408 -3.6517689 -0.8639200  H -0.4232028 -5.8062929 -2.8666750  C -2.1296868 -3.8488659 1.3985800  C -2.9331058 -4.7797389 2.3037470  C -3.3036128 -4.0690239 3.6018170  C -4.2123008 -2.8837589 3.2903200  H -1.9577358 -4.3247259 0.4311130  H -3.8511888 -5.0937089 1.7877580  H -2.3529358 -5.6866459 2.5037440  H -3.8026508 -4.7517819 4.2972280  H -2.3881218 -3.7154489 4.0942070  H -5.1994268 -3.2635019 2.9904460  H -4.3782368 -2.2760039 4.1865640  H -1.1295108 -3.6975659 1.8313790  O 0.0824742 -0.2763319 -0.2315620  O -0.6714998 1.8557031 0.7324150  O -0.3305408 1.7756481 -1.8493750  C 2.3763512 -1.6379659 -1.4016790  C 2.9363202 -0.4192549 -1.8973250  C 3.1946422 -2.5210429 -0.7357130  C 2.1600542 0.4998901 -2.6431680  C 4.3157832 -0.1318729 -1.6671350  C 4.5654832 -2.2445649 -0.5352310  H 2.7748472 -3.4486539 -0.3551570  C 2.7011832 1.6760151 -3.1016590  H 1.1207502 0.2734321 -2.8470050  C 4.8428312 1.0936621 -2.1517690  C 5.1379802 -1.0773009 -0.9761560  H 5.1832912 -2.9784679 -0.0212650  C 4.0573012 1.9797551 -2.8483060  H 2.0807172 2.3658561 -3.6667200  H 5.8834502 1.3359251 -1.9600940  H 4.4758912 2.9196051 -3.1976970  C -4.0881678 1.6385451 1.1599280  C -4.7882768 2.2464301 0.0684210  C -3.8042558 2.3885441 2.2752230  C -5.1476628 1.5156191 -1.0944510  C -5.1587038 3.6202551 0.1570800  C -4.1759638 3.7484681 2.3574860  H -3.2591878 1.9300421 3.0954020  C -5.8227988 2.1134481 -2.1272980  H -4.8820888 0.4651561 -1.1577480  C -5.8544758 4.2085391 -0.9328450  C -4.8359018 4.3746861 1.3300290  H -3.9241368 4.3110191 3.2526850  C -6.1766418 3.4782271 -2.0481840  H -6.0856278 1.5375071 -3.0090910  H -6.1367148 5.2551131 -0.8786640  H -6.7069498 3.9479841 -2.8709670  C 6.6072862 -0.8185859 -0.7710330  H 7.1274592 -0.7081119 -1.7293340  H 6.7877162 0.0955791 -0.1945400  H 7.0685612 -1.6535589 -0.2353200  C -5.2153378 5.8284871 1.4353500  H -4.7545488 6.4212441 0.6373620  H -6.2995018 5.9675451 1.3592000  H -4.8888218 6.2411361 2.3928150  P -0.7517768 1.0710701 -0.6298990  O -2.2700408 0.5681911 -0.7604740 | **TS1-B**  C 3.1516623 2.4173482 0.0726449  O 3.7210123 2.7266412 -1.0748561  C 2.7415823 3.4471172 -1.8843851  C 1.5159603 3.3983102 -1.1169101  H 0.7308683 2.2514322 -1.4797261  C 3.8408533 1.6007242 1.0665599  C 3.0893533 0.7812322 1.9064009  C 5.2310673 1.6544342 1.1269149  C 3.7675323 -0.0052088 2.8369469  H 2.0089923 0.6914792 1.8235819  C 5.8852323 0.8758342 2.0768719  H 5.8043963 2.2781802 0.4503379  C 5.1621933 0.0438482 2.9381639  H 5.6774223 -0.5691808 3.6648409  O 3.0726543 3.8822772 -2.9462091  N 1.9432383 2.8871862 0.1549679  C 0.5229543 4.5436812 -1.1327381  C -0.0873717 4.7207072 -2.5226481  H 0.9988023 5.4762932 -0.8022951  H -0.2647997 4.3052372 -0.4078841  H -0.7976617 5.5516252 -2.5252471  H 0.6888613 4.9231912 -3.2650181  H -0.6204777 3.8127002 -2.8205011  O 7.2384443 0.9727422 2.0850369  O 2.9921963 -0.8133748 3.5967419  C 7.9559493 0.1535672 2.9817859  H 9.0101243 0.3637392 2.8036279  H 7.7086423 0.3898502 4.0236999  H 7.7593043 -0.9091528 2.7930009  C 3.6289373 -1.6766428 4.5118039  H 4.1897003 -1.1155418 5.2693319  H 2.8324043 -2.2395058 4.9974769  H 4.3061263 -2.3709478 3.9982559  H 1.2418133 2.6361002 0.8731229  C -1.5177337 -2.0479008 -0.6587511  C -0.2268377 -1.5328608 -0.5229371  C 0.8525823 -2.0081598 -1.2645591  C 0.5754523 -2.9676428 -2.2342981  C -0.7195047 -3.4257138 -2.4847441  C -1.7823347 -2.9635108 -1.6953631  H 1.4009133 -3.3554998 -2.8283521  C -2.5681027 -1.5830048 0.2971809  C -3.2140807 -2.4644428 1.1878169  C -2.8506497 -0.2205748 0.3694749  C -4.1990907 -1.9604618 2.0494899  C -3.8223727 0.3085362 1.2215599  C -4.4938937 -0.5951378 2.0394639  H -5.2612107 -0.2130448 2.7097349  C -0.9256057 -4.4517798 -3.5833191  C -2.3748157 -4.5498918 -4.0531131  C -3.3056427 -4.6380448 -2.8467291  C -3.2124737 -3.3569478 -2.0219321  H -0.2627507 -4.2188898 -4.4240121  H -2.6350257 -3.6606508 -4.6417321  H -2.4956837 -5.4176158 -4.7098791  H -4.3417427 -4.7998478 -3.1615411  H -3.0185437 -5.4996268 -2.2276581  H -3.6586487 -2.5306228 -2.5946771  H -3.8145327 -3.4384898 -1.1132281  H -0.6073537 -5.4340108 -3.2059761  C -2.7673037 -3.9125508 1.2954279  C -3.8006377 -4.8101408 1.9715989  C -4.2664817 -4.1809518 3.2810279  C -4.9754087 -2.8604818 2.9923919  H -2.4959087 -4.3162758 0.3171969  H -4.6647877 -4.9443228 1.3060619  H -3.3701477 -5.8021958 2.1427259  H -4.9365767 -4.8536138 3.8264249  H -3.3949347 -4.0010008 3.9241669  H -5.9553237 -3.0748958 2.5426979  H -5.1791927 -2.3194358 3.9230439  H -1.8386997 -3.9267778 1.8848449  O -0.0203287 -0.4932118 0.3720019  O -0.3793287 1.8962722 1.0464409  O -0.0065667 1.3260742 -1.4635431  C 2.2630933 -1.6042508 -0.9860301  C 2.9878573 -0.7270178 -1.8531971  C 2.9169953 -2.1914908 0.0700829  C 2.3451263 -0.0271628 -2.9104921  C 4.3909043 -0.5512218 -1.6591081  C 4.3036693 -2.0013768 0.2666249  H 2.3650563 -2.8454958 0.7397539  C 3.0567043 0.8064122 -3.7367111  H 1.2733053 -0.1420468 -3.0341531  C 5.0974283 0.2992342 -2.5509421  C 5.0531813 -1.2292878 -0.5860091  H 4.7935363 -2.5051468 1.0973179  C 4.4494263 0.9656432 -3.5597771  H 2.5486983 1.3594602 -4.5204141  H 6.1653543 0.4394812 -2.4149701  H 4.9985633 1.6394282 -4.2096861  C -4.0763517 1.7711512 1.3107669  C -4.5108547 2.5382272 0.1812569  C -3.8945527 2.4023812 2.5173339  C -4.7663397 1.9399572 -1.0807211  C -4.7156737 3.9413112 0.3274809  C -4.1104977 3.7907852 2.6583459  H -3.5502007 1.8246192 3.3703689  C -5.1791047 2.6900832 -2.1516361  H -4.6313397 0.8689512 -1.1915881  C -5.1377187 4.6895092 -0.8042221  C -4.5051517 4.5668232 1.5979809  H -3.9469347 4.2538872 3.6279169  C -5.3608787 4.0835792 -2.0143841  H -5.3645207 2.2126672 -3.1088341  H -5.2889187 5.7597012 -0.7049251  H -5.6812657 4.6736902 -2.8676851  C 6.5462333 -1.1249778 -0.4166351  H 7.0694133 -1.5436308 -1.2843141  H 6.8812323 -0.0892638 -0.2991511  H 6.8675553 -1.6813758 0.4693329  C -4.7169407 6.0481642 1.7686009  H -4.0583987 6.6266172 1.1112299  H -5.7473497 6.3364482 1.5324009  H -4.5110467 6.3482952 2.7988189  P -0.5963557 0.9597502 -0.0887531  O -2.1395987 0.6409072 -0.4493971 |
| **INT1-B**  C 3.2134322 2.4299102 0.1236851  O 3.7781092 2.7730752 -1.0128899  C 2.8170052 3.5561852 -1.7998609  C 1.6494202 3.5612312 -0.9998059  H 0.6523902 2.0510772 -1.4683409  C 3.8749342 1.5663082 1.0927391  C 3.1111652 0.7364102 1.9132821  C 5.2666302 1.5857562 1.1590981  C 3.7715802 -0.0862788 2.8234251  H 2.0297462 0.6684992 1.8304151  C 5.9045672 0.7655442 2.0841431  H 5.8528562 2.2206062 0.5041631  C 5.1670912 -0.0740478 2.9255551  H 5.6685682 -0.7173968 3.6353161  O 3.1539372 3.9833042 -2.8769409  N 2.0055672 2.9275502 0.2030241  C 0.4939502 4.5192682 -1.0820749  C -0.0475408 4.6302922 -2.5089539  H 0.7943592 5.5145942 -0.7256969  H -0.2969288 4.1700682 -0.4058789  H -0.8518978 5.3693462 -2.5565779  H 0.7472532 4.9272732 -3.1975659  H -0.4487018 3.6691562 -2.8457229  O 7.2610202 0.8326942 2.0916301  O 2.9769352 -0.8973948 3.5641631  C 7.9602792 -0.0253658 2.9647111  H 9.0190622 0.1657372 2.7915931  H 7.7189882 0.1878842 4.0131471  H 7.7402132 -1.0784328 2.7486261  C 3.5966192 -1.8065648 4.4446611  H 4.1711402 -1.2865048 5.2210321  H 2.7897652 -2.3704588 4.9123311  H 4.2583122 -2.4958258 3.9045591  H 1.3198412 2.7149322 0.9356001  C -1.5446218 -2.0319918 -0.6647439  C -0.2482168 -1.5310798 -0.5306219  C 0.8254732 -2.0029258 -1.2818699  C 0.5340522 -2.9502288 -2.2597459  C -0.7651788 -3.3964498 -2.5084209  C -1.8212758 -2.9344778 -1.7099019  H 1.3538132 -3.3368968 -2.8621029  C -2.5915838 -1.5671688 0.2949021  C -3.2500878 -2.4476778 1.1771501  C -2.8641028 -0.2047178 0.3771521  C -4.2333858 -1.9377448 2.0377231  C -3.8294538 0.3339022 1.2288811  C -4.5136678 -0.5690578 2.0372741  H -5.2782608 -0.1835948 2.7085321  C -0.9834438 -4.4097128 -3.6163229  C -2.4351468 -4.4932708 -4.0810189  C -3.3618388 -4.5854638 -2.8718829  C -3.2557818 -3.3127768 -2.0356619  H -0.3220608 -4.1731318 -4.4569919  H -2.6913338 -3.5974178 -4.6612749  H -2.5646708 -5.3543888 -4.7445839  H -4.4003638 -4.7359248 -3.1839929  H -3.0792978 -5.4547348 -2.2616489  H -3.6964298 -2.4775988 -2.5998269  H -3.8563698 -3.3980178 -1.1264289  H -0.6701318 -5.3972488 -3.2490849  C -2.8197458 -3.9010248 1.2763211  C -3.8681618 -4.7917048 1.9382241  C -4.3332578 -4.1680828 3.2506121  C -5.0236618 -2.8358488 2.9708461  H -2.5455468 -4.2994258 0.2966601  H -4.7298508 -4.9093318 1.2664671  H -3.4506858 -5.7902028 2.1031681  H -5.0152868 -4.8366668 3.7858861  H -3.4633348 -4.0056558 3.9004311  H -6.0046258 -3.0325528 2.5156621  H -5.2232838 -2.2998478 3.9052221  H -1.8958218 -3.9295948 1.8723941  O -0.0256558 -0.4992598 0.3801641  O -0.3627238 1.9024202 1.0767261  O -0.0392968 1.3161802 -1.4481919  C 2.2416912 -1.6124378 -1.0118529  C 2.9567562 -0.7109168 -1.8614789  C 2.9060012 -2.2340888 0.0177001  C 2.3065582 0.0182782 -2.8940029  C 4.3604252 -0.5365168 -1.6723709  C 4.2951612 -2.0530778 0.2032241  H 2.3606482 -2.9075468 0.6734711  C 3.0053232 0.8956852 -3.6851539  H 1.2380422 -0.1132278 -3.0308999  C 5.0555982 0.3529492 -2.5342769  C 5.0345422 -1.2520448 -0.6315039  H 4.7941712 -2.5851968 1.0101591  C 4.3967372 1.0612752 -3.5066019  H 2.4897422 1.4795902 -4.4409609  H 6.1226502 0.4970502 -2.3964839  H 4.9324692 1.7767012 -4.1214739  C -4.0552078 1.8001692 1.3302761  C -4.4573808 2.5877192 0.2029671  C -3.8654098 2.4159072 2.5437441  C -4.7230668 2.0068532 -1.0649829  C -4.6163848 3.9958412 0.3576411  C -4.0396928 3.8091852 2.6934761  H -3.5452128 1.8220492 3.3951551  C -5.0994248 2.7779862 -2.1343609  H -4.6266208 0.9321512 -1.1818609  C -5.0012488 4.7657382 -0.7727539  C -4.3973308 4.6052052 1.6346311  H -3.8708678 4.2599252 3.6678501  C -5.2331598 4.1761492 -1.9892869  H -5.2933118 2.3135522 -3.0962399  H -5.1165668 5.8397542 -0.6672499  H -5.5248618 4.7824122 -2.8413849  C 6.5296752 -1.1562168 -0.4771249  H 7.0404002 -1.5406348 -1.3677179  H 6.8671132 -0.1265108 -0.3221839  H 6.8609822 -1.7474278 0.3820711  C -4.5599148 6.0915982 1.8137071  H -3.8759798 6.6508852 1.1658381  H -5.5777638 6.4162762 1.5705791  H -4.3528508 6.3777982 2.8475341  P -0.6063688 0.9520272 -0.0282569  O -2.1314238 0.6570922 -0.4322359 | **INT2-B**  C 3.3163063 1.6677031 -0.1254120  O 4.3493283 1.8701121 -0.9242750  C 3.8645163 2.1874791 -2.2488100  C 2.4702853 2.2338151 -2.1001310  N 2.2032943 1.9132491 -0.7810390  C 3.4583523 1.1312401 1.2155320  C 2.3269293 0.9431451 2.0149930  C 4.7285073 0.7630521 1.6624100  C 2.4847713 0.4028691 3.2861580  H 1.3231283 1.1708791 1.6697910  C 4.8581523 0.1972931 2.9278080  H 5.6061193 0.8903361 1.0381760  C 3.7466693 0.0247391 3.7592140  H 3.8558443 -0.4125479 4.7416990  C 1.3887603 2.6601711 -3.0394770  C 1.8612903 2.7428841 -4.4901080  H 0.9922063 3.6372171 -2.7301290  H 0.5445553 1.9599821 -2.9737110  H 1.0615243 3.1440591 -5.1180980  H 2.7425723 3.3810421 -4.5827810  H 2.1274823 1.7535851 -4.8694380  O 4.6685673 2.2994301 -3.1657230  C 2.1628163 -0.7343469 -3.1557500  O 0.9854633 -0.5166999 -3.4387380  C 3.2242023 -0.6190969 -4.2144900  H 2.7449503 -0.5111139 -5.1879240  H 3.8606463 0.2562851 -4.0153540  H 3.8753823 -1.4981959 -4.2004570  C 2.5916283 -1.1681099 -1.7912380  C 1.6531253 -1.5589989 -0.8907000  C 1.8308573 -2.0507229 0.4741720  C 0.7134573 -2.0330669 1.3237770  C 3.0437583 -2.5673109 0.9630710  C 0.8055273 -2.5071459 2.6262660  H -0.2303407 -1.6439879 0.9548140  C 3.1234733 -3.0598639 2.2585830  H 3.9150383 -2.6021899 0.3213010  C 2.0072693 -3.0328509 3.0941720  H -0.0705507 -2.4818439 3.2675930  H 4.0636303 -3.4688069 2.6179300  H 2.0744773 -3.4322129 4.1029970  H 0.6194903 -1.5240889 -1.2314580  C 3.9930693 -1.0993779 -1.5318710  C 5.1795013 -0.9217829 -1.3553160  C 6.5518823 -0.6062999 -1.1057010  C 7.1415363 0.4725661 -1.7833670  C 7.2885333 -1.3259529 -0.1540240  C 8.4642943 0.8049921 -1.5146050  H 6.5419163 1.0520621 -2.4819850  C 8.6085513 -0.9806619 0.1059290  H 6.8125113 -2.1407819 0.3831220  C 9.1994973 0.0806911 -0.5774590  H 8.9204033 1.6417121 -2.0342980  H 9.1756943 -1.5362569 0.8462130  H 10.2311173 0.3496261 -0.3715480  O 6.1198033 -0.1698039 3.2805920  O 1.3417723 0.2773981 4.0130480  C 6.2992933 -0.7796449 4.5365340  H 5.7175423 -1.7069989 4.6178960  H 7.3618063 -1.0112659 4.6116760  H 6.0170853 -0.1060379 5.3553410  C 1.4417113 -0.2732399 5.3045160  H 0.4279103 -0.2907759 5.7055270  H 1.8373893 -1.2958749 5.2710910  H 2.0783553 0.3407401 5.9540330  H -0.3588977 -0.1551489 -2.6715180  H 1.2576623 1.7282551 -0.4194980  C -4.4754117 -0.4500839 -0.3955010  C -3.3396687 -1.2097649 -0.6791380  C -3.3480087 -2.3195339 -1.5232190  C -4.5669937 -2.6560799 -2.1007330  C -5.7205607 -1.8917449 -1.8966930  C -5.6758607 -0.7639399 -1.0625450  H -4.6115227 -3.5220049 -2.7575840  C -4.3286787 0.6878651 0.5642570  C -5.0169627 0.7478071 1.7924310  C -3.3523367 1.6468221 0.2970370  C -4.7573317 1.8161871 2.6663830  C -3.0078727 2.6641681 1.1877260  C -3.7595687 2.7436931 2.3565300  H -3.5282407 3.5329011 3.0684600  C -7.0041217 -2.3244479 -2.5810540  C -8.0371287 -1.2057779 -2.6968660  C -8.1823867 -0.4900219 -1.3563010  C -6.8628997 0.1794821 -0.9808240  H -6.7671447 -2.7341399 -3.5689020  H -7.7157397 -0.4814879 -3.4563410  H -8.9950987 -1.6177229 -3.0296530  H -8.9785167 0.2600741 -1.3973480  H -8.4634767 -1.2174449 -0.5820430  H -6.6711737 1.0056301 -1.6807650  H -6.9290687 0.6443371 0.0059920  H -7.4401237 -3.1519429 -2.0037240  C -5.9110707 -0.3993749 2.2315310  C -6.8887057 -0.0086239 3.3373330  C -6.1448367 0.6877981 4.4734730  C -5.5240357 1.9863471 3.9644420  H -6.4470527 -0.8324739 1.3842360  H -7.6524557 0.6695091 2.9325360  H -7.4102577 -0.9006169 3.6987920  H -6.8155517 0.8987581 5.3124770  H -5.3566957 0.0220341 4.8485540  H -6.3249687 2.7207221 3.7993450  H -4.8624587 2.4219951 4.7209550  H -5.2545197 -1.2007619 2.6016900  O -2.1158297 -0.8154709 -0.1411750  O -2.6264487 1.5254441 -0.8874810  P -1.4239807 0.4657641 -0.8463040  O -0.2182327 0.8328461 -0.0755470  O -1.2662807 0.1686041 -2.3710110  C -2.0716947 -3.0325859 -1.8137550  C -1.4279177 -3.8307679 -0.8138410  C -1.4664437 -2.8701289 -3.0361500  C -2.0256687 -4.0674869 0.4528000  C -0.1544277 -4.4073749 -1.0922760  C -0.2009287 -3.4418539 -3.3052420  H -1.9432777 -2.2468859 -3.7871840  C -1.3970007 -4.8380969 1.3972580  H -2.9997977 -3.6342789 0.6601250  C 0.4751403 -5.1863349 -0.0841600  C 0.4701763 -4.1793039 -2.3610000  H 0.2634743 -3.2541729 -4.2694980  C -0.1294737 -5.4014469 1.1276190  H -1.8710407 -5.0127349 2.3586220  H 1.4534723 -5.6133749 -0.2790760  H 0.3707203 -5.9948039 1.8867210  C -1.7757547 3.4693291 0.9640600  C -1.5987807 4.3019871 -0.1855250  C -0.7284957 3.3040111 1.8393270  C -2.6594537 4.5574021 -1.0939180  C -0.3254427 4.8945351 -0.4298520  C 0.5333103 3.8885061 1.5881310  H -0.8455847 2.6462441 2.6969210  C -2.4756787 5.3602201 -2.1898910  H -3.6288267 4.1062281 -0.9038320  C -0.1702597 5.7227701 -1.5747450  C 0.7645843 4.6449701 0.4648360  H 1.3495333 3.6940211 2.2794070  C -1.2152487 5.9507181 -2.4339070  H -3.2991227 5.5444901 -2.8727390  H 0.7952853 6.1793651 -1.7689680  H -1.0757177 6.5864131 -3.3027630  C 1.8536173 -4.7062559 -2.6358590  H 2.5728173 -4.3122779 -1.9070970  H 1.8921913 -5.7997249 -2.5795160  H 2.1868693 -4.4071879 -3.6328620  C 2.1429533 5.1738341 0.1678750  H 2.5137213 4.7899671 -0.7915370  H 2.1582083 6.2679641 0.1135620  H 2.8458373 4.8632681 0.9449540 |
| **1a’**  C 1.4148675 -3.1301697 0.0360213  O 2.4937645 -3.6820967 0.0519533  C 0.1217445 -3.9105447 0.0579243  H 0.3502955 -4.9756457 0.0847543  H -0.4755935 -3.6284647 0.9308873  H -0.4777725 -3.6736927 -0.8269717  C 1.3131075 -1.6205477 -0.0074777  C 0.0074045 -1.0497117 -0.0045957  C -1.1305665 -0.6289597 0.0156343  C -2.4571565 -0.0947347 0.0354173  C -2.6679185 1.2617863 0.3238083  C -3.5579635 -0.9199197 -0.2361997  C -3.9569675 1.7789253 0.3364433  H -1.8130935 1.8948103 0.5415253  C -4.8436645 -0.3949617 -0.2209157  H -3.3904685 -1.9690877 -0.4587087  C -5.0467035 0.9538983 0.0643033  H -4.1126965 2.8294643 0.5612613  H -5.6906775 -1.0395587 -0.4338477  H -6.0527625 1.3614933 0.0761193  C 2.4769855 -0.9286847 -0.0133127  C 2.7247915 0.5097403 -0.0560297  C 4.0198085 0.9513423 0.2608133  C 1.7606905 1.4671533 -0.4121557  C 4.3387145 2.3027773 0.2540693  H 4.7768815 0.2162303 0.5214533  C 2.0854575 2.8175713 -0.4294677  H 0.7642225 1.1491273 -0.6935417  C 3.3692205 3.2416573 -0.0902867  H 5.3438135 2.6230463 0.5100463  H 1.3323105 3.5457683 -0.7151837  H 3.6148645 4.2991573 -0.1032027  H 3.3650585 -1.5571697 0.0394613 |  |
| **TS2-B-*SR***  C 3.0532964 1.3950627 1.0960777  O 4.1473724 0.8839187 1.6551107  C 3.7866234 -0.3019373 2.3522477  C 2.3732984 -0.4710473 2.0819587  N 1.9915114 0.7058257 1.4465397  C 3.1031244 2.5970687 0.2813397  C 1.9124374 3.1825617 -0.1596943  C 4.3507744 3.0885497 -0.1048973  C 1.9888684 4.2756487 -1.0194383  H 0.9324154 2.8201207 0.1375607  C 4.4015194 4.1924817 -0.9536843  H 5.2711264 2.6148067 0.2181957  C 3.2269864 4.7923287 -1.4222323  H 3.2725144 5.6455377 -2.0847513  C 1.3827454 -1.2275893 2.9267057  C 2.0352054 -2.1181903 3.9856037  H 0.7271944 -0.4958143 3.4155217  H 0.7311644 -1.8286873 2.2794407  H 1.2580564 -2.6595713 4.5318787  H 2.6106684 -1.5215373 4.6978227  H 2.7166134 -2.8453663 3.5381037  O 4.6215724 -0.9471333 2.9317617  C 2.6455044 0.0251397 -1.4932063  O 1.3969924 0.0829997 -1.4060433  C 3.3374924 0.8603767 -2.5401683  H 2.9105154 1.8684727 -2.5216703  H 3.1277284 0.4254817 -3.5244233  H 4.4165494 0.9085837 -2.3940603  C 3.3958994 -0.8555913 -0.6411283  C 2.6570434 -1.6684713 0.2529777  C 3.0475654 -2.9629593 0.8338257  C 4.3613364 -3.3248753 1.1697607  C 2.0147334 -3.8887223 1.0657067  C 4.6219114 -4.5766233 1.7183077  H 5.1728604 -2.6263883 1.0159107  C 2.2801664 -5.1397643 1.6077467  H 0.9960294 -3.6268453 0.7881177  C 3.5895634 -5.4859953 1.9386107  H 5.6424794 -4.8398223 1.9785767  H 1.4664014 -5.8421193 1.7607867  H 3.8037844 -6.4607263 2.3671037  H 1.5843314 -1.5944013 0.0734377  C 4.8180114 -0.8195763 -0.6453553  C 6.0259564 -0.7480613 -0.5409313  C 7.4210484 -0.6801283 -0.2289573  C 7.8031454 -0.7130933 1.1226107  C 8.4037914 -0.5857543 -1.2232593  C 9.1491524 -0.6598843 1.4627507  H 7.0298814 -0.7727763 1.8851247  C 9.7469774 -0.5265033 -0.8711033  H 8.1030064 -0.5622373 -2.2659003  C 10.1232404 -0.5664213 0.4699327  H 9.4392864 -0.6868333 2.5087537  H 10.5036844 -0.4514803 -1.6462613  H 11.1738444 -0.5233953 0.7407507  O 5.6447684 4.6185947 -1.2875833  O 0.7991604 4.7881157 -1.4221153  C 5.7575384 5.7374347 -2.1380803  H 5.2984874 5.5449317 -3.1156203  H 6.8257004 5.9090497 -2.2688343  H 5.2979734 6.6272027 -1.6903853  C 0.8054964 5.7690107 -2.4358943  H -0.2416076 5.9541497 -2.6746743  H 1.3335934 5.4121457 -3.3286503  H 1.2695074 6.7024407 -2.0889323  C -4.4035346 0.1141637 0.5628207  C -3.6953636 1.3090037 0.5133677  C -3.7527896 2.2836977 1.5097237  C -4.5937466 2.0192307 2.5851337  C -5.3040976 0.8199097 2.6998507  C -5.1903626 -0.1596053 1.7015667  H -4.6729366 2.7613337 3.3767857  C -4.2096596 -0.8780223 -0.5390683  C -5.2833706 -1.3547313 -1.3164533  C -2.9207296 -1.3574243 -0.7957123  C -5.0616266 -2.4052193 -2.2176833  C -2.6817496 -2.4353313 -1.6528593  C -3.7816556 -2.9489163 -2.3357333  H -3.6224496 -3.7894923 -3.0087943  C -6.1855606 0.6184257 3.9182637  C -6.4809956 -0.8501903 4.2110847  C -6.9276566 -1.5496973 2.9312757  C -5.7984886 -1.5372273 1.9042737  H -5.7193216 1.0997917 4.7848517  H -5.5762786 -1.3410713 4.5928427  H -7.2449116 -0.9293343 4.9912267  H -7.2285316 -2.5827933 3.1333077  H -7.8062396 -1.0326133 2.5206897  H -4.9933886 -2.2033353 2.2478057  H -6.1425676 -1.9611653 0.9583137  H -7.1359796 1.1447137 3.7503157  C -6.6390886 -0.6740143 -1.2465733  C -7.7720346 -1.5461173 -1.7815453  C -7.4030216 -2.0923343 -3.1578813  C -6.1843356 -3.0047263 -3.0436123  H -6.8585186 -0.3385663 -0.2298563  H -7.9519366 -2.3833983 -1.0925443  H -8.6971796 -0.9624683 -1.8257253  H -8.2391816 -2.6416513 -3.6024213  H -7.1762546 -1.2524363 -3.8275603  H -6.4916856 -3.9510953 -2.5762233  H -5.8029256 -3.2656183 -4.0369053  H -6.5786806 0.2429047 -1.8509333  O -2.8349386 1.5017257 -0.5637623  O -1.8439136 -0.7890683 -0.1241803  P -1.4269446 0.7472347 -0.4137323  O -0.8895106 0.9143587 -1.8693053  O -0.5701646 1.2023887 0.7075727  C -2.8640106 3.4757397 1.4502087  C -2.9701626 4.4407667 0.3989887  C -1.8730576 3.6148857 2.3905227  C -3.9959566 4.3690207 -0.5794673  C -2.0291546 5.5080357 0.3320517  C -0.9406746 4.6737707 2.3166227  H -1.7731906 2.8658297 3.1706317  C -4.0885706 5.3011127 -1.5806353  H -4.7188526 3.5608737 -0.5230253  C -2.1556576 6.4582867 -0.7162673  C -0.9886136 5.6054837 1.3097477  H -0.1542826 4.7372287 3.0641787  C -3.1566536 6.3601907 -1.6506333  H -4.8806476 5.2280397 -2.3193903  H -1.4501016 7.2823237 -0.7714353  H -3.2399136 7.1013917 -2.4402793  C -1.3291376 -3.0432793 -1.7979533  C -1.0898166 -4.3886593 -1.3591743  C -0.2950316 -2.3275453 -2.3476873  C -2.0959466 -5.1682233 -0.7262813  C 0.2084504 -4.9584503 -1.5279703  C 0.9934614 -2.8919333 -2.4880153  H -0.4698636 -1.3118193 -2.6853753  C -1.8441436 -6.4493213 -0.3046273  H -3.0754806 -4.7298883 -0.5665533  C 0.4321364 -6.2885423 -1.0835203  C 1.2665024 -4.1789363 -2.0976273  H 1.7884884 -2.2824303 -2.9098803  C -0.5665156 -7.0210053 -0.4917263  H -2.6277396 -7.0234843 0.1805477  H 1.4181744 -6.7249253 -1.2076303  H -0.3735616 -8.0366803 -0.1592823  C 0.0383604 6.7029597 1.2322887  H 0.5877084 6.6539727 0.2854537  H -0.4219186 7.6950747 1.3009697  H 0.7621814 6.6093667 2.0453757  C 2.6548444 -4.7455003 -2.2360963  H 3.0405344 -5.0928463 -1.2704213  H 2.6748344 -5.5959123 -2.9277283  H 3.3412164 -3.9824493 -2.6117343  H 0.1137314 0.7318987 -1.8845333  H 1.0267184 0.8826377 1.0966297 | **TS2-B-*RS***  C 2.8510898 -0.9536220 -0.6807933  O 3.7611818 -1.7201810 -0.0848123  C 3.1470538 -2.3526190 1.0295827  C 1.8515058 -1.7025340 1.1487667  N 1.6838548 -1.0768470 -0.0909033  C 3.1950778 -0.1804040 -1.8607233  C 2.2112648 0.5499660 -2.5324713  C 4.5383868 -0.1287620 -2.2391853  C 2.5960008 1.3403950 -3.6124473  H 1.1653638 0.5338810 -2.2369983  C 4.8974528 0.6761420 -3.3185833  H 5.3006098 -0.6812820 -1.7016933  C 3.9361748 1.4149720 -4.0145323  H 4.2218838 2.0360680 -4.8519093  C 0.6658888 -2.2547610 1.8981477  C 1.0172328 -3.3407210 2.9174127  H 0.1445568 -1.4309600 2.4019137  H -0.0446592 -2.6582910 1.1682007  H 0.0976698 -3.6713180 3.4096467  H 1.7110458 -2.9779430 3.6786097  H 1.4790658 -4.2038360 2.4360307  O 3.7466948 -3.1703490 1.6720397  C 2.4568728 1.7724890 0.5860387  O 1.2057128 1.8076570 0.5670397  C 3.1943028 2.7903290 -0.2520973  H 2.4671708 3.3648130 -0.8273463  H 3.9098768 2.3015270 -0.9215483  H 3.7716658 3.4559220 0.3987187  C 3.2206738 0.8332070 1.3651117  C 2.5204908 -0.0308860 2.2527277  C 3.0801648 -0.5800630 3.5078497  C 2.2583618 -0.5622820 4.6451407  C 4.3725168 -1.1103110 3.6228667  C 2.7109738 -1.0504890 5.8645557  H 1.2530158 -0.1549770 4.5627607  C 4.8209408 -1.6041690 4.8437827  H 5.0213918 -1.1496670 2.7562157  C 3.9974088 -1.5759340 5.9658177  H 2.0608328 -1.0229990 6.7337017  H 5.8206808 -2.0217350 4.9145627  H 4.3554178 -1.9636670 6.9147967  H 1.4803048 0.2655020 2.3727807  C 4.6342688 0.8440850 1.2224587  C 5.8310898 0.8434350 1.0116187  C 7.2440148 0.8205180 0.7929177  C 7.7693828 1.0404770 -0.4898363  C 8.1215828 0.5644810 1.8576317  C 9.1434248 1.0109340 -0.6948923  H 7.0909278 1.2205680 -1.3188413  C 9.4934888 0.5312340 1.6415627  H 7.7098788 0.3962580 2.8482057  C 10.0095028 0.7570120 0.3667097  H 9.5401578 1.1786140 -1.6920243  H 10.1639828 0.3304440 2.4717827  H 11.0821628 0.7311980 0.2009927  O 6.2213108 0.6880170 -3.6266653  O 1.5962928 2.0175610 -4.2286613  C 6.6461408 1.4925660 -4.7048893  H 6.1811208 1.1747250 -5.6460033  H 7.7256368 1.3584410 -4.7724443  H 6.4210188 2.5510070 -4.5266153  C 1.9322668 2.8769140 -5.2938453  H 0.9962878 3.3286840 -5.6214523  H 2.3802718 2.3246790 -6.1295363  H 2.6226798 3.6649280 -4.9684243  H 0.8206748 -0.6073340 -0.4243463  H 0.0038558 1.1728370 1.1945577  C -4.4643572 -0.9709220 -0.4925453  C -3.2558772 -1.6423270 -0.2986993  C -2.9108092 -2.7930070 -1.0089113  C -3.8090292 -3.2220760 -1.9814703  C -4.9825352 -2.5246100 -2.2777003  C -5.3127412 -1.3792330 -1.5392193  H -3.5688032 -4.1221710 -2.5436623  C -4.7871292 0.1700620 0.4162047  C -5.8979022 0.1517710 1.2836067  C -3.8970392 1.2388870 0.4828937  C -6.1260132 1.2540540 2.1201387  C -4.0844202 2.3363830 1.3228077  C -5.2267182 2.3231430 2.1185797  H -5.4057032 3.1688130 2.7794217  C -5.8882812 -3.0579720 -3.3707193  C -6.8622732 -2.0136260 -3.9108883  C -7.5431722 -1.2900340 -2.7514653  C -6.5063242 -0.5264930 -1.9310493  H -5.2753882 -3.4698990 -4.1799383  H -6.3192722 -1.2806710 -4.5217543  H -7.5991842 -2.4937000 -4.5627943  H -8.3069792 -0.5963030 -3.1172913  H -8.0538382 -2.0242870 -2.1126023  H -6.1252722 0.3136660 -2.5298923  H -6.9690132 -0.0707780 -1.0518583  H -6.4627262 -3.9016040 -2.9622973  C -6.7610792 -1.0921090 1.4005777  C -8.1257542 -0.8158250 2.0256537  C -7.9577592 -0.0354050 3.3258447  C -7.3370492 1.3283160 3.0320037  H -6.8791092 -1.5846670 0.4327047  H -8.7411282 -0.2316410 1.3273097  H -8.6481262 -1.7618570 2.2007897  H -8.9180962 0.0946080 3.8350407  H -7.3060892 -0.6024310 4.0033277  H -8.0929402 1.9661080 2.5521307  H -7.0582422 1.8349410 3.9625507  H -6.2171452 -1.8112810 2.0305617  O -2.3555992 -1.1186790 0.6322207  O -2.7577052 1.1947240 -0.3137093  P -1.5712932 0.1964190 0.1105307  O -0.8866452 0.6935280 1.4213507  O -0.6723462 -0.0253210 -1.0468203  C -1.6835752 -3.5808990 -0.6871133  C -0.4645652 -3.4361830 -1.4217253  C -1.7455742 -4.4981150 0.3355757  C -0.3500582 -2.5366250 -2.5157013  C 0.6707728 -4.2191640 -1.0508233  C -0.6258502 -5.2867230 0.6812047  H -2.6729502 -4.6122490 0.8901177  C 0.8287928 -2.4114860 -3.2058303  H -1.2075162 -1.9295040 -2.7846713  C 1.8836538 -4.0462110 -1.7730483  C 0.5735598 -5.1602350 0.0224477  H -0.7129992 -5.9986110 1.4980197  C 1.9612338 -3.1685880 -2.8256263  H 0.9002878 -1.7135000 -4.0342763  H 2.7589228 -4.6205990 -1.4860413  H 2.8982128 -3.0438020 -3.3612103  C -3.0627532 3.4121580 1.4273127  C -2.7191772 4.2416020 0.3119177  C -2.4181292 3.5978240 2.6259687  C -3.3786252 4.1272830 -0.9398533  C -1.6970442 5.2232100 0.4628427  C -1.4066242 4.5733020 2.7689647  H -2.6680512 2.9574270 3.4671807  C -3.0405432 4.9346190 -1.9952263  H -4.1635952 3.3862080 -1.0539493  C -1.3680012 6.0386910 -0.6527673  C -1.0290962 5.3734310 1.7200067  H -0.9066332 4.6780450 3.7281507  C -2.0204622 5.9005100 -1.8513393  H -3.5559872 4.8308270 -2.9450753  H -0.5881592 6.7863500 -0.5497053  H -1.7549902 6.5347360 -2.6917643  C 1.7661048 -5.9877850 0.4252867  H 2.1154418 -6.6144020 -0.4029023  H 2.6051378 -5.3549110 0.7389827  H 1.5099258 -6.6432480 1.2613687  C 0.0728128 6.3862980 1.8858947  H 0.9015488 6.1860770 1.1976217  H -0.2807842 7.4039760 1.6860297  H 0.4676538 6.3602270 2.9040657 |
| **TS2-B-*RR***  C -2.2096443 2.7495748 0.1889045  O -3.5300683 2.6609058 0.2440745  C -3.8869193 1.7218248 1.2715185  C -2.6384353 1.2781058 1.7928755  N -1.6644683 1.9868578 1.1110325  C -1.5506803 3.6431368 -0.7479565  C -0.1581453 3.7543848 -0.7523885  C -2.3435863 4.3642358 -1.6417275  C 0.4343047 4.6245618 -1.6620605  H 0.4753787 3.1673498 -0.0940575  C -1.7262283 5.2160598 -2.5535255  H -3.4221943 4.2618868 -1.6457715  C -0.3351473 5.3607688 -2.5712525  H 0.1372197 6.0327548 -3.2741205  C -2.3581743 0.5915358 3.0953245  C -3.6339423 0.2749388 3.8784505  H -1.7138523 1.2431038 3.7004965  H -1.7729563 -0.3198852 2.9310685  H -3.3815573 -0.2808452 4.7860885  H -4.1520633 1.1937538 4.1660935  H -4.3399013 -0.3095812 3.2824895  O -5.0545233 1.4622438 1.4611735  C -2.8678053 -2.6241202 2.0249345  O -1.6174063 -2.7751362 2.2773195  C -3.7471653 -3.5568102 2.8003585  H -3.1303173 -4.2284662 3.3964095  H -4.4098003 -2.9767492 3.4516595  H -4.3920623 -4.1233352 2.1216055  C -3.4535863 -1.7239872 1.0942295  C -2.6524523 -0.8925542 0.3053745  C -2.9640943 -0.1563492 -0.9003515  C -1.8684373 0.4163008 -1.5790405  C -4.2631393 0.0347208 -1.4217645  C -2.0617203 1.1638218 -2.7349265  H -0.8610223 0.2705328 -1.1929735  C -4.4408463 0.7798438 -2.5752325  H -5.1224723 -0.3762402 -0.9083085  C -3.3461573 1.3523058 -3.2312675  H -1.2055653 1.6109698 -3.2315345  H -5.4434593 0.9307888 -2.9629765  H -3.5019383 1.9489418 -4.1257385  H -1.5838013 -0.9659802 0.4983315  C -4.8825663 -1.7185572 1.0408345  C -6.0944053 -1.6924322 1.0444275  C -7.5066283 -1.4568762 1.0151405  C -7.9540953 -0.1271592 0.9596985  C -8.4331843 -2.5068312 1.0314625  C -9.3180153 0.1357298 0.9174295  H -7.2175703 0.6723498 0.9635435  C -9.7947313 -2.2301202 0.9931085  H -8.0768693 -3.5314822 1.0718225  C -10.2388273 -0.9106272 0.9340265  H -9.6646183 1.1637908 0.8764445  H -10.5109163 -3.0459842 1.0071385  H -11.3032283 -0.6984012 0.9028595  O -2.5596653 5.8756048 -3.3993485  O 1.7900237 4.7064198 -1.5980315  C -1.9857613 6.7487878 -4.3456035  H -1.3095673 6.2142948 -5.0244435  H -2.8167333 7.1636378 -4.9159995  H -1.4386563 7.5644488 -3.8572115  C 2.4518037 5.5548158 -2.5087565  H 3.5159927 5.4501268 -2.2977605  H 2.2561857 5.2570168 -3.5460865  H 2.1547777 6.6015688 -2.3687415  H -0.8889513 -2.1284142 1.8958475  H -0.6460003 1.7312158 1.1209835  C 3.5796027 -2.1812512 -0.2756265  C 2.2077047 -2.2659162 -0.5271525  C 1.5660687 -3.4854592 -0.7559855  C 2.3487897 -4.6352002 -0.6795065  C 3.7043917 -4.5993202 -0.3489205  C 4.3278137 -3.3642932 -0.1229995  H 1.8736747 -5.5965622 -0.8648895  C 4.1824037 -0.8230252 -0.1358925  C 5.1761047 -0.3460842 -1.0131265  C 3.6546387 0.0316958 0.8316175  C 5.6808727 0.9488378 -0.8270395  C 4.1045047 1.3433788 1.0003375  C 5.1435367 1.7614458 0.1725645  H 5.5216177 2.7747968 0.2921045  C 4.4691737 -5.9079712 -0.2750095  C 5.7692457 -5.8042952 0.5194605  C 6.5515777 -4.5718152 0.0747055  C 5.7560707 -3.3077562 0.3895525  H 3.8202877 -6.6838662 0.1463515  H 5.5440537 -5.7178362 1.5904805  H 6.3601917 -6.7165672 0.3870205  H 7.5271277 -4.5271132 0.5699215  H 6.7412977 -4.6305772 -1.0064045  H 5.7080487 -3.1781672 1.4807635  H 6.2748277 -2.4221452 0.0145355  H 4.7050697 -6.2300372 -1.2993085  C 5.5960767 -1.1670652 -2.2204665  C 6.9313367 -0.7228802 -2.8128535  C 6.9333817 0.7873918 -3.0313915  C 6.8009487 1.5010288 -1.6883775  H 5.6206067 -2.2336242 -1.9858605  H 7.7473187 -0.9911552 -2.1272855  H 7.1133677 -1.2554102 -3.7521575  H 7.8477977 1.1113748 -3.5393135  H 6.0891747 1.0573798 -3.6794465  H 7.7479937 1.3988988 -1.1395815  H 6.6488257 2.5763238 -1.8349525  H 4.8110447 -1.0544042 -2.9830245  O 1.4621727 -1.1004972 -0.5446515  O 2.6082127 -0.4392202 1.6082935  P 1.1421037 -0.4003072 0.9028415  O 0.7167087 0.9988378 0.5895605  O 0.2603427 -1.2896132 1.7339975  C 0.1037387 -3.5786822 -1.0096615  C -0.5248563 -2.9249082 -2.1211935  C -0.6617633 -4.3618732 -0.1783745  C 0.2150797 -2.1607202 -3.0626925  C -1.9299463 -3.0710492 -2.3153155  C -2.0523373 -4.5046282 -0.3794965  H -0.1909063 -4.8517342 0.6693785  C -0.4009963 -1.5669282 -4.1340885  H 1.2870927 -2.0598592 -2.9301475  C -2.5395353 -2.4260992 -3.4258495  C -2.6999383 -3.8649532 -1.4067735  H -2.6245473 -5.1178422 0.3141725  C -1.7959653 -1.6976822 -4.3173705  H 0.1853067 -0.9908742 -4.8439175  H -3.6122273 -2.5127452 -3.5662985  H -2.2801283 -1.2099622 -5.1580965  C 3.4142007 2.2964378 1.9085995  C 3.2879747 2.0728088 3.3158095  C 2.8483607 3.4197078 1.3542105  C 3.8921317 0.9581198 3.9542645  C 2.5512397 3.0025948 4.1068355  C 2.1173857 4.3353718 2.1422495  H 2.9173607 3.5817008 0.2818315  C 3.7660847 0.7608068 5.3050035  H 4.4616037 0.2572888 3.3520875  C 2.4364267 2.7644838 5.5030315  C 1.9494447 4.1469538 3.4920415  H 1.6631557 5.1962598 1.6565615  C 3.0255797 1.6729908 6.0888865  H 4.2344927 -0.0986822 5.7744435  H 1.8742767 3.4625978 6.1152145  H 2.9258307 1.5097698 7.1578405  C -4.1932593 -3.9660782 -1.5601475  H -4.6590083 -2.9740312 -1.5124255  H -4.4739473 -4.4180822 -2.5181375  H -4.6244463 -4.5727452 -0.7589835  C 1.1485837 5.1251598 4.3110295  H 0.3049327 4.6347968 4.8094045  H 1.7596057 5.5920958 5.0917895  H 0.7488437 5.9191628 3.6757835 | **TS2-B-*SS***  C -3.9404757 1.1500239 -0.3831980  O -4.9527397 1.9629329 -0.1394890  C -4.4872337 3.0198659 0.7473960  C -3.0789937 2.8038339 0.8388070  N -2.8401817 1.5962739 0.1764120  C -4.1442777 -0.0768001 -1.1359610  C -3.0702887 -0.9299341 -1.3739110  C -5.4385287 -0.3972811 -1.5536590  C -3.2956487 -2.1307581 -2.0472210  H -2.0779257 -0.7244291 -0.9989590  C -5.6514397 -1.6191221 -2.1846310  H -6.2767817 0.2625489 -1.3636930  C -4.5849867 -2.4843651 -2.4544840  H -4.7582747 -3.4355331 -2.9380530  C -2.1630077 3.2516689 1.9444110  C -2.8834767 4.0755579 3.0125810  H -1.7153287 2.3579749 2.3981630  H -1.3222117 3.8234009 1.5341210  H -2.1640497 4.4111959 3.7641870  H -3.6570127 3.4841639 3.5120610  H -3.3711957 4.9524629 2.5796100  O -5.2726557 3.8430699 1.1405150  C -0.9684257 2.7576539 -2.1050370  O 0.2440343 2.3585499 -2.3084990  C -2.0165747 2.0487629 -2.9115900  H -1.9074797 0.9677249 -2.7621800  H -1.8027057 2.2426089 -3.9684790  H -3.0448877 2.3339919 -2.6978230  C -1.1661587 3.8337049 -1.2155870  C -2.4642257 4.2982839 -0.9094100  C -2.8412117 5.5375489 -0.2406530  C -4.1815577 5.9499009 -0.3568090  C -1.9583897 6.3331839 0.5165310  C -4.6311897 7.1082639 0.2626320  H -4.8737817 5.3490939 -0.9385770  C -2.4152937 7.4868729 1.1363600  H -0.9192857 6.0470369 0.6132930  C -3.7496007 7.8772049 1.0154580  H -5.6710067 7.4018699 0.1645830  H -1.7253607 8.0885889 1.7197880  H -4.0981537 8.7809039 1.5062990  H -3.2588437 3.8898079 -1.5274210  C 0.0300253 4.2756229 -0.5624350  C 1.1032143 4.5113979 -0.0505230  C 2.3560003 4.7237319 0.6088950  C 2.7773163 6.0116929 0.9694460  C 3.1689883 3.6187479 0.9045990  C 3.9958773 6.1901319 1.6140500  H 2.1457643 6.8628789 0.7332130  C 4.3866653 3.8073659 1.5473020  H 2.8403063 2.6246709 0.6107540  C 4.8040183 5.0900039 1.8997930  H 4.3183793 7.1897809 1.8893660  H 5.0209953 2.9497369 1.7541380  H 5.7608743 5.2314399 2.3935850  O -6.9409297 -1.9001861 -2.5001950  O -2.2079037 -2.9033851 -2.2465130  C -7.2244337 -3.1790571 -3.0252790  H -6.7557337 -3.3228181 -4.0064200  H -8.3077977 -3.2269111 -3.1341970  H -6.8868777 -3.9670961 -2.3408180  C -2.3611967 -4.0865391 -2.9973020  H -1.3650157 -4.5224491 -3.0707180  H -2.7407327 -3.8703421 -4.0038920  H -3.0339257 -4.7910701 -2.4936270  H 0.3116693 1.2810819 -2.3793080  C 1.6230933 -2.9583801 0.8569680  C 0.2968433 -2.5481591 0.6976420  C -0.6898457 -2.8424541 1.6400830  C -0.3048677 -3.5881331 2.7509170  C 1.0175933 -3.9692581 2.9765480  C 2.0038043 -3.6216621 2.0425870  H -1.0624747 -3.8369501 3.4916470  C 2.6272833 -2.6037301 -0.1921160  C 3.4111213 -3.5781781 -0.8434180  C 2.8168583 -1.2562321 -0.5100290  C 4.4638433 -3.1668091 -1.6713500  C 3.8835803 -0.8208681 -1.3031380  C 4.7046343 -1.8031571 -1.8491200  H 5.5460803 -1.4887451 -2.4640050  C 1.3408993 -4.7492501 4.2369840  C 2.8076253 -4.6479251 4.6461670  C 3.6955133 -4.9182761 3.4355450  C 3.4713893 -3.8517801 2.3661250  H 0.6880903 -4.4111011 5.0491760  H 3.0175013 -3.6407911 5.0293480  H 3.0191593 -5.3529761 5.4567350  H 4.7527413 -4.9345761 3.7199920  H 3.4584053 -5.9098271 3.0242850  H 3.8786483 -2.8944851 2.7232710  H 4.0439053 -4.0936601 1.4688130  H 1.0939193 -5.8068061 4.0658660  C 3.0640983 -5.0518671 -0.7189170  C 4.2290743 -5.9704811 -1.0777640  C 4.8085423 -5.5696871 -2.4314790  C 5.3887353 -4.1601001 -2.3502150  H 2.6845413 -5.2828011 0.2793550  H 5.0109983 -5.8948631 -0.3086530  H 3.8896783 -7.0114731 -1.0886410  H 5.5821633 -6.2736141 -2.7553430  H 4.0096823 -5.5990221 -3.1841510  H 6.3322543 -4.1963561 -1.7871600  H 5.6453793 -3.7911361 -3.3494060  H 2.2279373 -5.2523111 -1.4050190  O -0.0377007 -1.8480871 -0.4439510  O 1.9405953 -0.3211041 0.0201630  P 0.4299153 -0.2943621 -0.5840780  O 0.4079103 -0.0521301 -2.0823280  O -0.3769227 0.6076619 0.2979120  C -2.0928487 -2.3344741 1.5637620  C -3.1617887 -3.1534441 1.0783540  C -2.3858637 -1.1022651 2.0961970  C -2.9165347 -4.4260771 0.5001270  C -4.5065837 -2.6842001 1.1679700  C -3.7214107 -0.6450331 2.1871220  H -1.5754447 -0.4759731 2.4584320  C -3.9513617 -5.2099851 0.0508980  H -1.8892117 -4.7700681 0.4205030  C -5.5516457 -3.5179721 0.6880240  C -4.7782027 -1.4017011 1.7441760  H -3.9122417 0.3339579 2.6252310  C -5.2847057 -4.7530481 0.1506230  H -3.7468727 -6.1889531 -0.3741200  H -6.5781967 -3.1697471 0.7523120  H -6.0993127 -5.3846841 -0.1933550  C 4.1549843 0.6261779 -1.5291100  C 5.3336063 1.2398769 -0.9909570  C 3.2750303 1.3904019 -2.2542670  C 6.2583543 0.5243329 -0.1824500  C 5.5746453 2.6231049 -1.2432950  C 3.4935283 2.7712429 -2.4544540  H 2.3964103 0.9162859 -2.6763330  C 7.3777323 1.1333359 0.3274470  H 6.0620433 -0.5200821 0.0377340  C 6.7485893 3.2178779 -0.7081970  C 4.6133803 3.3972469 -1.9672250  H 2.7362963 3.3465139 -2.9800340  C 7.6324303 2.4954079 0.0530390  H 8.0682543 0.5673929 0.9456330  H 6.9368983 4.2705499 -0.8935740  H 8.5225183 2.9706949 0.4544510  C -6.1897277 -0.8883131 1.8543420  H -6.6797927 -0.8602471 0.8744050  H -6.7982767 -1.5238081 2.5075590  H -6.1994857 0.1255729 2.2624960  C 4.7904603 4.8837689 -2.1193820  H 4.8472293 5.3664769 -1.1353000  H 5.7054293 5.1354919 -2.6677750  H 3.9424703 5.3152909 -2.6565470  H -1.8917477 1.1282319 0.1678940 |
| **INT3-B-*SR***  C 2.9588656 1.6946419 1.1685782  O 4.1377306 1.3596469 1.7041672  C 3.9894886 0.0970599 2.3168312  C 2.5908476 -0.3795241 1.9752502  N 2.0121356 0.8623559 1.4784172  C 2.8523636 2.8858909 0.3416392  C 1.5947086 3.2747759 -0.1230278  C 4.0282096 3.5205059 -0.0623208  C 1.5260096 4.3252909 -1.0356228  H 0.6740896 2.7860289 0.1833432  C 3.9331716 4.5779589 -0.9647328  H 5.0019206 3.1877859 0.2773172  C 2.6877036 4.9835559 -1.4594218  H 2.6206176 5.7985939 -2.1674628  C 1.7660516 -0.9688221 3.1313902  C 2.5205746 -1.8373011 4.1393402  H 1.3023306 -0.1287361 3.6594472  H 0.9477226 -1.5380361 2.6749172  H 1.8013906 -2.2393501 4.8582852  H 3.2620236 -1.2551261 4.6910782  H 3.0341376 -2.6757781 3.6654932  O 4.8822956 -0.4027301 2.9172642  C 2.6780966 0.0788029 -1.3523838  O 1.4048446 0.1439569 -1.1985538  C 3.2828286 0.8307499 -2.5078628  H 2.9890826 1.8853819 -2.4355098  H 2.8672566 0.4406209 -3.4436808  H 4.3703386 0.7559709 -2.5324698  C 3.4391396 -0.6544511 -0.4494858  C 2.6926456 -1.3368971 0.6759402  C 3.0902776 -2.7422101 1.0703352  C 4.4052626 -3.1343851 1.3443082  C 2.0650176 -3.6848151 1.2151812  C 4.6780756 -4.4351721 1.7605932  H 5.2173386 -2.4255111 1.2378862  C 2.3359736 -4.9824071 1.6387642  H 1.0420816 -3.4017591 0.9726812  C 3.6478746 -5.3605401 1.9154282  H 5.7040616 -4.7229691 1.9696112  H 1.5233966 -5.6965291 1.7337712  H 3.8673846 -6.3716771 2.2456172  H 1.6469786 -1.3815401 0.3513852  C 4.8548756 -0.6294811 -0.4847748  C 6.0697606 -0.5745261 -0.4315488  C 7.4921846 -0.5457261 -0.3034188  C 8.0763796 -0.6441931 0.9704702  C 8.3230696 -0.4271521 -1.4277278  C 9.4587336 -0.6347531 1.1078302  H 7.4291906 -0.7156421 1.8401812  C 9.7042586 -0.4111041 -1.2794068  H 7.8717756 -0.3520541 -2.4120368  C 10.2777886 -0.5180681 -0.0137778  H 9.8996246 -0.7148151 2.0970332  H 10.3374206 -0.3207711 -2.1571288  H 11.3576106 -0.5095261 0.0980482  O 5.1059266 5.1538769 -1.3208228  O 0.2851806 4.6446989 -1.4645578  C 5.0748106 6.1879999 -2.2804968  H 4.6501706 5.8374679 -3.2289198  H 6.1118646 6.4840449 -2.4357168  H 4.5008576 7.0503849 -1.9200988  C 0.1611446 5.5383849 -2.5504808  H -0.8982494 5.5518049 -2.8028428  H 0.7484966 5.1954109 -3.4108078  H 0.4783436 6.5528179 -2.2719778  C -4.3781924 -0.0582951 0.5343922  C -3.7005004 1.1552899 0.5013032  C -3.8266254 2.1362659 1.4855752  C -4.6996304 1.8621709 2.5309962  C -5.3808624 0.6443049 2.6296302  C -5.2015794 -0.3407241 1.6462602  H -4.8287214 2.6088059 3.3116572  C -4.1194034 -1.0584741 -0.5482798  C -5.1542674 -1.5861701 -1.3462758  C -2.8084334 -1.5006571 -0.7582098  C -4.8737584 -2.6519761 -2.2116618  C -2.5109954 -2.5929451 -1.5816258  C -3.5746204 -3.1573171 -2.2813338  H -3.3702804 -4.0057701 -2.9320698  C -6.3042774 0.4308549 3.8147752  C -6.5725804 -1.0419481 4.1122882  C -6.9485374 -1.7654271 2.8232232  C -5.7800284 -1.7322201 1.8419932  H -5.8854434 0.9328339 4.6939682  H -5.6709994 -1.5046641 4.5345772  H -7.3645574 -1.1337211 4.8625922  H -7.2297994 -2.8043311 3.0239432  H -7.8233524 -1.2760671 2.3725142  H -4.9713564 -2.3721181 2.2247502  H -6.0743124 -2.1766761 0.8888892  H -7.2609944 0.9299149 3.6045712  C -6.5309504 -0.9455321 -1.3385418  C -7.6212734 -1.8714511 -1.8718588  C -7.2001244 -2.4479861 -3.2208818  C -5.9532484 -3.3120421 -3.0495608  H -6.7888774 -0.5790641 -0.3417608  H -7.7908424 -2.6919511 -1.1602908  H -8.5639514 -1.3212371 -1.9576398  H -8.0059024 -3.0404431 -3.6663508  H -6.9871684 -1.6218791 -3.9119648  H -6.2397644 -4.2556471 -2.5635958  H -5.5372634 -3.5866651 -4.0251828  H -6.4809654 -0.0506641 -1.9759668  O -2.8102704 1.3756219 -0.5405028  O -1.7730394 -0.8883551 -0.0687118  P -1.3794474 0.6594299 -0.3585708  O -0.7772594 0.8555789 -1.7669968  O -0.5907474 1.1153959 0.8209662  C -2.9696454 3.3531249 1.4331542  C -3.1489044 4.3523689 0.4252822  C -1.9362114 3.4798209 2.3272652  C -4.2133164 4.2833659 -0.5105448  C -2.2404584 5.4471959 0.3565622  C -1.0278034 4.5596379 2.2434592  H -1.7811764 2.7035649 3.0703212  C -4.3815814 5.2529059 -1.4661008  H -4.9023524 3.4457939 -0.4577478  C -2.4468914 6.4366129 -0.6407358  C -1.1472564 5.5265589 1.2768312  H -0.2015224 4.6097949 2.9481552  C -3.4898824 6.3463119 -1.5295568  H -5.2027084 5.1834369 -2.1727988  H -1.7683984 7.2832339 -0.6961238  H -3.6332734 7.1175279 -2.2809148  C -1.1295224 -3.1399041 -1.6898668  C -0.8330524 -4.4896911 -1.3003848  C -0.1136554 -2.3475021 -2.1648448  C -1.8165534 -5.3554331 -0.7485598  C 0.5025826 -4.9775341 -1.4367418  C 1.2099096 -2.8291481 -2.2658418  H -0.3312824 -1.3297121 -2.4673628  C -1.5102354 -6.6425881 -0.3842218  H -2.8239874 -4.9791181 -0.6049048  C 0.7819866 -6.3166861 -1.0547968  C 1.5396886 -4.1141621 -1.9159448  H 1.9869676 -2.1570581 -2.6188498  C -0.1971084 -7.1345401 -0.5484678  H -2.2786754 -7.2842341 0.0365282  H 1.7957336 -6.6910431 -1.1581518  H 0.0383436 -8.1562111 -0.2653438  C -0.1385344 6.6394569 1.1769212  H 0.3559436 6.6331319 0.1975092  H -0.6026604 7.6236389 1.3063462  H 0.6338496 6.5267499 1.9416162  C 2.9678926 -4.5847371 -1.9948378  H 3.3224846 -4.9366621 -1.0184468  H 3.0849056 -5.4071861 -2.7101798  H 3.6185746 -3.7632001 -2.3051508  H 0.2962596 0.6550989 -1.7121468  H 1.0331716 0.9105749 1.0998462 | **INT3-B-*RS***  C 2.7231529 -1.1055483 -0.6474705  O 3.6820329 -1.7326923 0.0435205  C 3.1784249 -1.9798703 1.3367385  C 1.8573899 -1.2391123 1.4239845  N 1.6146629 -0.9886973 0.0013215  C 3.0752319 -0.5146743 -1.9353145  C 2.1077229 0.1757907 -2.6650085  C 4.4224229 -0.5206323 -2.2979675  C 2.5146149 0.8641767 -3.8064015  H 1.0627149 0.2094847 -2.3662385  C 4.8075859 0.1905657 -3.4332285  H 5.1717459 -1.0241903 -1.6978475  C 3.8612279 0.8815487 -4.1958065  H 4.1651379 1.4339207 -5.0744695  C 0.6924349 -1.9685833 2.1151205  C 1.0560799 -2.8882693 3.2806065  H -0.0089361 -1.1968843 2.4507085  H 0.1664049 -2.5448263 1.3507145  H 0.1270809 -3.2788593 3.7065275  H 1.6040189 -2.3681933 4.0697935  H 1.6577209 -3.7391543 2.9526025  O 3.7970019 -2.6283333 2.1146635  C 2.5190389 2.0081987 0.3705925  O 1.2371409 2.3074187 0.1699395  C 3.4139369 2.8119437 -0.5334855  H 2.7944719 3.4272517 -1.1890195  H 4.0433289 2.1499017 -1.1394945  H 4.0843609 3.4505557 0.0499945  C 3.0129869 1.0858787 1.2522605  C 2.0977669 0.2136717 2.0804465  C 2.3893429 0.1240267 3.5636205  C 1.3477659 0.4372867 4.4441555  C 3.6209699 -0.2771953 4.0907385  C 1.5227789 0.3380297 5.8207225  H 0.3912029 0.7593267 4.0364905  C 3.7944079 -0.3756213 5.4686395  H 4.4450219 -0.5194343 3.4294225  C 2.7483409 -0.0738403 6.3368605  H 0.7019199 0.5830587 6.4879355  H 4.7537159 -0.6954373 5.8644515  H 2.8895719 -0.1562773 7.4104035  H 1.0939219 0.6293347 2.0065955  C 4.4231479 0.8817107 1.2436375  C 5.6184419 0.6877067 1.1419565  C 7.0214159 0.4442467 1.0035195  C 7.6252359 0.4964217 -0.2625585  C 7.8079689 0.1415727 2.1247385  C 8.9869879 0.2544237 -0.3968335  H 7.0163829 0.7218547 -1.1340025  C 9.1674859 -0.1045693 1.9804385  H 7.3389749 0.1036857 3.1031355  C 9.7618479 -0.0472893 0.7213165  H 9.4441529 0.2968997 -1.3809505  H 9.7663919 -0.3412793 2.8545335  H 10.8248409 -0.2387303 0.6118185  O 6.1344759 0.1681667 -3.7167485  O 1.5350809 1.5049457 -4.4829255  C 6.5882949 0.9048047 -4.8323195  H 6.1445929 0.5311777 -5.7629185  H 7.6682499 0.7640037 -4.8655105  H 6.3629239 1.9724107 -4.7225445  C 1.8961809 2.3193697 -5.5760255  H 0.9700489 2.7708777 -5.9302995  H 2.3447619 1.7298357 -6.3853235  H 2.5936239 3.1087717 -5.2697835  H 0.6475649 -0.6149323 -0.4249785  H 0.5628409 1.8314657 0.7202065  C -4.4666551 -0.9428753 -0.3526185  C -3.2759481 -1.6157953 -0.0509435  C -2.9717061 -2.8658123 -0.5959385  C -3.8875311 -3.4108003 -1.4923985  C -5.0451431 -2.7340953 -1.8817135  C -5.3312291 -1.4789053 -1.3252615  H -3.6742511 -4.3877253 -1.9225355  C -4.7340341 0.3610757 0.3283945  C -5.8441631 0.5845927 1.1658115  C -3.7760571 1.3642357 0.1948005  C -5.9993191 1.8438457 1.7661425  C -3.8714171 2.6018557 0.8354155  C -5.0175601 2.8234167 1.5927265  H -5.1314961 3.7829767 2.0932265  C -5.9752001 -3.3973683 -2.8817325  C -6.9204781 -2.4165933 -3.5720245  C -7.5630781 -1.4995383 -2.5353275  C -6.4918541 -0.6538293 -1.8532465  H -5.3811301 -3.9457923 -3.6212845  H -6.3597401 -1.8058063 -4.2916535  H -7.6794571 -2.9664373 -4.1381625  H -8.3097911 -0.8464483 -2.9986695  H -8.0877791 -2.1081103 -1.7854395  H -6.0809491 0.0592187 -2.5829695  H -6.9309111 -0.0395493 -1.0638195  H -6.5735661 -4.1517783 -2.3512685  C -6.7858831 -0.5534893 1.5176725  C -8.1385461 -0.0700613 2.0354575  C -7.9362571 0.9502947 3.1522335  C -7.2165801 2.1815467 2.6065935  H -6.9195211 -1.2332983 0.6727085  H -8.7039791 0.3944277 1.2153625  H -8.7261071 -0.9245883 2.3869585  H -8.8932991 1.2439507 3.5960195  H -7.3366531 0.4933767 3.9503785  H -7.9186481 2.7551357 1.9846455  H -6.9199311 2.8481247 3.4239825  H -6.2981311 -1.1529863 2.3004085  O -2.3699541 -1.0114643 0.8050295  O -2.6549721 1.0863257 -0.5653705  P -1.4965611 0.2229537 0.1701445  O -0.8413381 0.9338487 1.3125235  O -0.6155531 -0.2819943 -0.9514905  C -1.7400791 -3.6156003 -0.2005505  C -0.5536881 -3.5987553 -1.0016275  C -1.7525231 -4.3638203 0.9531595  C -0.4851681 -2.8648393 -2.2161015  C 0.5974149 -4.3259983 -0.5698275  C -0.6201141 -5.1050823 1.3604825  H -2.6503081 -4.3797573 1.5644365  C 0.6640759 -2.8424963 -2.9638395  H -1.3548711 -2.2970223 -2.5277105  C 1.7780429 -4.2672093 -1.3615585  C 0.5480139 -5.0933383 0.6366855  H -0.6700731 -5.6829163 2.2800495  C 1.8108199 -3.5473433 -2.5297865  H 0.7000829 -2.2684353 -3.8847205  H 2.6618639 -4.8093803 -1.0395885  H 2.7207249 -3.5148163 -3.1227885  C -2.7124171 3.5333247 0.8365225  C -2.1198581 4.0282637 -0.3690945  C -2.1246091 3.8340447 2.0413265  C -2.7205001 3.8210957 -1.6384345  C -0.8955711 4.7523657 -0.2998895  C -0.9197841 4.5670037 2.1052745  H -2.5563301 3.4361927 2.9553305  C -2.1322551 4.2851187 -2.7861605  H -3.6625691 3.2836877 -1.6880415  C -0.3039381 5.2053217 -1.5094215  C -0.2839221 5.0012197 0.9695335  H -0.4658591 4.7516657 3.0755475  C -0.9033101 4.9783877 -2.7220155  H -2.6084961 4.1164577 -3.7472275  H 0.6363629 5.7461677 -1.4675745  H -0.4374121 5.3373337 -3.6353435  C 1.7578589 -5.8557293 1.1102925  H 2.0722659 -6.6083713 0.3786555  H 2.6114529 -5.1876903 1.2793035  H 1.5436239 -6.3669823 2.0519815  C 1.0459159 5.6997137 1.0538105  H 1.8153759 5.1249747 0.5249655  H 1.0125029 6.6996067 0.6067465  H 1.3598299 5.8049877 2.0950305 |
| **TS3-B-*SRS***  C 1.6155975 0.6659319 -3.6774042  O 0.5344565 -0.0274821 -3.7652642  C 2.0805795 1.2079449 -4.9928112  H 1.6645005 2.2146319 -5.1198152  H 3.1672775 1.2847449 -5.0538192  H 1.6961345 0.5713779 -5.7911362  C 2.3046185 0.9120739 -2.4703382  C 1.9969385 0.1196229 -1.4055212  C 1.6270015 -0.5527491 -0.3991792  C 1.9889245 -1.7928171 0.2867798  C 1.2991435 -2.1461651 1.4508768  C 3.0360235 -2.6050771 -0.1718772  C 1.6816415 -3.2754311 2.1694738  H 0.4702935 -1.5260641 1.7818048  C 3.4143465 -3.7293721 0.5510118  H 3.5750895 -2.3184271 -1.0716572  C 2.7482405 -4.0570731 1.7320408  H 1.1408985 -3.5470881 3.0717858  H 4.2471205 -4.3362931 0.2070848  H 3.0592495 -4.9235871 2.3087958  H -0.0307525 -0.0942401 -2.8969552  H 0.5971955 0.0828469 0.1067828  C 3.3731485 2.0036969 -2.4267702  C 4.2017135 2.0314409 -1.1152542  C 2.7728255 3.3572639 -2.7842222  C 3.4090695 2.2252269 0.1773728  N 4.9018055 0.7677239 -0.9078432  C 5.2399295 3.1814019 -1.1670922  C 1.5677005 3.7912659 -2.2227292  C 3.4202965 4.1781649 -3.7109192  O 3.8218145 1.2211959 1.0285178  O 2.6701625 3.0971129 0.5182948  C 4.6779385 0.4259799 0.2979268  C 6.1730875 3.2333479 0.0395538  H 5.8224315 3.0508829 -2.0869682  H 4.6860605 4.1211719 -1.2527662  C 1.0369345 5.0280839 -2.5767092  H 1.0396135 3.1667369 -1.5063632  C 2.8898605 5.4155559 -4.0644602  H 4.3503315 3.8393459 -4.1642192  C 5.2541715 -0.7214691 1.0133238  H 6.8827585 4.0566749 -0.0753522  H 6.7440495 2.3066129 0.1427028  H 5.6200335 3.4055319 0.9693398  C 1.6942705 5.8441079 -3.4946292  H 0.0996335 5.3458819 -2.1306572  H 3.4065785 6.0383529 -4.7884722  C 6.2587755 -1.4432941 0.3873788  C 4.7627765 -1.0712011 2.2768268  H 1.2720955 6.8055049 -3.7712422  C 6.7916035 -2.5617541 1.0362398  H 6.6244905 -1.1553881 -0.5914862  C 5.2943045 -2.1962631 2.8969838  H 3.9616205 -0.4919561 2.7172548  C 6.3137775 -2.9405251 2.2862678  O 7.7691965 -3.2222121 0.3650338  O 4.8821425 -2.6708611 4.0980328  H 6.6906465 -3.8048521 2.8193118  C 8.3204825 -4.3678581 0.9763168  C 3.8697385 -1.9453881 4.7672968  H 9.0708825 -4.7485941 0.2838498  H 7.5554595 -5.1360191 1.1439948  H 8.7972385 -4.1197861 1.9322758  H 4.1970745 -0.9222291 4.9872578  H 3.6865975 -2.4772191 5.7010448  H 2.9501365 -1.9140591 4.1720098  H 4.1325505 1.7661179 -3.1847552  C -4.5416195 0.2864709 0.8194048  C -3.5683685 1.2828909 0.7042558  C -3.3187925 2.2148319 1.7109668  C -4.1145745 2.1402629 2.8510438  C -5.0763875 1.1433659 3.0235108  C -5.2681345 0.1797689 2.0200188  H -3.9536815 2.8674329 3.6447728  C -4.6915745 -0.6757581 -0.3140062  C -5.8880575 -0.8354901 -1.0404342  C -3.5555585 -1.3731201 -0.7248772  C -5.9307045 -1.7646701 -2.0909252  C -3.5631405 -2.2758291 -1.7892532  C -4.7764485 -2.4718991 -2.4401492  H -4.8140265 -3.1742481 -3.2704892  C -5.9031125 1.1455199 4.2961278  C -6.5372285 -0.2068711 4.6120928  C -7.2012065 -0.7755131 3.3607778  C -6.1503005 -1.0281531 2.2828038  H -5.2821385 1.4849099 5.1325518  H -5.7653265 -0.9075541 4.9566298  H -7.2605045 -0.0981401 5.4269988  H -7.7302605 -1.7070541 3.5872548  H -7.9491425 -0.0618431 2.9878088  H -5.4938665 -1.8473851 2.6118488  H -6.6186855 -1.3845241 1.3624368  H -6.7015435 1.8930259 4.1862098  C -7.0705595 0.0816029 -0.7796692  C -8.3881235 -0.4770531 -1.3110442  C -8.2243715 -0.8999491 -2.7682032  C -7.2069135 -2.0338171 -2.8665722  H -7.1566895 0.3245929 0.2820678  H -8.6901125 -1.3470151 -0.7112902  H -9.1766995 0.2753089 -1.2068112  H -9.1803055 -1.2187651 -3.1967482  H -7.8780785 -0.0387761 -3.3544372  H -7.6634455 -2.9543281 -2.4756592  H -6.9579715 -2.2390631 -3.9136662  H -6.8605565 1.0376219 -1.2813472  O -2.7961385 1.3257449 -0.4471292  O -2.3545855 -1.1150271 -0.0829012  P -1.5926965 0.2461389 -0.5488932  O -1.1551365 0.2080339 -1.9755242  O -0.5499705 0.4977499 0.5319678  C -2.2268965 3.2129279 1.5512798  C -1.0562785 3.1646399 2.3734918  C -2.3224825 4.1712599 0.5729798  C -0.8800525 2.1714309 3.3744858  C -0.0164555 4.1158649 2.1612008  C -1.2884855 5.1102689 0.3682388  H -3.2051985 4.1980889 -0.0594742  C 0.2469285 2.1465899 4.1544438  H -1.6470725 1.4151829 3.5028148  C 1.1377075 4.0586199 2.9871908  C -0.1415225 5.0948619 1.1248748  H -1.4083805 5.8653229 -0.4053582  C 1.2649365 3.1079179 3.9658508  H 0.3634625 1.3788439 4.9139448  H 1.9396115 4.7702769 2.8231428  H 2.1566795 3.0815719 4.5850578  C -2.2851875 -2.8781131 -2.2540722  C -1.5051785 -3.7339521 -1.4132322  C -1.8012545 -2.5295771 -3.4910712  C -1.9749415 -4.1685271 -0.1461012  C -0.2251765 -4.1727271 -1.8588662  C -0.5309225 -2.9676931 -3.9274562  H -2.3788595 -1.8538051 -4.1153182  C -1.2217385 -5.0009491 0.6408748  H -2.9515045 -3.8355471 0.1917368  C 0.5343555 -5.0248071 -1.0134462  C 0.2675295 -3.7545721 -3.1354562  H -0.1655115 -2.6371951 -4.8959882  C 0.0483445 -5.4350251 0.2013058  H -1.6006275 -5.3282091 1.6045818  H 1.5162435 -5.3558981 -1.3360012  H 0.6462425 -6.0858881 0.8323508  C 0.9706005 6.0732669 0.8548568  H 1.2141755 6.6672459 1.7427448  H 1.8786985 5.5440559 0.5438138  H 0.6893885 6.7630969 0.0545718  C 1.6404885 -4.1678841 -3.5954172  H 2.4082575 -3.8463731 -2.8818122  H 1.7247475 -5.2562771 -3.6937932  H 1.8697365 -3.7225071 -4.5666512 | **TS3-B-*SRR***  C -2.7975477 -1.8170671 2.1568779  O -3.3405177 -3.0025591 1.6977769  C -4.5494947 -2.6723601 1.1363169  C -4.7590247 -1.1813271 1.4107029  N -3.5250777 -0.7770081 2.0691819  C -1.3961027 -1.8687911 2.5814679  C -0.8061757 -0.6765221 2.9899499  C -0.6567817 -3.0386131 2.4306889  C 0.5629423 -0.6484671 3.2192989  H -1.3907837 0.2320889 3.0691039  C 0.7118803 -2.9984441 2.6952529  H -1.1078137 -3.9597891 2.0807479  C 1.3325533 -1.8101851 3.0871699  H 2.4008463 -1.7822211 3.2557989  C -5.9642707 -1.0458301 2.3614479  C -5.7760717 -1.7825231 3.6847569  H -6.1196937 0.0248849 2.5439819  H -6.8466987 -1.4279321 1.8389639  H -6.6569647 -1.6509421 4.3183779  H -4.9053587 -1.4040671 4.2266349  H -5.6431617 -2.8586661 3.5272819  O -5.2615197 -3.4784921 0.6146859  C -3.6394407 0.7392279 -1.7696031  O -2.5850617 0.9627129 -2.4752921  C -4.7903147 1.6183989 -2.1386771  H -5.3617227 1.9266729 -1.2609291  H -5.4712417 1.0481409 -2.7825671  H -4.4202907 2.4961209 -2.6695051  C -3.7424967 -0.2730531 -0.7905601  C -4.9618157 -0.3069051 0.1343879  C -6.2407327 -0.6489221 -0.6179331  C -6.2675157 -1.6509161 -1.5899801  C -7.4136657 0.0650319 -0.3595001  C -7.4423787 -1.9376481 -2.2781431  H -5.3607097 -2.2060941 -1.8137061  C -8.5910727 -0.2188021 -1.0453031  H -7.4008867 0.8573459 0.3874719  C -8.6073477 -1.2233711 -2.0087081  H -7.4461217 -2.7219231 -3.0289471  H -9.4912727 0.3493229 -0.8313401  H -9.5212717 -1.4458931 -2.5506661  C -2.8184497 -1.2771841 -0.8380421  C -1.9072267 -2.1528181 -0.8465391  C -1.6550327 -3.4805581 -1.4131161  C -2.6799877 -4.4309931 -1.5083821  C -0.3629187 -3.8110561 -1.8337101  C -2.4108797 -5.6879881 -2.0374201  H -3.6741827 -4.1896391 -1.1421871  C -0.1071037 -5.0645471 -2.3800531  H 0.4369293 -3.0824301 -1.7344081  C -1.1284997 -6.0056821 -2.4824921  H -3.2076717 -6.4227841 -2.1028761  H 0.8986133 -5.3012551 -2.7164791  H -0.9267657 -6.9866351 -2.9024201  O 1.3845263 -4.1662771 2.5143649  O 1.0830983 0.5564559 3.5637339  C 2.7308553 -4.2275051 2.9384189  H 3.3687143 -3.5506231 2.3577319  H 3.0533533 -5.2538771 2.7617269  H 2.8151673 -3.9904111 4.0063709  C 2.4865293 0.6913019 3.4983499  H 2.6923723 1.7532479 3.6388709  H 2.8681733 0.3663799 2.5232909  H 2.9829273 0.1230719 4.2960759  H -1.7006377 0.5548259 -2.1542471  H -0.8268757 -1.6456721 -0.2461571  H -5.0724377 0.7035759 0.5501319  C 2.5295503 2.6135079 -0.3360191  C 1.1777523 2.4431709 -0.0343911  C 0.2224273 3.4496379 -0.2196741  C 0.7053913 4.6639889 -0.7107221  C 2.0272013 4.8437719 -1.1198921  C 2.9481463 3.7985949 -0.9716631  H 0.0139373 5.4976879 -0.8067911  C 3.4775233 1.5252029 0.0508719  C 4.5396613 1.7766829 0.9476159  C 3.2557763 0.2208329 -0.4068001  C 5.4073693 0.7287369 1.2885939  C 4.1165963 -0.8334141 -0.0842571  C 5.1907053 -0.5410971 0.7534659  H 5.8719713 -1.3492941 1.0147069  C 2.4343603 6.1922409 -1.6844321  C 3.7116113 6.1362719 -2.5192361  C 4.7883643 5.3538359 -1.7717941  C 4.3375683 3.9086369 -1.5741041  H 1.6073433 6.6051349 -2.2726351  H 3.5078923 5.6383439 -3.4760471  H 4.0503363 7.1516059 -2.7497001  H 5.7365453 5.3730549 -2.3187921  H 4.9700763 5.8245489 -0.7949981  H 4.3055153 3.4097339 -2.5536771  H 5.0696763 3.3493889 -0.9864211  H 2.5889893 6.8862029 -0.8459601  C 4.6682413 3.1271419 1.6361489  C 6.0279133 3.3491439 2.2945019  C 6.4154243 2.1391379 3.1383689  C 6.5799743 0.9247089 2.2304829  H 4.4514313 3.9465429 0.9492299  H 6.7912083 3.5070329 1.5200309  H 5.9949793 4.2596849 2.9013389  H 7.3416103 2.3233239 3.6921659  H 5.6278763 1.9473289 3.8803409  H 7.4952033 1.0500499 1.6346109  H 6.7239563 0.0130559 2.8209349  H 3.8828093 3.1847869 2.4040789  O 0.7825493 1.2033499 0.4465829  O 2.1865123 -0.0249281 -1.2491811  P 0.6612813 0.0547259 -0.6977201  O 0.3161223 -1.2140621 0.0730809  O -0.2134337 0.4467209 -1.8381331  C -1.2060567 3.2755929 0.1582029  C -2.2663627 3.8562659 -0.6221631  C -1.5310617 2.6411579 1.3383069  C -2.0652817 4.3659029 -1.9351301  C -3.5839937 3.9164629 -0.0757701  C -2.8629377 2.5879769 1.8032749  H -0.7448787 2.1954429 1.9401269  C -3.0682947 5.0123249 -2.6137581  H -1.1019047 4.2174449 -2.4103811  C -4.5881257 4.6316359 -0.7848861  C -3.8791397 3.2430809 1.1513179  H -3.0803697 2.0395409 2.7157149  C -4.3356977 5.1861379 -2.0132661  H -2.8882097 5.3885099 -3.6161221  H -5.5772997 4.7263499 -0.3473091  H -5.1163357 5.7278769 -2.5386941  C 3.9241603 -2.2251171 -0.5810011  C 4.8931463 -2.8276731 -1.4493701  C 2.8529223 -2.9687171 -0.1517191  C 5.9960073 -2.0988441 -1.9691891  C 4.7398333 -4.1960711 -1.8233091  C 2.7109933 -4.3263741 -0.5222941  H 2.1011453 -2.5142761 0.4875389  C 6.9127213 -2.6930861 -2.7993791  H 6.0994643 -1.0502751 -1.7094491  C 5.7107213 -4.7797641 -2.6799711  C 3.6237733 -4.9497251 -1.3364861  H 1.8558873 -4.8788401 -0.1398531  C 6.7725893 -4.0519481 -3.1546581  H 7.7452453 -2.1156361 -3.1898681  H 5.6046993 -5.8215731 -2.9655471  H 7.5020903 -4.5171071 -3.8107371  C -5.2758047 3.2500079 1.7170229  H -5.5960277 4.2627249 1.9882459  H -6.0114097 2.8654489 0.9981839  H -5.3243897 2.6294619 2.6158229  C 3.4501503 -6.3985641 -1.7119371  H 3.3672103 -6.5252621 -2.7978291  H 4.2990013 -7.0068861 -1.3798071  H 2.5434793 -6.8038951 -1.2554951 |
| **TS3-B-*RSS***  C -4.7025977 0.3979484 0.5805503  C -5.9471297 -0.0945786 1.3577883  C -6.0677987 0.4943794 2.7601813  H -5.8805277 -1.1872666 1.4169633  H -6.8312017 0.1563424 0.7641423  H -6.9896507 0.1459474 3.2333373  H -5.2236347 0.1939754 3.3862983  H -6.1040447 1.5895634 2.7381283  C -2.9483377 -0.7792086 -2.7082287  O -1.8549667 -0.6204726 -3.3796607  C -3.8093127 -1.8682926 -3.2693507  H -4.2956667 -2.4347656 -2.4743237  H -4.6012057 -1.4282866 -3.8863847  H -3.1911537 -2.5291556 -3.8772547  C -3.3193107 -0.0201016 -1.5804087  C -4.5448687 -0.4120996 -0.7444337  C -5.8228997 -0.4083776 -1.5748477  C -6.0872237 0.6032154 -2.5001997  C -6.7387897 -1.4563746 -1.4495267  C -7.2390437 0.5660234 -3.2803817  H -5.3830297 1.4214494 -2.6128587  C -7.8920187 -1.4977626 -2.2279417  H -6.5396277 -2.2518076 -0.7326347  C -8.1436677 -0.4847746 -3.1494077  H -7.4280997 1.3609334 -3.9952197  H -8.5889757 -2.3232186 -2.1192217  H -9.0380857 -0.5148946 -3.7638777  C -2.5043027 1.0244274 -1.2371747  C -1.6078127 1.7714424 -0.7596927  C -1.0721797 3.0954494 -0.4906457  C 0.2097203 3.1980654 0.0603763  C -1.8648067 4.2409994 -0.6424247  C 0.6927643 4.4385094 0.4630963  H 0.8130553 2.3041234 0.1993133  C -1.3784707 5.4736684 -0.2272657  H -2.8703067 4.1465394 -1.0425277  C -0.1038017 5.5731464 0.3327623  H 1.6923083 4.5091254 0.8849743  H -2.0015537 6.3575854 -0.3227487  H 0.2606923 6.5371794 0.6776663  H -4.3786597 -1.4388996 -0.3863467  H -1.0686567 -0.1139106 -2.9460577  H -0.9708337 0.7930614 -0.2617527  C 3.8639513 -1.4355216 -0.7867927  C 3.4633483 -0.1153296 -1.0162297  C 4.3528543 0.8446664 -1.5234837  C 5.5954523 0.3856744 -1.9501917  C 5.9585763 -0.9627006 -1.9117107  C 5.0921583 -1.8857426 -1.3168667  H 6.2997093 1.1127514 -2.3493317  C 3.0145073 -2.3199186 0.0714773  C 3.4738653 -2.8325326 1.3037143  C 1.7015873 -2.5701156 -0.3121987  C 2.6177263 -3.6491116 2.0587753  C 0.8220293 -3.3674106 0.4232633  C 1.3246793 -3.9157586 1.6007553  H 0.6754323 -4.5539636 2.1973283  C 7.3137633 -1.3678056 -2.4606397  C 7.4357283 -2.8680406 -2.7147137  C 6.9179373 -3.6409996 -1.5048657  C 5.4289553 -3.3664806 -1.3087437  H 7.5158723 -0.8042786 -3.3783127  H 6.8430723 -3.1441476 -3.5964907  H 8.4771503 -3.1259686 -2.9326307  H 7.0816513 -4.7166666 -1.6266847  H 7.4740273 -3.3300176 -0.6090927  H 4.8680363 -3.8382476 -2.1285807  H 5.0668513 -3.8449426 -0.3950277  H 8.0867203 -1.0646996 -1.7399587  C 4.8191573 -2.4064946 1.8660653  C 5.3297333 -3.3310296 2.9685673  C 4.2414833 -3.5545196 4.0135623  C 3.0543823 -4.2690886 3.3739863  H 5.5658193 -2.3118016 1.0746223  H 5.6212673 -4.2975956 2.5342893  H 6.2276833 -2.8993716 3.4223233  H 4.6177953 -4.1400996 4.8585813  H 3.9217833 -2.5829376 4.4140023  H 3.3280673 -5.3178086 3.1909593  H 2.2012183 -4.2922816 4.0614783  H 4.6984723 -1.3912336 2.2742943  O 2.2045913 0.2935424 -0.6182537  O 1.2379673 -1.9199266 -1.4431397  P 0.8209243 -0.3758656 -1.1742147  O -0.1259817 -0.2728776 0.0098853  O 0.3601843 0.1834684 -2.4833577  C 4.0982543 2.3141304 -1.4428797  C 3.0618323 2.9864744 -2.1640907  C 4.9157813 3.0499264 -0.6166427  C 2.2116423 2.2920434 -3.0556037  C 2.8718613 4.3881514 -1.9830747  C 4.7364903 4.4426114 -0.4533377  H 5.7000213 2.5444384 -0.0598767  C 1.1716243 2.9286234 -3.6840847  H 2.3720833 1.2338054 -3.2230967  C 1.7993563 5.0219484 -2.6635247  C 3.7360853 5.1178514 -1.1070157  H 5.3996703 4.9851884 0.2159543  C 0.9582523 4.3084094 -3.4806737  H 0.5089103 2.3583764 -4.3268947  H 1.6295563 6.0835794 -2.5150787  H 0.1279083 4.8067664 -3.9718937  C -0.5597957 -3.6457606 -0.0542317  C -1.7048637 -3.4133936 0.7743553  C -0.7441967 -4.1919766 -1.3031607  C -1.6046567 -2.7445436 2.0223813  C -2.9917327 -3.8425726 0.3316263  C -2.0239537 -4.5872596 -1.7480187  H 0.1156263 -4.3470266 -1.9479257  C -2.7038597 -2.5403776 2.8142513  H -0.6345897 -2.3701476 2.3276323  C -4.1080397 -3.6408466 1.1906103  C -3.1369537 -4.4545936 -0.9529127  H -2.1233807 -5.0363756 -2.7332837  C -3.9694857 -3.0110466 2.4024223  H -2.6062277 -1.9973346 3.7496813  H -5.0869697 -3.9935466 0.8768443  H -4.8351687 -2.8619846 3.0420523  C 3.5339953 6.5941654 -0.8927207  H 3.6171333 7.1524644 -1.8316617  H 2.5372663 6.7994364 -0.4832347  H 4.2768963 6.9895134 -0.1957187  C -4.4795367 -4.9525356 -1.4224987  H -4.8593807 -5.7469246 -0.7699617  H -5.2360247 -4.1578806 -1.4326287  H -4.4064087 -5.3541446 -2.4362787  N -3.5147707 0.2836474 1.4252723  C -4.8614337 1.9076254 0.3844933  O -3.9312547 2.4980884 1.1994073  C -3.1668167 1.4682364 1.7271663  O -5.6398157 2.5424284 -0.2637177  C -1.9822017 1.9210144 2.4694043  C -1.8811837 3.2596414 2.8199483  C -0.9350377 1.0124934 2.6843383  C -0.6930607 3.7204544 3.3954043  H -2.6748417 3.9668524 2.6124423  C 0.2403793 1.4957544 3.2501183  H -1.0400637 -0.0081976 2.3347693  C 0.3631643 2.8462454 3.6131853  H 1.3095583 3.1661314 4.0317793  O -0.6562317 5.0473394 3.6809843  O 1.3480633 0.7518654 3.4814413  C 0.5775143 5.5837814 4.1000033  H 1.3584683 5.4089314 3.3489373  H 0.4175833 6.6557174 4.2188353  H 0.8978593 5.1568214 5.0583593  C 1.3327053 -0.6014196 3.0666013  H 2.3315793 -0.9887226 3.2728593  H 0.5981943 -1.1766666 3.6474313  H 1.1121383 -0.6913126 1.9955383 | **TS3-B-*RSR***  C -4.8290774 1.4637301 -0.6792344  C -6.1785604 1.6796131 -1.3930464  C -6.0353044 2.3696021 -2.7471124  H -6.6555894 0.7034231 -1.5188794  H -6.8145604 2.2746261 -0.7271484  H -7.0146384 2.4712391 -3.2217044  H -5.4005704 1.7876321 -3.4253884  H -5.6005254 3.3666191 -2.6416694  C -3.5662134 -0.8024869 2.1837506  O -2.4553954 -1.4173479 2.3979686  C -4.6676604 -1.3329419 3.0508596  H -5.1246284 -2.2018379 2.5662076  H -5.4424444 -0.5919669 3.2416916  H -4.2112174 -1.6672239 3.9853316  C -3.6823234 0.3071901 1.3045266  C -5.0043804 0.8863911 0.7728546  C -2.5072074 0.9916501 1.1673156  C -1.3679754 1.5280411 1.0822086  C -0.5544844 2.3986491 1.9474526  C -0.8810694 3.7429741 2.1392466  C 0.5633546 1.8500151 2.5847106  C -0.1076344 4.5233021 2.9950396  H -1.7399584 4.1614971 1.6214106  C 1.3279896 2.6363101 3.4390286  H 0.8197266 0.8075261 2.4173756  C 0.9880946 3.9699081 3.6540016  H -0.3668464 5.5670051 3.1502886  H 2.1977576 2.2077651 3.9281216  H 1.5895366 4.5808291 4.3216916  H -1.6598774 -1.2424139 1.7716936  H -0.7026534 1.0095511 0.0513456  C 2.7422406 -3.1660159 -0.3730914  C 1.3967196 -3.1192379 -0.7292824  C 0.5235736 -4.1935999 -0.5647764  C 1.0616756 -5.3580649 -0.0260434  C 2.3905366 -5.4376589 0.3966636  C 3.2376056 -4.3281189 0.2538896  H 0.4125126 -6.2229259 0.0990186  C 3.5835736 -1.9572439 -0.6263024  C 4.7389456 -2.0214159 -1.4323354  C 3.1951616 -0.7270339 -0.0800844  C 5.5596796 -0.8909529 -1.5410634  C 3.9895646 0.4180231 -0.1970604  C 5.1830426 0.2903961 -0.9051064  H 5.8210446 1.1677081 -0.9970394  C 2.8804746 -6.7490489 0.9831816  C 4.1411876 -6.6006839 1.8304646  C 5.1679586 -5.7549329 1.0827086  C 4.6258016 -4.3438769 0.8706616  H 2.0744896 -7.2084199 1.5661466  H 3.8950676 -6.1076049 2.7799006  H 4.5450086 -7.5887279 2.0746876  H 6.1134026 -5.7082029 1.6330196  H 5.3833966 -6.2185549 0.1095406  H 4.5573916 -3.8382879 1.8451416  H 5.3267346 -3.7479649 0.2821286  H 3.0905516 -7.4409109 0.1549816  C 5.0377206 -3.2605659 -2.2598384  C 6.4985226 -3.3549709 -2.6931454  C 6.9386596 -2.0446309 -3.3395314  C 6.8634336 -0.9138989 -2.3169624  H 4.7348766 -4.1704119 -1.7374904  H 7.1316386 -3.5620369 -1.8185774  H 6.6232286 -4.1943069 -3.3851934  H 7.9558096 -2.1221889 -3.7372924  H 6.2760626 -1.8231189 -4.1863824  H 7.6902366 -1.0285659 -1.6013604  H 7.0135406 0.0567271 -2.8022284  H 4.4030526 -3.2139629 -3.1569614  O 0.8885936 -1.9157999 -1.2034754  O 2.0276396 -0.6533959 0.6593786  P 0.5601696 -0.8684299 -0.0167494  O 0.0925286 0.3972471 -0.7317714  O -0.3139904 -1.4247079 1.0578786  C -0.9041834 -4.0743309 -0.9610314  C -1.9596454 -4.2400279 -0.0095414  C -1.2206364 -3.8018139 -2.2699844  C -1.6995014 -4.4285979 1.3747866  C -3.3151124 -4.1805419 -0.4546694  C -2.5614224 -3.7209229 -2.6978524  H -0.4207244 -3.6498819 -2.9886434  C -2.7217764 -4.6089039 2.2708396  H -0.6718134 -4.3939149 1.7204106  C -4.3471104 -4.3855219 0.5011556  C -3.6074084 -3.9173719 -1.8305244  H -2.7696234 -3.5012249 -3.7413004  C -4.0622444 -4.6069549 1.8256846  H -2.5020744 -4.7395139 3.3260086  H -5.3806464 -4.3751409 0.1708496  H -4.8691634 -4.7729469 2.5345966  C 3.5931856 1.7506491 0.3381826  C 4.4186466 2.4410211 1.2868286  C 2.4723586 2.3764221 -0.1513864  C 5.5325606 1.8186101 1.9116866  C 4.1077176 3.7900351 1.6362216  C 2.1537856 3.6987361 0.2307686  H 1.8314876 1.8653201 -0.8634694  C 6.3089646 2.4932931 2.8207886  H 5.7553046 0.7840431 1.6703466  C 4.9405566 4.4622821 2.5689576  C 2.9600266 4.4266121 1.0673876  H 1.2393676 4.1468401 -0.1470864  C 6.0147326 3.8346711 3.1486676  H 7.1486136 1.9932651 3.2940096  H 4.7134626 5.4908411 2.8324616  H 6.6342256 4.3654801 3.8653746  C -5.0268094 -3.8306659 -2.3275334  H -5.6173864 -4.7067379 -2.0374704  H -5.5306464 -2.9358899 -1.9442474  H -5.0401434 -3.7634819 -3.4179094  C 2.6306716 5.8622461 1.3820646  H 2.4915126 6.0255361 2.4562776  H 3.4275756 6.5375661 1.0475346  H 1.7009366 6.1497501 0.8816766  N -4.1035924 2.7294071 -0.6259924  C -3.0603574 2.5824911 -1.3361504  C -3.9414124 0.5748631 -1.5574554  O -2.8640164 1.3468711 -1.9197884  C -2.0157704 3.5813491 -1.5919324  C -0.8030034 3.1888391 -2.1520304  C -2.2751094 4.9095101 -1.2555194  C 0.1696876 4.1629331 -2.3835364  H -0.5874774 2.1510161 -2.3825044  C -1.2950464 5.8672691 -1.4988724  H -3.2242594 5.2000391 -0.8198704  C -0.0684834 5.5055111 -2.0699174  H 0.6941306 6.2504471 -2.2521754  O -4.0955324 -0.5436439 -1.9472944  C -6.1802344 -0.0696389 0.8376346  C -7.3723974 0.3549051 1.4265476  C -6.1226724 -1.3488989 0.2765156  C -8.4911974 -0.4745759 1.4580046  H -7.4249644 1.3482001 1.8671666  C -7.2442654 -2.1731519 0.2966126  H -5.1960884 -1.6950889 -0.1756464  C -8.4307234 -1.7413129 0.8872706  H -9.4081754 -0.1271579 1.9242006  H -7.1962534 -3.1583719 -0.1588584  H -9.3012184 -2.3896609 0.9012966  H -5.2654584 1.7721191 1.3658216  O 1.3375836 3.7120721 -2.8994874  O -1.6141954 7.1417411 -1.1550404  C -0.6610934 8.1517231 -1.3986964  H -1.1166154 9.0833351 -1.0632884  H 0.2627936 7.9760741 -0.8333234  H -0.4224824 8.2275331 -2.4666874  C 2.4070146 4.6236881 -3.0344924  H 2.6637216 5.0755301 -2.0682304  H 3.2539306 4.0380941 -3.3913734  H 2.1708786 5.4086201 -3.7640094 |
| **INT4-B-*SRR***  C -3.0130515 -1.5523348 2.2909197  O -3.6605935 -2.6985648 1.8730627  C -4.8416345 -2.2797848 1.3034217  C -4.9330275 -0.7742428 1.5672247  N -3.6566615 -0.4590538 2.1964367  C -1.6069385 -1.7070278 2.6825077  C -0.9072645 -0.5590898 3.0433557  C -0.9724975 -2.9406288 2.5611817  C 0.4601145 -0.6485688 3.2684477  H -1.4069285 0.4005322 3.1004687  C 0.3959145 -3.0159688 2.8189227  H -1.5079145 -3.8295928 2.2485147  C 1.1199165 -1.8797508 3.1851837  H 2.1861755 -1.9402098 3.3599567  C -6.1035845 -0.5346808 2.5393277  C -5.9537135 -1.2814948 3.8618347  H -6.1653815 0.5457182 2.7212707  H -7.0247335 -0.8411328 2.0340897  H -6.8065545 -1.0710278 4.5121847  H -5.0425615 -0.9790298 4.3848417  H -5.9185975 -2.3652918 3.7056467  O -5.6143965 -3.0305448 0.7881507  C -3.5893135 1.0305352 -1.6540693  O -2.4509685 1.2042812 -2.0601003  C -4.7089005 1.8916552 -2.1752223  H -5.2714695 2.3497222 -1.3561263  H -5.4159585 1.2667792 -2.7326753  H -4.2879685 2.6683042 -2.8142073  C -3.9123065 -0.0683048 -0.6840653  C -5.0799095 0.0996912 0.2836747  C -6.4293755 -0.0493928 -0.4031003  C -6.6610515 -1.0566468 -1.3415243  C -7.4605125 0.8483952 -0.1142253  C -7.8980595 -1.1683478 -1.9690063  H -5.8609085 -1.7465128 -1.5900763  C -8.6988355 0.7403562 -0.7402093  H -7.2883045 1.6422992 0.6104327  C -8.9204565 -0.2712798 -1.6709403  H -8.0609835 -1.9573068 -2.6966393  H -9.4867725 1.4498492 -0.5061953  H -9.8832865 -0.3571258 -2.1650913  C -3.1449905 -1.1346978 -0.7425953  C -2.2917395 -2.1245348 -0.7562233  C -2.3539585 -3.3907738 -1.5049153  C -3.5584515 -3.9845318 -1.8909173  C -1.1429125 -4.0249788 -1.8034793  C -3.5473435 -5.1873628 -2.5887783  H -4.4995205 -3.5250558 -1.6006373  C -1.1360405 -5.2235348 -2.5073353  H -0.2114695 -3.5620758 -1.4844073  C -2.3385055 -5.8055758 -2.9040703  H -4.4859615 -5.6502618 -2.8781513  H -0.1894775 -5.7014328 -2.7436363  H -2.3354735 -6.7442978 -3.4498323  O 0.9616595 -4.2438608 2.6629517  O 1.0948405 0.5159182 3.5558007  C 2.2887625 -4.4238468 3.1127227  H 2.9998875 -3.8312758 2.5251077  H 2.5108675 -5.4819428 2.9722787  H 2.3805275 -4.1638468 4.1745687  C 2.4985415 0.5353922 3.4017247  H 2.7862905 1.5871402 3.4288077  H 2.7942205 0.0981682 2.4403167  H 3.0007775 0.0028612 4.2199357  H -0.9000585 0.6064602 -1.7086873  H -1.4015935 -1.9798058 -0.1383493  H -5.0130775 1.1251122 0.6735407  C 2.9861945 2.4035162 -0.2433023  C 1.6168865 2.3626242 0.0278557  C 0.7571145 3.4416152 -0.2043063  C 1.3648195 4.6112432 -0.6653073  C 2.7129635 4.6771052 -1.0160863  C 3.5292575 3.5487872 -0.8562663  H 0.7516815 5.5004702 -0.7903203  C 3.8124205 1.2119012 0.1189797  C 4.8939125 1.3087282 1.0208157  C 3.4467175 -0.0449478 -0.3756013  C 5.6322115 0.1546282 1.3265167  C 4.1561585 -1.2089118 -0.0684033  C 5.2608535 -1.0693258 0.7680927  H 5.8354895 -1.9604418 1.0144337  C 3.2616825 5.9918532 -1.5399113  C 4.5584505 5.8357542 -2.3310833  C 5.5289675 4.9343562 -1.5719643  C 4.9347085 3.5354332 -1.4301093  H 2.4972185 6.4895172 -2.1466763  H 4.3449055 5.3858782 -3.3092973  H 4.9998585 6.8199352 -2.5184913  H 6.4929195 4.8761282 -2.0878103  H 5.7208165 5.3597132 -0.5764873  H 4.8726635 3.0740462 -2.4264943  H 5.5952795 2.8875442 -0.8493233  H 3.4481435 6.6527202 -0.6813923  C 5.1876185 2.6136772 1.7453337  C 6.5855215 2.6654122 2.3561487  C 6.8511105 1.4038052 3.1707747  C 6.8385875 0.1902892 2.2463567  H 5.0290895 3.4744362 1.0938907  H 7.3354865 2.7463322 1.5569597  H 6.6797135 3.5626072 2.9764207  H 7.8098085 1.4655782 3.6955997  H 6.0699095 1.2968222 3.9357987  H 7.7474565 0.2063962 1.6285357  H 6.8795915 -0.7393518 2.8245637  H 4.4461725 2.7246642 2.5497387  O 1.0935855 1.1774312 0.5273267  O 2.3464855 -0.1333648 -1.2179183  P 0.8839315 -0.0201228 -0.5412743  O 0.3877385 -1.2132558 0.1603147  O 0.0939165 0.5496492 -1.7661773  C -0.7073205 3.3774142 0.0560597  C -1.6448335 4.0299992 -0.8206993  C -1.1893305 2.7657922 1.1935237  C -1.2877935 4.5000212 -2.1139413  C -2.9994965 4.1903902 -0.4005913  C -2.5575265 2.8256042 1.5385787  H -0.5006935 2.2616732 1.8641377  C -2.1795335 5.1888312 -2.8970743  H -0.2969375 4.2810312 -2.4960033  C -3.8830925 4.9517002 -1.2131633  C -3.4526245 3.5668302 0.8049107  H -2.8971465 2.3072932 2.4318047  C -3.4833425 5.4542342 -2.4251353  H -1.8820955 5.5261892 -3.8850813  H -4.9008085 5.1177622 -0.8727663  H -4.1751755 6.0285662 -3.0339083  C 3.7486945 -2.5545648 -0.5677463  C 4.5164875 -3.2343508 -1.5661863  C 2.6496235 -3.1747138 -0.0268243  C 5.6468935 -2.6350968 -2.1823903  C 4.1285135 -4.5468898 -1.9691423  C 2.2736105 -4.4784698 -0.4254993  H 2.0458245 -2.6541388 0.7093887  C 6.3672095 -3.3004788 -3.1421553  H 5.9302665 -1.6290478 -1.8888483  C 4.8994205 -5.2064218 -2.9626143  C 2.9841425 -5.1701768 -1.3750873  H 1.4045025 -4.9317298 0.0469237  C 5.9910495 -4.6033688 -3.5352193  H 7.2249015 -2.8230368 -3.6060723  H 4.6150725 -6.2058118 -3.2761223  H 6.5633595 -5.1250938 -4.2962013  C -4.8717485 3.7426442 1.2820237  H -5.0771895 4.7893532 1.5350357  H -5.6096695 3.4498502 0.5243707  H -5.0492105 3.1400342 2.1775757  C 2.5697595 -6.5603698 -1.7822093  H 2.3461825 -6.6153178 -2.8539763  H 3.3598575 -7.2922378 -1.5795283  H 1.6756395 -6.8673088 -1.2339183 | **Pro-*SRR***  C 0.7689514 -1.0157356 -0.7747051  O 0.3113294 0.0366104 -1.5378861  C -1.0645996 -0.0628416 -1.5341421  C -1.3961136 -1.3720746 -0.8087871  N -0.0929906 -1.8400106 -0.3389131  C 2.2113444 -1.0089376 -0.4908061  C 2.7857264 -2.1299076 0.1018849  C 2.9539824 0.1390174 -0.7581371  C 4.1399184 -2.0984806 0.4244949  H 2.1944194 -3.0119046 0.3183579  C 4.3038844 0.1609614 -0.4093021  H 2.5019574 1.0121024 -1.2151651  C 4.9095734 -0.9548506 0.1761999  H 5.9584314 -0.9348946 0.4380069  C -1.9749976 -2.3530426 -1.8505531  C -1.0247636 -2.6486316 -3.0075061  H -2.2285556 -3.2787526 -1.3206431  H -2.9075166 -1.9206436 -2.2260581  H -1.4909266 -3.3463836 -3.7077911  H -0.0935396 -3.0966556 -2.6502741  H -0.7779336 -1.7389666 -3.5663541  O -1.7606686 0.7329484 -2.0871931  C -2.6672176 -0.2803656 2.7512809  O -3.4033926 -1.2154816 2.9774849  C -2.4675416 0.8488044 3.7353369  H -2.7914976 1.7940794 3.2875469  H -1.4088466 0.9564584 3.9880029  H -3.0494226 0.6436554 4.6337799  C -1.9089286 -0.2260666 1.4499269  C -2.3589256 -1.2338236 0.4021599  C -3.8089406 -0.9793236 0.0148189  C -4.2459816 0.2813754 -0.3964901  C -4.7330516 -2.0236106 0.0820929  C -5.5758556 0.4849214 -0.7542591  H -3.5395616 1.1029544 -0.4550041  C -6.0626516 -1.8224946 -0.2739791  H -4.4062646 -2.9983906 0.4352149  C -6.4871716 -0.5655656 -0.6968961  H -5.8996116 1.4691734 -1.0787991  H -6.7686366 -2.6449806 -0.2106461  H -7.5248236 -0.4035686 -0.9726371  C -0.9007206 0.6025274 1.3151009  C 0.1407394 1.3868684 1.1697299  C 0.1346294 2.7224634 0.5475419  C 1.3505574 3.3736114 0.3162869  C -1.0572416 3.3453554 0.1599579  C 1.3746044 4.6238584 -0.2938361  H 2.2806264 2.8895604 0.6065399  C -1.0311136 4.5932834 -0.4464241  H -2.0033776 2.8405484 0.3387239  C 0.1849994 5.2371114 -0.6752161  H 2.3251054 5.1170904 -0.4722931  H -1.9613366 5.0655554 -0.7466241  H 0.2034334 6.2121024 -1.1522831  O 4.9583644 1.3222914 -0.6687681  O 4.6384174 -3.2267226 0.9890799  C 6.3293374 1.3960874 -0.3455681  H 6.4940924 1.2592904 0.7303529  H 6.6544454 2.3945384 -0.6370481  H 6.9104564 0.6486664 -0.8994331  C 6.0022464 -3.2440126 1.3468149  H 6.1875694 -4.2274656 1.7780569  H 6.2291974 -2.4719466 2.0923399  H 6.6471964 -3.1035066 0.4706149  H 1.1108544 0.9992334 1.4871969  H -2.3216736 -2.2130656 0.8954959 |
|  | **(*S*)-B2**  C -0.7440890 1.5009730 -0.1286883  C -1.4494120 0.3939380 0.3472917  C -2.8351800 0.2802310 0.2497017  C -3.5058530 1.3344630 -0.3677533  C -2.8377300 2.4298020 -0.9159793  C -1.4395510 2.5093490 -0.8221043  H -4.5897810 1.2791110 -0.4490383  C 0.7371930 1.5219950 0.0580197  C 1.4003500 2.5165480 0.8020077  C 1.4750060 0.4572150 -0.4589033  C 2.7986630 2.4633240 0.9154907  C 2.8586120 0.3592700 -0.3236753  C 3.4992390 1.3981740 0.3472037  H 4.5813190 1.3555090 0.4571277  C -3.6550160 3.5164490 -1.5890053  C -2.8403720 4.3677210 -2.5595133  C -1.5420850 4.8121010 -1.8915623  C -0.6796130 3.5958930 -1.5631023  H -4.5117250 3.0611640 -2.0977493  H -2.6008940 3.7824970 -3.4569043  H -3.4312890 5.2300640 -2.8845133  H -0.9821570 5.4958050 -2.5377883  H -1.7767950 5.3601100 -0.9683583  H -0.3110440 3.1552210 -2.5008953  H 0.2150540 3.8970580 -1.0129093  H -4.0731140 4.1697020 -0.8100713  C 0.6086550 3.5578180 1.5739997  C 1.4386040 4.7836170 1.9476377  C 2.7390530 4.3504780 2.6186187  C 3.5835450 3.5437720 1.6353757  H -0.2900610 3.8545010 1.0284237  H 1.6706760 5.3632210 1.0432717  H 0.8554940 5.4353020 2.6061937  H 3.3070630 5.2166150 2.9731297  H 2.5026380 3.7367950 3.4976507  H 4.0006990 4.2281530 0.8831397  H 4.4415410 3.0897320 2.1433077  H 0.2461780 3.0771960 2.4944897  O -0.7261990 -0.6519700 0.9205427  O 0.7893970 -0.5742190 -1.0911833  P 0.0266950 -1.6223790 -0.1215513  O 1.1369770 -2.1710110 0.8872117  H 1.6192990 -2.9307590 0.5228467  O -0.7497660 -2.5983390 -0.8847973  C -3.5595170 -0.8983690 0.7963427  C -4.3390990 -1.7503170 -0.0499543  C -3.5000850 -1.1611510 2.1433317  C -4.3764030 -1.5831970 -1.4600763  C -5.0771820 -2.8225300 0.5331617  C -4.2218020 -2.2327600 2.7110537  H -2.8943700 -0.5252640 2.7825867  C -5.1327150 -2.4091770 -2.2513513  H -3.7743670 -0.8018450 -1.9118073  C -5.8573140 -3.6521140 -0.3161153  C -5.0113390 -3.0526960 1.9436297  H -4.1553880 -2.4044770 3.7822237  C -5.8915610 -3.4495580 -1.6721863  H -5.1392400 -2.2708230 -3.3280833  H -6.4292170 -4.4651870 0.1195587  H -6.4919410 -4.0971200 -2.3039583  C 3.6013620 -0.8115570 -0.8647273  C 4.2843920 -1.7207910 0.0072227  C 3.6359950 -1.0315610 -2.2204423  C 4.2174420 -1.6001670 1.4228217  C 5.0222430 -2.8057790 -0.5547533  C 4.3618610 -2.1112510 -2.7689123  H 3.0983290 -0.3573570 -2.8807883  C 4.8705580 -2.4873220 2.2403217  H 3.6173170 -0.8044250 1.8517197  C 5.6958110 -3.6972560 0.3234597  C 5.0587380 -2.9871600 -1.9737123  H 4.3706130 -2.2463500 -3.8471193  C 5.6278130 -3.5424300 1.6846177  H 4.7996340 -2.3827760 3.3185677  H 6.2681130 -4.5189710 -0.0950613  H 6.1469650 -4.2372570 2.3377397  C -5.7831970 -4.1821280 2.5737887  H -6.8598180 -4.0806100 2.3976707  H -5.4761730 -5.1523450 2.1677567  H -5.6190020 -4.2023700 3.6536897  C 5.8354210 -4.1251370 -2.5820923  H 6.8991330 -4.0665030 -2.3262193  H 5.4685370 -5.0954190 -2.2296613  H 5.7492320 -4.1087010 -3.6708783 |
| **INT0’-C**  C -3.9270601 0.8293165 0.0400250  O -3.2516961 0.3545285 -1.0813410  C -1.9340361 0.6009045 -0.8540100  C -1.8354741 1.2655045 0.5054640  C -5.3834661 0.6355595 0.0256230  C -6.1205671 1.0805415 1.1205960  C -5.9935111 0.0104515 -1.0591500  C -7.4999981 0.8955795 1.1216250  H -5.6337641 1.5638575 1.9594320  C -7.3768541 -0.1678465 -1.0414380  H -5.4238751 -0.3268835 -1.9168380  C -8.1403891 0.2705185 0.0438110  H -9.2119991 0.1276745 0.0523530  O -1.0944611 0.3374995 -1.6796160  N -3.2200991 1.3544065 0.9504330  C -1.1277061 2.6239695 0.4230780  C -1.8311931 3.6085245 -0.5051150  H -0.1002681 2.4410065 0.0930530  H -1.0685781 3.0224795 1.4397900  H -1.2873501 4.5561995 -0.5405590  H -1.8823361 3.2224155 -1.5303360  H -2.8511301 3.8107975 -0.1641440  O -7.9029681 -0.7820405 -2.1308990  O -8.1563401 1.3537065 2.2170880  C -9.3009921 -0.9605815 -2.1804740  H -9.5089961 -1.4445645 -3.1344400  H -9.6510901 -1.6030235 -1.3631480  H -9.8270121 0.0010265 -2.1375810  C -9.5556351 1.1890825 2.2704140  H -9.8345391 0.1285135 2.2442000  H -9.8740391 1.6220575 3.2184980  H -10.0520601 1.7154465 1.4460140  H 0.5683639 0.6858715 -1.5034880  O 1.5155569 0.8161905 -1.2320340  P 1.8136379 0.0629395 0.1177630  O 3.3112539 0.5022965 0.5075690  O 1.9793139 -1.4629395 -0.4072040  O 0.8919999 0.2426655 1.2467730  C 4.3141739 0.4028285 -0.4504330  C 2.3110999 -2.3763905 0.5873310  C 4.9696009 -0.8070635 -0.6296740  C 4.6152129 1.5243795 -1.2369940  C 3.6513499 -2.5847835 0.8737190  C 1.2967619 -3.0006815 1.3262710  C 4.8994819 -2.1086875 0.1532050  C 5.8932669 -0.9290685 -1.6736490  C 5.5836379 1.3801085 -2.2360910  C 3.8696479 2.8052605 -1.0953710  C 4.0012469 -3.3756125 1.9721100  C 1.6834199 -3.8347235 2.3815880  C -0.1455911 -2.7227405 1.0822930  C 5.3350649 -3.1177345 -0.9470580  C 5.9215389 -2.1349845 1.3245670  C 6.2119679 0.1598775 -2.4724650  C 6.3834939 -2.3550305 -1.7762490  H 5.8231069 2.2453715 -2.8473680  C 3.8178079 3.5288285 0.1388910  C 3.2034889 3.2946685 -2.1971500  C 3.0225739 -4.0124075 2.7226130  C 5.4985789 -3.3451195 2.1796150  H 0.9045349 -4.3254985 2.9579620  C -0.7621711 -2.9305085 -0.1934560  C -0.9006081 -2.2425405 2.1309860  H 4.4649729 -3.3457835 -1.5728850  H 5.7063399 -4.0565725 -0.5249850  H 5.8060759 -1.2139785 1.9069470  H 6.9560399 -2.1874675 0.9714980  H 6.9317499 0.0616015 -3.2801330  H 6.4419159 -2.7029655 -2.8117550  H 7.3862749 -2.4591825 -1.3424710  C 4.5273899 3.1226275 1.3012960  C 3.0401899 4.7210385 0.2026110  C 2.4410009 4.4811095 -2.1314060  H 3.2305789 2.7243395 -3.1211640  H 3.2917529 -4.6277925 3.5763320  H 5.7714089 -3.2353005 3.2333370  H 5.9658049 -4.2706915 1.8196890  C -0.0609521 -3.4464115 -1.3172010  C -2.1453681 -2.6226145 -0.3445410  C -2.2726211 -1.9446015 1.9777440  H -0.4129561 -2.0499965 3.0819720  C 4.4512169 3.8460175 2.4631090  H 5.1389429 2.2270105 1.2593180  C 2.9763459 5.4443025 1.4233060  C 2.3525129 5.1740145 -0.9529060  H 1.9161069 4.8289625 -3.0158010  C -0.6888491 -3.6125625 -2.5240410  H 0.9861129 -3.7104795 -1.2100680  C -2.7663611 -2.8021615 -1.6095600  C -2.8822971 -2.1307325 0.7638420  H -2.8343351 -1.5501445 2.8191900  C 3.6613989 5.0183965 2.5299130  H 5.0005139 3.5188865 3.3405300  H 2.3737359 6.3484655 1.4577810  H 1.7612439 6.0840075 -0.8855070  C -2.0560291 -3.2797055 -2.6777090  H -0.1338891 -4.0022535 -3.3720330  H -3.8187891 -2.5471695 -1.7079970  H -3.9381241 -1.9033915 0.6281820  H 3.6061279 5.5789555 3.4580370  H -2.5366991 -3.4085405 -3.6425210  H -1.2618161 0.6093685 1.1730660 | **TS1’-C**  C -3.4851115 1.0196157 -0.2031287  O -2.9485325 0.6333057 -1.4328067  C -1.6722935 1.0072297 -1.3869797  C -1.3976945 1.5888927 -0.1078157  C -4.9012485 0.7165107 0.0144843  C -5.4424985 0.9699127 1.2731453  C -5.6788645 0.1980647 -1.0189867  C -6.7905245 0.6998167 1.4936513  H -4.8302775 1.3738817 2.0710823  C -7.0270505 -0.0660993 -0.7807837  H -5.2645865 0.0012137 -2.0012147  C -7.5955015 0.1806077 0.4724463  H -8.6408885 -0.0285073 0.6511243  O -0.9332445 0.7190787 -2.3787867  N -2.6530435 1.5880927 0.5739043  C -0.5036615 2.8158917 0.0502803  C -1.2351725 4.1072817 -0.3065127  H 0.3813445 2.6873587 -0.5837097  H -0.1466115 2.8495707 1.0851923  H -0.6007025 4.9750937 -0.1045207  H -1.5066515 4.1228877 -1.3680817  H -2.1506815 4.2049367 0.2840823  O -7.7211095 -0.5662413 -1.8343257  O -7.2483425 0.9694317 2.7420353  C -9.0904075 -0.8492773 -1.6514077  H -9.4501965 -1.2289893 -2.6073257  H -9.2406465 -1.6121023 -0.8777257  H -9.6523675 0.0540687 -1.3844417  C -8.6077905 0.7182067 3.0193153  H -8.8525025 -0.3431983 2.8891703  H -8.7595845 0.9981427 4.0615243  H -9.2636015 1.3229077 2.3810683  H 0.1110505 0.7358427 -2.0914617  O 1.3653765 0.6272897 -1.5517417  P 1.5700935 -0.0667143 -0.2306167  O 2.9039875 0.4459927 0.5230093  O 1.9035355 -1.6139553 -0.5684477  O 0.4703265 0.0342277 0.8129973  C 4.0826235 0.4036317 -0.2119007  C 2.0840575 -2.4466373 0.5305753  C 4.8373045 -0.7609673 -0.2149467  C 4.4524335 1.5199697 -0.9750367  C 3.3533235 -2.5704933 1.0740713  C 0.9739385 -3.0833613 1.1047533  C 4.6922715 -2.0429023 0.5887863  C 5.9511595 -0.8468043 -1.0561327  C 5.6047785 1.4179117 -1.7622537  C 3.6126995 2.7476197 -1.0437857  C 3.5212835 -3.2940713 2.2585293  C 1.1873555 -3.8461793 2.2577583  C -0.4046515 -2.8592203 0.5902933  C 5.3970585 -3.0492353 -0.3652927  C 5.4649725 -1.9692883 1.9367043  C 6.3463545 0.2406057 -1.8228327  C 6.5389235 -2.2384543 -1.0044787  H 5.8994045 2.2792467 -2.3546427  C 3.2529825 3.5033797 0.1183603  C 3.1769045 3.1633757 -2.2832097  C 2.4459405 -3.9446403 2.8474303  C 4.9483505 -3.1754383 2.7431143  H 0.3334545 -4.3423043 2.7100393  C -0.7673495 -3.1226213 -0.7692597  C -1.3416755 -2.3342333 1.4538683  H 4.6835205 -3.3535523 -1.1391547  H 5.7355835 -3.9482473 0.1590393  H 5.1874745 -1.0400823 2.4469013  H 6.5498875 -1.9685193 1.7930333  H 7.2128825 0.1737427 -2.4745877  H 6.8217445 -2.6093833 -1.9940337  H 7.4415215 -2.2610583 -0.3805437  C 3.6860765 3.1607697 1.4279413  C 2.4383165 4.6621747 -0.0418697  C 2.3785265 4.3175527 -2.4376597  H 3.4270245 2.5636347 -3.1532847  H 2.5751515 -4.5057493 3.7687233  H 5.0111625 -3.0214663 3.8243573  H 5.5203485 -4.0831333 2.5119113  C 0.1274955 -3.7034373 -1.7085317  C -2.0858025 -2.7993603 -1.2016177  C -2.6504005 -2.0240063 1.0234343  H -1.0431555 -2.0996803 2.4714373  C 3.3027815 3.9023837 2.5154683  H 4.3297985 2.2975297 1.5616693  C 2.0582985 5.4095037 1.1050903  C 2.0160105 5.0517007 -1.3393857  H 2.0488115 4.6109957 -3.4293947  C -0.2581825 -3.9265093 -3.0045937  H 1.1282845 -3.9718363 -1.3852027  C -2.4514695 -3.0328883 -2.5541857  C -3.0128715 -2.2491503 -0.2795357  H -3.3592325 -1.5879563 1.7219333  C 2.4715995 5.0365867 2.3568103  H 3.6395865 3.6187827 3.5076823  H 1.4341165 6.2894487 0.9662993  H 1.3980075 5.9405907 -1.4421527  C -1.5607235 -3.5808473 -3.4378987  H 0.4411385 -4.3684493 -3.7076587  H -3.4567725 -2.7657133 -2.8705637  H -4.0135735 -2.0033413 -0.6273427  H 2.1729435 5.6128707 3.2270223  H -1.8487035 -3.7541673 -4.4702547  H -0.5554285 0.6711087 0.4124933 |
| **INT1’-C**  C -3.5436617 1.2714314 -0.4207489  O -3.0380377 0.8677284 -1.6176659  C -1.7738117 1.3579124 -1.6439479  C -1.5466527 2.0400764 -0.4771909  C -4.9233507 0.9110554 -0.0960219  C -5.3851367 1.1512704 1.1974351  C -5.7559727 0.3477464 -1.0627449  C -6.6978827 0.8198704 1.5216631  H -4.7362027 1.5963454 1.9428981  C -7.0671447 0.0209954 -0.7208119  H -5.4091007 0.1665234 -2.0739749  C -7.5527037 0.2506914 0.5700351  H -8.5705277 -0.0064086 0.8270341  O -1.0851967 1.1067134 -2.7448419  N -2.7072317 1.9511614 0.2919881  C -0.4125137 2.9483904 -0.0999579  C -0.8841177 4.4038584 -0.0404599  H 0.3976143 2.8380004 -0.8303919  H -0.0011807 2.6604084 0.8750871  H -0.0847597 5.0542234 0.3258641  H -1.1907207 4.7516244 -1.0320119  H -1.7413687 4.4939944 0.6327351  O -7.8144637 -0.5225796 -1.7171309  O -7.0727977 1.0814644 2.8010231  C -9.1518877 -0.8625286 -1.4311199  H -9.5642997 -1.2670046 -2.3553729  H -9.2123207 -1.6234606 -0.6430619  H -9.7323797 0.0179654 -1.1292549  C -8.3951207 0.7752304 3.1795941  H -8.6021197 -0.2977096 3.0804051  H -8.4834527 1.0638894 4.2269021  H -9.1228747 1.3405934 2.5843031  H -0.1386747 0.9701964 -2.4972989  O 1.2932733 0.5768674 -1.6452049  P 1.5444793 -0.0908236 -0.3534479  O 2.8573853 0.3616864 0.4552681  O 1.7527533 -1.6680686 -0.5870219  O 0.4778163 0.0972784 0.7937591  C 4.0438243 0.2461314 -0.2746879  C 1.9055433 -2.4707516 0.5440981  C 4.7320533 -0.9565276 -0.2606149  C 4.4650153 1.3299494 -1.0548179  C 3.1691393 -2.6416366 1.0852271  C 0.7671443 -3.0345836 1.1369921  C 4.5311283 -2.2060786 0.5770771  C 5.8283193 -1.1188316 -1.1138229  C 5.5951923 1.1497294 -1.8586679  C 3.7163563 2.6163084 -1.0834469  C 3.3026803 -3.3339516 2.2931751  C 0.9426543 -3.7647046 2.3163551  C -0.5914027 -2.7665606 0.5932951  C 5.1727993 -3.2729606 -0.3545549  C 5.3176383 -2.1338966 1.9172001  C 6.2666813 -0.0702696 -1.9103859  C 6.3439993 -2.5378196 -1.0332119  H 5.9294163 1.9846444 -2.4674619  C 3.5196433 3.3970284 0.1007751  C 3.2265093 3.0702474 -2.2877119  C 2.1970683 -3.9052586 2.9075001  C 4.7374983 -3.2802686 2.7664031  H 0.0654803 -4.2022976 2.7840751  C -0.9466397 -3.1000166 -0.7526799  C -1.5064407 -2.1268836 1.4025991  H 4.4348663 -3.5658776 -1.1097149  H 5.4755423 -4.1705126 0.1933301  H 5.1008383 -1.1734136 2.3982591  H 6.3994013 -2.2026426 1.7665671  H 7.1155163 -0.1982416 -2.5760919  H 6.5901083 -2.9494946 -2.0163069  H 7.2547253 -2.5896056 -0.4229809  C 4.0146353 3.0068134 1.3745151  C 2.8163343 4.6319584 -0.0016239  C 2.5295383 4.2958134 -2.3834909  H 3.3546233 2.4530894 -3.1722959  H 2.3009443 -4.4397106 3.8475151  H 4.8165843 -3.0938046 3.8413521  H 5.2541923 -4.2269786 2.5632361  C -0.0795507 -3.8105286 -1.6263869  C -2.2295607 -2.7151546 -1.2353829  C -2.7814087 -1.7544046 0.9209971  H -1.2134417 -1.8569396 2.4136401  C 3.8027853 3.7845294 2.4832491  H 4.5715153 2.0795594 1.4640301  C 2.6148063 5.4158454 1.1656531  C 2.3303853 5.0603984 -1.2645409  H 2.1483933 4.6204884 -3.3465029  C -0.4597687 -4.1015196 -2.9104859  H 0.8915973 -4.1299276 -1.2609559  C -2.5877827 -3.0178236 -2.5763219  C -3.1298927 -2.0372216 -0.3748569  H -3.4741807 -1.2259026 1.5700861  C 3.0898493 5.0026854 2.3822561  H 4.1856363 3.4651094 3.4475721  H 2.0753763 6.3553674 1.0710411  H 1.7907323 6.0024644 -1.3236669  C -1.7249207 -3.6933976 -3.3972339  H 0.2147173 -4.6475946 -3.5629679  H -3.5631157 -2.6989066 -2.9344909  H -4.1014397 -1.7414366 -0.7616289  H 2.9262373 5.6078784 3.2685681  H -2.0063647 -3.9199516 -4.4209109  H -0.4054137 0.3516394 0.4479571 | **INT2’-C**  C 2.7920002 1.9063588 -0.5440369  O 2.0421642 2.1968528 0.5488101  C 0.7731662 2.3018248 0.0831851  C 0.7842742 2.0834318 -1.2658629  N 2.0956172 1.8226908 -1.6366129  C 4.2310962 1.7336848 -0.3613939  C 5.0287282 1.4937798 -1.4804409  C 4.7899702 1.7742038 0.9168711  C 6.3952702 1.2920278 -1.3109059  H 4.5997662 1.4773438 -2.4760139  C 6.1603502 1.5741528 1.0647581  H 4.1806662 1.9574448 1.7949791  C 6.9803982 1.3413248 -0.0419299  H 8.0365732 1.1487508 0.0874561  C -0.3441798 2.1393218 -2.2462469  C 0.0057032 3.0283488 -3.4407269  H -1.2391818 2.5143548 -1.7349619  H -0.5828848 1.1298538 -2.6033649  H -0.8174408 3.0579008 -4.1607409  H 0.2116232 4.0517698 -3.1097689  H 0.9016722 2.6511908 -3.9414999  O -0.1392048 2.5981688 0.9958321  C 1.3407022 -1.1706262 -1.6873689  O 0.1694592 -1.0942562 -2.0385289  C 2.4182502 -1.3379162 -2.7222829  H 1.9527262 -1.4863552 -3.6966529  H 3.0279872 -0.4273422 -2.7263219  H 3.0676192 -2.1796932 -2.4712259  C 1.7518672 -1.0469442 -0.2539799  C 0.8158102 -0.6533972 0.6463321  C 0.9310422 -0.3860902 2.0742921  C -0.2648018 -0.2286672 2.7944361  C 2.1522582 -0.2633502 2.7550031  C -0.2420928 -0.0034682 4.1645961  H -1.2114828 -0.2863202 2.2623321  C 2.1695452 -0.0258232 4.1234561  H 3.0848102 -0.3276452 2.2068461  C 0.9760022 0.0932308 4.8341591  H -1.1752098 0.1092638 4.7078901  H 3.1211132 0.0766068 4.6367001  H 0.9963352 0.2772608 5.9042861  H -0.1854768 -0.5173642 0.2456121  C 3.1161632 -1.3388252 0.0311951  C 4.3007242 -1.5661832 0.1621821  C 5.7064782 -1.7704612 0.3109061  C 6.3074452 -1.7070812 1.5762801  C 6.5022222 -2.0035752 -0.8203289  C 7.6795452 -1.8849682 1.7041161  H 5.6913462 -1.5058862 2.4474231  C 7.8730512 -2.1805442 -0.6834909  H 6.0353962 -2.0273292 -1.8004039  C 8.4668082 -2.1193212 0.5770801  H 8.1366272 -1.8417542 2.6885791  H 8.4804562 -2.3711742 -1.5636429  H 9.5379452 -2.2644402 0.6822611  O 6.6271192 1.5985138 2.3442991  O 7.1019472 1.0332668 -2.4455529  C 8.0245202 1.5908878 2.5301841  H 8.4982032 2.4318978 2.0086081  H 8.1870872 1.6918768 3.6038921  H 8.4714932 0.6520798 2.1811721  C 8.5071472 0.9874688 -2.3451519  H 8.8779242 0.8354978 -3.3593869  H 8.9078432 1.9283388 -1.9477059  H 8.8375782 0.1576078 -1.7085109  H -1.3477168 -0.9764272 -1.5527849  O -2.3510868 -0.9504682 -1.4872989  P -2.9840438 -0.1078802 -0.3371239  O -3.4821678 -1.1403272 0.8076601  O -4.3492798 0.3568928 -1.0530599  O -2.1844308 0.9551938 0.3018861  C -4.1308868 -2.2899012 0.3559481  C -5.2459668 1.0314348 -0.2218319  C -5.4959368 -2.2476762 0.1131241  C -3.3779928 -3.4397702 0.0891971  C -6.1847938 0.2954978 0.4874251  C -5.1434388 2.4191428 -0.0749059  C -6.5261358 -1.1812082 0.4326381  C -6.1162228 -3.3456562 -0.4901939  C -4.0401758 -4.5431692 -0.4611839  C -1.9269728 -3.5179382 0.4140481  C -7.0086518 0.9465978 1.4087821  C -6.0208068 3.0478428 0.8175921  C -4.1600438 3.2268098 -0.8471789  C -7.6000558 -1.4964122 -0.6465469  C -7.1320758 -1.3810292 1.8518091  C -5.3965028 -4.4982962 -0.7736249  C -7.5654668 -3.0303742 -0.7828259  H -3.4620438 -5.4392822 -0.6686999  C -0.9569988 -3.8391962 -0.5908319  C -1.5194578 -3.3494542 1.7191801  C -6.9397188 2.3239918 1.5711611  C -7.8611258 -0.0582642 2.1488151  H -5.9487668 4.1257358 0.9326691  C -3.2019608 4.0497218 -0.1725989  C -4.2183398 3.2344368 -2.2230819  H -7.2912898 -1.0351202 -1.5910839  H -8.5850888 -1.1013322 -0.3793159  H -6.3119558 -1.5163312 2.5653681  H -7.7788528 -2.2621602 1.9047991  H -5.8784778 -5.3519572 -1.2415359  H -7.8757488 -3.3755612 -1.7733649  H -8.2279168 -3.5104442 -0.0512189  C -1.2812028 -3.9201822 -1.9728899  C 0.3957772 -4.0479092 -0.1960479  C -0.1725438 -3.5442642 2.1021021  H -2.2567938 -3.0842182 2.4722401  H -7.5821098 2.8309048 2.2856471  H -7.9302908 0.1581378 3.2188891  H -8.8850678 -0.0700502 1.7538381  C -3.0256358 4.0278768 1.2386251  C -2.3658868 4.9052528 -0.9478319  C -3.3891568 4.0871988 -2.9861329  H -4.9376128 2.5933418 -2.7246789  C -0.3183568 -4.2106882 -2.9064099  H -2.2997528 -3.7152182 -2.2864589  C 1.3680812 -4.3486162 -1.1859239  C 0.7608102 -3.9063762 1.1680371  H 0.1142942 -3.3944512 3.1386051  C -2.0860548 4.8232458 1.8417431  H -3.6239408 3.3459228 1.8338361  C -1.4015588 5.7148178 -0.2908049  C -2.4983698 4.9191038 -2.3605279  H -3.4728478 4.0857718 -4.0685929  C 1.0201002 -4.4352392 -2.5104569  H -0.5815748 -4.2512022 -3.9587609  H 2.3993322 -4.4924482 -0.8705329  H 1.8025302 -4.0466982 1.4466101  C -1.2654168 5.6801508 1.0713591  H -1.9484688 4.7754268 2.9173751  H -0.7650538 6.3548528 -0.8966369  H -1.8633208 5.5854478 -2.9386619  H 1.7741492 -4.6579912 -3.2599929  H -0.5153998 6.2919888 1.5624331  H -0.9977508 2.1966728 0.7372211 |
| **TS2’-C-*SR***  C -2.9418128 1.4820849 -0.5941724  O -2.0122108 1.6436909 -1.5996204  C -0.8171758 1.4778779 -1.0113014  C -1.0445988 1.1425949 0.3506696  N -2.4212848 1.2436999 0.5567906  C -4.3518898 1.6101969 -0.9473664  C -5.3030018 1.3911779 0.0501346  C -4.7282448 1.9080819 -2.2550874  C -6.6524768 1.4773389 -0.2780474  H -4.9993318 1.1551709 1.0648036  C -6.0848898 1.9955429 -2.5628404  H -3.9921518 2.0610369 -3.0353094  C -7.0597118 1.7809569 -1.5831754  H -8.1102918 1.8453459 -1.8297604  C -0.0607288 1.3040529 1.4834606  C -0.7709698 1.5150269 2.8180386  H 0.5921772 2.1565989 1.2652316  H 0.6032072 0.4398729 1.5502606  H -0.0386418 1.5456059 3.6292586  H -1.3284218 2.4563499 2.8154846  H -1.4902998 0.7129339 3.0108276  O 0.1985902 1.5703919 -1.7655364  C -0.4538418 -1.9365631 2.3315736  O 0.8485752 -1.9271701 2.2574606  C -0.9343248 -2.4607021 3.6540266  H -0.0754858 -2.7479401 4.2599796  H -1.5162938 -1.6926361 4.1736136  H -1.5977188 -3.3180451 3.5063856  C -1.3682578 -1.5669131 1.3389026  C -0.9433418 -1.1221111 0.0467696  C -1.6702458 -1.2592601 -1.2080114  C -0.8947658 -1.2365551 -2.3863864  C -3.0680548 -1.3393841 -1.3213684  C -1.4969048 -1.2925891 -3.6315864  H 0.1887262 -1.1790191 -2.3070294  C -3.6667598 -1.3896971 -2.5770304  H -3.6848578 -1.3474711 -0.4319784  C -2.8884918 -1.3627561 -3.7297884  H -0.8811298 -1.2767011 -4.5253964  H -4.7494698 -1.4310161 -2.6490034  H -3.3630428 -1.3933711 -4.7062574  H 0.1350462 -1.0960711 -0.0972444  C -2.7437768 -1.6808411 1.7142006  C -3.8764498 -1.8122141 2.1296966  C -5.2152098 -1.9785081 2.6079426  C -6.3059898 -1.5286611 1.8506616  C -5.4495788 -2.5969071 3.8458046  C -7.6008018 -1.6934171 2.3264006  H -6.1302548 -1.0419541 0.8965636  C -6.7473368 -2.7596751 4.3125256  H -4.6038288 -2.9430611 4.4322366  C -7.8274088 -2.3096411 3.5546466  H -8.4351748 -1.3345901 1.7312156  H -6.9169958 -3.2382291 5.2721776  H -8.8403848 -2.4365901 3.9239176  O -6.3765698 2.2903549 -3.8563834  O -7.5235328 1.2363619 0.7386646  C -7.7337788 2.4023669 -4.2190484  H -8.2345088 3.1988669 -3.6550024  H -7.7432088 2.6513189 -5.2800794  H -8.2688398 1.4572209 -4.0634634  C -8.9020568 1.3726429 0.4796586  H -9.4057008 1.1803469 1.4274896  H -9.1459808 2.3858589 0.1375536  H -9.2449178 0.6463879 -0.2686374  H 1.2864662 -1.5494111 1.4286286  O 2.1530372 -0.9758071 0.2850656  P 3.0275792 0.1304949 -0.2125614  O 4.1315802 -0.4659471 -1.2518954  O 3.9359852 0.6378749 1.0411856  O 2.4265742 1.3320719 -0.8973974  C 4.7983122 -1.5879431 -0.7770164  C 4.8730292 1.6148599 0.7260806  C 5.9497962 -1.4124191 -0.0239824  C 4.2616692 -2.8645441 -0.9909464  C 6.1335992 1.2208009 0.2947246  C 4.5223392 2.9698589 0.7816326  C 6.7624432 -0.1594301 0.2381646  C 6.5417572 -2.5141251 0.5979986  C 4.9035102 -3.9558721 -0.3940484  C 3.0761272 -3.0755371 -1.8681144  C 7.0401462 2.1837499 -0.1552844  C 5.4662082 3.9136729 0.3593836  C 3.2137032 3.4371329 1.3215336  C 7.4521862 -0.5476581 1.5763086  C 7.8337182 0.0630339 -0.8677464  C 6.0256332 -3.7902651 0.4147426  C 7.7017482 -2.0637051 1.4571716  H 4.4904472 -4.9477491 -0.5567904  C 1.8800922 -3.6944481 -1.3792784  C 3.1588742 -2.7193551 -3.1956564  C 6.7146732 3.5331019 -0.1241744  C 8.2937312 1.5192959 -0.6761984  H 5.1932442 4.9648229 0.3978686  C 2.2892472 4.1635269 0.5046366  C 2.9335572 3.2445729 2.6563826  H 6.7527032 -0.3535311 2.3968576  H 8.3620242 0.0329539 1.7589576  H 7.3502302 -0.0430711 -1.8449874  H 8.6466702 -0.6674551 -0.8081684  H 6.4823432 -4.6486351 0.8992866  H 7.7241432 -2.5670441 2.4283526  H 8.6597552 -2.2720091 0.9635586  C 1.6744232 -3.9816661 -0.0020844  C 0.8305482 -3.9863991 -2.2964864  C 2.1128822 -3.0081711 -4.1024024  H 4.0592442 -2.2282651 -3.5533344  H 7.4165342 4.2818219 -0.4805404  H 8.6571022 1.9749389 -1.6021064  H 9.1062742 1.5878439 0.0586686  C 2.4691852 4.3196029 -0.8971114  C 1.1200942 4.7131289 1.1070456  C 1.7781622 3.8005269 3.2516216  H 3.6336382 2.6797379 3.2653656  C 0.4929312 -4.5273301 0.4327736  H 2.4534272 -3.7249971 0.7089326  C -0.3783958 -4.5552681 -1.8145754  C 0.9843182 -3.6508741 -3.6670854  H 2.2208312 -2.7310561 -5.1467154  C 1.5406102 4.9863779 -1.6538474  H 3.3343482 3.8627899 -1.3652524  C 0.1818032 5.4057249 0.2955126  C 0.8992802 4.5334459 2.4976596  H 1.5976382 3.6487849 4.3116456  C -0.5480848 -4.8196531 -0.4809274  H 0.3488642 -4.7184631 1.4929566  H -1.1791688 -4.7460251 -2.5250654  H 0.1758502 -3.8878921 -4.3544244  C 0.3849422 5.5406439 -1.0525674  H 1.6827782 5.0774439 -2.7261634  H -0.7069958 5.8178899 0.7667166  H 0.0099212 4.9686549 2.9467046  H -1.4843118 -5.2335451 -0.1189244  H -0.3431888 6.0634519 -1.6652094  H 1.1927192 1.4438339 -1.3041754 | **TS2’-C-*RS***  C -4.1819816 -0.0951182 0.1694846  O -3.0349106 -0.5708582 -0.3923784  C -2.0108156 0.1293688 0.2198886  C -2.6432686 1.1294678 1.0044356  N -4.0114596 0.8719378 1.0091126  C -5.4353186 -0.7354612 -0.2208644  C -6.6410976 -0.1634112 0.1834836  C -5.4065096 -1.9140672 -0.9665344  C -7.8337026 -0.7911102 -0.1638304  H -6.6531866 0.7521398 0.7642716  C -6.6091436 -2.5367182 -1.2912684  H -4.4695506 -2.3526942 -1.2915484  C -7.8322026 -1.9837902 -0.8988874  H -8.7619076 -2.4708972 -1.1574254  C -1.9958436 1.9578458 2.0657466  C -2.0636116 1.3087948 3.4512156  H -0.9525806 2.1382348 1.7840536  H -2.4971276 2.9327678 2.0981336  H -1.6834826 1.9919978 4.2163906  H -1.4598686 0.3969598 3.4812476  H -3.0969366 1.0496088 3.7018896  O -0.8398366 -0.1100072 -0.0926194  C -0.3116466 1.6590848 -1.8133664  O 0.9964224 1.7556258 -1.8184464  C -0.9083436 0.6662198 -2.7640974  H -0.3291306 -0.2589032 -2.7167974  H -0.8328746 1.0804808 -3.7771754  H -1.9489406 0.4273628 -2.5548144  C -1.0053896 2.6152968 -1.0533224  C -2.4029326 2.5059998 -0.8645454  C -3.3399146 3.5438418 -0.4158404  C -4.7048556 3.3238038 -0.6724594  C -2.9691716 4.7294878 0.2380976  C -5.6663306 4.2473788 -0.2902894  H -5.0085266 2.4102518 -1.1771404  C -3.9357776 5.6552468 0.6162866  H -1.9255346 4.9271028 0.4481166  C -5.2836036 5.4201318 0.3576106  H -6.7135936 4.0521468 -0.4991454  H -3.6306346 6.5675058 1.1197936  H -6.0319076 6.1472628 0.6583906  H -2.8948796 1.7661908 -1.4908064  C -0.1790086 3.5266458 -0.3173744  C 0.6016564 4.2125378 0.3063086  C 1.5161614 4.9574818 1.1199286  C 1.6835974 6.3379108 0.9485336  C 2.2670964 4.2888518 2.0977126  C 2.5850224 7.0365988 1.7443076  H 1.1063464 6.8504898 0.1852486  C 3.1651134 4.9935768 2.8898996  H 2.1492014 3.2150968 2.2074316  C 3.3264434 6.3676228 2.7169036  H 2.7098464 8.1062238 1.6048806  H 3.7439684 4.4678168 3.6432986  H 4.0283564 6.9165168 3.3374136  O -6.4988786 -3.6941382 -1.9953614  O -8.9691816 -0.1747182 0.2557086  C -7.6865826 -4.3604212 -2.3598434  H -8.2610396 -4.6655012 -1.4766034  H -7.3782116 -5.2470352 -2.9136214  H -8.3159066 -3.7319232 -3.0017214  C -10.2054376 -0.7674152 -0.0707614  H -10.9719106 -0.1149522 0.3473086  H -10.2997246 -1.7670552 0.3710856  H -10.3416296 -0.8375742 -1.1571124  H 1.4142244 0.9150278 -2.1419194  O 1.5286484 -0.2561832 0.7733146  P 2.2550364 -0.9649292 -0.4117524  O 2.2669844 -2.5568082 -0.1353024  O 3.7671504 -0.4946162 -0.1001164  O 1.8036954 -0.7373292 -1.8043064  C 2.6029554 -2.9765622 1.1484966  C 4.7287384 -1.0226392 -0.9575724  C 3.9421124 -3.1145922 1.4876636  C 1.5840164 -3.2079392 2.0839986  C 5.3162564 -2.2335372 -0.6275144  C 5.0373324 -0.3603792 -2.1540574  C 5.1978054 -3.0695692 0.6322696  C 4.2857094 -3.3887812 2.8153556  C 1.9658734 -3.5377262 3.3903486  C 0.1323744 -3.0813922 1.7681406  C 6.1916864 -2.8416352 -1.5318414  C 5.9575874 -0.9727472 -3.0133354  C 4.3767394 0.9074098 -2.5729164  C 6.2492764 -2.6545192 1.6991976  C 5.5490454 -4.4754962 0.0656096  C 3.3032764 -3.6056642 3.7704146  C 5.7872224 -3.3588732 2.9876236  H 1.1833444 -3.7246542 4.1197406  C -0.4981226 -3.8593412 0.7411486  C -0.6416526 -2.2643982 2.5654756  C 6.5247304 -2.2108982 -2.7221914  C 6.6068284 -4.2018442 -1.0192824  H 6.2075044 -0.4637072 -3.9395864  C 4.4406854 2.1017438 -1.7863234  C 3.7207804 0.9276658 -3.7861664  H 6.1974414 -1.5682282 1.8336816  H 7.2693414 -2.9124122 1.3982096  H 4.6495874 -4.8984262 -0.3957784  H 5.8875004 -5.1646152 0.8454696  H 3.5688814 -3.8213182 4.8015006  H 6.0910744 -2.8269152 3.8940776  H 6.1968764 -4.3746352 3.0571316  C 0.2141034 -4.7617902 -0.0936264  C -1.9116156 -3.7651492 0.5783046  C -2.0448376 -2.1847762 2.4071246  H -0.1532686 -1.6737082 3.3368946  H 7.2059954 -2.6789312 -3.4271794  H 6.6149004 -4.9616372 -1.8063704  H 7.6168384 -4.1686982 -0.5908464  C 5.1335994 2.1743978 -0.5474664  C 3.8058874 3.2799208 -2.2768234  C 3.1035654 2.1015948 -4.2718554  H 3.6588094 0.0083808 -4.3616724  C -0.4334696 -5.4993862 -1.0515964  H 1.2856854 -4.8707402 0.0345326  C -2.5531946 -4.5407442 -0.4234524  C -2.6659476 -2.9240672 1.4363116  H -2.6269206 -1.5373772 3.0565376  C 5.1711804 3.3419598 0.1702306  H 5.6402784 1.2908928 -0.1730394  C 3.8531824 4.4688198 -1.5021714  C 3.1433684 3.2526688 -3.5296064  H 2.5908464 2.0792768 -5.2288144  C -1.8323246 -5.3848742 -1.2266444  H 0.1326664 -6.1798792 -1.6802754  H -3.6325496 -4.4590872 -0.5329054  H -3.7447506 -2.8746842 1.3022266  C 4.5192214 4.5039208 -0.3068564  H 5.6996124 3.3778028 1.1185186  H 3.3410084 5.3526668 -1.8748884  H 2.6613944 4.1606578 -3.8827074  H -2.3314586 -5.9726212 -1.9909054  H 4.5310114 5.4137168 0.2872816  H 0.5452474 -0.1102112 0.5852156 |
| **TS2’-C-*RR***  C 3.0305738 1.6653491 0.9255230  O 1.9787768 2.3395971 0.3163950  C 0.8753668 1.8942831 0.9292460  C 1.2660878 0.8920641 1.8577850  N 2.6600868 0.8756731 1.8700660  C 4.3766788 1.9147241 0.4251610  C 5.4118378 1.1300981 0.9310350  C 4.6100048 2.8740091 -0.5613600  C 6.6997988 1.3040201 0.4345180  H 5.2197178 0.3739931 1.6832780  C 5.9079308 3.0399001 -1.0428050  H 3.8111388 3.4874751 -0.9623570  C 6.9619258 2.2605221 -0.5545700  H 7.9643268 2.3920701 -0.9373910  C 0.4718108 0.5224391 3.0750140  C 0.4426628 1.6787711 4.0794780  H 0.9338548 -0.3630529 3.5224790  H -0.5473562 0.2519191 2.7823400  H -0.1098432 1.3943111 4.9794720  H 1.4574658 1.9651111 4.3715780  H -0.0493772 2.5536571 3.6408000  O -0.2494732 2.3658311 0.5726180  C 1.7510938 0.2759981 -1.7654250  O 0.6533798 0.8790641 -2.1042150  C 2.8676778 0.5242761 -2.7349040  H 2.5139618 1.1835881 -3.5269390  H 3.7241838 0.9750631 -2.2212590  H 3.2113328 -0.4236219 -3.1619780  C 1.9421948 -0.5326329 -0.6330080  C 0.9128888 -0.6951949 0.3434690  C 0.8187758 -1.8049919 1.3011770  C -0.4672582 -2.2312799 1.6646940  C 1.9318018 -2.4150609 1.9000590  C -0.6404432 -3.2701629 2.5716200  H -1.3380922 -1.7510739 1.2271080  C 1.7524858 -3.4548089 2.8038100  H 2.9272038 -2.0480709 1.6823200  C 0.4693978 -3.8909599 3.1356060  H -1.6437302 -3.6045719 2.8165210  H 2.6196428 -3.9207279 3.2625500  H 0.3370128 -4.7089779 3.8377620  H -0.0621182 -0.3309689 0.0379570  C 3.2381948 -1.1234069 -0.5301650  C 4.3860778 -1.5173669 -0.5379680  C 5.7644188 -1.8986539 -0.5333220  C 6.3009508 -2.6649119 0.5096920  C 6.6066528 -1.4650969 -1.5691810  C 7.6515468 -2.9931299 0.5127030  H 5.6506668 -2.9942019 1.3143820  C 7.9526798 -1.8058249 -1.5639670  H 6.1901638 -0.8588699 -2.3682270  C 8.4804528 -2.5696289 -0.5233690  H 8.0580198 -3.5856219 1.3267290  H 8.5940678 -1.4757029 -2.3764190  H 9.5334538 -2.8354479 -0.5222920  O 6.0599108 3.9895161 -2.0026620  O 7.6479528 0.4896291 0.9601290  C 7.3548978 4.2144931 -2.5114340  H 7.7582568 3.3141331 -2.9915730  H 7.2537598 5.0048601 -3.2549230  H 8.0430928 4.5420401 -1.7224690  C 8.9838498 0.6832611 0.5561620  H 9.5690628 -0.0676609 1.0873560  H 9.1009578 0.5269581 -0.5226580  H 9.3371458 1.6874851 0.8234370  H -1.0975142 1.7277441 0.8934030  H -0.2322802 0.6455771 -1.6566690  O -1.7190362 0.2961211 -1.4588210  P -2.6271662 0.3867771 -0.2756860  O -3.3538952 -1.0389579 0.0405470  O -3.8565922 1.3585791 -0.6934080  O -2.0417962 0.8102651 1.0611930  C -3.9893402 -1.6277519 -1.0482520  C -4.8211812 1.5788661 0.2799180  C -5.2944002 -1.2597619 -1.3505990  C -3.2957332 -2.5448399 -1.8471100  C -5.8917222 0.7018991 0.3585700  C -4.6793262 2.6377971 1.1882850  C -6.2955032 -0.4298879 -0.5660820  C -5.8856992 -1.7300579 -2.5273060  C -3.9352162 -3.0397409 -2.9881250  C -1.9296102 -3.0557139 -1.5179770  C -6.8108642 0.8253091 1.4033150  C -5.6460022 2.7638171 2.1938330  C -3.5214202 3.5733941 1.1626400  C -7.2108272 0.0690241 -1.7189700  C -7.1177362 -1.3123799 0.4168760  C -5.2127122 -2.6201859 -3.3515600  C -7.2493112 -1.1040899 -2.7150130  H -3.3966102 -3.7514099 -3.6071270  C -1.7896552 -4.1693099 -0.6282840  C -0.8210852 -2.5586189 -2.1647310  C -6.6980812 1.8602181 2.3209670  C -7.8116732 -0.3063929 1.3539630  H -5.5472932 3.5844061 2.8985250  C -3.1916322 4.3515441 0.0059870  C -2.7788512 3.7266691 2.3145660  H -6.7342392 0.9363231 -2.1894790  H -8.1986082 0.3752791 -1.3607910  H -6.4204292 -1.9302819 0.9936920  H -7.8120412 -1.9782459 -0.1052590  H -5.6685712 -2.9830399 -4.2684730  H -7.4211162 -0.7802319 -3.7458360  H -8.0460952 -1.8156749 -2.4633130  C -2.8899542 -4.7174929 0.0844940  C -0.5028062 -4.7544209 -0.4485700  C 0.4570158 -3.1373279 -1.9714070  H -0.9380352 -1.7082179 -2.8300010  H -7.4115992 1.9621841 3.1337290  H -8.0214112 -0.7235009 2.3434280  H -8.7682802 0.0334911 0.9366710  C -3.9469862 4.3039401 -1.1971800  C -2.0801862 5.2415731 0.0711650  C -1.6941822 4.6289141 2.3801890  H -3.0253692 3.1193521 3.1814190  C -2.7209062 -5.7903209 0.9240320  H -3.8732982 -4.2758329 -0.0466190  C -0.3630532 -5.8670179 0.4209080  C 0.6136398 -4.2099609 -1.1345250  H 1.3167588 -2.7216139 -2.4891180  C -3.5998732 5.0700971 -2.2792360  H -4.8133162 3.6534111 -1.2513060  C -1.7410842 6.0147281 -1.0709180  C -1.3445252 5.3615181 1.2771740  H -1.1374932 4.7290981 3.3073590  C -1.4439672 -6.3765869 1.0926710  H -3.5734432 -6.1972279 1.4599810  H 0.6259198 -6.2987539 0.5532950  H 1.5936718 -4.6533559 -0.9775580  C -2.4779352 5.9304721 -2.2218850  H -4.1898172 5.0181311 -3.1891170  H -0.8833932 6.6797231 -1.0092890  H -0.5025242 6.0481521 1.3086330  H -1.3248242 -7.2264049 1.7582290  H -2.2094932 6.5257171 -3.0892460 | **TS2’-C-*SS***  C 4.1968734 -0.1344196 0.1606641  O 3.3240404 0.3331194 -0.7830419  C 2.0868584 0.1510374 -0.2655759  C 2.2607934 -0.3809466 1.0399241  N 3.6190534 -0.5803456 1.2275391  C 5.6184604 -0.1262986 -0.1646549  C 6.5222224 -0.6128596 0.7809631  C 6.0554984 0.3805944 -1.3880489  C 7.8825114 -0.5853596 0.4918991  H 6.1752634 -1.0063596 1.7294851  C 7.4224274 0.4027674 -1.6589299  H 5.3597374 0.7730174 -2.1203399  C 8.3488744 -0.0769646 -0.7275659  H 9.4073434 -0.0578536 -0.9459369  C 1.2421694 -1.0387406 1.9101831  C 1.8083664 -1.4075526 3.2798051  H 0.3545364 -0.4031096 2.0027841  H 0.8763724 -1.9510736 1.4225941  H 1.0203394 -1.8290686 3.9106351  H 2.2389444 -0.5389106 3.7904311  H 2.6055254 -2.1466166 3.1753871  O 1.1214894 0.5453394 -0.9799419  C 0.0528604 1.7320734 3.2336271  O -1.2398976 1.5728484 3.3298451  C 0.7252384 1.6381254 4.5661811  H 1.7850454 1.8829564 4.5453341  H 0.5761994 0.6376404 4.9821701  H 0.2165294 2.3432854 5.2326651  C 0.6623044 2.0662774 2.0166981  C 2.0650774 1.9316194 1.8764911  C 2.9709104 2.6076594 0.9687361  C 4.3438614 2.5221244 1.2685891  C 2.5754584 3.2980244 -0.1967459  C 5.2963824 3.1078464 0.4424511  H 4.6580714 1.9809114 2.1574821  C 3.5316504 3.8737194 -1.0162749  H 1.5282734 3.3532744 -0.4637289  C 4.8916604 3.7777584 -0.7060139  H 6.3510264 3.0091544 0.6811501  H 3.2178254 4.3943484 -1.9154999  H 5.6314324 4.2197274 -1.3667389  H 2.5707244 1.5102844 2.7409691  C -0.1961206 2.5039644 0.9559881  C -0.9202996 2.9173084 0.0761291  C -1.6367086 3.4840584 -1.0301679  C -1.5205746 4.8595504 -1.2788229  C -2.4065456 2.6850394 -1.8862809  C -2.1519486 5.4251664 -2.3801319  H -0.9270466 5.4701754 -0.6047579  C -3.0366746 3.2624904 -2.9823219  H -2.4904536 1.6215864 -1.6925199  C -2.9062366 4.6258754 -3.2362459  H -2.0525826 6.4896304 -2.5714379  H -3.6411216 2.6416464 -3.6361479  H -3.4036716 5.0690514 -4.0941859  O 7.7735714 0.9205614 -2.8652319  O 8.7013924 -1.0748306 1.4593541  C 9.1419744 0.9430984 -3.2010939  H 9.7168384 1.5619964 -2.5007649  H 9.2016974 1.3783524 -4.1985689  H 9.5654854 -0.0687136 -3.2202439  C 10.0895324 -1.0767226 1.2154481  H 10.5509644 -1.5067136 2.1043791  H 10.4716514 -0.0593316 1.0648911  H 10.3424084 -1.6906726 0.3423361  H 0.0479474 0.1854324 -0.8229969  H -1.6251456 1.2192334 2.4690011  O -1.9214596 -0.1246956 1.5564011  P -2.0795736 -0.6214976 0.1614281  O -3.5791496 -0.3139176 -0.4226789  O -2.0412746 -2.2565056 0.2320521  O -1.1544816 -0.1671416 -0.9500179  C -4.6049216 -0.6224896 0.4641981  C -2.3091416 -2.9563556 -0.9361419  C -5.0848016 -1.9222986 0.5307911  C -5.1047906 0.3642224 1.3232271  C -3.6282116 -3.2729706 -1.2379569  C -1.2739166 -3.3304186 -1.8064299  C -4.8847826 -3.0889806 -0.4120689  C -5.9651706 -2.2852026 1.5546581  C -6.0270916 -0.0130686 2.3034881  C -4.6911626 1.7898854 1.2131861  C -3.9387676 -3.8934256 -2.4512089  C -1.6157326 -3.9818396 -2.9985719  C 0.1703794 -3.0759956 -1.5510249  C -5.1424496 -4.2808056 0.5496601  C -5.9633936 -3.0706986 -1.5340289  C -6.4364266 -1.3369396 2.4505401  C -6.2416846 -3.7717896 1.5026611  H -6.4064866 0.7541034 2.9722411  C -5.1486136 2.5977964 0.1221081  C -3.9567106 2.3631174 2.2226001  C -2.9366526 -4.2513436 -3.3399989  C -5.4342496 -4.0472266 -2.5974829  H -0.8107486 -4.2709476 -3.6678279  C 0.8506524 -3.6559806 -0.4332569  C 0.8889184 -2.3575896 -2.4820129  H -4.2238116 -4.4762326 1.1141681  H -5.4154746 -5.1954766 0.0138271  H -6.0075466 -2.0593406 -1.9540969  H -6.9583306 -3.3255676 -1.1560129  H -7.1154606 -1.6162456 3.2512541  H -6.1944526 -4.2389446 2.4909861  H -7.2427016 -3.9714916 1.0990171  C -5.9461566 2.0794494 -0.9311889  C -4.8322656 3.9864034 0.1174681  C -3.5957116 3.7314654 2.1849961  H -3.6494386 1.7457924 3.0614811  H -3.1735726 -4.7311356 -4.2853869  H -5.7844166 -3.8098706 -3.6065059  H -5.7436946 -5.0781606 -2.3815729  C 0.1786514 -4.4000516 0.5751741  C 2.2601884 -3.4730536 -0.3154829  C 2.2886554 -2.2015216 -2.3744439  H 0.3583744 -1.8928686 -3.3078229  C -6.4215096 2.9005034 -1.9236959  H -6.1917886 1.0218504 -0.9343739  C -5.3377176 4.8074374 -0.9221479  C -4.0293216 4.5257254 1.1579711  H -2.9772506 4.1387194 2.9790381  C 0.8607084 -4.8921466 1.6588241  H -0.8903236 -4.5632376 0.4871591  C 2.9364194 -3.9912866 0.8204761  C 2.9624844 -2.7549036 -1.3177349  H 2.8214264 -1.6201886 -3.1207129  C -6.1217336 4.2817624 -1.9168399  H -7.0384496 2.4883384 -2.7168669  H -5.0844736 5.8647634 -0.9165889  H -3.7673826 5.5801964 1.1180521  C 2.2540904 -4.6803946 1.7892731  H 0.3283314 -5.4461986 2.4258451  H 4.0054114 -3.8138646 0.9107341  H 4.0389104 -2.6323526 -1.2164319  H -6.5038896 4.9216224 -2.7068659  H 2.7784474 -5.0647776 2.6592411 |
| **INT0’-B**  C -5.0047598 0.2736120 0.0713779  O -4.4068948 0.0576820 -1.1689611  C -3.0917998 0.3681260 -1.0090691  C -2.9066658 0.7739390 0.4395209  C -6.4466548 -0.0004750 0.1394599  C -7.0914078 0.1815530 1.3612439  C -7.1339668 -0.4358050 -0.9897441  C -8.4548358 -0.0809370 1.4473709  H -6.5439538 0.5163170 2.2345159  C -8.5006078 -0.6943060 -0.8858721  H -6.6372358 -0.5814090 -1.9410541  C -9.1717518 -0.5205270 0.3274149  H -10.2311708 -0.7228910 0.4006869  O -2.3201218 0.3230900 -1.9339411  N -4.2465578 0.6763360 1.0031309  C -2.2952898 2.1766160 0.5549019  C -3.1729258 3.2446230 -0.0879881  H -1.3025328 2.1556660 0.0864339  H -2.1424388 2.3902810 1.6168829  H -2.7635698 4.2362850 0.1133949  H -3.2249628 3.1178830 -1.1761061  H -4.1891488 3.2074270 0.3167839  O -9.1061938 -1.1164040 -2.0251181  O -9.0191038 0.1143770 2.6665249  C -10.4819718 -1.4189850 -1.9697301  H -10.7588518 -1.7480870 -2.9710481  H -10.6849928 -2.2241120 -1.2528421  H -11.0751128 -0.5362750 -1.7008771  C -10.3946528 -0.1569720 2.8114529  H -10.6201208 -1.2080890 2.5933039  H -10.6350818 0.0549230 3.8531079  H -11.0008898 0.4862210 2.1617549  H -0.6121858 0.4433450 -1.8561551  H -2.2355718 0.0499710 0.9160789  C 3.9439792 0.4501500 -0.1441641  C 2.8533352 1.3188110 -0.1996811  C 2.9056592 2.5530420 -0.8483731  C 4.1098352 2.8832480 -1.4663501  C 5.2035332 2.0169880 -1.4939581  C 5.1185582 0.7741250 -0.8506681  H 4.1838142 3.8456750 -1.9684931  C 3.7830072 -0.8349150 0.5981199  C 4.5742942 -1.1761900 1.7129819  C 2.7520732 -1.6891140 0.2110359  C 4.3696202 -2.4140890 2.3388789  C 2.5177852 -2.9197020 0.8251109  C 3.3627512 -3.2644560 1.8760969  H 3.2077282 -4.2222310 2.3683659  C 6.4626642 2.4596430 -2.2146371  C 7.3647572 1.2956060 -2.6160171  C 7.5583342 0.3594720 -1.4262321  C 6.2216642 -0.2559710 -1.0193531  H 6.1872992 3.0554950 -3.0916461  H 6.9039402 0.7377420 -3.4415541  H 8.3249652 1.6755560 -2.9794251  H 8.2724122 -0.4356280 -1.6641901  H 7.9765932 0.9260390 -0.5823341  H 5.8963732 -0.9561850 -1.8029021  H 6.3372692 -0.8625370 -0.1180311  H 7.0254112 3.1321480 -1.5517101  C 5.5423752 -0.1650440 2.3021619  C 6.5908832 -0.7995660 3.2122389  C 5.9168972 -1.7159950 4.2294609  C 5.2245172 -2.8688640 3.5064789  H 6.0246782 0.4234870 1.5178949  H 7.1670102 -0.0144850 3.7126209  H 6.6427522 -2.1085040 4.9489129  H 5.1768062 -1.1381870 4.7985099  H 5.9896922 -3.5615100 3.1282689  H 4.6073682 -3.4472300 4.2027019  H 4.9499752 0.5549600 2.8858379  O 1.6624362 0.9275030 0.3996079  O 1.8986522 -1.2699850 -0.8068771  P 0.7768732 -0.2002880 -0.3516661  O -0.3026978 -0.6882560 0.5175609  O 0.3723382 0.4217540 -1.7386121  C 1.7313212 3.4589880 -0.9652381  C 1.0761302 4.0272100 0.1750539  C 1.2979182 3.8036470 -2.2238071  C 1.4718612 3.7184050 1.5038489  C 0.0080522 4.9528450 -0.0234681  C 0.2386642 4.7171850 -2.4126551  H 1.7780942 3.3544020 -3.0883521  C 0.8291202 4.2693250 2.5825789  H 2.2947052 3.0291790 1.6604809  C -0.6264638 5.5146550 1.1175729  C -0.4008988 5.3048960 -1.3496221  H -0.0669868 4.9674240 -3.4251741  C -0.2365448 5.1759440 2.3883809  H 1.1416232 4.0112170 3.5895779  H -1.4361978 6.2242710 0.9758899  H -0.7393038 5.6119540 3.2462929  C 1.3753892 -3.7883240 0.4361239  C 1.2813732 -4.3784530 -0.8647561  C 0.3913902 -4.0388980 1.3604569  C 2.2824162 -4.1840820 -1.8523011  C 0.1614272 -5.2038610 -1.1756791  C -0.7164058 -4.8561590 1.0452589  H 0.4532592 -3.5725180 2.3394079  C 2.1792392 -4.7644050 -3.0905461  H 3.1435232 -3.5681050 -1.6124671  C 0.0845122 -5.7859410 -2.4690151  C -0.8523668 -5.4341920 -0.1916991  H -1.4824578 -5.0184670 1.7987949  C 1.0646092 -5.5725780 -3.4046681  H 2.9548652 -4.6027040 -3.8327291  H -0.7666878 -6.4111680 -2.7187551  H 0.9853992 -6.0263070 -4.3879471  C -1.4904448 6.3195570 -1.5806991  H -2.4472138 6.0027450 -1.1510811  H -1.2354178 7.2853530 -1.1302841  H -1.6429708 6.4772450 -2.6509571  C -2.0486218 -6.2922190 -0.5100641  H -2.6159548 -5.8886530 -1.3560121  H -1.7532388 -7.3140900 -0.7732901  H -2.7200038 -6.3463970 0.3500599  H 7.2990502 -1.3844360 2.6086849 | **TS1’-B**  C -4.2133910 1.2411540 -0.0160058  O -3.6894900 1.1499960 -1.3065058  C -2.4448570 1.6232540 -1.2138118  C -2.1774020 1.9504460 0.1543692  C -5.5790030 0.7438540 0.1655942  C -6.0181400 0.4863590 1.4625172  C -6.4024650 0.5286710 -0.9365458  C -7.3087050 -0.0012890 1.6520262  H -5.3700760 0.6575260 2.3142292  C -7.6907080 0.0382950 -0.7294268  H -6.0663020 0.7255650 -1.9478818  C -8.1549360 -0.2341960 0.5611212  H -9.1529450 -0.6200800 0.7142602  O -1.7113140 1.5897990 -2.2447508  N -3.4071240 1.7224800 0.8435912  C -1.3073890 3.1280560 0.5693702  C -2.0391930 4.4627810 0.4525082  H -0.4030520 3.1328070 -0.0502568  H -0.9777080 2.9612920 1.6003452  H -1.4080740 5.2768520 0.8224532  H -2.2993960 4.6763870 -0.5903258  H -2.9620970 4.4487140 1.0397252  O -8.4347950 -0.1484490 -1.8492688  O -7.6682020 -0.2324490 2.9400332  C -9.7483640 -0.6378190 -1.6972638  H -10.1627320 -0.7073790 -2.7027188  H -9.7530230 -1.6320000 -1.2332858  H -10.3622990 0.0448680 -1.0970668  C -8.9656580 -0.7244600 3.1891322  H -9.1192490 -1.7040430 2.7199562  H -9.0464220 -0.8268330 4.2711222  H -9.7329250 -0.0265330 2.8323072  H -0.6331010 1.5502430 -1.9605908  H -1.3430440 0.9434360 0.4246592  C 3.8852470 -0.5942990 -0.0879498  C 3.3257150 0.6829820 -0.0148568  C 3.9541650 1.8231020 -0.5174528  C 5.2128860 1.6386840 -1.0855498  C 5.7984420 0.3777040 -1.2141678  C 5.1177950 -0.7583980 -0.7492678  H 5.7376720 2.5093690 -1.4736868  C 3.0928390 -1.7425270 0.4510122  C 3.5543400 -2.5786970 1.4871872  C 1.7992090 -1.9305450 -0.0366408  C 2.7416060 -3.6361460 1.9225842  C 0.9461610 -2.9356830 0.4223122  C 1.4619250 -3.7979330 1.3847062  H 0.8270540 -4.6003070 1.7548642  C 7.1687090 0.2771400 -1.8581548  C 7.4723780 -1.1099320 -2.4185748  C 7.1320280 -2.1734200 -1.3784438  C 5.6358830 -2.1481670 -1.0760828  H 7.2583050 1.0383000 -2.6409888  H 6.8725120 -1.2856890 -3.3211128  H 8.5248350 -1.1697470 -2.7143698  H 7.4204720 -3.1700170 -1.7280368  H 7.6996130 -1.9777600 -0.4578768  H 5.0871770 -2.4990880 -1.9625428  H 5.3917230 -2.8592790 -0.2835048  H 7.9263680 0.5265380 -1.1015648  C 4.8476220 -2.2570200 2.2154092  C 5.4217870 -3.4524230 2.9715302  C 4.3428550 -4.0827650 3.8478842  C 3.2142940 -4.6229710 2.9731282  H 5.5920890 -1.8388340 1.5340582  H 6.2776030 -3.1313630 3.5741762  H 4.7560140 -4.8888090 4.4630432  H 3.9487780 -3.3220710 4.5343542  H 3.5661170 -5.5302550 2.4616182  H 2.3624170 -4.9317550 3.5888122  H 4.6263350 -1.4540370 2.9338522  O 2.0637500 0.8193690 0.5480172  O 1.3106370 -1.0240300 -0.9738578  P 0.8026570 0.3769030 -0.3535308  O -0.3460470 0.1655010 0.6162902  O 0.5763320 1.3299780 -1.5041128  C 3.2453810 3.1297140 -0.5865168  C 2.6790810 3.7732700 0.5614622  C 3.0827660 3.7098540 -1.8223698  C 2.8543020 3.2627800 1.8747552  C 1.9268580 4.9727280 0.3896062  C 2.3495800 4.9048020 -1.9836618  H 3.4881270 3.2112230 -2.6981228  C 2.2883830 3.8805550 2.9602152  H 3.4472780 2.3643110 2.0117822  C 1.3561300 5.5889610 1.5363512  C 1.7618960 5.5358360 -0.9159518  H 2.2318790 5.3206490 -2.9808238  C 1.5221870 5.0547240 2.7890262  H 2.4302470 3.4674310 3.9539442  H 0.7853740 6.5047160 1.4156022  H 1.0748790 5.5397390 3.6513552  C -0.4813300 -2.9635340 0.0018712  C -0.8717200 -3.1462300 -1.3619978  C -1.4447200 -2.7055020 0.9465402  C 0.0728830 -3.4563130 -2.3748438  C -2.2456840 -3.0164780 -1.7180448  C -2.8048140 -2.5738420 0.5882472  H -1.1430030 -2.5277430 1.9746412  C -0.3163940 -3.6274970 -3.6783068  H 1.1185510 -3.5582300 -2.0998718  C -2.6132690 -3.1957690 -3.0783488  C -3.2191680 -2.7078340 -0.7139378  H -3.5342600 -2.3332930 1.3578582  C -1.6763740 -3.4936700 -4.0350138  H 0.4212070 -3.8629070 -4.4394598  H -3.6561290 -3.0960440 -3.3620098  H -1.9789490 -3.6265620 -5.0693098  C 0.9614670 6.7960970 -1.1148048  H -0.0766440 6.6684070 -0.7871248  H 1.3820610 7.6340080 -0.5475858  H 0.9475090 7.0776450 -2.1703138  C -4.6683840 -2.5269090 -1.0812608  H -4.7939360 -1.7275610 -1.8200438  H -5.0900460 -3.4417700 -1.5135688  H -5.2614790 -2.2593230 -0.2022758  H 5.7935640 -4.1993480 2.2561902 |
| **INT1’-B**  C -3.2068645 2.4837056 -0.2502454  O -2.9185265 2.5765916 -1.5633674  C -1.6846125 3.1615716 -1.6156224  C -1.2815425 3.4187096 -0.3448884  C -4.4769945 1.8734496 0.1426126  C -4.7338905 1.6705306 1.4978416  C -5.4057285 1.5101436 -0.8303784  C -5.9428785 1.0947976 1.8772786  H -4.0100425 1.9572826 2.2518306  C -6.6191765 0.9554886 -0.4320104  H -5.2109955 1.6635206 -1.8852984  C -6.8996085 0.7331426 0.9202036  H -7.8415945 0.2966896 1.2223996  O -1.1053575 3.2832846 -2.8038574  N -2.2713155 2.9594346 0.5222726  C -0.0012565 4.0274636 0.1249306  C -0.2441055 5.3412496 0.8717266  H 0.6471955 4.1822546 -0.7449174  H 0.5300325 3.3236086 0.7830126  H 0.6916615 5.7385666 1.2743136  H -0.6868675 6.0861046 0.2029976  H -0.9364925 5.1803306 1.7033186  O -7.4824885 0.6488186 -1.4364454  O -6.1187785 0.9159576 3.2128276  C -8.7311405 0.0934766 -1.0923914  H -9.2628235 -0.0560634 -2.0320734  H -8.6151395 -0.8730074 -0.5852444  H -9.3077145 0.7718306 -0.4516214  C -7.3156675 0.3113866 3.6460366  H -7.4284215 -0.6966284 3.2273526  H -7.2448835 0.2454016 4.7316356  H -8.1908575 0.9148406 3.3755066  H -0.3893295 2.6078276 -2.8117494  H -1.1162305 1.3505196 0.5061606  C 3.1349965 -1.2961044 -0.1786464  C 2.9507055 0.0855316 -0.2178764  C 3.8929995 0.9628096 -0.7517184  C 5.0507965 0.3942206 -1.2744974  C 5.2534245 -0.9874014 -1.3138214  C 4.2802145 -1.8481894 -0.7849024  H 5.8092515 1.0554206 -1.6883424  C 2.0643295 -2.1328214 0.4411906  C 2.2895625 -2.9415374 1.5727366  C 0.7727225 -2.0376354 -0.0730544  C 1.2299475 -3.7039744 2.0847306  C -0.3049755 -2.7669904 0.4278146  C -0.0351155 -3.6144084 1.4986856  H -0.8527955 -4.2040154 1.9080506  C 6.5424805 -1.5143634 -1.9158784  C 6.4546905 -2.9768454 -2.3447454  C 5.8320195 -3.8085424 -1.2268374  C 4.3982555 -3.3504354 -0.9731844  H 6.8326445 -0.8810744 -2.7613604  H 5.8317295 -3.0619374 -3.2446294  H 7.4506825 -3.3489864 -2.6055154  H 5.8392535 -4.8735554 -1.4800704  H 6.4260515 -3.6908394 -0.3096754  H 3.7784125 -3.6214004 -1.8404954  H 3.9652325 -3.8890094 -0.1267434  H 7.3407765 -1.4103484 -1.1674954  C 3.6219405 -2.8871324 2.2999486  C 3.8513565 -4.0828934 3.2204966  C 2.6352255 -4.2943384 4.1172486  C 1.4191065 -4.6418164 3.2625566  H 4.4514755 -2.7815564 1.5965716  H 4.7554945 -3.9197594 3.8159716  H 2.8167565 -5.0886724 4.8483986  H 2.4400005 -3.3733524 4.6820006  H 1.5365435 -5.6644024 2.8768656  H 0.5078015 -4.6474344 3.8704146  H 3.6298665 -1.9660154 2.9011946  O 1.7510565 0.6040616 0.2677226  O 0.5404665 -1.1361604 -1.1119914  P 0.4809365 0.4176736 -0.7179284  O -0.7265305 0.4760156 0.2930376  O 0.4867325 1.2808666 -1.9114384  C 3.6510705 2.4296416 -0.8084954  C 3.5771345 3.2220506 0.3815836  C 3.5187035 3.0398726 -2.0314014  C 3.7046105 2.6492556 1.6745826  C 3.3810135 4.6295756 0.2702036  C 3.3041375 4.4328096 -2.1341524  H 3.5528085 2.4339056 -2.9321134  C 3.6346045 3.4250856 2.8036766  H 3.8601585 1.5783876 1.7614986  C 3.3296655 5.4044186 1.4601336  C 3.2327895 5.2322686 -1.0201084  H 3.1843285 4.8767876 -3.1188014  C 3.4471455 4.8204676 2.6960826  H 3.7300375 2.9672116 3.7833736  H 3.1892705 6.4785056 1.3860726  H 3.4004775 5.4304976 3.5929456  C -1.6867185 -2.5922714 -0.0963734  C -2.0349515 -2.9366024 -1.4416934  C -2.6542535 -2.0897074 0.7402436  C -1.0846385 -3.4668794 -2.3538664  C -3.3802645 -2.7594594 -1.8782304  C -3.9850325 -1.9144934 0.3008496  H -2.3812075 -1.7997924 1.7510876  C -1.4385325 -3.7885124 -3.6390804  H -0.0626795 -3.6165674 -2.0196534  C -3.7112335 -3.1010474 -3.2170234  C -4.3660285 -2.2493114 -0.9750174  H -4.7208505 -1.5016534 0.9876856  C -2.7670675 -3.5987324 -4.0782564  H -0.6961225 -4.1883554 -4.3228804  H -4.7296135 -2.9571424 -3.5633194  H -3.0391265 -3.8486304 -5.0992904  C 2.9831555 6.7117026 -1.1495024  H 2.0575405 7.0041786 -0.6399164  H 3.7978565 7.2979406 -0.7100654  H 2.8921765 6.9948556 -2.2005504  C -5.7989195 -2.0935384 -1.4097834  H -5.9126695 -1.3341324 -2.1914984  H -6.2020325 -3.0358444 -1.7975154  H -6.4117195 -1.7853204 -0.5585314  H 4.0208945 -4.9863574 2.6181636 | **INT2’-B**  C 3.5961880 1.9960057 -0.2425832  O 2.8037070 1.9653757 -1.3539682  C 1.5747350 2.3245597 -0.9016512  C 1.6504200 2.5585537 0.4412738  N 2.9606810 2.3308177 0.8337108  C 5.0209590 1.7085787 -0.3835062  C 5.8098020 1.6993027 0.7681108  C 5.5793040 1.4355507 -1.6311762  C 7.1701090 1.4332637 0.6577248  H 5.3714690 1.9103987 1.7369618  C 6.9395520 1.1422527 -1.7179142  H 4.9829860 1.4614307 -2.5363032  C 7.7538040 1.1524457 -0.5824652  H 8.8055030 0.9118877 -0.6527402  C 0.5877590 3.0388877 1.3752388  C 1.0846320 4.1956397 2.2422248  H 0.2578750 2.2152177 2.0201198  H -0.2882090 3.3392247 0.7893308  H 0.3049050 4.5215387 2.9380688  H 1.9620010 3.8879417 2.8177868  H 1.3693350 5.0513907 1.6214258  O 0.6150300 2.3988107 -1.8168462  C 2.0954560 -1.1053173 -1.8747612  O 0.9639830 -0.9487313 -2.3120062  C 3.2233070 -1.3934973 -2.8278122  H 2.8420430 -1.3656153 -3.8480252  H 4.0279520 -0.6639383 -2.6902942  H 3.6427720 -2.3807683 -2.6064222  C 2.4177840 -1.0160983 -0.4166692  C 1.4232490 -0.6512883 0.4332248  C 1.4209050 -0.4748143 1.8776938  C 0.1685210 -0.3038073 2.4918908  C 2.5758110 -0.4651383 2.6757088  C 0.0668320 -0.1731403 3.8706248  H -0.7247210 -0.2787623 1.8720928  C 2.4688850 -0.3285163 4.0539318  H 3.5520150 -0.5438453 2.2113128  C 1.2182100 -0.1927313 4.6557728  H -0.9085100 -0.0475293 4.3307178  H 3.3674820 -0.3172833 4.6630198  H 1.1435430 -0.0869123 5.7341488  H 0.4590680 -0.4810313 -0.0373882  C 3.7655410 -1.3217633 -0.0682662  C 4.9428060 -1.5777673 0.0785138  C 6.3388460 -1.8407533 0.2276088  C 6.9710820 -1.6756913 1.4678228  C 7.0953770 -2.2377663 -0.8851442  C 8.3344020 -1.9108813 1.5908248  H 6.3881840 -1.3429643 2.3210488  C 8.4579570 -2.4750843 -0.7527662  H 6.6050240 -2.3465863 -1.8480302  C 9.0820360 -2.3108283 0.4835418  H 8.8157070 -1.7768193 2.5551518  H 9.0356340 -2.7916443 -1.6165022  H 10.1466340 -2.4999353 0.5856388  O 7.3988230 0.8486727 -2.9655282  O 7.8728390 1.4387127 1.8244408  C 8.7720550 0.5667347 -3.1065722  H 9.0607580 -0.3093173 -2.5125142  H 8.9315390 0.3586967 -4.1649772  H 9.3889950 1.4236747 -2.8084332  C 9.2795350 1.4043517 1.7394328  H 9.6472280 1.5181697 2.7600028  H 9.6355400 0.4506527 1.3308898  H 9.6581700 2.2278257 1.1211858  H -0.2321070 2.1414517 -1.3818442  H -0.5757010 -0.8999653 -1.9473472  C -5.3347290 0.0361897 -0.0937002  C -4.5734590 0.9705887 -0.7945272  C -4.7577600 2.3494907 -0.6960322  C -5.8296960 2.7719687 0.0873258  C -6.6235190 1.8780387 0.8093868  C -6.3490550 0.5005777 0.7647838  H -6.0286650 3.8389267 0.1664578  C -4.9446130 -1.4037073 -0.2039092  C -5.8120110 -2.4073483 -0.6754672  C -3.6300750 -1.7533373 0.1170298  C -5.3687530 -3.7394083 -0.6997612  C -3.1580790 -3.0644903 0.0856518  C -4.0642740 -4.0460693 -0.3049362  H -3.7302490 -5.0815473 -0.3302142  C -7.7700710 2.4338047 1.6336318  C -8.2151790 1.5032907 2.7590148  C -8.3868990 0.0849887 2.2221588  C -7.0471910 -0.4495013 1.7217728  H -7.4878850 3.4145047 2.0321688  H -7.4600670 1.4960697 3.5559088  H -9.1461840 1.8731227 3.2004568  H -8.7833700 -0.5813593 2.9949988  H -9.1139910 0.0915167 1.3980768  H -6.3783480 -0.5960763 2.5826338  H -7.1703150 -1.4398123 1.2776388  H -8.6223380 2.6108007 0.9622498  C -7.1582750 -2.0364483 -1.2721872  C -8.1341730 -3.2092723 -1.3192412  C -7.4659570 -4.4161623 -1.9720672  C -6.2810770 -4.8750123 -1.1250362  H -7.6017350 -1.1858523 -0.7489692  H -8.4475990 -3.4725183 -0.2993092  H -9.0354670 -2.9171343 -1.8675972  H -8.1760190 -5.2400623 -2.0966942  H -7.1188030 -4.1348303 -2.9747982  H -6.6626530 -5.3683583 -0.2197642  H -5.6941120 -5.6304323 -1.6589162  H -6.9742790 -1.6845663 -2.2978242  O -3.5422500 0.4891747 -1.5966022  O -2.7262880 -0.7364243 0.4287208  P -2.1819860 0.1069557 -0.8398572  O -1.5774210 -0.8937323 -1.8723222  O -1.3222490 1.2099907 -0.3575312  C -3.7818960 3.2606057 -1.3530542  C -2.9702300 4.1588057 -0.5839132  C -3.5669280 3.1574977 -2.7064782  C -3.1127620 4.3035717 0.8233218  C -1.9554810 4.9144967 -1.2450622  C -2.5501550 3.8945437 -3.3483422  H -4.1707420 2.4673537 -3.2882642  C -2.3451570 5.1961637 1.5280668  H -3.8382320 3.6893397 1.3453348  C -1.1704870 5.8200477 -0.4807882  C -1.7355630 4.7504507 -2.6495882  H -2.3964990 3.7626577 -4.4158922  C -1.3729010 5.9770697 0.8663928  H -2.4759080 5.2934487 2.6018598  H -0.3970260 6.3968367 -0.9771832  H -0.7671640 6.6790047 1.4314318  C -1.7524130 -3.3659183 0.4838468  C -0.7534020 -3.7287873 -0.4758252  C -1.4006210 -3.2605983 1.8079808  C -1.0455380 -3.8284003 -1.8640422  C 0.5827470 -3.9660183 -0.0356272  C -0.0811450 -3.5146333 2.2389398  H -2.1520230 -2.9639233 2.5353748  C -0.0685760 -4.1464923 -2.7719312  H -2.0560340 -3.6197983 -2.2008912  C 1.5724110 -4.2855043 -1.0054172  C 0.9107370 -3.8543603 1.3528858  H 0.1611720 -3.3983123 3.2923378  C 1.2560850 -4.3786043 -2.3378592  H -0.3088160 -4.2059943 -3.8287722  H 2.5960290 -4.4494063 -0.6800572  H 2.0258310 -4.6243463 -3.0642492  C -0.6116240 5.4743597 -3.3405262  H -0.6933450 6.5608177 -3.2270472  H 0.3540080 5.1624327 -2.9276632  H -0.6099330 5.2451487 -4.4085082  C 2.3215050 -4.0609793 1.8340568  H 2.6931660 -5.0613133 1.5846128  H 3.0051460 -3.3325423 1.3815888  H 2.3748380 -3.9367473 2.9183928 |
| **TS2’-B-*SR***  C -3.6634753 1.5189263 -0.8265299  O -2.7093593 1.4951333 -1.8308469  C -1.5317753 1.5128553 -1.1940749  C -1.7782533 1.4802453 0.2063691  N -3.1672283 1.5744653 0.3548421  C -5.0712123 1.4403033 -1.2024419  C -6.0119773 1.4220163 -0.1735499  C -5.4521433 1.3303223 -2.5389589  C -7.3606633 1.2985673 -0.4939079  H -5.7036993 1.4915933 0.8633211  C -6.8067673 1.2062353 -2.8406029  H -4.7230163 1.3273583 -3.3409829  C -7.7722773 1.1882743 -1.8276129  H -8.8213953 1.0920613 -2.0697499  C -0.8315423 2.0068223 1.2621911  C -1.5516203 2.8445563 2.3144071  H -0.0614113 2.6079383 0.7660201  H -0.2927013 1.1920083 1.7537191  H -0.8257253 3.2084663 3.0480071  H -2.0490813 3.7050483 1.8556001  H -2.3147543 2.2566343 2.8332891  O -0.4813323 1.4742873 -1.9051079  C -1.6542703 -0.9625717 2.9203791  O -0.3619153 -0.9228817 3.1089261  C -2.4067533 -1.1420347 4.2064431  H -2.9520933 -2.0910727 4.1928051  H -1.7054113 -1.1309737 5.0403641  H -3.1505043 -0.3493847 4.3278651  C -2.3348953 -0.9007877 1.7044321  C -1.6377783 -0.7359187 0.4601051  C -2.0604053 -1.2388277 -0.8469149  C -1.0360433 -1.4921967 -1.7821819  C -3.3977593 -1.3973117 -1.2531819  C -1.3402423 -1.8713897 -3.0806539  H 0.0054217 -1.4059917 -1.4726419  C -3.6943083 -1.7716067 -2.5595439  H -4.2011963 -1.1996877 -0.5568059  C -2.6713373 -2.0023427 -3.4759759  H -0.5353473 -2.0651607 -3.7824799  H -4.7328393 -1.8635157 -2.8641659  H -2.9095603 -2.2856987 -4.4969679  H -0.5557913 -0.6981297 0.5512461  C -3.7576673 -1.0260637 1.7728351  C -4.9630953 -1.1436957 1.8546771  C -6.3854393 -1.3076617 1.8721891  C -7.0433323 -1.8159507 0.7411891  C -7.1383703 -0.9629247 3.0025601  C -8.4206363 -1.9887477 0.7520471  H -6.4620983 -2.0754027 -0.1388739  C -8.5171263 -1.1399147 3.0066781  H -6.6310643 -0.5581547 3.8726371  C -9.1617443 -1.6579497 1.8862981  H -8.9178953 -2.3905927 -0.1263649  H -9.0895533 -0.8749477 3.8904631  H -10.2383523 -1.8021287 1.8947671  O -7.1073283 1.0994113 -4.1623349  O -8.2158523 1.2879883 0.5585481  C -8.4637683 0.9762923 -4.5235949  H -9.0437333 1.8521153 -4.2076069  H -8.4813913 0.9051293 -5.6111109  H -8.9123863 0.0718323 -4.0937979  C -9.5969043 1.2305713 0.2886611  H -10.0921073 1.2320623 1.2600931  H -9.9239333 2.1003463 -0.2954229  H -9.8630253 0.3089423 -0.2434869  H 0.2381047 -0.8571457 2.3036561  H 0.4208917 1.3320713 -1.3003719  C 5.2843757 0.4798063 -0.4015929  C 4.3886857 1.3079103 0.2759161  C 4.4126947 2.6992403 0.1751231  C 5.3736417 3.2508003 -0.6659009  C 6.2499797 2.4599433 -1.4146029  C 6.1966037 1.0617123 -1.3041419  H 5.4238777 4.3339543 -0.7601549  C 5.1801257 -0.9976657 -0.1868309  C 6.2368687 -1.7699247 0.3346901  C 3.9608157 -1.6228257 -0.4599989  C 6.0771547 -3.1592807 0.4516291  C 3.7752127 -3.0007497 -0.3394299  C 4.8614897 -3.7503387 0.0973441  H 4.7453297 -4.8280197 0.1952451  C 7.2569857 3.1500873 -2.3162489  C 7.7979697 2.2447833 -3.4196469  C 8.2272067 0.9064953 -2.8253219  C 7.0189937 0.1856863 -2.2336599  H 6.8031177 4.0510653 -2.7436329  H 7.0182567 2.0707313 -4.1726009  H 8.6334407 2.7364053 -3.9286069  H 8.6985507 0.2732843 -3.5841369  H 8.9762197 1.0803633 -2.0401649  H 6.3575327 -0.1332247 -3.0525109  H 7.3282157 -0.7360757 -1.7349959  H 8.0982437 3.4951243 -1.6982359  C 7.4858477 -1.0939787 0.8748911  C 8.6751847 -2.0427317 1.0033311  C 8.2577037 -3.3076337 1.7479861  C 7.2001147 -4.0548867 0.9400301  H 7.7549667 -0.2223287 0.2734201  H 9.0423587 -2.3138037 0.0034541  H 9.4963647 -1.5355997 1.5201881  H 9.1176417 -3.9599607 1.9319971  H 7.8478537 -3.0286627 2.7276111  H 7.6826607 -4.5171037 0.0669981  H 6.7769617 -4.8785697 1.5256421  H 7.2376007 -0.6955527 1.8695781  O 3.4137467 0.7115173 1.0581311  O 2.8978617 -0.8421117 -0.8771099  P 2.1600367 0.0875203 0.2393611  O 1.3760457 -0.7338837 1.2072241  O 1.4918337 1.1747853 -0.5614639  C 3.4657977 3.5327633 0.9742551  C 2.3093917 4.1383503 0.3876711  C 3.6993637 3.7013743 2.3171341  C 1.9886227 3.9639763 -0.9849189  C 1.4273907 4.9028283 1.2085041  C 2.8342117 4.4768283 3.1204841  H 4.5655357 3.2264513 2.7690011  C 0.8417567 4.4952753 -1.5177329  H 2.6479517 3.3597393 -1.5987819  C 0.2566547 5.4544413 0.6199191  C 1.7167207 5.0799453 2.5983951  H 3.0608437 4.5962193 4.1768741  C -0.0364483 5.2481813 -0.7048939  H 0.5999787 4.3245353 -2.5621129  H -0.4215053 6.0373333 1.2354271  H -0.9436403 5.6653943 -1.1319619  C 2.4523107 -3.6037467 -0.6756049  C 1.4636857 -3.8222037 0.3351671  C 2.1528677 -3.9016807 -1.9822079  C 1.7317387 -3.5741087 1.7072151  C 0.1671767 -4.2779417 -0.0432459  C 0.8822197 -4.4038497 -2.3435899  H 2.9014207 -3.7319237 -2.7510539  C 0.7589487 -3.7414687 2.6580851  H 2.7170747 -3.2168377 1.9890931  C -0.8270723 -4.4120857 0.9637921  C -0.1136863 -4.5756307 -1.4137659  H 0.6800447 -4.6294217 -3.3880469  C -0.5398083 -4.1506387 2.2797121  H 0.9751197 -3.5280537 3.7001431  H -1.8276513 -4.7254597 0.6833131  H -1.3129953 -4.2605867 3.0355801  C 0.8141307 5.9135793 3.4705161  H 0.7555837 6.9469893 3.1107401  H -0.2081093 5.5192273 3.4904161  H 1.1863907 5.9348303 4.4974851  C -1.4887203 -5.0264767 -1.8277239  H -1.7780713 -5.9564947 -1.3256729  H -2.2387053 -4.2651917 -1.5780419  H -1.5306373 -5.1928757 -2.9071539 | **TS2’-B-*RS***  C -4.7052316 -0.7307912 -0.0252675  O -3.6454356 -0.9943332 -0.8502605  C -2.5429296 -0.4681112 -0.1990635  C -3.0504636 0.2173658 0.9347495  N -4.4013816 -0.0690702 1.0417365  C -6.0337516 -1.1663572 -0.4462015  C -7.1194646 -0.8641602 0.3783985  C -6.2134386 -1.8349412 -1.6568455  C -8.3953686 -1.2514902 -0.0157965  H -6.9728686 -0.3297402 1.3101545  C -7.5002306 -2.2148952 -2.0361315  H -5.3814886 -2.0507742 -2.3168015  C -8.6015486 -1.9316652 -1.2232845  H -9.5963916 -2.2284902 -1.5238715  C -2.2229256 0.7239028 2.0788755  C -3.0071186 0.7545958 3.3884785  H -1.3396126 0.0797428 2.1771195  H -1.8333416 1.7261358 1.8602655  H -2.3736326 1.1188058 4.2023115  H -3.3717516 -0.2437402 3.6489815  H -3.8792636 1.4086518 3.3019915  O -1.4178466 -0.5868362 -0.7012935  C -0.9640636 1.9672808 -1.6694785  O 0.2861584 2.3639868 -1.6386885  C -1.3678166 1.1518028 -2.8595845  H -0.6294346 0.3625358 -3.0189895  H -1.3558856 1.8210138 -3.7294005  H -2.3495196 0.6925778 -2.7694295  C -1.8048476 2.5070298 -0.6835825  C -3.1453256 2.0772228 -0.5402655  C -4.2218946 2.7305188 0.2067375  C -5.5383956 2.4311378 -0.1849215  C -4.0317576 3.5740648 1.3140905  C -6.6288486 2.9425598 0.5041725  H -5.7033976 1.7772448 -1.0380325  C -5.1263336 4.0847668 2.0012745  H -3.0311326 3.8226588 1.6439665  C -6.4251296 3.7708598 1.6047055  H -7.6348166 2.6845098 0.1877465  H -4.9616116 4.7308528 2.8583245  H -7.2736476 4.1710368 2.1514955  H -3.5234516 1.4639628 -1.3536565  C -1.1834476 3.4690878 0.1793235  C -0.5655026 4.2857078 0.8270515  C 0.1655084 5.2472968 1.5963215  C -0.2710416 6.5780088 1.6717565  C 1.3438864 4.8711078 2.2557185  C 0.4627654 7.5148008 2.3889885  H -1.1808396 6.8646938 1.1536795  C 2.0702494 5.8141968 2.9747675  H 1.6841244 3.8429798 2.1816975  C 1.6354584 7.1366288 3.0409465  H 0.1204914 8.5439648 2.4379475  H 2.9820484 5.5153748 3.4840095  H 2.2072724 7.8704148 3.6007045  O -7.5946386 -2.8570492 -3.2295685  O -9.4019286 -0.9228382 0.8362065  C -8.8770256 -3.2359192 -3.6754865  H -9.3441486 -3.9514132 -2.9875365  H -8.7317856 -3.7102392 -4.6458655  H -9.5332166 -2.3642892 -3.7911825  C -10.7134726 -1.3056382 0.4913925  H -11.3516426 -0.9641952 1.3064175  H -10.8007546 -2.3948842 0.3946925  H -11.0367716 -0.8329722 -0.4446305  H 0.8765804 1.7255408 -2.1216725  C 4.1889604 -2.3596072 0.4964685  C 2.9016664 -2.6436902 0.0427885  C 2.0436404 -3.5421852 0.6763335  C 2.5401404 -4.1808392 1.8091755  C 3.8083784 -3.9038542 2.3254065  C 4.6356244 -2.9653252 1.6876435  H 1.9038344 -4.8992612 2.3227385  C 4.9903834 -1.3384682 -0.2475985  C 6.2457994 -1.6120972 -0.8261675  C 4.4469054 -0.0621502 -0.4082615  C 6.9657734 -0.5689982 -1.4283715  C 5.1408184 0.9912918 -1.0037615  C 6.4140194 0.7143368 -1.4869045  H 6.9809554 1.5172438 -1.9537915  C 4.2584594 -4.6473222 3.5699495  C 5.3797394 -3.9379332 4.3242615  C 6.4768284 -3.5253232 3.3477915  C 5.9304234 -2.5148752 2.3426455  H 3.3957964 -4.8124392 4.2247735  H 4.9843494 -3.0421552 4.8207055  H 5.7745194 -4.5934652 5.1072945  H 7.3313664 -3.0915092 3.8772705  H 6.8443594 -4.4142452 2.8162275  H 5.7222774 -1.5685682 2.8631825  H 6.6887834 -2.2736342 1.5941835  H 4.6097064 -5.6448012 3.2696505  C 6.7586494 -3.0400952 -0.8991605  C 8.2653764 -3.1227462 -1.1286135  C 8.6602444 -2.2472452 -2.3147775  C 8.3530744 -0.7841262 -2.0045095  H 6.4718184 -3.6080002 -0.0107985  H 8.7966934 -2.7801402 -0.2295595  H 8.5562504 -4.1648252 -1.2966835  H 9.7224654 -2.3624632 -2.5538395  H 8.0937484 -2.5655192 -3.1996025  H 9.0904914 -0.4130612 -1.2786435  H 8.4717844 -0.1665992 -2.9017095  H 6.2431514 -3.5304882 -1.7377135  O 3.1618164 0.1856618 0.0559725  P 1.9337084 -0.4434892 -0.7787445  O 1.6116844 0.1785628 -2.0851775  O 0.8571384 -0.4514752 0.3444555  C 0.6774964 -3.7842972 0.1345215  C -0.5007576 -3.4617942 0.8818325  C 0.5439774 -4.3056882 -1.1301745  C -0.4408986 -2.8457652 2.1611515  C -1.7816716 -3.7271522 0.3108335  C -0.7258776 -4.5505272 -1.6940465  H 1.4363464 -4.5307082 -1.7071185  C -1.5853396 -2.5422242 2.8558605  H 0.5303864 -2.5946042 2.5754835  C -2.9449356 -3.4033932 1.0597625  C -1.8810566 -4.2796542 -1.0039855  H -0.7873736 -4.9609492 -2.6985275  C -2.8541976 -2.8303772 2.3032005  H -1.5171146 -2.0632532 3.8289245  H -3.9234056 -3.5964292 0.6292655  H -3.7570916 -2.5771942 2.8512735  C 4.4969744 2.3348978 -1.0814335  C 3.7369904 2.7232518 -2.2294215  C 4.5609214 3.1796508 -0.0019515  C 3.6641134 1.9037488 -3.3870085  C 3.0165884 3.9533258 -2.2071875  C 3.8596724 4.4061878 0.0068045  H 5.1350174 2.8837768 0.8718785  C 2.9110334 2.2764248 -4.4706065  H 4.1979124 0.9586838 -3.3932575  C 2.2428644 4.3060488 -3.3455045  C 3.0717634 4.7927828 -1.0489825  H 3.9186624 5.0453008 0.8836155  C 2.1895594 3.4908788 -4.4484945  H 2.8605094 1.6318708 -5.3425635  H 1.6806554 5.2340208 -3.3354955  H 1.5919464 3.7791068 -5.3084005  C -3.2281506 -4.5390302 -1.6221695  H -3.8093386 -5.2606662 -1.0369895  H -3.8154646 -3.6162082 -1.6762195  H -3.1178286 -4.9346262 -2.6345725  C 2.2715664 6.0664048 -0.9829305  H 2.5730324 6.7744528 -1.7636715  H 1.2034214 5.8587348 -1.1157935  H 2.3968874 6.5506738 -0.0099145  H -0.1094106 -0.4921042 0.0031795  O 2.4344804 -1.9494532 -1.0661005 |
| **TS2’-B-*RR***  C 3.5692113 2.0627193 0.0669564  O 2.5718463 2.3674573 -0.8383056  C 1.4159143 2.3246133 -0.1293126  C 1.7622253 1.9440113 1.1932404  N 3.1409813 1.8648493 1.2687844  C 4.9450183 2.0164333 -0.4092856  C 5.9172823 1.5057603 0.4511724  C 5.2763163 2.4432573 -1.6966926  C 7.2339143 1.4146563 0.0131494  H 5.6518213 1.1624473 1.4443224  C 6.6025293 2.3523773 -2.1168206  H 4.5306053 2.8482263 -2.3716456  C 7.5926213 1.8372843 -1.2735846  H 8.6180163 1.7673633 -1.6086756  C 0.9195493 2.1999123 2.4014774  C 1.0555883 3.6582403 2.8557684  H 1.2397833 1.5259333 3.2031914  H -0.1292307 1.9736883 2.1775444  H 0.4581423 3.8422703 3.7547064  H 2.1009553 3.8905793 3.0798554  H 0.7070763 4.3399893 2.0734434  O 0.3244643 2.5676643 -0.7099506  C 2.3674973 -0.3787577 -1.7366656  O 1.3234123 0.0460663 -2.3639086  C 3.5465743 -0.5670297 -2.6379246  H 3.2196573 -0.4860787 -3.6745216  H 4.3002013 0.2018713 -2.4235406  H 4.0108023 -1.5380647 -2.4545516  C 2.4678203 -0.6164397 -0.3432356  C 1.3997273 -0.3233797 0.5340994  C 1.2555393 -0.7675627 1.9220004  C -0.0440937 -0.8235697 2.4496204  C 2.3356943 -1.0848807 2.7623624  C -0.2643847 -1.2336067 3.7587844  H -0.8879007 -0.5499737 1.8246444  C 2.1091493 -1.5034757 4.0675284  H 3.3497443 -0.9755027 2.4013494  C 0.8101823 -1.5884747 4.5695184  H -1.2793337 -1.2775357 4.1423804  H 2.9544373 -1.7496467 4.7034334  H 0.6411523 -1.9113557 5.5927054  H 0.4528063 -0.1009467 0.0545834  C 3.7378813 -1.1178947 0.0747104  C 4.8697943 -1.4953497 0.2985254  C 6.2220513 -1.8765107 0.5634284  C 6.6457603 -2.1598407 1.8686274  C 7.1490763 -1.9369617 -0.4883996  C 7.9708943 -2.4983507 2.1148244  H 5.9277363 -2.1079937 2.6818274  C 8.4679223 -2.2895447 -0.2354926  H 6.8212403 -1.6979537 -1.4957546  C 8.8845803 -2.5681417 1.0658544  H 8.2904013 -2.7130427 3.1299874  H 9.1764933 -2.3463597 -1.0571836  H 9.9174793 -2.8411707 1.2605724  O 6.8479223 2.7917423 -3.3799886  O 8.1146653 0.8871253 0.9010694  C 8.1765453 2.7528323 -3.8471176  H 8.5614583 1.7256753 -3.8772036  H 8.1528183 3.1583743 -4.8585656  H 8.8374923 3.3677443 -3.2240366  C 9.4744233 0.8477813 0.5372784  H 9.9957073 0.3846353 1.3754034  H 9.6320263 0.2361843 -0.3595916  H 9.8695623 1.8574283 0.3659944  H -0.6950947 2.0933083 -0.1580646  H 0.4139313 0.0483923 -1.9130006  C -5.2262377 -0.1939717 -0.2385996  C -4.5664997 0.9587123 -0.6679106  C -4.9686367 2.2423373 -0.3067836  C -6.0969047 2.3406813 0.5033654  C -6.7726077 1.2171663 0.9821914  C -6.3213697 -0.0671707 0.6370404  H -6.4374827 3.3311283 0.7989554  C -4.6522427 -1.5192187 -0.6243456  C -5.3589977 -2.4841497 -1.3649846  C -3.3310667 -1.7836227 -0.2545296  C -4.7569537 -3.7280457 -1.6113236  C -2.7109147 -3.0109497 -0.4823526  C -3.4628427 -3.9747697 -1.1496756  H -3.0083197 -4.9459137 -1.3360886  C -7.9833767 1.4257063 1.8721594  C -8.3076777 0.2123473 2.7395814  C -8.3118037 -1.0477727 1.8786754  C -6.9195307 -1.2947797 1.3028054  H -7.8265507 2.3149973 2.4925194  H -7.5511877 0.1064643 3.5280554  H -9.2734987 0.3547833 3.2349804  H -8.6286357 -1.9197947 2.4598944  H -9.0348227 -0.9257057 1.0603774  H -6.2422227 -1.5891767 2.1180484  H -6.9361167 -2.1466707 0.6192394  H -8.8509357 1.6468013 1.2343094  C -6.7001317 -2.1411527 -1.9881536  C -7.5164767 -3.3765497 -2.3597716  C -6.6616747 -4.3367857 -3.1829826  C -5.4882507 -4.8375257 -2.3441666  H -7.2764497 -1.4760547 -1.3398626  H -7.8588967 -3.8837347 -1.4467786  H -8.4107107 -3.0751177 -2.9147666  H -7.2542067 -5.1863487 -3.5376926  H -6.2863747 -3.8112187 -4.0706626  H -5.8646787 -5.5541987 -1.6005366  H -4.7764697 -5.3905797 -2.9668406  H -6.4988007 -1.5597177 -2.8996016  O -3.4314337 0.8072803 -1.4571776  O -2.6016387 -0.7568457 0.3381154  P -2.0765097 0.3831553 -0.6926746  O -1.1101827 -0.1384847 -1.6978296  O -1.5973417 1.4925933 0.2525994  C -4.2003467 3.4409483 -0.7439166  C -3.4157657 4.1875883 0.1919214  C -4.2337407 3.8337463 -2.0586986  C -3.2888227 3.7910393 1.5505514  C -2.7074407 5.3402623 -0.2565736  C -3.5276747 4.9755783 -2.4966886  H -4.8137307 3.2550833 -2.7720476  C -2.5206817 4.5109043 2.4290914  H -3.7925317 2.8883203 1.8804714  C -1.9291657 6.0684493 0.6833704  C -2.7783137 5.7324813 -1.6306336  H -3.5790887 5.2578683 -3.5449426  C -1.8405927 5.6703323 1.9929344  H -2.4278697 4.1862933 3.4613364  H -1.3902927 6.9510423 0.3540724  H -1.2353877 6.2373863 2.6944094  C -1.3330007 -3.3140737 0.0081024  C -0.2115977 -3.3626997 -0.8804206  C -1.1488867 -3.6304187 1.3319784  C -0.3354927 -3.0310097 -2.2563506  C 1.0632103 -3.7603667 -0.3766436  C 0.1206023 -4.0008417 1.8299884  H -1.9985537 -3.5955987 2.0092044  C 0.7391853 -3.1253847 -3.1036676  H -1.2933437 -2.6793277 -2.6240626  C 2.1537043 -3.8497387 -1.2826806  C 1.2208823 -4.0665007 1.0131504  H 0.2281553 -4.2170047 2.8898724  C 1.9934483 -3.5555097 -2.6143146  H 0.6287653 -2.8606417 -4.1507126  H 3.1284763 -4.1501257 -0.9107376  H 2.8350113 -3.6437337 -3.2959336  C -2.0292207 6.9434223 -2.1208116  H -2.3371937 7.8500793 -1.5882846  H -0.9494517 6.8288303 -1.9749406  H -2.2099357 7.1004663 -3.1866896  C 2.5686183 -4.4451307 1.5681264  H 2.9462553 -5.3688877 1.1146874  H 3.3084223 -3.6581497 1.3793244  H 2.5049613 -4.5966067 2.6486344 | **TS2’-B-*SS***  C -4.8749126 0.1315025 0.0087664  O -4.0275256 0.5700045 0.9955854  C -2.7886006 0.4924215 0.4689044  C -2.9209276 0.0345455 -0.8639426  N -4.2675796 -0.2119395 -1.0790856  C -6.2962506 0.0456315 0.3215574  C -7.1678846 -0.4257275 -0.6611816  C -6.7639406 0.4337195 1.5765334  C -8.5265236 -0.5101035 -0.3747376  H -6.7987986 -0.7286395 -1.6343196  C -8.1284846 0.3430465 1.8451404  H -6.0932426 0.8120035 2.3389194  C -9.0225506 -0.1274265 0.8779254  H -10.0793766 -0.1966375 1.0940454  C -1.8490706 -0.5693185 -1.7094116  C -2.3893626 -1.1326445 -3.0195016  H -1.0564026 0.1631405 -1.9027716  H -1.3561606 -1.3685805 -1.1388936  H -1.5717146 -1.5561335 -3.6091976  H -2.8912976 -0.3575445 -3.6103696  H -3.1228896 -1.9207765 -2.8315556  O -1.8303406 0.8795565 1.2022564  C -0.8771626 2.3697275 -3.0654276  O 0.4155084 2.3647275 -3.2492946  C -1.6223846 2.2103345 -4.3526186  H -1.4455416 1.2039315 -4.7455536  H -1.1890846 2.9146995 -5.0697706  H -2.6910586 2.3977605 -4.2765936  C -1.4396446 2.5957225 -1.7983126  C -2.8244326 2.3830345 -1.5981666  C -3.7077416 2.9784515 -0.6144806  C -5.0908406 2.8700525 -0.8535856  C -3.2722976 3.6099465 0.5695184  C -6.0154146 3.3741265 0.0548444  H -5.4361226 2.3813835 -1.7611906  C -4.2007476 4.1031725 1.4708724  H -2.2139846 3.6863235 0.7833104  C -5.5715416 3.9843035 1.2219564  H -7.0777116 3.2639575 -0.1404566  H -3.8559626 4.5774655 2.3842384  H -6.2888106 4.3653525 1.9425494  H -3.3547986 1.9798665 -2.4564116  C -0.5345886 3.0398995 -0.7781636  C 0.3122984 3.4419905 -0.0085776  C 1.2701814 3.9353875 0.9396134  C 1.4359784 5.3172845 1.1143664  C 2.0330784 3.0424815 1.7020784  C 2.3495944 5.7952465 2.0454854  H 0.8422964 6.0022455 0.5160364  C 2.9589454 3.5317885 2.6179984  H 1.8889464 1.9760915 1.5765384  C 3.1150684 4.9031875 2.7956344  H 2.4703554 6.8664355 2.1820774  H 3.5615824 2.8357825 3.1927894  H 3.8425294 5.2793175 3.5084414  O -8.5103646 0.7408315 3.0865674  O -9.3129176 -0.9791905 -1.3782416  C -9.8759786 0.6433715 3.4216164  H -10.4945126 1.2729055 2.7699824  H -9.9603126 0.9964945 4.4492904  H -10.2288566 -0.3936445 3.3636244  C -10.6958876 -1.1027335 -1.1348126  H -11.1293286 -1.4964905 -2.0539946  H -11.1511616 -0.1309775 -0.9064896  H -10.8963766 -1.7990315 -0.3112206  H -0.8032046 0.5688335 0.8770374  H 0.8780734 2.0009875 -2.4319656  C 3.2700714 -2.5073905 0.6973494  C 1.9180804 -2.4960025 0.3481884  C 0.9625844 -3.2558945 1.0238324  C 1.4078204 -4.0337505 2.0867474  C 2.7386074 -4.0272765 2.5104584  C 3.6790794 -3.2408565 1.8306674  H 0.6828204 -4.6434485 2.6221134  C 4.2164344 -1.6612355 -0.0914326  C 5.3345704 -2.1876715 -0.7661256  C 3.9539064 -0.2922065 -0.1803886  C 6.2277464 -1.3084055 -1.3964826  C 4.8165844 0.5992965 -0.8178966  C 5.9670234 0.0638675 -1.3894466  H 6.6645834 0.7400375 -1.8801576  C 3.1220104 -4.8926105 3.6958804  C 4.4132624 -4.4442265 4.3748254  C 5.4939994 -4.2162645 3.3218514  C 5.0849014 -3.0811895 2.3860624  H 2.2940184 -4.9105545 4.4130864  H 4.2396334 -3.5070945 4.9197064  H 4.7296574 -5.1919105 5.1096144  H 6.4548174 -3.9787875 3.7904244  H 5.6379454 -5.1394965 2.7429974  H 5.1103054 -2.1323325 2.9420264  H 5.8176694 -2.9666045 1.5843354  H 3.2465144 -5.9275965 3.3464514  C 5.5110794 -3.6900945 -0.9032096  C 6.9415254 -4.0949745 -1.2494626  C 7.4392564 -3.2838135 -2.4424976  C 7.4917144 -1.8030145 -2.0751256  H 5.1711284 -4.2107975 -0.0046706  H 7.5974774 -3.9125415 -0.3864516  H 6.9804774 -5.1685865 -1.4611516  H 8.4284774 -3.6236495 -2.7663276  H 6.7539814 -3.4320605 -3.2873886  H 8.3395654 -1.6367445 -1.3951576  H 7.6897564 -1.1924825 -2.9630706  H 4.8404564 -4.0265015 -1.7076516  O 1.5083324 -1.6798325 -0.6914676  O 2.8036474 0.1925305 0.4111394  P 1.3867794 -0.0886115 -0.3448116  O 1.2718844 0.6130895 -1.6529636  O 0.3792304 0.1176395 0.7612314  C -0.4811106 -3.2268285 0.6542284  C -0.9588446 -3.9068525 -0.5114066  C -1.3800346 -2.5794485 1.4679164  C -0.0735626 -4.5553075 -1.4122226  C -2.3589036 -3.9303855 -0.7845496  C -2.7671656 -2.6041555 1.1890744  H -1.0151416 -2.0314045 2.3320224  C -0.5454006 -5.1976445 -2.5287266  H 0.9918984 -4.5304395 -1.2055096  C -2.8126586 -4.6139785 -1.9444746  C -3.2727336 -3.2684315 0.0974324  H -3.4487926 -2.0846945 1.8600384  C -1.9317886 -5.2300385 -2.7971576  H 0.1478474 -5.6819165 -3.2097156  H -3.8761986 -4.6375705 -2.1605036  H -2.2988036 -5.7418165 -3.6817996  C 4.5097354 2.0574915 -0.9028186  C 5.1413184 2.9922385 -0.0247316  C 3.6413434 2.5193475 -1.8615116  C 6.0183844 2.5697135 1.0093294  C 4.8920544 4.3867585 -0.1891976  C 3.3655044 3.8990255 -1.9925086  H 3.1676634 1.8074685 -2.5293886  C 6.6437074 3.4804045 1.8237904  H 6.1880394 1.5058095 1.1457194  C 5.5709714 5.3010135 0.6591724  C 3.9772404 4.8341675 -1.1938326  H 2.6593834 4.2222185 -2.7539196  C 6.4280014 4.8641075 1.6378334  H 7.3119144 3.1384445 2.6086714  H 5.3974124 6.3655155 0.5342384  H 6.9355344 5.5808425 2.2769734  C -4.7549176 -3.2995455 -0.1692196  H -4.9994596 -2.8135275 -1.1210936  H -5.1311656 -4.3280415 -0.2086326  H -5.2983416 -2.7685785 0.6170664  C 3.7070324 6.3030765 -1.3850316  H 3.3523734 6.7667425 -0.4583876  H 4.6117494 6.8388495 -1.6953686  H 2.9446694 6.4544865 -2.1532486 |
| **INT0-POMe**  C 0.5297636 1.6206443 -0.3347285  O 0.8305966 2.9069323 -0.6873805  C -0.3739374 3.6079133 -0.6993025  C -1.4493484 2.6073263 -0.3209055  H -2.1592854 2.4646083 -1.1431475  C 1.6572536 0.6822343 -0.2536585  C 1.4024926 -0.6602937 0.0227655  C 2.9519976 1.1539463 -0.4536585  C 2.4767706 -1.5425717 0.1022255  H 0.3970696 -1.0402067 0.1670185  C 4.0156466 0.2562183 -0.3695275  H 3.1515966 2.1957833 -0.6721285  C 3.7892036 -1.0944317 -0.0908225  H 4.6175116 -1.7864257 -0.0264095  O -0.4275854 4.7710983 -0.9405705  N -0.7016174 1.3712143 -0.1145295  C -2.2261024 3.0562043 0.9223645  C -1.3405524 3.2161953 2.1535075  H -3.0173514 2.3195953 1.0859275  H -2.7090684 4.0058433 0.6684245  H -1.9326124 3.5421963 3.0123145  H -0.8585074 2.2701243 2.4181755  H -0.5584244 3.9666163 1.9888015  O 5.2468826 0.7849093 -0.5745825  O 2.1598006 -2.8322977 0.3720155  C 6.3603136 -0.0776347 -0.4968105  H 7.2348926 0.5416163 -0.6942695  H 6.4493076 -0.5259917 0.5001855  H 6.3015656 -0.8730347 -1.2497885  C 3.2053426 -3.7771867 0.4254735  H 3.9151676 -3.5432917 1.2284545  H 2.7323986 -4.7372237 0.6304385  H 3.7418436 -3.8329697 -0.5296745  P -3.5090594 -0.5879147 -0.3284195  O -3.8118804 0.7769333 -0.7967945  O -4.6339254 -1.1535177 0.6605645  O -2.1492784 -0.8005637 0.4418685  O -3.4201724 -1.6973457 -1.4881595  C -4.5012164 -1.7145117 -2.4226415  H -4.6111394 -0.7346347 -2.8936965  H -4.2560004 -2.4672957 -3.1717475  H -5.4357124 -1.9851247 -1.9195835  C -4.4185754 -2.4235907 1.2772885  H -3.5430364 -2.3828207 1.9300735  H -5.3121114 -2.6417927 1.8618525  H -4.2729814 -3.1982887 0.5180015  H -1.5379364 -0.0071927 0.2851975 | **INT0’-POMe**  C -1.0476905 -0.2496274 0.0207721  O -0.4613235 -1.5062774 0.1814181  C 0.8802005 -1.2948404 0.1815961  C 1.0935115 0.1882246 -0.0301809  H 1.6138325 0.3219456 -0.9886029  C -2.5161995 -0.2305234 0.0122881  C -3.1551625 0.9984626 -0.1376589  C -3.2345305 -1.4146594 0.1486241  C -4.5460195 1.0353486 -0.1498999  H -2.5849135 1.9136276 -0.2451989  C -4.6282025 -1.3597914 0.1331731  H -2.7426395 -2.3726464 0.2637851  C -5.2948455 -0.1406884 -0.0151579  H -6.3753775 -0.1058974 -0.0261569  O 1.6665465 -2.1947624 0.3428431  N -0.2556175 0.7324096 -0.0978599  C 1.9477115 0.8234616 1.0734471  C 2.3939095 2.2254366 0.6805501  H 2.8201595 0.1853196 1.2506791  H 1.3651405 0.8395536 2.0016681  H 3.0124915 2.6715666 1.4643031  H 2.9800255 2.1774246 -0.2427679  H 1.5288095 2.8717516 0.5071341  O -5.2621225 -2.5515204 0.2688561  O -5.1033475 2.2635876 -0.2978999  C -6.6720635 -2.5592514 0.2533831  H -6.9683775 -3.6016794 0.3686411  H -7.0841055 -1.9710874 1.0826811  H -7.0639475 -2.1730204 -0.6955009  C -6.5099325 2.3533606 -0.3374409  H -6.9595465 2.0007306 0.5989441  H -6.7403315 3.4098716 -0.4728609  H -6.9231065 1.7811196 -1.1771919  P 4.7462625 -0.5892564 -0.6501499  O 3.7106215 -0.0837394 -1.5679449  O 6.0371695 -1.1319434 -1.4183979  O 4.3396125 -1.7460354 0.3478541  H 3.3702025 -1.9528014 0.3234861  O 5.3366645 0.4994636 0.3763951  C 5.9434055 1.6545736 -0.2077559  H 5.2922695 2.0867346 -0.9731989  H 6.0932545 2.3727266 0.5980891  H 6.9067955 1.3904716 -0.6557359  C 7.0651455 -1.7711734 -0.6579039  H 6.6860025 -2.6962684 -0.2177209  H 7.8743645 -1.9902664 -1.3539429  H 7.4252935 -1.1094164 0.1365171 |
| **TS1- POMe**  C 0.4129235 1.5324403 -0.1813181  O 0.3583685 2.6446713 -0.8917811  C -1.0026965 3.1725893 -0.7884541  C -1.7446195 2.1659483 -0.0554591  H -2.4576385 1.2495763 -0.8916891  C 1.6294955 0.7261603 -0.1353011  C 1.5606555 -0.5877987 0.3285729  C 2.8264105 1.3020283 -0.5538241  C 2.7376655 -1.3320997 0.3842879  H 0.6248985 -1.0623217 0.6123329  C 3.9920885 0.5405633 -0.4886511  H 2.8686575 2.3199853 -0.9223721  C 3.9581355 -0.7754767 -0.0170961  H 4.8656095 -1.3613387 0.0278099  O -1.2624345 4.2245703 -1.2832971  N -0.7171595 1.2694973 0.4020859  C -2.7815165 2.6205843 0.9527929  C -2.1933545 3.4030943 2.1265659  H -3.3339075 1.7425453 1.3097509  H -3.4997355 3.2390533 0.4048849  H -2.9751525 3.7133903 2.8257899  H -1.4687995 2.7955943 2.6788359  H -1.6827425 4.3027583 1.7678759  O 5.1211705 1.1608683 -0.9054441  O 2.6081435 -2.6002767 0.8379239  C 6.3357475 0.4438453 -0.8517311  H 7.1008185 1.1230943 -1.2262841  H 6.5799215 0.1515823 0.1767239  H 6.3002545 -0.4495727 -1.4866451  C 3.7638235 -3.4068627 0.9073499  H 4.5065875 -2.9813547 1.5929319  H 3.4318645 -4.3721587 1.2877879  H 4.2156645 -3.5418827 -0.0827081  P -2.9897905 -0.9599497 -0.4329711  O -3.0001835 0.2918363 -1.3324251  O -4.5010975 -1.0826447 0.1073299  O -1.9920015 -1.0252657 0.6805399  O -2.8075945 -2.2386297 -1.3831511  C -3.6227525 -2.3138507 -2.5534781  H -3.4155325 -1.4675557 -3.2129371  H -3.3688005 -3.2499837 -3.0509431  H -4.6832985 -2.3139527 -2.2820151  C -4.7679375 -2.1414747 1.0266309  H -4.1147495 -2.0614647 1.8994529  H -5.8111715 -2.0413927 1.3266759  H -4.6145735 -3.1135327 0.5459649  H -0.9896625 0.3445663 0.7990779 | **TS1’- POMe**  C -0.8265854 -0.1639125 0.6334826  O -0.2168594 -1.3901735 0.9030996  C 1.0555636 -1.0984825 1.1776626  C 1.2533546 0.3064805 0.9999096  H 2.0174906 0.2081495 -0.1313014  C -2.2561454 -0.1996615 0.3182966  C -2.8921794 1.0017225 0.0111626  C -2.9503104 -1.4064915 0.3325436  C -4.2517114 0.9866135 -0.2841534  H -2.3432794 1.9359125 -0.0010254  C -4.3122154 -1.4030625 0.0339486  H -2.4615344 -2.3447355 0.5652956  C -4.9753194 -0.2122415 -0.2757184  H -6.0313844 -0.2161095 -0.5069034  O 1.8517406 -2.0568425 1.4109966  N -0.0378774 0.8338155 0.7000736  C 2.1769996 1.1379235 1.8810436  C 2.4886926 2.4925035 1.2502426  H 3.0993106 0.5735645 2.0542796  H 1.6984086 1.2811505 2.8567946  H 3.1217816 3.0886975 1.9125026  H 3.0111396 2.3630495 0.2978086  H 1.5649166 3.0446095 1.0586336  O -4.9220114 -2.6153955 0.0668236  O -4.8051954 2.1916665 -0.5749494  C -6.2935334 -2.6751455 -0.2539274  H -6.5737384 -3.7260935 -0.1860904  H -6.8957754 -2.0919505 0.4536636  H -6.4808484 -2.3137145 -1.2726384  C -6.1811594 2.2341175 -0.8779814  H -6.7874604 1.8815855 -0.0343214  H -6.4135074 3.2801465 -1.0769614  H -6.4144434 1.6353525 -1.7670684  P 4.2169326 -0.5324015 -0.4316784  O 2.9336686 0.0740565 -0.9943454  O 4.8583266 -1.3822485 -1.6234824  O 4.0939696 -1.3470695 0.8401286  H 2.9048326 -1.7539095 1.2331086  O 5.2931516 0.6131985 -0.1188174  C 5.5937786 1.5356125 -1.1694324  H 4.6778816 2.0038685 -1.5406744  H 6.2535846 2.2897895 -0.7416484  H 6.0991836 1.0221805 -1.9932484  C 6.0430046 -2.1293835 -1.3297774  H 5.8542676 -2.8299065 -0.5127994  H 6.3004306 -2.6710525 -2.2391604  H 6.8606616 -1.4557835 -1.0533964 |
| **TS2- POMe-*cis***  C -0.0705194 0.0380724 1.1906529  O 1.2009186 0.1171964 1.5659809  C 1.6993376 -1.2002236 1.7553789  C 0.6197716 -2.0645886 1.3168649  N -0.4772904 -1.2131086 1.1780809  C -0.8365994 1.2526184 0.9531289  C -2.2318595 1.2209864 0.9908389  C -0.1347504 2.4212544 0.6563309  C -2.9328335 2.3961044 0.7293659  H -2.7771974 0.3168334 1.2362059  C -0.8577574 3.5782234 0.3699169  H 0.9490856 2.4370184 0.6137629  C -2.2567395 3.5775144 0.4022319  H -2.8092034 4.4827214 0.1920249  C 0.4230166 -3.5048166 1.7141449  C 1.6070816 -4.0925686 2.4844319  H -0.4860514 -3.5682086 2.3242979  H 0.2298486 -4.1095596 0.8187709  H 1.4058476 -5.1431186 2.7106679  H 1.7682746 -3.5595986 3.4248669  H 2.5320136 -4.0302016 1.9063799  O 2.8276756 -1.3707506 2.1318819  C -0.2698354 -0.5460906 -1.7471971  O -1.1486274 -1.4483536 -1.6796021  C -0.6448574 0.7828674 -2.3469971  H 0.1344556 1.5339884 -2.2193551  H -1.5754944 1.1288054 -1.8822551  H -0.8420434 0.6386114 -3.4147701  C 1.0544396 -0.8069486 -1.2732091  C 1.2883156 -2.1092256 -0.7552031  C 2.5710156 -2.8297236 -0.6731401  C 3.8107946 -2.2308496 -0.4035131  C 2.5306416 -4.2209156 -0.8714461  C 4.9625376 -3.0075396 -0.3280451  H 3.8753256 -1.1626536 -0.2441631  C 3.6822656 -4.9925946 -0.7989741  H 1.5782116 -4.6940606 -1.0982131  C 4.9050535 -4.3847116 -0.5211751  H 5.9122856 -2.5281106 -0.1126341  H 3.6267646 -6.0646086 -0.9612291  H 5.8097646 -4.9822026 -0.4608231  H 0.4495686 -2.7706606 -0.9627551  C 2.0346856 0.2218294 -1.2381781  C 2.8779546 1.0830514 -1.0869451  C 3.9000416 2.0110534 -0.7095611  C 4.5078506 1.8499314 0.5474179  C 4.3090316 3.0597364 -1.5439421  C 5.5134166 2.7196934 0.9495839  H 4.1738396 1.0410044 1.1929519  C 5.3110136 3.9287794 -1.1285791  H 3.8383356 3.1807004 -2.5143101  C 5.9173396 3.7603434 0.1148859  H 5.9801605 2.5874284 1.9208679  H 5.6234605 4.7394814 -1.7798251  H 6.7020976 4.4396884 0.4333739  O -0.1172594 4.6706064 0.0653479  O -4.2813475 2.3135254 0.8371809  C -0.7933304 5.8724814 -0.2310221  H -1.4404954 5.7604874 -1.1097021  H -0.0182554 6.6081484 -0.4439201  H -1.3933144 6.2133714 0.6215749  C -5.0508364 3.4152474 0.4088859  H -6.0926624 3.1041594 0.4873259  H -4.8273524 3.6729724 -0.6329401  H -4.8863534 4.2925794 1.0466829  H -2.5104264 -1.2442666 -1.8459021  H -1.4333544 -1.5135386 0.8854639  O -3.5737214 -1.1946266 -1.7881971  P -4.0883195 -1.6104806 -0.3831481  O -5.0356784 -2.8890736 -0.5626991  O -5.0968145 -0.4158946 -0.0264901  O -3.0920314 -1.8840436 0.6890759  C -5.6885584 -0.4229596 1.2710949  H -4.9209944 -0.3377296 2.0448639  H -6.3473324 0.4436594 1.3174059  H -6.2647034 -1.3417316 1.4251999  C -6.0212635 -2.8534996 -1.5952681  H -5.5453015 -2.7156316 -2.5693021  H -6.5389794 -3.8120666 -1.5635671  H -6.7331904 -2.0412426 -1.4171961 | **TS2- POMe-*trans***  C -0.9771346 -0.9420094 1.2164091  O 0.0895514 -1.7285574 1.2379661  C 1.2467264 -0.9257064 1.5360511  C 0.7321314 0.4009086 1.6600201  N -0.6443546 0.2911446 1.5123561  C -2.3010396 -1.4600444 0.9109331  C -3.3704486 -0.5761164 0.7545281  C -2.4534406 -2.8347454 0.7320341  C -4.6174776 -1.0947904 0.4181871  H -3.2623196 0.4995876 0.8577911  C -3.7105726 -3.3319254 0.3975261  H -1.6159066 -3.5143764 0.8357541  C -4.8017606 -2.4715584 0.2371611  H -5.7745396 -2.8634124 -0.0249249  C 1.3361724 1.5708406 2.3741261  C 2.7694224 1.3124786 2.8395621  H 0.7064674 1.8080156 3.2413731  H 1.3018634 2.4629246 1.7365201  H 3.1629794 2.2064376 3.3318971  H 2.8050564 0.4824386 3.5500031  H 3.4243724 1.0490916 2.0052641  O 2.3383314 -1.4424924 1.5607921  C 2.5469124 3.0763746 -0.3748229  O 1.5634864 3.9015116 -0.3744509  C 3.8809014 3.7168146 -0.1294499  H 3.7560884 4.7947806 -0.0346739  H 4.3246725 3.3048416 0.7830961  H 4.5695425 3.4835006 -0.9467979  C 2.4771764 1.6739506 -0.5925769  C 1.2302754 1.0467456 -0.7107619  C 0.8659034 -0.2358344 -1.2819419  C -0.4974126 -0.3973694 -1.5983949  C 1.7477634 -1.3144114 -1.4974549  C -0.9735876 -1.6000584 -2.1078969  H -1.1834996 0.4332856 -1.4364179  C 1.2608534 -2.5109404 -1.9983859  H 2.7949764 -1.2194604 -1.2423519  C -0.0960536 -2.6616064 -2.2987399  H -2.0321176 -1.7107554 -2.3250709  H 1.9429434 -3.3421644 -2.1460579  H -0.4647706 -3.6096364 -2.6787879  H 0.3807544 1.7155476 -0.6249739  C 3.7038154 0.9419526 -0.5769949  C 4.7266495 0.2961186 -0.4960749  C 5.8187134 -0.6091724 -0.2993569  C 5.5862115 -1.7644074 0.4641441  C 7.0871615 -0.3761074 -0.8453309  C 6.6186524 -2.6715214 0.6695361  H 4.5981035 -1.9212114 0.8898811  C 8.1126485 -1.2891534 -0.6289599  H 7.2576795 0.5185886 -1.4358789  C 7.8805925 -2.4373034 0.1256651  H 6.4373625 -3.5644524 1.2599421  H 9.0950175 -1.1064754 -1.0536309  H 8.6834295 -3.1495634 0.2898691  O -3.7875416 -4.6776414 0.2379571  O -5.6132956 -0.1840734 0.2774201  C -5.0384075 -5.2348414 -0.0976229  H -5.4035195 -4.8500254 -1.0577949  H -4.8775296 -6.3097464 -0.1776719  H -5.7860546 -5.0363374 0.6800781  C -6.9040766 -0.6554744 -0.0362969  H -7.5437546 0.2257176 -0.0802739  H -6.9203595 -1.1641494 -1.0083879  H -7.2797965 -1.3381614 0.7358141  H 0.5442434 3.6221766 -0.5952919  O -1.8875216 2.5852126 1.2516591  P -2.0028656 3.1746566 -0.1251679  O -2.8422146 4.5444136 -0.0973389  O -2.9444606 2.1623666 -0.9729229  O -0.7632666 3.4412146 -0.9561429  H -1.2680436 1.1486456 1.4850061  C -4.0325805 4.5520436 0.6900021  H -4.4688906 5.5458526 0.5875791  H -4.7388705 3.7989476 0.3235341  H -3.7962466 4.3483416 1.7371601  C -3.1393316 2.4491066 -2.3555469  H -2.1860316 2.4118606 -2.8901769  H -3.8189716 1.6882396 -2.7417459  H -3.5855396 3.4411136 -2.4810579 |
| **TS2’- POMe-*cis***  C -0.5473838 -1.4831469 -0.9839211  O 0.3417632 -2.4778509 -0.6493361  C 1.5659752 -1.9280539 -0.7797801  C 1.3825502 -0.5686569 -1.1436021  N 0.0172042 -0.3752639 -1.3208441  C -1.9733378 -1.7808059 -0.9083701  C -2.8697888 -0.7411489 -1.1605891  C -2.4193288 -3.0456579 -0.5348071  C -4.2323048 -0.9751119 -1.0134631  H -2.5092568 0.2370941 -1.4597911  C -3.7905238 -3.2662709 -0.4090741  H -1.7279028 -3.8529319 -0.3235631  C -4.7098198 -2.2376849 -0.6388461  H -5.7704678 -2.4136359 -0.5266891  C 2.3980762 0.3444621 -1.7787211  C 1.7401642 1.5489891 -2.4500141  H 2.9796842 -0.2246489 -2.5144181  H 3.1260272 0.6892031 -1.0379691  H 2.5067922 2.2285081 -2.8332811  H 1.1051812 1.2311391 -3.2810391  H 1.1002512 2.0923071 -1.7484251  O 2.5626752 -2.6579549 -0.5014821  C 2.4987902 2.3824801 0.8800489  O 3.7558792 2.0660491 0.9841139  C 2.2896482 3.8621051 0.7385339  H 3.2528442 4.3684021 0.7927529  H 1.8085542 4.0775051 -0.2215481  H 1.6216302 4.2340231 1.5202579  C 1.3974982 1.5139431 0.9267649  C 1.5670552 0.1083791 1.0897009  C 0.6513922 -0.8145879 1.7433409  C 1.2238442 -1.9769989 2.3030079  C -0.7416368 -0.6490169 1.8087359  C 0.4293562 -2.9324759 2.9128239  H 2.2999382 -2.1156999 2.2381729  C -1.5341958 -1.6167179 2.4183559  H -1.2040998 0.2226731 1.3642779  C -0.9557238 -2.7560939 2.9667529  H 0.8833732 -3.8213039 3.3387269  H -2.6121538 -1.4860429 2.4380839  H -1.5810068 -3.5127369 3.4313669  H 2.5997512 -0.2305919 1.1281919  C 0.1131182 2.1291801 0.8002019  C -0.9465758 2.7043971 0.6635359  C -2.2144608 3.3502501 0.5071729  C -3.3757798 2.5911371 0.3006909  C -2.3090878 4.7490541 0.5527229  C -4.6016828 3.2230081 0.1372149  H -3.3119598 1.5083481 0.2615989  C -3.5400628 5.3723691 0.3916049  H -1.4094138 5.3349461 0.7144889  C -4.6895858 4.6124831 0.1827069  H -5.4882188 2.6204061 -0.0339971  H -3.6027408 6.4556341 0.4282609  H -5.6494088 5.1028791 0.0528719  O -4.1503348 -4.5237859 -0.0432791  O -5.0441208 0.0941571 -1.2421811  C -5.5247728 -4.8114679 0.0781229  H -6.0505658 -4.6564779 -0.8720271  H -5.5890738 -5.8625149 0.3592689  H -5.9956948 -4.1985709 0.8569239  C -6.4376768 -0.1041339 -1.1587201  H -6.8944678 0.8528371 -1.4130441  H -6.7760978 -0.8660969 -1.8712211  H -6.7421678 -0.3940279 -0.1452381  H 4.0367902 1.0788111 1.0074559  O 4.6486602 -0.3023849 0.9834789  P 5.3993002 -0.8078539 -0.2214381  O 6.7993302 -1.4660099 0.1962969  O 5.8060182 0.4853381 -1.0895701  O 4.7159572 -1.8173789 -1.1317121  H 3.5977162 -2.2345209 -0.7945601  C 6.4296832 0.2599771 -2.3536531  H 5.7916862 -0.3622449 -2.9866771  H 6.5745172 1.2388101 -2.8109101  H 7.3984912 -0.2323579 -2.2192931  C 7.6163702 -0.7295889 1.1066539  H 7.0579572 -0.5020779 2.0181189  H 8.4734402 -1.3613329 1.3391389  H 7.9579472 0.2026301 0.6445559 | **TS2’-POMe-*trans***  C -0.4037959 -0.8134093 1.4383268  O 0.4043831 -1.9011953 1.1496028  C 1.6651421 -1.4518233 1.2778328  C 1.6003131 -0.0613623 1.5433438  N 0.2538301 0.2506467 1.7333748  C -1.8492819 -0.9751203 1.3455308  C -2.6492929 0.0968227 1.7398858  C -2.4083219 -2.1291413 0.7998418  C -4.0293019 0.0028297 1.5923678  H -2.2074039 0.9952637 2.1553188  C -3.7903919 -2.1888513 0.6220678  H -1.7972369 -2.9747493 0.5050428  C -4.6143239 -1.1336663 1.0236398  H -5.6837429 -1.1807153 0.8720018  C 2.6924371 0.7672847 2.1516848  C 3.0827961 0.2578487 3.5419888  H 2.3237361 1.7965837 2.2053638  H 3.5791111 0.7673117 1.5082768  H 3.8439001 0.9038107 3.9882978  H 2.2130771 0.2356777 4.2056258  H 3.4980681 -0.7525383 3.4754768  O 2.6059491 -2.2870743 1.1058928  C 0.8336481 -1.5300843 -1.8323922  O 1.9641391 -2.1554113 -1.8747822  C -0.2954069 -2.3233273 -2.4130592  H 0.0688491 -3.3044633 -2.7151372  H -1.1013969 -2.4193973 -1.6778432  H -0.7155269 -1.7956413 -3.2754922  C 0.6143901 -0.2350033 -1.3136982  C 1.6699251 0.4703937 -0.6816622  C 1.7177221 1.9216377 -0.4764092  C 2.9779671 2.5311997 -0.5760952  C 0.5993631 2.7123977 -0.1709702  C 3.1175621 3.9034707 -0.4091132  H 3.8480231 1.9090527 -0.7751032  C 0.7468191 4.0821027 0.0036488  H -0.3690079 2.2458857 -0.0378352  C 1.9998551 4.6824777 -0.1217102  H 4.0979651 4.3615927 -0.4952662  H -0.1209259 4.6868177 0.2482748  H 2.1046021 5.7545087 0.0160128  H 2.6561031 0.0342077 -0.8015252  C -0.7247749 0.2446037 -1.4417432  C -1.9077229 0.4805457 -1.5726182  C -3.3174169 0.6970637 -1.6629352  C -3.9132579 1.8133157 -1.0591382  C -4.1279349 -0.2387003 -2.3236092  C -5.2895409 1.9886157 -1.1191902  H -3.2894929 2.5242257 -0.5260642  C -5.5041909 -0.0546553 -2.3825382  H -3.6663339 -1.1092033 -2.7804332  C -6.0902549 1.0579267 -1.7805152  H -5.7401569 2.8540817 -0.6419822  H -6.1213269 -0.7793563 -2.9062222  H -7.1654119 1.2031417 -1.8323142  O -4.2571899 -3.3217463 0.0349588  O -4.7435839 1.0805217 2.0092778  C -5.6435469 -3.4102223 -0.2074422  H -5.9822379 -2.5983993 -0.8635142  H -5.8037659 -4.3700423 -0.6985862  H -6.2150579 -3.3818473 0.7284948  C -6.1497039 0.9682067 2.0317978  H -6.5187379 1.8957217 2.4707868  H -6.5583199 0.8581987 1.0199008  H -6.4682289 0.1196667 2.6497378  O 4.2570501 -1.1159993 -1.4842772  P 5.2643701 -1.0264683 -0.3736002  O 5.8701271 0.4678897 -0.2858842  O 6.5309171 -1.9267283 -0.7716142  O 4.8139871 -1.3481853 1.0456608  C 7.5817191 -2.0405693 0.1891058  H 8.3175581 -2.7246473 -0.2330382  H 8.0433921 -1.0638273 0.3689648  H 7.1937481 -2.4378423 1.1301468  C 6.4184281 1.0069017 -1.4892302  H 5.6860981 0.9539237 -2.3005612  H 6.6730201 2.0468367 -1.2811692  H 7.3189701 0.4557897 -1.7784652  H 3.6516481 -1.8391433 1.1265588  H 2.8328411 -1.6864463 -1.6093362 |
